# Oligonucleotides Containing 1-Aminomethyl or 1-Mercaptomethyl-2-deoxy-d-ribofuranoses: Synthesis, Purification, Characterization, and Conjugation with Fluorophores and Lipids

**DOI:** 10.1021/acs.bioconjchem.0c00717

**Published:** 2021-02-05

**Authors:** Virginia Martín-Nieves, Carme Fàbrega, Marc Guasch, Susana Fernández, Yogesh S. Sanghvi, Miguel Ferrero, Ramon Eritja

**Affiliations:** † Departamento de Química Orgánica e Inorgánica, Universidad de Oviedo, 33006-Oviedo, Asturias, Spain; ‡ Department of Chemical and Biomolecular Nanotechnology, Institute for Advanced Chemistry of Catalonia (IQAC, CSIC), 08034-Barcelona, Spain; § CIBER-BBN Networking Centre on Bioengineering, Biomaterials and Nanomedicine, 08034-Barcelona, Spain; # Rasayan Inc., 2802 Crystal Ridge Road, Encinitas, California 92024-6615, United States

## Abstract

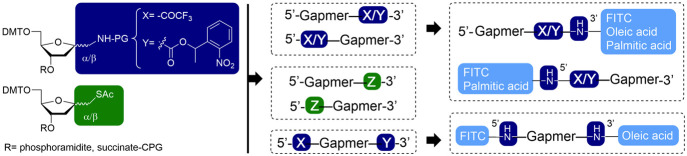

Oligonucleotide conjugates are widely used as therapeutic drugs, gene analysis, and diagnostic tools. A critical step in the biologically relevant oligonucleotide conjugates is the design and synthesis of functional molecules that connect oligonucleotide with ligands. Here, we report the synthesis and application for oligonucleotide functionalization of novel tethers based on aminomethyl and mercaptomethyl sugar derivatives. Starting from a common cyano sugar precursor, three novel phosphoramidites have been prepared in the two α- and β-anomeric forms. The mercaptomethyl sugar was protected with the *S*-acetyl group, while two different protecting groups have been developed for the aminomethyl sugar. These two protecting groups are orthogonal, as they can be removed independently using photolysis or ammonolysis. This combination allowed the introduction of two different ligands in a single oligonucleotide.

## Introduction

Therapeutic oligonucleotides have tremendous potential for treating a variety of diseases if they can reach the target cells successfully upon administration. More recently, this task has been accomplished by covalent conjugation of peptides, lipids, and GalNAc to oligonucleotides.^1,2^ Often, preparation of these conjugates requires the presence of a reactive group such as an amino or thiol group within an oligonucleotide.^3−5^ The therapeutic applications of oligonucleotides have triggered a high demand for oligonucleotide conjugates with enhanced active or passive targeting properties and with the possibility to achieve tissue-specific delivery.^6−8^ Toward this end, researchers are developing nucleosidic and non-nucleosidic phosphoramidite derivatives that enable efficient preparation of oligonucleotide conjugates.^3,9^ Some of the conventional strategies are postsynthetic protocols where a reactive group is added to the oligonucleotide. This approach has been employed for the preparation and screening of several conjugates using a common reactive species.^6^ Some of the most common reactive groups used for the preparation of oligonucleotide conjugates are amino and thiol groups, although a large number of reactions using click chemistry have been also developed.^10^

Amino groups react readily with carboxylic acid derivatives via amide formation as well as with isothiocyanates to form thioureas.^11^ Although nucleobases have amino functions, these groups are aromatic amines and have low reactivity. For this reason, it is possible to use primary alkylamino groups for the selective introduction of ligands to oligonucleotides. Aminoalkylalcohols, such as 6-aminohexanol^6,12^ or 5′-amino-2′,5′-dideoxynucleoside^13^ derivatives, are utilized for the introduction of amino groups at the 5′-end. However, the introduction of amino groups at the 3′-end or at internal positions of oligonucleotides requires the use of aminoalkyldiols such as 2-amino-1,3-propanediol^14^ or 2-aminobutyl-1,3-propanediol derivatives.^15^

On the other hand, thiol groups have a selective reactivity with maleimide and haloacetamide derivatives to form thioethers.^11^ The introduction of thiol groups in oligonucleotides is usually done by preparing 3-mercaptopropanol and 6-mercaptohexanol derivatives protected either by trityl^16^ or disulfide groups.^17,18^

We have recently described the synthesis of novel 1′-homo-*N*-2′-deoxy-α-nucleosides^19^ and 1β-[(thymin-1-yl)acetylaminomethyl]-1,2-dideoxy-d-*erythro*-pentofuranose as model compounds for nucleosides containing an extended link between the ribose and the nucleobase.^20^ These nucleoside derivatives are prepared from the cyano sugar derivatives (**1**α or **1**β) which can be used as common and valuable intermediates for the synthesis of amino (**2**α or **2**β) and thiol (**14**α or **14**β) linkers for the introduction of reactive groups into oligonucleotides. Aminomethyl and mercaptomethyl sugar derivatives are ideal linker molecules, because they are cyclic aminodiol or mercaptodiol compatible with oligonucleotide synthesis. These sugar derivatives can be obtained in a defined stereochemistry as single α- or β-anomers, and they can be conveniently introduced at any position within the oligonucleotide. Additionally, utilization of the 2-deoxyribose framework offers an unique advantage of maintaining normal distance between two nucleosidic units when incorporated in the middle of an oligonucleotide. Here, we describe the synthesis of several solid supports and phosphoramidite derivatives of aminomethyl and mercaptomethyl sugar derivatives and the use of these solid supports and phosphoramidites for the preparation of amino- and mercapto-oligonucleotides. Another objective of the present work is the study of orthogonal protecting groups in order to synthesize oligonucleotide conjugates carrying two or more distinct ligands. Specifically, we studied the base-labile trifluoroacetyl and the photolabile 1-(2-nitrophenyl)ethoxycarbonyl (NPEC) groups for the aminomethyl sugar derivative and the base labile acetyl group for the mercaptomethyl sugar derivative. Several oligonucleotides carrying lipid and fluorescent compounds are prepared to demonstrate the utility of the novel phosphoramidites described in this work.

## Results

### Synthesis of 1-Functionalized 1,2-Dideoxy-d-*erythro*-pentofuranose Phosphoramidites **5α**/**5β**, **8α**/**8β**, and **16α**/**16β**

**Scheme 1:**
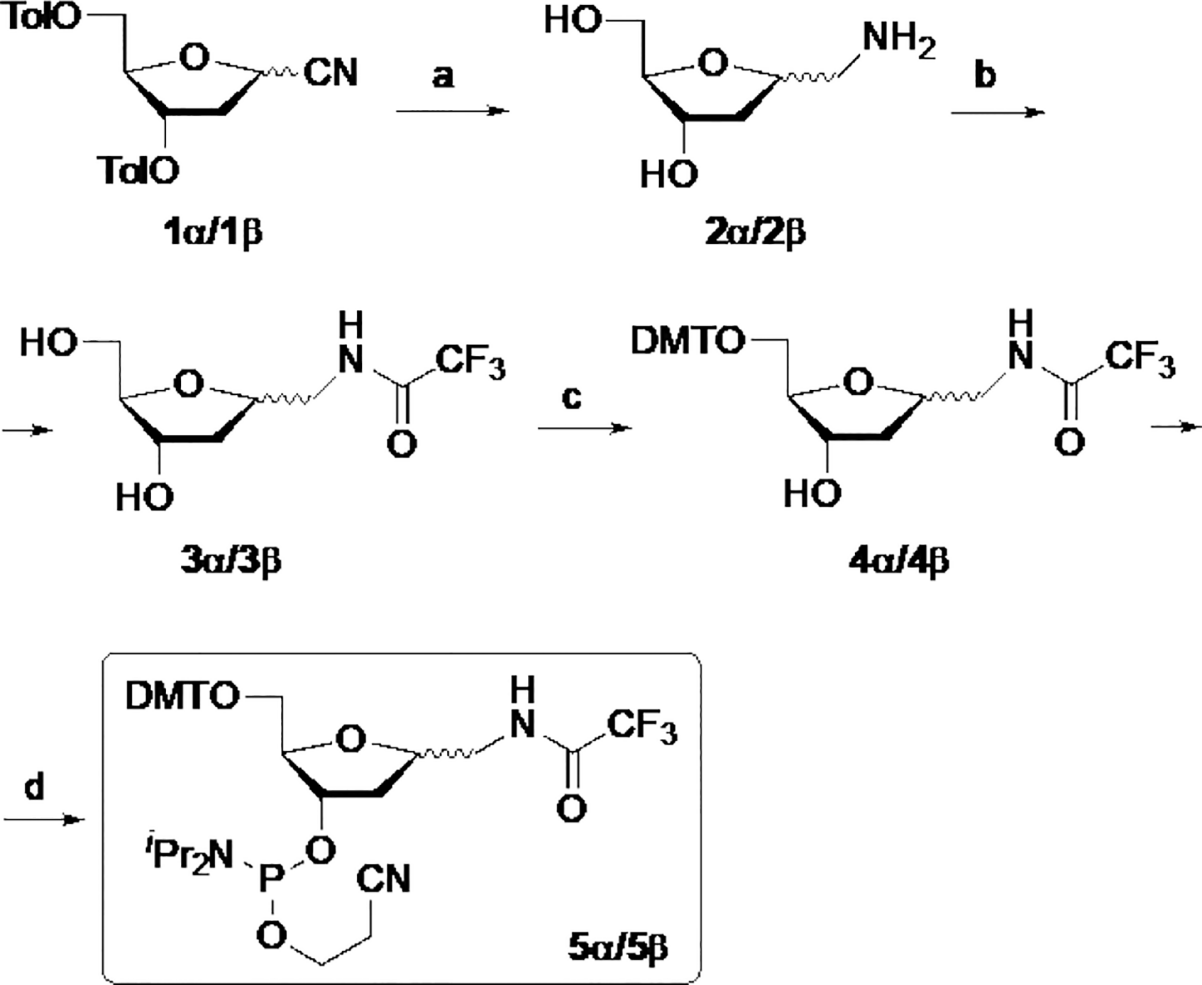
Synthesis of 1-Trifluoroacetylaminomethyl-1,2-dideoxy-d-*erythro*-pentofuranosyl-3-phosphoramidites Reagents and conditions: (a) LiAlH_4_, THF, reflux, 4 h; (b) Ethyl trifluoroacetate, Et_3_N, DMF, 80 °C, 24 h, 70% (**3α**) and 80% (**3β**) two steps; (c) DMTCl, Et_3_N, 1,4-dioxane, 30 °C, 2 h, 65% (**4α**) and 70% (**4β**); (d) 2-Cyanoethyl *N,N*-diisopropylchlorophosphoramidite, ^*i*^Pr_2_NEt, CH_2_Cl_2_, rt, 1 h, 84% (**5α**) and 70% (**5β**).

The synthesis of phosphoramidites was carried out starting from α- or β-cyano sugar derivative **1** (Scheme 1), which is easily accessible to perform on a large scale.^21^

Treatment of the latter with LiAlH_4_ in THF at reflux enabled simultaneous reduction of the cyano group and cleavage of the toluoyl groups, furnishing amino diol **2α**/**2β**. Subsequent protection of the amino group with ethyl trifluoroacetate in Et_3_N and DMF at 80 °C gave **3α** or **3β** in 70% and 80% yield, respectively, from the starting substrate **1α**/**1β**. Next, protection of the primary alcohol with 4,4′-dimethoxytrityl chloride in the presence of Et_3_N and 1,4-dioxane at 30 °C afforded the respective DMT-protected compounds **4α** (65% yield) or **4β** (70% yield). Phosphitylation of **4** with 2-cyanoethyl *N*,*N*-diisopropylchlorophosphoramidite gave the desired phosphoramidite derivatives **5α** or **5β** in 84% and 70% yield, respectively.

**Scheme 2:**
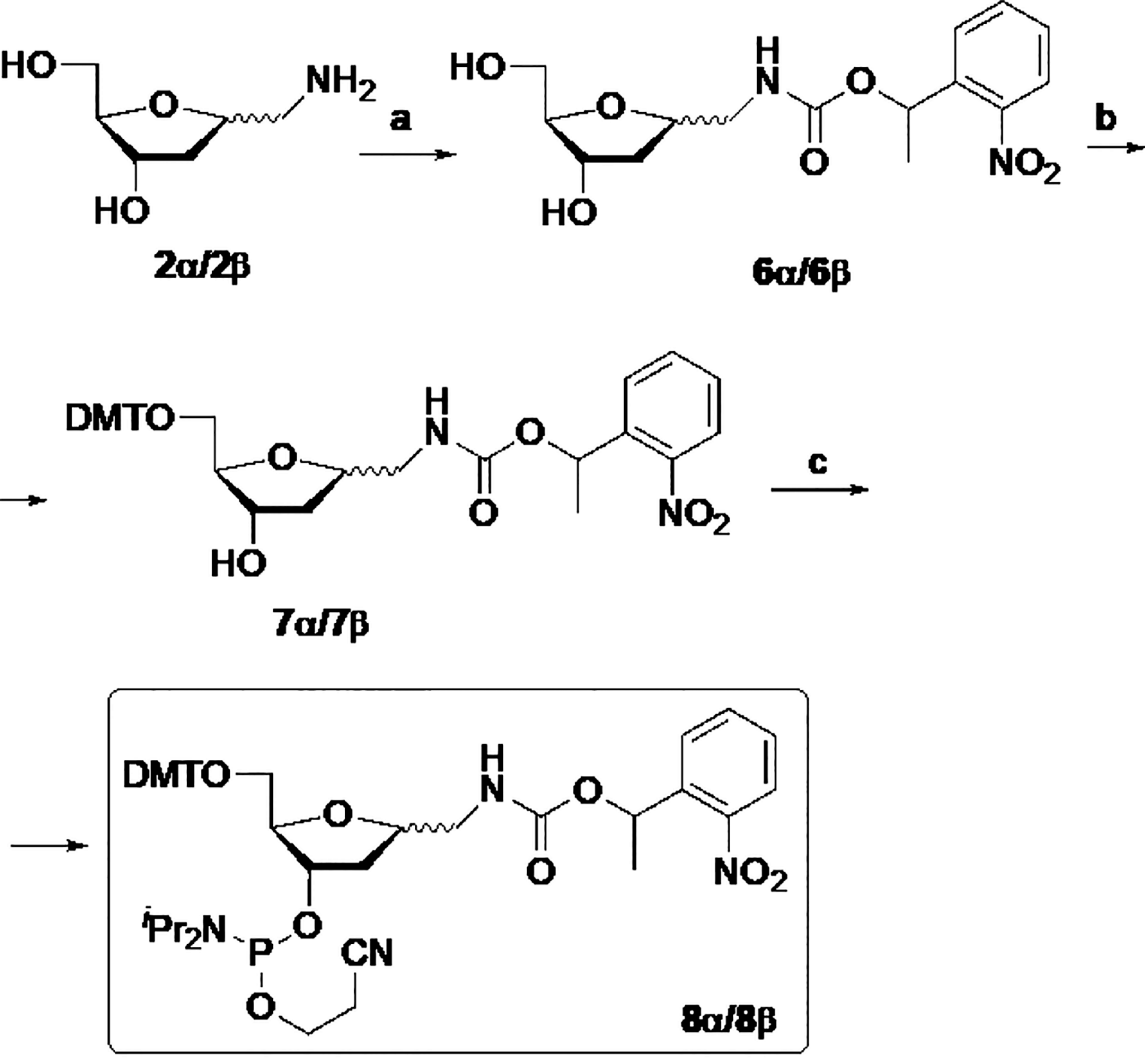
Synthesis of 1-Aminomethyl-1,2-dideoxy-d-*erythro*-pentofuranosyl-3-phosphoramidites Bearing a Photolabile Protecting Group at the Amino Function Reagents and conditions: (a) 1-(2-Nitrophenyl)ethyl *N*-succinimidyl carbonate, Et_3_N, MeOH, 30 °C, 1 h, 55% (**6α**) and 50% (**6β**); (b) DMTCl, Et_3_N, 1,4-dioxane, 35 °C, 2 h, 80% (**7α**) and 80% (**7β**); (c) 2-Cyanoethyl *N,N*-diisopropylchlorophosphoramidite, ^*i*^Pr_2_NEt, CH_2_Cl_2_, rt, 1 h, 78% (**8α**) and 72% (**8β**).

Preparation of phosphoramidites of 1α- and 1β-aminomethyl-1,2-dideoxy-d-*erythro*-pentofuranoses bearing a photolabile protecting group at the amino function is outlined in Scheme 2. The amino diol **2** was reacted with 1-(2-nitrophenyl)ethyl-*N*-succinimidyl carbonate^22^ to afford carbamates **6α** (55% yield) or **6β** (50% yield). As above, protection of the primary alcohol with DMT group yielded **7α**/**7β**, and subsequent phosphitylation gave derivatives **8α** or **8β** in 78% and 72% yield, respectively.

**Scheme 3:**
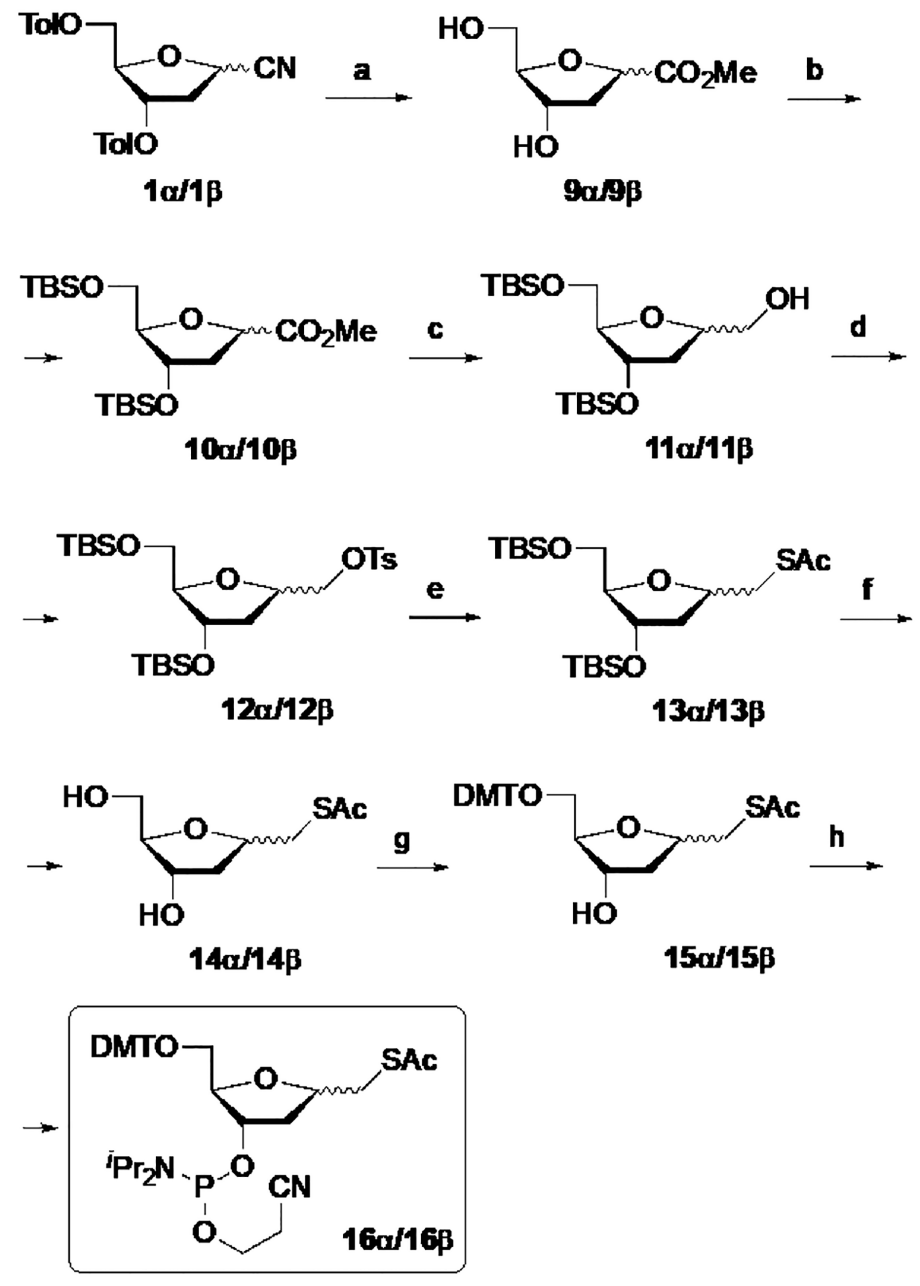
Synthesis of 1-*S*-Acetylmercaptomethyl-1,2-dideoxy-d-*erythro*-pentofuranose Phosphoramidites Reagents and conditions: (a) KOH, MeOH, H_2_O, 25 °C, 3 h, 85% (**9α**) and 75% (**9β**); (b) TBSCl, Imidazole, CH_2_Cl_2_, 50 °C, 5 h, 85% (**10α**) and 80% (**10β**); (c) LiAlH_4_, THF, −45 °C, 0.5 h, 90% (**11α**) and 1 h, 70% (**11β**); (d) TsCl, DMAP, Py, 0 °C → rt, 9 h, 90% (**12α**) and 80% (**12β**); (e) Potassium thioacetate, DMF, 65 °C, 6 h, 70% (**13α**) and 75% (**13β**); (f) (−)-CSA, MeOH, 0 °C → rt, 2 h, 80% (**14α**) and 80% (**14β**); (g) DMTCl, Et_3_N, 1,4-dioxane, 30 °C, 2 h, 80% (**15α**) and 85% (**15β**); (h) 2-Cyanoethyl *N,N*-diisopropylchlorophosphoramidite, ^*i*^Pr_2_NEt, CH_2_Cl_2_, rt, 1 h, 72% (**16α**) and 68% (**16β**).

The synthetic protocol for the 1-*S*-mercaptomethyl-1,2-dideoxy-d-*erythro*-pentofuranosyl-3-*O*-phosphoramidites **16α** or **16β** is summarized in Scheme 3. The nitriles **1α**/**1β** were treated with potassium hydroxide in MeOH/H_2_O. Under these conditions, hydrolysis of nitrile and in situ esterification in addition to the removal of the toluoyl protecting groups generated esters **9α** or **9β** in 85% and 75% yield, respectively. Then, alcohol groups were protected as *tert*-butyldimethylsilyl ether to give derivatives **10α**/**10β**. The reduction of esters **10** with lithium aluminum hydride in THF at −45 °C afforded alcohols **11α** (90% yield) or **11β** (70% yield), which were transformed into the tosylates **12α**/**12β** by treatment with *p*-toluensulfonyl chloride and catalytic DMAP in pyridine. The displacement of the tosylate group with potassium thioacetate in DMF afforded the thioesters **13α** or **13β** in 70% and 75% yields, respectively. Next, deprotection of the silyl groups with (−)-CSA in MeOH gave alcohols **14α**/**14β**. Each isomer was transformed in the phosphoramidites **16α** or **16β** after DMT protection of the primary hydroxyl giving place to **15α**/**15β** and phosphitylation of the secondary hydroxyl group.

### Synthesis of Solid Supports Functionalized with 1,2-Dideoxy-d-*erythro*-pentofuranose Monomers **4α**/**4β**, **7α**/**7β**, or **15α**/**15β**

**Scheme 4:**
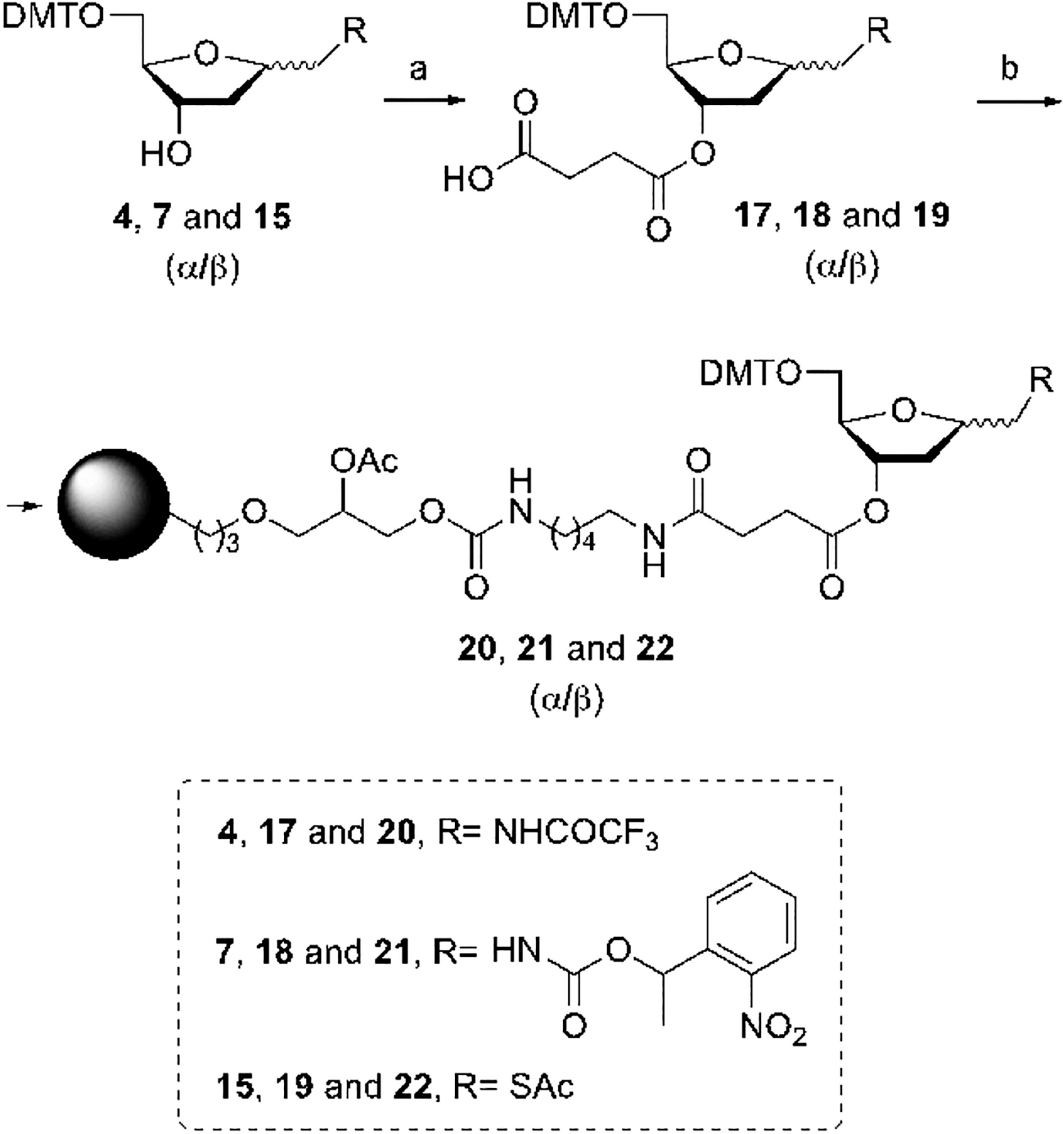
Preparation of CPG Solid Supports Functionalized with 1-Aminomethyl- or 1-Mercaptomethyl-1,2-dideoxy-d-*erythro*-pentofuranoses Reagents and conditions: (a) Succinic anhydride, DMAP, rt, overnight; (b) 2,2′-Dithio-bis(5-nitropyridine), Ph_3_P, LCAA-CPG, rt, 2 h (20-25 μmol/g).

In order to connect 1,2-dideoxy-d-*erythro*-pentofuranose monomers **4α/4β**, **7α/7β**, and **15α/15β** to the oligonucleotides on their 3′-end, we prepared the appropriate solid supports carrying these different derivatives. For this reason, the secondary alcohol at position 3 of the pentafuranose ring of each one of these derivatives was reacted with succinic anhydride yielding the corresponding succinate derivatives **17α/17β**, **18α/18β**, and **19α/19β** (Scheme 4). These compounds were used to functionalize the amino-controlled pore glass support (LCAA-CPG) to yield the CPG solid supports **20α/20β**, **21α/21β**, and **22α/22β**.

### Synthesis, Purification, and Characterization of Oligonucleotides Incorporating **4α**/**4β**, **7α**/**7β**, or **15α**/**15β** 1,2-Dideoxy-d-erythro-pentofuranose Monomers

The phosphoramidites **5α/5β**, **8α/8β**, and **16α/16β** and solid supports **20α/20β**, **21α/21β**, and **22α/22β** were used to prepare oligonucleotides containing these modified nucleotides either at the 3′-end or at the 5′-end of the sequence. All of the sequences shown in Table 1 were made on the automated DNA synthesizer using standard protocols.^23^ The short model sequence RS carrying the four natural bases was prepared to study their stability during all of the synthesis process and to obtain the optimal cleavage conditions. Next, we used all the derivatives to prepare the gapmer oligonucleotides, which contained the complementary sequence of the *Renilla* luciferase gene modified at their ends with 2′-*O*-methyl-RNA. Often, gapmer oligonucleotides are used for antisense gene expression inhibition experiments.

**Table 1:** Sequence of Oligonucleotides and Its Characterization by MALDI-TOF^a^

code	sequences (5′ → 3′)	MW (calcd)	MW (found)
RS**4α**	CATTGTCCA-**4α**	2880.5	2880.3
RS**7α**	CATTGTCCA-**7α**	2880.5/3072.5^b^	2880.5/3073.5^b^
RS**7β**	CATTGTCCA-**7β**	2880.5/3072.5^b^	2880.5/3073.5^b^
RS**15α**	CATTGTCCA-**15α**	2897.5/5792.1^c^	2897.2
Gapmer**4α**	cguuTCCTTTGTTCugga-**4α**	5865	5853.8
Gapmer**4β**	cguuTCCTTTGTTCugga-**4β**	5865	5854.5
Gapmer**7α**	cguuTCCTTTGTTCugga-**7α**	5866/6061^b^	5864.6
Gapmer**7β**	cguuTCCTTTGTTCugga-**7β**	5866/6061^b^	5855/6057
Gapmer**15α**	cguuTCCTTTGTTCugga-**15α**	5884	5880.8
Gapmer**15β**	cguuTCCTTTGTTCugga-**15β**	5883	5880.5
**4α**Gapmer	**4α**-cguuTCCTTTGTTCugga	5865	5864.5
**4β**Gapmer	**4β**-cguuTCCTTTGTTCugga	5865	5866.8
**7α**Gapmer	**7α**-cguuTCCTTTGTTCugga	5866	5865/6042
**7β**Gapmer	**7β**-cguuTCCTTTGTTCugga	5866	5849/6057
**15α**Gapmer	**15α**-cguuTCCTTTGTTCugga	5883	5878
**15β**Gapmer	**15β**-cguuTCCTTTGTTCugga	5883	5880.8
**7α**Gapmer**4α**	**7β**-cguuTCCTTTGTTCugga-**4α**	6075/6268	6074.0/6267.6

a Sequences of the synthesized oligonucleotide with the 2-deoxy-d-ribofuranose derivatives. T, G, C are 2′-deoxynucleotides. a, c, g, u are 2′-OMe-nucleotides.

b Expected MW with a photolabile protecting group.

c Expected MW of the dimer form with a disulfide bridge.

**Figure 1:**
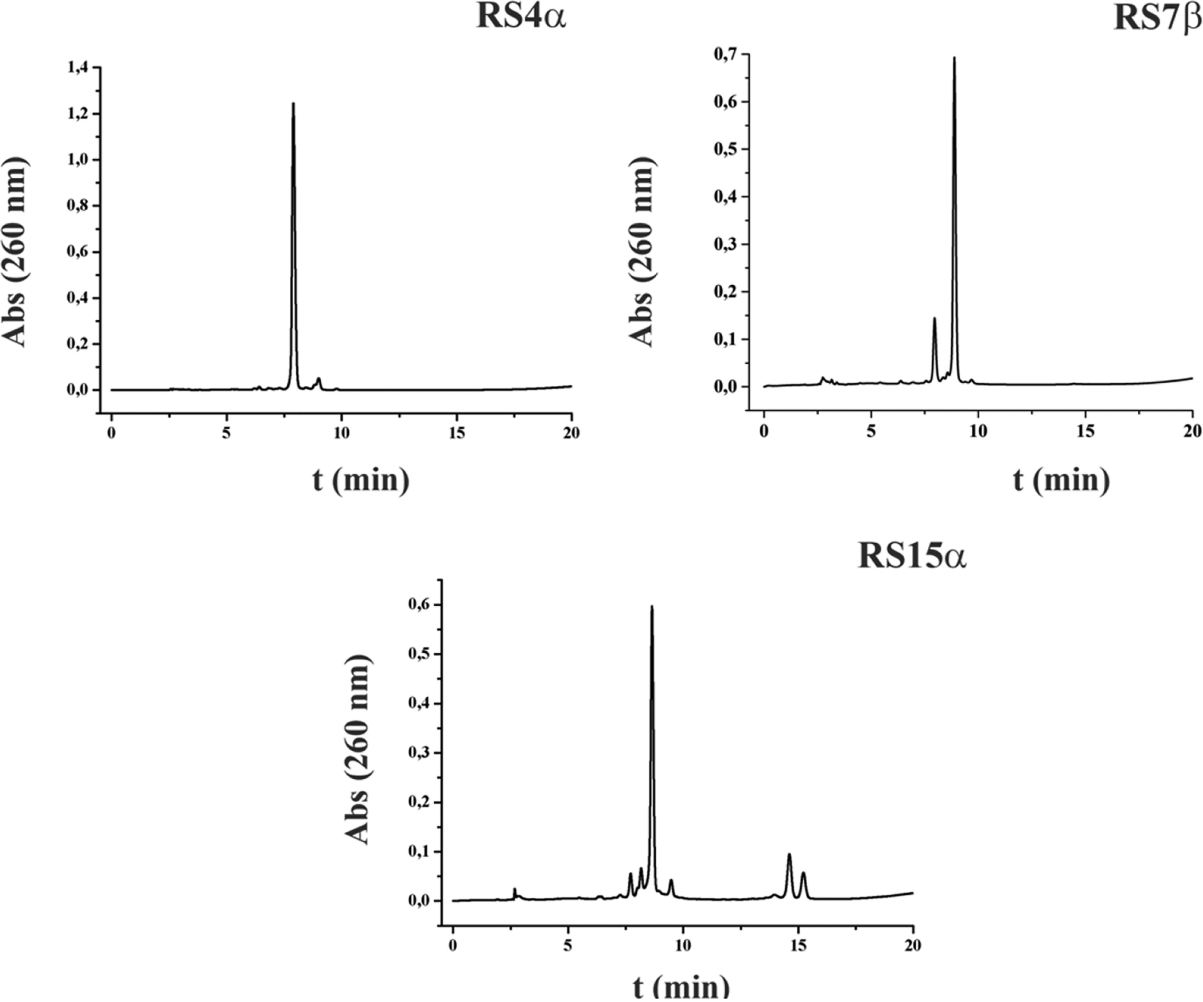
HPLC profiles of model oligonucleotides modified in the 3′-end with **4α**, **7β**, and **15α** 1,2-dideoxy-d-*erythro*-pentofuranose derivatives. ACE 3 μm HILA-3-1546-A column was used.

Next, the two RS oligonucleotides containing the aminomethyl 1,2-dideoxy-d-*erythro*-pentofuranoses (**4α** and **7β**) were treated with an ammonia solution overnight at 55 °C. The resulting crudes were analyzed by HPLC and characterized by MALDI-TOF. As expected, RS**4α** gave a unique peak with the correct mass which corresponds to the desired product deprotected. In the case of oligonucleotide RS**7β**, a side peak was present in the HPLC profile. Both products were collected and analyzed by mass spectrometry. The product with higher retention time corresponds to the desired product protected with the photolabile protecting group, and the minor product is the RS**7β** deprotected. This result indicated that the photolabile group is very sensitive to the light, and extra precautions, like working in the dark, need to be considered during deprotection in order to prevent its cleavage. The HPLC profiles are depicted in Figure 1, and the MW are shown in Table 1.

**Figure 2:**
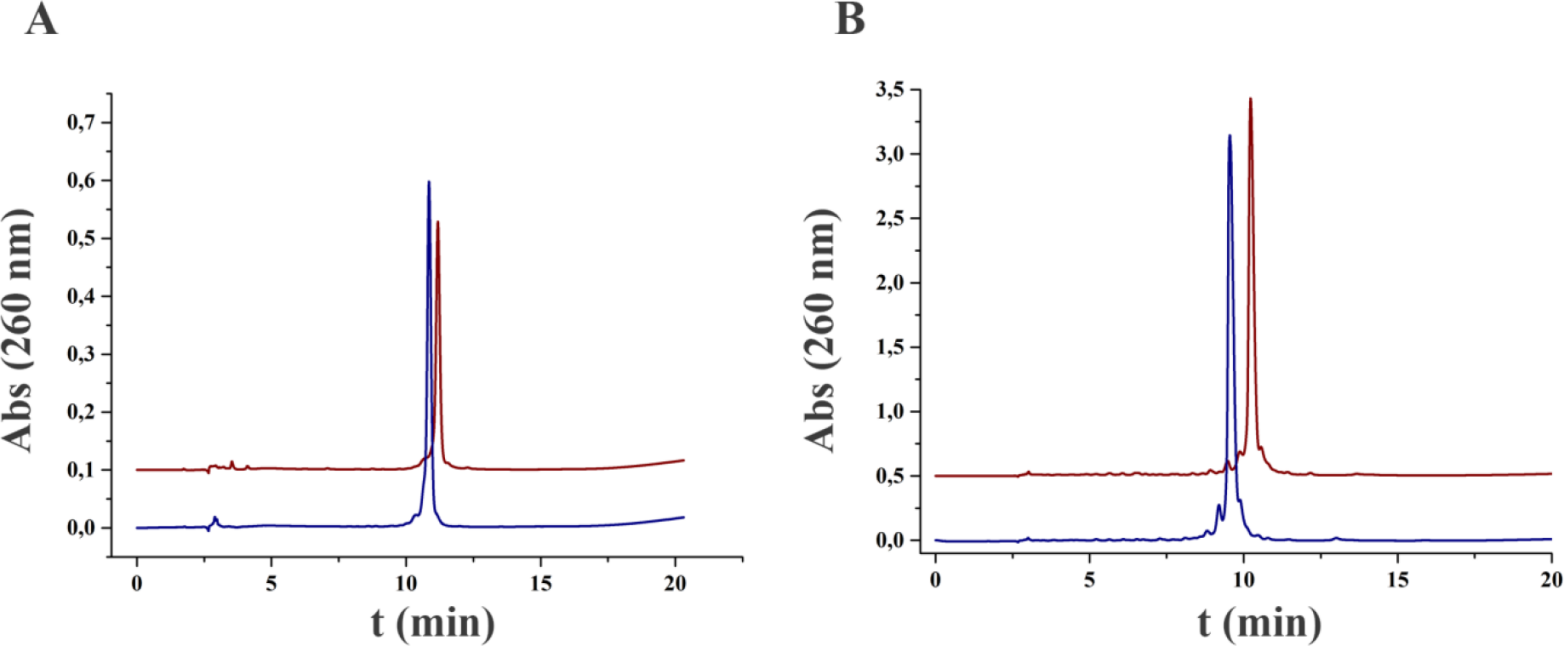
HPLC profiles of the Gapmer oligonucleotides modified with the amino group: (A) 5′-modified Gapmer and (B) 3′-modified Gapmer. In blue and red are drawn the **α** and **β** isomer forms, respectively. ACE 3 μm HILA-3-1546-A column was used.

In the case of the oligonucleotide RS**15α**, some modifications in the deprotection process were introduced to prevent side products. First, it was treated with a DBU solution followed by a wash with a 5% solution of Et_3_N. This treatment was necessary to remove the cyanoethyl protecting groups, as they can react with the free thiol function of the **15α** sugar giving the cyanoethylmercapto derivative as a byproduct. Next, it was treated with an ammonium solution containing 0.1 M DTT overnight at 55 °C to avoid dimerization. The HPLC analysis presented a unique peak, with the mass corresponding to the correct product. The optimal deprotection conditions found for each derivative were used for the deprotection of all the other gapmer sequences. The mass for the resulting products are shown in Table 1. All the gapmer sequences were obtained in a good yield which ranged 42–96%. The HPLC chromatograms of the 5′- and 3′-aminomethyl-modified gapmers are shown in Figure 2. The two isomeric forms (α- and β-) can be perfectly distinguishes by their different retention times in the HPLC profiles. These results confirmed the enantiomeric purity of these two novel α- and β-amino-linkers.

### Removal of the Photolabile Protecting Group in Modified Oligonucleotides with **7α** and **7β** Monomers

We studied the efficiency in the removal of the photolabile protecting group NPEC of the **7α** and **7β** oligonucleotide derivatives attached to the solid support and when they were already cleaved from the resin in order to compare both systems. In both cases, the modified gapmers were exposed to irradiation at 340 nm for different periods of time. As shown in Table 2, the NPEC protecting group needed a longer time to be removed when the **7α** and **7β** derivatives were attached to the solid support versus in solution. However, after 2 h of reaction the NPEC group was completely removed from the solid support, and no difference was observed between **7α** and **7β** derivatives. These results confirmed that the presence of the solid support does not interfere in the formation of the free amino oligonucleotide derivative product attached to it, allowing further coupling reactions in the solid phase.

**Table 2:** Data from the Kinetic Studies for the Removal of the NPEC of **7α** and **7β** Gapmers

photolysis	on the CPG support^a^		in solution phase^b^			
reaction time (min)	30	60	15	30	45	60
Gapmer**7α** (%)	78	91	60	89	100	100
Gapmer**7β** (%)	71	92	80	93	99	100

a Deprotection reaction on the CPG support was realized with 2 mg of resin.

b Deprotection reaction in solution was realized with 2 mg of oligonucleotide.

### Preparation of Oligonucleotide Conjugates

The incorporation of fluorescent and delivery elements to oligonucleotides is important for the development of new diagnostic and therapeutic tools. The introduction of functional groups with orthogonal deprotection procedures is essential in order to incorporate multiple elements in the same oligonucleotide. In this case, the presence of NPEC in **7α**- and **7β**-1,2-dideoxy-d-*erythro*-pentofuranose derivatives allowed conjugation reaction directly on the solid support.

**Scheme 5:**
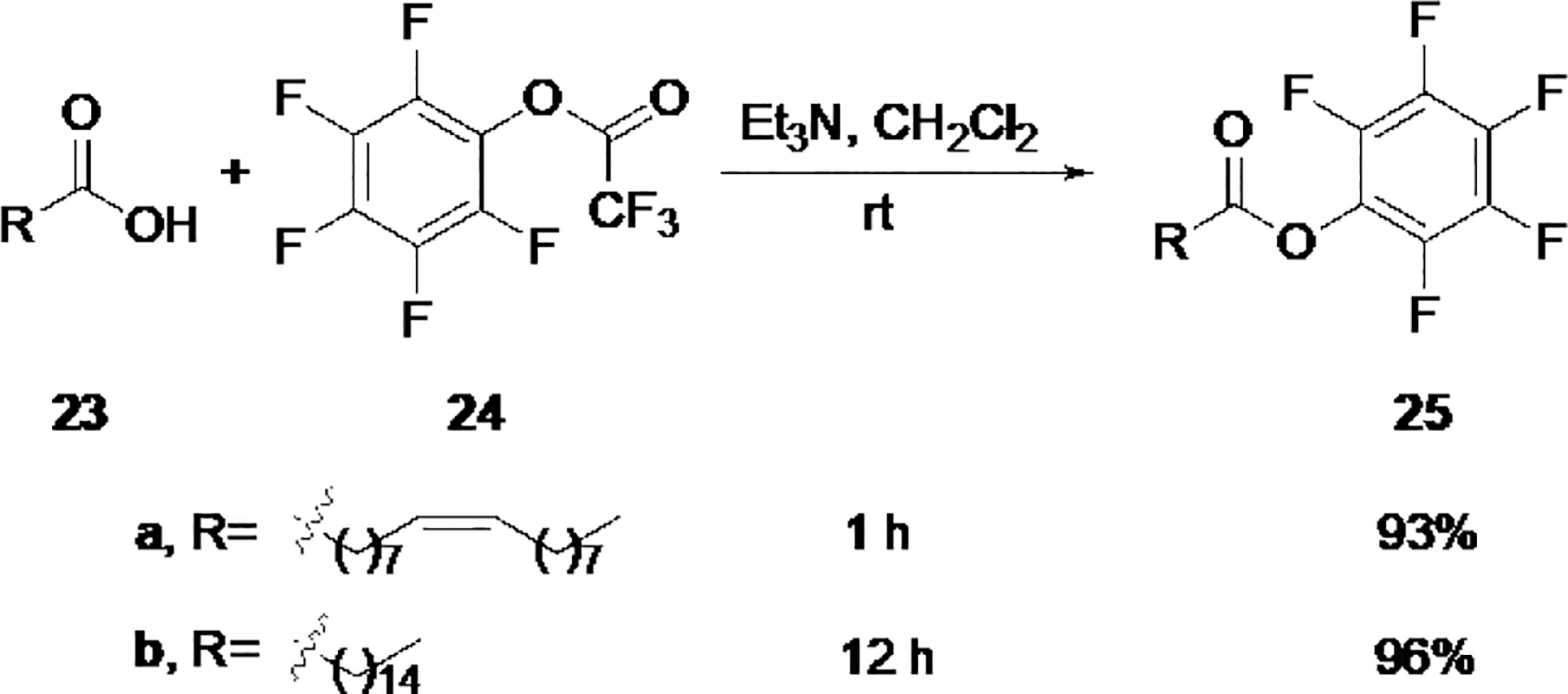
Synthesis of Pentafluorophenyl Esters of Fatty Acids

Prior to the incorporation of delivery elements to these modified oligonucleotides in the solid support, the gapmer**4α** and the RS**4α** oligonucleotides containing the amino derivative in the 3′-end was conjugated with fluorescein (FITC) and two different types of fatty acids (palmitic and oleic acids) in solution, respectively. The incorporation of the FITC and the two fatty acids was done by the reaction of the free amines of the modified nucleotide in the oligonucleotides with fluorescein isothiocyanate and the pentafluorophenyl ester of each one of the fatty acids. Before the conjugation reaction took place, the pentafluorophenyl esters of the oleate (**25a**) and palmitate (**25b**) were prepared as described in the literature^6,24−26^ with a 93% and 96% yield, respectively (Scheme 5).

Next, the gapmer **4α** oligonucleotide was treated with fluorescein isothiocyanate (FITC) and the RS**4α** was treated with the pentafluorophenyl oleate or palmitate in different buffer conditions to evaluate the influence of an organic cosolvent in the final yield of the oligonucleotide conjugate. In the case of the RS**4α**–fatty acids conjugation, DMF was added to the mixture of the carbonate buffer/acetonitrile solution to increase the relative amount of organic phase in the reaction. HPLC analysis revealed the presence of a product with a higher retention time then the free amino oligonucleotides in all the cases, and its mass corresponded with the desired conjugates (Table 3). However, conjugate gapmer**4α**–FITC was only obtained in a 50% yield with respect to the 76% and 64% yield of the RS**4α**–fatty acid conjugates. These results indicate that the solubility of the fatty acid in the reaction conditions was crucial to improve the final yield of the conjugates.

**Table 3:** Oligonucleotide Conjugates and Their Characterization by MALDI-TOF

oligonucleotide-conjugates	yield (%)	MW (calc)	MW (found)
Gapmer**4α**-FITC	50	6256.1	6258
RS**4α**-Oleic	76	3146	3145.6
RS**4α**-Palmitic	64	3121	3123.6
RS**7α**-Palmitic^a^	61	3121	3124.0
RS**7α**-Palmitic^b^	19	3121	3124.0
Palmitic-**7α**Gapmer^a^	72	6108	6107.5
Palmitic-**7α**Gapmer^b^	66	6108	6107.5
Palmitic-**7α**Gapmer^c^	46	6108	6107.5
FITC**-7α**Gapmer**4α**	6	6463	6465.2
FITC**-7α**Gapmer**4α-**Oleic	20	6728	6742.7

a Both photolysis and conjugation in solution.

b Photolysis over the solid support and conjugation in solution.

c Both photolysis and conjugation on the solid support.

Next, we evaluated the conjugation of the RS**7α** and **7α**gapmer over the solid support. These two **7α**-modified oligonucleotides were incubated with pentafluorophenyl palmitate in solution or on the solid support, in order to compare the reaction efficiency between both strategies. All the products were HPLC purified and characterized by mass spectrometry. The yields obtained in the conjugation of the fatty acid with amino-oligonucleotides are shown in Table 3. The result showed that RS**7α**-palmitic is only obtained with the desired yield (61%) when the reaction was done in solution. One of the reasons for the low yields could be due to the steric hindrance of the amino at the 3′-position with the solid support which reduces the conjugation efficiency. These results were confirmed as the palmitic-**7α**gapmer conjugate was obtained in the solid support when the **7α**-modified nucleotide was in its 5′-end. However, the conjugation reaction is less efficient (46%) than when the reaction is carried out in solution with a 66% yield. Despite this fact, solid phase is still a useful method for conjugation reactions due to its shorter reaction times and efficient removal of the excess of reagents, and because it allows orthogonal conjugation reactions with multiple elements.

**Scheme 6:**
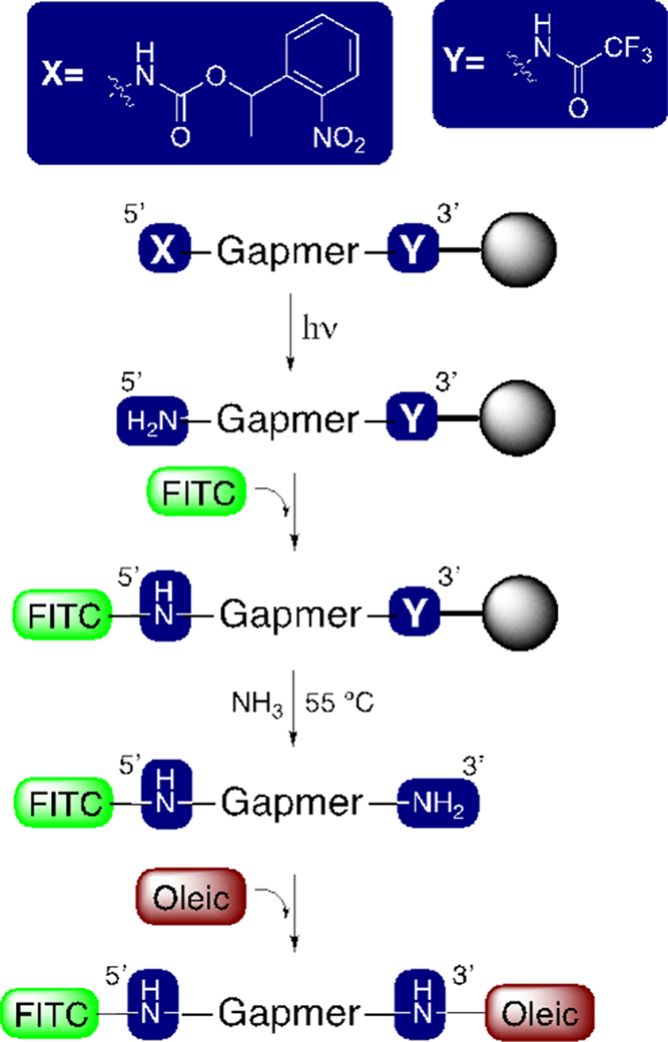
Synthesis of an Oligonucleotide Carrying Both a Fluorophore (FITC) and a Lipid (Oleic)

Finally, to investigate the possibility of preparing an oligonucleotide with two distinct ligands, we carried out the conjugation in an orthogonal manner of a lipid and a fluorescent compound at each end of the **7α**gapmer**4α** (Scheme 6). For this purpose, the **7α**gapmer**4α** modified at each end with the same nucleosidic derivative but with different protecting groups was used. First, irradiation of the gapmer bound to the solid support gave place selectively to the free amino group at 5′-end. Then, the resulting oligonucleotide was incubated with fluorescein (FITC) on the solid support, followed by the deprotection of 3′-trifluoroacetylamino group, which also liberated the oligo from the support. The FITC-**7α**gapmer**4α** 3′-amino was conjugated with the pentafluorophenyl oleate in solution. The final product was HPLC purified and characterized by mass spectrometry. The yield obtained is shown in Table 3. These results are a step forward to obtain multiple functionalized oligonucleotides for diagnostic and therapeutic applications.

## Discussion

During the past decade, we have witnessed large interest in oligonucleotide conjugates for gene analysis and therapeutic application. An important step in the production of these conjugates is the design, preparation, and functionalization of linking molecules for the connection of the ligand to the oligonucleotide. Here, we describe the synthesis of a novel series of connecting stereospecific linkers based on cyano sugar ribose precursors that can be obtained in the pure form in the two possible (α- and β-) isomeric forms. To this end, we described the synthesis of the appropriate reagents for oligonucleotide synthesis following solid-phase phosphoramidite chemistry. First, the synthesis of the aminomethyl sugar derivative from the ditoluoyl cyano-1,2-dideoxy-d-*erythro*-pentofuranose (**1α**/**1β**) is described. Conversion of the cyano group to the aminomethyl group is achieved in a single step that removed the toluoyl protecting group at the same time. The resulting aminomethyl sugar was protected with the base-labile trifluoroacetyl and the photolabile moieties. Second, the transformation of the cyano to the mercaptomethyl group required multiple synthesis steps. The synthesis protocol began with the conversion of the cyano group to the methyl carboxylate followed by reduction to hydroxymethyl group. Tosylation of the hydroxyl group followed by nucleophilic displacement with potassium thioacetate yielded the desired *S*-acetyl derivative. Then, both sugar derivatives were protected at the primary alcohol with the DMT group and were processed with the conventional methods to obtain the desired phosphoramidites and the corresponding functionalized CPG solid supports. The novel reagents are compatible with solid-phase synthesis protocols providing the desired amino or thiolated functionalized oligonucleotides (Table 1).

This demonstrated the usefulness of the novel amino linkers for the preparation of lipid– and fluorescent–oligonucleotide conjugates. The development of two different and orthogonal protecting groups for the aminomethyl-oligonucleotides allows the introduction of two different ligands in a single oligonucleotide.

**Figure 3:**
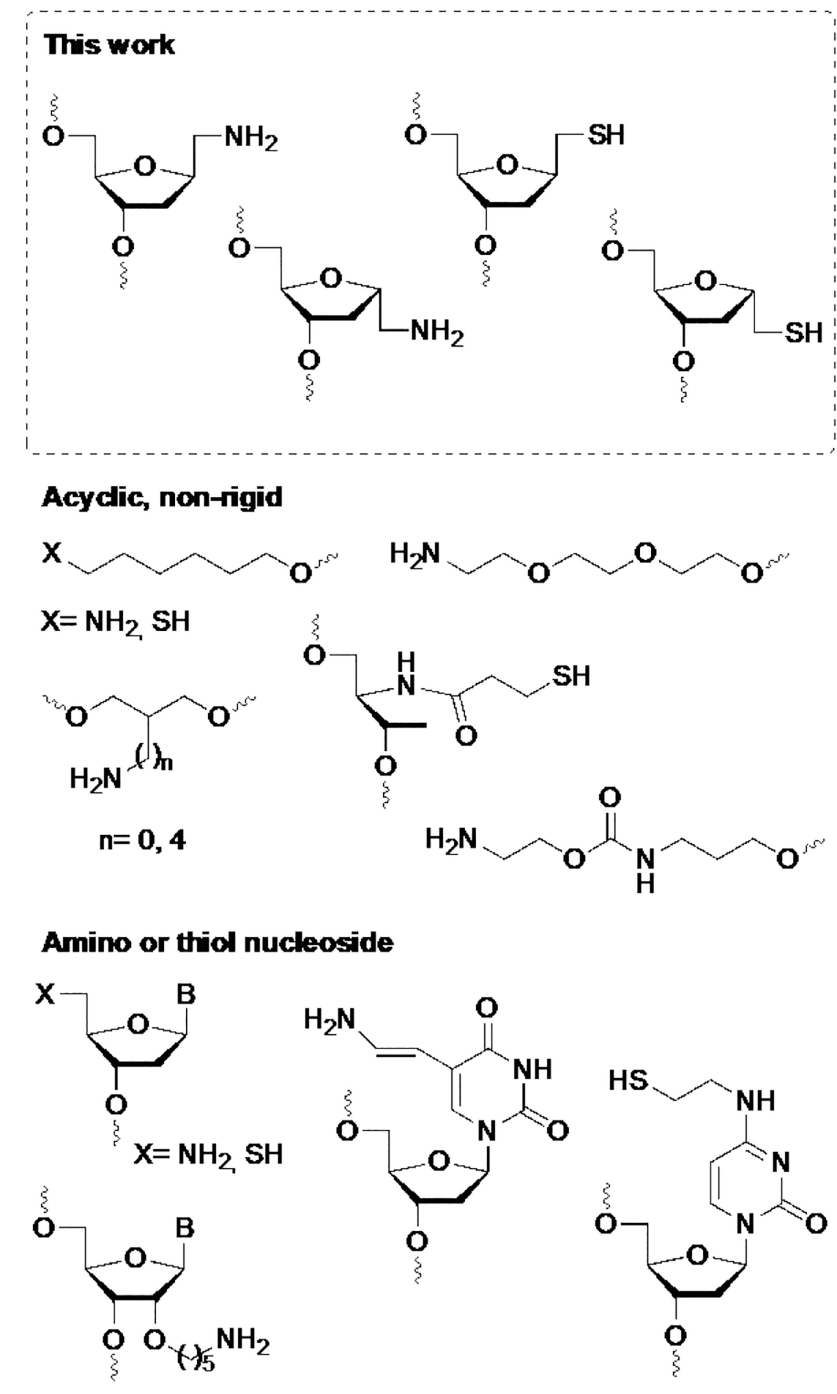
Amino and mercapto linkers for the functionalization of oligonucleotides.

The novel linkers developed in this work (Figure 3) are enantiomerically pure, semirigid, hydrophilic, and totally compatible with nucleic acid structural properties. Several amino and thiol linkers have been described in the literature.^5^ The simplest linkers are derived from aminoalcohols or mercaptoalcohols such as 6-aminohexanol^12^ or 6-mercaptohexanol.^16^ 5′-Amino^13^ and 5′-mercapto^27^ dideoxynucleosides have also been used for the introduction of reactive groups at the 5′-position of oligonucleotides. In amino linkers, the presence of an ether function at the β-position increases the nucleophilicity of the reactive amino group and allows more efficient conjugation reactions.^28−30^ However, all these linkers can only be introduced at the 5′-end of the oligonucleotides, whereas the novel linkers described in this work can be incorporated at any position in an oligonucleotide.

The incorporation of amino groups at the 3′-end utilized aminoalkyldiols. Most of them are acyclic and nonrigid, but some of them are not enantiomerically pure such as 2-amino-1,3-propanol^14^ and 2-butylamino-1,3-propanol^15^ and may produce diastereoisomeric mixtures. In addition, it has been described that the 2-amino-1,3-propanol linker may produce intramolecular side reactions.^31^ Threoninol derivatives have also been described for the preparation of thiolated oligonucleotides.^32^ Amino-^33^ and mercapto-^18^functionalized nucleosides at the nucleobases or at the 2′-position of a ribonucleotide^34^ have also been reported for the incorporation of amino and thiol reactive groups. The novel linkers described herein are enantiomerically pure and are free of side reactions. They do not require the use of expensive nucleosides but can be considered similar to nucleosides functionalized at the nucleobases or at the 2′-position of a ribonucleotide. Their smaller size similar to a nucleoside would be appropriate for the introduction of local probes such as fluorescent–quencher pairs.^35^ Furthermore, the cyano sugar ribose precursor could be transformed to other interesting reactive groups such as azide or alkyne groups for conjugation using cycloaddition reactions.^10^ The choice of using the 2-deoxyribose framework for the attachment of the reactive group allows easy incorporation into an oligonucleotide using standard solid-phase amidite chemistry.

## Conclusions

A key step in the synthesis of oligonucleotide conjugates is the preparation of the appropriate tethers that connect ligands with oligonucleotides. In this work, we provide an efficient solution to this problem that uses a common sugar precursor, cyano-2-deoxyribofuranose, for the generation of reactive aminomethyl and mercaptomethyl sugars. These intermediates have been converted to the appropriate solid supports and phosphoramidites in excellent yields for the preparation of oligonucleotides carrying amino or thiol groups at any predefined position. Oligonucleotides carrying the new tethers have been functionalized with lipids and fluoresceine demonstrating the usefulness of these enantiomerically pure, hydrophilic, and DNA-compatible linkers. Two orthogonal amino-protecting groups have been studied that can be removed under different conditions allowing the introduction of two ligands in a single oligonucleotide. The novel amidites described herein should ease the assembly of functional conjugates of oligonucleotides and pave the way for enhanced tissue targeting, cell internalization, and resistance to nucleases.

## Materials and Methods

### 1 General

#### 1.1 Reagents

Oleoyl chloride, oleic, and palmitic acids were purchased from Sigma. The standard 2′-deoxy and 2′-*O*-methyl-ribonucleoside phosphoramidites, reagents solutions, supports, and LCAA-CPG were purchased from Applied Biosystems (PEBiosystems Hispania S.A., Spain) and Link Technologies Ltd. (Lanarkshire, Scotland, UK). The rest of the chemicals were purchased from Aldrich, Sigma, or Fluka (Sigma-Aldrich S.A., Spain), and used without further purification. Anhydrous solvents and deuterated solvents (CDCl_3_ and MeOH-*d*_4_) were obtained from reputable sources and used as received. Thin-layer chromatography (TLC) was carried out on aluminum-backed Silica-Gel 60 F_254_ plates. The spots were visualized with UV light. Column chromatography was performed using Silica Gel (60 Å, 230 × 400 mesh). Matrix for MALDI-TOF experiments was composed of 2′,4′,6′-trihydroxyacetophenone monohydrate (THAP, Aldrich) and ammonium citrate dibasic (Fluka). Solvents for HPLC analysis were prepared using triethylammonium acetate (TEAA) and acetonitrile (Merck) as a mobile phase. The desalted columns with Sephadex G-25 (NAP-10 or NAP-5) were from GE Healthcare (Little Chalfont, UK). The rest of the chemicals were analytical reagent grade from commercial sources as specified. Ultrapure water (Millipore) was used in all experiments.

#### 1.2 Instrumentation

NMR spectra (^1^H, ^13^C, ^19^F, and ^31^P) were measured on Bruker DPX-300 (^1^H 300.13 MHz, ^13^C 75.5 MHz, and ^31^P 121.5 MHz) or Varian Mercury-400 (^1^H 400.13 MHz, ^13^C 100.6 MHz, ^19^F 376.5 MHz, and ^31^P 162.0 MHz). Chemical shifts for ^1^H, ^13^C, ^19^F, and ^31^P NMR are given in parts per million (ppm) from the residual solvent signal as the reference or tetramethylsilane (TMS) and coupling constants (*J*) values are given in Hertz (Hz). Modified oligonucleotides were synthesized on an Applied Biosystems 3400 DNA Synthesizer (Applied Biosystems). Semipreparative and analytical reverse-phase (RP) HPLC was performed on a Waters chromatography system with a 2695 Separations Module equipped with a Waters 2998 Photodiode Array Detector using different types of semipreparative columns: column A: Nucleosil 120 C_18_ (250 × 8 mm), column B: Xbridge OST C_18_ 2.5 μm (10 × 50 mm) and analytical columns: column C: XbridgeTM OST C_18_ 2.5 μm (4.6 × 50 mm) and column D: Column ACE 3 μm HILA-3-1546-A (4.6 × 150 mm). High resolution mass spectra (HRMS) were recorded on a mass spectrometer under electron spray ionization (ESI), and mass spectra of oligos were recorded on a MALDI Voyager DETM RP time-of-flight (TOF) spectrometer (Applied Biosystems). Molecular absorption spectra between 220 and 550 nm were recorded with a Jasco V650 spectrophotometer. The temperature was controlled with an 89090A Agilent Peltier device. Hellman quartz cuvettes were used.

### 2 Synthesis of 1-Functionalized 1,2-Dideoxy-d-erythro-pentofuranose Phosphoramidites

#### 2.1 Preparation of 1-Trifluoroacetylaminomethyl-1,2-dideoxy-d-erythro-pentofuranose Phosphoramidites **5α** and **5β**

##### 2.1.1 Synthesis of **2α**/**2β**

LiAlH_4_ (8 equiv) was added to a solution of **1α** or **1β** in anhydrous THF (0.15M). The reaction was stirred at reflux during 4 h. After cooling, excess of the reagent was decomposed by addition of THF and MeOH, and the mixture was filtered through Celite. The solvents were evaporated, and the crude product was subjected to column chromatography (gradient eluent MeOH–10% NH_3_/MeOH) to afford **2α** or **2β** (both contains traces of silica gel).

##### **1α**-Aminomethyl-1,2-dideoxy-d-*erythro*-pentofuranose (**2α**)

Yellowish oil. *R*_f_: 0.20 (1% NH_3_/MeOH); IR (NaCl): ν 3415, 2955, 1598 cm^–1^; ^1^H NMR (300.13 MHz, MeOH-*d*_4_): δ 1.76 (ddd, 1H, H_2_, *J* = 13.5, 4.8, 3.9 Hz), 2.41 (m, 1H, H_2_) 3.13 (m, 2H, H_6_), 3.52 (dd, 1H, H_5_, *J* = 11.7, 5.6 Hz), 3.59 (dd, 1H, H_5_, *J* = 11.7, 4.3 Hz), 3.97 (dt, 1H, H_4_, *J* = 5.5, 4.1 Hz), 4.26 (dt, 1H, H_3_, *J* = 6.6, 3.6 Hz), 4.40 (m, 1H, H_1_) ppm; ^13^C NMR (75.5 MHz, MeOH-*d*_4_): δ 38.6 (C_2_), 44.8 (C_6_), 63.4 (C_5_), 73.3 (C_3_), 76.1 (C_1_), 88.1 (C_4_) ppm; HRMS (ESI^+^, *m*/*z*): calcd for C_6_H_14_NO_3_ [M + H]^+^: 148.0968, found: 148.0977.

##### **1β**-Aminomethyl-1,2-dideoxy-d-*erythro*-pentofuranose (**2β**)

Yellowish oil. *R*_f_: 0.25 (1% NH_3_/MeOH); IR (NaCl): ν 3420, 2953, 1644 cm^–1^; ^1^H NMR (300.13 MHz, MeOH-*d*_4_): δ 1.88 (m, 2H, H_2_), 2.74 (dd, 1H, H_6_, *J* = 13.2, 6.9 Hz), 2.92 (dd, 1H, H_6_, *J* = 13.2, 3.5 Hz), 3.53 (dd, 1H, H_5_, *J* = 11.7, 5.0 Hz), 3.61 (dd, 1H, H_5_, *J* = 11.7, 4.1 Hz), 3.81 (q, 1H, H_4_, *J* = 4.4 Hz), 4.10 (m, 2H, H_1_ + H_3_) ppm; ^13^C NMR (75.5 MHz, MeOH-*d*_4_): δ 38.9 (C_2_), 45.9 (C_6_), 63.8 (C_5_), 73.9 (C_3_), 79.1 (C_1_), 88.8 (C_4_) ppm; HRMS (ESI^+^, *m*/*z*): calcd for C_6_H_14_NO_3_ [M + H]^+^: 148.0968, found: 148.0974.

##### 2.1.2 Synthesis of **3α**/**3β**

To a solution of **2α** or **2β** in anhydrous DMF (0.1 M) was added anhydrous Et_3_N (5.5 equiv) and ethyl trifluoroacetate (3.3 equiv). The mixture was stirred at 80 °C during 24 h, and then evaporated to leave a residue, which was purified by column chromatography (gradient eluent 5–20% 2-propanol/CH_2_Cl_2_) affording **3α** (70% yield) or **3β** (80% yield). Isolated yields are for two steps.

##### 1,2-Dideoxy-**1α**-[*N*-(trifluoroacetyl)aminomethyl]-d-*erythro*-pentofuranose (**3α**)

Clear oil. *R*_f_: 0.58 (20% MeOH/CH_2_Cl_2_); IR (NaCl): ν 3404, 3302, 2940, 1713, 1216, 1191, 1160 cm^–1^; ^1^H NMR (300.13 MHz, MeOH-*d*_4_): δ 1.69 (ddd, 1H, H_2_, *J* = 13.2, 5.8, 4.6 Hz), 2.34 (ddd, 1H, H_2_, *J* = 13.1, 7.7, 6.6 Hz), 3.45 (d, 2H, H_6_, *J* = 5.4 Hz), 3.52 (dd, 1H, H_5_, *J* = 11.7, 4.9 Hz), 3.57 (dd, 1H, H_5_, *J* = 11.7, 4.2 Hz), 3.87 (dt, 1H, H_4_, *J* = 5.3, 4.0 Hz), 4.25 (overlapped signal, 2H, H_1_ + H_3_) ppm; ^13^C NMR (75.5 MHz, MeOH-*d*_4_): δ 38.7 (C_2_), 45.3 (C_6_), 63.3 (C_5_), 73.2 (C_3_), 77.5 (C_1_), 87.6 (C_4_), 117.6 (q, *C*F_3_, *J* = 286.7 Hz), 159.2 (q, *C*=O, *J* = 36.8 Hz) ppm; HRMS (ESI^+^, *m*/*z*): calcd for C_8_H_13_F_3_NO_4_ [M + H]^+^: 244.0791, found: 244.0786, calcd for C_8_H_12_F_3_NNaO_4_ [M + Na]^+^: 266.0611, found: 266.0603, calcd for C_8_H_12_F_3_KNO_4_ [M+K]^+^: 282.0350, found: 282.0342.

##### 1,2-Dideoxy-**1β**-[*N*-(trifluoroacetyl)aminomethyl]-d-*erythro*-pentofuranose (**3β**)

Clear oil. *R*_f_: 0.57 (20% MeOH/CH_2_Cl_2_); IR (NaCl): ν 3395, 3315, 2945, 1712, 1192, 1162 cm^–1^; ^1^H NMR (300.13 MHz, MeOH-*d*_4_): δ 1.87 (m, 2H, H_2_), 3.44 (t, 2H, H_6_, *J* = 5.0 Hz), 3.52 (dd, 1H, H_5_, *J* = 11.7, 4.9 Hz), 3.60 (dd, 1H, H_5_, *J* = 11.7, 4.2 Hz), 3.80 (dt, 1H, H_4_, *J* = 4.5, 3.1 Hz), 4.22 (dt, 1H, H_3_, *J* = 5.9, 2.9 Hz), 4.29 (m, 1H, H_1_) ppm; ^13^C NMR (75.5 MHz, MeOH-*d*_4_): δ 38.9 (C_2_), 44.6 (C_6_), 63.6 (C_5_), 73.7 (C_3_), 77.8 (C_1_), 88.7 (C_4_), 117.6 (q, *C*F_3_, *J* = 286.6 Hz), 159.3 (q, *C*=O, *J* = 37.0 Hz) ppm; HRMS (ESI^+^, *m*/*z*): calcd for C_8_H_13_F_3_NO_4_ [M + H]^+^: 244.0791, found: 244.0780, calcd for C_8_H_12_F_3_NNaO_4_ [M + Na]^+^: 266.0611, found: 266.0599, calcd for C_8_H_12_F_3_KNO_4_ [M+K]^+^: 282.0350, found: 282.0337.

##### 2.1.3 Synthesis of **4α**/**4β**

Anhydrous Et_3_N (10 equiv) and 4,4′-dimethoxytrityl chloride (1.5 equiv) were successively added to a solution of **3α** or **3β** in anhydrous 1,4-dioxane (0.1 M). The mixture was stirred at 30 °C during 2 h. Then, saturated aqueous NaHCO_3_ was added and the solution was extracted with CH_2_Cl_2_. The organic layer was dried, filtered, and evaporated to dryness. The crude residue was purified by column chromatography (40% EtOAc/Hexane). The column was previously packed with silica gel using a 10% Et_3_N solution in EtOAc:Hexane (4:6, v-v). Isolated yields of **4α** or **4β** were 65% and 70%, respectively.

##### 1,2-Dideoxy-5-*O*-(4,4′-dimethoxytrityl)-**1α**-[*N*-(trifluoroacetyl)aminomethyl]-d-*erythro*-pentofuranose (**4α**)

Intense yellow oil. *R*_f_: 0.22 (40% EtOAc/Hexane); IR (NaCl): ν 3414, 3282, 2934, 1715, 1509, 1252, 1202, 1177 cm^–1^; ^1^H NMR (300.13 MHz, MeOH-*d*_4_): δ 1.68 (ddd, 1H, H_2_, *J* = 13.2, 5.8, 4.2 Hz), 2.35 (ddd, 1H, H_2_, *J* = 13.1, 7.7, 6.4 Hz), 3.07 (dd, 1H, H_5_, *J* = 9.9, 5.1 Hz), 3.14 (dd, 1H, H_5_, *J* = 10.0, 4.3 Hz), 3.47 (m, 2H, H_6_),3.77 (s, 6H, *Me*-DMT), 4.03 (q, 1H, H_4_, *J* = 4.1 Hz), 4.26 (dt, 1H, H_3_, *J* = 6.5, 3.8 Hz), 4.34 (m, 1H, H_1_), 6.85 (d, 4H, H_g_, *J* = 8.9 Hz), 7.24 (m, 3H, H_c_ + H_d_), 7.31 (d, 4H, H_f_, *J* = 8.9 Hz), 7.43 (d, 2H, H_b_, *J* = 7.1 Hz) ppm; ^13^C NMR (75.5 MHz, MeOH-*d*_4_): δ 38.7 (C_2_), 45.4 (C_6_), 55.7 (2 O-*C*H_3_), 65.5 (C_5_), 74.0 (C_3_), 77.8 (C_1_), 86.9 (C_4_), 87.5 (C_10_), 114.1 (4C_g_), 117.6 (q, *C*F_3_, *J* = 286.7 Hz), 127.7 (C_d_), 128.7 (2C_c_), 129.3 (2C_b_), 131.3 (4C_f_), 137.2 (C_e_), 137.3 (C_e_), 146.4 (C_a_), 159.2 (q, *C*=O, *J* = 37.0 Hz), 160.1 (2C_h_) ppm; HRMS (ESI^+^, *m*/*z*): calcd for C_29_H_30_F_3_NNaO_6_ [M + Na]^+^: 568.1917, found: 568.1893, calcd for C_29_H_30_F_3_KNO_6_ [M+K]^+^: 584.1657, found: 584.1632.

##### 1,2-Dideoxy-5-*O*-(4,4′-dimethoxytrityl)-**1β**-[*N*-(trifluoroacetyl)aminomethyl]-d-*erythro*-pentofuranose (**4β**)

Yellowish oil. *R*_f_: 0.13 (40% EtOAc/Hexane); IR (NaCl): ν 3424, 3331, 2934, 1721, 1510, 1251, 1216, 1177 cm^–1^; ^1^H NMR (300.13 MHz, MeOH-*d*_4_): δ 1.75 (ddd, 1H, H_2_, *J* = 13.1, 10.0, 5.8 Hz), 1.90 (ddd, 1H, H_2_, *J* = 13.0, 5.5, 1.9 Hz), 3.10 (m, 2H, H_5_), 3.39 (dd, 1H, H_6_, *J* = 13.7, 6.3 Hz), 3.49 (dd, 1H, H_6_, *J* = 13.7, 4.6 Hz), 3.76 (s, 6H, *Me*-DMT), 3.94 (dt, 1H, H_4_, *J* = 5.0, 2.3 Hz), 4.23 (m, 1H, H_3_), 4.29 (m, 1H, H_1_), 6.84 (d, 4H, H_g_, *J* = 8.9 Hz), 7.21 (m, 3H, H_c_ + H_d_), 7.31 (d, 4H, H_f_, *J* = 8.9 Hz), 7.44 (m, 2H, H_b_) ppm; ^13^C NMR (75.5 MHz, MeOH-*d*_4_): δ39.2 (C_2_), 44.6 (C_6_), 55.7 (2 O-*C*H_3_), 65.6 (C_5_), 74.4 (C_3_), 77.9 (C_1_), 87.4 (C_10_), 87.7 (C_4_), 114.1 (4C_g_), 117.5 (q, *C*F_3_, *J* = 286.8 Hz), 127.8 (C_d_), 128.7 (2C_c_), 129.3 (2C_b_), 131.3 (4C_f_), 137.2 (C_e_), 137.3 (C_e_), 146.4 (C_a_), 159.2 (q, *C*=O, *J* = 36.8 Hz), 160.1 (2C_h_) ppm; HRMS (ESI^+^, *m*/*z*): calcd for C_29_H_30_F_3_NNaO_6_ [M + Na]^+^: 568.1917, found: 568.1884, calcd for C_29_H_30_F_3_KNO_6_ [M+K]^+^: 584.1657, found: 584.1623.

##### 2.1.4 Synthesis of **5α**/**5β**

Compound **4α** or **4β** was coevaporated twice with anhydrous MeCN under reduced pressure and left in a freeze-dryer overnight. Next, the product was dissolved in anhydrous CH_2_Cl_2_ (0.1 M) and anhydrous ^*i*^Pr_2_NEt (3 equiv) was added. The resulting solution was cooled in an ice bath and 2-cyanoethyl *N,N*-diisopropylchlorophosphoramidite (1.5 equiv) was added dropwise with a syringe. After 15 min, the reaction was allowed to reach rt and stirred for an additional 1 h. Then, the reaction was quenched with brine and extracted with CH_2_Cl_2_. The organic layer was dried, filtered, and evaporated to dryness. The crude residue was purified by column chromatography (40% EtOAc/Hexane) to afford **5α** (84% yield) or **5β** (70% yield). The column was previously packed with silica gel using a 10% Et_3_N solution in EtOAc:Hexane (4:6, v–v).

##### 1,2-Dideoxy-5-*O*-(4,4′-dimethoxytrityl)-**1α**-[*N*-(trifluoroacetyl)aminomethyl]-d-*erythro*-pentofuranosyl-3-*O*-(2-cyanoethyl-*N,N*-diisopropyl)phosphoramidite (**5α-A**)

Clear oil. *R*_f_: 0.63 (40% EtOAc/Hexane); IR (NaCl): ν 3318, 2966, 2254, 1723, 1509, 1251, 1213, 1179, 1033 cm^–1^; ^1^H NMR (300.13 MHz, MeOH-*d*_4_): δ 1.15 (d, 6H, H_v_, *J* = 6.9 Hz), 1.18 (d, 6H, H_v_, *J* = 6.9 Hz), 1.79 (dt, 1H, H_2_, *J* = 13.1, 5.0 Hz), 2.40 (dt, 1H, H_2_, *J* = 13.6, 7.0 Hz), 2.52 (t, 2H, H_*y*_, *J* = 6.0 Hz), 3.10 (dd, 1H, H_5_, *J* = 10.1, 4.6 Hz), 3.25 (dd, 1H, H_5_, *J* = 10.1, 4.0 Hz), 3.43 (dd, 1H, H_6_, *J* = 13.7, 4.1 Hz), 3.63 (overlapped signal, 5H, H_6_ + H_w_ + H_*x*_), 3.78 (s, 6H, *Me*-DMT), 4.15 (q, 1H, H_4_, *J* = 4.0 Hz), 4.34 (m, 1H, H_1_), 4.48 (m, 1H, H_3_), 6.85 (d, 4H, H_g_, *J* = 8.9 Hz), 7.24 (m, 3H, H_c_ + H_d_), 7.30 (d, 4H, H_f_, *J* = 8.9 Hz), 7.45 (d, 2H, H_b_, *J* = 7.0 Hz) ppm; ^13^C NMR (75.5 MHz, MeOH-*d*_4_): δ 20.9 (d, C_*y*_, *J* = 7.3 Hz), 25.0 (d, 4C_v_, *J* = 7.4 Hz), 38.2 (d, C_2_, *J* = 4.0 Hz), 44.4 (d, 2C_w_, *J* = 12.3 Hz), 45.2 (C_6_), 55.7 (2 O-*C*H_3_), 59.7 (d, C_*x*_, *J* = 18.6 Hz), 65.1 (C_5_), 76.1 (d, C_3_, *J* = 16.2 Hz), 78.4 (C_1_), 86.0 (d, C_4_, *J* = 4.4 Hz), 87.5 (C_10_), 114.1 (4C_g_), 117.5 (q, *C*F_3_, *J* = 286.8 Hz), 119.3 (*C*N), 127.8 (C_d_), 128.8 (2C_c_), 129.3 (2C_b_), 131.3 (4C_f_), 137.2 (C_e_), 137.3 (C_e_), 146.4 (C_a_), 159.2 (q, *C*=O, *J* = 37.6 Hz), 160.1 (2C_h_) ppm; ^31^P NMR (121.5 MHz, MeOH-*d*_4_): δ148.1 ppm; HRMS (ESI^+^, *m*/*z*): calcd for C_38_H_47_F_3_KNaO_7_P [M+K]^+^: 784.2735, found: 784.2702.

##### 1,2-Dideoxy-5-*O*-(4,4′-dimethoxytrityl)-**1α**-[*N*-(trifluoroacetyl)aminomethyl]-d-*erythro*-pentofuranosyl-3-*O*-(2-cyanoethyl-*N,N*-diisopropyl)phosphoramidite (**5α-B**)

Clear oil. *R*_f_: 0.53 (40% EtOAc/Hexane); IR (NaCl): ν 3424, 3324, 2967, 2254, 1725, 1509, 1251, 1215, 1178, 1034 cm^–1^; ^1^H NMR (300.13 MHz, MeOH-*d*_4_): δ 1.07 (d, 6H, H_v_, *J* = 6.8 Hz), 1.17 (d, 6H, H_v_, *J* = 6.8 Hz), 1.89 (dt, 1H, H_2_, *J* = 13.2, 4.9 Hz), 2.40 (dt, 1H, H_2_, *J* = 13.7, 7.0 Hz), 2.68 (t, 2H, H_*y*_, *J* = 5.8 Hz), 3.08 (dd, 1H, H_5_, *J* = 10.0, 4.8 Hz), 3.19 (dd, 1H, H_5_, *J* = 10.0, 4.5 Hz), 3.54 (overlapped signal, 4H, H_6_ + H_w_), 3.77 (s, 6H, *Me*-DMT), 3.78 (m, 2H, H_*x*_), 4.12 (q, 1H, H_4_, *J* = 4.0 Hz), 4.33 (m, 1H, H_1_), 4.46 (m, 1H, H_3_), 6.85 (d, 4H, H_g_, *J* = 8.9 Hz), 7.23 (m, 3H, H_c_ + H_d_), 7.30 (d, 4H, H_f_, *J* = 8.9 Hz), 7.43 (d, 2H, H_b_, *J* = 7.0 Hz) ppm; ^13^C NMR (75.5 MHz, MeOH-*d*_4_): δ 20.9 (d, C_*y*_, *J* = 6.8 Hz), 23.6 (d, 2C_v_, *J* = 7.1 Hz), 23.6 (d, 2C_v_, *J* = 7.1 Hz), 38.3 (d, C_2_, *J* = 2.8 Hz), 44.4 (d, 2C_w_, *J* = 12.4 Hz), 45.3 (C_6_), 55.7 (2 O-*C*H_3_), 59.7 (d, C_*x*_, *J* = 19.0 Hz), 65.2 (C_5_), 76.6 (d, C_3_, *J* = 16.8 Hz), 78.4 (C_1_), 85.9 (d, C_4_, *J* = 5.9 Hz), 87.5 (C_10_), 114.1 (4C_g_), 117.5 (q, *C*F_3_, *J* = 286.8 Hz), 119.5 (*C*N), 127.8 (C_d_), 128.8 (2C_c_), 129.3 (2C_b_), 131.3 (4C_f_), 137.1 (C_e_), 137.2 (C_e_), 146.4 (C_a_), 159.2 (q, *C*=O, *J* = 37.6 Hz), 160.1 (2C_h_) ppm; ^31^P NMR (121.5 MHz, MeOH-*d*_4_): δ148.1 ppm; HRMS (ESI^+^, *m*/*z*): calcd for C_38_H_48_F_3_N_3_O_7_P [M + H]^+^: 746.3176, found: 746.3151, calcd for C_38_H_47_F_3_N_3_NaO_7_P [M + Na]^+^: 768.2996, found: 768.2965, calcd for C_38_H_47_F_3_KN_3_O_7_P [M+K]^+^: 784.2735, found: 784.2709.

##### 1,2-Dideoxy-5-*O*-(4,4′-dimethoxytrityl)-**1β**-[*N*-(trifluoroacetyl)aminomethyl]-d-*erythro*-pentofuranosyl-3-*O*-(2-cyanoethyl-*N,N*-diisopropyl)phosphoramidite (**5β-A**)

Clear oil. *R*_f_: 0.34 (40% EtOAc/Hexane); IR (NaCl): ν 3425, 3324, 2968, 2254, 1726, 1509, 1251, 1215, 1178, 1034 cm^–1^; ^1^H NMR (300.13 MHz, MeOH-*d*_4_): δ 1.16 (d, 6H, H_v_, *J* = 6.8 Hz), 1.19 (d, 6H, H_v_, *J* = 6.8 Hz), 1.83 (m, 1H, H_2_), 2.01 (m, 1H, H_2_), 2.54 (t, 2H, H_*y*_, *J* = 6.0 Hz), 3.15 (m, 2H, H_5_), 3.47 (m, 2H, H_6_), 3.65 (overlapped signal, 4H, H_*x*_ + H_w_), 3.78 (s, 6H, *Me*-DMT), 4.06 (m, 1H, H_4_), 4.27 (m, 1H, H_1_), 4.43 (m, 1H, H_3_), 6.86 (d, 4H, H_g_, *J* = 8.9 Hz), 7.22 (m, 3H, H_c_ + H_d_), 7.32 (d, 4H, H_f_, *J* = 7.5 Hz), 7.45 (d, 2H, H_b_, *J* = 7.3 Hz) ppm; ^13^C NMR (75.5 MHz, MeOH-*d*_4_): δ 20.9 (d, C_*y*_, *J* = 6.8 Hz), 25.0 (d, 2C_v_, *J* = 7.5 Hz), 25.0 (d, 2C_v_, *J* = 7.5 Hz), 38.5 (d, C_2_, *J* = 4.6 Hz), 44.4 (d, 2C_w_, *J* = 12.0 Hz), 44.5 (C_6_), 55.7 (2 O-*C*H_3_), 59.7 (d, C_*x*_, *J* = 18.6 Hz), 65.1 (C_5_), 76.5 (d, C_3_, *J* = 16.4 Hz), 78.2 (C_1_), 87.1 (d, C_4_, *J* = 4.0 Hz), 87.5 (C_10_), 114.1 (4C_g_), 117.5 (q, *C*F_3_, *J* = 286.8 Hz), 119.3 (*C*N), 127.8 (C_d_), 128.7 (2C_c_), 129.3 (2C_b_), 131.3 (4C_f_), 137.2 (C_e_), 137.3 (C_e_), 146.4 (C_a_), 159.2 (q, *C*=O, *J* = 36.8 Hz), 160.1 (2C_h_) ppm; ^31^P NMR (121.5 MHz, MeOH-*d*_4_): δ148.1 ppm; HRMS (ESI^+^, *m*/*z*): calcd for C_38_H_47_F_3_N_3_NaO_7_P [M + Na]^+^: 768.2996, found: 768.2968.

##### 1,2-Dideoxy-5-*O*-(4,4′-dimethoxytrityl)-**1β**-[*N*-(trifluoroacetyl)aminomethyl]-d-*erythro*-pentofuranosyl-3-*O*-(2-cyanoethyl-*N,N*-diisopropyl)phosphoramidite (**5β-B**)

Clear oil. *R*_f_: 0.28 (30% EtOAc/Hexane); IR (NaCl): ν 3424, 3322, 2967, 2254, 1726, 1510, 1251, 1215, 1179, 1034 cm^–1^; ^1^H NMR (300.13 MHz, MeOH-*d*_4_): δ 1.09 (d, 6H, H_v_, *J* = 6.8 Hz), 1.18 (d, 6H, H_v_, *J* = 6.8 Hz), 1.80 (m, 1H, H_2_), 2.12 (dd, 1H, H_2_, *J* = 12.9, 4.7 Hz), 2.68 (t, 2H, H_*y*_, *J* = 6.0 Hz), 3.12 (d, 2H, H_5_, *J* = 4.9 Hz), 3.46 (m, 2H, H_6_), 3.59 (m, 2H, H_w_), 3.77 (m, 8H, *Me*-DMT + H_*x*_), 4.02 (m, 1H, H_4_), 4.28 (dq, 1H, H_1_, *J* = 10.0, 5.5 Hz), 4.42 (m, 1H, H_3_), 6.85 (d, 4H, H_g_, *J* = 8.9 Hz), 7.23 (m, 3H, H_c_ + H_d_), 7.31 (d, 4H, H_f_, *J* = 8.9 Hz), 7.43 (d, 2H, H_b_, *J* = 7.2 Hz) ppm; ^13^C NMR (75.5 MHz, MeOH-*d*_4_): δ 20.9 (d, C_*y*_, *J* = 7.1 Hz), 25.0 (d, 2C_v_, *J* = 7.0 Hz), 25.0 (d, 2C_v_, *J* = 7.0 Hz), 38.5 (d, C_2_, *J* = 3.6 Hz), 44.4 (d, 2C_w_, *J* = 12.7 Hz), 44.5 (C_6_), 55.7 (2 O-*C*H_3_), 59.8 (d, C_*x*_, *J* = 18.9 Hz), 65.2 (C_5_), 76.8 (d, C_3_, *J* = 17.0 Hz), 78.1 (C_1_), 86.9 (d, C_4_, *J* = 5.6 Hz), 87.5 (C_10_), 114.1 (4C_g_), 117.5 (q, *C*F_3_, *J* = 286.8 Hz), 119.5 (*C*N), 127.8 (C_d_), 128.8 (2C_c_), 129.3 (2C_b_), 131.3 (4C_f_), 137.1 (C_e_), 137.2 (C_e_), 146.4 (C_a_), 159.2 (q, *C*=O, *J* = 37.6 Hz), 160.1 (2C_h_) ppm; ^31^P NMR (121.5 MHz, MeOH-*d*_4_): δ147.7 ppm; HRMS (ESI^+^, *m*/*z*): calcd for C_38_H_48_F_3_N_3_O_7_P [M + H]^+^: 746.3176, found: 746.3156, calcd for C_38_H_47_F_3_N_3_NaO_7_P [M + Na]^+^: 768.2996, found: 768.2972.

#### 2.2 Preparation of 1-NPEC-aminomethyl-1,2-dideoxy-d-erythro-pentofuranose Phosphoramidites **8α** and **8β**

##### 2.2.1 Synthesis of **6α**/**6β**

To a solution of **2α** or **2β** in anhydrous MeOH (0.1M) was added anhydrous Et_3_N (1.5 equiv) and 1-(2-nitrophenyl)ethyl-*N*-succinimidyl carbonate^22^ (1 equiv). The mixture was stirred at 30 °C during 1 h, and then evaporated to leave a residue, which was poured into saturated aqueous NaCl and extracted with EtOAc. The organic layer was dried, filtered, and evaporated to dryness. The crude residue was purified by column chromatography (5% MeOH/CH_2_Cl_2_) to afford **6α** (55% yield from **1**) or **6β** (50% yield from **1**).

##### 1,2-Dideoxy-**1α**-[(1-(2-nitrophenyl)ethoxy)carbonylaminomethyl]-d-*erythro*-pentofuranose (**6α**)

Yellow oil. *R*_f_: 0.29 (10% MeOH/CH_2_Cl_2_); IR (NaCl): ν 3360, 2939, 1694, 1538, 1259 cm^–1^; ^1^H NMR (300.13 MHz, MeOH-*d*_4_): δ 1.60 (d, 3H, H_11_, *J* = 6.8 Hz), 1.64 (m, 1H, H_2_), 2.24 (m, 1H, H_2_), 3.19 (m, 2H, H_6_), 3.50 (dd, 1H, H_5_, *J* = 11.7, 5.6 Hz), 3.59 (dd, 1H, H_5_, *J* = 11.7, 3.9 Hz), 3.79 (m, 1H, H_4_), 4.09 (m, 1H, H_1_), 4.19 (m, 1H, H_3_), 6.14 (q, 1H, H_10_, *J* = 6.8 Hz), 7.48 (m, 1H, H_arom_), 7.72 (m, 2H, H_arom_), 7.94 (d, 1H, H_arom_, *J* = 7.7 Hz) ppm; ^13^C NMR (75.5 MHz, MeOH-*d*_4_): δ 22.5 (C_11_), 38.6 (C_2_), 46.1 (C_6_), 46.1 (C_6_), 63.3 (C_5_), 69.6 (C_10_), 73.2 (C_3_), 78.2 (C_1_), 78.3 (C_1_), 87.0 (C_4_), 125.2 (CH_arom_), 128.3 (CH_arom_), 129.5 (CH_arom_), 134.8 (CH_arom_), 134.9 (CH_arom_), 139.9 (C_12_), 149.1 (C_13_), 157.9 (*C*=O), 158.0 (*C*=O) ppm; HRMS (ESI^+^, *m*/*z*): calcd for C_15_H_21_N_2_O_7_ [M + H]^+^: 341.1343, found: 341.1332, calcd for C_15_H_20_N_2_NaO_7_ [M + Na]^+^: 363.1163, found: 363.1153, calcd for C_15_H_20_KN_2_O_7_ [M+K]^+^: 379.0902, found: 379.0891.

##### 1,2-Dideoxy-**1β**-[(1-(2-nitrophenyl)ethoxy)carbonylaminomethyl]-d-*erythro*-pentofuranose (**6β**)

Light brown oil. *R*_f_: 0.26 (10% MeOH/CH_2_Cl_2_); IR (NaCl): ν 3355, 2937, 1703, 1525, 1261 cm^–1^; ^1^H NMR (300.13 MHz, MeOH-*d*_4_): δ 1.61 (d, 3H, H_11_, *J* = 6.6 Hz), 1.63–1.84 (several m, 2H, H_2_), 3.19 (m, 2H, H_6_), 3.53 (m, 2H, H_5_), 3.75 (m, 1H, H_4_), 4.16 (m, 2H, H_1_ + H_3_), 6.13 (q, 1H, H_10_, *J* = 6.5 Hz), 7.50 (m, 1H, H_arom_), 7.72 (m, 2H, H_arom_), 7.95 (d, 1H, H_arom_, *J* = 7.6 Hz) ppm; ^13^C NMR (75.5 MHz, MeOH-*d*_4_): δ 22.3 (C_11_), 22.4 (C_11_), 38.4 (C_2_), 38.6 (C_2_), 45.2 (C_6_), 45.5 (C_6_), 63.6 (C_5_), 63.7 (C_5_), 69.6 (C_10_), 73.6 (C_3_), 78.7 (C_1_), 78.8 (C_1_), 88.5 (C_4_),88.6 (C_4_), 125.3 (CH_arom_), 128.2 (CH_arom_), 129.6 (CH_arom_), 134.8 (CH_arom_), 139.8 (C_12_), 149.2 (C_13_), 158.2 (*C*=O) ppm; HRMS (ESI^+^, *m*/*z*): calcd for C_15_H_21_N_2_O_7_ [M + H]^+^: 341.1343, found: 341.1339, calcd for C_15_H_20_N_2_NaO_7_ [M + Na]^+^: 363.1163, found: 363.1157, calcd for C_15_H_20_KN_2_O_7_ [M+K]^+^: 379.0902, found: 379.0896.

##### 2.2.2 Synthesis of **7α**/**7β**

A procedure similar to that described for the synthesis of **4α/4β**, starting from **6α/6β** and with a reaction temperature of 35 °C, gave **7α** (80% yield) or **7β** (80% yield).

##### 1,2-Dideoxy-5-*O*-(4,4′-dimethoxytrityl)-**1α**-[(1-(2-nitrophenyl)ethoxy)carbonylamino-methyl]-d-*erythro*-pentofuranose (**7α**)

Yellowish oil. *R*_f_: 0.19 (50% EtOAc/Hexane); IR (NaCl): ν 3422, 2932, 1719, 1525, 1508, 1252 cm^–1^; ^1^H NMR (300.13 MHz, MeOH-*d*_4_): δ 1.58 (d, 3H, H_11_, *J* = 6.5 Hz), 1.59 (m, 1H, H_2_), 2.23 (m, 1H, H_2_), 3.10 (m, 2H, H_5_), 3.23 (m, 2H, H_6_), 3.74 and 3.75 (2s, 6H, *Me*-DMT), 6.15 (q, 1H, H_4_, *J* = 6.4 Hz), 4.14 (m, 1H, H_1_), 4.22 (m, 1H, H_3_), 6.15 (q, 1H, H_10_, *J* = 6.4 Hz), 6.82 (m, 4H, H_arom_), 7.14–7.36 (several m, 8H, H_arom_), 7.44 (m, 2H, H_arom_), 7.67 (m, 2H, H_arom_), 7.91 (m, 1H, H_arom_) ppm; ^13^C NMR (75.5 MHz, MeOH-*d*_4_): δ 22.5 (C_11_), 38.7 (C_2_), 38.8 (C_2_), 46.3 (C_6_), 55.7 (2 O-*C*H_3_), 65.4 (C_5_), 65.5 (C_5_), 69.6 (C_10_), 74.1 (C_3_), 74.2 (C_3_), 78.7 (C_1_), 78.9 (C_1_), 86.4 (C_4_), 87.4 (C_18_), 114.0 (4C_g_), 125.2 (CH_arom_), 127.7 (C_d_), 128.1 (CH_arom_), 128.2 (CH_arom_), 128.7 (2C_c_), 129.4 (2C_b_), 129.4 (CH_arom_), 131.3 (4C_f_), 134.9 (CH_arom_), 137.3 (2C_e_), 139.9 (C_12_), 146.5 (C_a_), 149.0 (C_13_), 158.0 (*C*=O), 160.0 (2C_h_) ppm; HRMS (ESI^+^, *m*/*z*): calcd for C_36_H_38_N_2_NaO_9_ [M + Na]^+^: 665.2470, found: 665.2462, calcd for C_36_H_38_KN_2_O_9_ [M+K]^+^: 681.2209, found: 681.2201.

##### 1,2-Dideoxy-5-*O*-(4,4′-dimethoxytrityl)-**1β**-[(1-(2-nitrophenyl)ethoxy)carbonylamino-methyl]-d-*erythro*-pentofuranose (**7β**)

Yellowish oil. *R*_f_: 0.16 (50% EtOAc/Hexane); IR (NaCl): ν 3424, 2931, 1722, 1525, 1510, 1252 cm^–1^; ^1^H NMR (300.13 MHz, MeOH-*d*_4_): δ 1.52 and 1.53 (2d, 3H, H_11_, *J* = 6.4 Hz), 1.65–1.85 (several m, 2H, H_2_), 3.12 (m, 2H, H_5_), 3.23 (m, 2H, H_6_), 3.77 and 3.78 (2s, 6H, *Me*-DMT), 3.91 (m, 1H, H_4_), 4.19 (m, 2H, H_1_ + H_3_), 6.13 (m, 1H, H_10_), 6.86 (m, 4H, H_arom_), 7.17–7.65 (several m, 12H, H_arom_), 7.93 (m, 1H, H_arom_) ppm; ^13^C NMR (75.5 MHz, MeOH-*d*_4_): δ 22.4 (C_11_), 22.5 (C_11_), 38.6 (C_2_), 38.9 (C_2_), 45.3 (C_6_), 45.5 (C_6_), 55.7 (2 O-*C*H_3_), 65.6 (C_5_), 65.71 (C_5_), 69.6 (C_10_), 74.4 (C_3_), 74.5 (C_3_), 78.6 (C_1_), 78.7 (C_1_), 87.4 (C_18_), 87.6 (C_4_), 114.1 (4C_g_), 125.2 (CH_arom_), 127.8 (C_d_), 128.2 (CH_arom_), 128.8 (2C_c_), 129.4 (2C_b_), 129.5 (CH_arom_), 131.3 (4C_f_), 134.8 (CH_arom_), 137.3 (C_e_), 137.4 (C_e_), 139.8 (C_12_), 146.5 (C_a_), 149.0 (C_13_), 157.9 (*C*=O), 160.1 (2C_h_) ppm; HRMS (ESI^+^, *m*/*z*): calcd for C_36_H_38_N_2_NaO_9_ [M + Na]^+^: 665.2470, found: 665.2454, calcd for C_36_H_38_KN_2_O_9_ [M+K]^+^: 681.2209, found: 681.2192.

##### 2.2.3 Synthesis of **8α**/**8β**

A procedure analogous to that described for the synthesis of **5α/5β**, starting from **7α/7β**, gave **8α** (78% yield) or **8β** (72% yield).

##### 1,2-Dideoxy-5-*O*-(4,4′-dimethoxytrityl)-**1α**-[(1-(2-nitrophenyl)ethoxy)carbonylamino-methyl]-d-*erythro*-pentofuranosyl-3-*O*-(2-cyanoethyl-*N,N*-diisopropyl)phosphoramidite (**8α-A**)

Clear oil. *R*_f_: 0.34 (40% EtOAc/Hexane); IR (NaCl): ν 3355, 2967, 2253, 1723, 1526, 1510, 1251, 1179, 1033 cm^–1^; ^1^H NMR (300.13 MHz, MeOH-*d*_4_): δ 1.10–1.18 (several d, 12H, H_v_, *J* = 6.8 Hz), 1.60 (d, 3H, H_11_, *J* = 6.5 Hz), 1.72 (m, 1H, H_2_), 2.29 (m, 1H, H_2_), 2.50 (t, 2H, H_*y*_, *J* = 6.0 Hz), 3.08 (m, 1H, H_5_), 3.24 (m, 3H, H_5_ + H_6_), 3.60 (m, 4H, H_*x*_ + H_w_), 3.77 and 3.78 (2s, 6H, *Me*-DMT), 4.09 (m, 1H, H_4_), 4.19 (m, 1H, H_1_), 4.44 (m, 1H, H_3_), 6.15 (m, 1H, H_10_), 6.85 (m, 4H, H_arom_), 7.17–7.49 (several m, 10H, H_arom_), 7.70 (m, 2H, H_arom_), 7.93 (m, 1H, H_arom_) ppm; ^13^C NMR (75.5 MHz, MeOH-*d*_4_): δ 20.8 (d, C_*y*_, *J* = 6.6 Hz), 22.5 (C_11_), 25.0 (d, 4C_v_, *J* = 7.3 Hz), 38.0 (d, C_2_, *J* = 4.0 Hz), 38.1 (d, C_2_, *J* = 4.0 Hz), 44.3 (d, C_w_, *J* = 12.2 Hz), 46.0 (C_6_), 46.2 (C_6_), 55.7 (2 O-*C*H_3_), 59.7 (d, C_*x*_, *J* = 18.3 Hz), 65.1 (C_5_), 69.6 (C_10_), 75.9 (d, C_3_, *J* = 15.6 Hz), 76.0 (d, C_3_, *J* = 16.4 Hz), 79.0 (C_1_), 79.3 (C_1_), 85.7 (C_4_), 87.5 (C_18_), 114.1 (4C_g_), 119.3 (*C*N), 125.2 (CH_arom_), 127.8 (C_d_), 128.2 (CH_arom_), 128.3 (CH_arom_), 128.8 (2C_c_), 129.4 (2C_b_), 129.5 (CH_arom_), 131.4 (4C_f_), 134.9 (CH_arom_), 137.2 (C_e_), 137.3 (C_e_), 137.4 (C_e_), 140.0 (C_12_), 146.5 (C_a_), 149.1 (C_13_), 158.0 (*C*=O), 160.1 (2C_h_) ppm; ^31^P NMR (121.5 MHz, MeOH-*d*_4_): δ 148.0 ppm; HRMS (ESI^+^, *m*/*z*): calcd for C_45_H_56_N_4_O_10_P [M + H]^+^: 843.3729, found: 843.3726, calcd for C_45_H_55_N_4_NaO_10_P [M + Na]^+^: 865.3548, found: 865.3545, calcd for C_45_H_55_KN_4_O_10_P [M+K]^+^: 881.3287, found: 881.3300.

##### 1,2-Dideoxy-5-*O*-(4,4′-dimethoxytrityl)-**1α**-[(1-(2-nitrophenyl)ethoxy)carbonylamino-methyl]-d-*erythro*-pentofuranosyl-3-*O*-(2-cyanoethyl-*N,N*-diisopropyl)phosphoramidite (**8α-B**)

Clear oil. *R*_f_: 0.30 (40% EtOAc/Hexane); IR (NaCl): ν 3360, 2967, 2253, 1723, 1526, 1510, 1252, 1179, 1033 cm^–1^; ^1^H NMR (300.13 MHz, MeOH-*d*_4_): δ 1.04 (d, 6H, H_v_, *J* = 6.8 Hz), 1.15 (d, 6H, H_v_, *J* = 6.8 Hz), 1.60 (d, 3H, H_11_, *J* = 6.6 Hz), 1.82 (m, 1H, H_2_), 2.31 (m, 1H, H_2_), 2.65 (t, 2H, H_*y*_, *J* = 5.9 Hz), 3.06 (m, 1H, H_5_), 3.16 (m, 1H, H_5_), 3.24 (m, 2H, H_6_), 3.55 (m, 2H, H_w_), 3.72 (m, 2H, H_*x*_), 3.77 (s, 6H, *Me*-DMT), 4.07 (m, 1H, H_4_), 4.17 (m, 1H, H_1_), 4.42 (m, 1H, H_3_), 6.14 (m, 1H, H_10_), 6.83 (m, 4H, H_arom_), 7.16–7.49 (m, 10H, H_arom_), 7.70 (m, 2H, H_arom_), 7.93 (m, 1H, H_arom_) ppm; ^13^C NMR (75.5 MHz, MeOH-*d*_4_): δ 20.9 (d, C_*y*_, *J* = 6.7 Hz), 22.5 (C_11_), 22.6 (C_11_), 24.9 (d, 2C_v_, *J* = 7.7 Hz), 25.0 (d, 2C_v_, *J* = 7.3 Hz), 38.2 (C_2_), 44.3 (d, C_w_, *J* = 12.6 Hz), 46.2 (C_6_), 54.8 (2 O-*C*H_3_), 59.7 (d, C_*x*_, *J* = 19.0 Hz), 65.2 (C_5_), 65.3 (C_5_), 69.6 (C_10_), 76.5 (d, C_3_, *J* = 16.2 Hz), 76.6 (d, C_3_, *J* = 17.7 Hz), 79.1 (C_1_), 79.3 (C_1_), 85.7 (d, C_4_, *J* = 5.7 Hz), 87.5 (C_18_), 114.1 (4C_g_), 119.5 (*C*N), 125.2 (CH_arom_), 127.8 (C_d_), 128.2 (CH_arom_), 128.3 (CH_arom_), 128.8 (2C_c_), 129.3 (2C_b_), 129.5 (CH_arom_), 131.3 (4C_f_), 134.8 (CH_arom_), 137.2 (2C_e_), 139.9 (C_12_), 146.4 (C_a_), 149.1 (C_13_), 157.9 (*C*=O), 160.1 (2C_h_) ppm; ^31^P NMR (121.5 MHz, MeOH-*d*_4_): δ 148.0 and 148.1 ppm; HRMS (ESI^+^, *m*/*z*): calcd for C_45_H_56_N_4_O_10_P [M + H]^+^: 843.3729, found: 843.3725, calcd for C_45_H_55_N_4_NaO_10_P [M + Na]^+^: 865.3548, found: 865.3550, calcd for C_45_H_55_KN_4_O_10_P [M+K]^+^: 881.3287, found: 881.3310.

##### 1,2-Dideoxy-5-*O*-(4,4′-dimethoxytrityl)-**1β**-[(1-(2-nitrophenyl)ethoxy)carbonylamino-methyl]-d-*erythro*-pentofuranosyl-3-*O*-(2-cyanoethyl-*N,N*-diisopropyl)phosphoramidite (**8β-A**)

Clear oil. *R*_f_: 0.41 (40% EtOAc/Hexane); IR (NaCl): ν 3356, 2932, 2253, 1725, 1526, 1510, 1251, 1179, 1033 cm^–1^; ^1^H NMR (300.13 MHz, MeOH-*d*_4_): δ 1.13 (d, 6H, H_v_, *J* = 6.8 Hz), 1.18 (d, 6H, H_v_, *J* = 6.8 Hz), 1.49 and 1.52 (2d, 3H, H_11_, 6.5 Hz), 1.66–1.96 (several m, 2H, H_2_), 2.52 (t, 2H, H_*y*_, *J* = 6.0 Hz), 3.07–3.29 (several m, 4H, H_5_ + H_6_), 3.48–3.71 (m, 4H, H_*x*_ + H_w_), 3.77 and 3.78 (2s, 6H, *Me*-DMT), 4.03 (m, 1H, H_4_), 4.16 (m, 1H, H_1_), 4.40 (m, 1H, H_3_), 6.11 (m, 1H, H_10_), 6.85 (m, 4H, H_arom_), 7.11–7.64 (several m, 12H, H_arom_), 7.91 (m, 1H, H_arom_) ppm; ^13^C NMR (75.5 MHz, MeOH-*d*_4_): δ 20.9 (d, C_*y*_, *J* = 6.8 Hz), 22.4 (C_11_), 22.5 (C_11_), 24.9 (d, 2C_v_, *J* = 7.2 Hz), 24.9 (d, 2C_v_, *J* = 7.3 Hz), 37.8 (C_2_), 38.2 (C_2_), 44.4 (d, C_w_, *J* = 11.9 Hz), 45.0 (C_6_), 55.7 (2 O-*C*H_3_), 59.7 (d, C_*x*_, *J* = 19.2 Hz), 65.2 (C_5_), 69.6 (C_10_), 77.6 (C_3_, cross-peak in HSQC), 78.9 (C_1_), 79.0 (C_1_), 87.0 (C_4_), 87.5 (C_18_), 114.1 (4C_g_), 119.3 (*C*N), 125.2 (CH_arom_), 127.8 (C_d_), 128.2 (CH_arom_), 128.8 (2C_c_), 129.4 (2C_b_), 129.5 (CH_arom_), 131.4 (4C_f_), 134.8 (CH_arom_), 137.2 (C_e_), 137.3 (C_e_), 139.6 (C_12_), 146.4 (C_a_), 149.1 (C_13_), 158.0 (*C*=O), 160.1 (2C_h_) ppm; ^31^P NMR (121.5 MHz, MeOH-*d*_4_): δ 148.0 ppm; HRMS (ESI^+^, *m*/*z*): calcd for C_45_H_56_N_4_O_10_P [M + H]^+^: 843.3729, found: 843.3723, calcd for C_45_H_55_N_4_NaO_10_P [M + Na]^+^: 865.3548, found: 865.3541.

##### 1,2-Dideoxy-5-*O*-(4,4′-dimethoxytrityl)-**1β**-[(1-(2-nitrophenyl)ethoxy)carbonylamino-methyl]-d-*erythro*-pentofuranosyl-3-*O*-(2-cyanoethyl-*N,N*-diisopropyl)phosphoramidite (**8β-B**)

Clear oil. *R*_f_: 0.34 (40% EtOAc/Hexane); IR (NaCl): ν 3363, 2933, 2253, 1729, 1509, 1250, 1179, 1034 cm^–1^; ^1^H NMR (300.13 MHz, MeOH-*d*_4_): δ 1.08 (d, 6H, H_v_, *J* = 6.8 Hz), 1.19 (d, 6H, H_v_, *J* = 6.8 Hz), 1.50 and 1.53 (2d, 3H, H_11_, *J* = 6.4 Hz), 1.70–1.95 (several m, 2H, H_2_), 2.68 (t, 2H, H_*y*_, *J* = 5.9 Hz), 3.08–3.41 (several m, 4H, H_5_ + H_6_), 3.60 (m, 2H, H_w_), 3.78 and 3.79 (2s, 6H, *Me*-DMT), 3.78 (m, 2H, H_*x*_), 4.01 (m, 1H, H_4_), 4.18 (m, 1H, H_1_), 4.41 (m, 1H, H_3_), 6.12 (m, 1H, H_10_), 6.85 (m, 4H, H_arom_), 7.18–7.63 (seveal m, 12H, H_arom_), 7.93 (m, 1H, H_arom_) ppm; ^13^C NMR (75.5 MHz, MeOH-*d*_4_): δ 20.9 (d, C_*y*_, *J* = 6.6 Hz), 22.4 (C_11_), 22.5 (C_11_), 24.9 (d, 2C_v_, *J* = 7.7 Hz), 25.0 (d, 2C_v_, *J* = 7.3 Hz), 38.0 (C_2_), 38.2 (C_2_), 44.4 (d, C_w_, *J* = 12.6 Hz), 45.0 (C_6_), 45.3 (C_6_), 55.7 (2 O-*C*H_3_), 59.7 (d, C_*x*_, *J* = 19.1 Hz), 65.2 (C_5_),65.3 (C_5_), 69.6 (C_10_), 77.0 (C_3_, cross-peak in HSQC), 78.8 (C_1_), 78.9 (C_1_), 86.8 (d, C_4_, *J* = 5.5 Hz), 87.5 (C_18_),114.1 (4C_g_), 119.5 (*C*N), 125.2 (CH_arom_), 127.8 (C_d_), 128.1 (CH_arom_), 128.23 (CH_arom_), 128.8 (2C_c_), 129.4 (2C_b_), 129.5 (CH_arom_), 131.3 (4C_f_), 134.8 (CH_arom_), 137.1 (C_e_), 137.2 (C_e_), 137.3 (C_e_), 139.8 (C_12_), 146.4 (C_a_), 149.0 (C_13_), 157.9 (*C*=O), 160.1 (2C_h_) ppm; ^31^P NMR (121.5 MHz, MeOH-*d*_4_): δ 147.6 ppm; HRMS (ESI^+^, *m*/*z*): calcd for C_45_H_56_N_4_O_10_P [M + H]^+^: 843.3729, found: 843.3732, calcd for C_45_H_55_N_4_NaO_10_P [M + Na]^+^: 865.3548, found: 865.3541.

#### 2.3 Preparation of 1-Acetylmercaptomethyl-1,2-dideoxy-d-erythro-pentofuranose Phosphoramidites **16α** and **16β**

##### 2.3.1 Synthesis of **9**, **10**, **11**, and **12**

Synthesis of **9α**, **10α**, **11α**, and **12α** was described previously by us.^19^ A procedure analogous to that afforded **9β**, **10β**, **11β**, and **12β**. Yields are indicated in Scheme 3.

##### 1,2-Dideoxy-**1β**-(methoxycarbonyl)-d-*erythro*-pentofuranose (**9β**)

Yellowish oil. *R*_f_: 0.36 (10% MeOH/CH_2_Cl_2_); IR (NaCl): ν 3387, 2954, 1738 cm^–1^; ^1^H NMR (300.13 MHz, MeOH-*d*_4_): δ 2.19 (m, 2H, H_2_), 3.57 (d, 2H, H_5_, *J* = 5.1 Hz), 3.75 (s, 3H, *Me*), 3.91 (dt, 1H, H_4_, *J* = 5.0, 2.8 Hz), 4.26 (dt, 1H, H_3_, *J* = 5.7, 2.9 Hz), 4.64 (dd, 1H, H_1_, *J* = 8.5, 7.4 Hz) ppm; ^13^C NMR (75.5 MHz, MeOH-*d*_4_): δ 39.7 (C_2_), 52.7 (O-*C*H_3_), 63.5 (C_5_), 73.2 (C_3_), 77.4 (C_1_), 89.5 (C_4_), 175.3 (*C*=O) ppm; HRMS (ESI^+^, *m*/*z*): calcd for C_7_H_12_NaO_5_ [M + Na]^+^: 199.0577, found: 199.0580.

##### 3,5-Bis-*O*-(*tert*-butyldimethylsilyl)-1,2-dideoxy-**1β**-(methoxycarbonyl)-d-*erythro*-pentofuranose (**10β**)

Viscous liquid. *R*_f_: 0.59 (20% EtOAc/Hexane); IR (NaCl): ν 2954, 2931, 2898, 2858, 1759, 1737 cm^–1^; ^1^H NMR (300.13 MHz, MeOH-*d*_4_): δ 0.08 (s, 3H, Si-*Me*), 0.09 (s, 3H, Si-*Me*), 0.108 (s, 3H, Si-*Me*), 0.113 (s, 3H, Si-*Me*), 0.91 (s, 9H, Si-^*t*^*Bu*), 0.92 (s, 9H, Si-^*t*^*Bu*), 2.12 (m, 2H, H_2_), 3.51 (dd, 1H, H_5_, *J* = 10.9, 6.5 Hz), 3.68 (dd, 1H, H_5_, *J* = 10.8, 4.2 Hz), 3.73 (s, 3H, O-*Me*), 3.89 (ddd, 1H, H_4_, *J* = 6.3, 4.3, 1.8 Hz), 4.42 (m, 1H, H_3_), 4.61 (dd, 1H, H_1_, *J* = 8.8, 7.4 Hz) ppm; ^13^C NMR (75.5 MHz, MeOH-*d*_4_): δ −5.32 (Si-*C*H_3_), −5.29 (Si-*C*H_3_), −4.53 (Si-*C*H_3_), −4.50 (Si-*C*H_3_), 18.7 (Si*C*Me_3_), 19.2 (Si*C*Me_3_), 26.3 (3 *C*H_3_-^*t*^Bu), 26.4 (3 *C*H_3_-^*t*^Bu), 39.8 (C_2_), 52.5 (O-*C*H_3_), 64.6 (C_5_), 75.0 (C_3_), 77.7 (C_1_), 90.0 (C_4_), 174.3 (*C*=O) ppm; HRMS (ESI^+^, *m*/*z*): calcd for C_19_H_40_NaO_5_Si_2_ [M + Na]^+^: 427.2306, found: 427.2308.

##### 3,5-Bis-*O*-(*tert*-butyldimethylsilyl)-1,2-dideoxy-**1β**-(hydroxymethyl)-d-*erythro*-pentofuranose (**11β**)

Viscous liquid. *R*_f_: 0.37 (20% EtOAc/Hexane); IR (NaCl): ν 3450, 2960, 2925, 1472, 1256 cm^–1^; ^1^H NMR (300.13 MHz, MeOH-*d*_4_): δ 0.08 (s, 6H, Si-*Me*), 0.10 (s, 6H, Si-*Me*), 0.91 (s, 9H, Si-^*t*^*Bu*), 0.92 (s, 9H, Si-^*t*^*Bu*), 1.84 (m, 2H, H_2_), 3.50 (m, 2H, H_6_), 3.60 (dd, 1H, H_5_, *J* = 11.6, 4.0 Hz), 3.65 (dd, 1H, H_5_, *J* = 10.8, 4.2 Hz), 3.79 (ddd, 1H, H_4_, *J* = 6.3, 4.2, 2.5 Hz), 4.21 (m, 1H, H_1_), 4.36 (dt, 1H, H_3_, *J* = 5.1, 2.6 Hz) ppm; ^13^C NMR (75.5 MHz, MeOH-*d*_4_): δ −5.3 (Si-*C*H_3_), −5.2 (Si-*C*H_3_), −4.5 (Si-*C*H_3_), −4.4 (Si-*C*H_3_), 18.9 (Si*C*Me_3_), 19.2 (Si*C*Me_3_), 26.3 (3 *C*H_3_-^*t*^Bu), 26.5 (3 *C*H_3_-^*t*^Bu), 38.0 (C_2_), 64.9 (C_5_), 65.4 (C_6_), 75.1 (C_3_), 80.6 (C_1_), 89.0 (C_4_) ppm; HRMS (ESI^+^, *m*/*z*): calcd for C_18_H_40_NaO_4_Si_2_ [M + Na]^+^: 399.2357, found: 399.2361.

##### 3,5-Bis-*O*-(*tert*-butyldimethylsilyl)-1,2-dideoxy-**1β**-(tosyloxy)methyl-d-*erythro*-pentofuranose (**12β**)

Viscous liquid. *R*_f_: 0.64 (20% EtOAc/Hexane); IR (NaCl): ν 2954, 2930, 2896, 2857, 1471, 1366, 1255 cm^–1^; ^1^H NMR (300.13 MHz, MeOH-*d*_4_): δ 0.02 (s, 3H, Si-*Me*), 0.04 (s, 3H, Si-*Me*), 0.07 (s, 6H, Si-*Me*), 0.88 (s, 9H, Si-^*t*^*Bu*), 0.89 (s, 9H, Si-^*t*^*Bu*), 1.80 (m, 2H, H_2_), 2.46 (s, 3H, Ts-*Me*), 3.41 (dd, 1H, H_5_, *J* = 10.9, 5.9 Hz), 3.53 (dd, 1H, H_5_, *J* = 10.9, 4.1 Hz), 3.75 (ddd, 1H, H_4_, *J* = 6.2, 4.1, 2.3 Hz), 3.95 (dd, 1H, H_6_, *J* = 10.5, 5.6 Hz), 4.10 (dd, 1H, H_6_, *J* = 10.5, 3.4 Hz), 4.29 (m, 2H, H_1_ + H_3_), 7.44 (d, 2H, H_arom_, *J* = 8.5 Hz), 7.79 (d, 2H, H_arom_, *J* = 8.4 Hz) ppm; ^13^C NMR (75.5 MHz, MeOH-*d*_4_): δ −5.3 (Si-*C*H_3_), −5.2 (Si-*C*H_3_), −4.6 (Si-*C*H_3_), −4.5 (Si-*C*H_3_), 18.8 (Si*C*Me_3_), 19.2 (Si*C*Me_3_), 21.6 (*C*H_3_-Ts), 26.3 (3 *C*H_3_-^*t*^Bu), 26.5 (3 ^*t*^Bu-*C*H_3_), 37.7 (C_2_), 64.6 (C_5_), 72.8 (C_6_), 74.8 (C_3_), 77.1 (C_1_), 89.2 (C_4_), 129.1 (2 C_arom_), 131.1 (2 C_arom_), 134.4 (C_ipso_), 146.5 (C_ipso_) ppm; HRMS (ESI^+^, *m*/*z*): calcd for C_25_H_47_O_6_SSi_2_ [M + H]^+^: 531.2626, found: 531.2633.

##### 2.3.2 Synthesis of **13α**/**13β**

A solution of potassium thioacetate (1.7 equiv) in anhydrous DMF (0.5 M) was added dropwise to a solution of **12α/12β** in anhydrous DMF (0.3 M). The reaction was stirred 6 h at 65 °C, and the mixture was dissolved in H_2_O and extracted with Et_2_O. The organic layer was dried, filtered, and evaporated to dryness. The residue was purified by column chromatography (gradient eluent 5–15% EtOAc/Hexane) to give **13α** (70% yield) or **13β** (75% yield).

##### **1α**-(Acetylmercaptomethyl)-3,5-bis-*O*-(*tert*-butyldimethylsilyl)-1,2-dideoxy-d-*erythro*-pentofuranose (**13α**)

Yellow oil. *R*_f_: 0.63 (20% EtOAc/Hexane); IR (NaCl): ν 2954, 1697, 1257, 1109, 626 cm^–1^; ^1^H NMR (300.13 MHz, CDCl_3_): δ 0.03 (s, 3H, Si-*Me*), 0.04 (s, 3H, Si-*Me*), 0.06 (s, 6H, Si-*Me*), 0.87 (s, 18H, Si-^*t*^*Bu*), 1.72 (dt, 1H, H_2_, *J* = 13.0, 4.2 Hz), 2.21 (ddd, 1H, H_2_, *J* = 13.2, 7.3, 6.0 Hz), 2.33 (s, 3H, CO-*Me*), 3.12 (dd, 1H, H_6_, *J* = 13.6, 5.6 Hz), 3.19 (dd, 1H, H_6_, *J* = 13.5, 7.4 Hz), 3.48 (dd, 1H, H_5_, *J* = 10.8, 5.6 Hz), 3.60 (dd, 1H, H_5_, *J* = 10.9, 3.8 Hz), 3.89 (m, 1H, H_4_), 4.15 (m, 1H, H_1_), 4.34 (dt, 1H, H_3_, *J* = 6.3, 3.4 Hz) ppm; ^13^C NMR (75.5 MHz, CDCl_3_): δ −5.3 (Si-*C*H_3_), −5.2 (Si-*C*H_3_), −4.7 (Si-*C*H_3_), −4.6 (Si-*C*H_3_), 18.1 (Si*C*Me_3_), 18.5 (Si*C*Me_3_), 25.9 (^t^Bu-*C*H_3_), 26.1 (^t^Bu-*C*H_3_), 30.7 (CO-*C*H_3_), 34.9 (C_6_), 39.9 (C_2_), 63.6 (C_5_), 73.7 (C_3_), 78.0 (C_1_), 87.2 (C_4_), 195.7 (*C*=O) ppm; HRMS (ESI^+^, *m*/*z*): calcd for C_20_H_43_O_4_SSi_2_ [M + H]^+^: 435.2415, found: 435.2413, calcd for C_20_H_42_NaO_4_SSi_2_ [M + Na]^+^: 457.2235, found: 457.2241, calcd for C_20_H_42_KO_4_SSi_2_ [M+K]^+^: 473.1974, found: 473.1971.

##### **1β**-(Acetylmercaptomethyl)-3,5-bis-*O*-(*tert*-butyldimethylsilyl)-1,2-dideoxy-d-*erythro*-pentofuranose (**13β**)

Yellow oil. *R*_f_: 0.75 (20% EtOAc/Hexane); IR (NaCl): ν 2955, 1961, 1255, 1108, 626 cm^–1^; ^1^H NMR (300.13 MHz, CDCl_3_): δ 0.05 (s, 12H, Si-*Me*), 0.87 (s, 9H, Si-^*t*^*Bu*), 0.89 (s, 9H, Si-^*t*^*Bu*), 1.67 (ddd, 1H, H_2_, *J* = 12.6, 9.6, 5.6 Hz), 1.85 (ddd, 1H, H_2_, *J* = 12.6, 5.5, 2.2 Hz), 2.34 (s, 3H, CO-*Me*), 2.98 (dd, 1H, H_6_, *J* = 13.6, 6.5 Hz), 3.18 (dd, 1H, H_6_, *J* = 13.6, 4.8 Hz), 3.45 (dd, 1H, H_5_, *J* = 10.7, 6.1 Hz), 3.61 (dd, 1H, H_5_, *J* = 10.8, 4.0 Hz), 3.79 (m, 1H, H_4_), 4.28 (m, 2H, H_1_ + H_3_) ppm; ^13^C NMR (75.5 MHz, CDCl_3_): δ −5.3 (Si-*C*H_3_), −5.2 (Si-*C*H_3_), −4.6 (Si-*C*H_3_),), −4.5 (Si-*C*H_3_), 18.1 (Si*C*Me_3_), 18.5 (Si*C*Me_3_), 25.9 (^t^Bu-*C*H_3_), 26.1 (^t^Bu-*C*H_3_), 30.7 (CO-*C*H_3_), 33.8 (C_6_), 40.1 (C_2_), 63.7 (C_5_), 74.1 (C_3_), 77.1 (C_1_), 88.0 (C_4_), 195.6 (*C*=O) ppm; HRMS (ESI^+^, *m*/*z*): calcd for C_20_H_43_O_4_SSi_2_ [M + H]^+^: 435.2415, found: 435.2416, calcd for C_20_H_42_NaO_4_SSi_2_ [M + Na]^+^: 457.2235, found: 457.2235, calcd for C_20_H_42_KO_4_SSi_2_ [M+K]^+^: 473.1974, found: 473.1974.

##### 2.3.3 Synthesis of **14α**/**14β**

(−)-CSA (2 equiv) was added to a solution of **13α/13β** in anhydrous MeOH (0.1 M) at 0 °C and the reaction was stirred at rt during 2 h. Solid NaHCO_3_ was then added and the mixture was stirred for a further 5 min. The solvent was evaporated, and the crude product was subjected to column chromatography (10% MeOH/CH_2_Cl_2_) to afford **14α** (80% yield) or **14β** (80% yield).

##### **1α**-(Acetylmercaptomethyl)-1,2-dideoxy-d-*erythro*-pentofuranose (**14α**)

Clear oil. *R*_f_: 0.47 (10% MeOH/CH_2_Cl_2_); IR (NaCl): ν 3374, 2931, 1692, 629 cm^–1^; ^1^H NMR (300.13 MHz, MeOH-*d*_4_): δ 1.71 (ddd, 1H, H_2_, *J* = 12.7, 6.7, 5.7 Hz), 2.30 (m, 1H, H_2_), 2.33 (s, 3H, CO-*Me*), 3.10 (dd, 1H, H_6_, *J* = 13.6, 5.9 Hz), 3.20 (dd, 1H, H_6_, *J* = 13.6, 6.4 Hz), 3.51 (dd, 1H, H_5_, *J* = 11.8, 5.1 Hz), 3.59 (dd, 1H, H_5_, *J* = 11.8, 3.9 Hz), 3.82 (q, 1H, H_4_, *J* = 4.0 Hz), 4.16 (m, 1H, H_1_), 4.21 (m, 1H, H_3_) ppm; ^13^C NMR (75.5 MHz, MeOH-*d*_4_): δ 30.4 (CO-*C*H_3_), 35.2 (C_6_), 40.4 (C_2_), 63.3 (C_5_), 73.3 (C_3_), 78.4 (C_1_), 87.4 (C_4_), 197.0 (*C*=O) ppm; HRMS (ESI^+^, *m*/*z*): calcd for C_8_H_15_O_4_S [M + H]^+^: 207.0686, found: 207.0688, calcd for C_8_H_14_NaO_4_S [M + Na]^+^: 229.0505, found: 229.0506, calcd for C_8_H_14_KO_4_S [M+K]^+^: 245.0244, found: 245.0245.

##### **1β**-(Acetylmercaptomethyl)-1,2-dideoxy-d-*erythro*-pentofuranose (**14β**)

Clear oil. *R*_f_: 0.47 (10% MeOH/CH_2_Cl_2_); IR (NaCl): ν 3390, 2930, 1691, 630 cm^–1^; ^1^H NMR (300.13 MHz, MeOH-*d*_4_): δ 1.76 (ddd, 1H, H_2_, *J* = 13.1, 9.6, 6.1 Hz), 1.92 (ddd, 1H, H_2_, *J* = 13.0, 5.6, 2.2 Hz), 2.33 (s, 3H, CO-*Me*), 3.10 (d, 2H, H_6_, *J* = 5.7 Hz), 3.52 (d, 2H, H_5_, *J* = 5.0 Hz), 3.77 (m, 1H, H_4_), 4.21 (m, 2H, H_1_ + H_3_) ppm; ^13^C NMR (75.5 MHz, MeOH-*d*_4_): δ 30.4 (CO-*C*H_3_), 34.2 (C_6_), 40.7 (C_2_), 63.9 (C_5_), 74.0 (C_3_), 78.6 (C_1_), 89.0 (C_4_), 196.9 (*C*=O) ppm; HRMS (ESI^+^, *m*/*z*): calcd for C_8_H_15_O_4_S [M + H]^+^: 207.0686, found: 207.0684, calcd for C_8_H_14_NaO_4_S [M + Na]^+^: 229.0505, found: 229.0502, calcd for C_8_H_14_KO_4_S [M+K]^+^: 245.0244, found: 245.0240.

##### 2.3.4 Synthesis of **15α**/**15β**

A procedure analogous to that described for the synthesis of **4α/4β**, starting from **14α/14β**, gave **15α** (80% yield) or **15β** (85% yield).

##### **1α**-(Acetylmercaptomethyl)-1,2-dideoxy-5-*O*-(4,4′-dimethoxytrityl)-d-*erythro*-pentofuranose (**15α**)

Clear oil. *R*_f_: 0.32 (40% EtOAc/Hexane); IR (NaCl): ν 3413, 2929, 1692, 1508, 625 cm^–1^; ^1^H NMR (300.13 MHz, MeOH-*d*_4_): δ 1.69 (ddd, 1H, H_2_, *J* = 12.6, 6.9, 5.4 Hz), 2.33 (m, 1H, H_2_), 2.34 (s, 3H, CO-*Me*), 3.12 (m, 4H, H_5_ + H_6_), 3.76 (s, 6H, *Me*-DMT), 3.98 (dt, 1H, H_4_, *J* = 5.1, 3.9 Hz), 4.23 (m, 2H, H_1_ + H_3_), 6.84 (d, 4H, H_g_, *J* = 8.9 Hz), 7.24 (m, 3H, H_c_ + H_d_), 7.31 (d, 4H, H_f_, *J* = 8.9 Hz), 7.44 (d, 2H, H_b_, *J* = 7.0 Hz)ppm; ^13^C NMR (75.5 MHz, MeOH-*d*_4_): δ 30.5 (CO-*C*H_3_), 35.2 (C_6_), 40.7 (C_2_), 55.7 (2 O-*C*H_3_), 65.5 (C_5_), 74.2 (C_3_), 78.8 (C_1_), 86.6 (C_4_), 87.4 (C_10_), 114.0 (4C_g_), 127.7 (C_d_), 128.7 (2C_c_), 129.3 (2C_b_), 131.3 (4C_f_), 137.3 (C_e_), 137.4 (C_e_), 146.5 (C_a_), 160.0 (2C_h_), 196.9 (*C*=O)ppm; HRMS (ESI^+^, *m*/*z*): calcd for C_29_H_32_NaO_6_S [M + Na]^+^: 531.1812, found: 531.1782, calcd for C_29_H_32_KO_6_S [M+K]^+^: 547.1551, found: 547.1520.

##### **1β**-(Acetylmercaptomethyl)-1,2-dideoxy-5-*O*-(4,4′-dimethoxytrityl)-d-*erythro*-pentofuranose (**15β**)

Clear oil. *R*_f_: 0.29 (40% EtOAc/Hexane); IR (NaCl): ν 3402, 2929, 1693, 1508, 627 cm^–1^; ^1^H NMR (300.13 MHz, MeOH-*d*_4_): δ 1.78 (ddd, 1H, H_2_, *J* = 13.0, 9.6, 5.8 Hz), 1.90 (ddd, 1H, H_2_, *J* = 13.1, 5.7, 2.2 Hz), 2.29 (s, 3H, CO-*Me*), 3.12 (m, 4H, H_5_+ H_6_), 3.76 (s, 6H, *Me*-DMT), 3.90 (m, 1H, H_4_), 4.22 (m, 1H, H_3_), 4.29 (dq, 1H, H_1_, *J* = 11.0, 5.6 Hz), 6.84 (d, 4H, H_g_, *J* = 8.9 Hz), 7.22 (m, 3H, H_c_ + H_d_), 7.33 (d, 4H, H_f_, *J* = 8.9 Hz), 7.46 (d, 2H, H_b_, *J* = 7.0 Hz) ppm; ^13^C NMR (75.5 MHz, MeOH-*d*_4_): δ 30.5 (CO-*C*H_3_), 34.3 (C_6_), 40.4 (C_2_), 55.7 (2 O-*C*H_3_), 65.6 (C_5_), 74.5 (C_3_), 78.5 (C_1_), 87.4 (C_10_), 87.8 (C_4_), 114.0 (4C_g_), 127.2 (C_d_), 128.7 (2C_c_), 129.4 (2C_b_), 131.3 (4C_f_), 137.3 (C_e_), 137.4 (C_e_), 146.5 (C_a_), 160.1 (2C_h_), 196.7 (*C*=O) ppm; HRMS (ESI^+^, *m*/*z*): calcd for C_29_H_32_NaO_6_S [M + Na]^+^: 531.1812, found: 531.1808, calcd for C_29_H_32_KO_6_S [M+K]^+^: 547.1551, found: 547.1547.

##### 2.3.5 Synthesis of **16α**/**16β**

A procedure analogous to that described for the synthesis of **5α/5β**, starting from **15α/15β**, gave **16α** (72% yield) or **16β** (68% yield).

##### **1α**-(Acetylmercaptomethyl)-1,2-dideoxy-d-*erythro*-pentofuranosyl-3-*O*-(2-cyanoethyl-*N,N*-diisopropyl)phosphoramidite (**16α-A**)

Yellowish oil. *R*_f_: 0.48 (40% EtOAc/Hexane); IR (NaCl): ν 2965, 2226, 1961, 1509, 1179, 1034, 590 cm^–1^; ^1^H NMR (300.13 MHz, MeOH-*d*_4_): δ1.15 (d, 6H, H_v_, *J* = 6.9 Hz), 1.18 (d, 6H, H_v_, *J* = 6.9 Hz), 1.84 (m, 1H, H_2_), 2.34 (s, 3H, CO-*Me*), 2.37 (m, 1H, H_2_), 2.51 (t, 2H, H_*y*_, *J* = 6.0 Hz), 3.04–3.26 (several m, 4H, H_5_ + H_6_), 3.62 (m, 4H, H_w_ + H_*x*_), 3.78 (s, 6H, *Me*-DMT), 4.12 (m, 1H, H_4_), 4.27 (m, 1H, H_1_), 4.47 (m, 1H, H_3_), 6.86 (d, 4H, H_g_, *J* = 8.9 Hz), 7.25 (m, 3H, H_c_ + H_d_), 7.31 (d, 4H, H_f_, *J* = 8.9 Hz), 7.44 (d, 2H, H_b_, *J* = 7.0 Hz) ppm; ^31^P NMR (121.5 MHz, MeOH-*d*_4_): δ 148.1 ppm; HRMS (ESI^+^, *m*/*z*): calcd for C_38_H_50_N_2_O_7_PS [M + H]^+^: 709.3071, found: 709.3063, calcd for C_38_H_49_N_2_NaO_7_PS [M + Na]^+^: 731.2890, found: 731.2884.

##### **1α**-(Acetylmercaptomethyl)-1,2-dideoxy-d-*erythro*-pentofuranosyl-3-*O*-(2-cyanoethyl-*N,N*-diisopropyl)phosphoramidite (**16α-A+B**)

Yellowish oil. *R*_f_: 0.48 and 0.44 (40% EtOAc/Hexane); IR (NaCl): ν 2967, 2231, 1970, 1509, 1178, 1033, 587 cm^–1^; ^31^P NMR (121.5 MHz, MeOH-*d*_4_): δ 148.0, 148.1 ppm.

##### **1β**-(Acetylmercaptomethyl)-1,2-dideoxy-d-*erythro*-pentofuranosyl-3-*O*-(2-cyanoethyl-*N,N*-diisopropyl)phosphoramidite (**16β-A**)

Yellowish oil. *R*_f_: 0.65 (40% EtOAc/Hexane); IR (NaCl): ν 2967, 2254, 1722,1509, 1178, 1033, 583 cm^–1^; ^1^H NMR (300.13 MHz, MeOH-*d*_4_): δ 1.15 (d, 6H, H_v_, *J* = 6.8 Hz), 1.18 (d, 6H, H_v_*J* = 6.8 Hz), 1.87 (m, 1H, H_2_), 2.01 (m, 1H, H_2_), 2.30 (s, 3H, CO-*Me*), 2.52 (t, 2H, H_*y*_, *J* = 5.9 Hz), 3.16 (m, 4H, H_5_ + H_6_), 3.62 (m, H_w_ + H_*x*_), 3.78 (s, 6H, *Me*-DMT), 4.02 (m, 1H, H_4_), 4.30 (m, 1H, H_1_), 4.45 (m, 1H, H_3_), 6.86 (d, 4H, H_g_, *J* = 8.9 Hz), 7.26 (m, 3H, H_c_ + H_d_), 7.33 (d, 4H, H_f_, *J* = 8.9 Hz), 7.46 (d, 2H, H_b_, *J* = 7.0 Hz) ppm; ^13^C NMR (75.5 MHz, MeOH-*d*_4_): δ 20.9 (d, C_*y*_, *J* = 7.2 Hz), 24.9 (d, C_v_, *J* = 7.4 Hz), 25.0 (d, C_v_, *J* = 7.4 Hz), 30.5 (CO-*C*H_3_), 34.1 (C_6_), 39.7 (d, C_2_, *J* = 4.3 Hz), 44.4 (d, 2C_w_, *J* = 12.6 Hz), 55.7 (2 O-*C*H_3_), 59.7 (d, C_*x*_, *J* = 18.4 Hz), 64.9 (C_5_), 76.3 (d, C_3_, *J* = 16.8 Hz), 78.7 (C_1_), 87.1 (d, C_4_, *J* = 4.6 Hz), 87.4 (C_10_), 114.1 (4C_g_), 119.3 (*C*N), 127.8 (C_d_), 128.8 (2C_c_), 129.4 (2C_b_), 131.4 (4C_f_), 137.3 (C_e_), 137.4 (C_e_), 146.5 (C_a_), 160.1 (2C_h_), 196.7 (*C*=O) ppm; ^31^P NMR (121.5 MHz, MeOH-*d*_4_): δ 148.0 ppm; HRMS (ESI^+^, *m*/*z*): calcd for C_38_H_50_N_2_O_7_PS [M + H]^+^: 709.3071, found: 709.3079, calcd for C_38_H_49_N_2_NaO_7_PS [M + Na]^+^: 731.2890, found: 731.2899, calcd for C_38_H_49_N_2_KO_7_PS [M+K]^+^: 747.2630, found: 747.2642.

##### **1β**-(Acetylmercaptomethyl)-1,2-dideoxy-d-*erythro*-pentofuranosyl-3-*O*-(2-cyanoethyl-*N,N*-diisopropyl)phosphoramidite (**16β-B**)

Yellowish oil. *R*_f_: 0.58 (40% EtOAc/Hexane); IR (NaCl): ν 2966, 2253, 1963, 1509, 1178, 1035, 588 cm^–1^; ^1^H NMR (300.13 MHz, MeOH-*d*_4_): δ 1.08 (d, 6H, H_v_, *J* = 6.8 Hz), 1.18 (d, 6H, H_v_*J* = 6.8 Hz), 1.87 (m, 1H, H_2_, *J* = 13.1, 9.3, 5.9 Hz), 2.12 (m, 1H, H_2_, *J* = 12.0, 5.2, 2.0 Hz), 2.31 (s, 3H, CO-*Me*), 2.69 (t, 2H, H_*y*_, *J* = 5.9 Hz), 3.16 (m, 4H, H_5_ + H_6_), 3.59 (m, 2H, H_w_), 3.79 (s, 6H, *Me*-DMT), 3.80 (m, 2H, H_*x*_),4.00 (m, 1H, H_4_), 4.31 (m, 1H, H_1_), 4.44 (m, 1H, H_3_), 6.87 (d, 4H, H_g_, *J* = 8.9 Hz), 7.26 (m, 3H, H_c_ + H_d_), 7.34 (d, 4H, H_f_, *J* = 8.9 Hz), 7.47 (d, 2H, H_b_, *J* = 7.0 Hz) ppm; ^13^C NMR (75.5 MHz, MeOH-*d*_4_): δ 20.9 (d, C_*y*_, *J* = 6.9 Hz), 24.9 (d, C_v_, *J* = 7.2 Hz), 25.0 (d, C_v_, *J* = 7.0 Hz), 30.5 (CO-*C*H_3_), 34.1 (C_6_), 39.7 (d, C_2_, *J* = 3.1 Hz), 44.4 (d, 2C_w_, *J* = 12.4 Hz), 55.7 (2 O-*C*H_3_), 59.8 (d, C_*x*_, *J* = 19.1 Hz), 65.1 (C_5_), 76.7 (d, C_3_, *J* = 16.5 Hz), 78.6 (C_1_), 86.9 (d, C_4_, *J* = 5.4 Hz), 87.4 (C_10_), 114.1 (4C_g_), 119.4 (*C*N), 127.8 (C_d_), 128.7 (2C_c_), 129.4 (2C_b_), 131.4 (4C_f_), 137.2 (C_e_), 137.3 (C_e_), 146.5 (C_a_), 160.1 (2C_h_), 196.8 (*C*=O) ppm; ^31^P NMR (121.5 MHz, MeOH-*d*_4_): δ 147.7 ppm; HRMS (ESI^+^, *m*/*z*): calcd for C_38_H_50_N_2_O_7_PS [M + H]^+^: 709.3071, found: 709.3085, calcd for C_38_H_49_N_2_NaO_7_PS [M + Na]^+^: 731.2890, found: 731.2905, calcd for C_38_H_49_N_2_KO_7_PS [M+K]^+^: 747.2630, found: 747.2661.

### 3 Synthesis of Solid Supports Functionalized with 1,2-Dideoxy-d-erythro-pentofuranose Derivatives

#### 3.1 Preparation of the 3-*O*-Succinyl-1,2-dideoxy-d-*erythro*-pentofuranose Derivatives **17α**, **17β**, **18α**, **18β**, **19α**, and **19β**

5-*O*-DMT-monomers (**4α**, **4β**, **7α**, **7β**, **15α**, or **15β**) were dried twice by evaporation with anhydrous CH_2_Cl_2_ and dissolved in anhydrous CH_2_Cl_2_ (0.02 M). Then, 1.5 equiv of succinic anhydride and 1.5 equiv of DMAP were added, and the reaction was stirred at rt overnight. After the addition of CH_2_Cl_2_, the mixture was washed with 0.1 M NaH_2_PO_4_ (pH 5). The organic layer was dried with Na_2_SO_4_, filtrated, and concentrated to dryness giving place to 3-*O*-succinate-2-deoxy-d-ribofuranose derivatives **17α**, **17β**, **18α**, **18β**, **19α**, and **19β**. The resulting succinates were used directly for the functionalization of the supports without further purification.

#### 3.2 Incorporation of the 3-*O*-Succinates to an LCAA-CPG Solid Support

The 5-*O*-DMT-3-*O*-succinate derivatives (**17α**, **17β**, **18α**, **18β**, **19α**, or **19β**) obtained in the previous step and 1 equiv of DMAP were dissolved in acetonitrile (0.1 M). Next, 1 equiv of 2,2′-dithio-bis(5-nitropyridine) dissolved in a mixture (0.3 M) of acetonitrile:CH_2_Cl_2_ (1:3) was added. Then, this solution was added to 1 equiv of Ph_3_P in acetonitrile 80 μL. This final solution was poured to a vial containing 0.5 equiv LCAA-CPG (70 μmol/g) that had been previously washed with acetonitrile. After 3 h of reaction, the resin was washed with CH_2_Cl_2_ and acetonitrile. Finally, a 1:1 mixture of acetic anhydride/Py/THF and methylimidazole/THF was added to the resin for 5 min. The solid support was washed with CH_2_Cl_2_ and acetonitrile and dried out. The degree of functionalization of all of the supports ranged around 20–25 μmol/g.

### 4 Synthesis of Pentafluorophenyl Fatty Acid Esters **25**

#### 4.1 Preparation of Pentafluorophenyl Oleate (**25a**)

Oleic acid **23a** (1 mmol, 282.46 mg) was dissolved in CH_2_Cl_2_ (1 mL/mmol). Et_3_N (16 mmol, 2.25 mL) and pentafluorophenyl trifluoroacetate **24** (4 mmol, 0.67 mL) were added to the solution. Then, the reaction mixture was stirred at rt for 1 h. Afterward, the reaction mixture was diluted in CH_2_Cl_2_ (6 mL/mmol) and washed with aqueous saturated NaHCO_3_ solution (5 mL/mmol) and 1 M NaH_2_PO_4_ solution (5 mL/mmol). The organic layer was separated, dried out with Na_2_SO_4_, filtered, and concentrated under reduced pressure. The crude product was purified by silica gel column chromatography and eluted with CH_2_Cl_2_/Hexane (1:1, v/v) to yield the desired oleic ester **25a** as a yellowish oil (420 mg, 93%). ^1^H NMR (CDCl_3_, 400.13 MHz): δ 0.86 (t, 3H, C*H*_3_, *J* = 7.0 Hz), 1.44–1.21 (m, 20H, (C*H*_2_)_n_), 1.70–1.82 (m, 2H,C*H*_2_CH_2_CO), 2.01 (m, 4H, C*H*_2_CH=CHC*H*_2_), 2.64 (t, 2H, C*H*_2_CO, *J* = 7.4 Hz), 5.30–5.38 (m, 2H, CH=CH) ppm; ^13^C NMR (CDCl_3_, 100.6 MHz): δ 14.0 (*C*H_3_), 22.6, 24.7, 27.1, 27.2, 28.8, 28.9, 29.0, 29.3, 29.5, 29.6, 29.7 (*C*H_2_), 31.9 (CO*C*H_2_), 33.3 (*C*H_2_CH=CH), 129.6, 130.0 (CH=CH), 136.5, 138.0, 139.0, 139.8, 140.6 (C_arom_), 142.4 (C_arom_O), 169.5 (CO) ppm; ^19^F NMR (CDCl_3_, 376.5 MHz): δ −162.5–162.7 (m, 2F), −158.4 (t, 1F, *J* = 21.6 Hz), −152.8–153.1 (m, 2F) ppm.

#### 4.2 Preparation of Pentafluorophenyl Palmitate (**25b**)

The palmitic acid ester **25b** was synthesized similarly to what has been described above for the pentafluorophenyl oleate. In this case, palmitic acid **23b** (1 mmol, 256.4 mg) was dissolved in 10 mL of CH_2_Cl_2_ due to solubility issues, and the reaction mixture was stirred at rt overnight. The isolation and purification steps were also mentioned in the preparation of the pentafluorophenyl oleate. The desired palmitic ester **25b** was obtained as a white solid (407 mg, 96%). ^1^H NMR (CDCl_3_, 400.13 MHz): δ 0.86 (t, 3H, Me, *J* = 6.8 Hz), 1.24 (s, 24H, (C*H*_2_)_n_), 1.75 (m, 2H, C*H*_2_CH_2_CO), 2.64 (t, 2H, C*H*_2_CO, *J* = 7.4 Hz) ppm; ^13^C NMR (CDCl_3_, 100.6 MHz): δ 14.1 (*C*H_3_), 22.6, 24.7, 28.8, 29.1, 29.3, 29.4, 29.5, 29.6, 29.6, 29.6, 29.7, 31.9 (*C*H_2_), 33.3 (CO*C*H_2_), 142.3–137.5 (6C_arom_), 169.6 (CO) ppm; ^19^F NMR (CDCl_3_, 376.5 MHz): δ −162.5–162.7 (m, 2F), −158.3 (t, 1F, *J* = 21.6 Hz), −152.8–152.9 (m, 2H) ppm.

### 5 Synthesis, Purification, and Characterization of Oligonucleotides Incorporating Monomers **4α**, **4β**, **7α**, **7β**, **15α**, and **15β**

#### 5.1 Oligonucleotide Synthesis

Oligonucleotide sequences, shown in Table 1, were synthesized on several batches between 0.5 and 1 μmol scale. In all cases, the 0.5–1 μmol standard solid-phase phosphoramidite chemistry protocols were carried out using an automatic DNA synthesizer.^23^ The 1,2-dideoxy-d-*erythro*-pentofuranose derivatives were site specifically inserted at 5′- and 3′-ends of the desired sequences. The solid supports of each one of them were used to introduce these modifications at the 3′-end of the sequence, and the corresponding phosphoramidites were incorporated at the 5′-end of the desired sequence. All the oligonucleotides were synthesized DMT-ON.

#### 5.2 Oligonucleotide Deprotection and Purification

According to the derivatives introduced in the sequence, different deprotection procedures were used for its deprotection. The 5′-*O*-DMT group of the 3′-modified gapmer with each one of the six derivatives **4α**, **4β**, **7α**, **7β**, **15α**, and **15β** were removed with a solution of 3% TCA in CH_2_Cl_2_ on the solid support.

The solid support of the Gapmer containing the **15α** and **15β** nucleoside derivatives either at the 3′-end or at the 5′-end and RS**15α** were treated with a solution of 1% DBU in acetonitrile followed by a couple of washes with acetonitrile and followed with a wash with a solution of 1% Et_3_N/acetonitrile for 1 min.

All the gapmer sequences containing only one **4α**, **4β**, **7α**, and **7β** derivative in its 3′- or 5′-end and the sequences RS**4α**, RS**7β**, and **7α**gapmer**4α** were treated with 32% aqueous ammonia solution at 55 °C overnight. The RS**15α** and the four gapmer**15α**, gapmer**15β**, **15α**gapmer, and **15β**gapmer were deprotected with the same ammonium solution with 0.1 M DTT. Then, the 5′-*O*-DMT group of the three RS sequences (**4α**, **7β**, **15α**) were removed by the direct addition of the ammonium solution over an OPC cartridge. Then, all the solutions of the RS sequences (**4α**, **7β**, and **15α**) and the 5′- and 3′-gapmers were desalted on a Sephadex G-25 using water as eluent.

The final products of RS**4α**, RS**7β**, RS**15α**, and the 3′-end-modified gapmers were HPLC analyzed with the DMT-OFF method with column D at a flow rate of 0.7 mL/min and an increasing gradient of acetonitrile (0% to 50%) over 0.1 M aqueous triethylammonium acetate, during 20 min.

The 5′-end gapmers (**4α**, **4β**, **7α**, **7β**, **15α**, and **15β**) were HPLC purified with the DMT-ON method with the column B using a flow rate of 2 mL/min and an increasing gradient of acetonitrile (0% to 70%) over 0.1 M aqueous triethylammonium acetate, during 20 min. The product fractions were collected and concentrated. The resulting products were detritylated by treating them with 1 mL of 50% acetic acid solution for 30 min at rt followed with extraction with Et_2_O. The deprotected oligonucleotides were desalted in a Sephadex column and analyzed by HPLC.

The length and homogeneity of all the modified oligonucleotide sequences were verified by MALDI-TOF. The retention time for the oligonucleotide and the calculated and found mass are shown in Table 1.

#### 5.3 Removal of the Photolabile Protecting Group of Oligonucleotides Modified with **7α** and **7β** Nucleoside Derivatives

The elimination of the photolabile protecting group NPEC from the oligonucleotide sequences was done directly on the solid support or in solution, after the release of the oligonucleotide from the support.

The oligonucleotides already detached from the solid support were exposed to irradiation at 340 nm (blacklight) for different periods of time (15, 30, 45, 60, and 120 min) in a solution of 100 μL H_2_O/acetonitrile (1:1, v/v). The samples of oligonucleotide still attached to the solid support were suspended in the same solvent conditions and placed under the UV–vis lamp for the 1, 2, and 6 h. Then, the oligonucleotides were deprotected and purified as explained in the previous section (section 5.2).

### 6 Preparation of Oligonucleotide Conjugates

#### 6.1 Oligonucleotides Conjugated with Fluorescein Isothiocyanate

The gapmer**4α** was left to react with fluorescein isothiocyanate (FITC) through its free amino group as follows. 52 nmol of gapmer**4α** was dissolved in 250 μL of an aqueous solution of 0.1 M NaHCO_3_ (pH 9) and 10 equiv of FITC (0.2 mg, 520 nmol) dissolved in 250 μL DMF was added and left to react at rt for 8 h. Then, 10 additional equiv of FITC was added and the mixture was left to react overnight at rt. The mixtures were concentrated to dryness and the residue resuspended in 1 mL of water. The solution was desalted by Sephadex G-25 and analyzed by HPLC.

#### 6.2 Conjugation Reactions in Solution

Oligonucleotides containing **7α** or **7β** nucleoside derivatives were dissolved in 350 μL carbonate buffer solution (pH 9.0), DMF, and acetonitrile (1:4:2, v:v:v). After that, 20 μL Et_3_N and 10 equiv of pentafluorophenyl oleate or palmitate were added, and the reaction mixture was stirred overnight at rt. The solution was concentrated to dryness. Then, the products were redissolved in water and desalted in a Sephadex column and HPLC purified. The yield of the final products obtained in each conjugation is shown in Table 3.

#### 6.3 Conjugation Reactions on the Solid Support

DMF (200 μL), Et_3_N (20 μL), and 10 equiv of oleoyl chloride or the corresponding ester were added to the oligonucleotides containing **7α** or **7β** nucleosides derivatives attached to either the 5′-end or the 3′-end and attached to the solid support. The reaction mixtures were left at rt for 2 h. Next, the excess of chemicals was washed off. The resulting solid supports were washed with acetonitrile and dried. Then, the solid supports were treated with ammonia for the removal of protecting groups and its release from the resin. The resulting oligonucleotide-conjugates were desalted and purified by HPLC. The yield of the final products obtained in each conjugation is shown in Table 3.

#### 6.4 Oligonucleotide Double Conjugation with Fluorescein Isothiocyanate and Oleic Acid (FITC-**7α**gapmer**4α**-oleic)

The **7α**gapmer**4α** first was photolyzed during 6 h, and then washed with acetonitrile and DMF. Then, it was left to react with fluorescein isothiocyanate (FITC) through its free amino group in the solid support as follows. 0.5 μmol of **7α**-gapmer**4α** was suspended in 100 μL of DMF, and 20 equiv of TEA (2 μL 10 μmol) was added, and 20 equiv of FITC (4 mg, 10 μmol) dissolved in 250 μL DMF was added and left to react at rt for 2 h. The reaction was washed with acetonitrile and dried. Then, the solid support was treated with 32% aqueous ammonia solution at 55 °C overnight. The solution was desalted by Sephadex G-25 and dried. Next, the FITC-**7α**-gapmer**4α** oligonucleotide (24 nmol) was dissolved in 70 μL carbonate buffer solution (pH 9.0), DMF and acetonitrile (1:4:2, v:v:v). After that, 1 μL Et_3_N and 20 equiv of pentafluorophenyl oleate were added, and the reaction mixture was stirred overnight at rt. The solution was concentrated to dryness. Successively, the product was redissolved in water and desalted in a Sephadex column and HPLC purified. The yield of the final products obtained in each conjugation is shown in Table 3.

## Supporting Information Available

Level of purity is indicated by the inclusion of copies of ^1^H, ^13^C, ^31^P, and DEPT NMR spectra; in addition, some 2D NMR experiments are shown, which were used to assign the peaks (PDF)

## Dedication

This paper is dedicated to the memory of Prof. Enrique Pedroso.

## Abbreviations

CPG: controlled pore glass
DBU: 1,8-diazabicyclo[5.4.0]undec-7-ene
DMAP: *N,N*-dimethylaminopyridine
DMF: *N,N*-dimethylformamide
DMT: dimethoxytrityl
DMSO: dimethylsulfoxide
DTT: dithiothreitol
FITC: fluorescein isothiocyanate
LCAA-CPG: long chain amino alkyl-controlled pore glass
MALDI: matrix-assisted laser disorption/ionization
NPEC: 1-(2-nitrophenyl)ethoxycarbonyl
OPC: oligonucleotide purification cartridge
Py: pyridine
RP-HPLC: reverse phase high performance liquid chromatography
TCA: trichloroacetic acid
TOF: time of flight
