# Supplementary material for: Oligonucleotides Containing 1-Aminomethyl or 1-Mercaptomethyl-2-deoxy-d-ribofuranoses: Synthesis, Purification, Characterization, and Conjugation with Fluorophores and Lipids
Source: Bioconjug Chem. 2021 Feb 5;32(2):350–66. doi: 10.1021/acs.bioconjchem.0c00717 (PMC9122261; doi:10.1021/acs.bioconjchem.0c00717)

Oligonucleotides Containing 1'-Aminomethyl or 1'-Mercaptomethyl-2'-deoxy-D-ribofuranoses: Synthesis, Purification, Characterization and Conjugation with Fluorophores and Lipids

Virginia Martín-Nieves,[†] Carme Fàbrega,[‡,§] Marc Guasch,[‡,§] Susana Fernández,[†] Yogesh S. Sanghvi,[#] Miguel Ferrero,[,†] and Ramón Eritja,[*,‡,§]*

[†]Departamento de Química Orgánica e Inorgánica, Universidad de Oviedo, 33006-Oviedo (Asturias), Spain.
[‡]Dpt. Chemical & Biomolecular Nanotechnology, Institute for Advanced Chemistry of Catalonia (IQAC), CSIC, 08034-Barcelona, Spain.
[§]CIBER-BBN Networking Centre on Bioengineering, Biomaterials and Nanomedicine, 08034-Barcelona, Spain.
[#]Rasayan Inc., 2802 Crystal Ridge Road, Encinitas, CA 92024-6615, USA.

ELECTRONIC SUPPLEMENTARY INFORMATION (page 1 of 181 pages)

Contents:

Copies of ^{1}H, ^{13}C, ^{31}P, DEPT, and some 2D NMR spectra of compounds **2α/2β**, **3α/3β**, **4α/4β**, **5α/5β**, **6α/6β**, **7α/7β**, **8α/8β**, **9β**, **10β**, **11β**, **12β**, **13α/13β**, **14α/14β**, **15α/15β**, and **16α/16β**:

- ^{1}H NMR of **2α** (S3)
- ^{13}C NMR of **2α** (S4)
- DEPT NMR of **2α** (S5)
- COSY of **2α** (S6)
- HSQC of **2α** (S7)
- HMBC of **2α** (S8)
- ^{1}H NMR of **2β** (S9)
- ^{13}C NMR of **2β** (S10)
- DEPT NMR of **2β** (S11)
- COSY of **2β** (S12)
- HSQC of **2β** (S13)
- HMBC of **2β** (S14)
- ^{1}H NMR of **3α** (S15)
- ^{13}C NMR of **3α** (S16)
- DEPT NMR of **3α** (S17)
- COSY of **3α** (S18)
- HSQC of **3α** (S19)
- HMBC of **3α** (S20)
- ^{1}H NMR of **3β** (S21)
- ^{13}C NMR of **3β** (S22)
- DEPT NMR of **3β** (S23)
- COSY of **3β** (S24)
- HSQC of **3β** (S25)
- HMBC of **3β** (S26)
- ^{1}H NMR of **4α** (S27)
- ^{13}C NMR of **4α** (S28)
- DEPT NMR of **4α** (S29)
- COSY of **4α** (S30)
- HSQC of **4α** (S31)
- HMBC of **4α** (S32)
- ^{1}H NMR of **4β** (S33)
- ^{13}C NMR of **4β** (S34)
- DEPT NMR of **4β** (S35)
- COSY of **4β** (S36)
- HSQC of **4β** (S37)
- HMBC of **4β** (S38)
- ^{1}H NMR of **5α-A** (S39)
- ^{13}C NMR of **5α-A** (S40)
- DEPT NMR of **5α-A** (S41)
- COSY of **5α-A** (S42)
- HSQC of **5α-A** (S43)
- HMBC of **5α-A** (S44)
- ^{31}P NMR **5α-A** (S45)
- ^{1}H NMR of **5α-B** (S46)
- ^{13}C NMR of **5α-B** (S47)
- DEPT NMR of **5α-B** (S48)
- COSY of **5α-B** (S49)
- HSQC of **5α-B** (S50)
- HMBC of **5α-B** (S51)
- ^{31}P NMR **5α-B** (S52)
- ^{1}H NMR of **5β-A** (S53)
- ^{13}C NMR of **5β-A** (S54)
- DEPT NMR of **5β-A** (S55)
- COSY of **5β-A** (S56)
- HSQC of **5β-A** (S57)
- HMBC of **5β-A** (S58)
- ^{31}P NMR **5β-A** (S59)
- ^{1}H NMR of **5β-B** (S60)
- ^{13}C NMR of **5β-B** (S61)
- DEPT NMR of **5β-B** (S62)

- COSY of **5β-B** (S63)
- HSQC of **5β-B** (S64)
- HMBC of **5β-B** (S65)
- ^{31}P NMR **5β-B** (S66)
- ^{1}H NMR of **6α** (S67)
- ^{13}C NMR of **6α** (S68)
- DEPT NMR of **6α** (S69)
- COSY of **6α** (S70)
- HSQC of **6α** (S71)
- HMBC of **6α** (S72)
- ^{1}H NMR of **6β** (S73)
- ^{13}C NMR of **6β** (S74)
- DEPT NMR of **6β** (S75)
- COSY of **6β** (S76)
- HSQC of **6β** (S77)
- HMBC of **6β** (S78)
- ^{1}H NMR of **7α** (S79)
- ^{13}C NMR of **7α** (S80)
- DEPT NMR of **7α** (S81)
- COSY of **7α** (S82)
- HSQC of **7α** (S83)
- HMBC of **7α** (S84)
- ^{1}H NMR of **7β** (S85)
- ^{13}C NMR of **7β** (S86)
- DEPT NMR of **7β** (S87)
- COSY of **7β** (S88)
- HSQC of **7β** (S89)
- HMBC of **7β** (S90)
- ^{1}H NMR of **8α-A** (S91)
- ^{13}C NMR of **8α-A** (S92)
- DEPT NMR of **8α-A** (S93)
- COSY of **8α-A** (S94)
- HSQC of **8α-A** (S95)
- ^{31}P NMR **8α-A** (S96)
- ^{1}H NMR of **8α-B** (S97)
- ^{13}C NMR of **8α-B** (S98)
- DEPT NMR of **8α-B** (S99)
- COSY of **8α-B** (S100)
- HSQC of **8α-B** (S101)
- ^{31}P NMR **8α-B** (S102)
- ^{1}H NMR of **8β-A** (S103)
- ^{13}C NMR of **8β-A** (S104)
- DEPT NMR of **8β-A** (S105)
- COSY of **8β-A** (S106)
- HSQC of **8β-A** (S107)
- ^{31}P NMR **8β-A** (S108)
- ^{1}H NMR of **8β-B** (S109)
- ^{13}C NMR of **8β-B** (S110)
- DEPT NMR of **8β-B** (S111)
- COSY of **8β-B** (S112)
- HSQC of **8β-B** (S113)
- ^{31}P NMR **8β-B** (S114)
- ^{1}H NMR of **9β** (S115)
- ^{13}C NMR of **9β** (S116)
- DEPT NMR of **9β** (S117)
- COSY of **9β** (S118)
- HSQC of **9β** (S119)
- ^{1}H NMR of **10β** (S120)
- ^{13}C NMR of **10β** (S121)
- DEPT NMR of **10β** (S122)
- COSY of **10β** (S123)
- HSQC of **10β** (S124)
- ^{1}H NMR of **11β** (S125)
- ^{13}C NMR of **11β** (S126)
- ^{1}H NMR of **12β** (S127)
- ^{13}C NMR of **12β** (S128)
- ^{1}H NMR of **13α** (S129)
- ^{13}C NMR of **13α** (S130)
- DEPT NMR of **13α** (S131)
- COSY of **13α** (S132)
- HSQC of **13α** (S133)
- HMBC of **13α** (S134)
- ^{1}H NMR of **13β** (S135)
- ^{13}C NMR of **13β** (S136)
- DEPT NMR of **13β** (S137)
- COSY of **13β** (S138)
- HSQC of **13β** (S139)
- HMBC of **13β** (S140)
- ^{1}H NMR of **14α** (S141)
- ^{13}C NMR of **14α** (S142)
- DEPT NMR of **14α** (S143)
- COSY of **14α** (S144)
- HSQC of **14α** (S145)
- HMBC of **14α** (S146)
- ^{1}H NMR of **14β** (S147)
- ^{13}C NMR of **14β** (S148)
- DEPT NMR of **14β** (S149)
- COSY of **14β** (S150)
- HSQC of **14β** (S151)
- HMBC of **14β** (S152)
- ^{1}H NMR of **15α** (S153)
- ^{13}C NMR of **15α** (S154)
- DEPT NMR of **15α** (S155)
- COSY of **15α** (S156)
- HSQC of **15α** (S157)
- HMBC of **15α** (S158)
- ^{1}H NMR of **15β** (S159)
- ^{13}C NMR of **15β** (S160)
- DEPT NMR of **15β** (S161)
- COSY of **15β** (S162)
- HSQC of **15β** (S163)
- HMBC of **15β** (S164)
- ^{1}H NMR of **16α-A** (S165)
- ^{31}P NMR **16α-A** (S166)
- ^{1}H NMR of **16α-A**+**B** (S167)
- ^{31}P NMR **16α-A+B** (S168)
- ^{1}H NMR of **16β-A** (S169)
- ^{13}C NMR of **16β-A** (S170)
- DEPT NMR of **16β-A** (S171)
- COSY of **16β-A** (S172)
- HSQC of **16β-A** (S173)
- HMBC of **16β-A** (S174)
- ^{31}P NMR **16β-A** (S175)
- ^{1}H NMR of **16β-B** (S176)
- ^{13}C NMR of **16β-B** (S177)
- COSY of **16β-B** (S178)
- HSQC of **16β-B** (S179)
- HMBC of **16β-B** (S180)
- ^{31}P NMR **16β-B** (S181)

1α-Aminomethyl-1,2-dideoxy-D-*erythro*-pentofuranose (2α)

^{1}H NMR (300.13 MHz, MeOH-d_4)

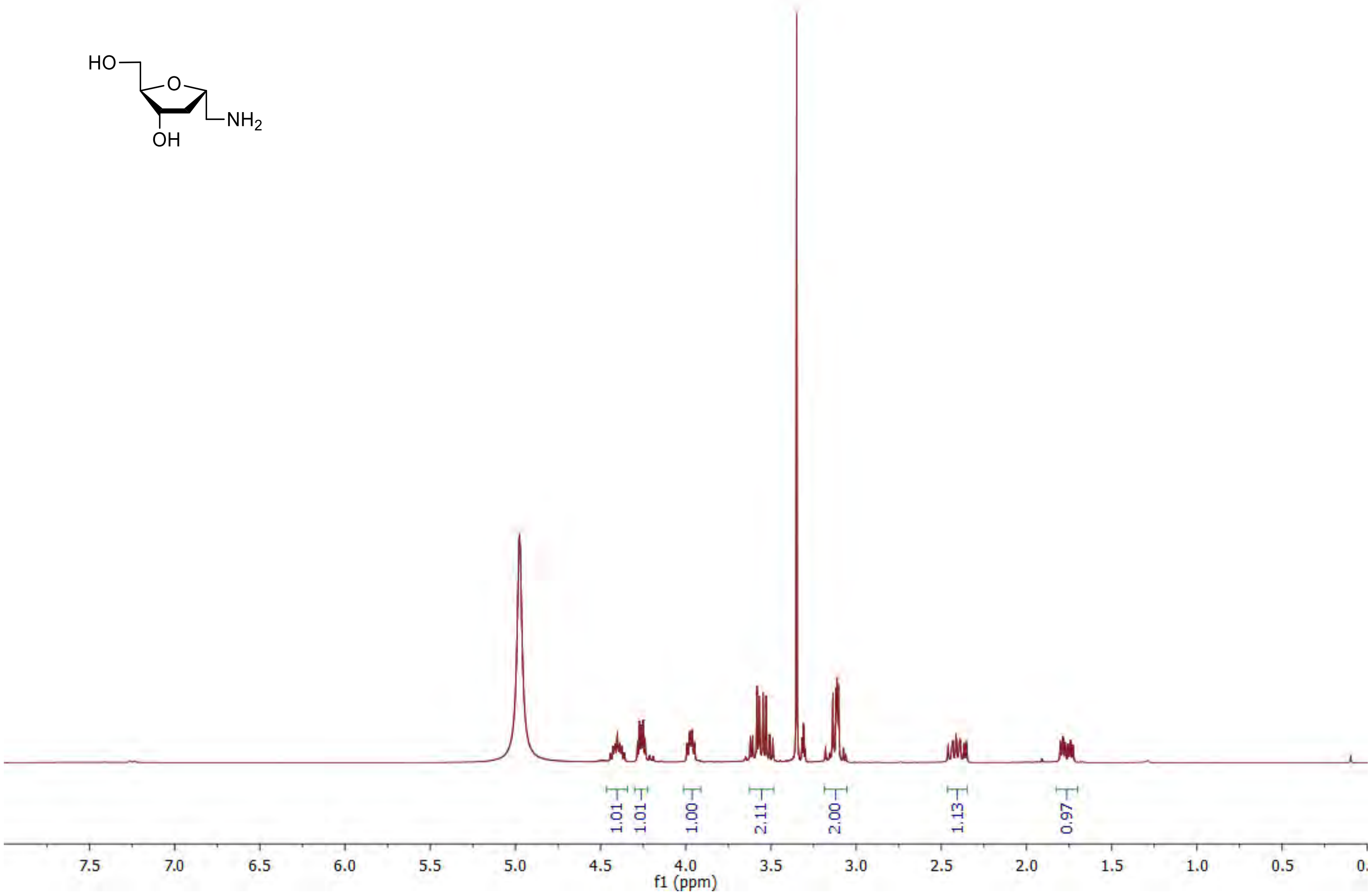

1α-Aminomethyl-1,2-dideoxy-D-*erythro*-pentofuranose (2α)

^{13}C NMR (75.5 MHz, MeOH-d_4)

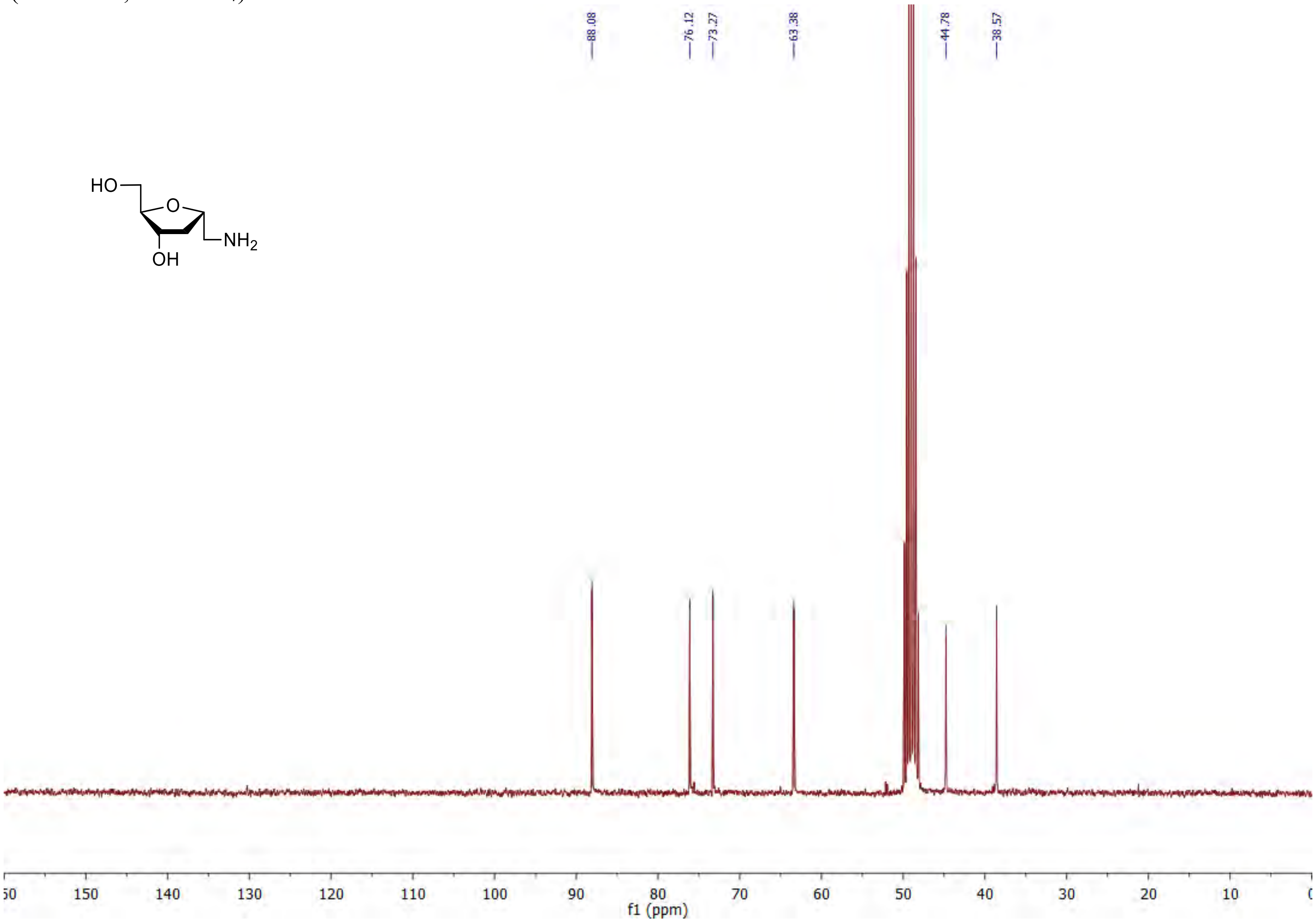

1α-Aminomethyl-1,2-dideoxy-D-*erythro*-pentofuranose (2α)

DEPT135 NMR (75.5 MHz, MeOH-d_4)

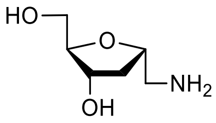

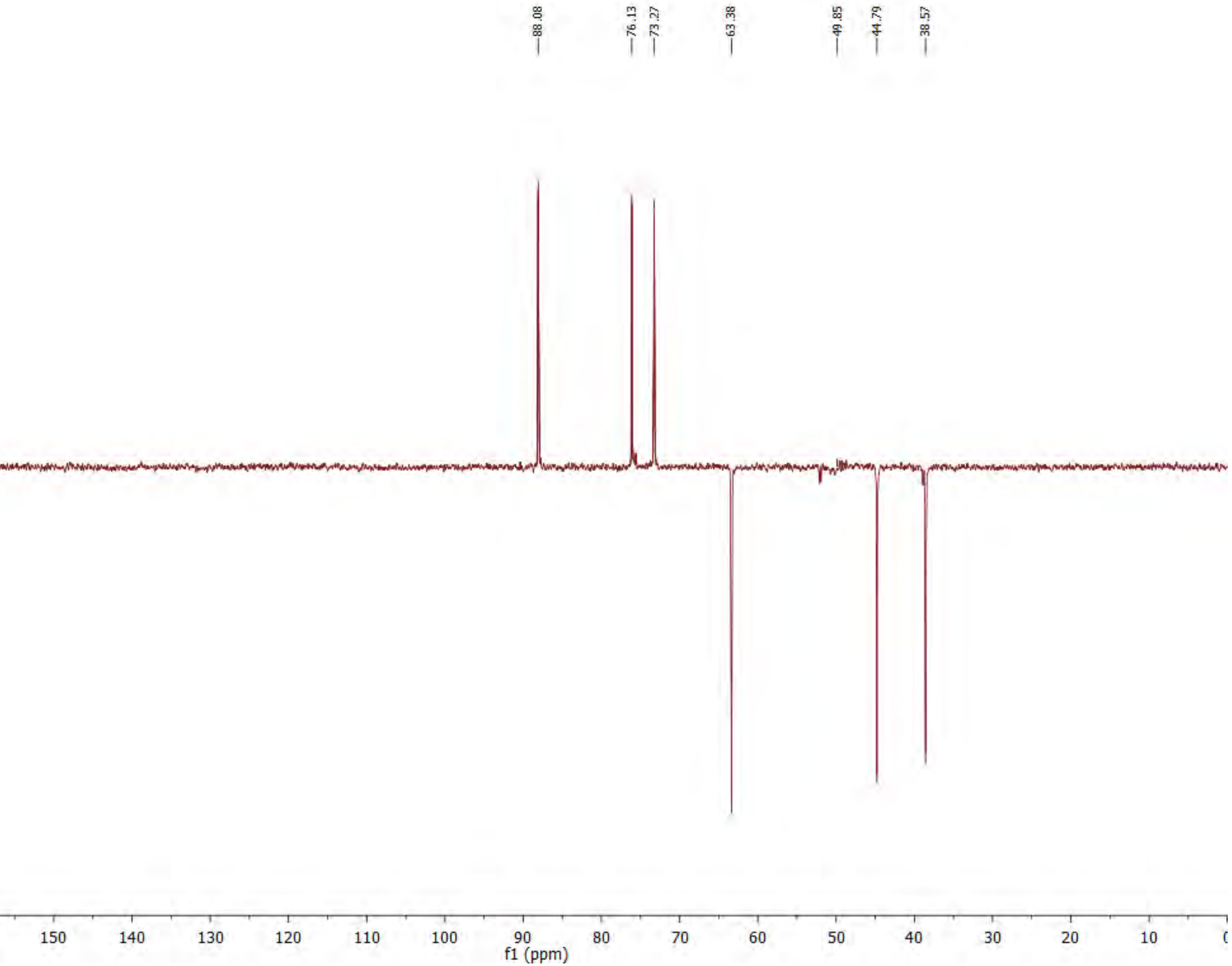

1α-Aminomethyl-1,2-dideoxy-D-*erythro*-pentofuranose (2α)

COSY NMR (MeOH-d_4)

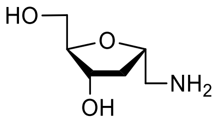

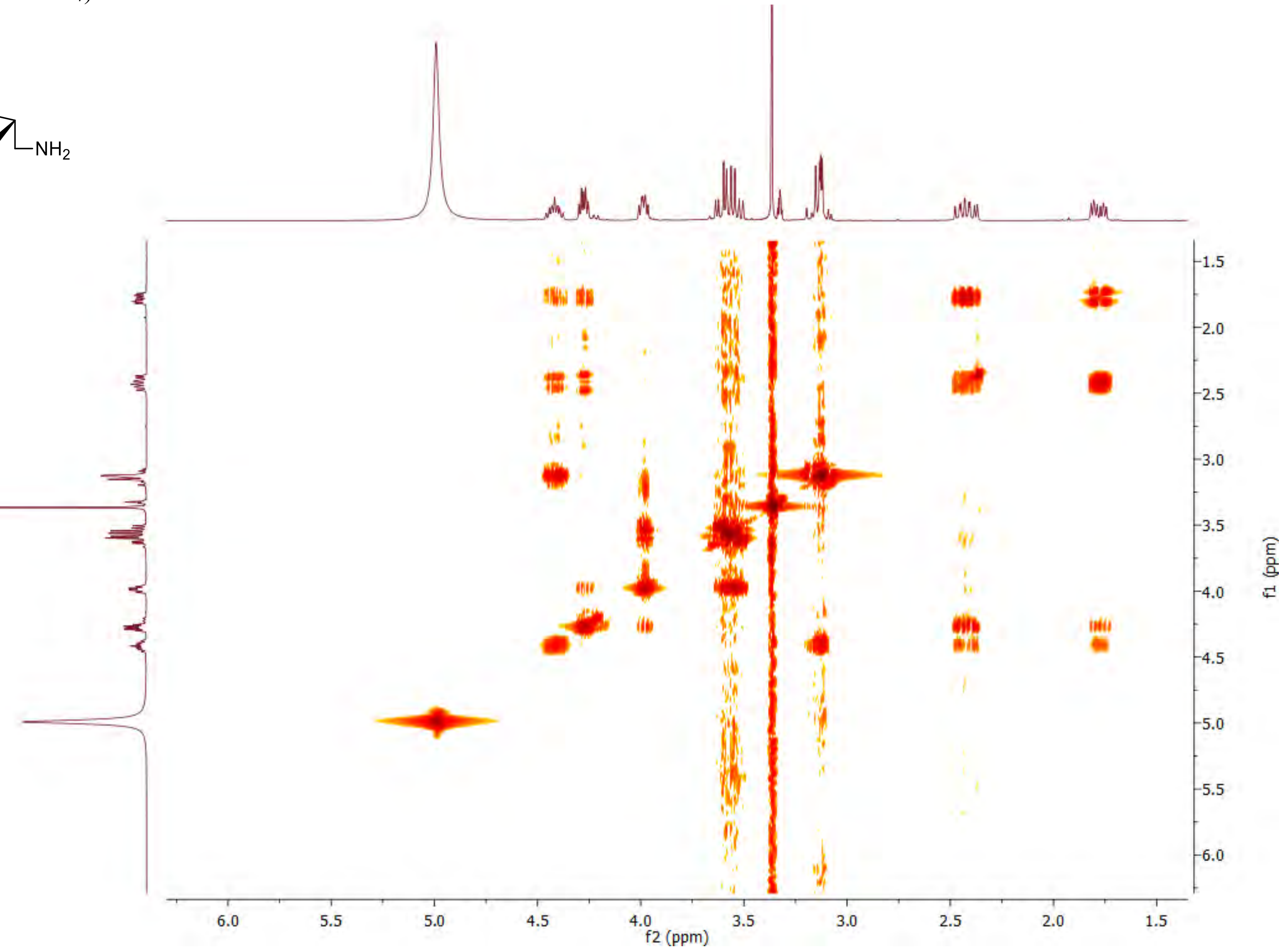

HSQC NMR (MeOH-d_4)

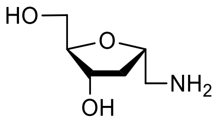

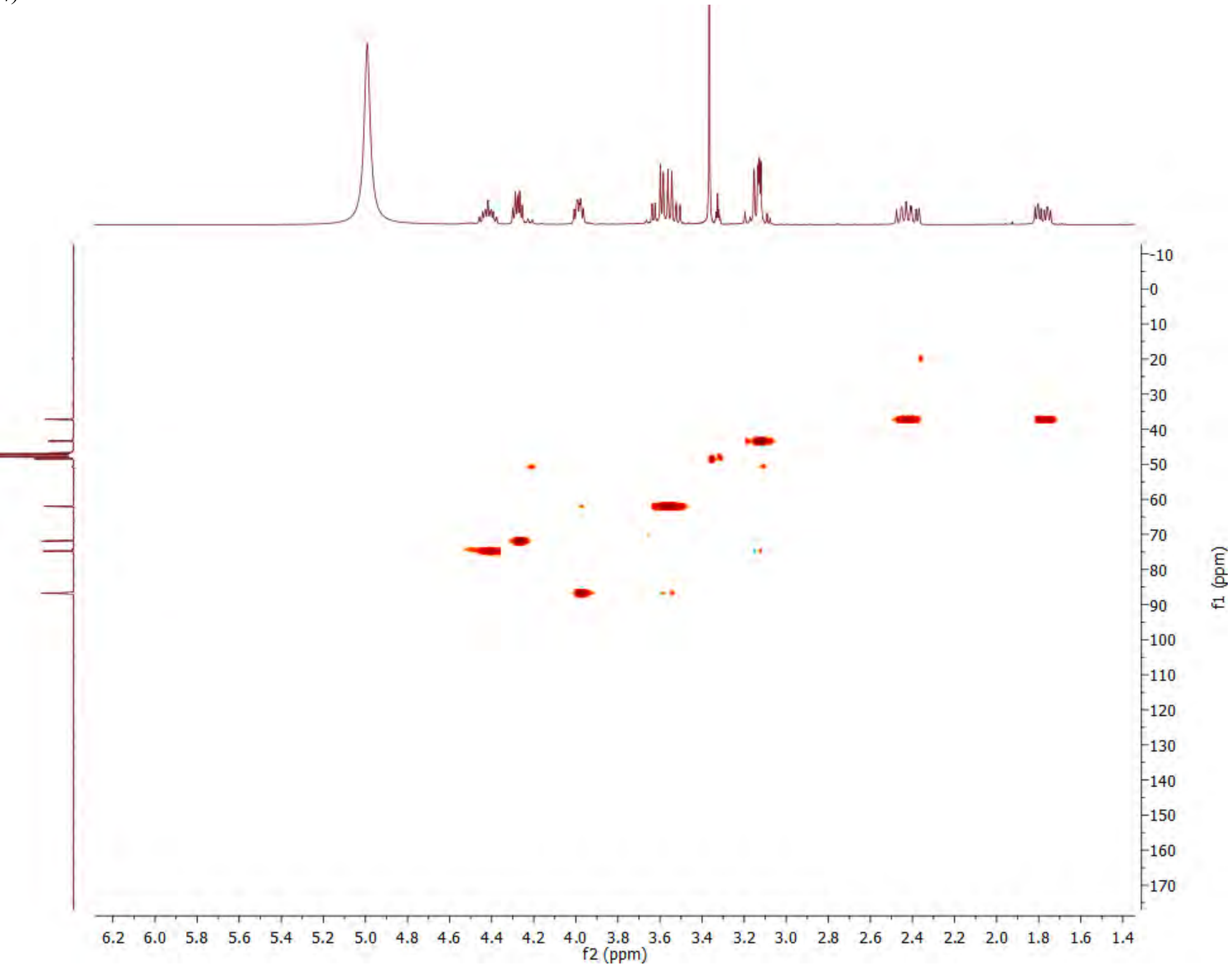

1α-Aminomethyl-1,2-dideoxy-D-*erythro*-pentofuranose (2α)

HMBC NMR (MeOH-d_4)

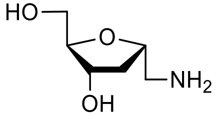

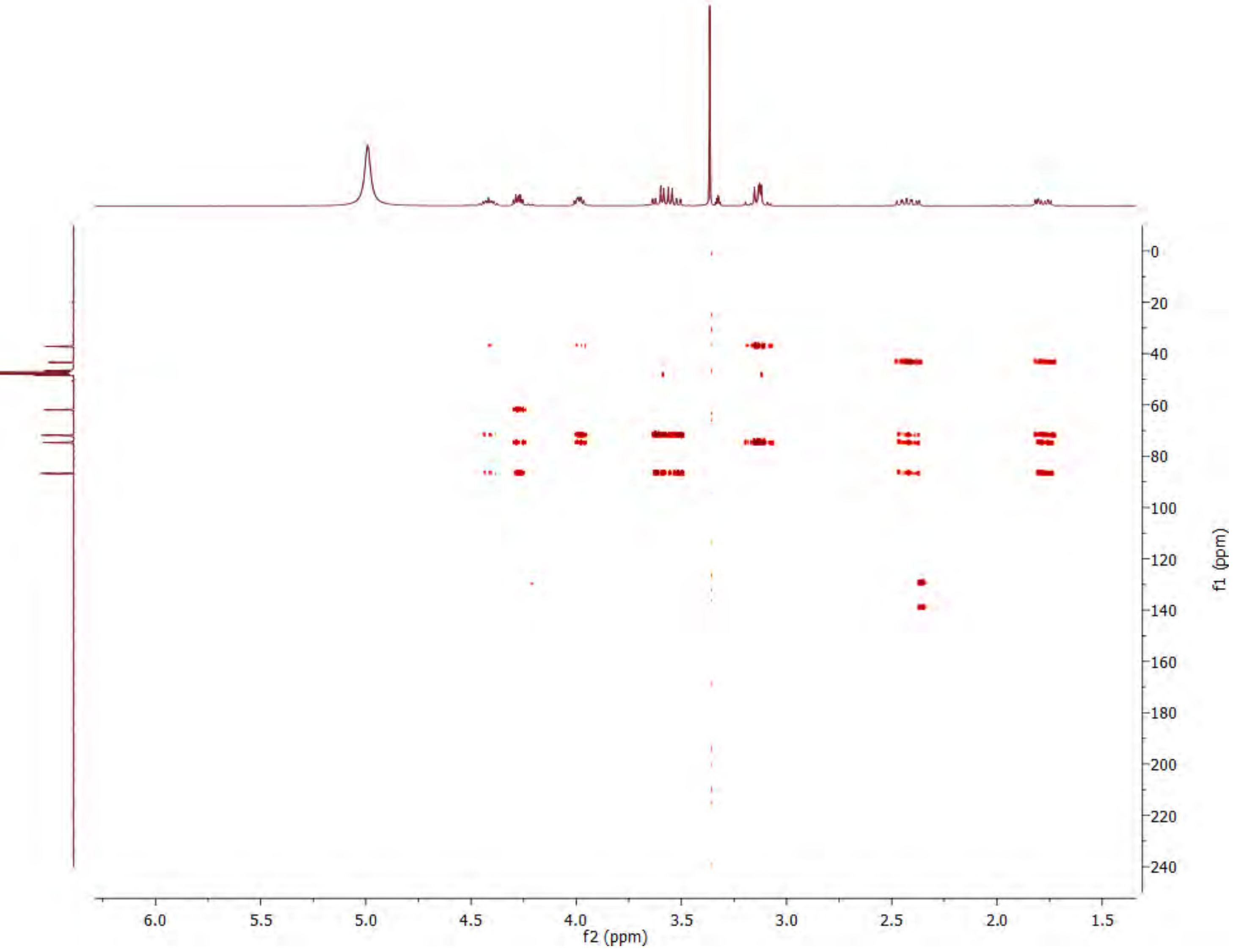

1β-Aminomethyl-1,2-dideoxy-D-*erythro*-pentofuranose (2β)

^{1}H NMR (300.13 MHz, MeOH-d_4)

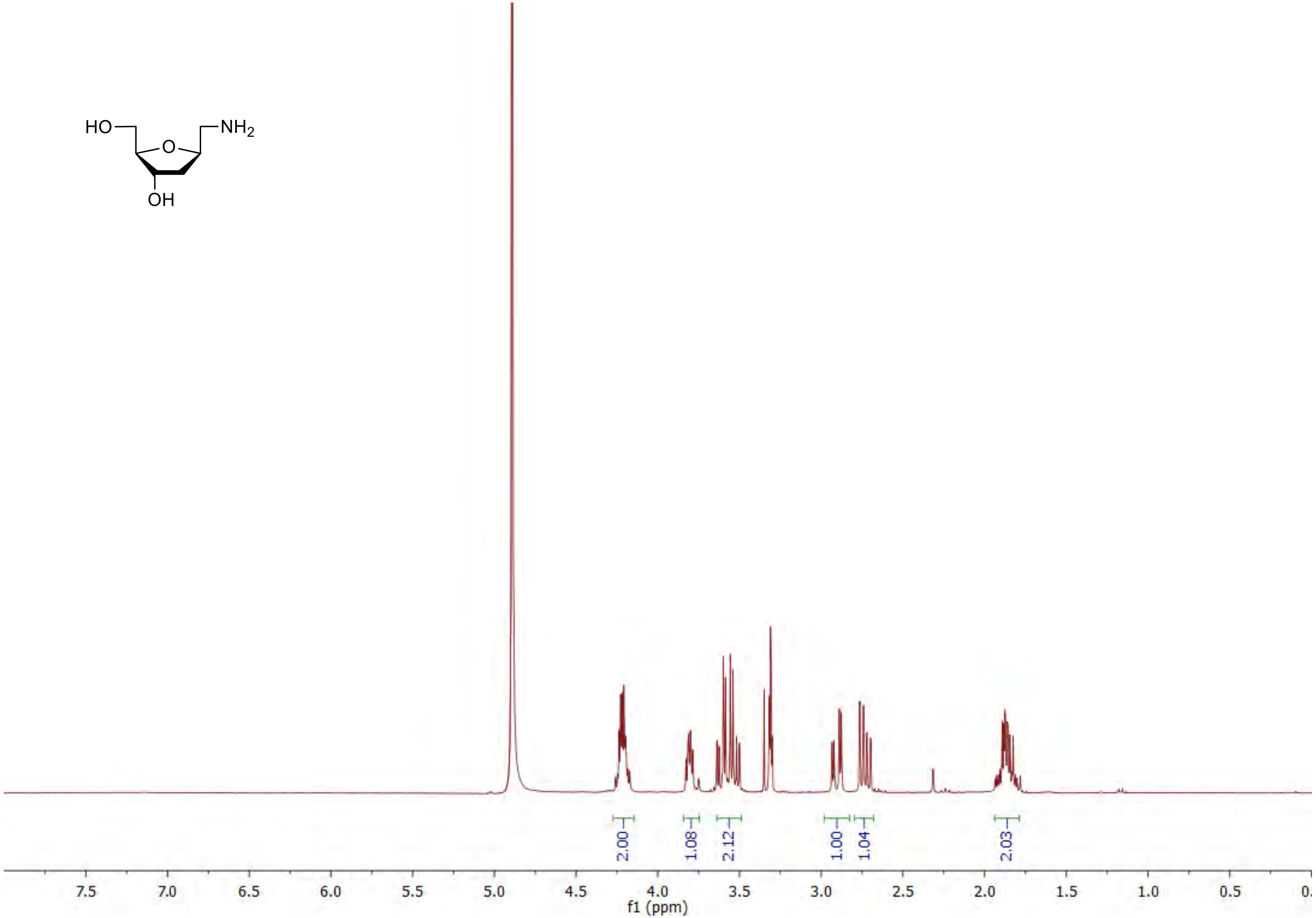

1β-Aminomethyl-1,2-dideoxy-D-*erythro*-pentofuranose (2β)

^{13}C NMR (75.5 MHz, MeOH-d_4)

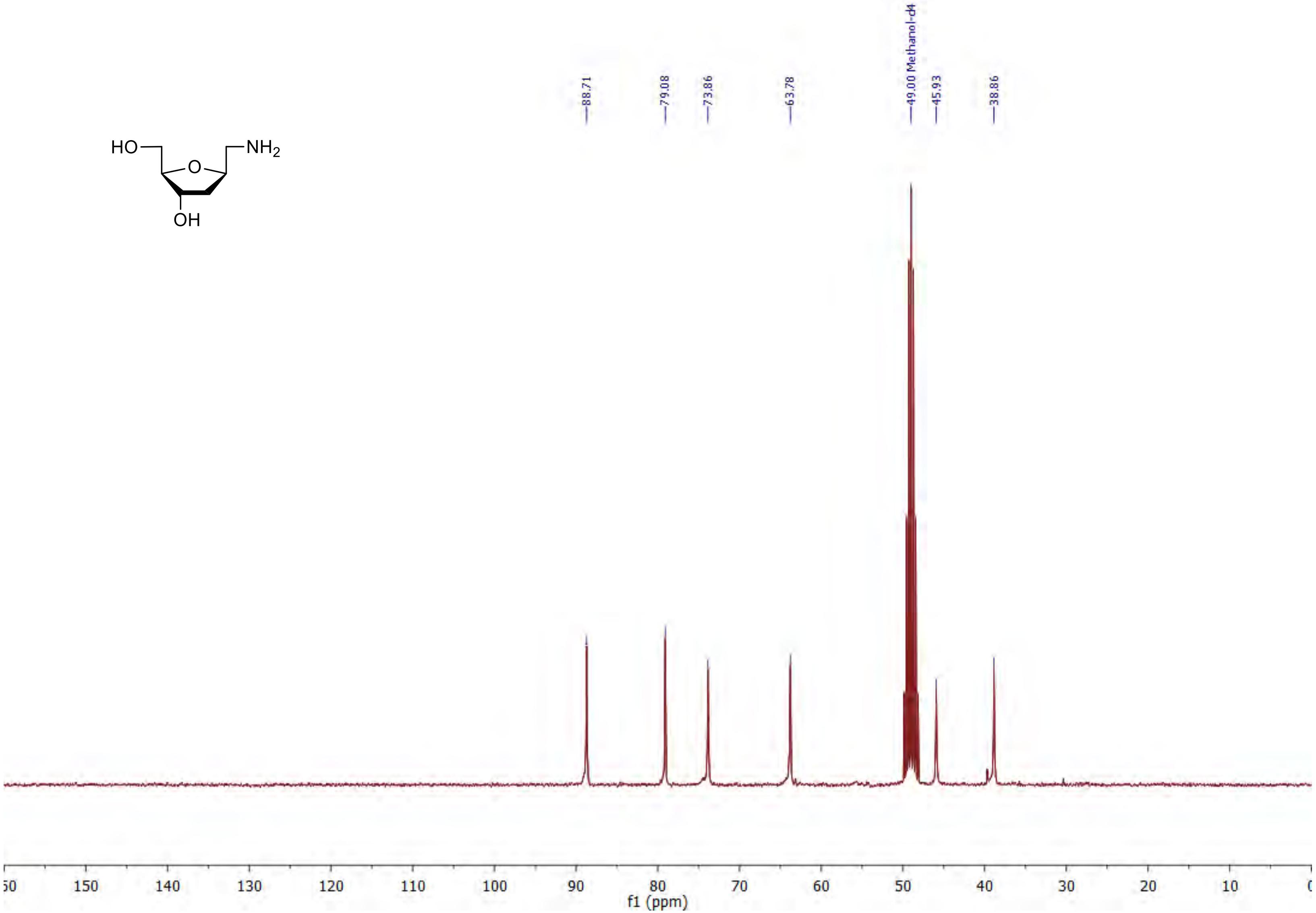

1β-Aminomethyl-1,2-dideoxy-D-*erythro*-pentofuranose (2β)

DEPT 135 NMR (75.5 MHz, MeOH-d_4)

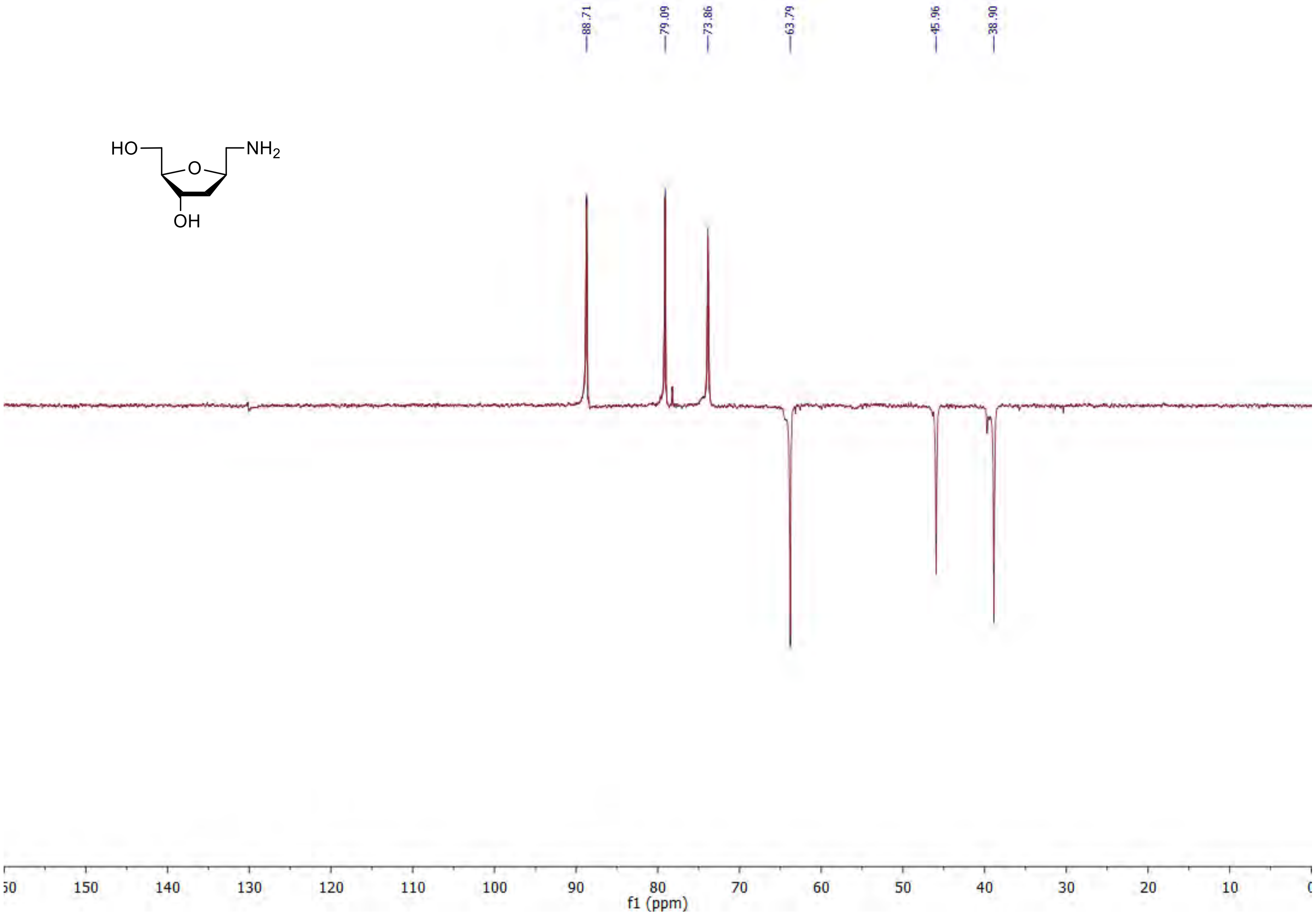

1β-Aminomethyl-1,2-dideoxy-D-*erythro*-pentofuranose (2β)

COSY NMR (MeOH-d_4)

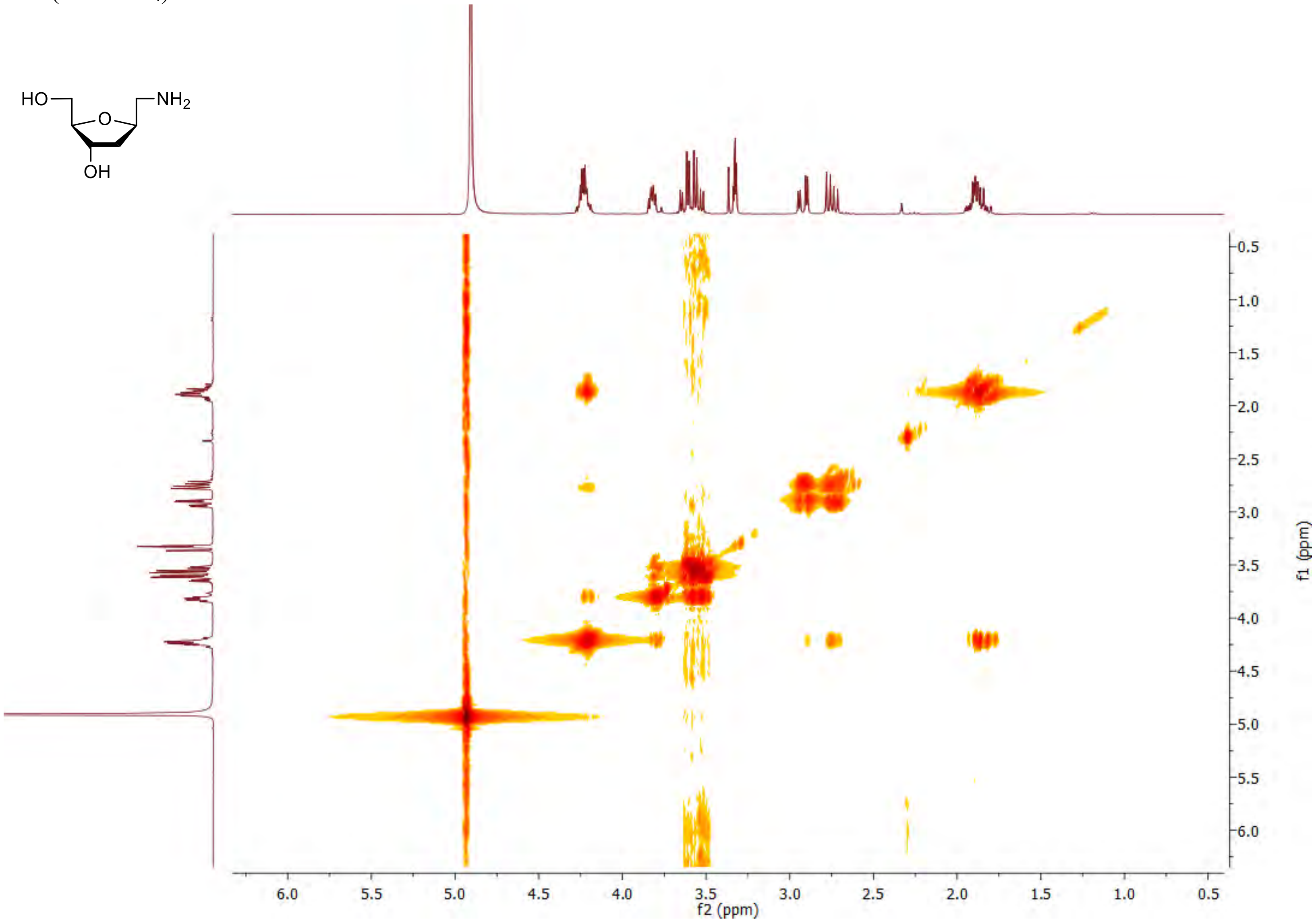

1β-Aminomethyl-1,2-dideoxy-D-*erythro*-pentofuranose (2β)

HSQC NMR (MeOH-d_4)

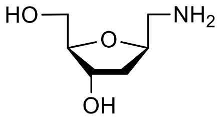

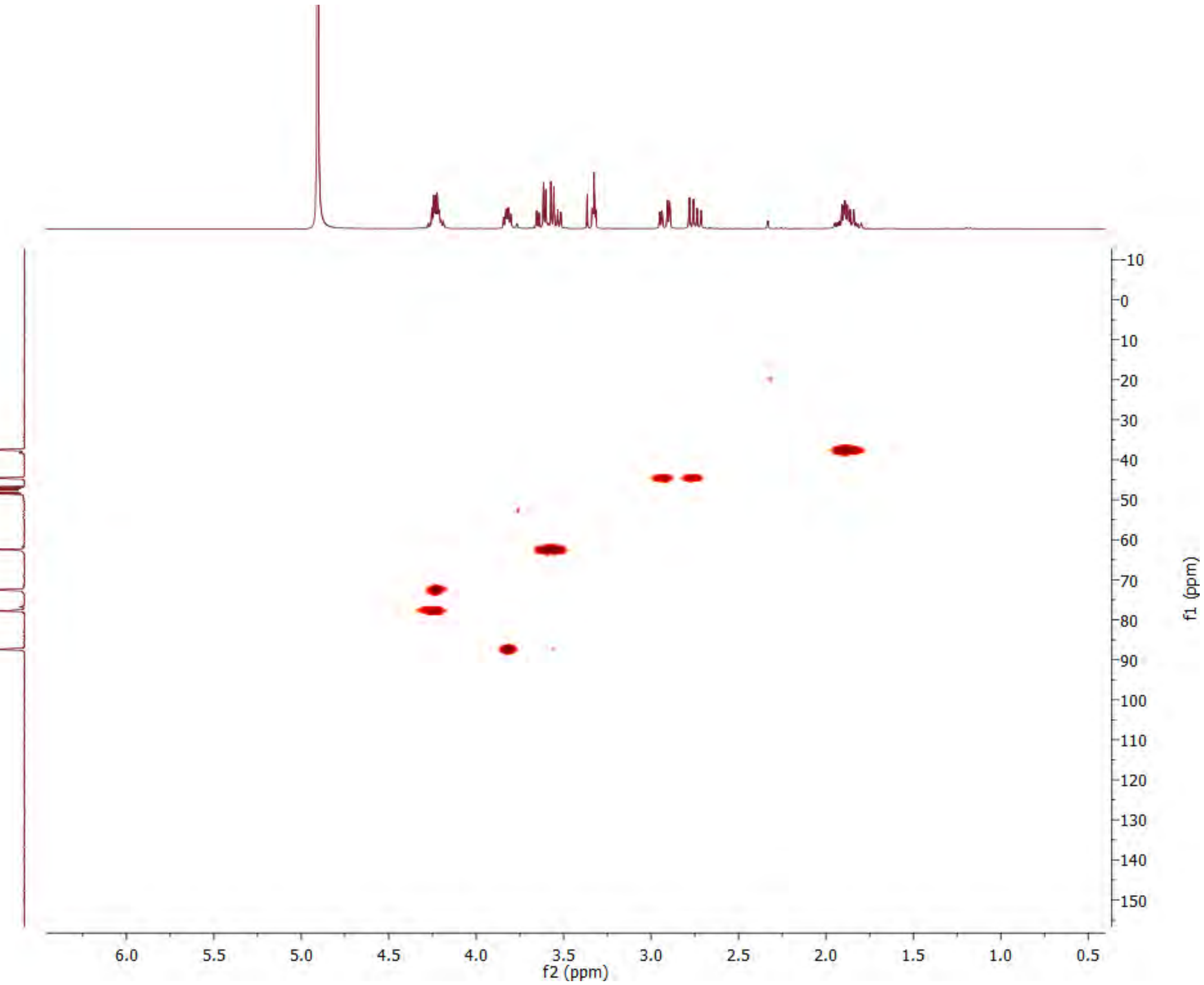

1β-Aminomethyl-1,2-dideoxy-ᴅ-*erythro*-pentofuranose (2β)

HMBC NMR (MeOH-d_4)

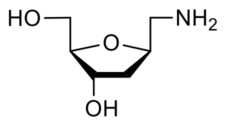

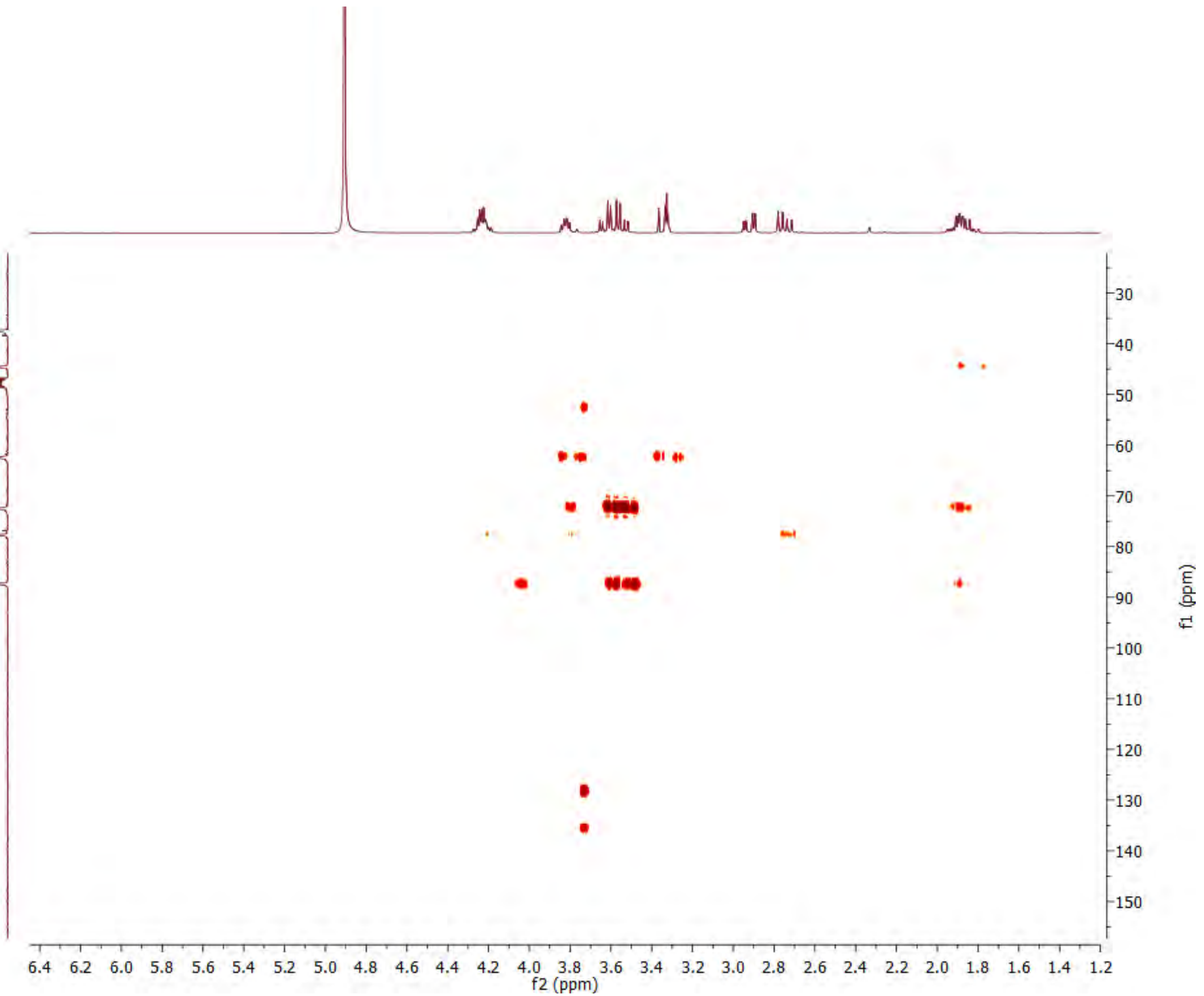

1,2-Dideoxy-1α-[*N*-(trifluoroacetyl)aminomethyl]-D-*erythro*-pentofuranose (3α)

^{1}H NMR (300.13 MHz, MeOH-d_4)

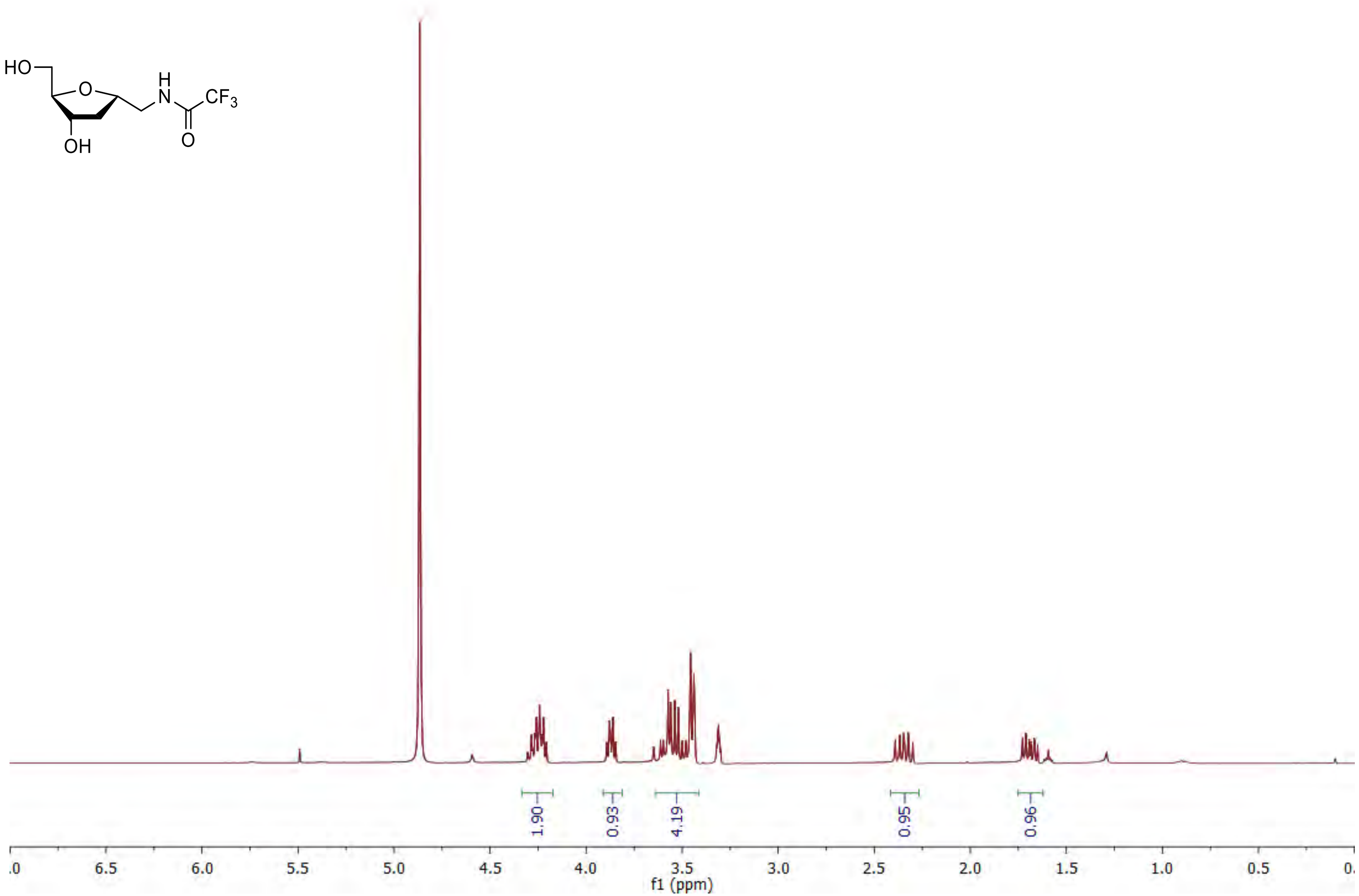

1,2-Dideoxy-1α-[*N*-(trifluoroacetyl)aminomethyl]-D-*erythro*-pentofuranose (3α)

^{13}C NMR (75.5 MHz, MeOH-d_4)

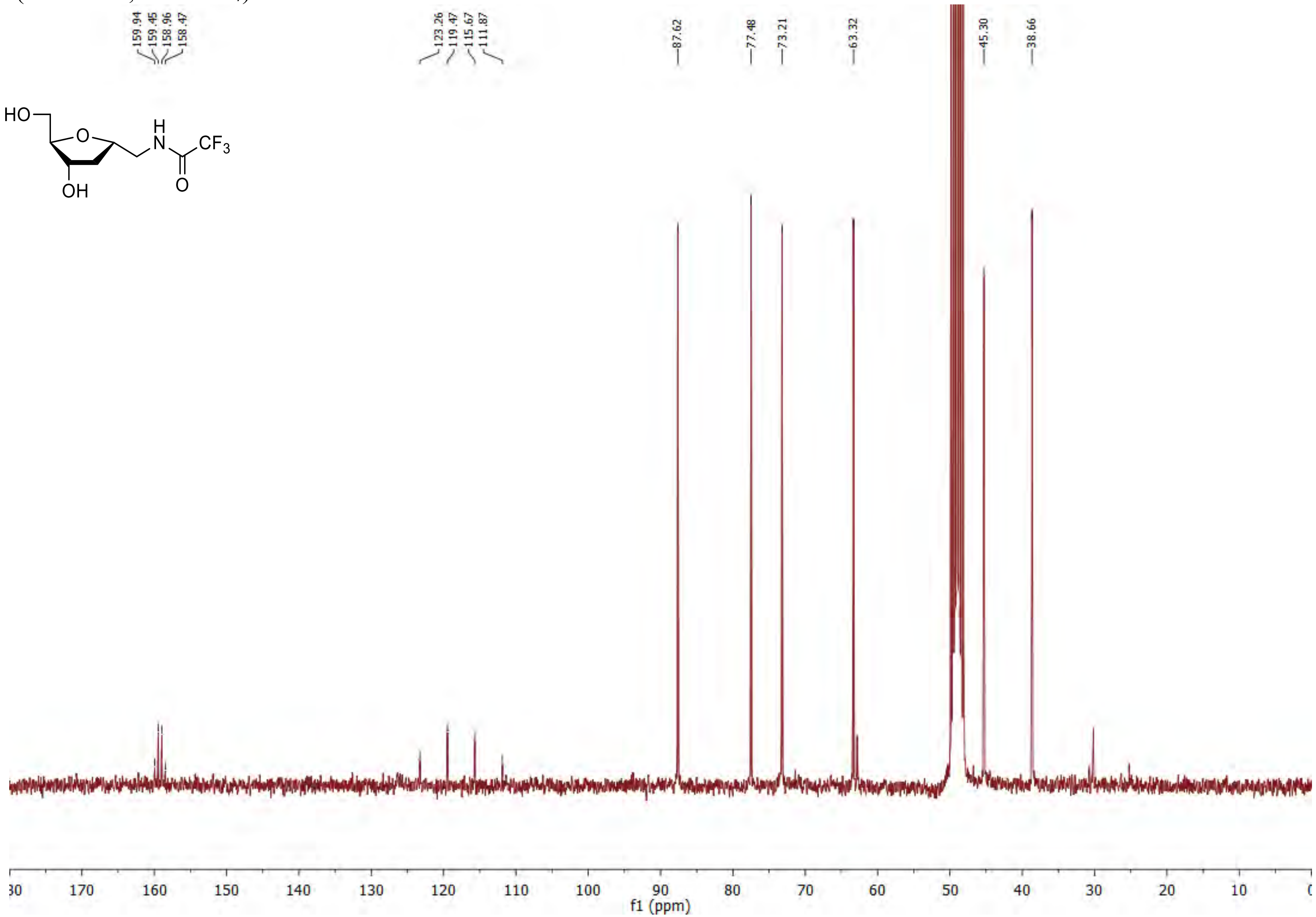

1,2-Dideoxy-1α-[*N*-(trifluoroacetyl)aminomethyl]-D-*erythro*-pentofuranose (3α)

DEPT135 NMR (75.5 MHz, MeOH-d_4)

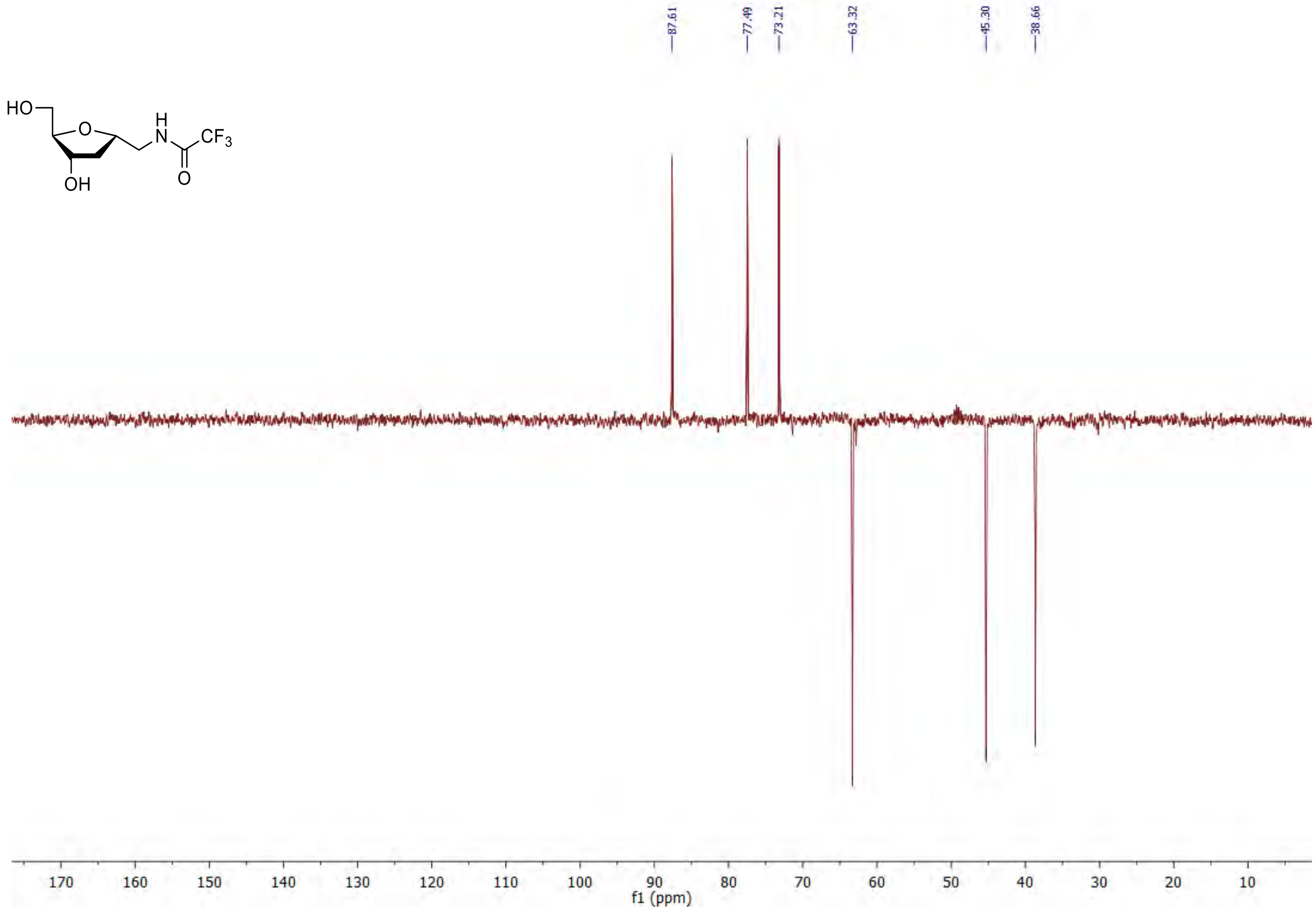

1,2-Dideoxy-1α-[*N*-(trifluoroacetyl)aminomethyl]-D-*erythro*-pentofuranose (3α)

COSY NMR (75.5 MHz, MeOH-d_4)

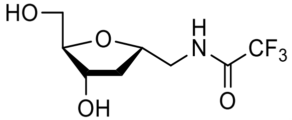

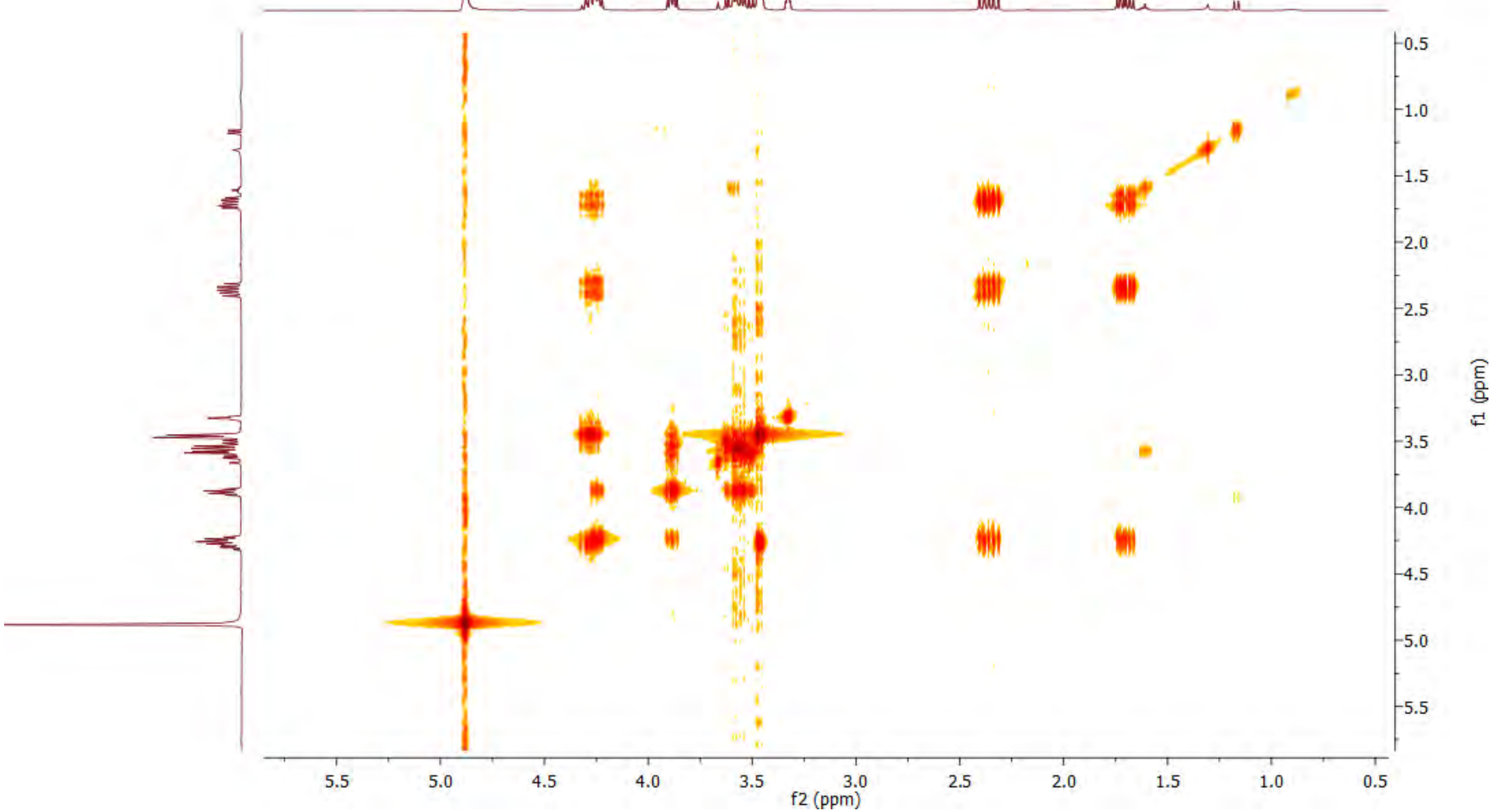

1,2-Dideoxy-1α-[*N*-(trifluoroacetyl)aminomethyl]-D-*erythro*-pentofuranose (3α)

HSQC NMR (75.5 MHz, MeOH-d_4)

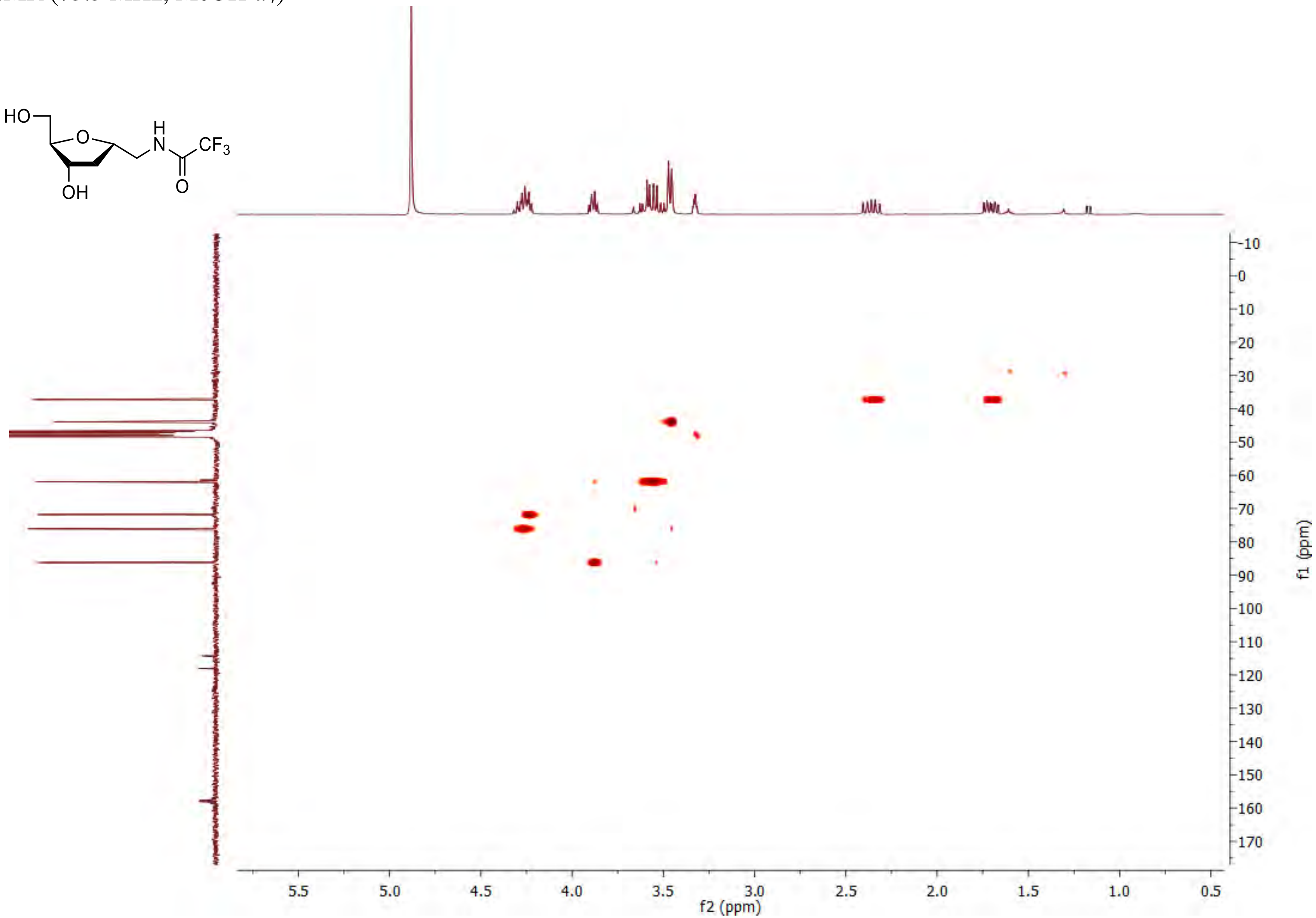

1,2-Dideoxy-1α-[*N*-(trifluoroacetyl)aminomethyl]-D-*erythro*-pentofuranose (3α)

HMBC NMR (75.5 MHz, MeOH-d_4)

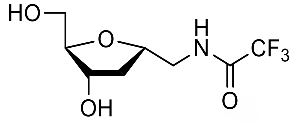

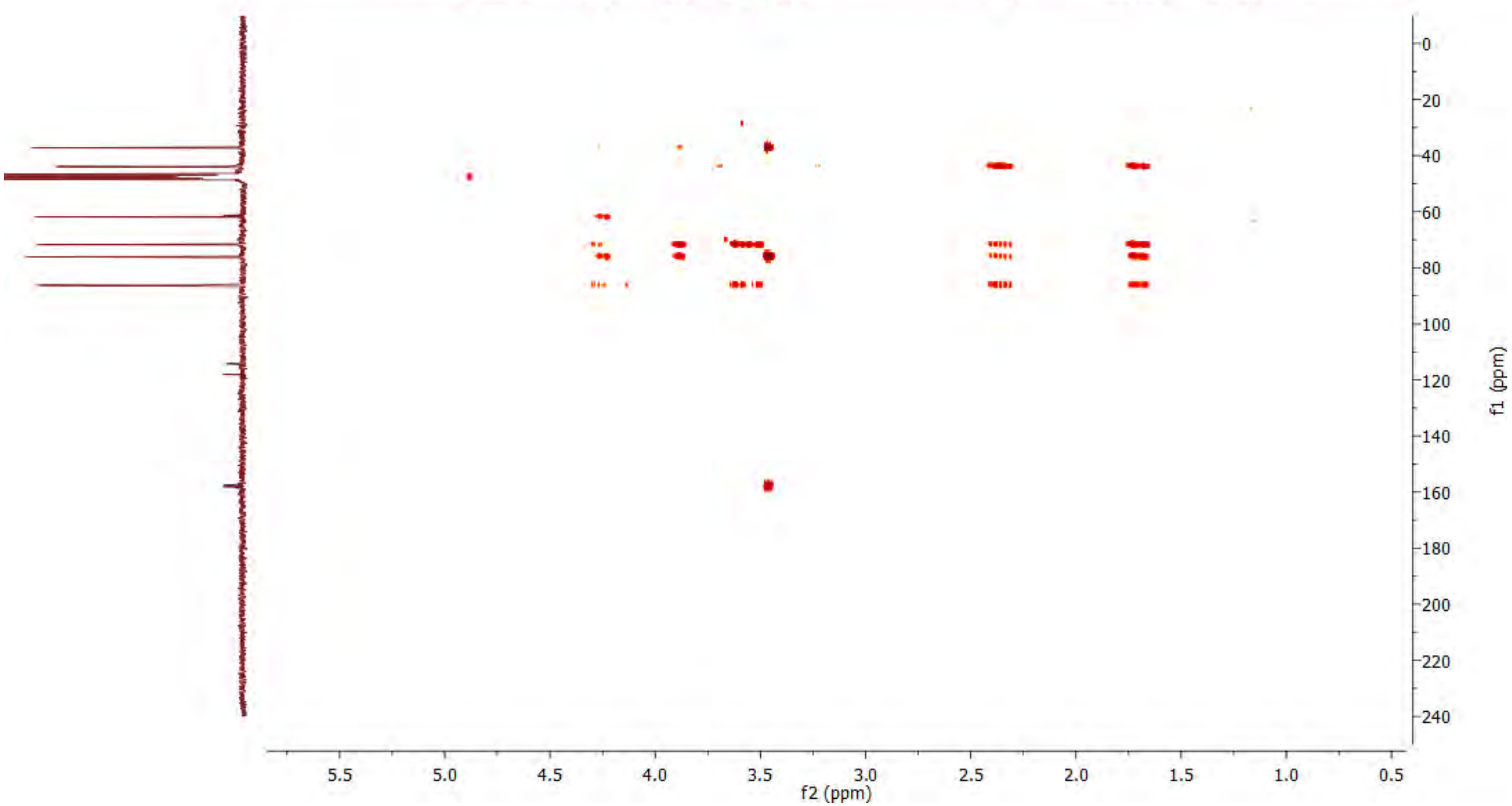

1,2-Dideoxy-1β-[*N*-(trifluoroacetyl)aminomethyl]-D-*erythro*-pentofuranose (3β)

^{1}H NMR (300.13 MHz, MeOH-d_4)

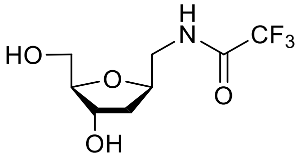

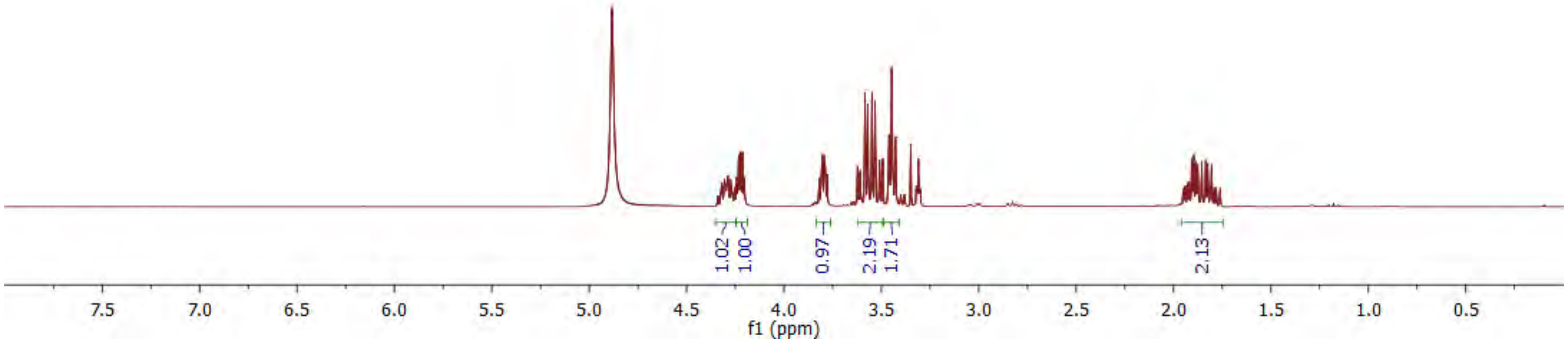

1,2-Dideoxy-1β-[*N*-(trifluoroacetyl)aminomethyl]-D-*erythro*-pentofuranose (3β)

^{13}C NMR (75.5 MHz, MeOH-d_4)

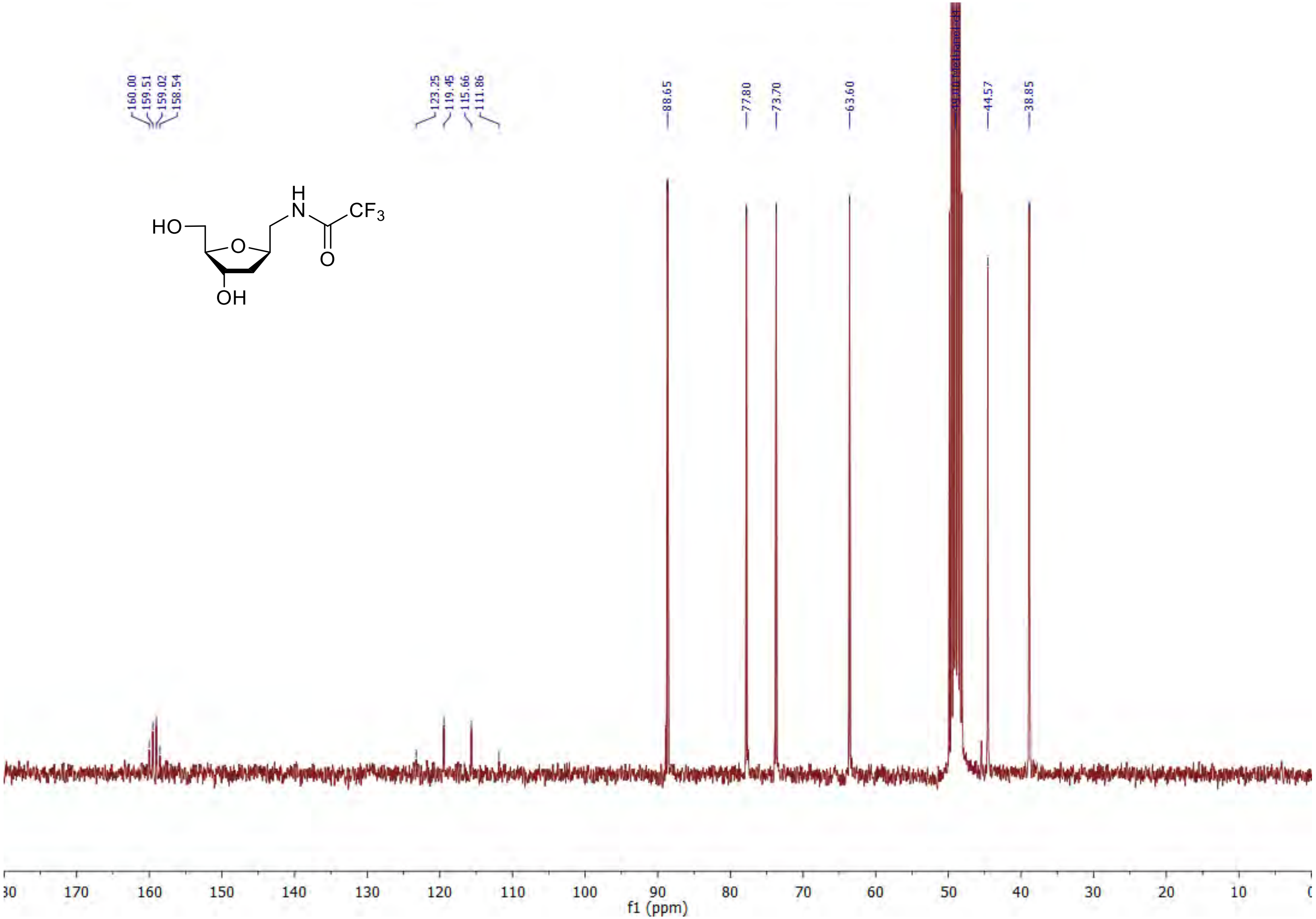

1,2-Dideoxy-1β-[*N*-(trifluoroacetyl)aminomethyl]-D-*erythro*-pentofuranose (3β)

DEPT 135 NMR (75.5 MHz, MeOH-d_4)

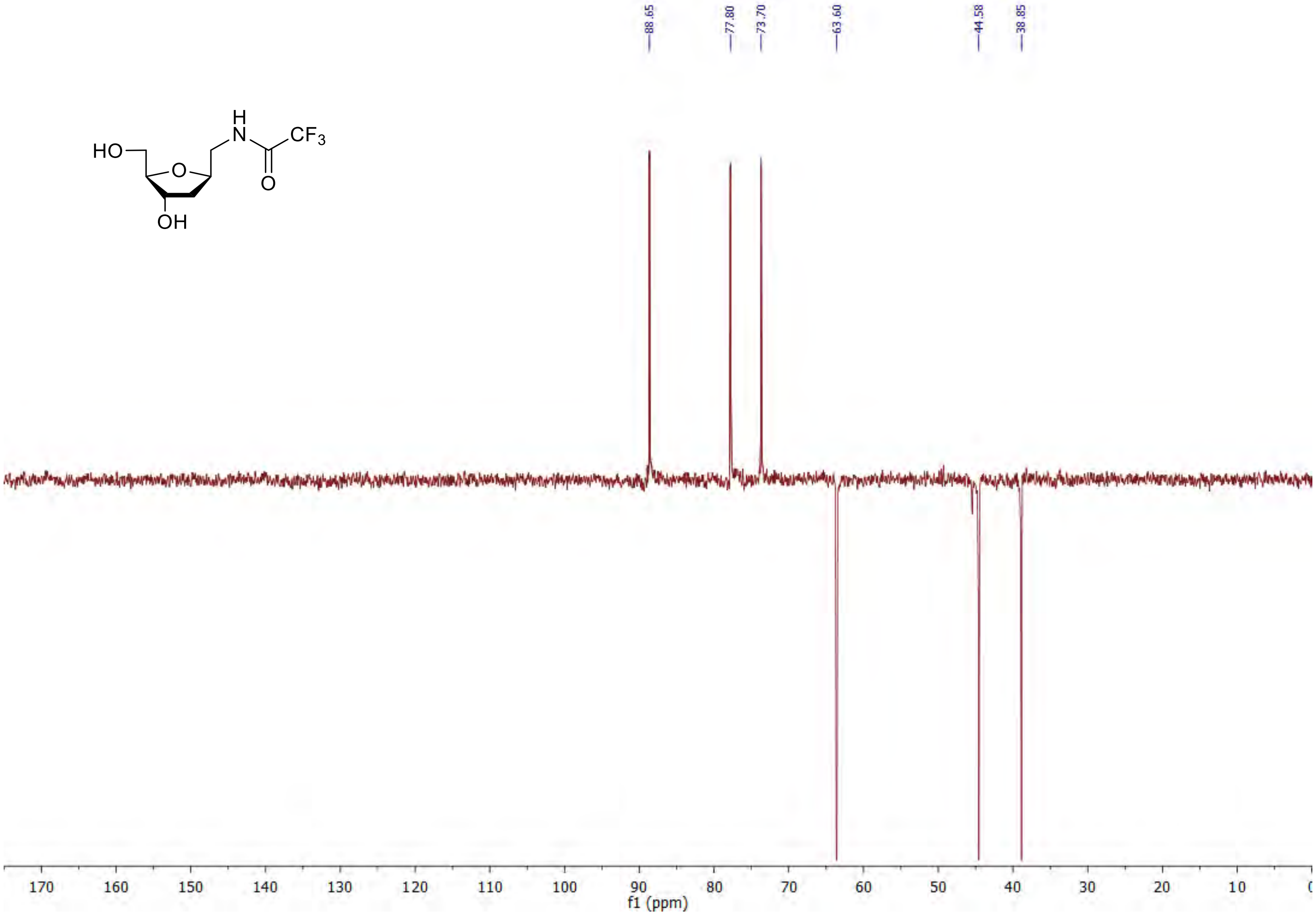

1,2-Dideoxy-1β-[*N*-(trifluoroacetyl)aminomethyl]-D-*erythro*-pentofuranose (3β)

COSY NMR (MeOH-d_4)

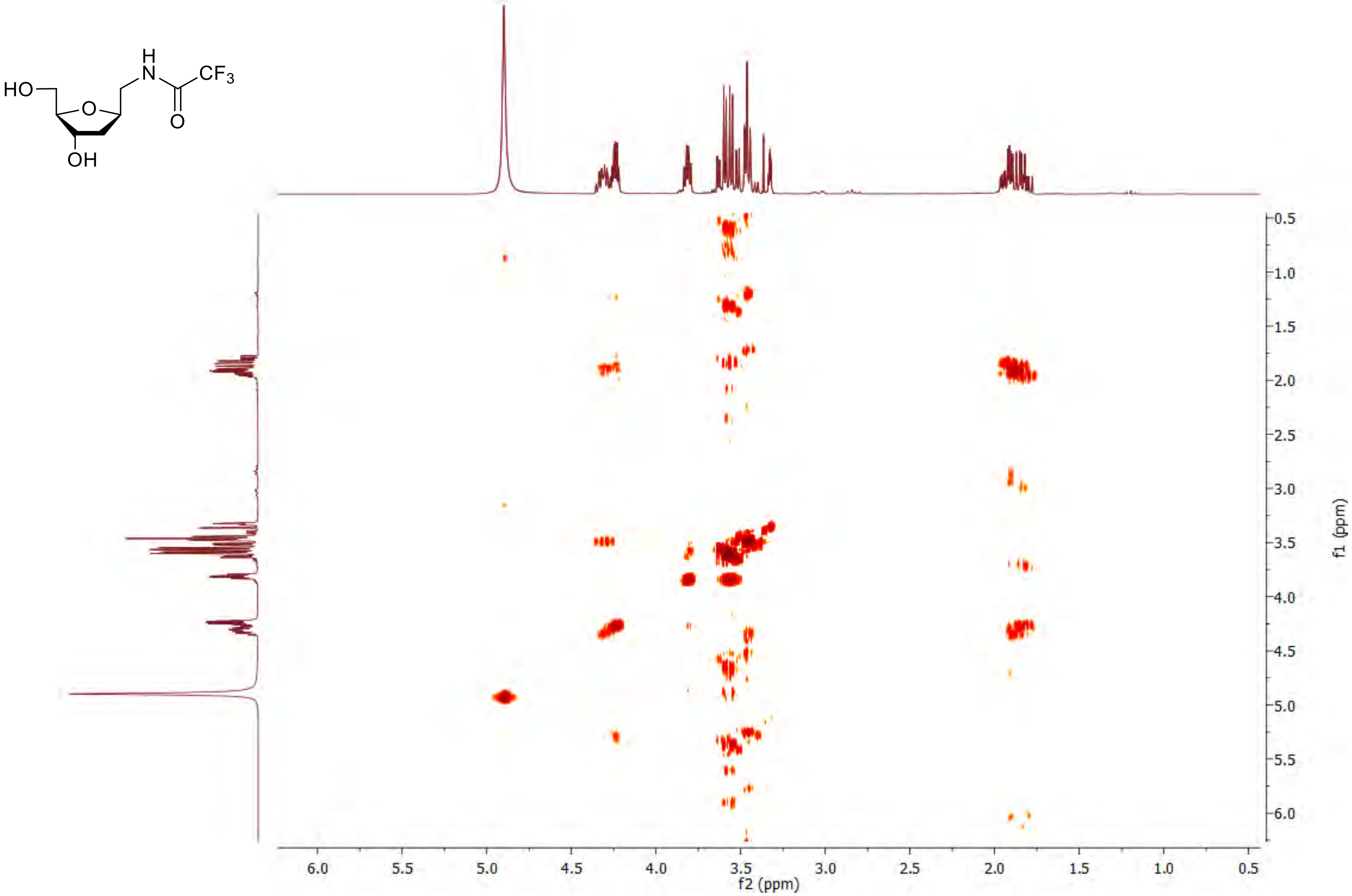

1,2-Dideoxy-1β-[*N*-(trifluoroacetyl)aminomethyl]-D-*erythro*-pentofuranose (3β)

HSQC NMR (MeOH-d_4)

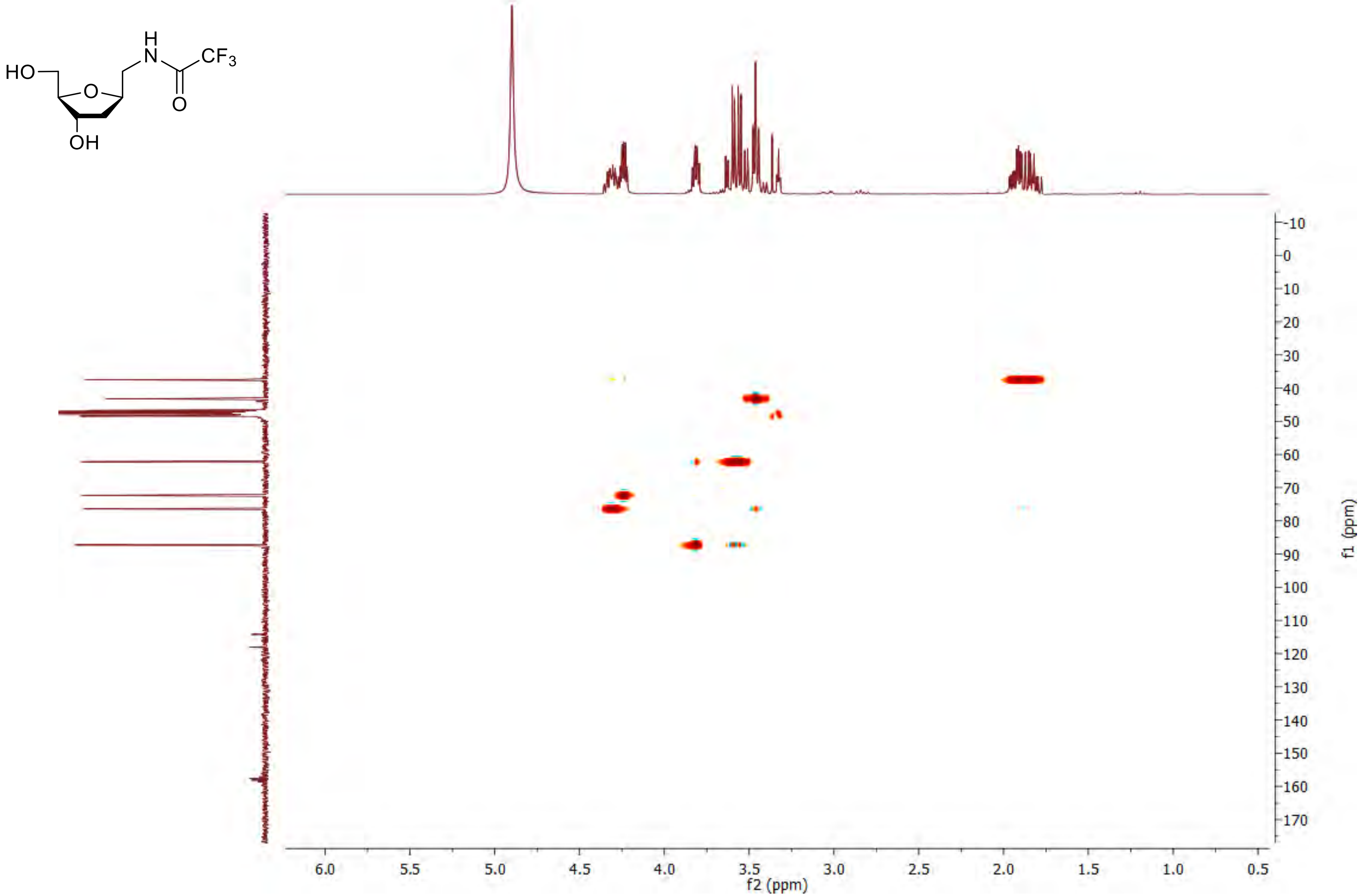

1,2-Dideoxy-1β-[*N*-(trifluoroacetyl)aminomethyl]-D-*erythro*-pentofuranose (3β)

HMBC NMR (MeOH-d_4)

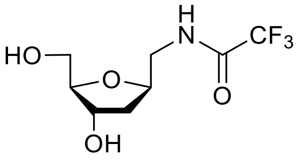

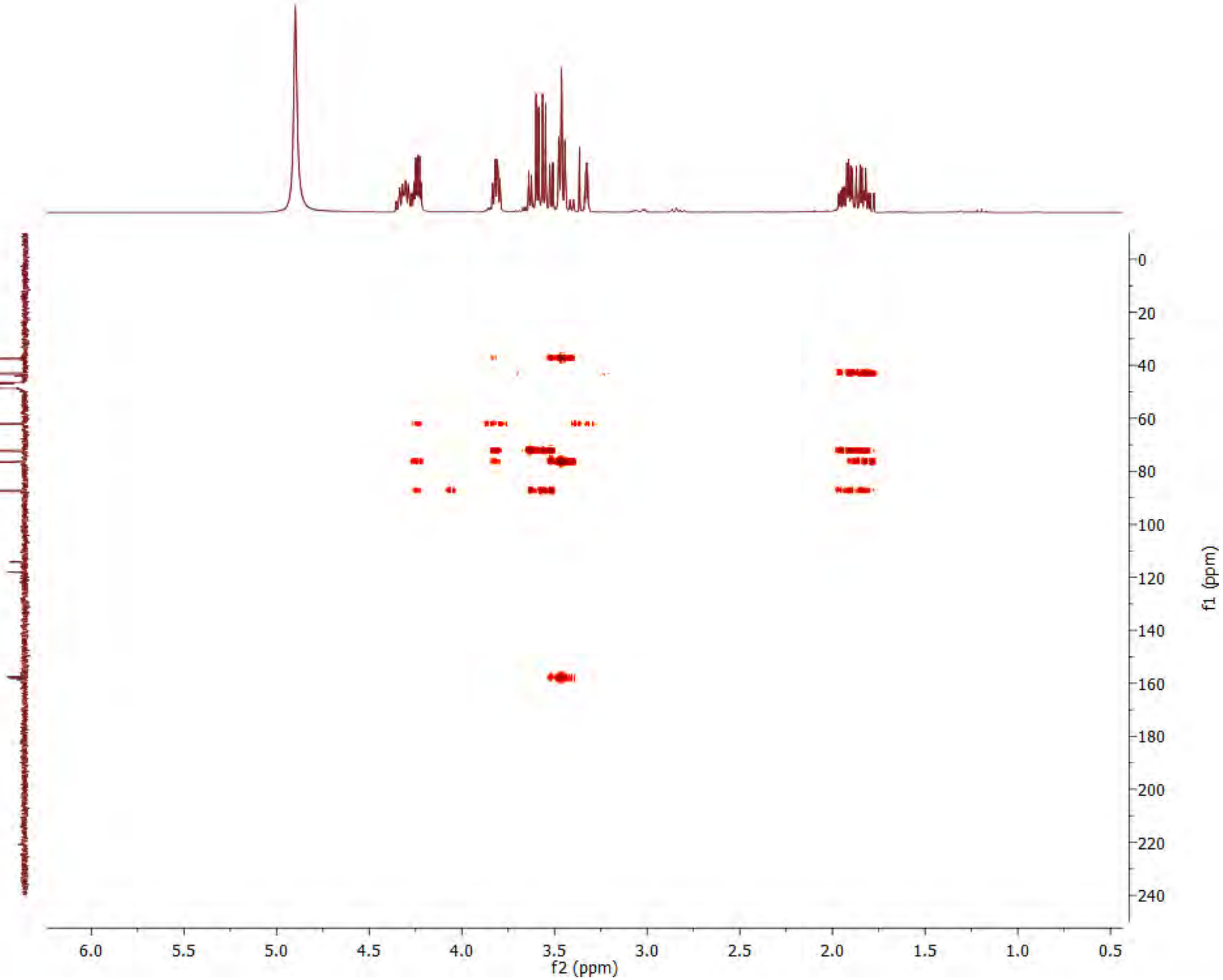

1,2-Dideoxy-5-*O*-(4,4'-dimethoxytrityl)-1α-[*N*-(trifluoroacetyl)aminomethyl]-D-*erythro*-pentofuranose (4α)

^{1}H NMR (300.13 MHz, MeOH-d_4)

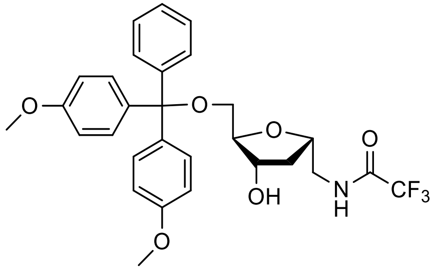

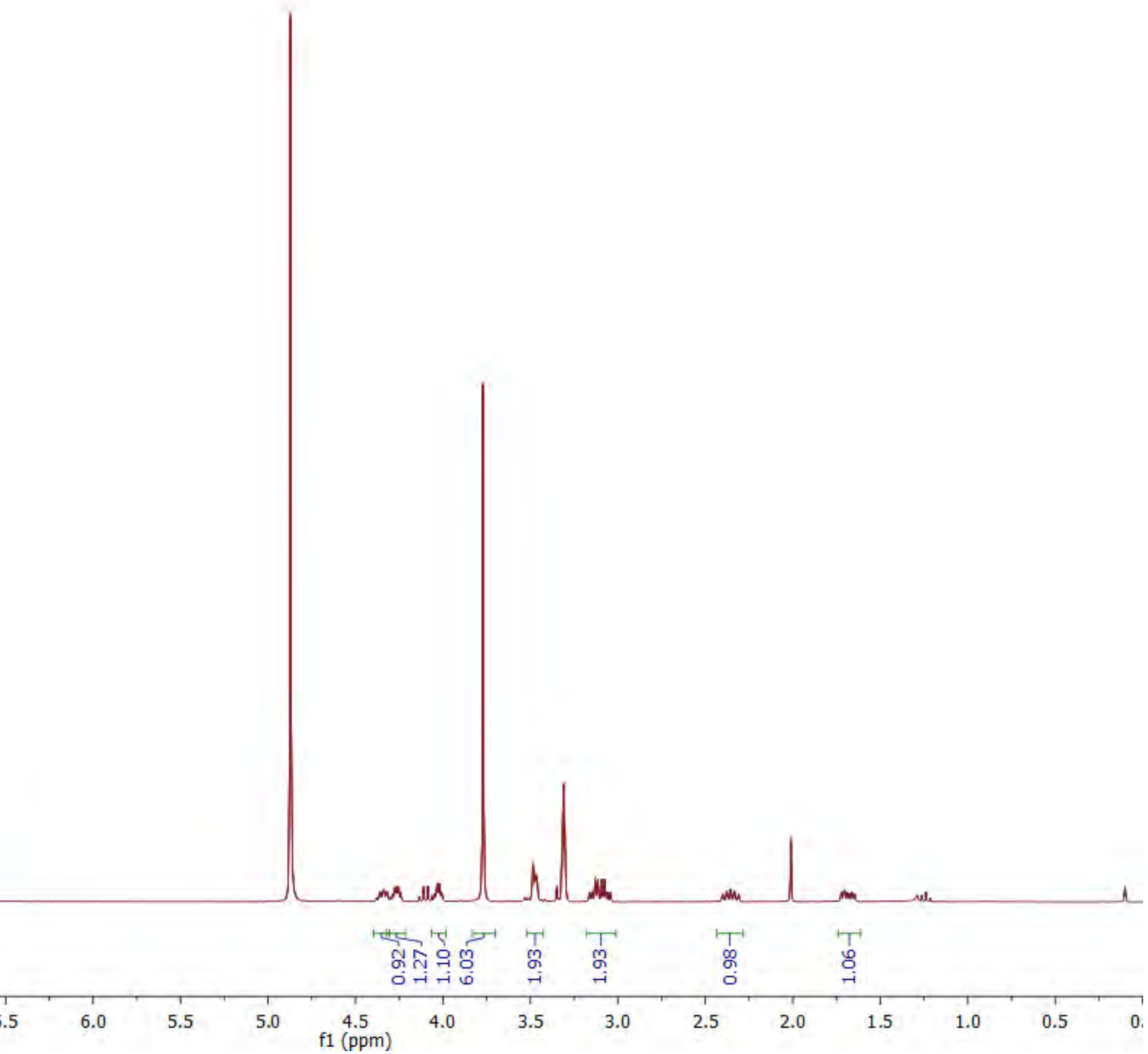

1,2-Dideoxy-5-*O*-(4,4'-dimethoxytrityl)-1α-[*N*-(trifluoroacetyl)aminomethyl]-D-*erythro*-pentofuranose (4α)

^{13}C NMR (75.5 MHz, MeOH-d_4)

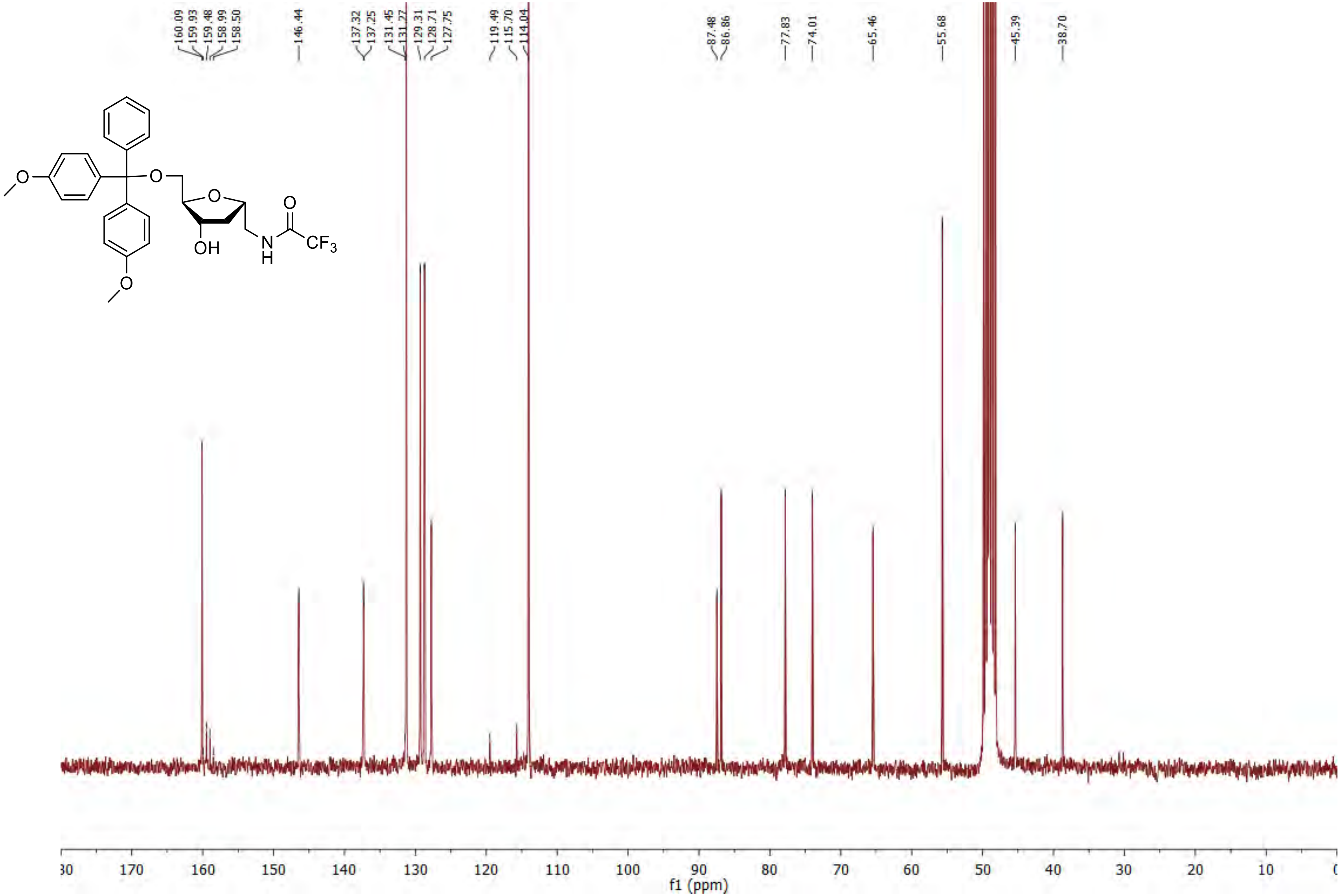

1,2-Dideoxy-5-*O*-(4,4'-dimethoxytrityl)-1α-[*N*-(trifluoroacetyl)aminomethyl]-D-*erythro*-pentofuranose (4α)

DEPT135 NMR (75.5 MHz, MeOH-d_4)

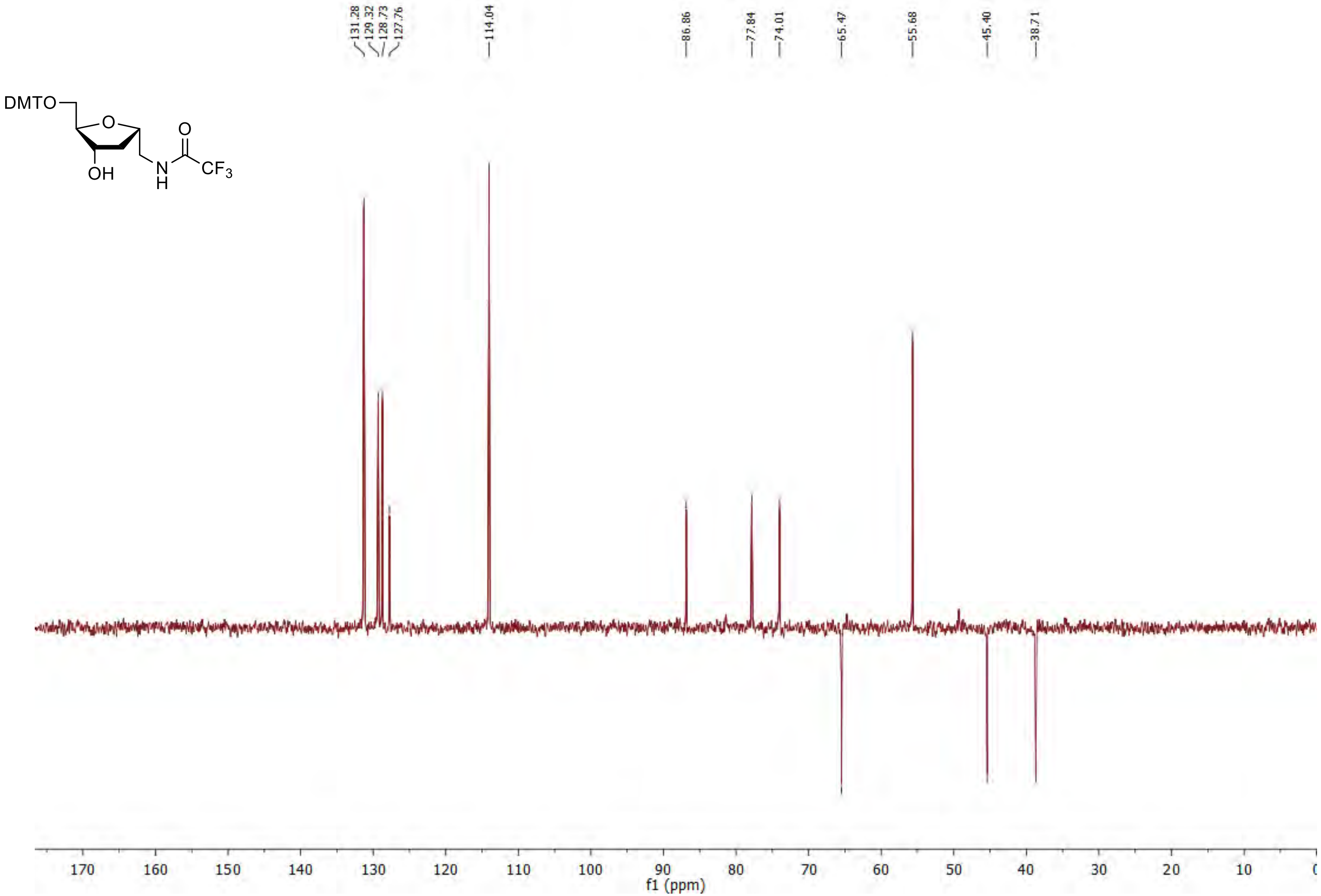

1,2-Dideoxy-5-*O*-(4,4'-dimethoxytrityl)-1α-[*N*-(trifluoroacetyl)aminomethyl]-D-*erythro*-pentofuranose (4α)

COSY NMR (75.5 MHz, MeOH-d_4)

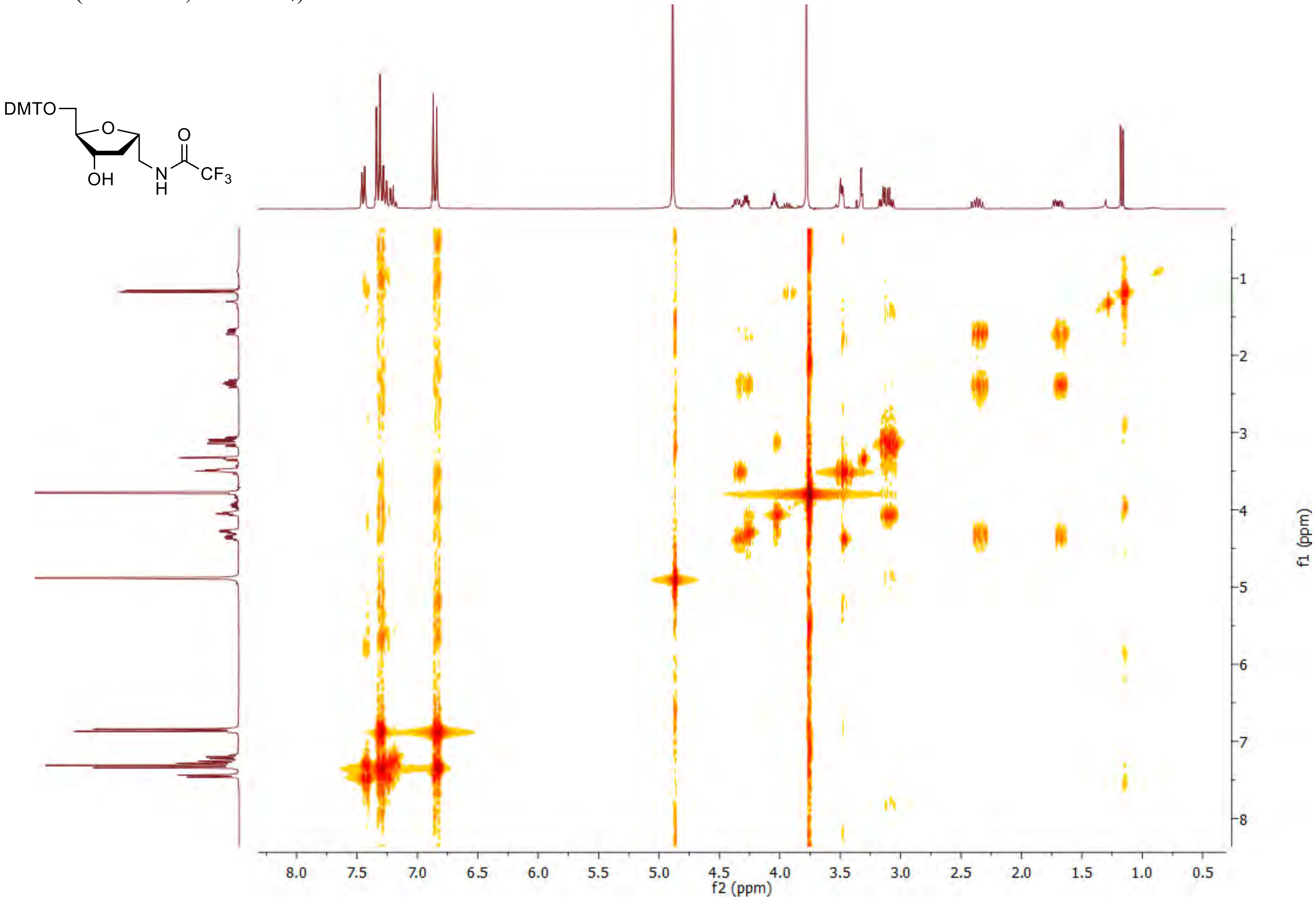

1,2-Dideoxy-5-*O*-(4,4'-dimethoxytrityl)-1α-[*N*-(trifluoroacetyl)aminomethyl]-D-*erythro*-pentofuranose (4α)

HSQC NMR (75.5 MHz, MeOH-d_4)

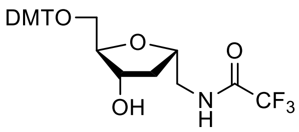

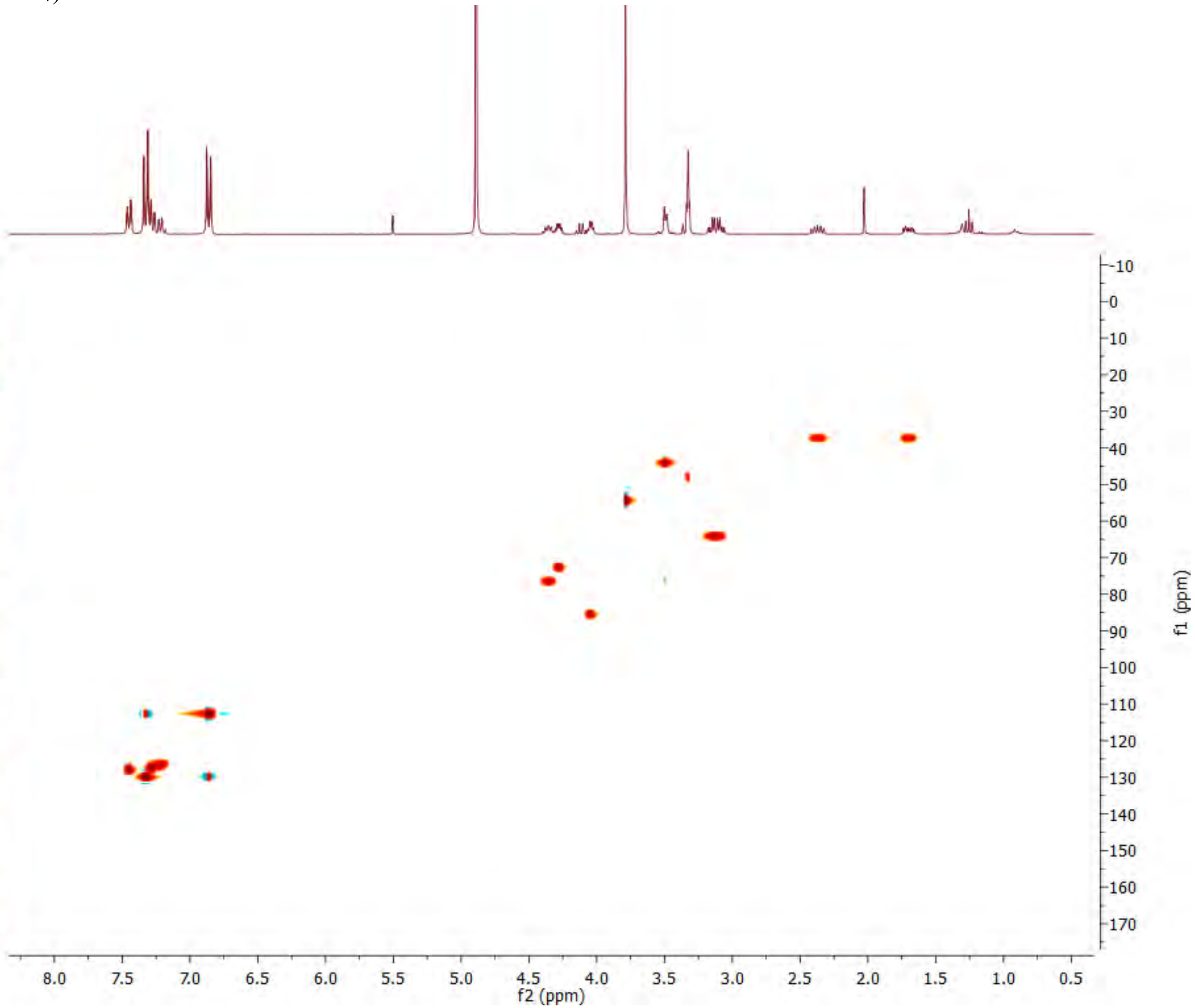

1,2-Dideoxy-5-*O*-(4,4'-dimethoxytrityl)-1α-[*N*-(trifluoroacetyl)aminomethyl]-D-*erythro*-pentofuranose (4α)

HMBC NMR (75.5 MHz, MeOH-d_4)

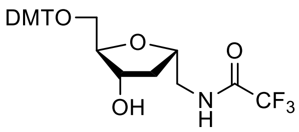

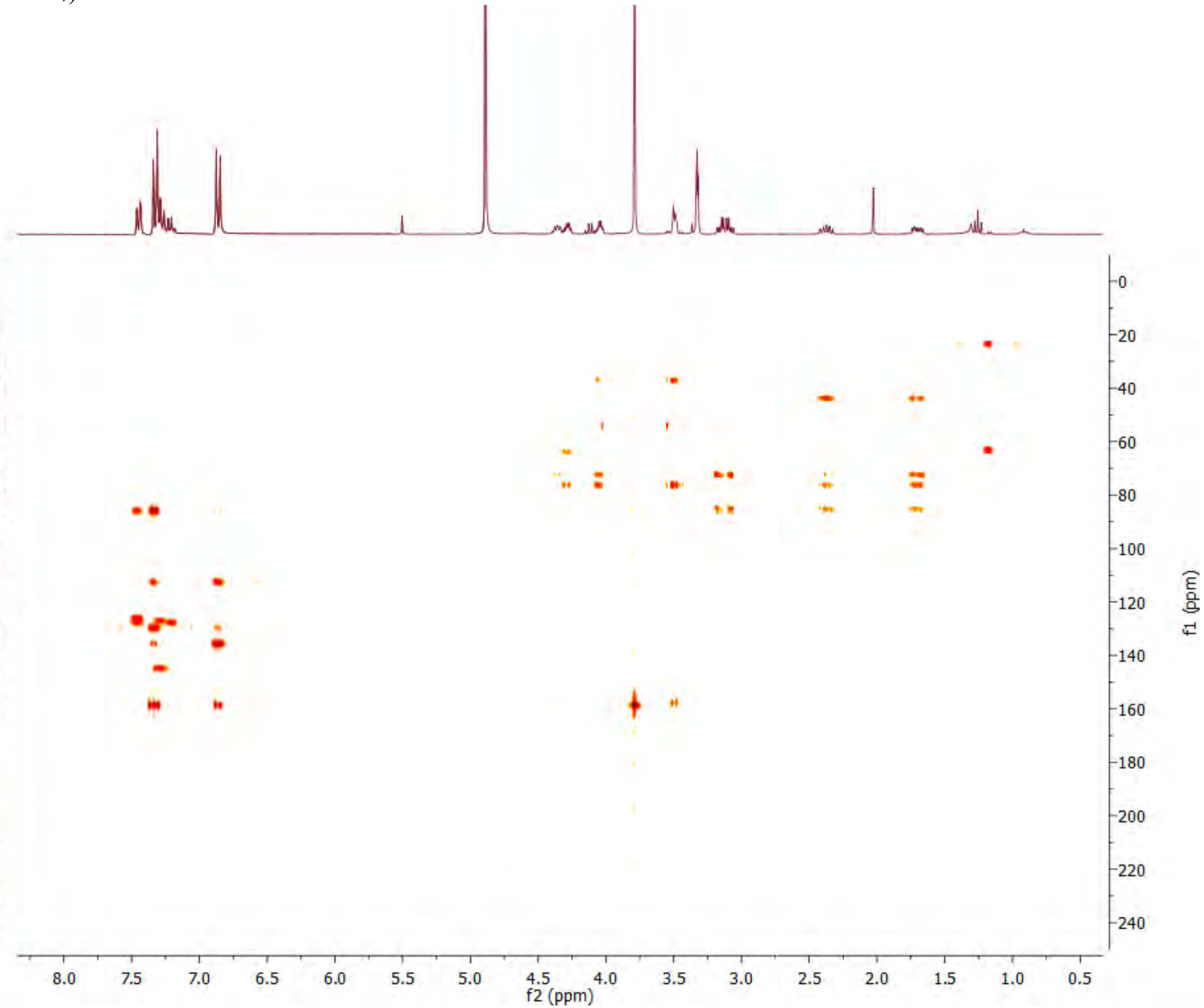

1,2-Dideoxy-5-*O*-(4,4’-dimethoxytrityl)-1β-[*N*-(trifluoroacetyl)aminomethyl]-D-*erythro*-pentofuranose (4β)

^{1}H NMR (300.13 MHz, MeOH-d_4)

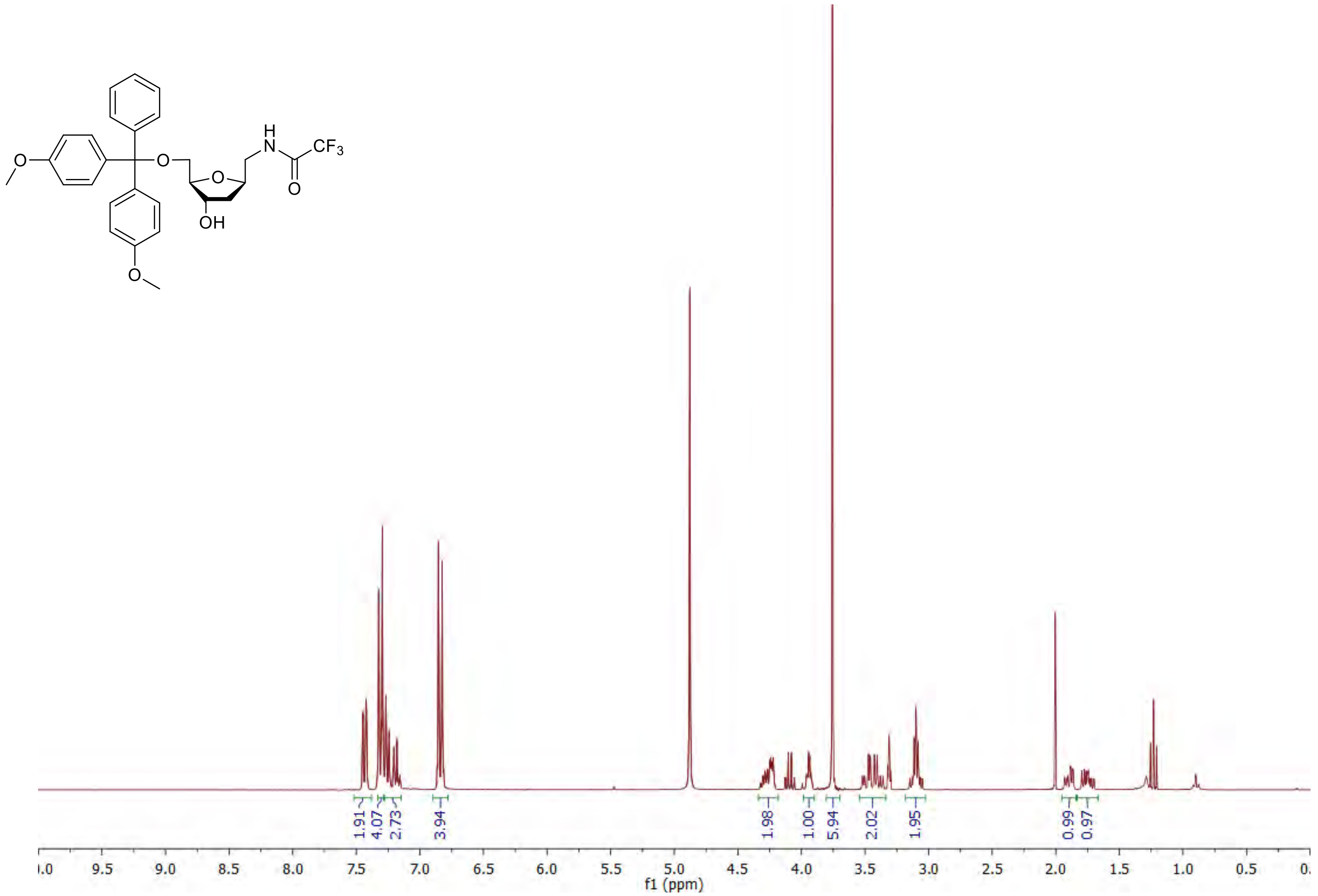

1,2-Dideoxy-5-*O*-(4,4’-dimethoxytrityl)-1β-[*N*-(trifluoroacetyl)aminomethyl]-D-*erythro*-pentofuranose (4β)

^{13}C NMR (75.5 MHz, MeOH-d_4)

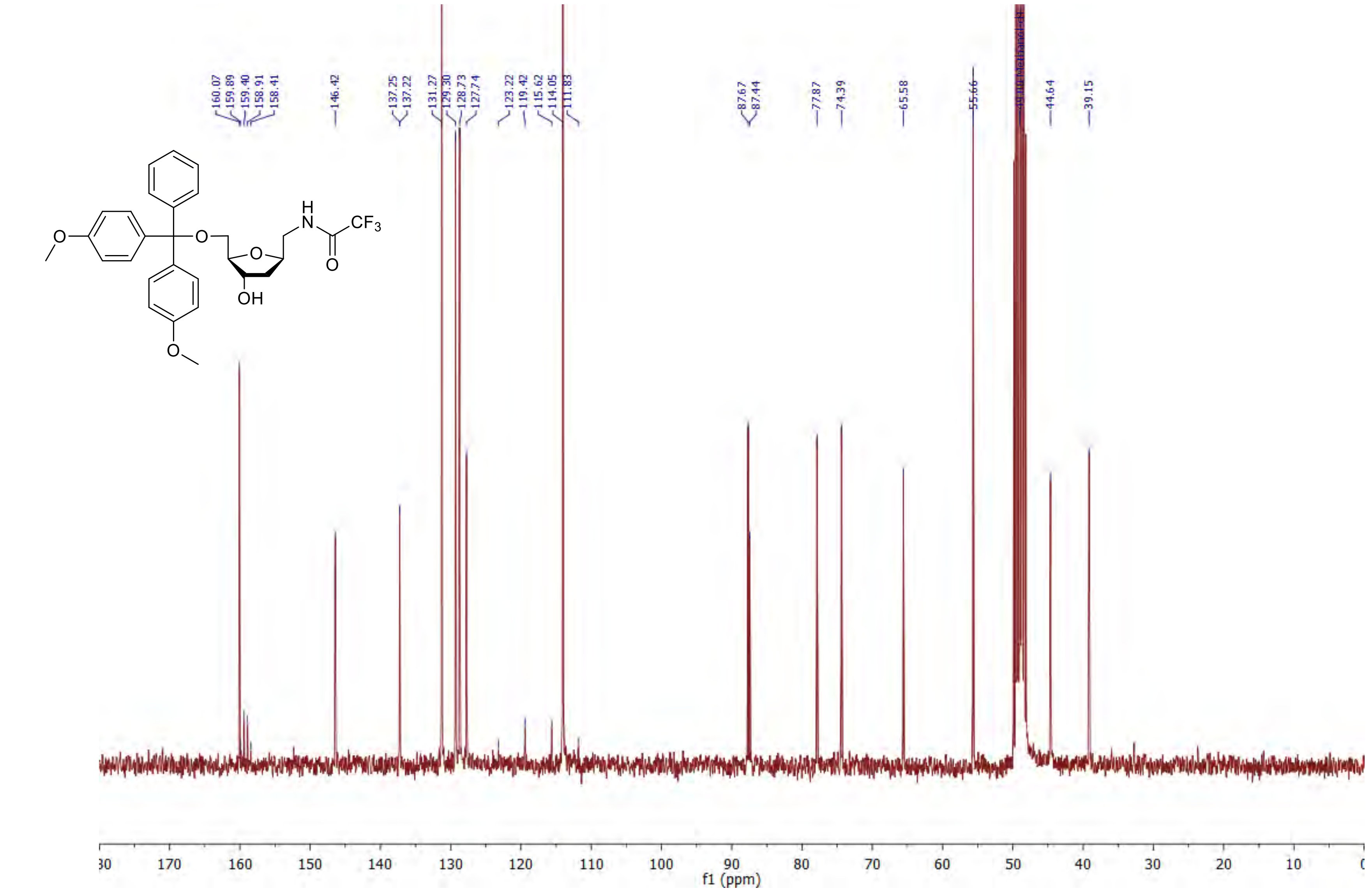

1,2-Dideoxy-5-*O*-(4,4'-dimethoxytrityl)-1β-[*N*-(trifluoroacetyl)aminomethyl]-D-*erythro*-pentofuranose (4β)

DEPT 135 NMR (75.5 MHz, MeOH-d_4)

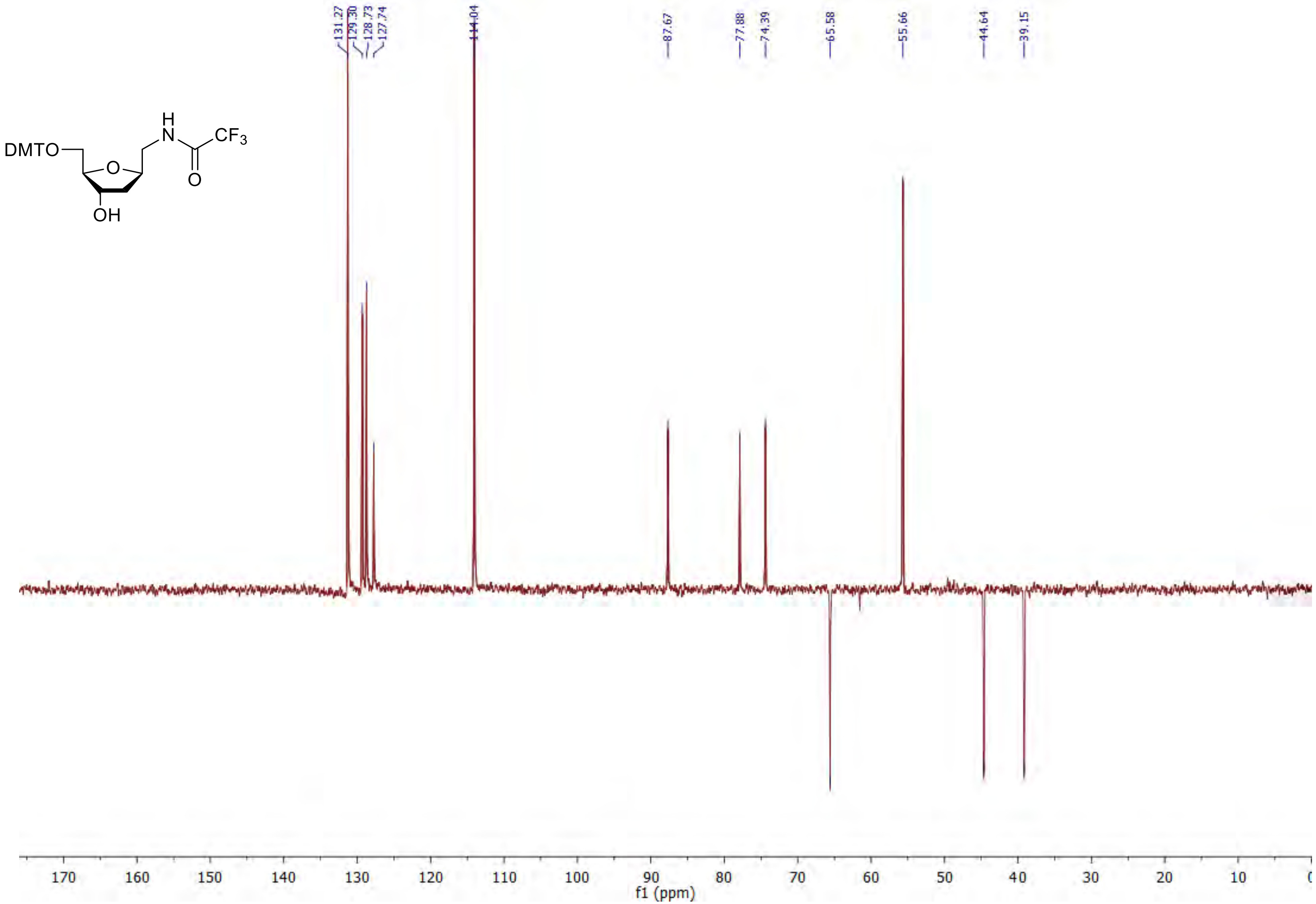

1,2-Dideoxy-5-*O*-(4,4'-dimethoxytrityl)-1β-[*N*-(trifluoroacetyl)aminomethyl]-D-*erythro*-pentofuranose (4β)

COSY NMR (MeOH-d_4)

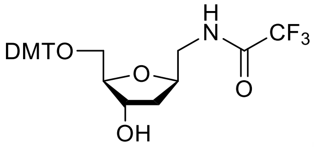

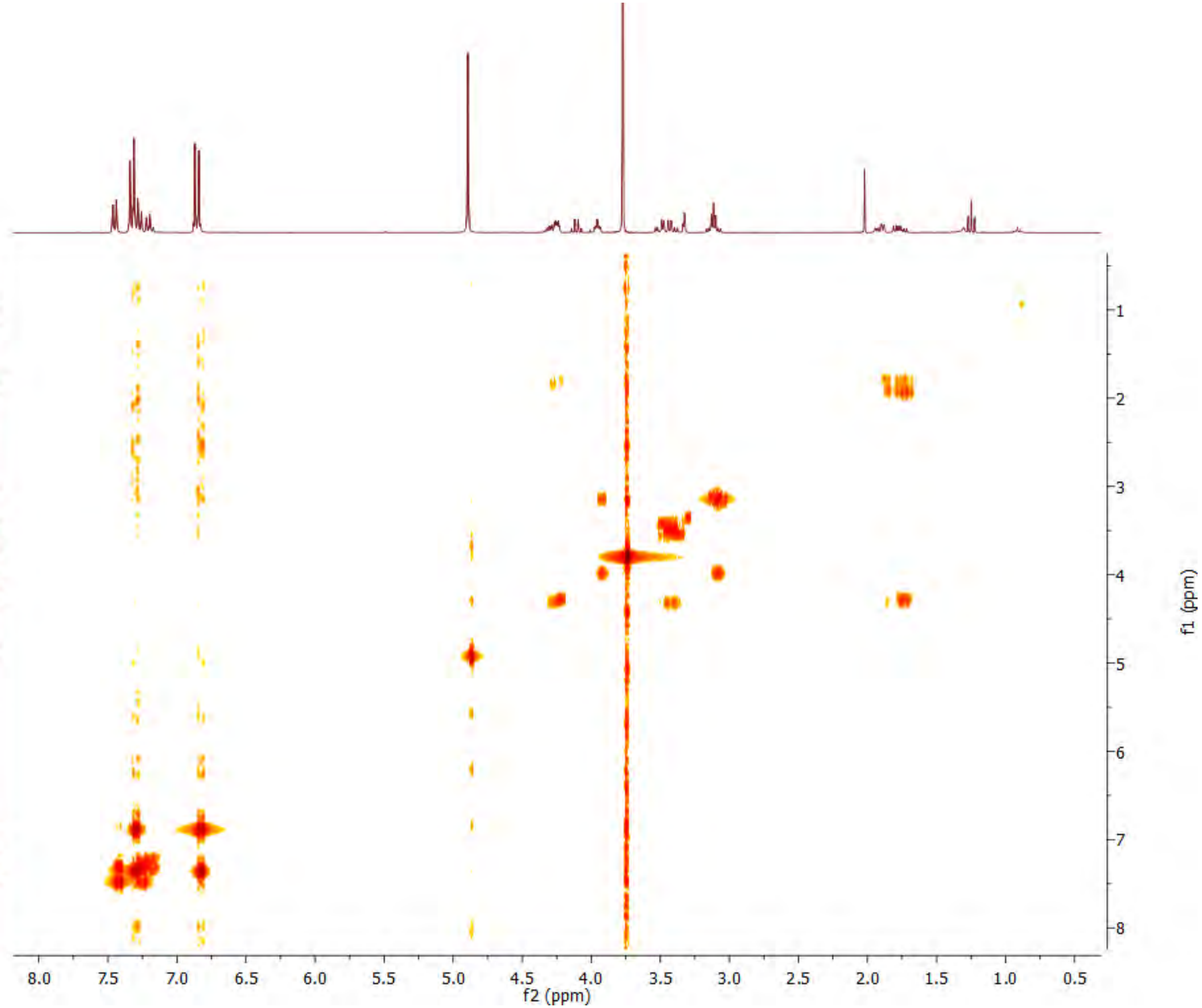

1,2-Dideoxy-5-*O*-(4,4'-dimethoxytrityl)-1β-[*N*-(trifluoroacetyl)aminomethyl]-D-*erythro*-pentofuranose (4β)

HSQC NMR (MeOH-d_4)

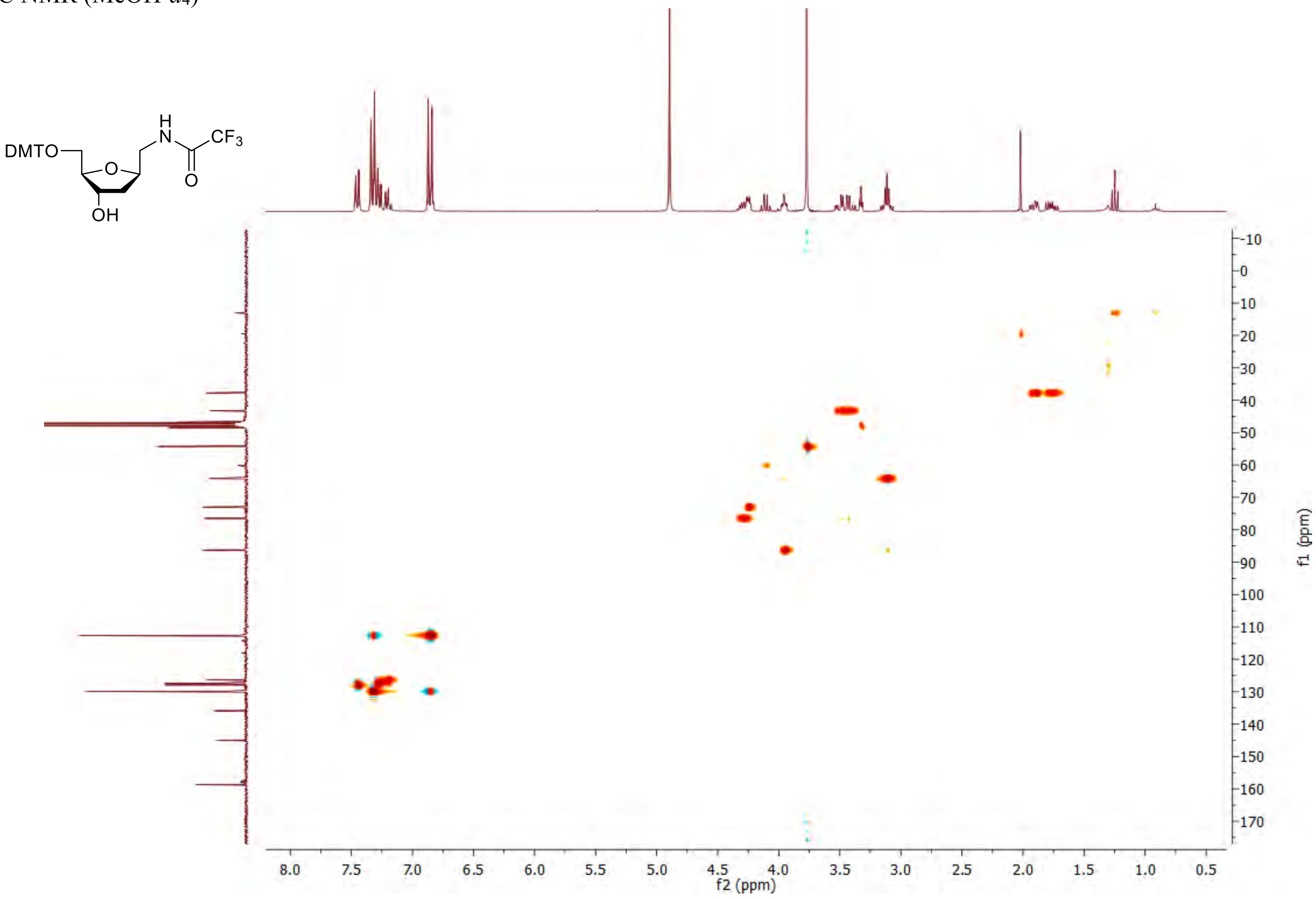

1,2-Dideoxy-5-*O*-(4,4'-dimethoxytrityl)-1β-[*N*-(trifluoroacetyl)aminomethyl]-D-*erythro*-pentofuranose (4β)

HMBC NMR (MeOH-d_4)

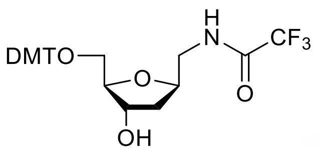

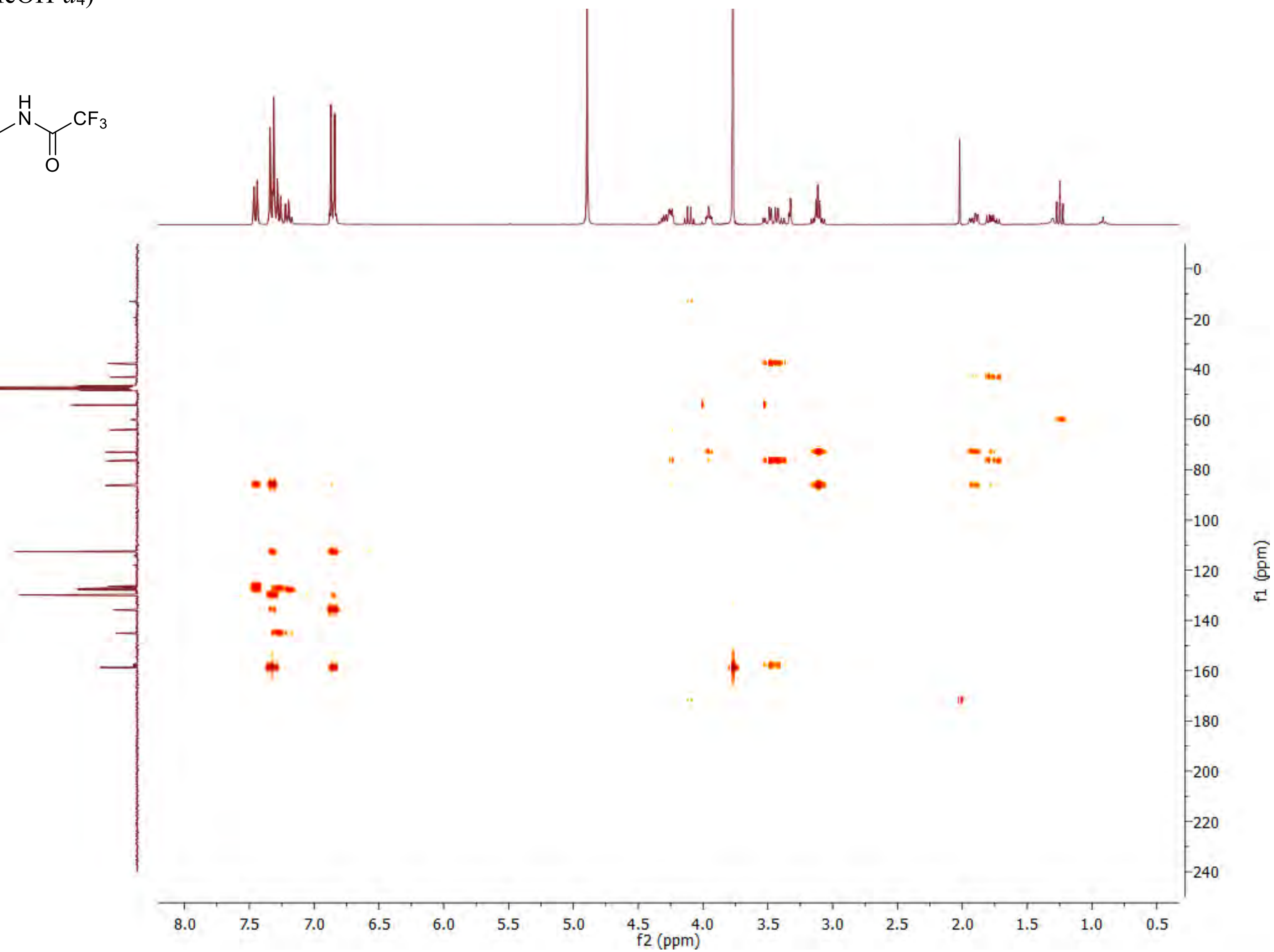

1,2-Dideoxy-5-*O*-(4,4'-dimethoxytrityl)-1α-[*N*-(trifluoroacetyl)aminomethyl]-D-*erythro*-pentofuranosyl-3-*O*-(2-cyanoethyl-*N*,*N*-diisopropyl)phosphoramidite (5α-A)

^{1}H NMR (300.13 MHz, MeOH-d_4)

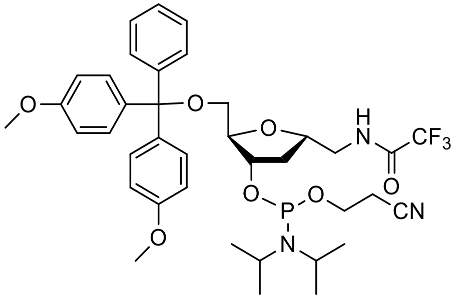

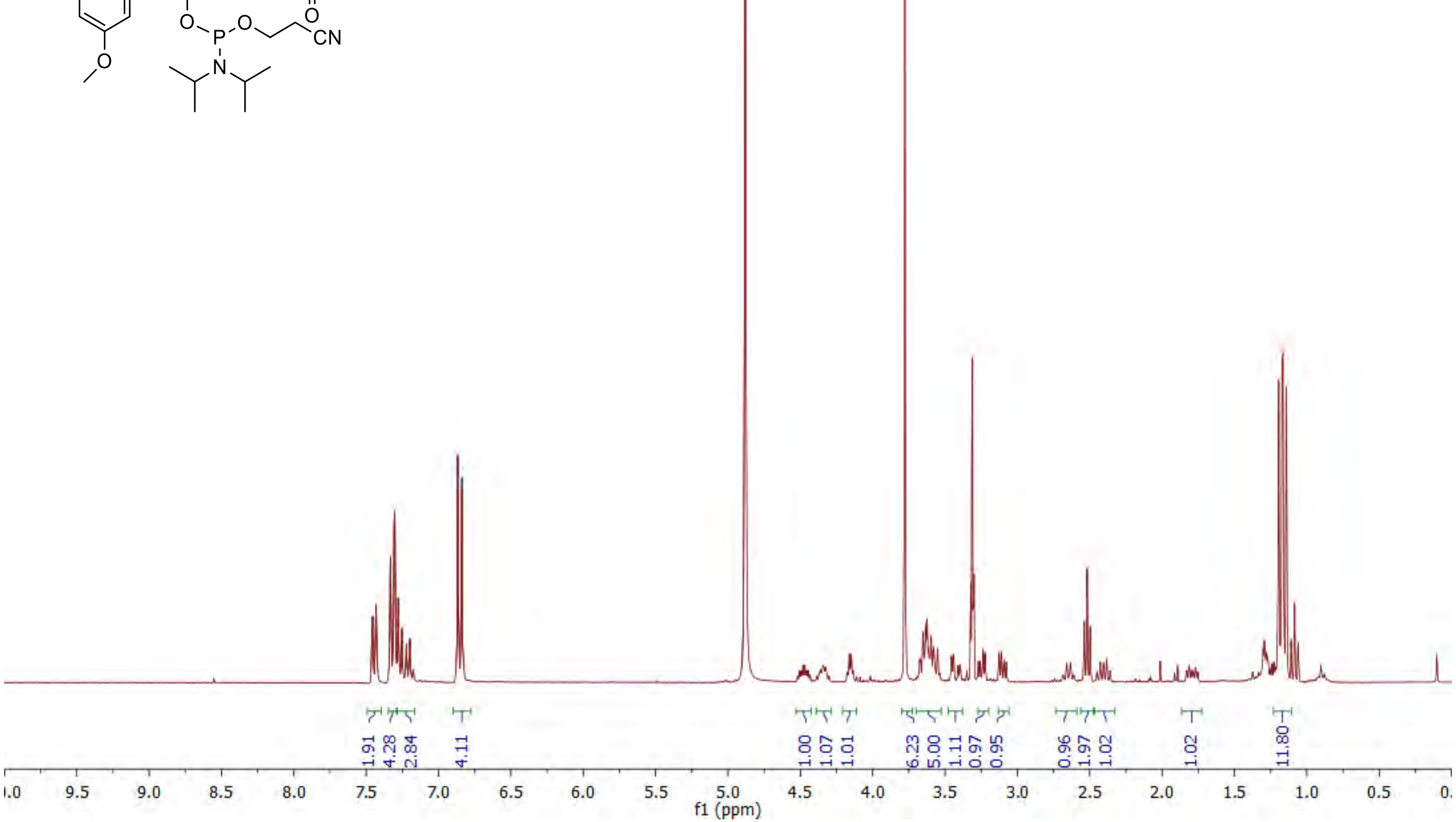

1,2-Dideoxy-5-*O*-(4,4’-dimethoxytrityl)-1α-[*N*-(trifluoroacetyl)aminomethyl]-ᴅ-*erythro*-pentofuranosyl-3-*O*-(2-cyanoethyl-*N*,*N*-diisopropyl)phosphoramidite (5α-A)

^{13}C NMR (75.5 MHz, MeOH-d_4)

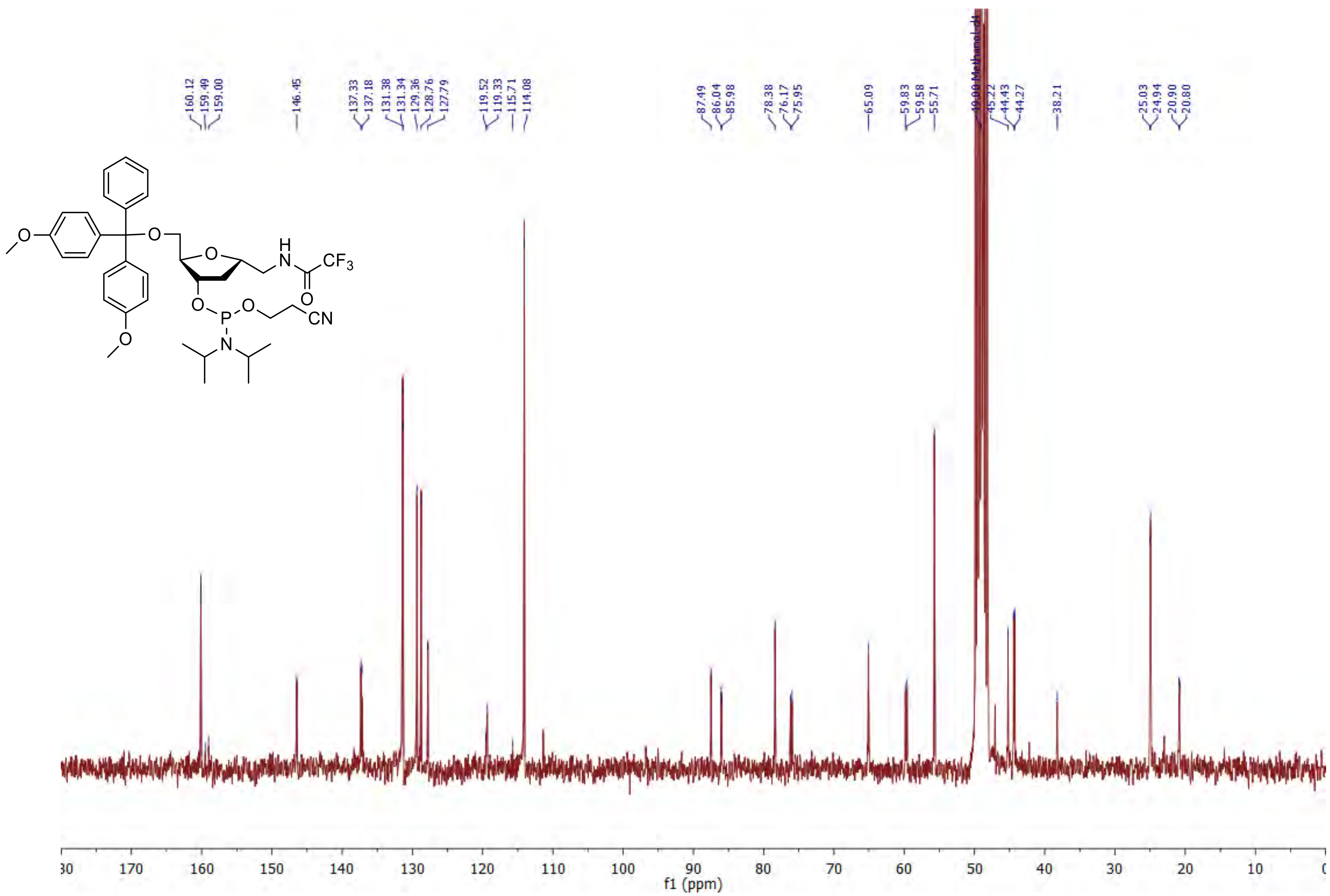

1,2-Dideoxy-5-*O*-(4,4'-dimethoxytrityl)-1α-[*N*-(trifluoroacetyl)aminomethyl]-D-*erythro*-pentofuranosyl-3-*O*-(2-cyanoethyl-*N*,*N*-diisopropyl)phosphoramidite (5α-A)

DEPT135 NMR (75.5 MHz, MeOH-d_4)

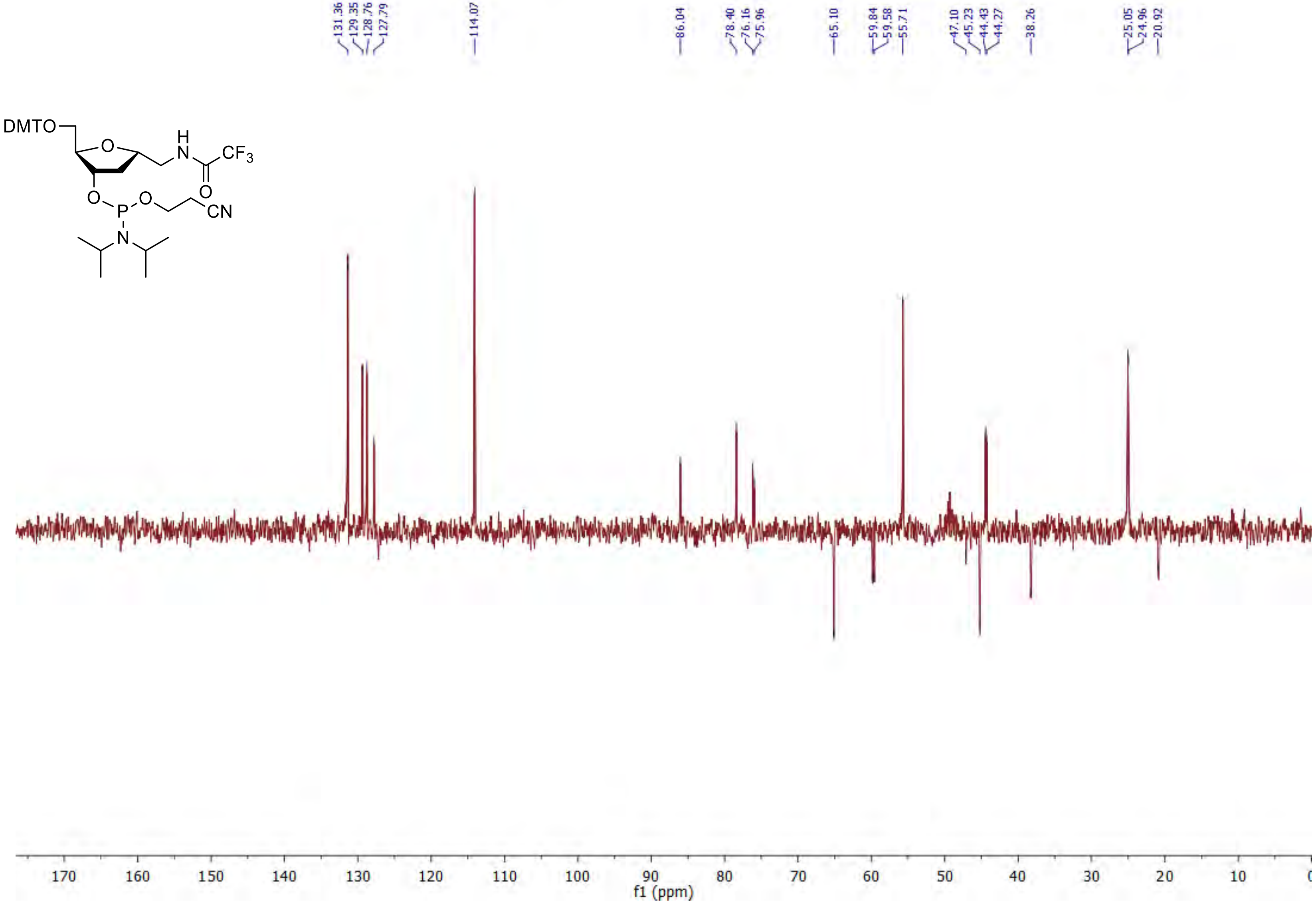

1,2-Dideoxy-5-*O*-(4,4'-dimethoxytrityl)-1α-[*N*-(trifluoroacetyl)aminomethyl]-D-*erythro*-pentofuranosyl-3-*O*-(2-cyanoethyl-*N*,*N*-diisopropyl)phosphoramidite (5α-A)

COSY NMR (MeOH-d_4)

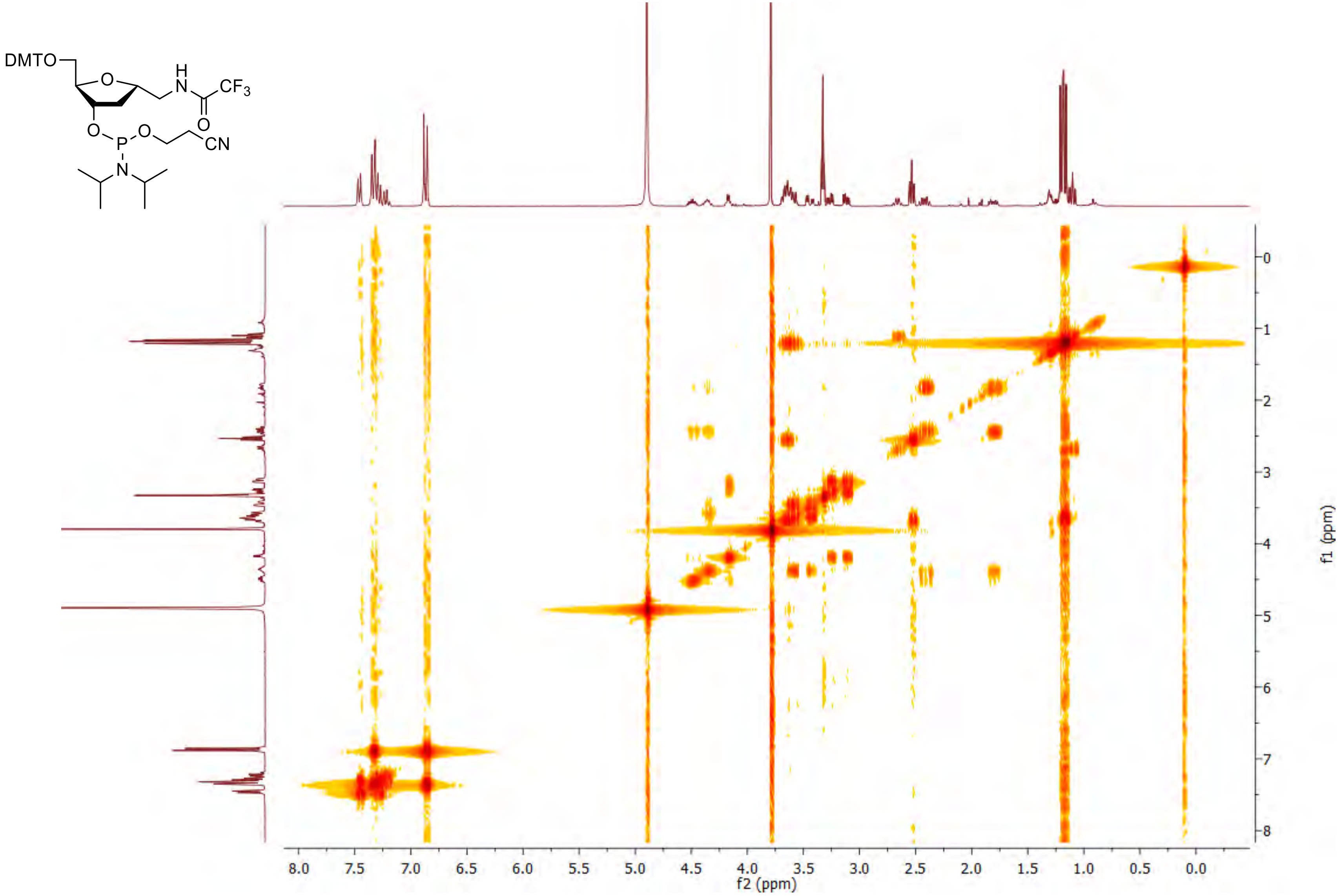

1,2-Dideoxy-5-*O*-(4,4'-dimethoxytrityl)-1α-[*N*-(trifluoroacetyl)aminomethyl]-D-*erythro*-pentofuranosyl-3-*O*-(2-cyanoethyl-*N*,*N*-diisopropyl)phosphoramidite (5α-A)

HSQC NMR (MeOH-d_4)

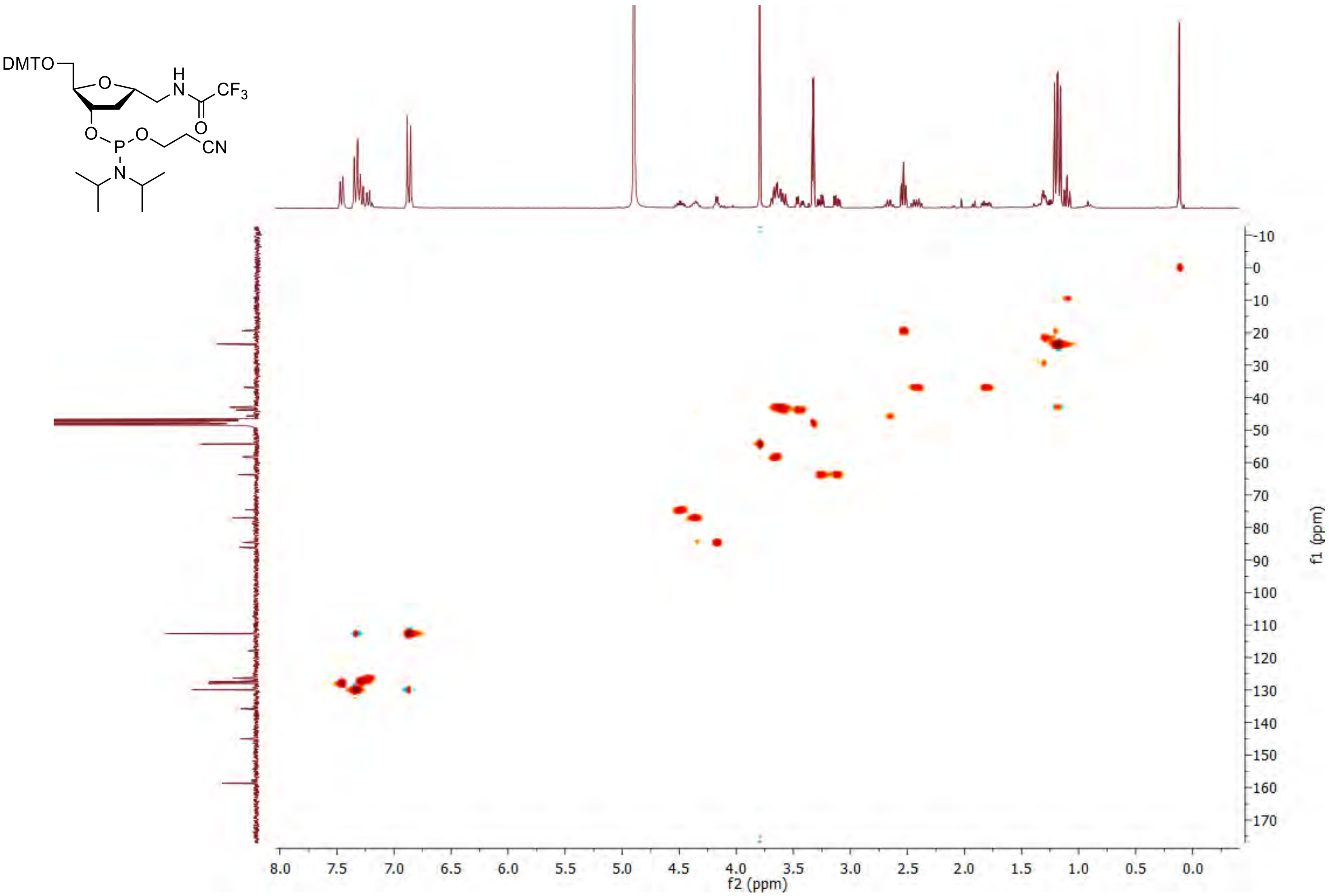

1,2-Dideoxy-5-*O*-(4,4'-dimethoxytrityl)-1α-[*N*-(trifluoroacetyl)aminomethyl]-D-*erythro*-pentofuranosyl-3-*O*-(2-cyanoethyl-*N*,*N*-diisopropyl)phosphoramidite (5α-A)

HMBC NMR (MeOH-d_4)

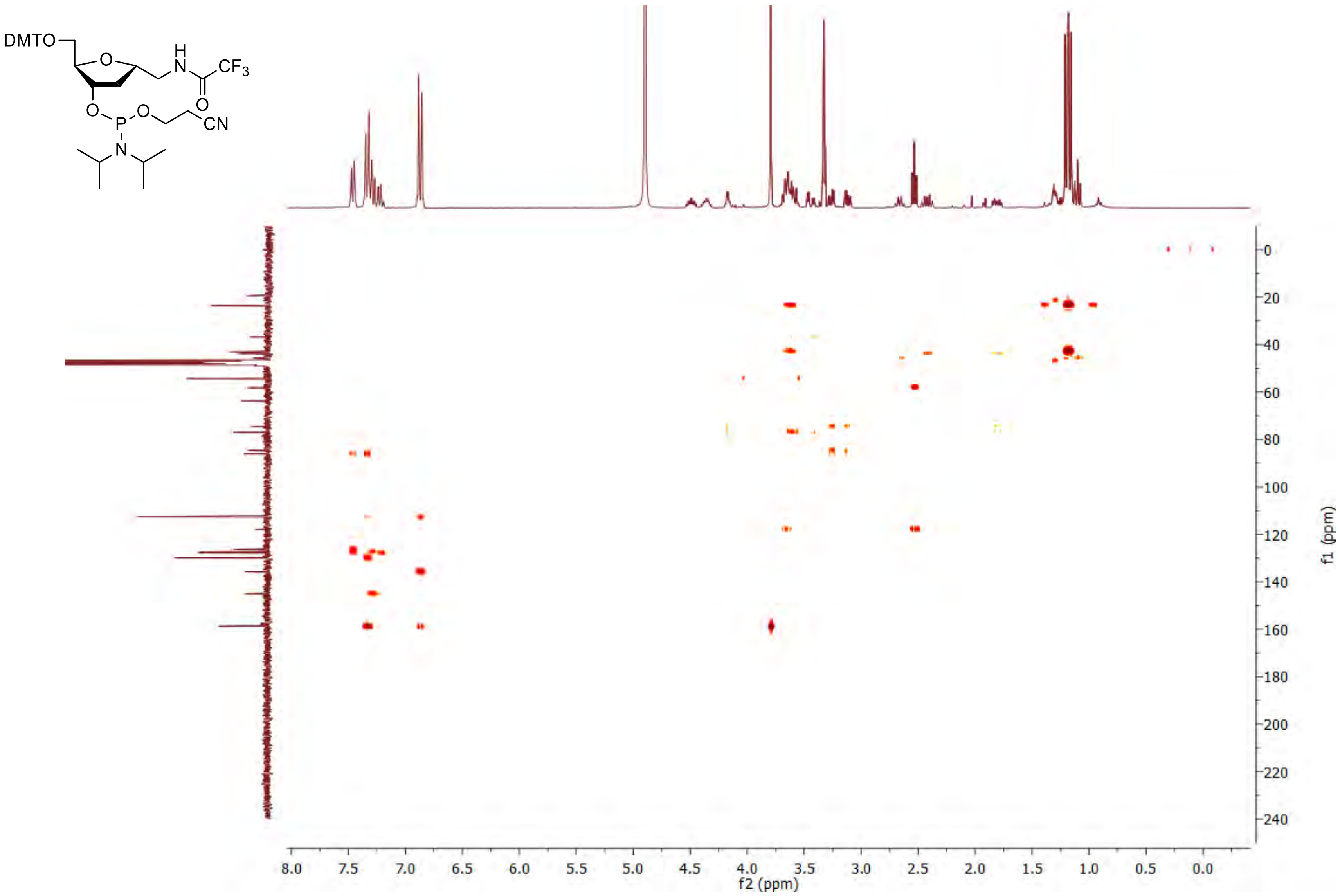

1,2-Dideoxy-5-*O*-(4,4'-dimethoxytrityl)-1α-[*N*-(trifluoroacetyl)aminomethyl]-D-*erythro*-pentofuranosyl-3-*O*-(2-cyanoethyl-*N*,*N*-diisopropyl)phosphoramidite (5α-A)

^{31}P NMR (121.5 MHz, MeOH-d_4)

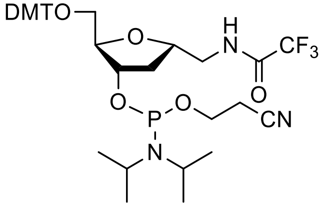

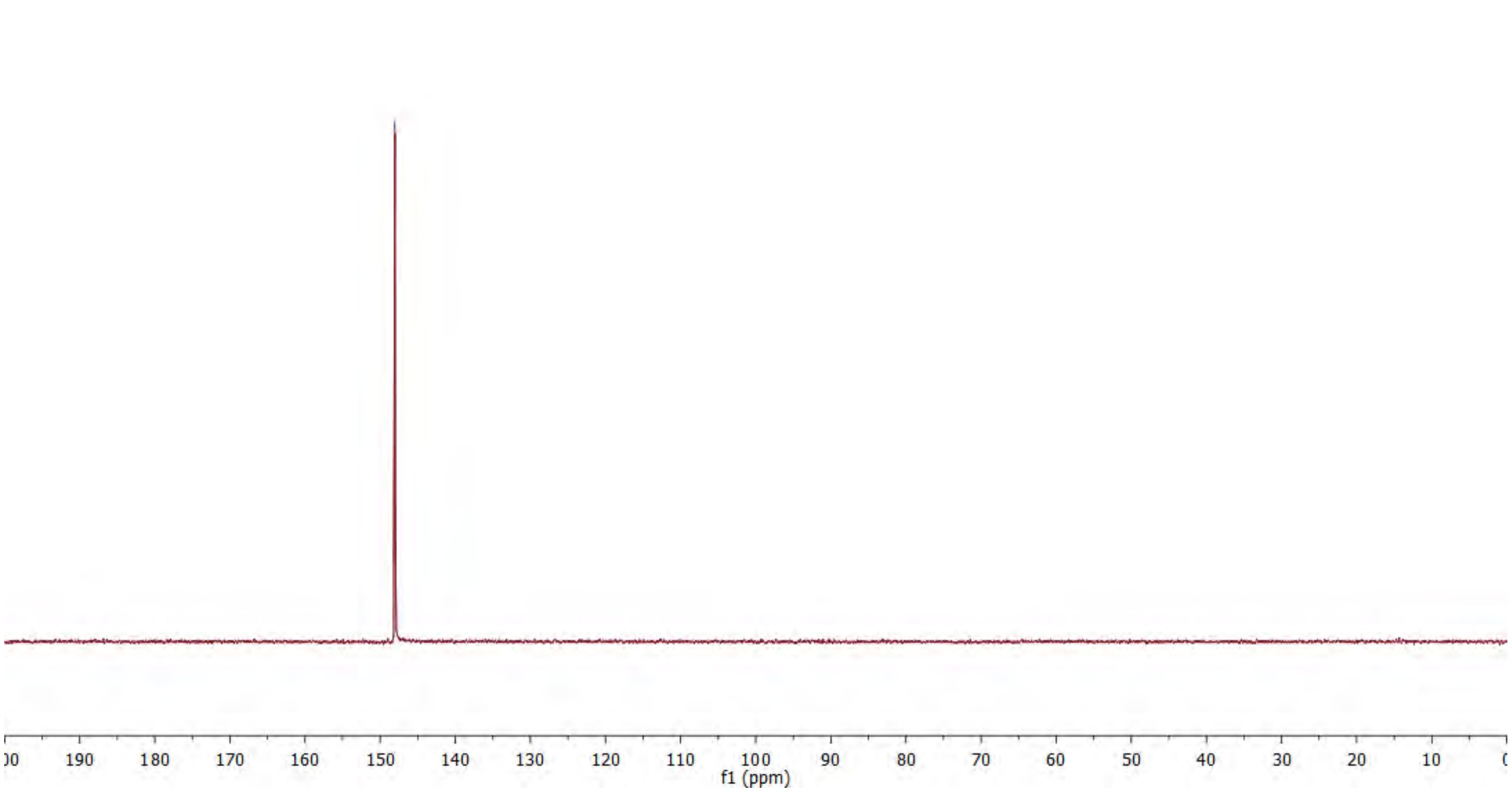

1,2-Dideoxy-5-*O*-(4,4'-dimethoxytrityl)-1α-[*N*-(trifluoroacetyl)aminomethyl]-D-*erythro*-pentofuranosyl-3-*O*-(2-cyanoethyl-*N*,*N*-diisopropyl)phosphoramidite (5α-B)

^{1}H NMR (300.13 MHz, MeOH-d_4)

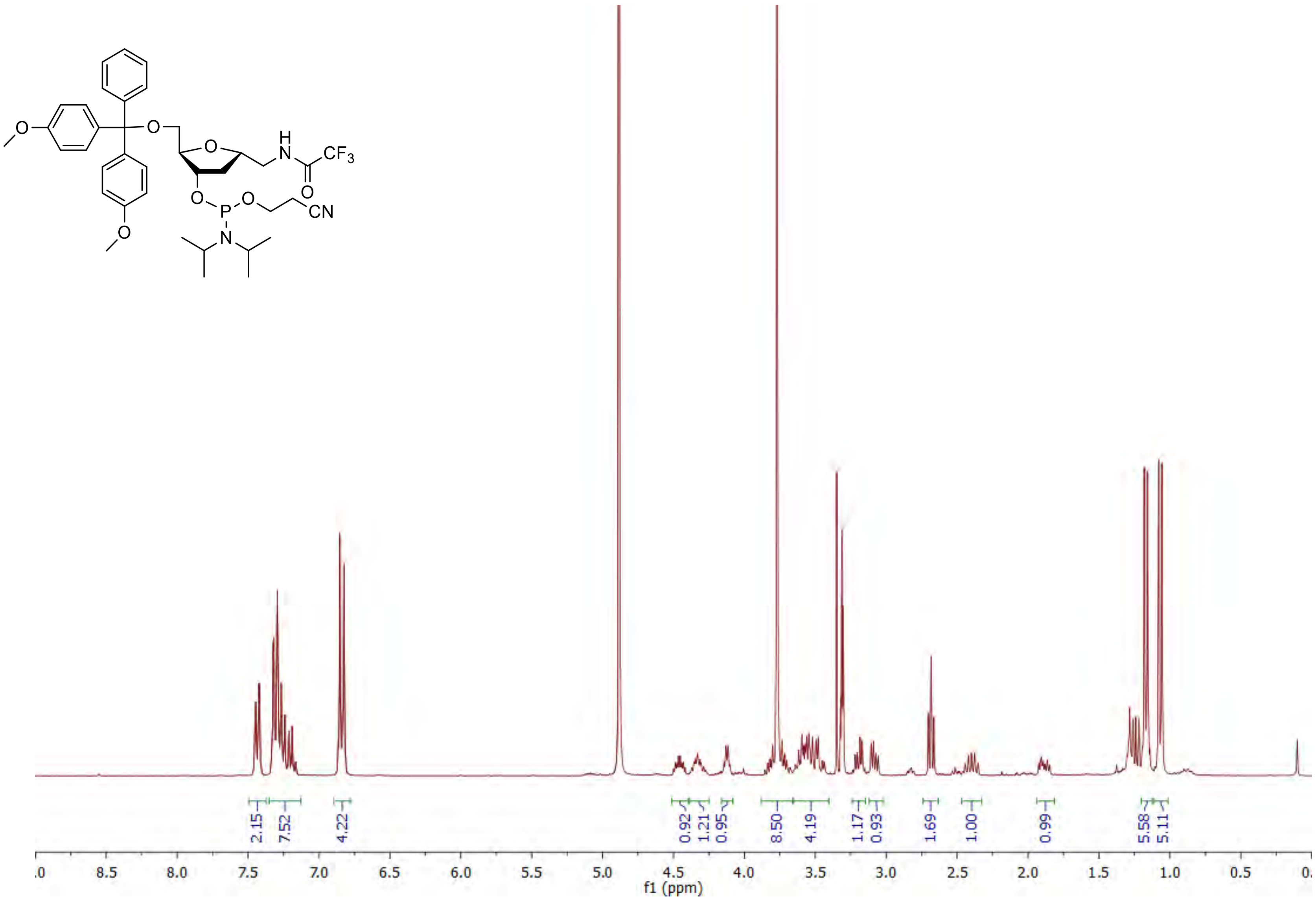

1,2-Dideoxy-5-*O*-(4,4'-dimethoxytrityl)-1α-[*N*-(trifluoroacetyl)aminomethyl]-D-*erythro*-pentofuranosyl-3-*O*-(2-cyanoethyl-*N*,*N*-diisopropyl)phosphoramidite (5α-B)

^{13}C NMR (75.5 MHz, MeOH-d_4)

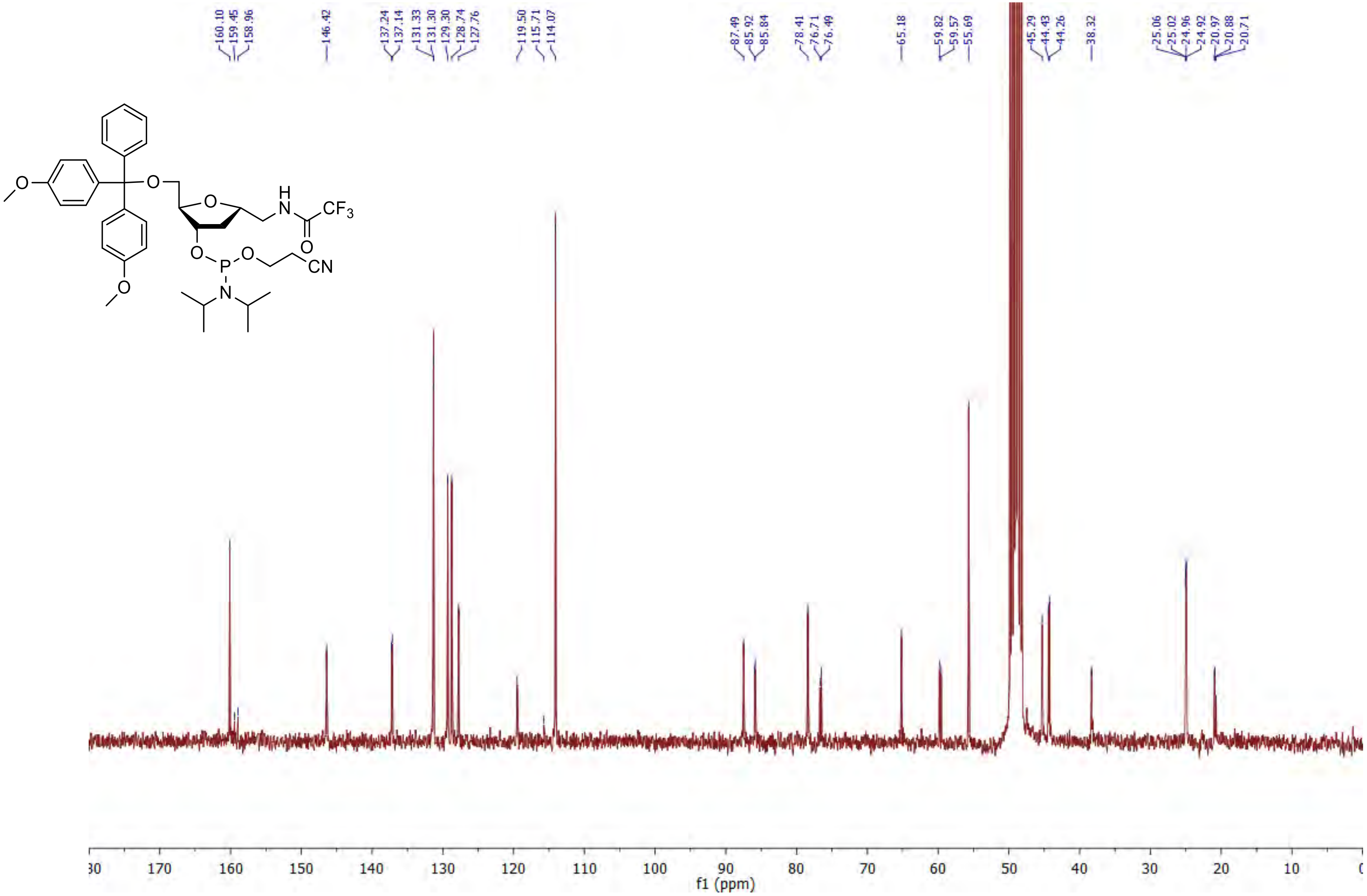

1,2-Dideoxy-5-*O*-(4,4'-dimethoxytrityl)-1α-[*N*-(trifluoroacetyl)aminomethyl]-D-*erythro*-pentofuranosyl-3-*O*-(2-cyanoethyl-*N*,*N*-diisopropyl)phosphoramidite (5α-B)

DEPT135 NMR (75.5 MHz, MeOH-d_4)

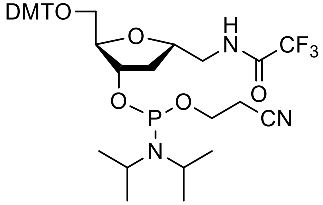

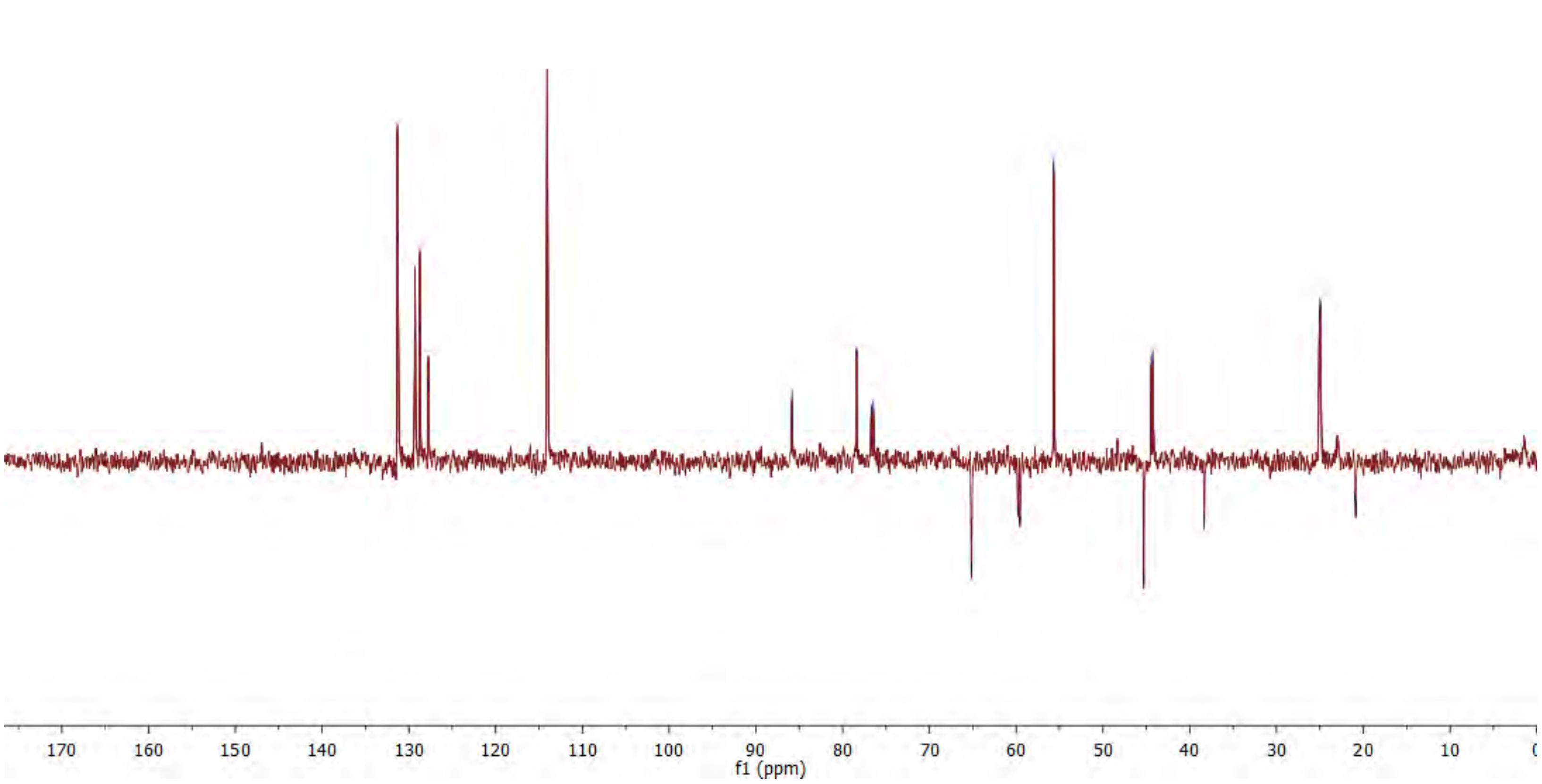

1,2-Dideoxy-5-*O*-(4,4'-dimethoxytrityl)-1α-[*N*-(trifluoroacetyl)aminomethyl]-ᴅ-*erythro*-pentofuranosyl-3-*O*-(2-cyanoethyl-*N*,*N*-diisopropyl)phosphoramidite (5α-B)

COSY NMR (MeOH-d_4)

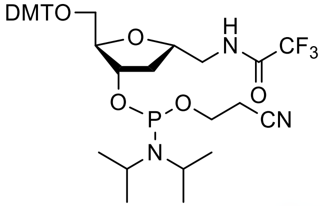

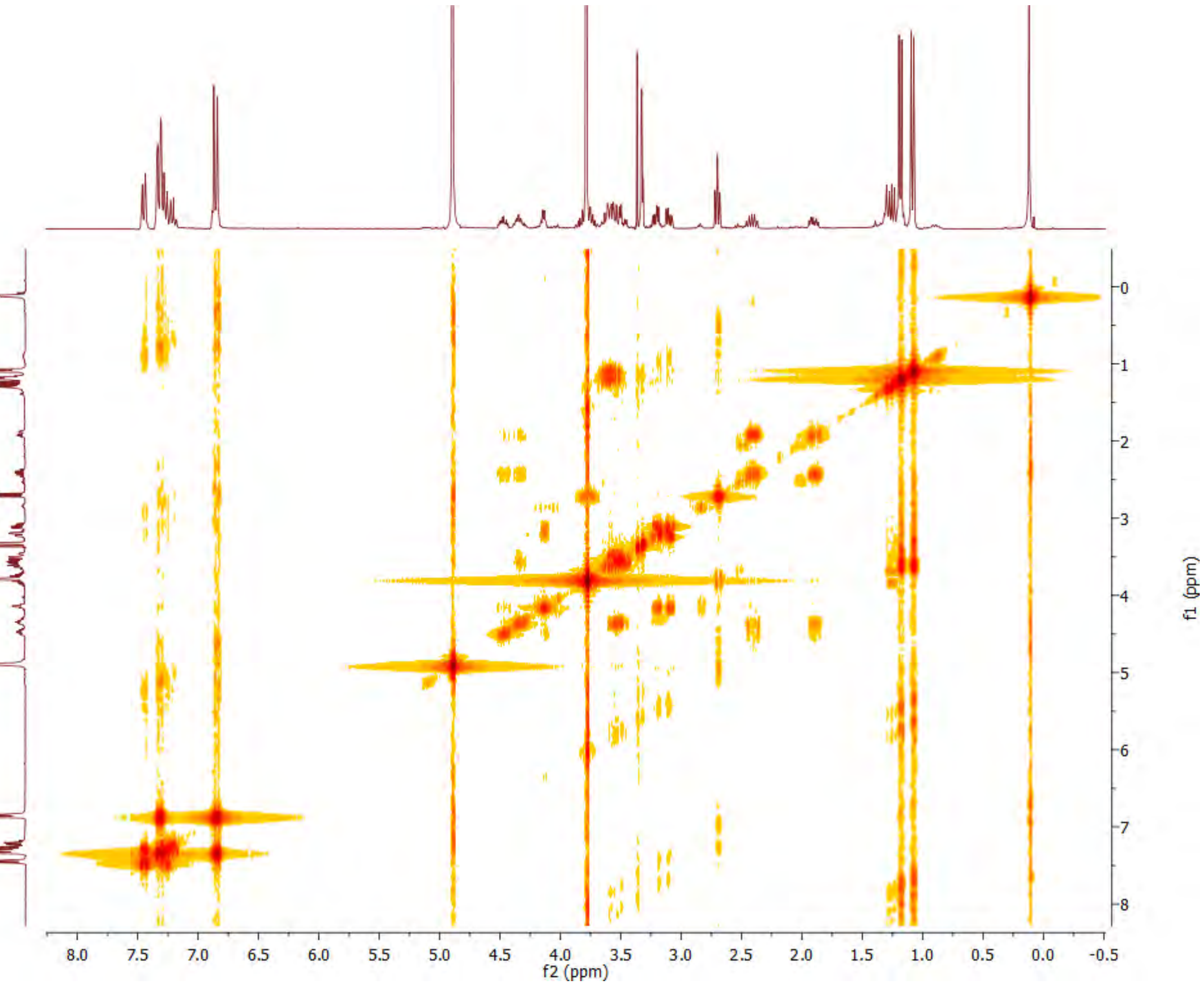

1,2-Dideoxy-5-*O*-(4,4'-dimethoxytrityl)-1α-[*N*-(trifluoroacetyl)aminomethyl]-D-*erythro*-pentofuranosyl-3-*O*-(2-cyanoethyl-*N*,*N*-diisopropyl)phosphoramidite (5α-B)

HSQC NMR (MeOH-d_4)

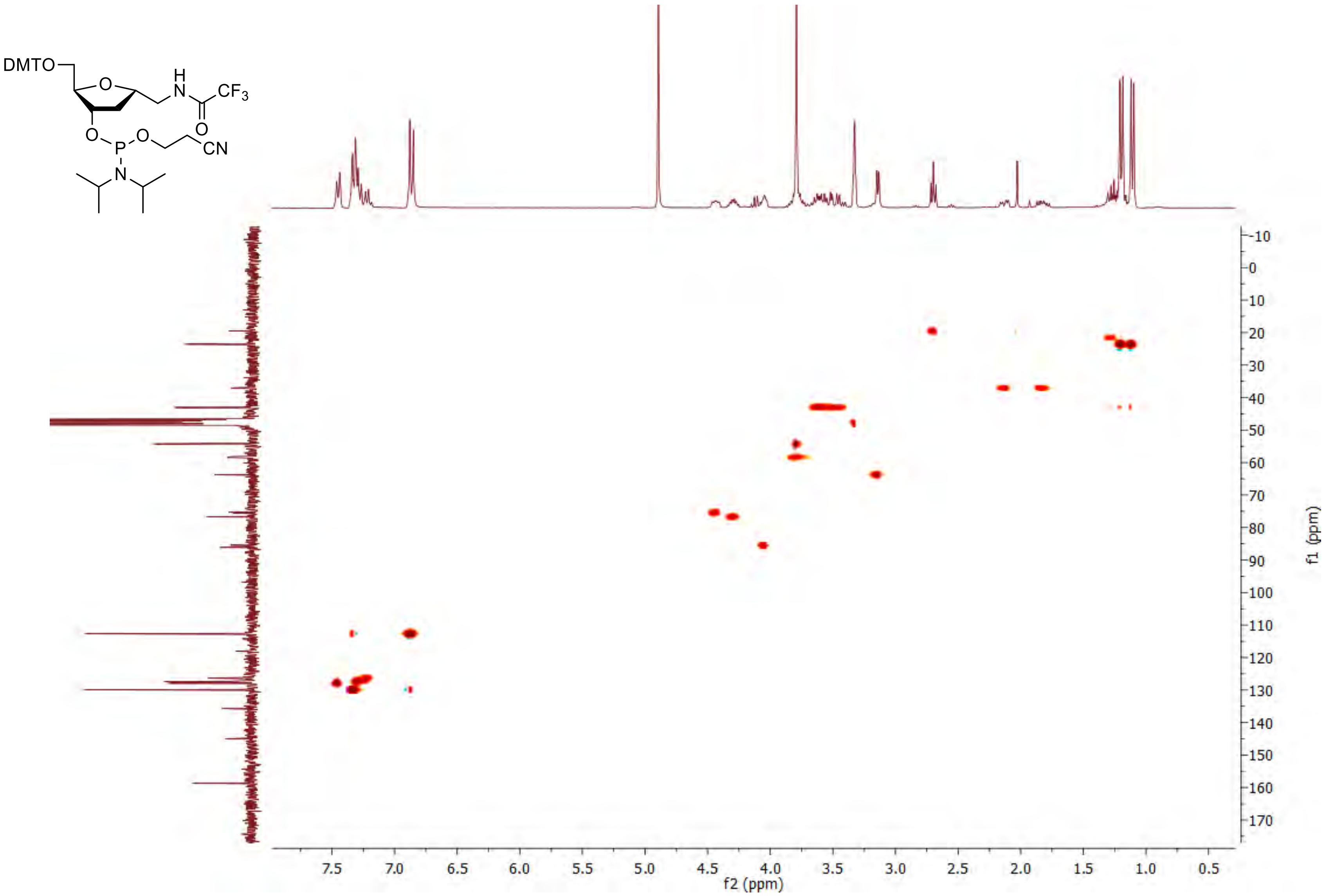

1,2-Dideoxy-5-*O*-(4,4'-dimethoxytrityl)-1α-[*N*-(trifluoroacetyl)aminomethyl]-D-*erythro*-pentofuranosyl-3-*O*-(2-cyanoethyl-*N*,*N*-diisopropyl)phosphoramidite (5α-B)

HMBC NMR (MeOH-d_4)

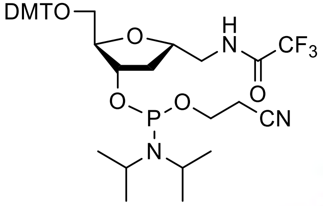

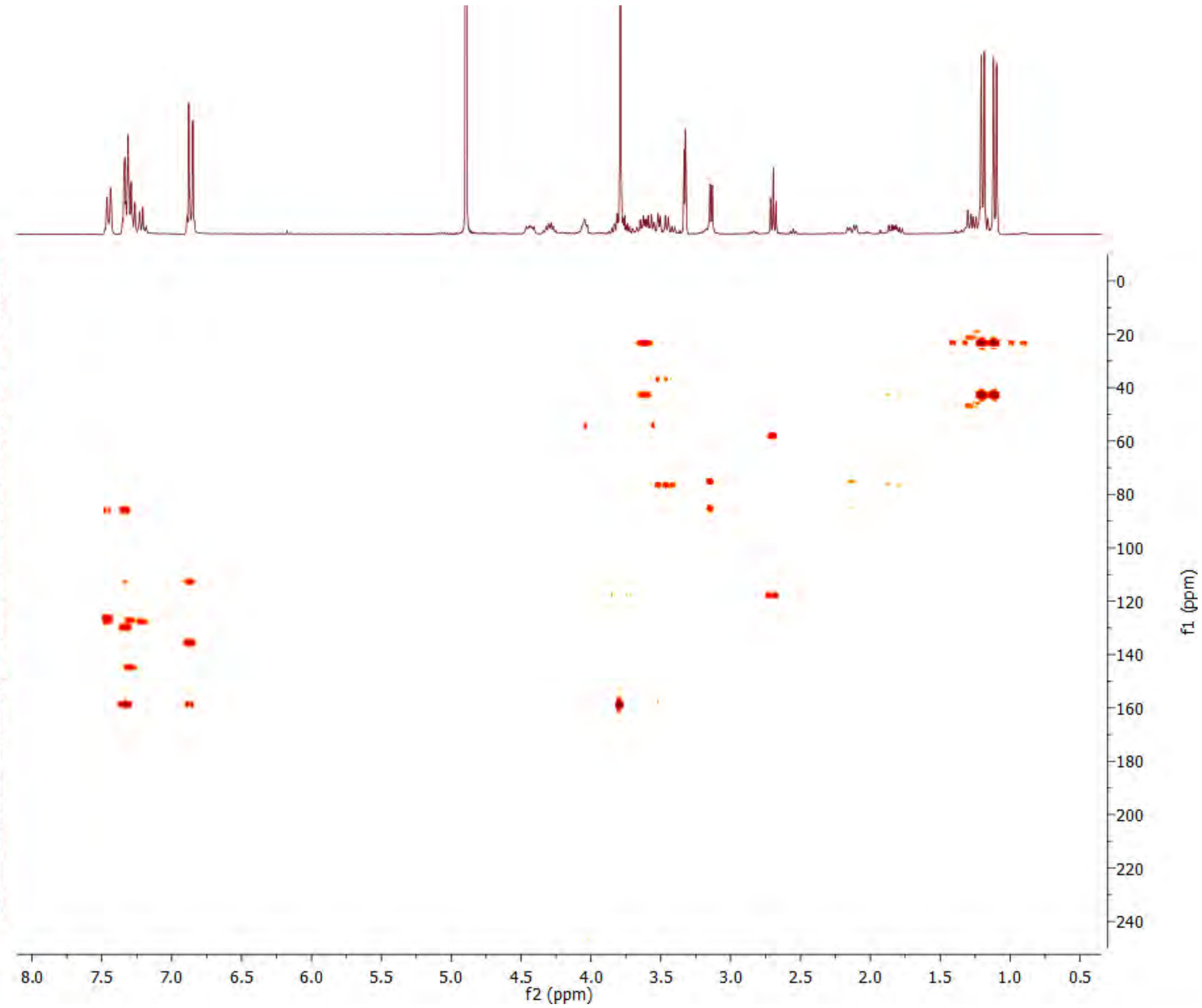

1,2-Dideoxy-5-*O*-(4,4'-dimethoxytrityl)-1α-[*N*-(trifluoroacetyl)aminomethyl]-D-*erythro*-pentofuranosyl-3-*O*-(2-cyanoethyl-*N*,*N*-diisopropyl)phosphoramidite (5α-B)

^{31}P NMR (121.5 MHz , MeOH-d_4)

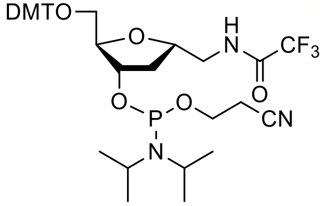

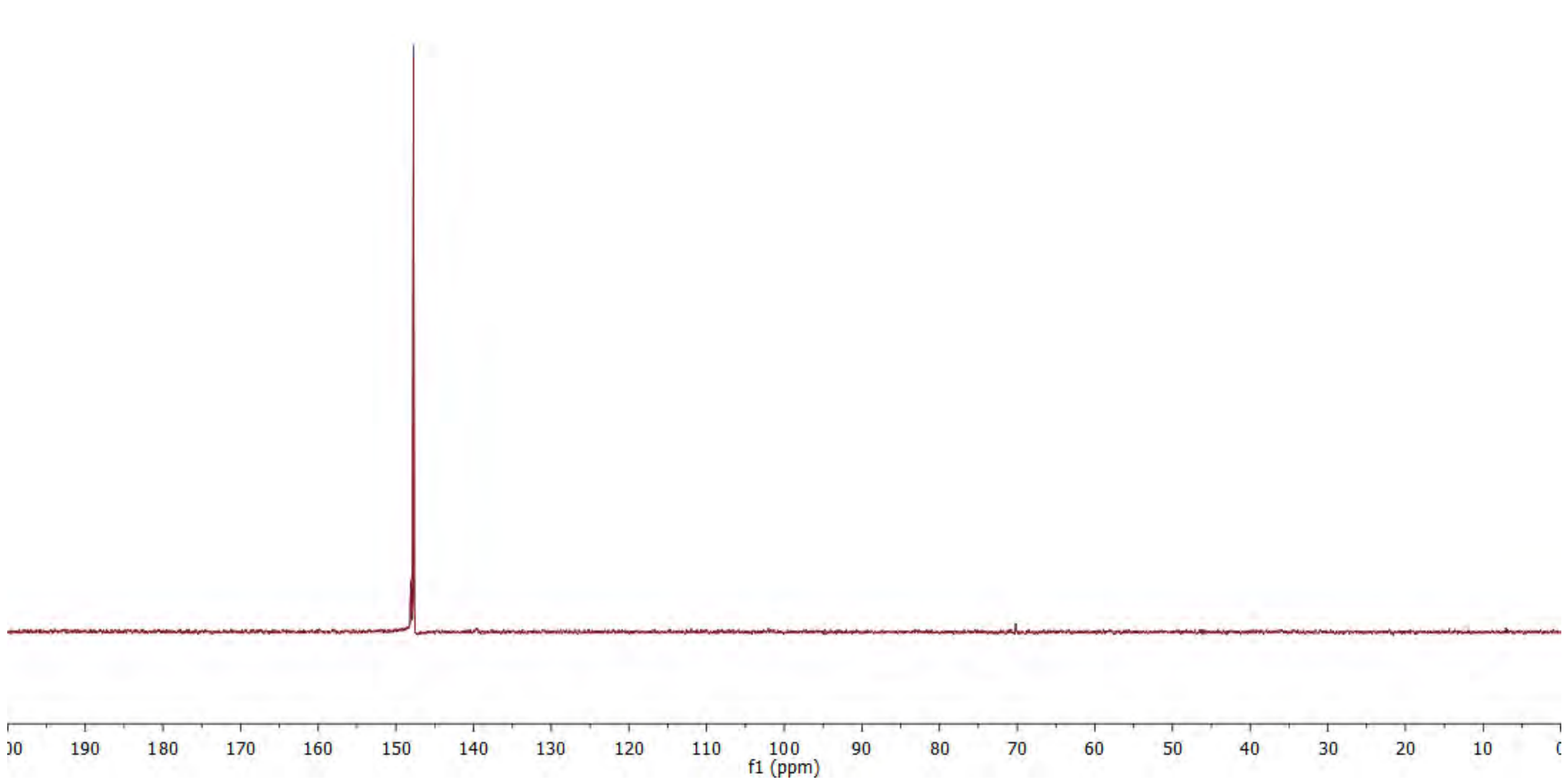

1,2-Dideoxy-5-*O*-(4,4'-dimethoxytrityl)-1β-[*N*-(trifluoroacetyl)aminomethyl]-D-*erythro*-pentofuranosyl-3-*O*-(2-cyanoethyl-*N*,*N*-diisopropyl)phosphoramidite (5β-A)

^{1}H NMR (300.13 MHz, MeOH-d_4)

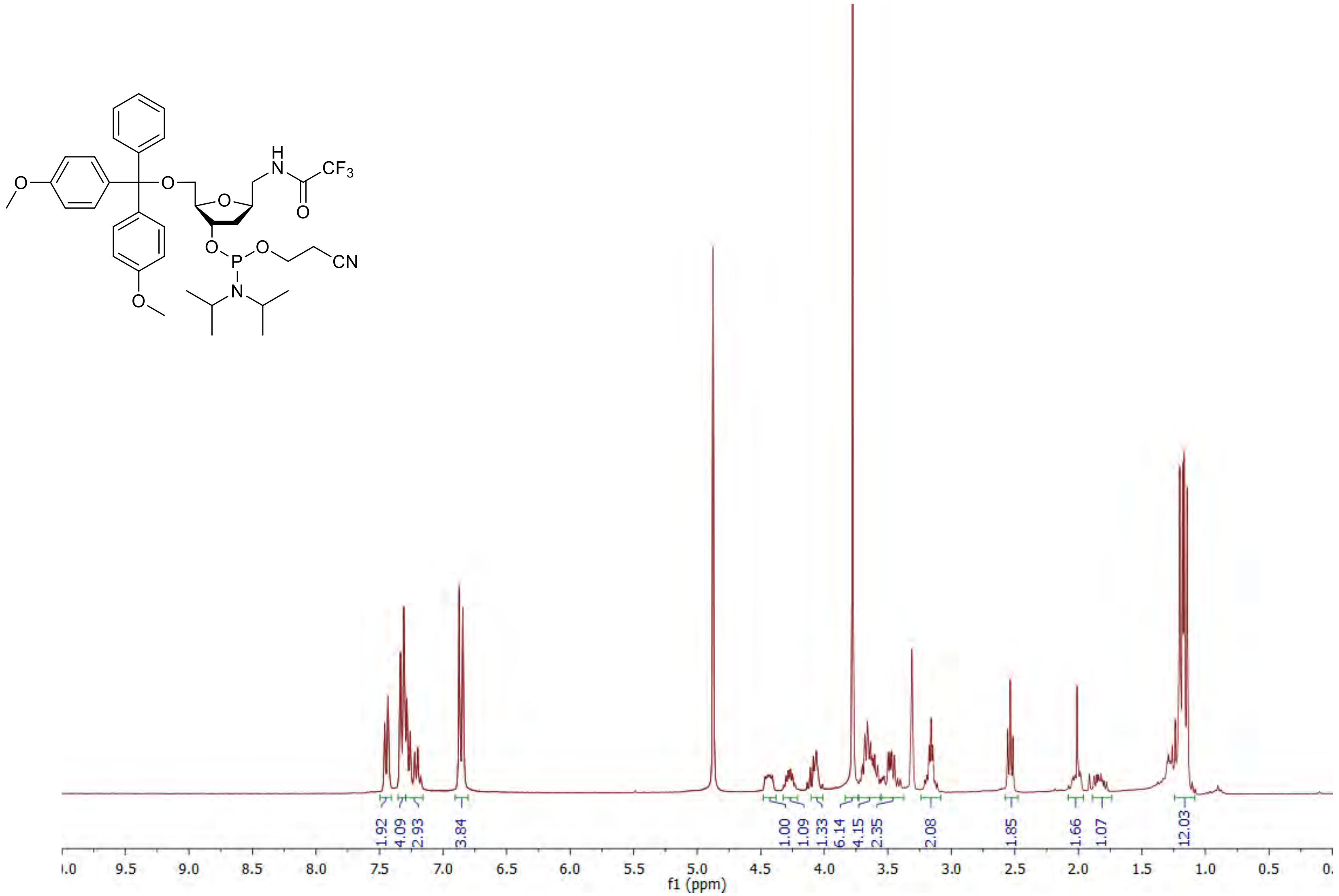

1,2-Dideoxy-5-*O*-(4,4'-dimethoxytrityl)-1β-[*N*-(trifluoroacetyl)aminomethyl]-D-*erythro*-pentofuranosyl-3-*O*-(2-cyanoethyl-*N*,*N*-diisopropyl)phosphoramidite (5β-A)

^{13}C NMR (75.5 MHz, MeOH-d_4)

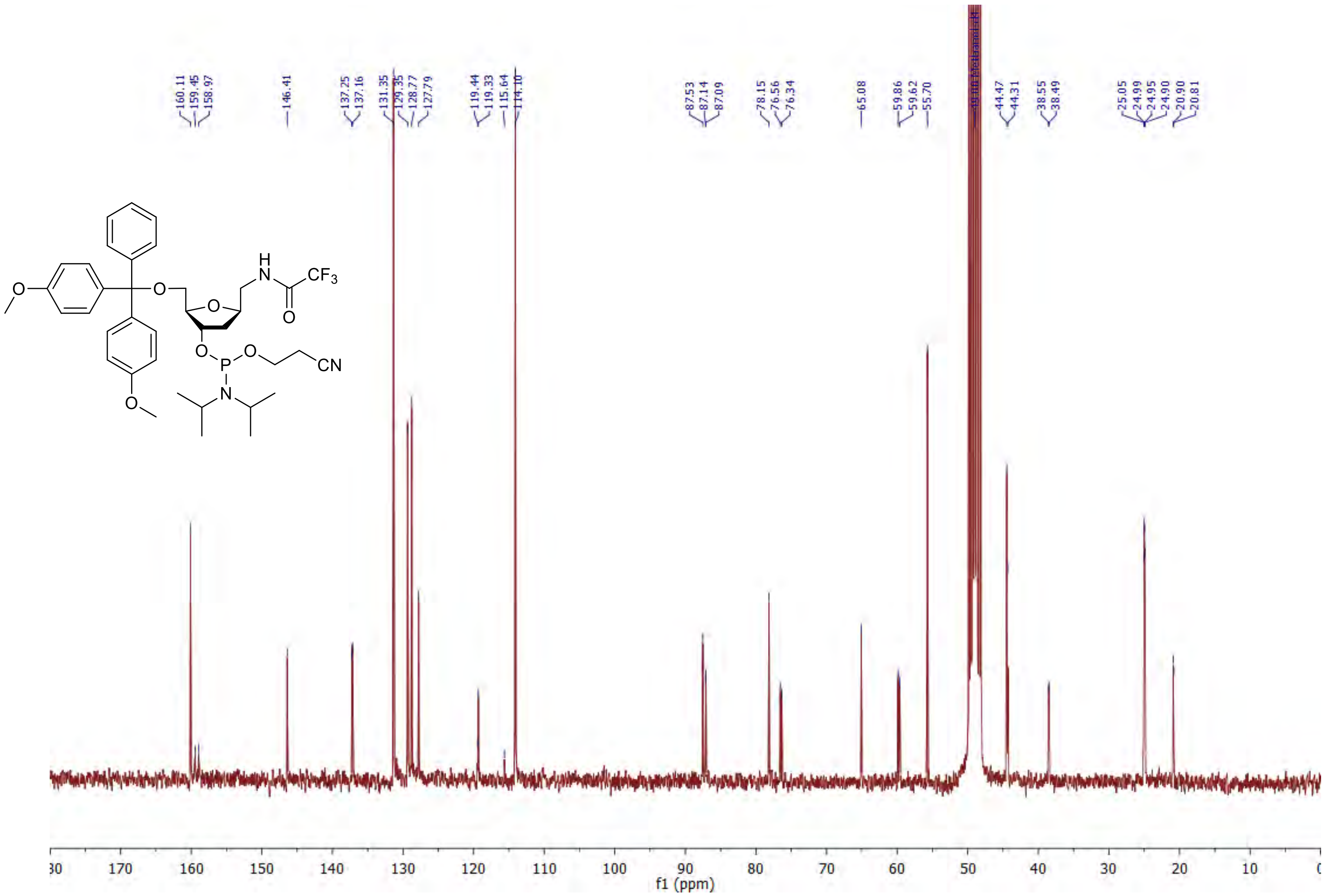

1,2-Dideoxy-5-*O*-(4,4’-dimethoxytrityl)-1β-[*N*-(trifluoroacetyl)aminomethyl]-D-*erythro*-pentofuranosyl-3-*O*-(2-cyanoethyl-*N*,*N*-diisopropyl)phosphoramidite (5β-A)

DEPT 135 NMR (75.5 MHz, MeOH-d_4)

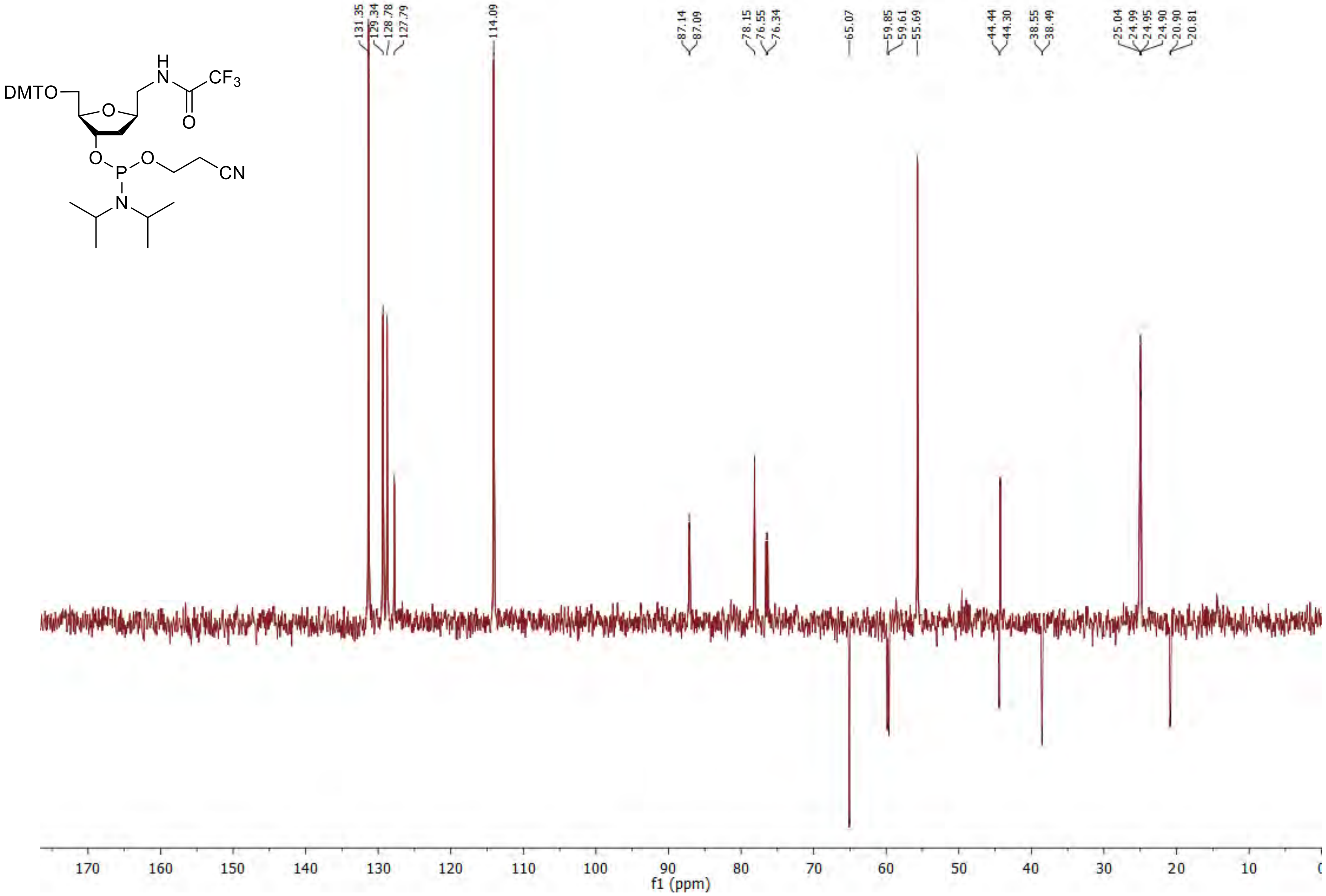

1,2-Dideoxy-5-*O*-(4,4'-dimethoxytrityl)-1β-[*N*-(trifluoroacetyl)aminomethyl]-D-*erythro*-pentofuranosyl-3-*O*-(2-cyanoethyl-*N*,*N*-diisopropyl)phosphoramidite (5β-A)

COSY NMR (MeOH-d_4)

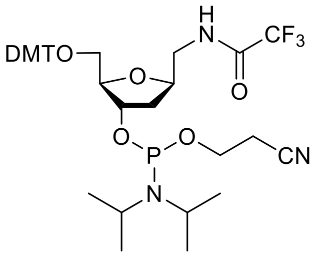

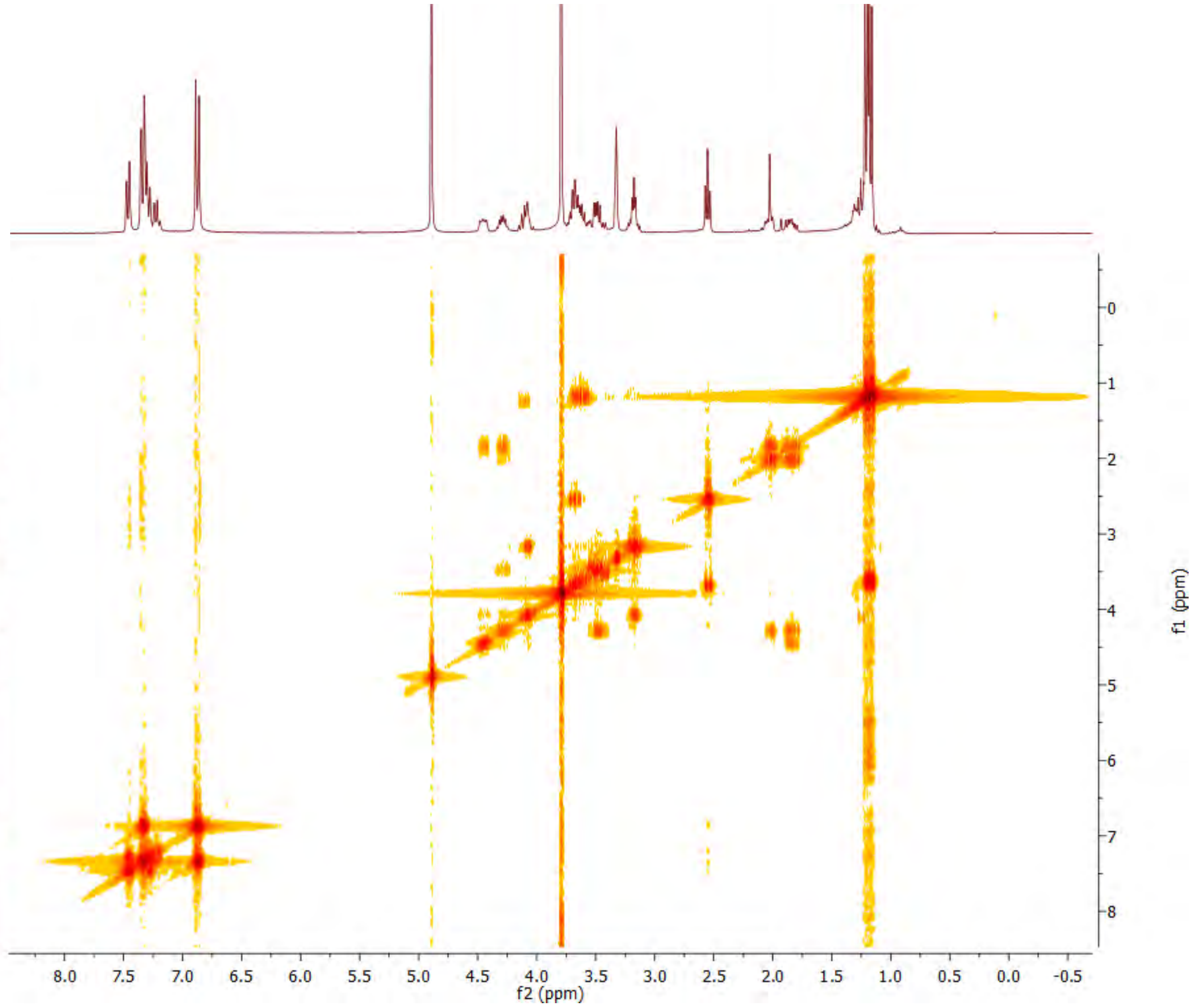

1,2-Dideoxy-5-*O*-(4,4'-dimethoxytrityl)-1β-[*N*-(trifluoroacetyl)aminomethyl]-D-*erythro*-pentofuranosyl-3-*O*-(2-cyanoethyl-*N*,*N*-diisopropyl)phosphoramidite (5β-A)

HSQC NMR (MeOH-d_4)

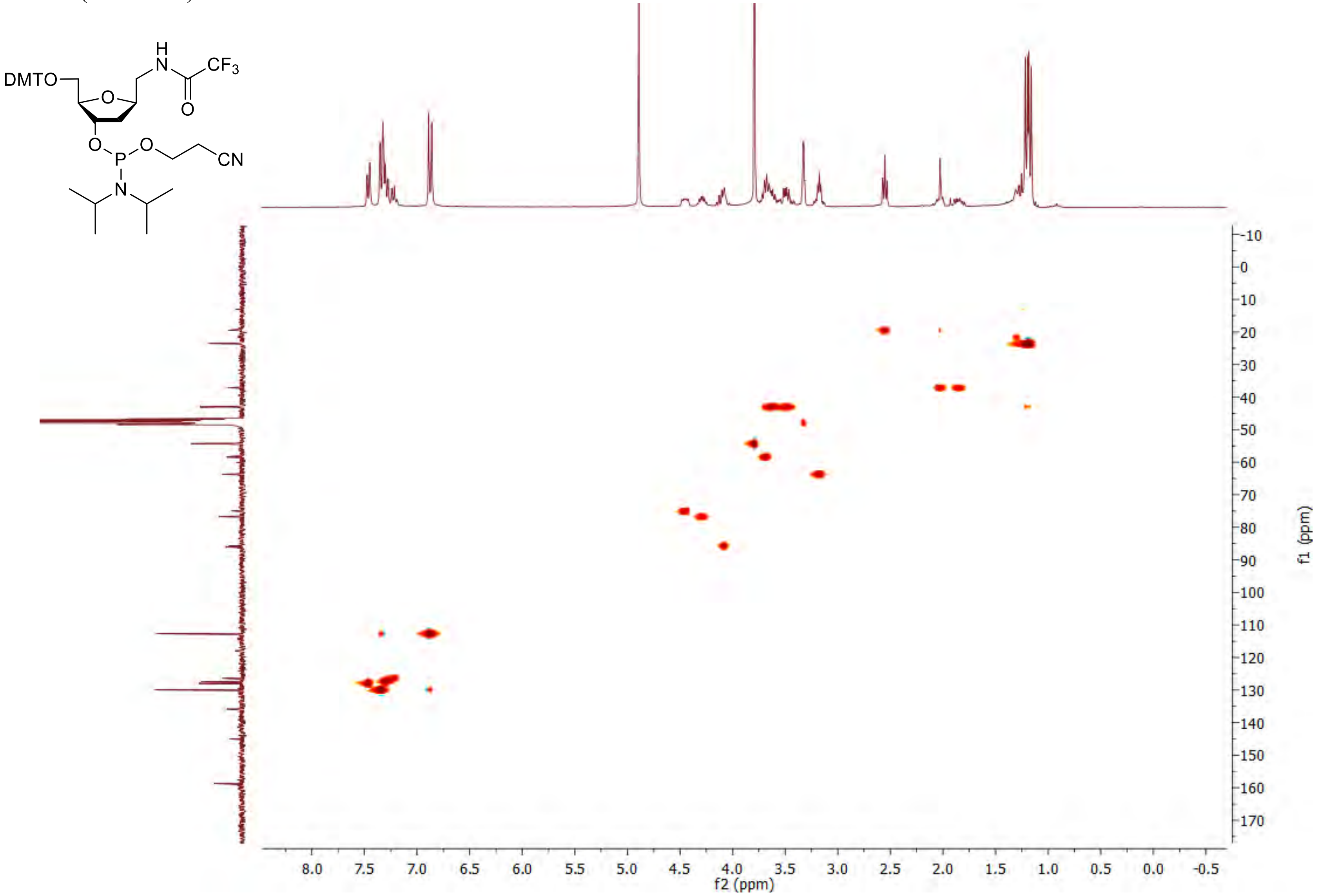

1,2-Dideoxy-5-*O*-(4,4'-dimethoxytrityl)-1β-[*N*-(trifluoroacetyl)aminomethyl]-D-*erythro*-pentofuranosyl-3-*O*-(2-cyanoethyl-*N,N*-diisopropyl)phosphoramidite (5β-A)

HMBC NMR (MeOH-d_4)

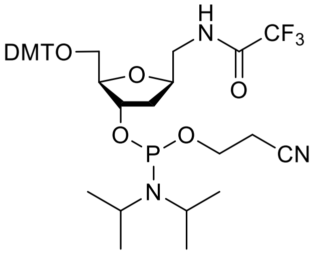

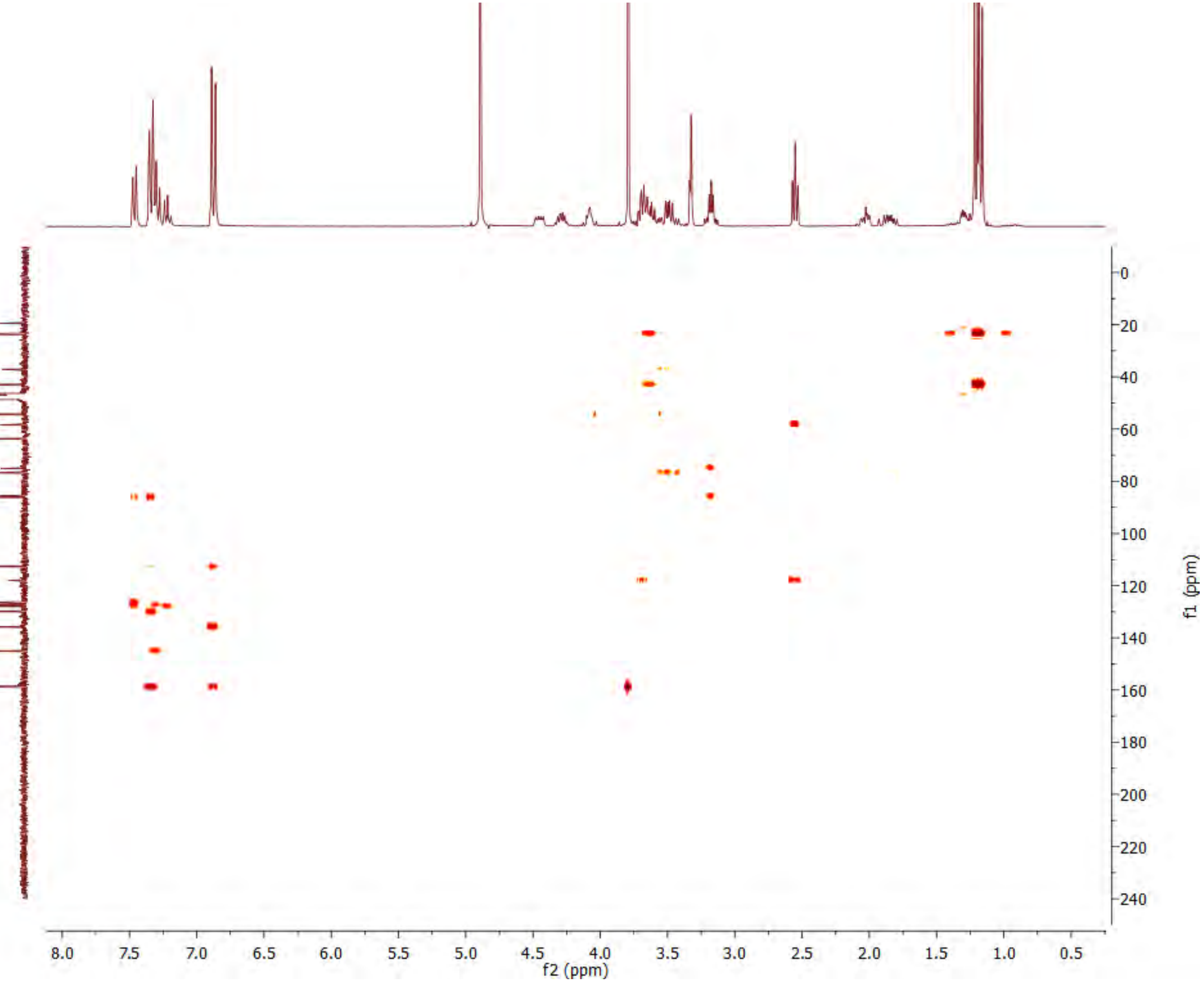

1,2-Dideoxy-5-*O*-(4,4'-dimethoxytrityl)-1β-[*N*-(trifluoroacetyl)aminomethyl]-D-*erythro*-pentofuranosyl-3-*O*-(2-cyanoethyl-*N,N*-diisopropyl)phosphoramidite (5β-A)

^{31}P NMR (121.5 MHz, MeOH-d_4)

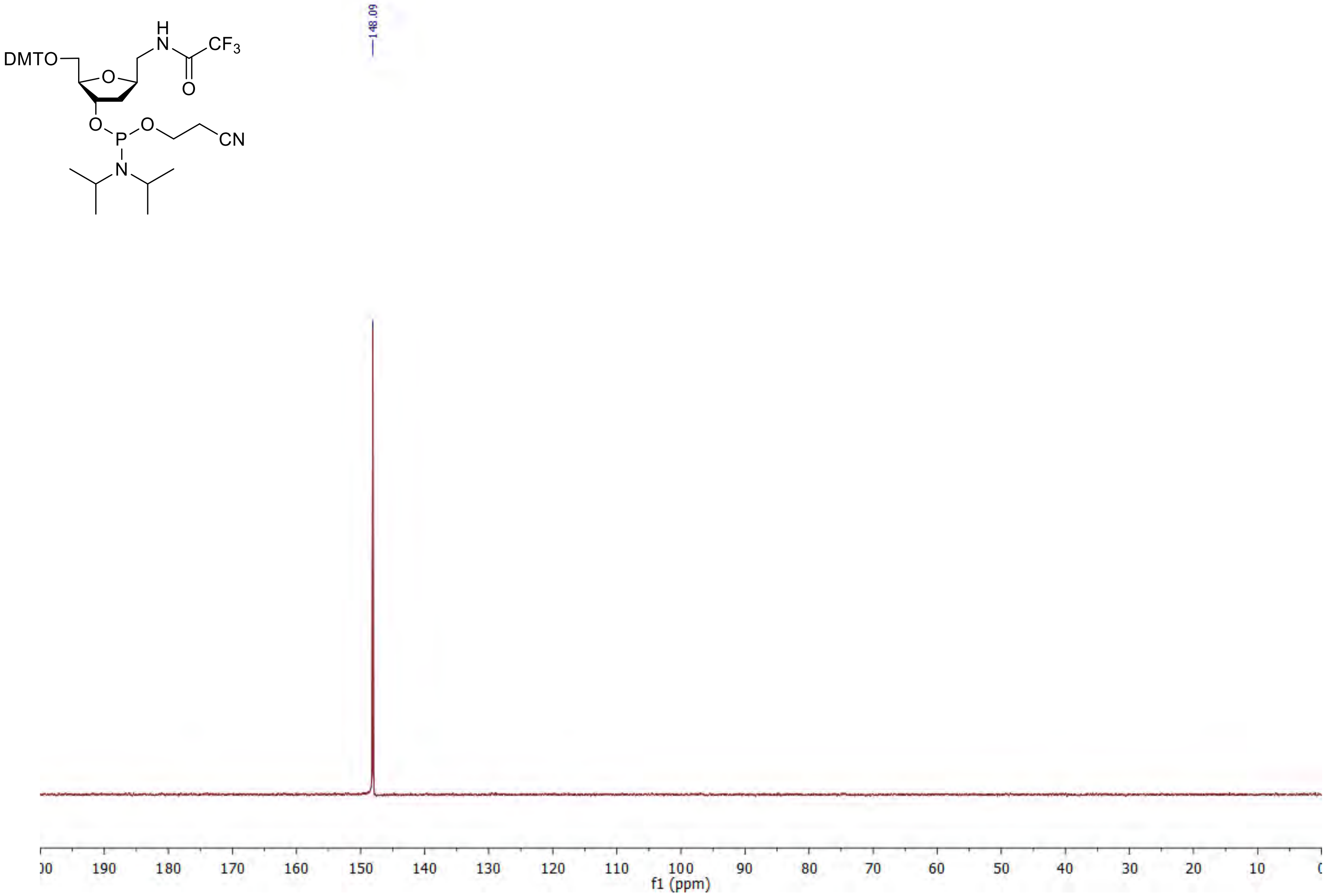

1,2-Dideoxy-5-*O*-(4,4'-dimethoxytrityl)-1β-[*N*-(trifluoroacetyl)aminomethyl]-D-*erythro*-pentofuranosyl-3-*O*-(2-cyanoethyl-*N,N*-diisopropyl)phosphoramidite (5β-B)

^{1}H NMR (300.13 MHz, MeOH-d_4)

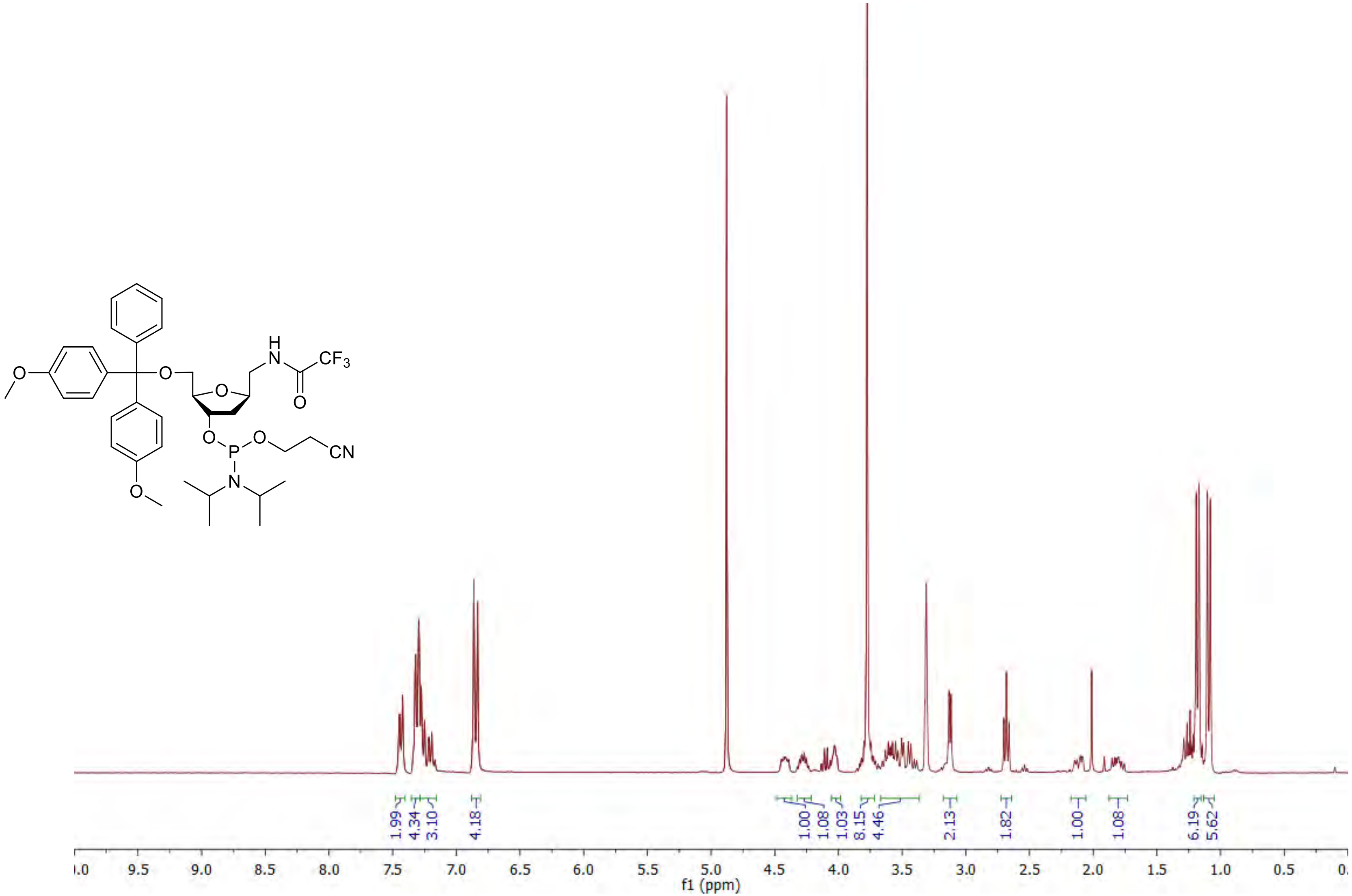

1,2-Dideoxy-5-*O*-(4,4'-dimethoxytrityl)-1β-[*N*-(trifluoroacetyl)aminomethyl]-D-*erythro*-pentofuranosyl-3-*O*-(2-cyanoethyl-*N*,*N*-diisopropyl)phosphoramidite (5β-B)

^{13}C NMR (75.5 MHz, MeOH-d_4)

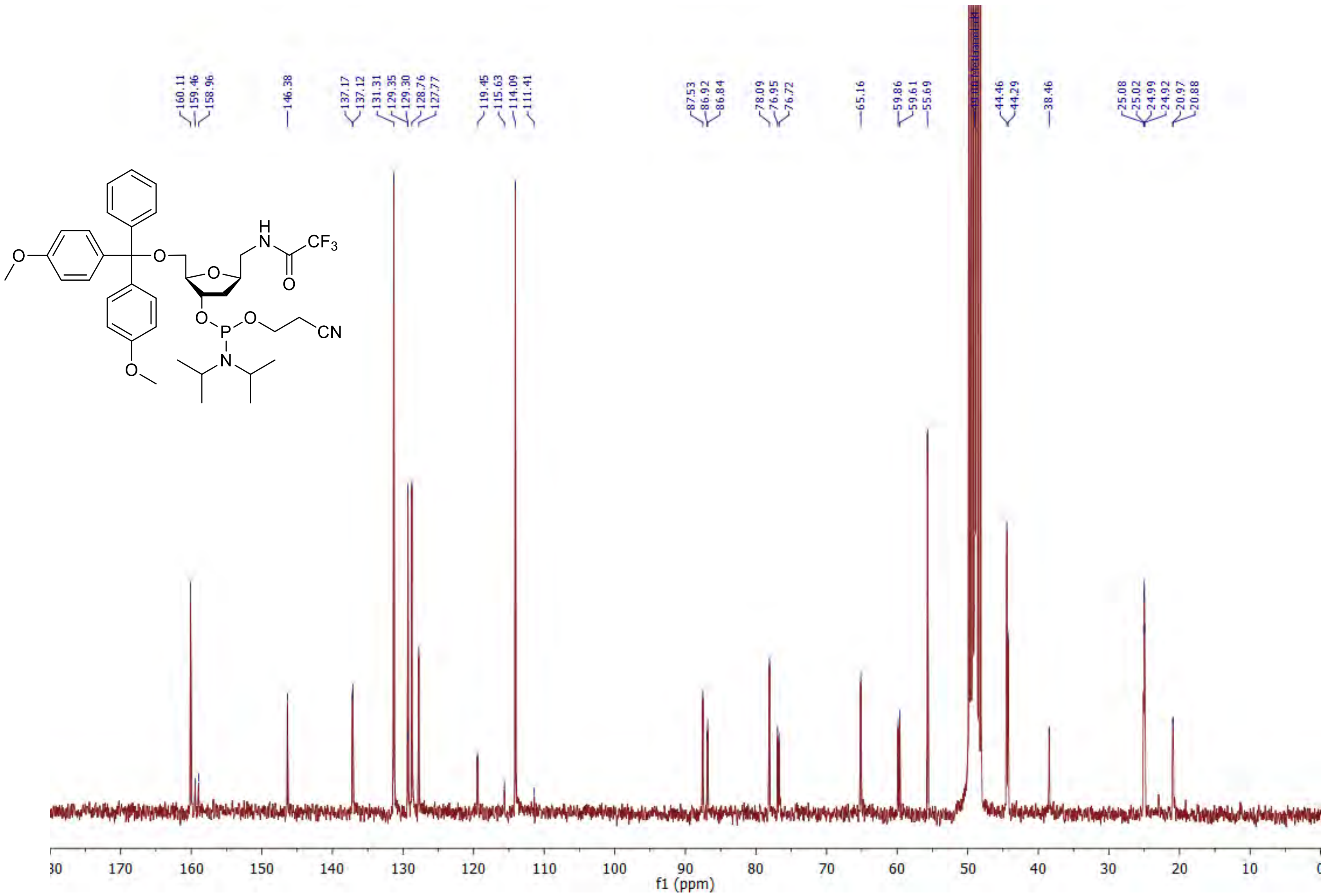

1,2-Dideoxy-5-*O*-(4,4'-dimethoxytrityl)-1β-[*N*-(trifluoroacetyl)aminomethyl]-D-*erythro*-pentofuranosyl-3-*O*-(2-cyanoethyl-*N,N*-diisopropyl)phosphoramidite (5β-B)

DEPT 135 NMR (75.5 MHz, MeOH-d_4)

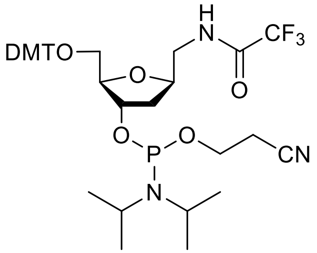

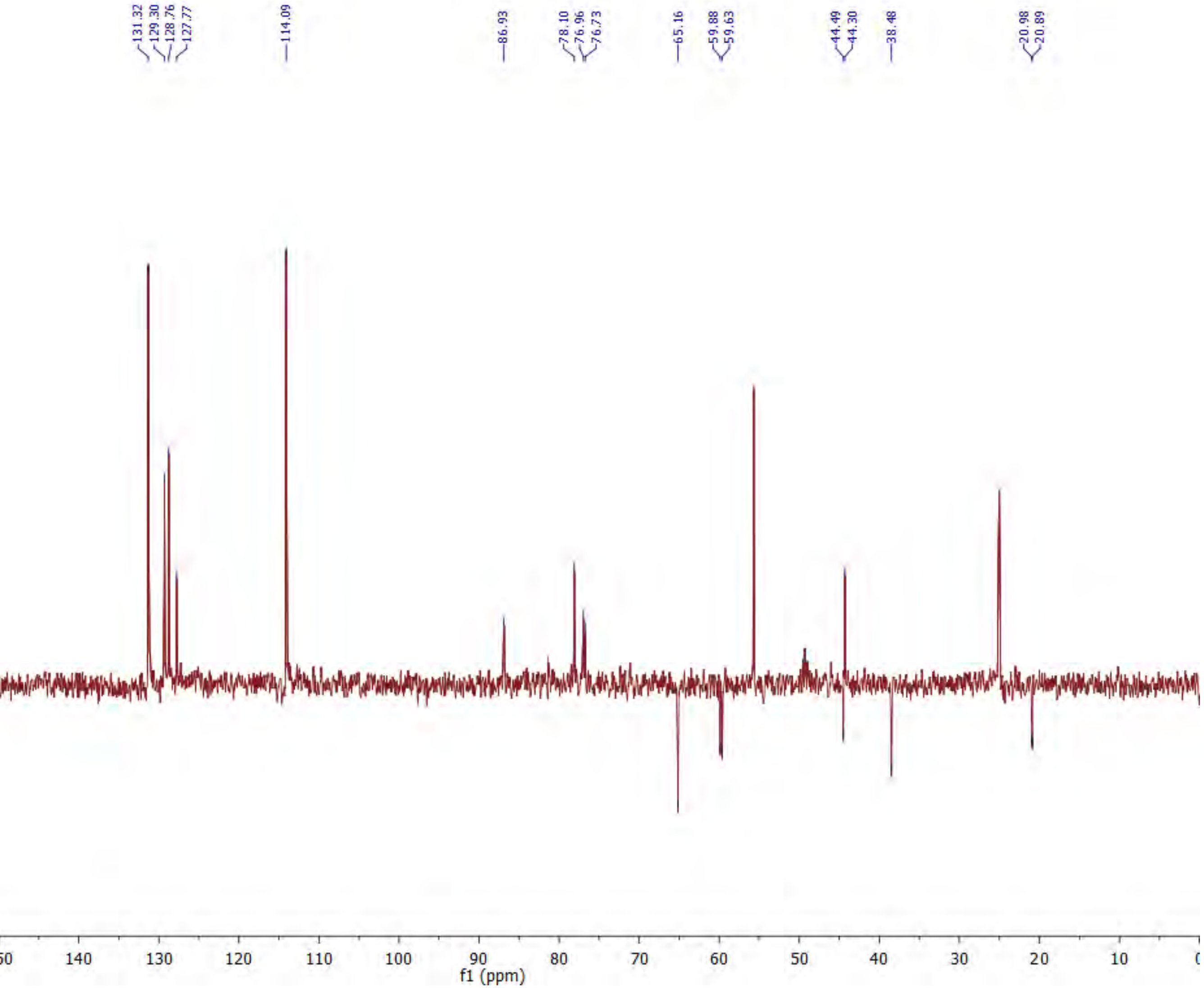

1,2-Dideoxy-5-*O*-(4,4'-dimethoxytrityl)-1β-[*N*-(trifluoroacetyl)aminomethyl]-D-*erythro*-pentofuranosyl-3-*O*-(2-cyanoethyl-*N*,*N*-diisopropyl)phosphoramidite (5β-B)

COSY NMR (MeOH-d_4)

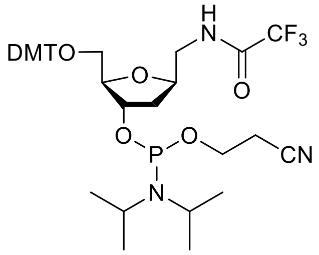

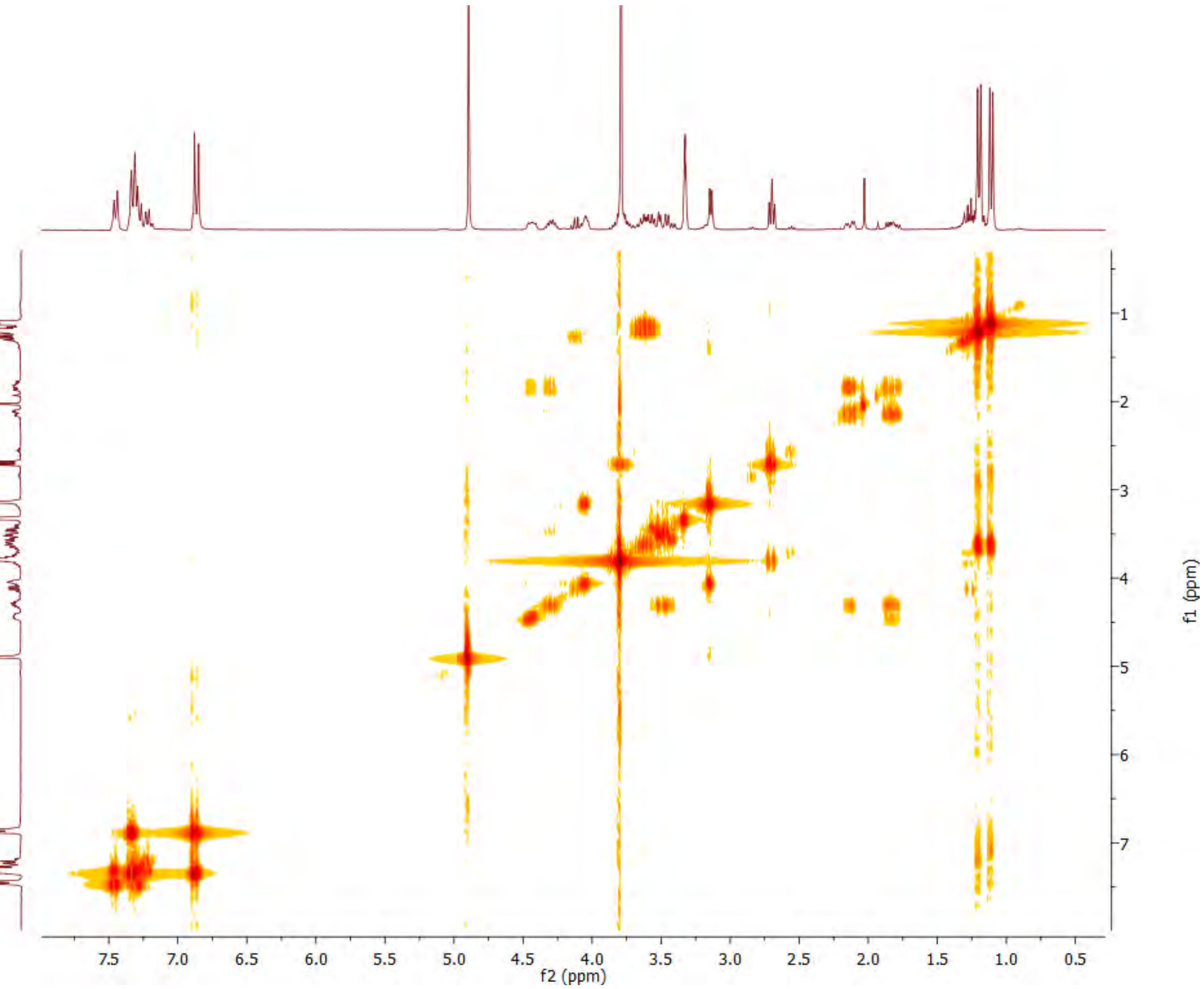

1,2-Dideoxy-5-*O*-(4,4'-dimethoxytrityl)-1β-[*N*-(trifluoroacetyl)aminomethyl]-ᴅ-*erythro*-pentofuranosyl-3-*O*-(2-cyanoethyl-*N*,*N*-diisopropyl)phosphoramidite (5β-B)

HSQC NMR (MeOH-d_4)

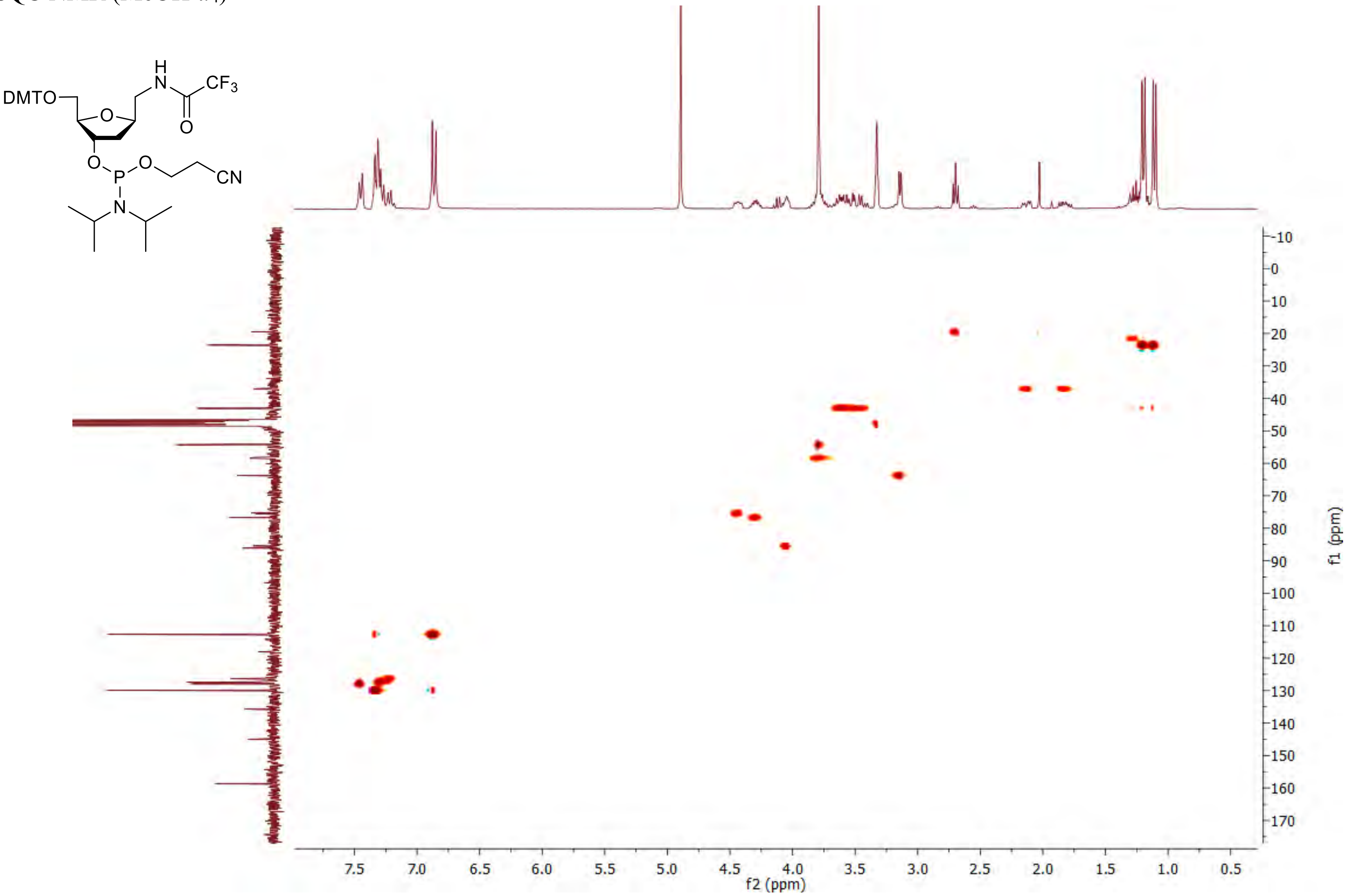

1,2-Dideoxy-5-*O*-(4,4'-dimethoxytrityl)-1β-[*N*-(trifluoroacetyl)aminomethyl]-D-*erythro*-pentofuranosyl-3-*O*-(2-cyanoethyl-*N*,*N*-diisopropyl)phosphoramidite (5β-B)

HMBC NMR (MeOH-d_4)

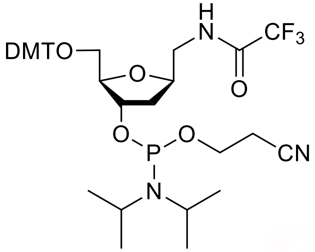

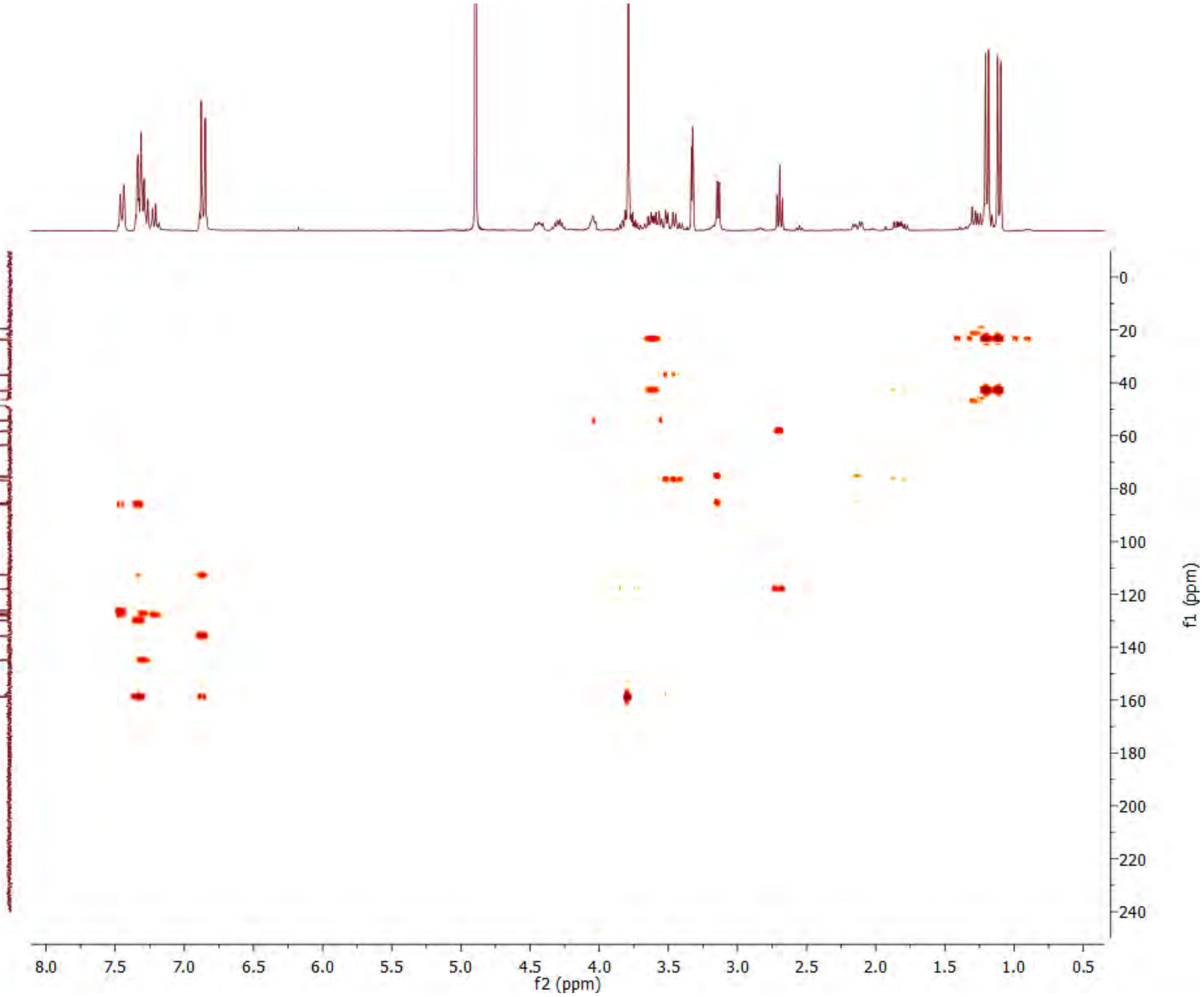

1,2-Dideoxy-5-*O*-(4,4'-dimethoxytrityl)-1β-[*N*-(trifluoroacetyl)aminomethyl]-ᴅ-*erythro*-pentofuranosyl-3-*O*-(2-cyanoethyl-*N,N*-diisopropyl)phosphoramidite (5β-B)

^{31}P NMR (121.5 MHz, MeOH-d_4)

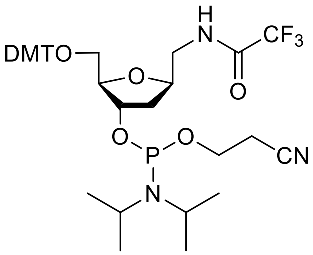

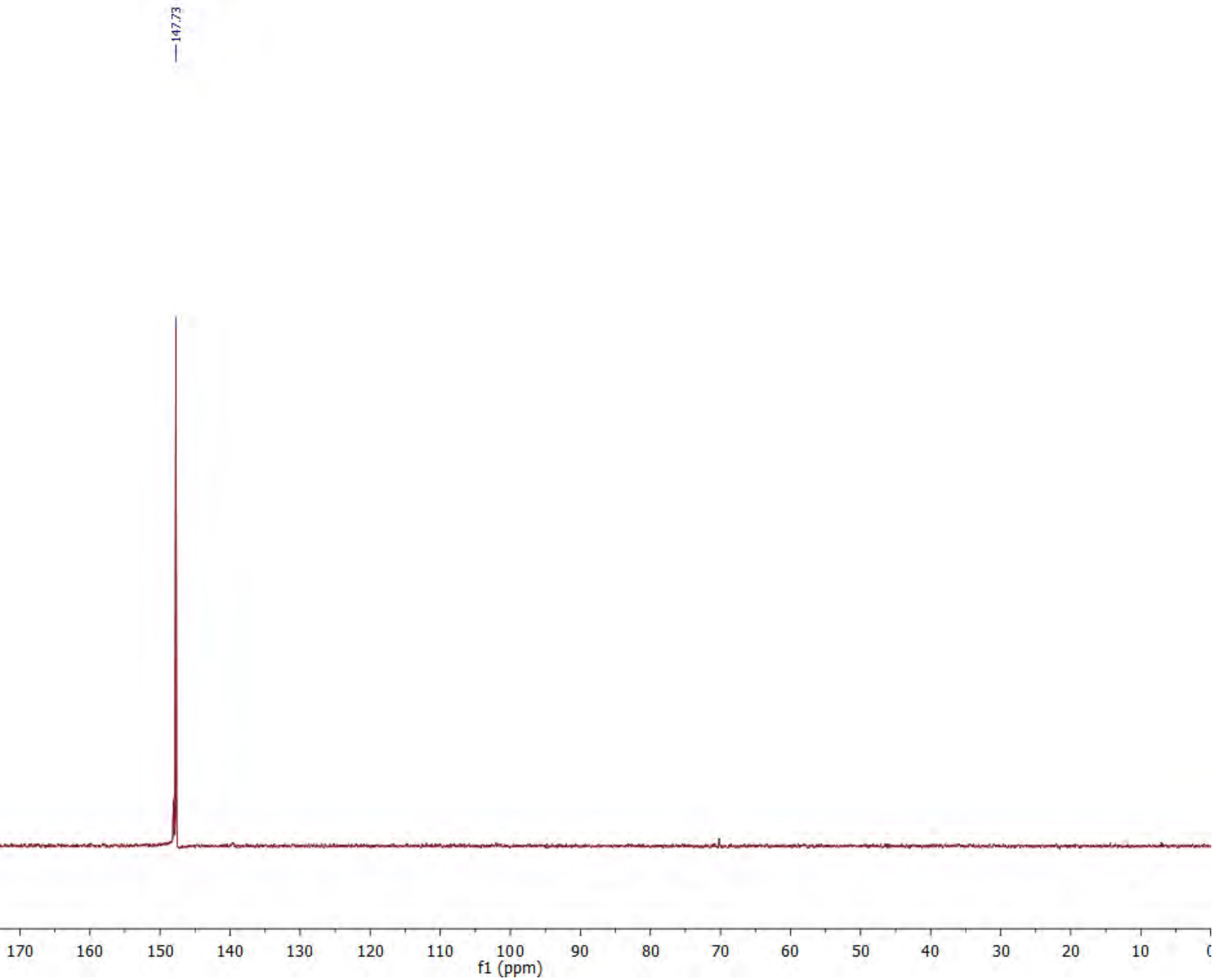

1,2-Dideoxy-1α-[(1-(2-nitrophenyl)ethoxy)carbonylaminomethyl]-D-*erythro*-pentofuranose (6α)

^{1}H NMR (300.13 MHz, MeOH-d_4)

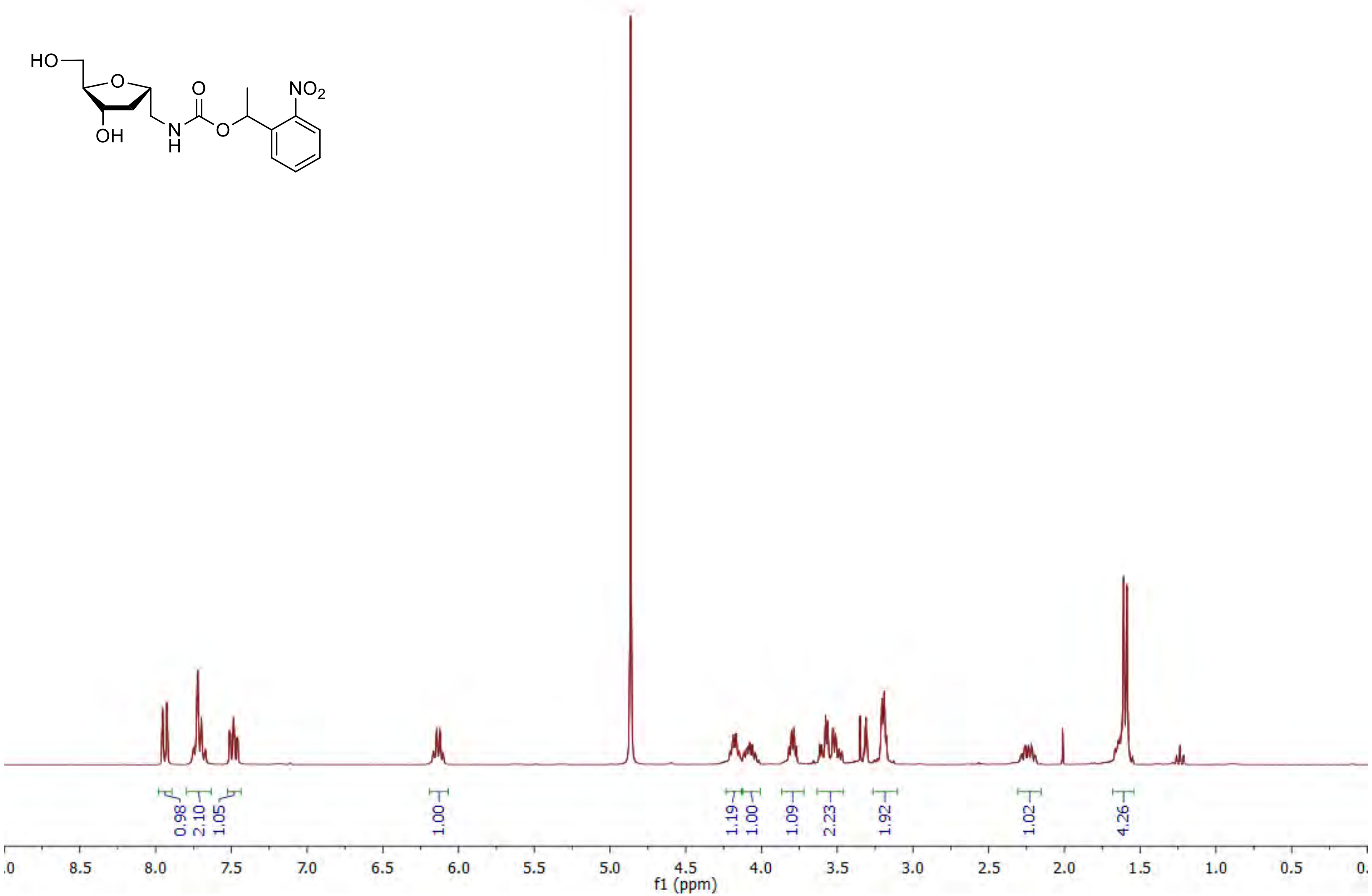

1,2-Dideoxy-1α-[(1-(2-nitrophenyl)ethoxy)carbonylaminomethyl]-D-*erythro*-pentofuranose (6α)

^{13}C NMR (75.5 MHz, MeOH-d_4)

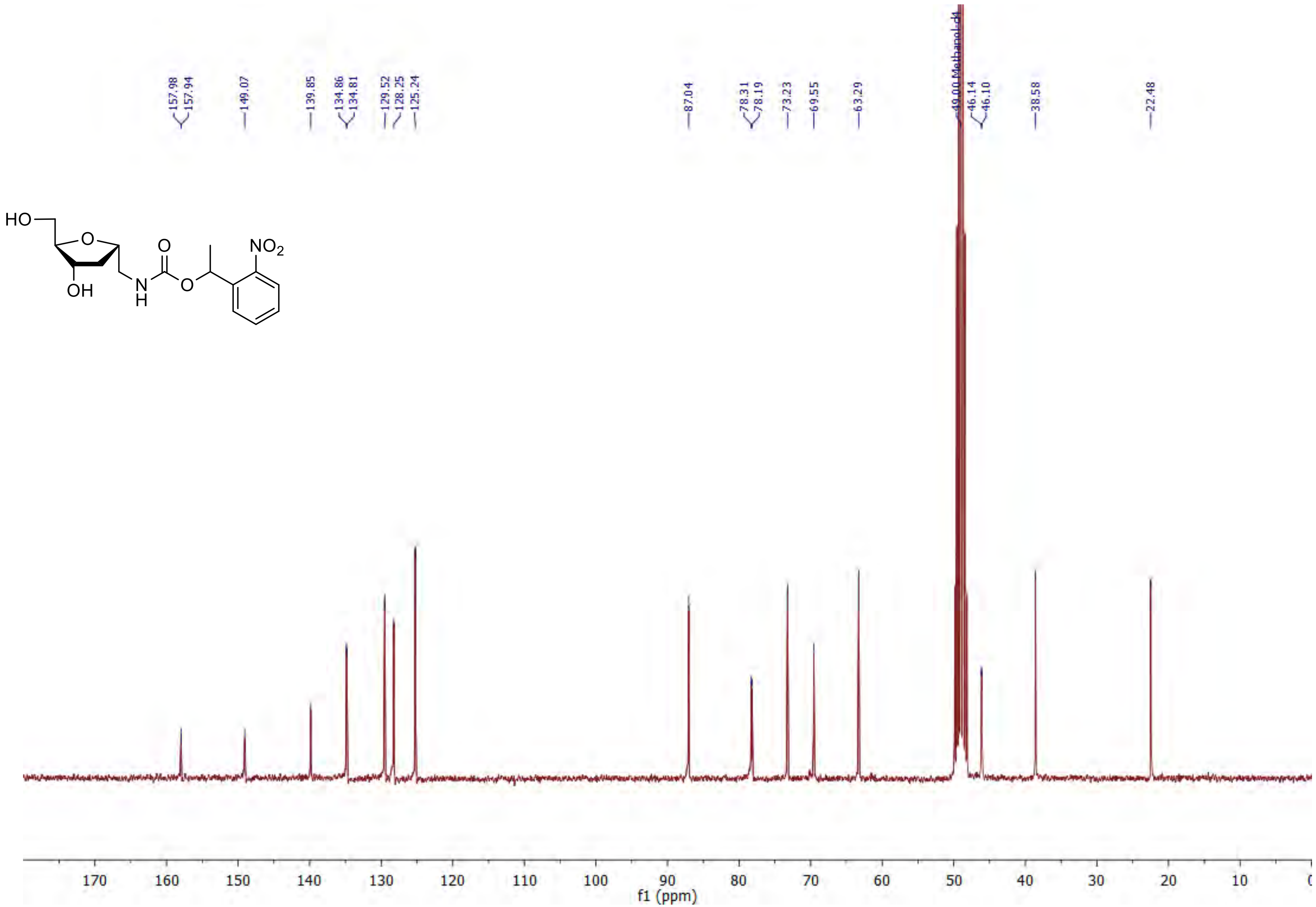

1,2-Dideoxy-1α-[(1-(2-nitrophenyl)ethoxy)carbonylaminomethyl]-D-*erythro*-pentofuranose (6α)

DEP135 NMR (75.5 MHz, MeOH-d_4)

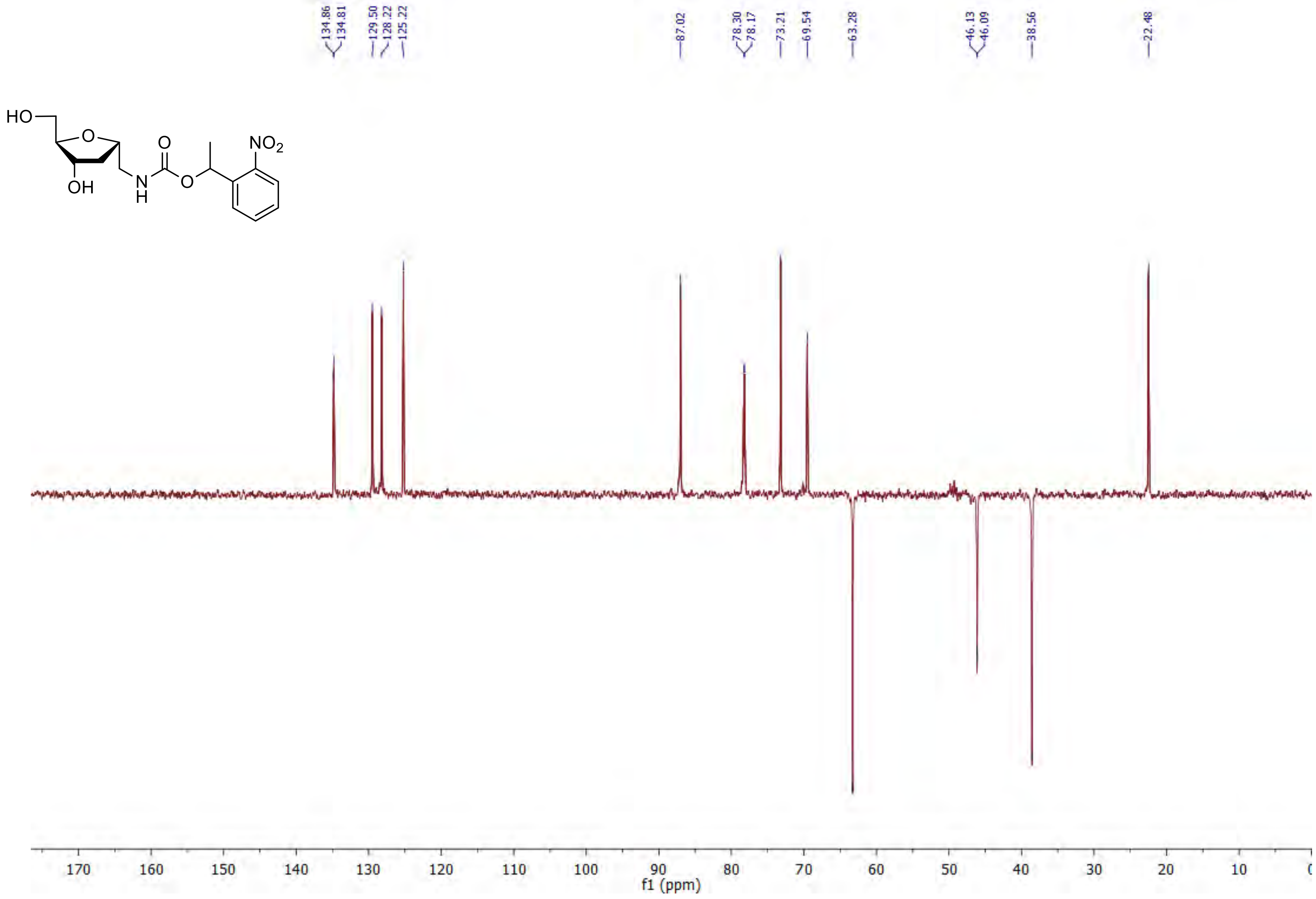

1,2-Dideoxy-1α-[(1-(2-nitrophenyl)ethoxy)carbonylaminomethyl]-D-*erythro*-pentofuranose (6α)

COSY NMR (MeOH-d_4)

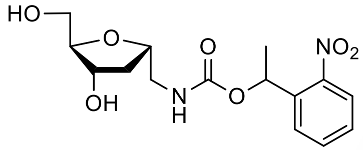

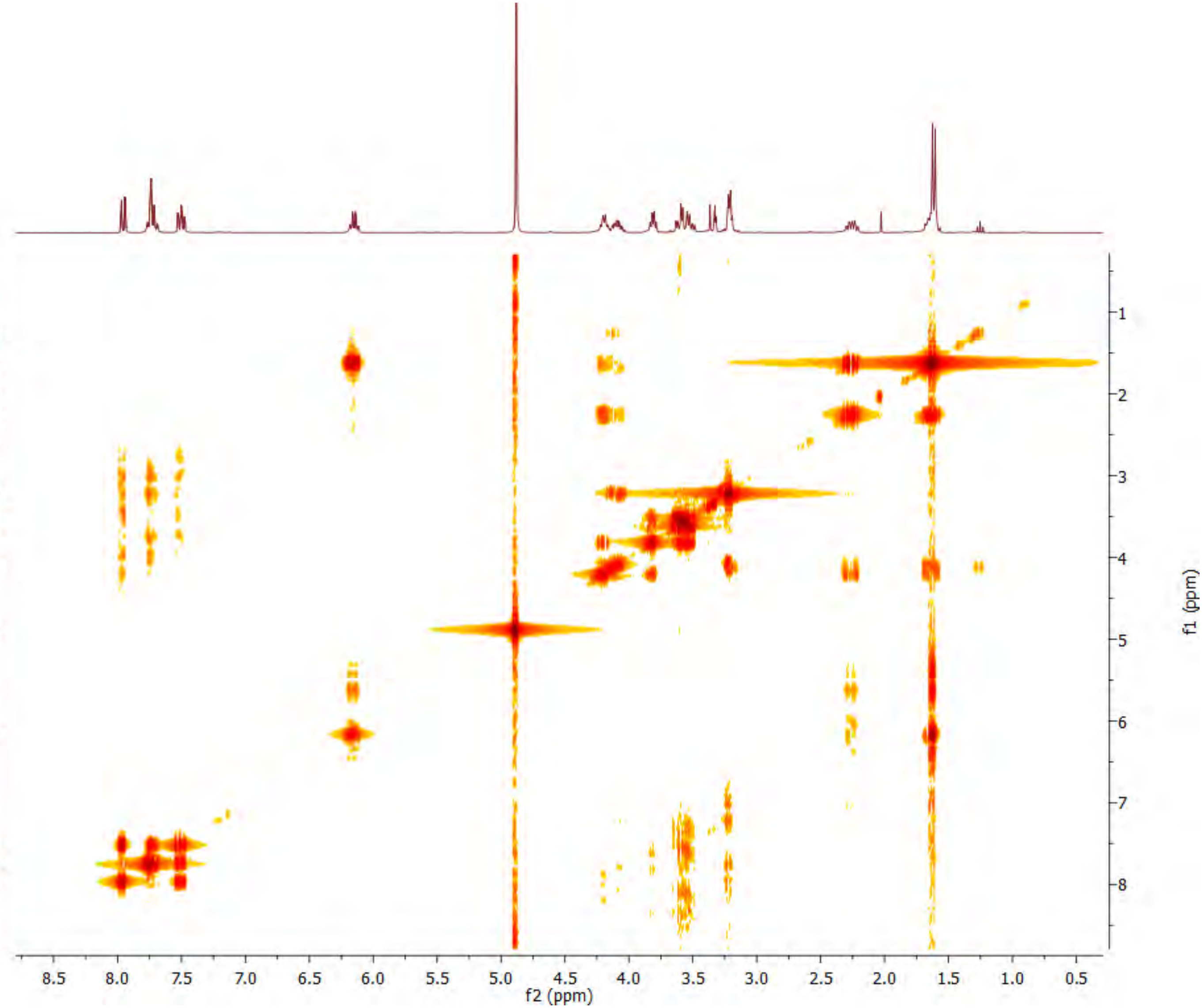

1,2-Dideoxy-1α-[(1-(2-nitrophenyl)ethoxy)carbonylaminomethyl]-D-*erythro*-pentofuranose (6α)

HSQC NMR (MeOH-d_4)

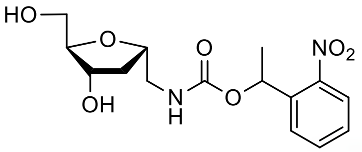

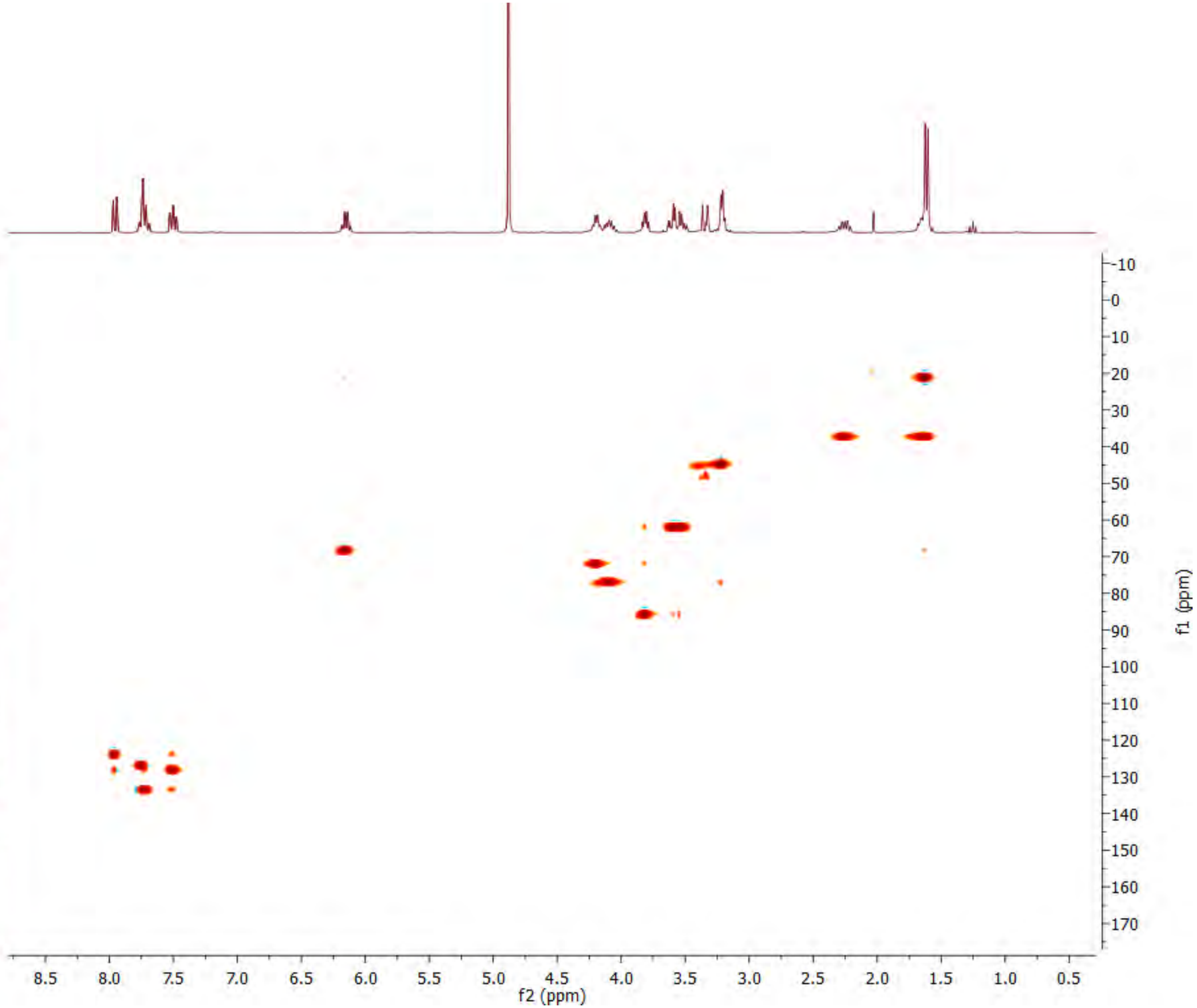

1,2-Dideoxy-1α-[(1-(2-nitrophenyl)ethoxy)carbonylaminomethyl]-D-*erythro*-pentofuranose (6α)

HMBC NMR (MeOH-d_4)

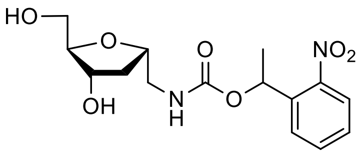

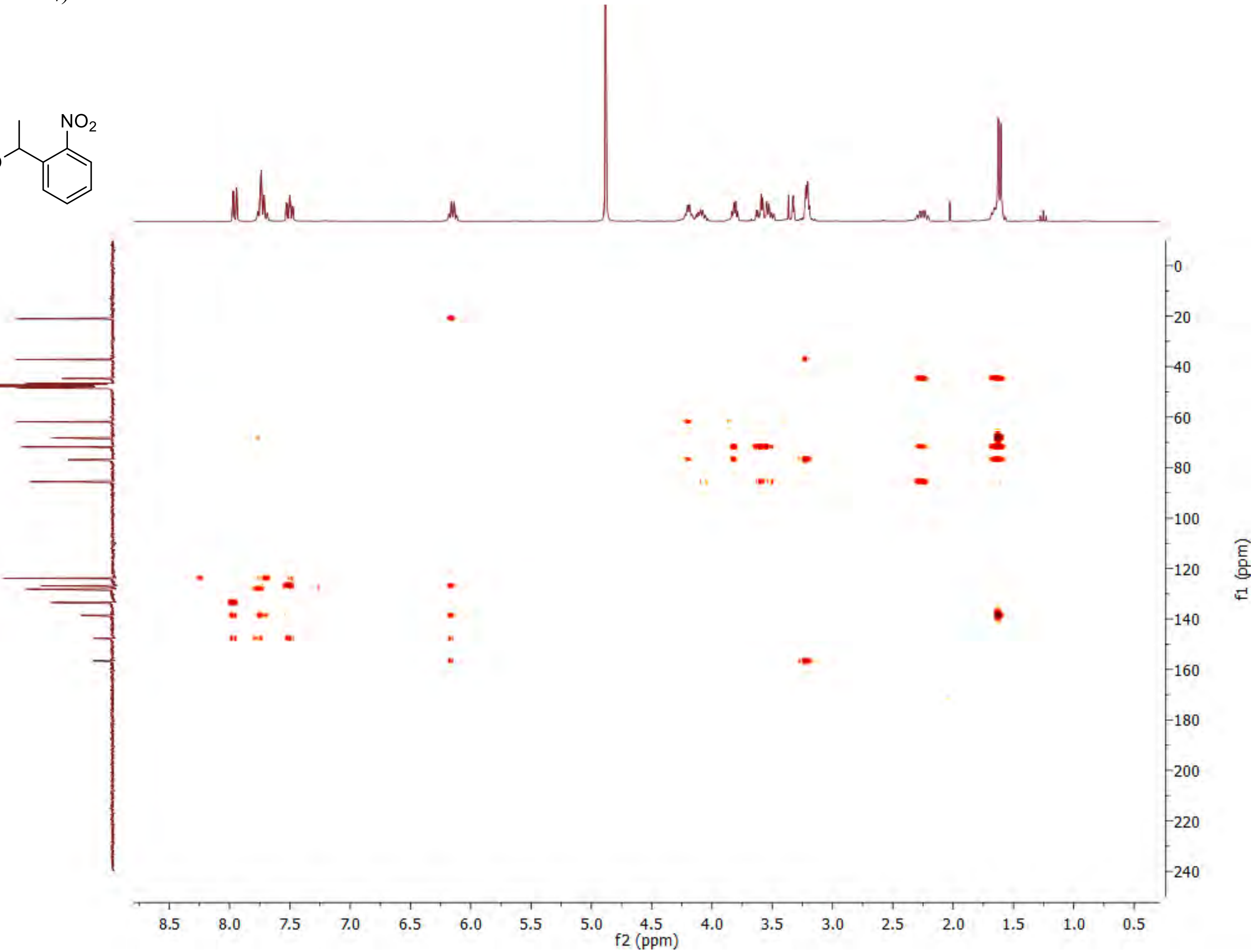

1,2-Dideoxy-1β-[(1-(2-nitrophenyl)ethoxy)carbonylaminomethyl]-D-*erythro*-pentofuranose (6β)

^{1}H NMR (300.13 MHz, MeOH-d_4)

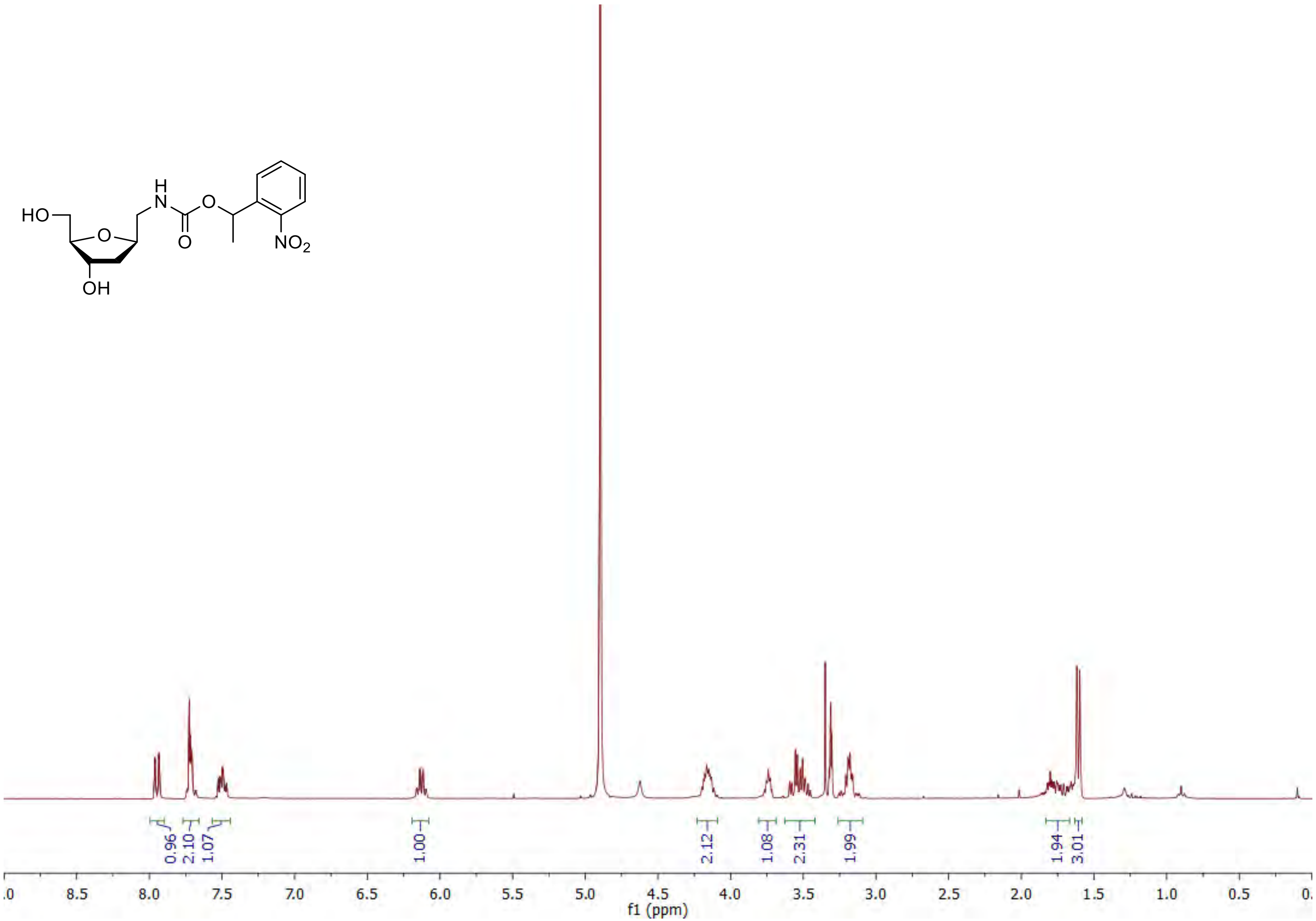

1,2-Dideoxy-1β-[(1-(2-nitrophenyl)ethoxy)carbonylaminomethyl]-D-*erythro*-pentofuranose (6β)

^{13}C NMR (75.5 MHz, MeOH-d_4)

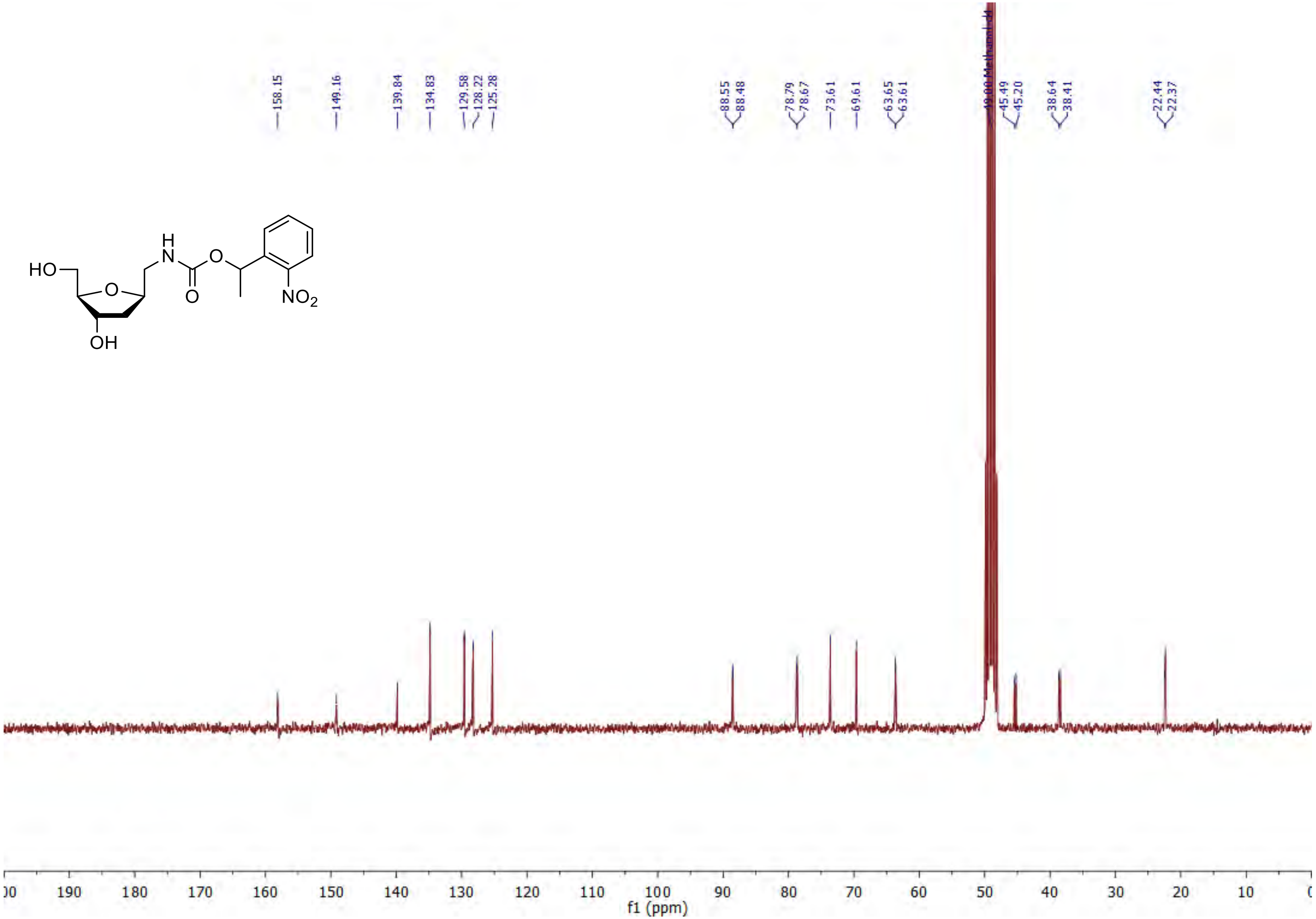

1,2-Dideoxy-1β-[(1-(2-nitrophenyl)ethoxy)carbonylaminomethyl]-D-*erythro*-pentofuranose (6β)

DEPT 135 NMR (75.5 MHz, MeOH-d_4)

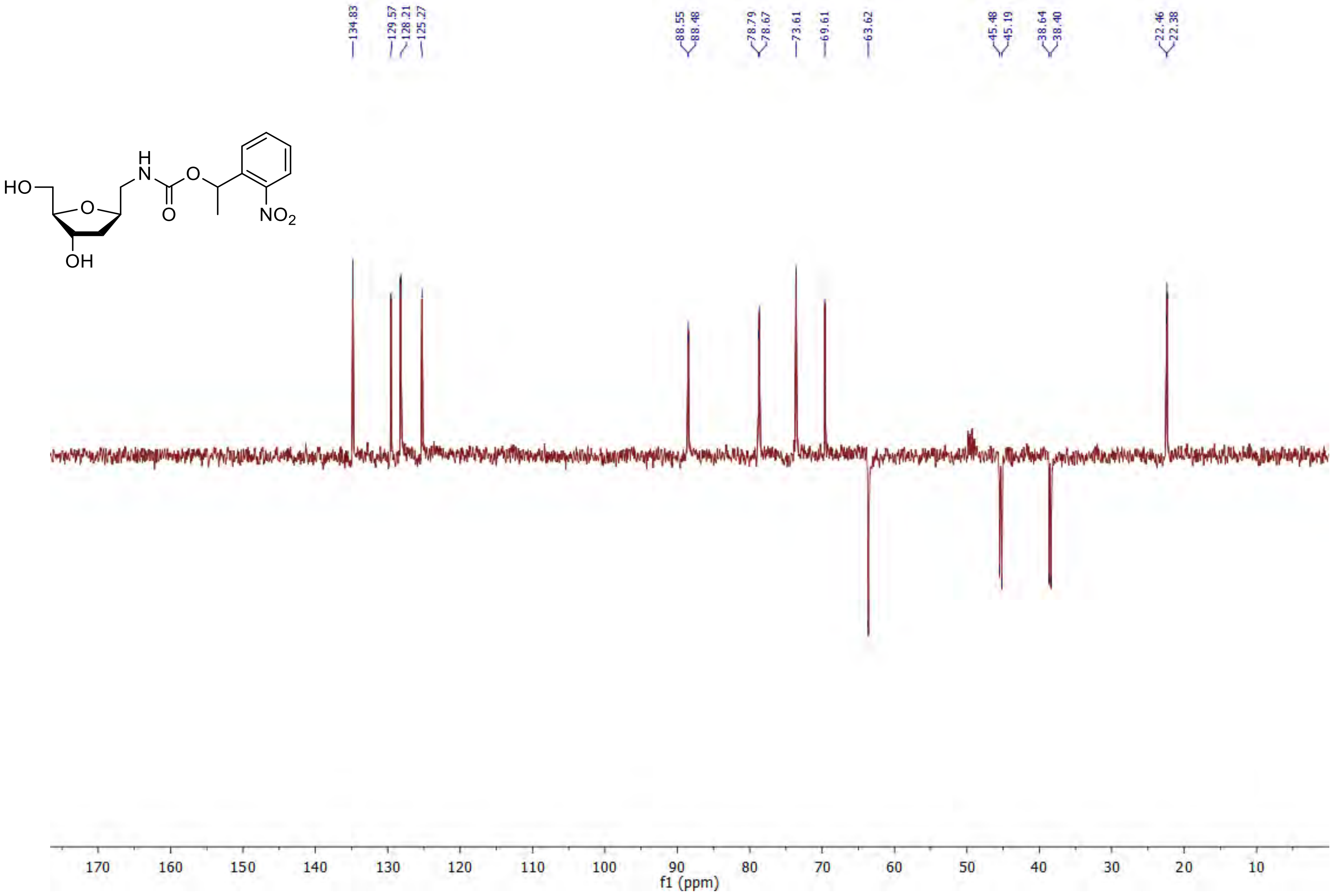

1,2-Dideoxy-1β-[(1-(2-nitrophenyl)ethoxy)carbonylaminomethyl]-D-*erythro*-pentofuranose (6β)

COSY NMR (MeOH-d_4)

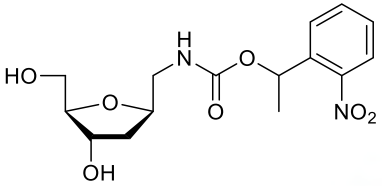

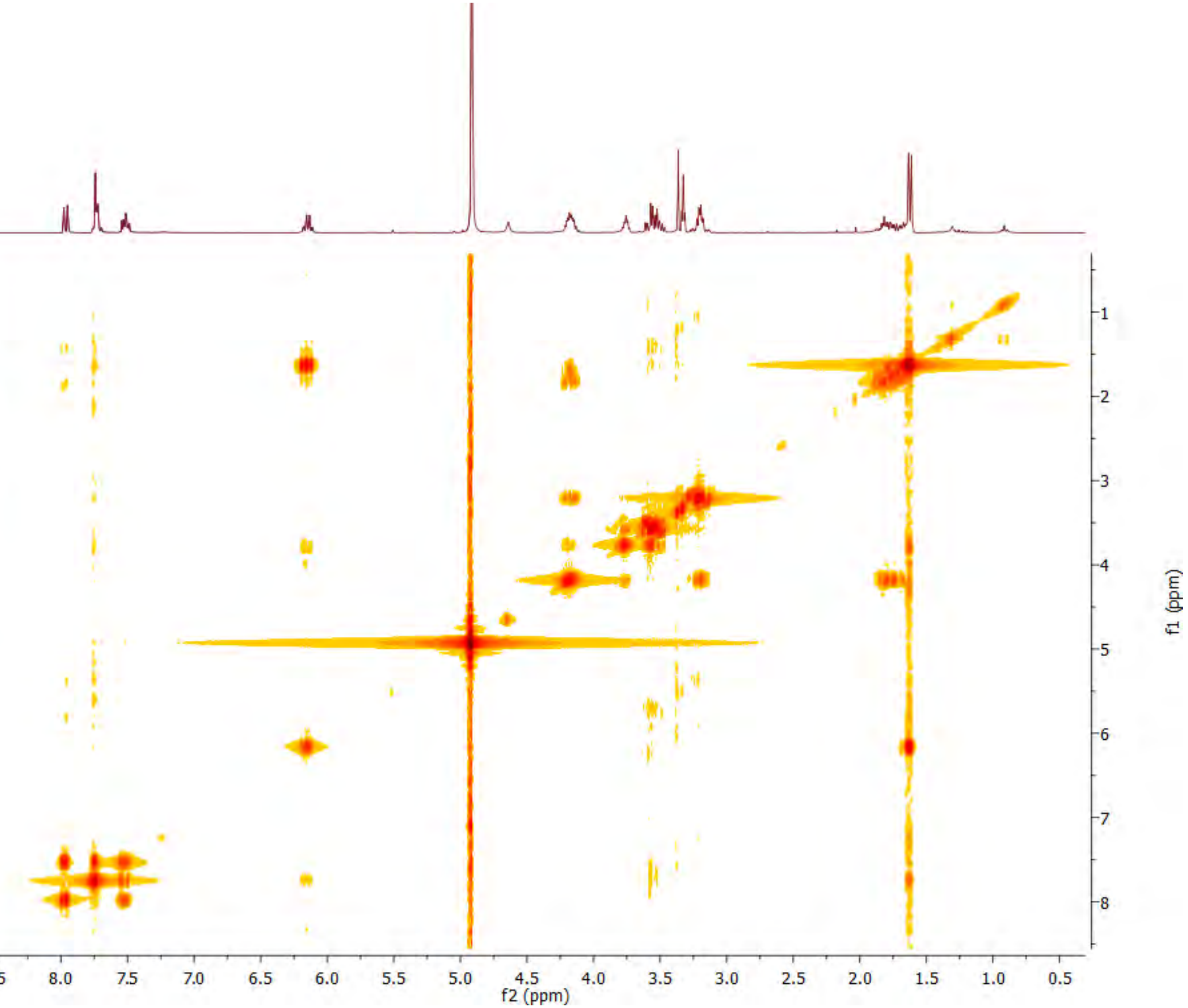

1,2-Dideoxy-1β-[(1-(2-nitrophenyl)ethoxy)carbonylaminomethyl]-D-*erythro*-pentofuranose (6β)

HSQC NMR (MeOH-d_4)

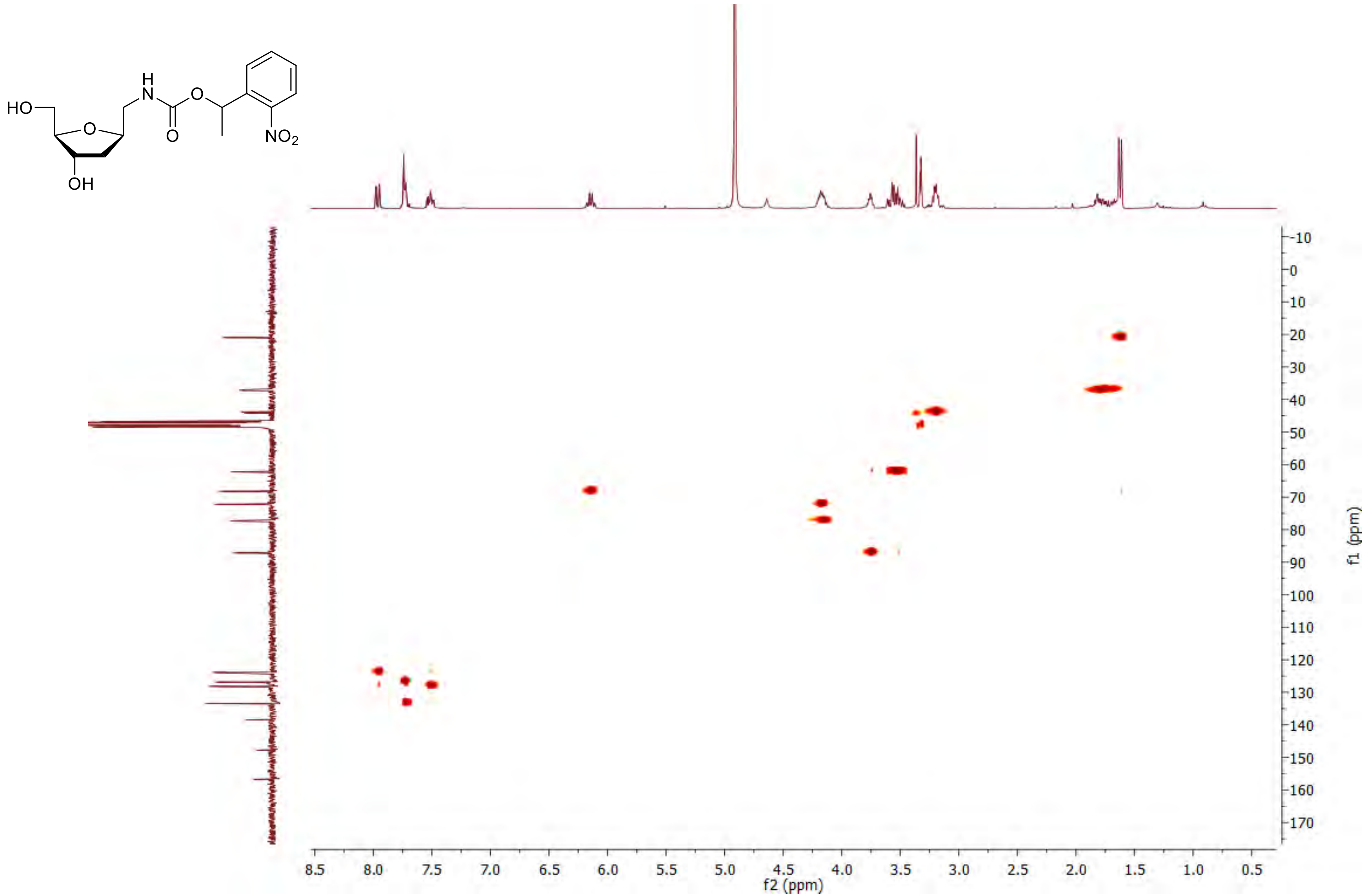

1,2-Dideoxy-1β-[(1-(2-nitrophenyl)ethoxy)carbonylaminomethyl]-D-*erythro*-pentofuranose (6β)

HMBC NMR (MeOH-d_4)

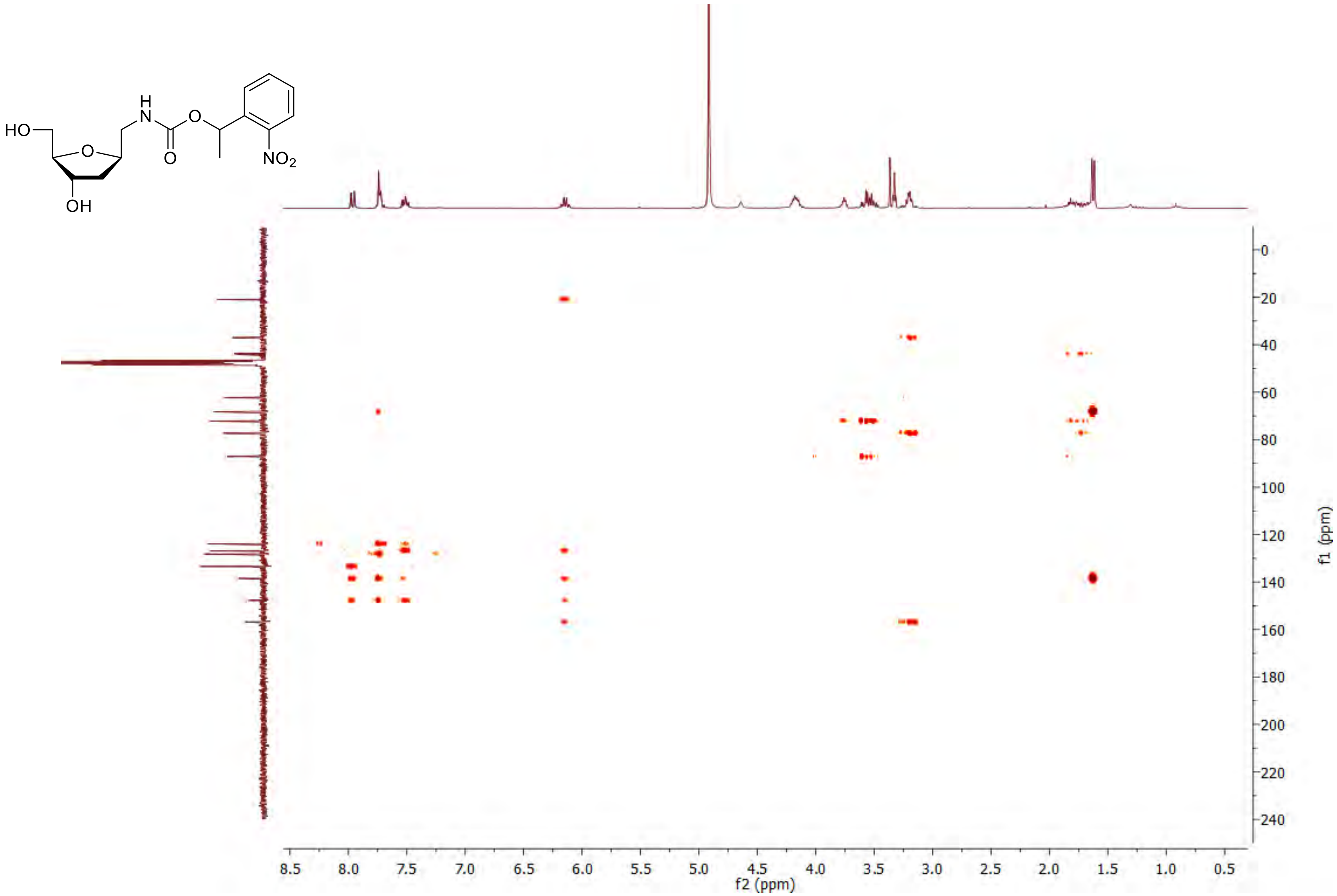

1,2-Dideoxy-5-*O*-(4,4’-dimethoxytrityl)-1α-[(1-(2-nitrophenyl)ethoxy)carbonylamino-methyl]-D-*erythro*-pentofuranose (7α)

^{1}H NMR (300.13 MHz, MeOH-d_4)

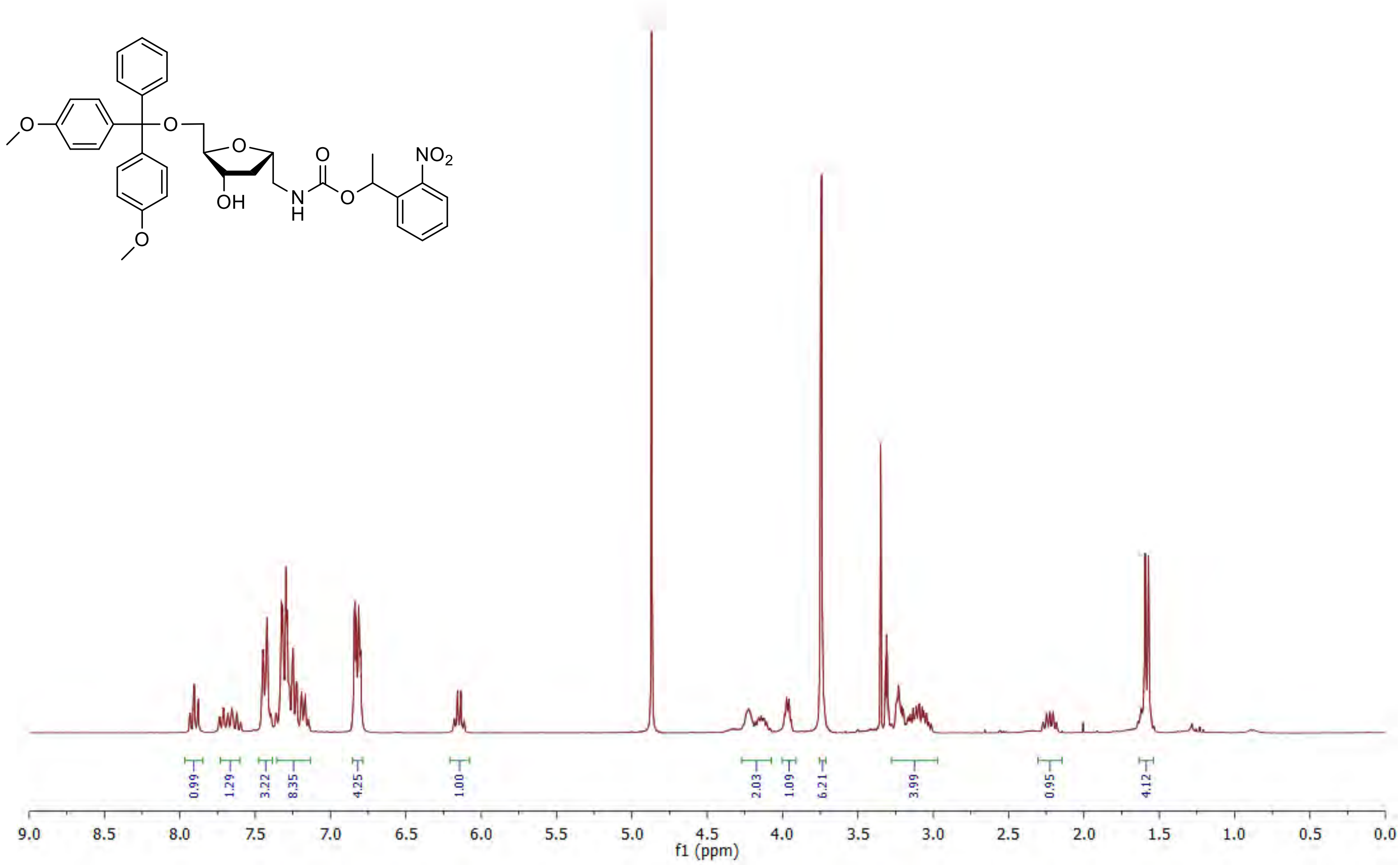

1,2-Dideoxy-5-*O*-(4,4'-dimethoxytrityl)-1α-[(1-(2-nitrophenyl)ethoxy)carbonylamino-methyl]-D-*erythro*-pentofuranose (7α)

^{13}C NMR (75.5 MHz, MeOH-d_4)

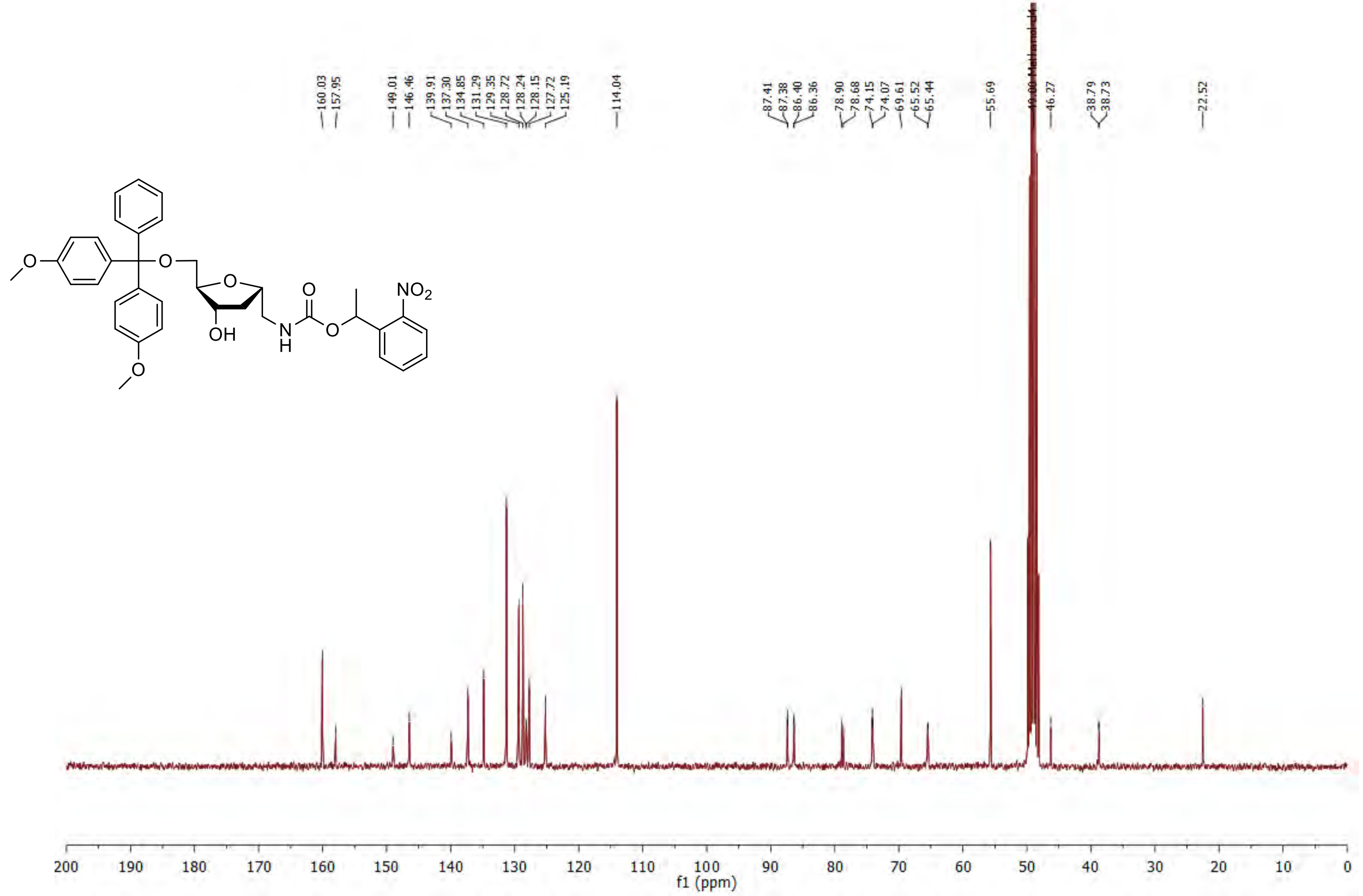

1,2-Dideoxy-5-*O*-(4,4'-dimethoxytrityl)-1α-[(1-(2-nitrophenyl)ethoxy)carbonylamino-methyl]-D-*erythro*-pentofuranose (7α)

DEPT135 NMR (75.5 MHz, MeOH-d_4)

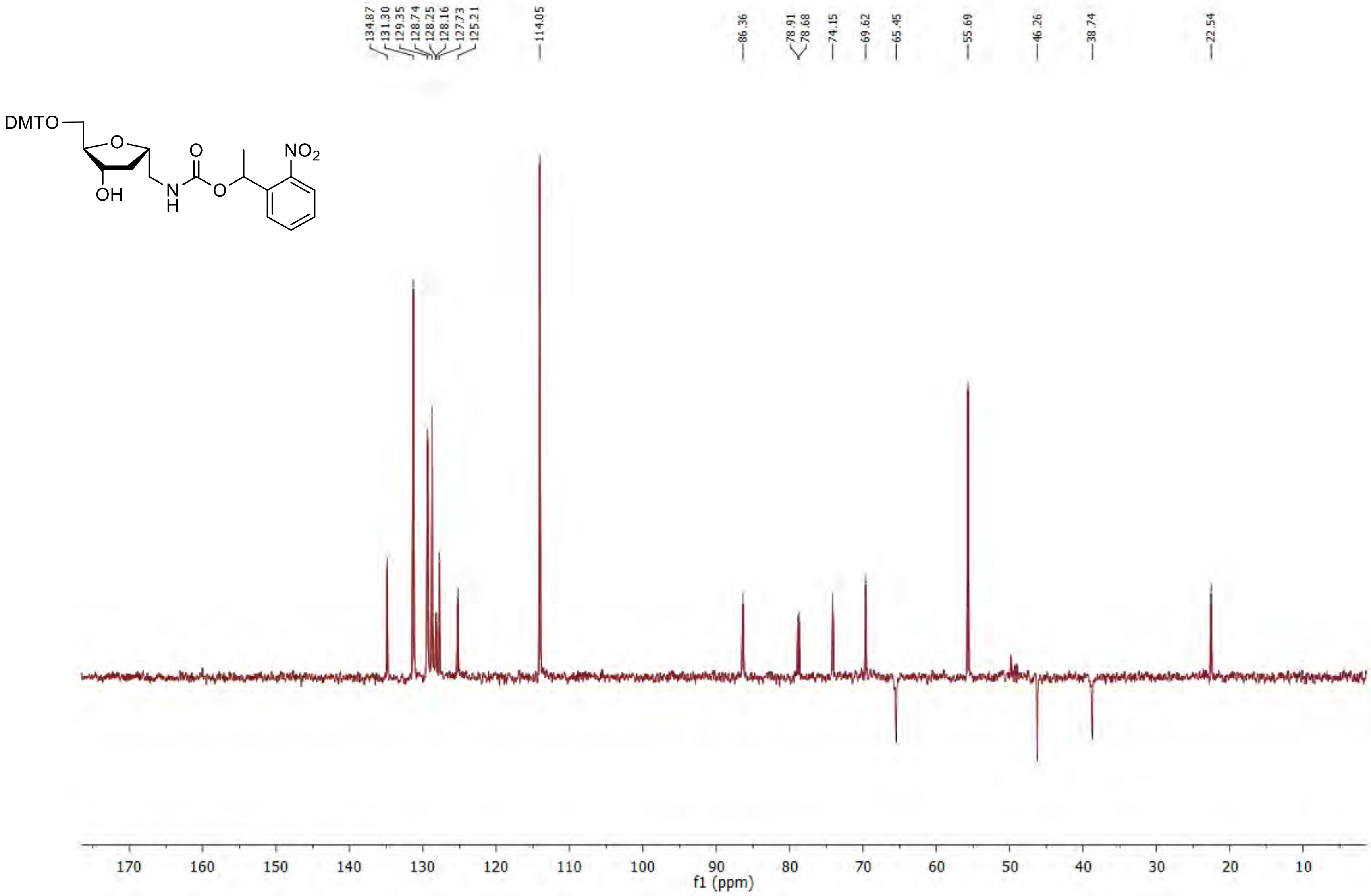

1,2-Dideoxy-5-*O*-(4,4’-dimethoxytrityl)-1α-[(1-(2-nitrophenyl)ethoxy)carbonylamino-methyl]-D-*erythro*-pentofuranose (7α)

COSY NMR (MeOH-d_4)

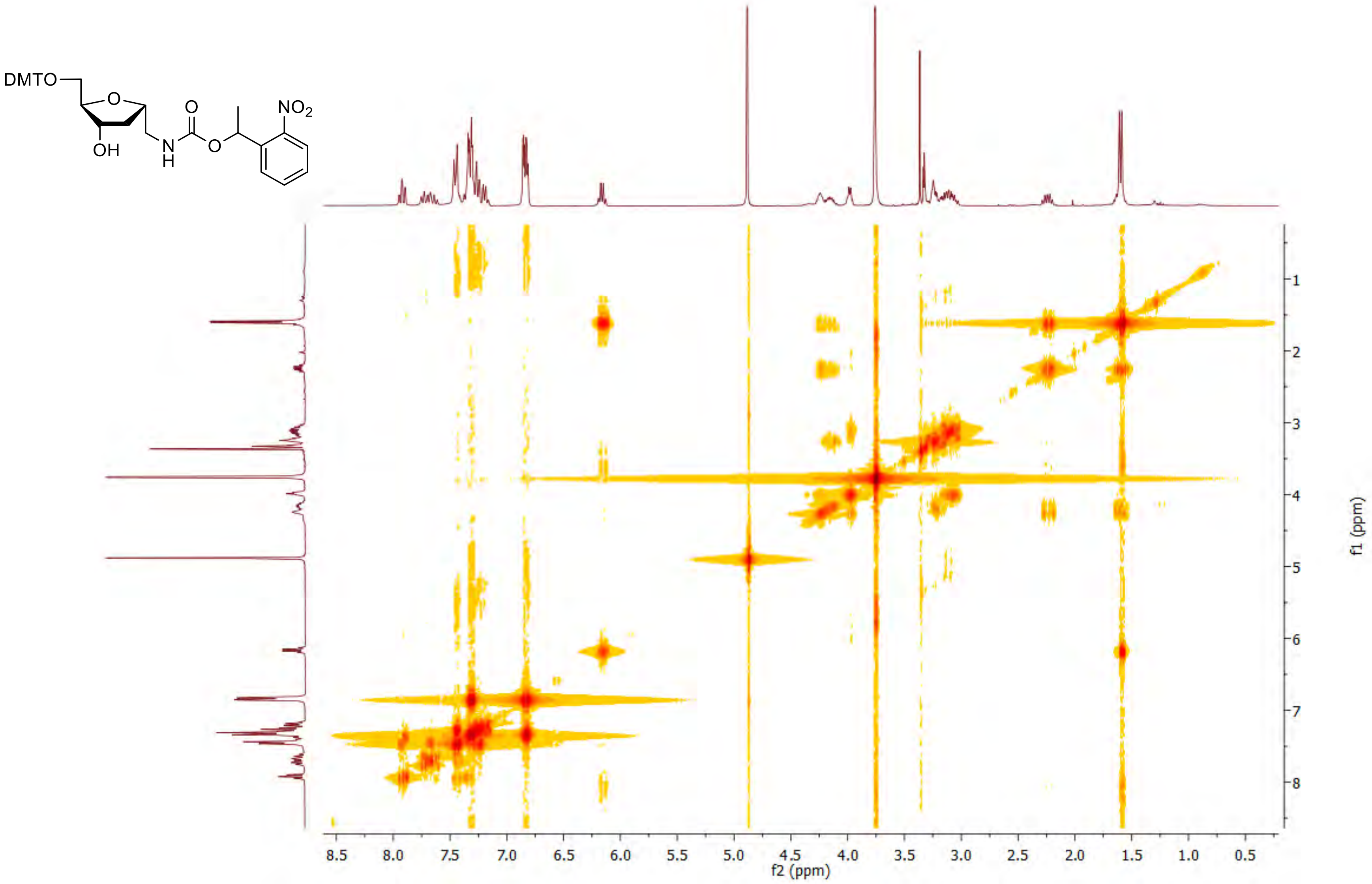

1,2-Dideoxy-5-*O*-(4,4'-dimethoxytrityl)-1α-[(1-(2-nitrophenyl)ethoxy)carbonylamino-methyl]-D-*erythro*-pentofuranose (7α)

HSQC NMR (MeOH-d_4)

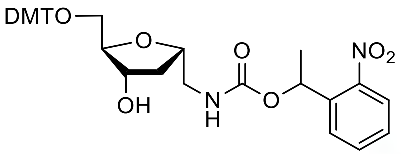

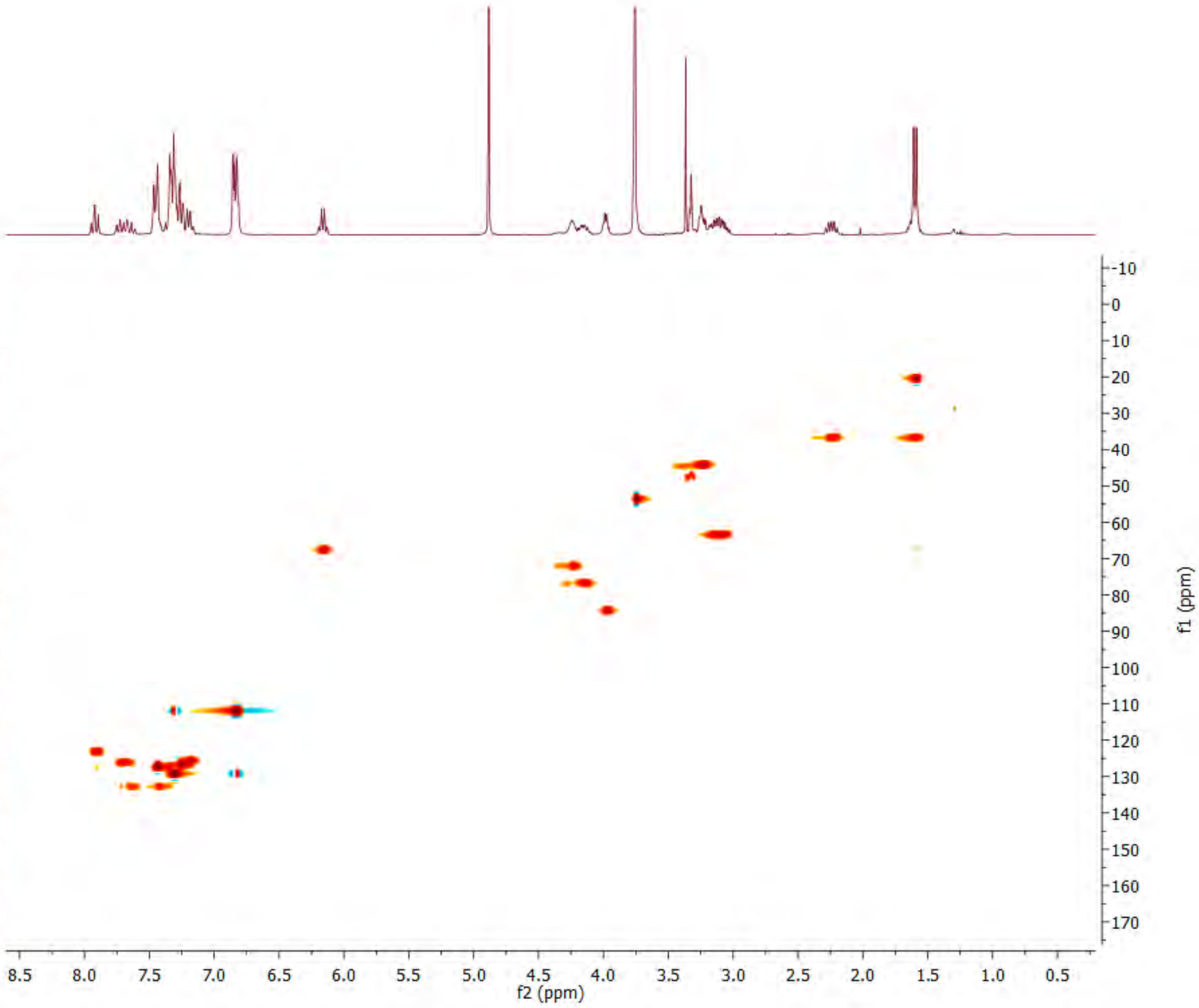

1,2-Dideoxy-5-*O*-(4,4’-dimethoxytrityl)-1α-[(1-(2-nitrophenyl)ethoxy)carbonylamino-methyl]-D-*erythro*-pentofuranose (7α)

HMBC NMR (MeOH-d_4)

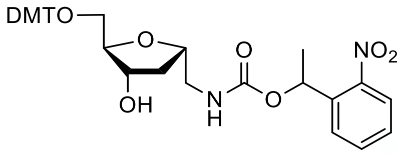

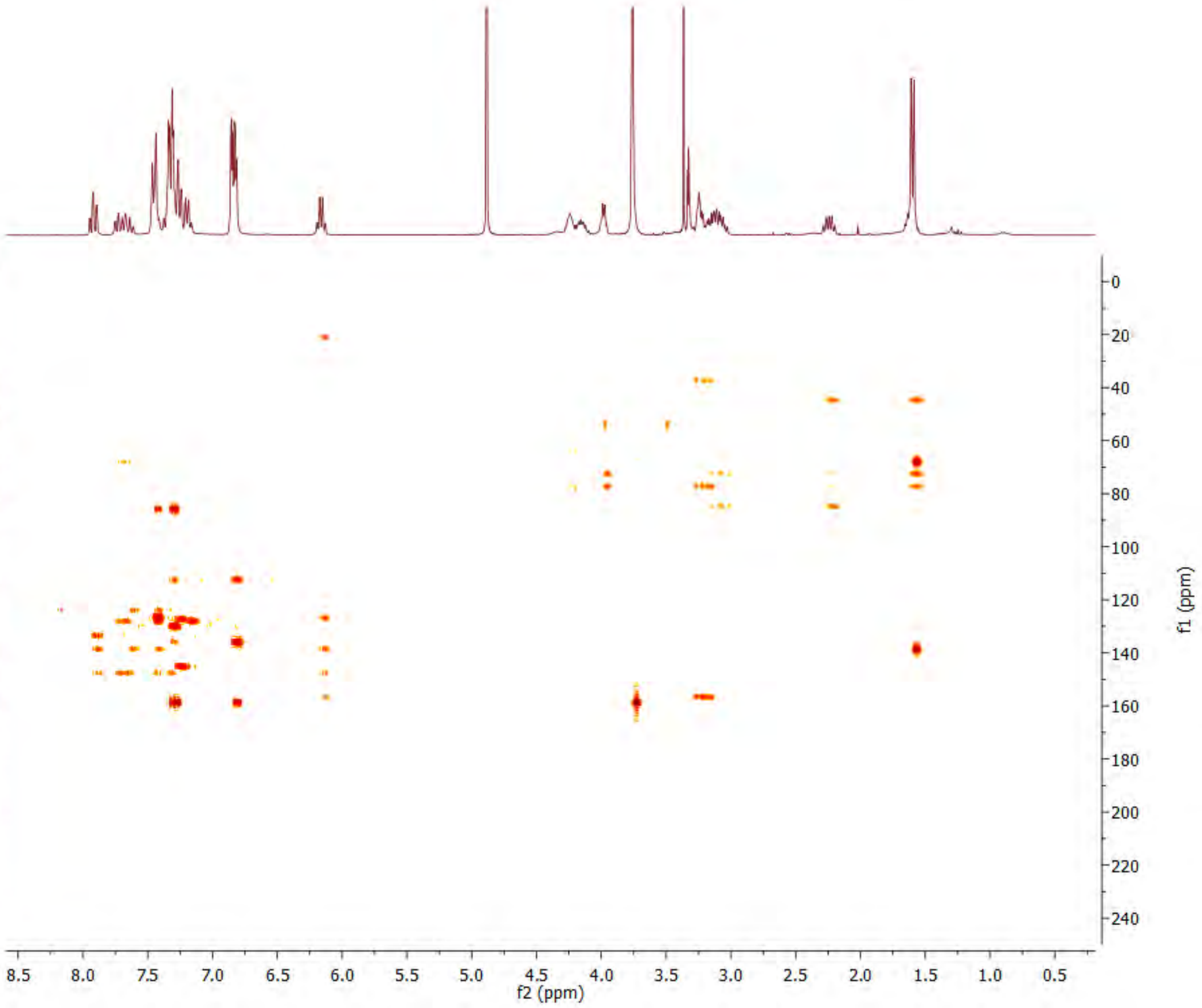

1,2-Dideoxy-5-*O*-(4,4’-dimethoxytrityl)-1β-[(1-(2-nitrophenyl)ethoxy)carbonylamino-methyl]-D-*erythro*-pentofuranose (7β)

^{1}H NMR (300.13 MHz, MeOH-d_4)

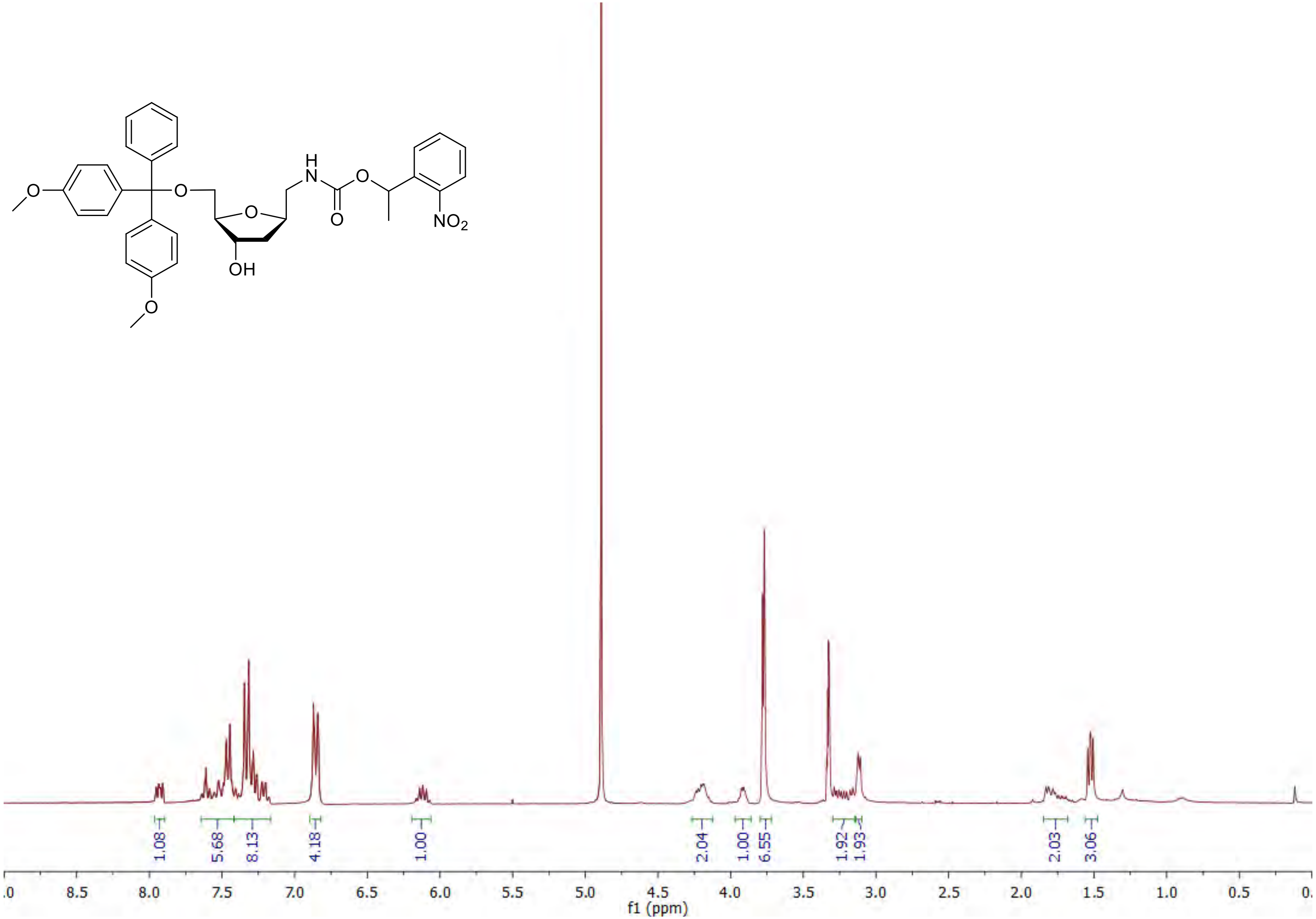

1,2-Dideoxy-5-*O*-(4,4’-dimethoxytrityl)-1β-[(1-(2-nitrophenyl)ethoxy)carbonylamino-methyl]-D-*erythro*-pentofuranose (7β)

^{13}C NMR (75.5 MHz, MeOH-d_4)

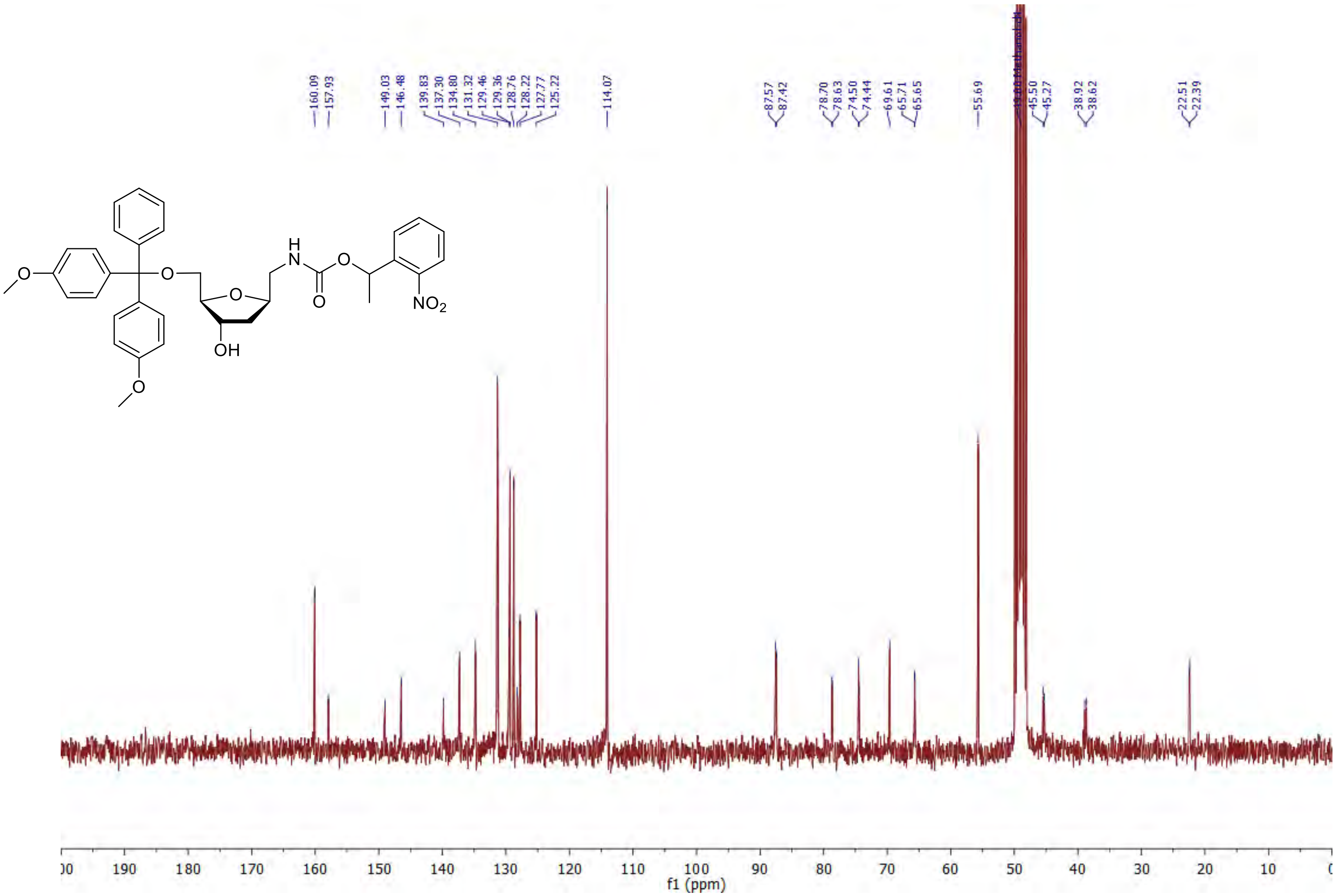

1,2-Dideoxy-5-*O*-(4,4'-dimethoxytrityl)-1β-[(1-(2-nitrophenyl)ethoxy)carbonylamino-methyl]-D-*erythro*-pentofuranose (7β)

DEPT 135 NMR (75.5 MHz, MeOH-d_4)

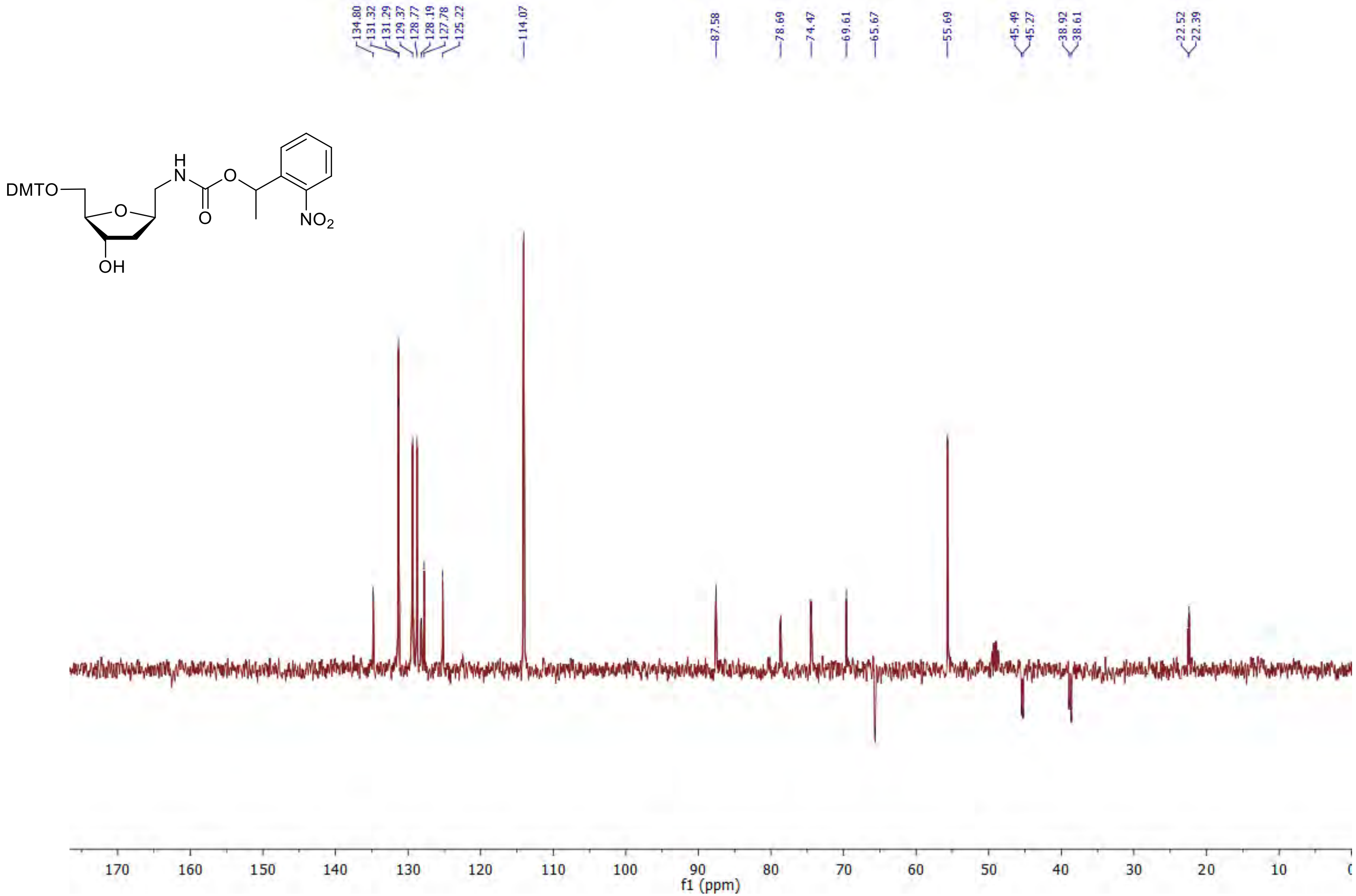

1,2-Dideoxy-5-*O*-(4,4'-dimethoxytrityl)-1β-[(1-(2-nitrophenyl)ethoxy)carbonylamino-methyl]-D-*erythro*-pentofuranose (7β)

COSY NMR (MeOH-d_4)

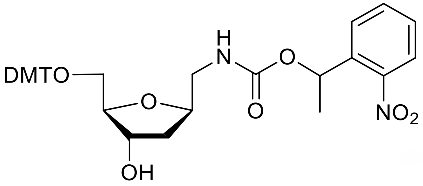

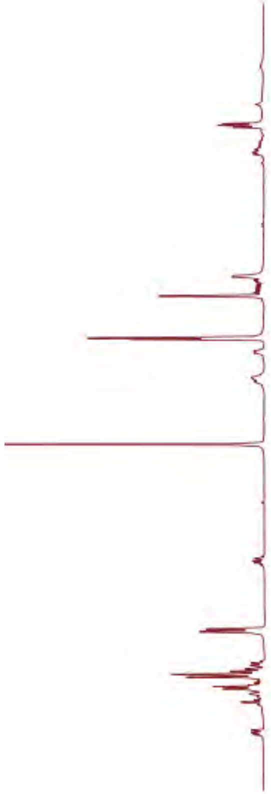

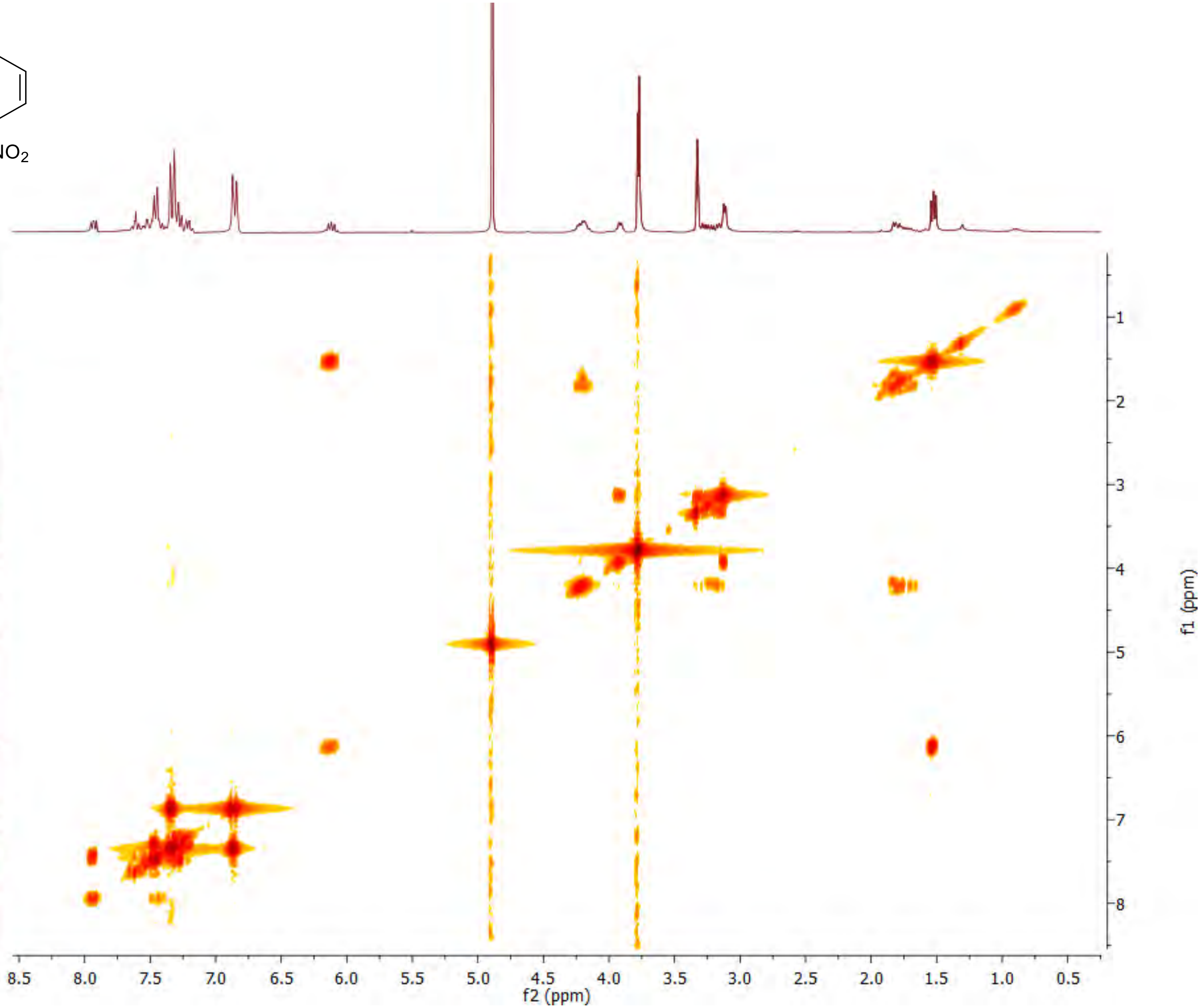

1,2-Dideoxy-5-*O*-(4,4'-dimethoxytrityl)-1β-[(1-(2-nitrophenyl)ethoxy)carbonylamino-methyl]-D-*erythro*-pentofuranose (7β)

HSQC NMR (MeOH-d_4)

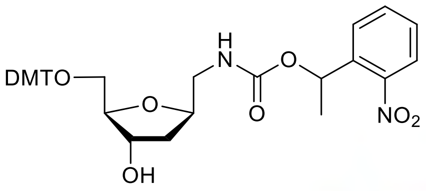

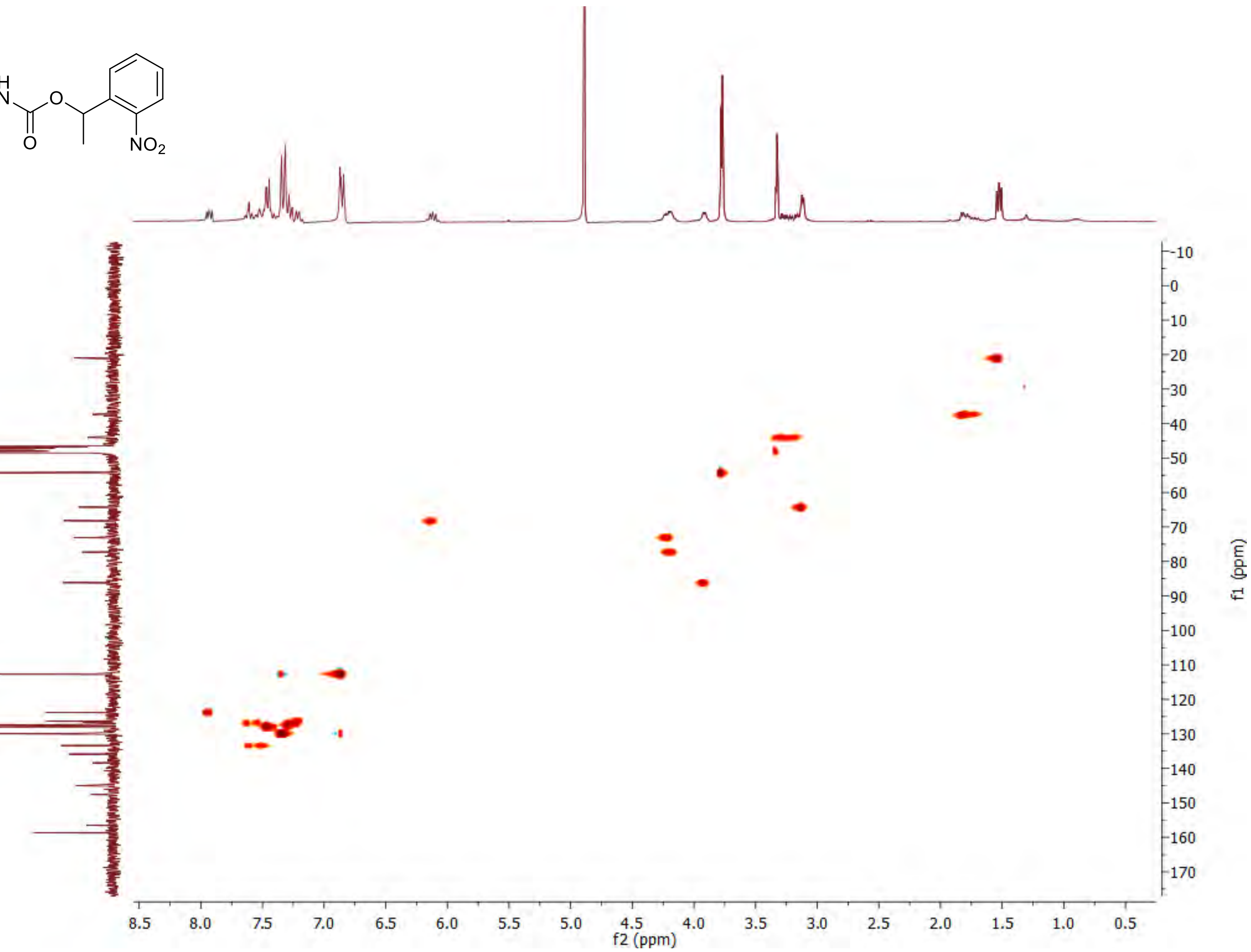

1,2-Dideoxy-5-*O*-(4,4'-dimethoxytrityl)-1β-[(1-(2-nitrophenyl)ethoxy)carbonylamino-methyl]-D-*erythro*-pentofuranose (7β)

HMBC NMR (MeOH-d_4)

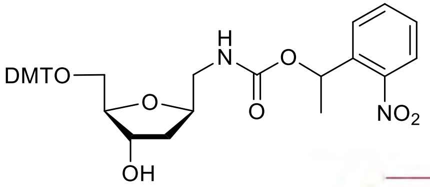

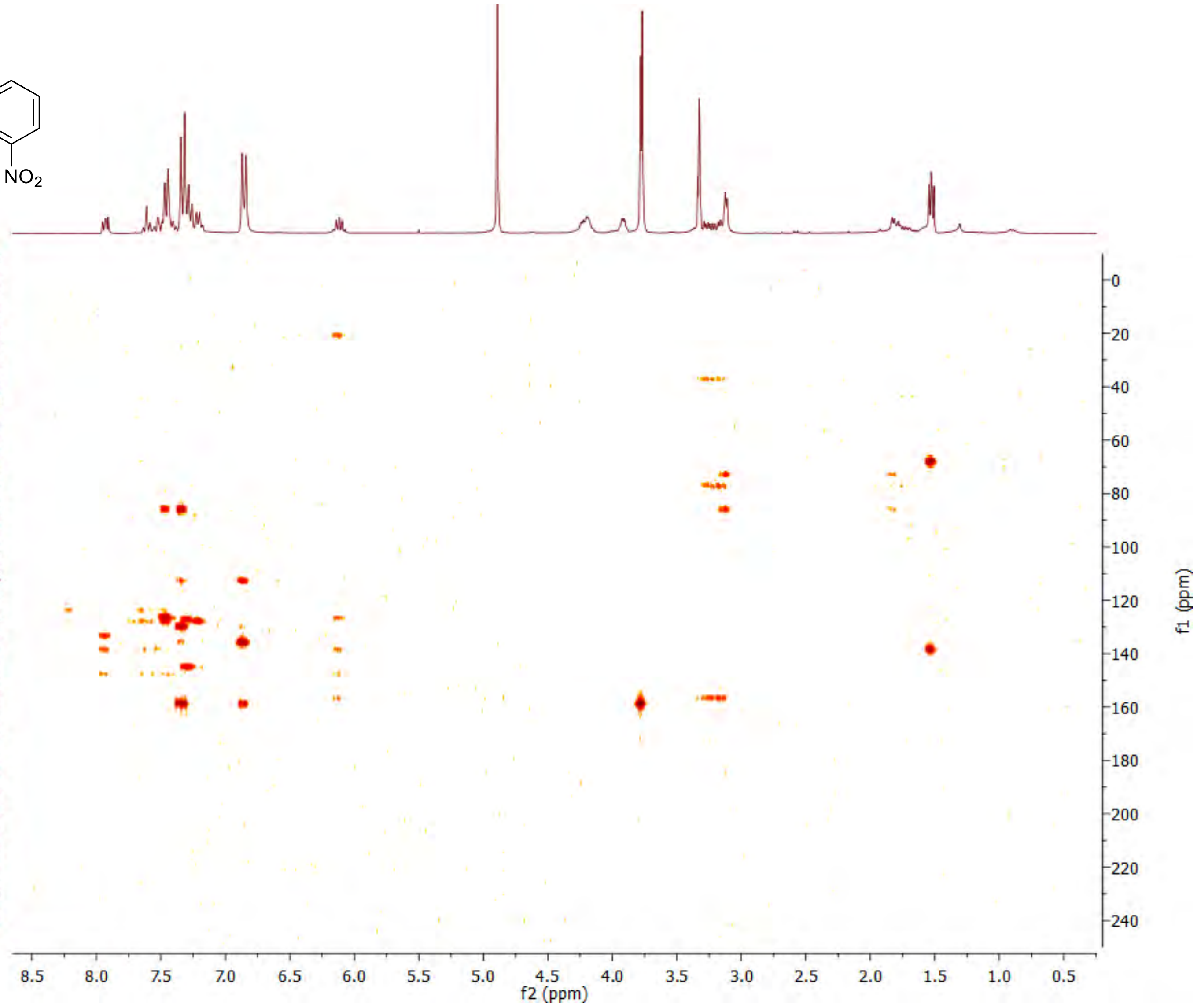

1,2-Dideoxy-5-*O*-(4,4’-dimethoxytrityl)-1α-[(1-(2-nitrophenyl)ethoxy)carbonylamino-methyl]-D-*erythro*-pentofuranosyl-3-*O*-(2-cyanoethyl-*N*,*N*-diisopropyl)phosphoramidite (8α-A)

^{1}H NMR (300.13 MHz, MeOH-d_4)

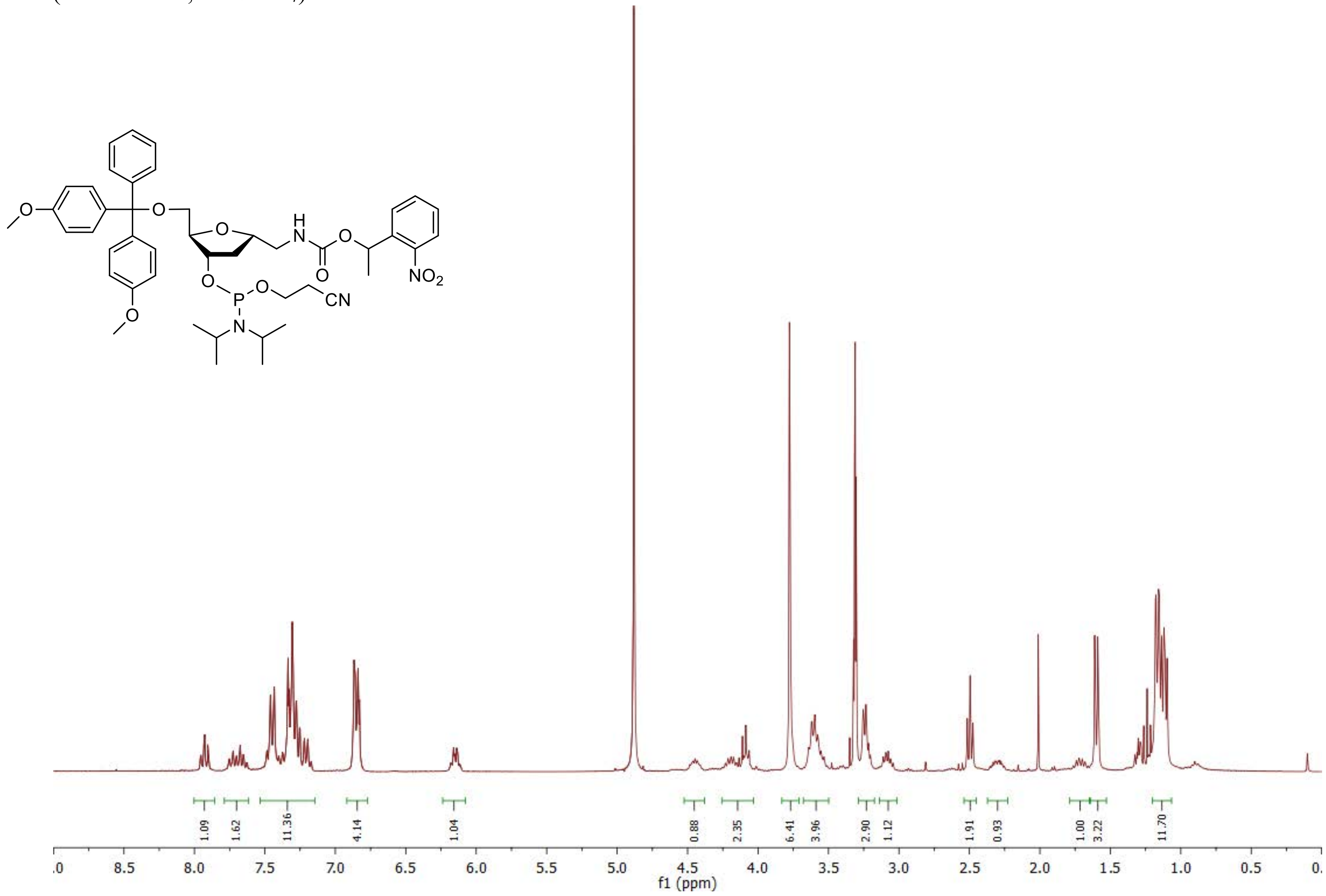

1,2-Dideoxy-5-*O*-(4,4'-dimethoxytrityl)-1α-[(1-(2-nitrophenyl)ethoxy)carbonylamino-methyl]-D-*erythro*-pentofuranosyl-3-*O*-(2-cyanoethyl-*N*,*N*-diisopropyl)phosphoramidite (8α-A)

^{13}C NMR (75.5 MHz, MeOH-d_4)

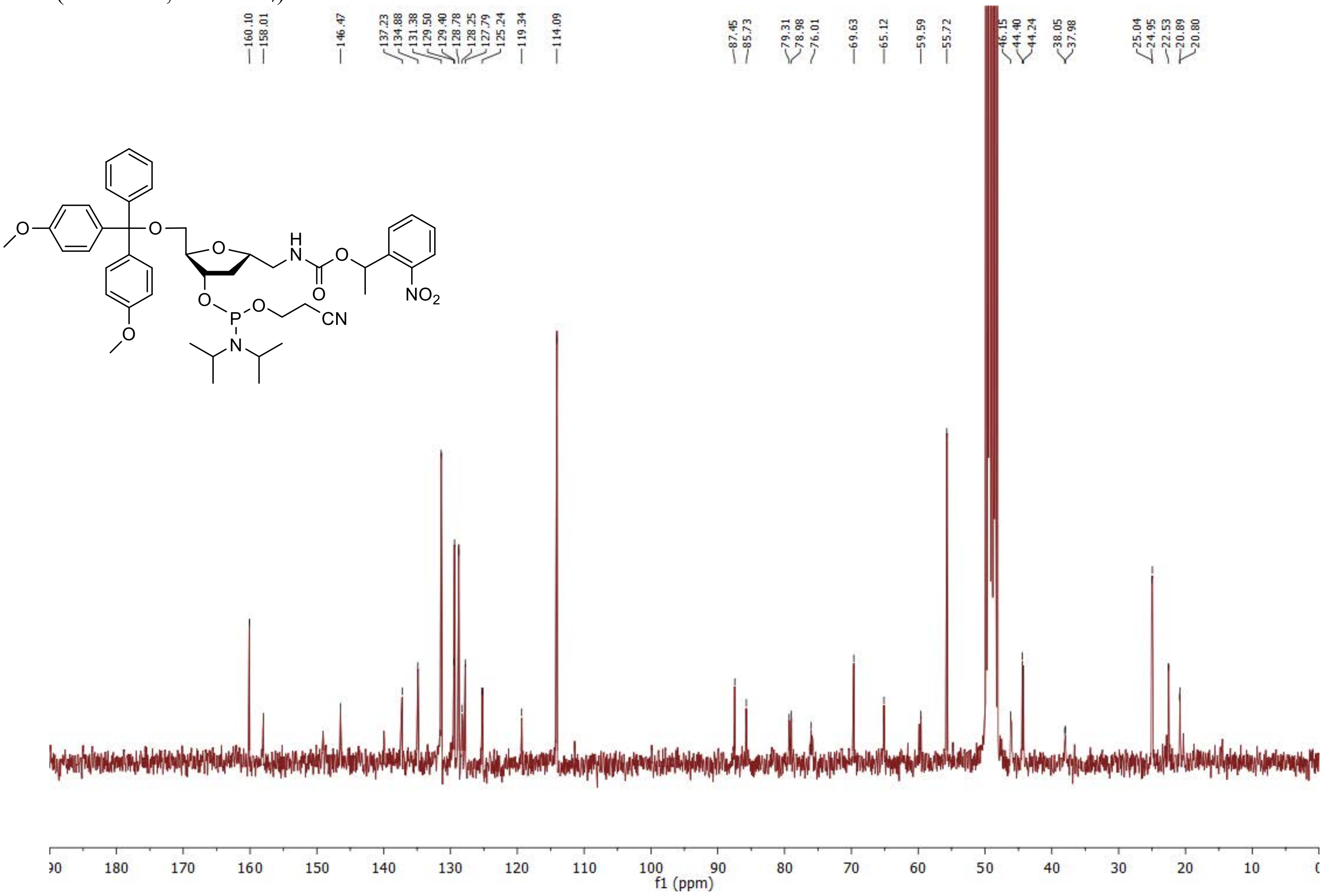

1,2-Dideoxy-5-*O*-(4,4'-dimethoxytrityl)-1α-[(1-(2-nitrophenyl)ethoxy)carbonylamino-methyl]-D-*erythro*-pentofuranosyl-3-*O*-(2-cyanoethyl-*N*,*N*-diisopropyl)phosphoramidite (8α-A)

DEPT 135 NMR (75.5 MHz, MeOH-d_4)

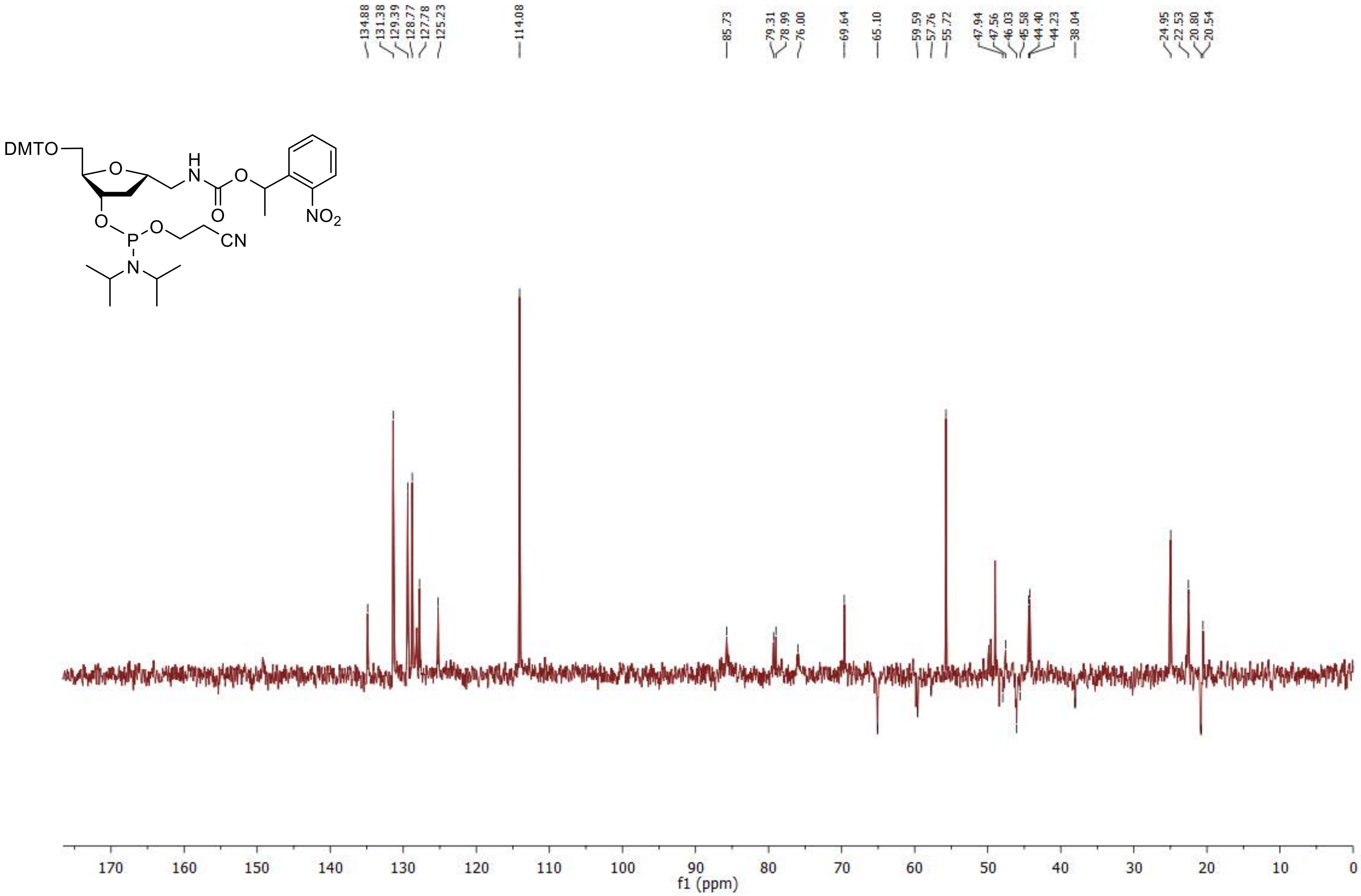

1,2-Dideoxy-5-*O*-(4,4'-dimethoxytrityl)-1α-[(1-(2-nitrophenyl)ethoxy)carbonylamino-methyl]-D-*erythro*-pentofuranosyl-3-*O*-(2-cyanoethyl-*N*,*N*-diisopropyl)phosphoramidite (8α-A)

COSY NMR (MeOH-d_4)

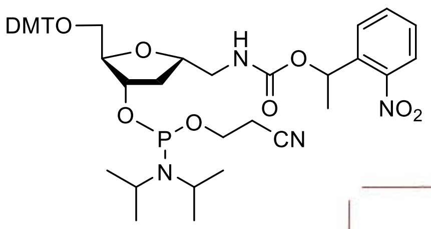

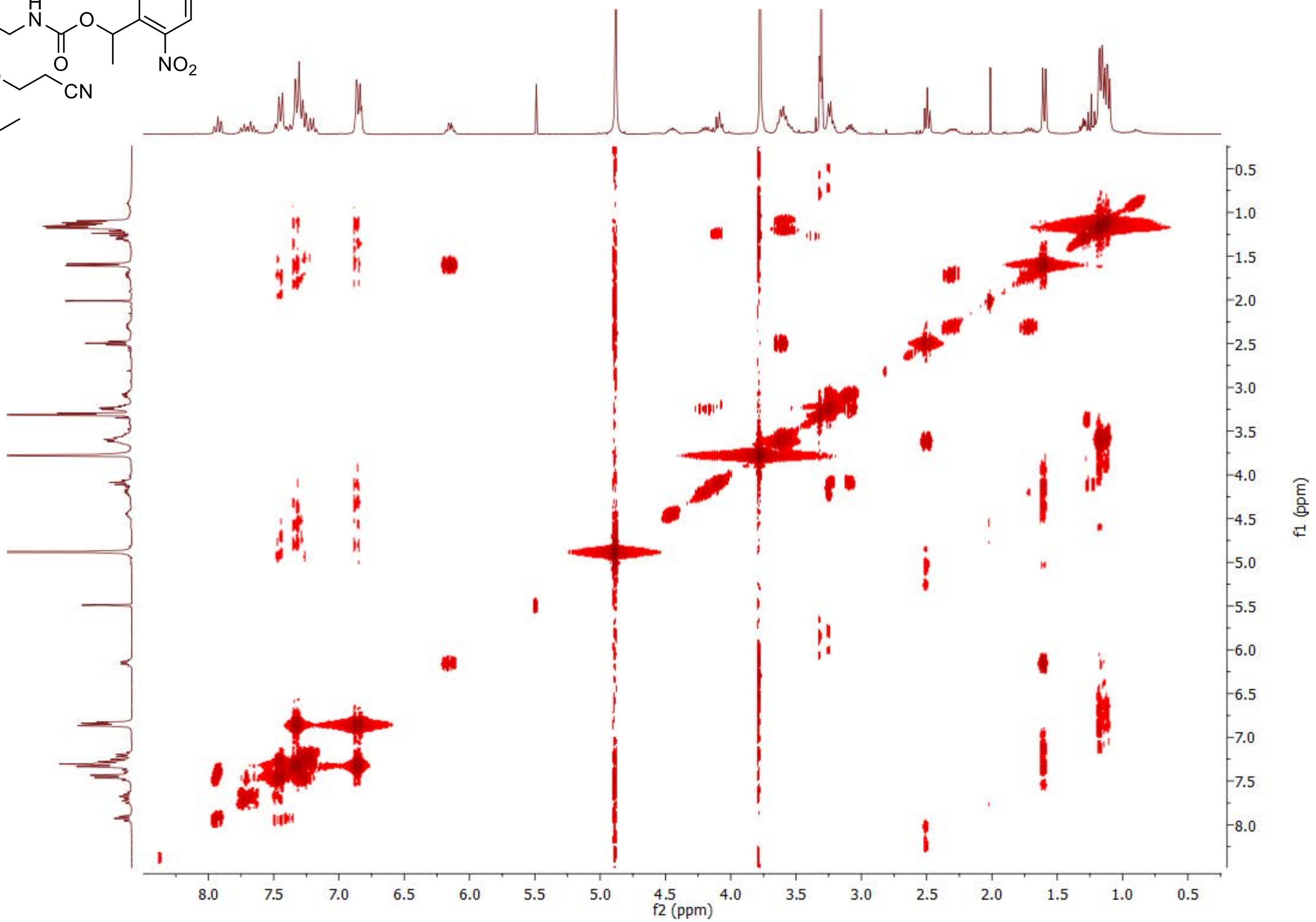

1,2-Dideoxy-5-*O*-(4,4'-dimethoxytrityl)-1α-[(1-(2-nitrophenyl)ethoxy)carbonylamino-methyl]-D-*erythro*-pentofuranosyl-3-*O*-(2-cyanoethyl-*N*,*N*-diisopropyl)phosphoramidite (8α-A)

HSQC NMR (MeOH-d_4)

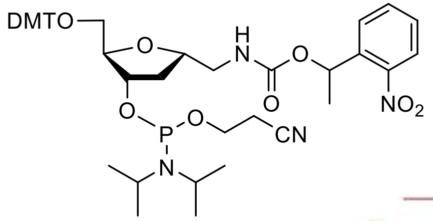

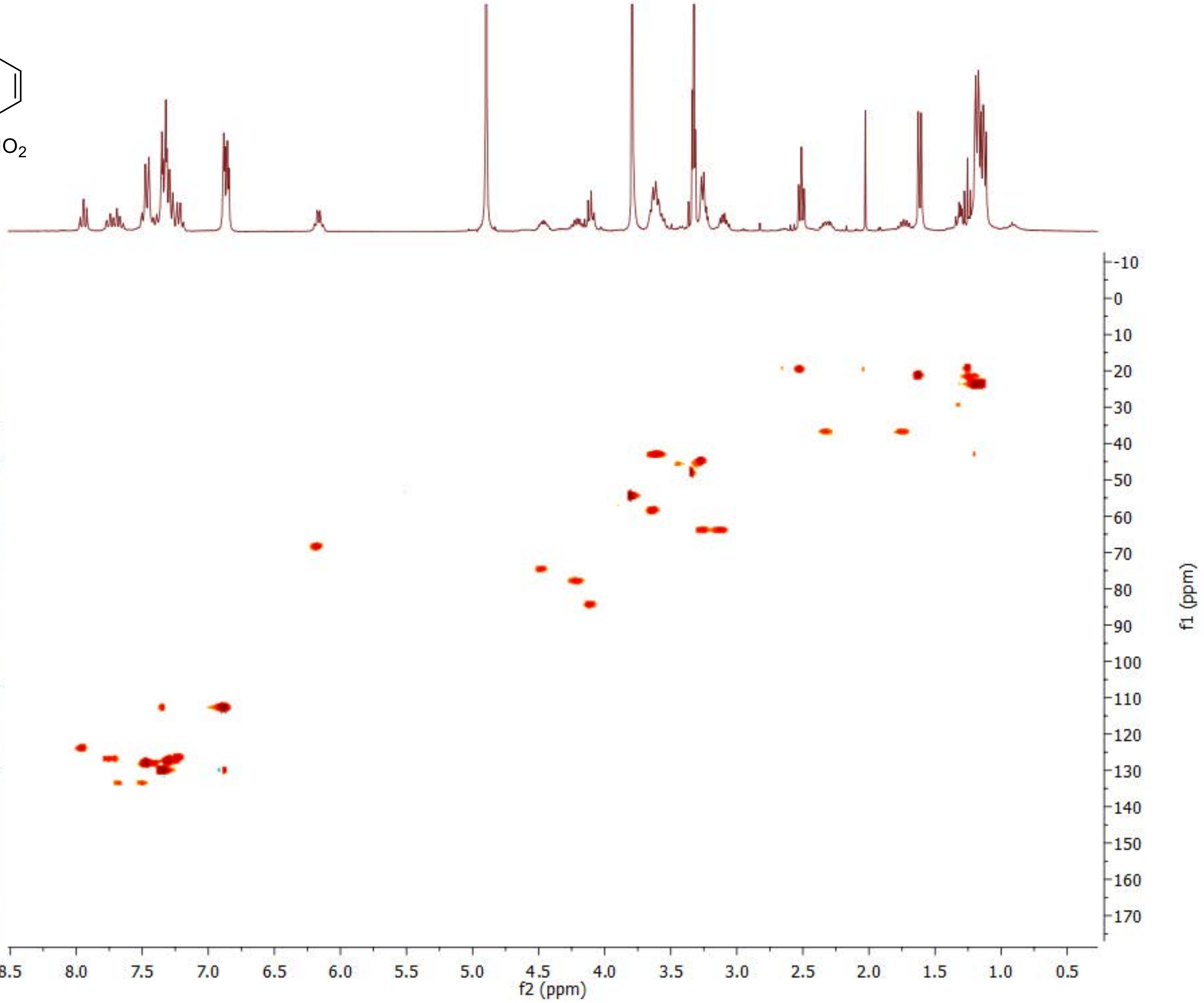

1,2-Dideoxy-5-*O*-(4,4’-dimethoxytrityl)-1α-[(1-(2-nitrophenyl)ethoxy)carbonylamino-methyl]-D-*erythro*-pentofuranosyl-3-*O*-(2-cyanoethyl-*N*,*N*-diisopropyl)phosphoramidite (8α-A)

^{31}P NMR (121.5 MHz, MeOH-d_4)

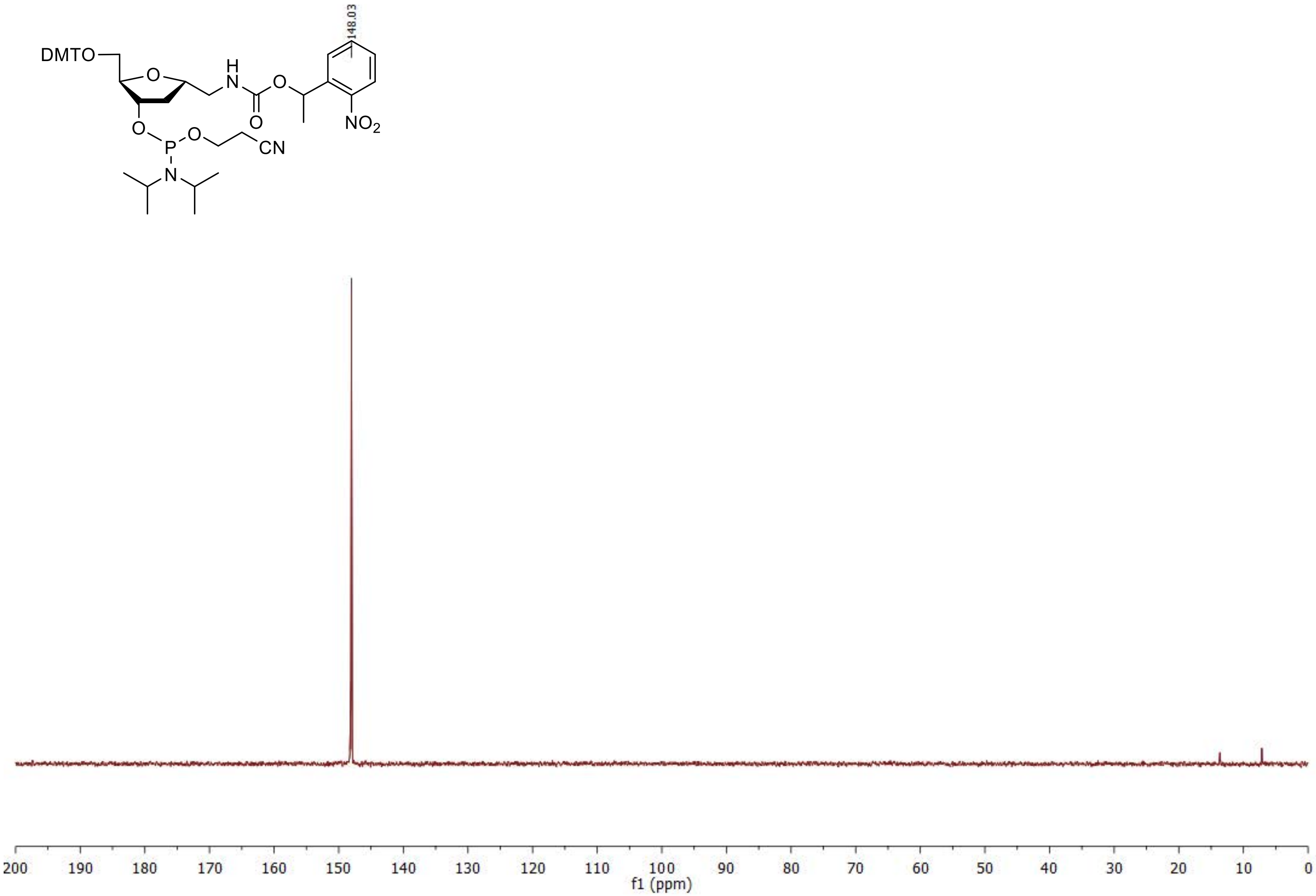

1,2-Dideoxy-5-*O*-(4,4'-dimethoxytrityl)-1α-[(1-(2-nitrophenyl)ethoxy)carbonylamino-methyl]-D-*erythro*-pentofuranosyl-3-*O*-(2-cyanoethyl-*N*,*N*-diisopropyl)phosphoramidite (8α-B)

^{1}H NMR (300.13 MHz, MeOH-d_4)

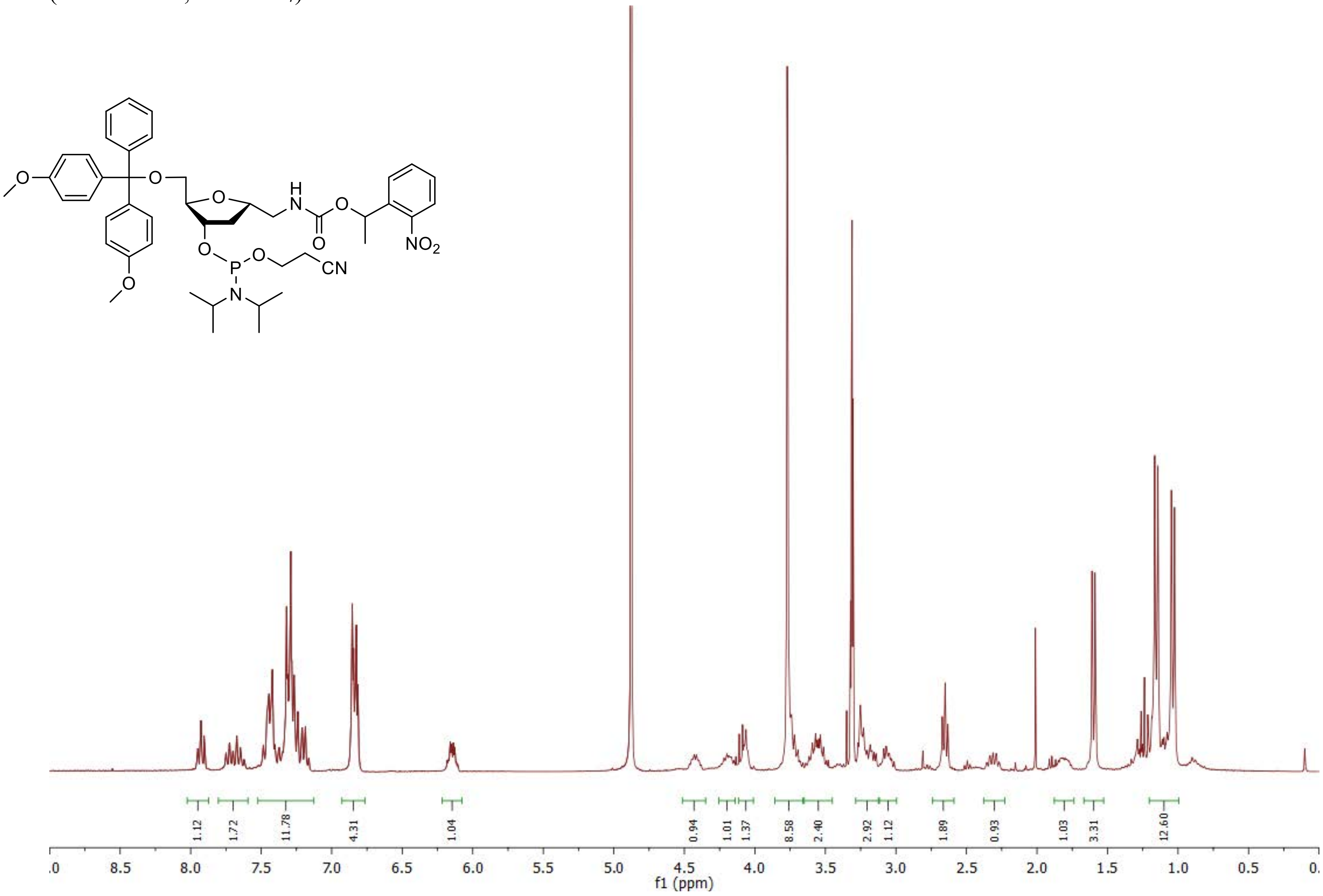

1,2-Dideoxy-5-*O*-(4,4'-dimethoxytrityl)-1α-[(1-(2-nitrophenyl)ethoxy)carbonylamino-methyl]-D-*erythro*-pentofuranosyl-3-*O*-(2-cyanoethyl-*N*,*N*-diisopropyl)phosphoramidite (8α-B)

^{13}C NMR (75.5 MHz, MeOH-d_4)

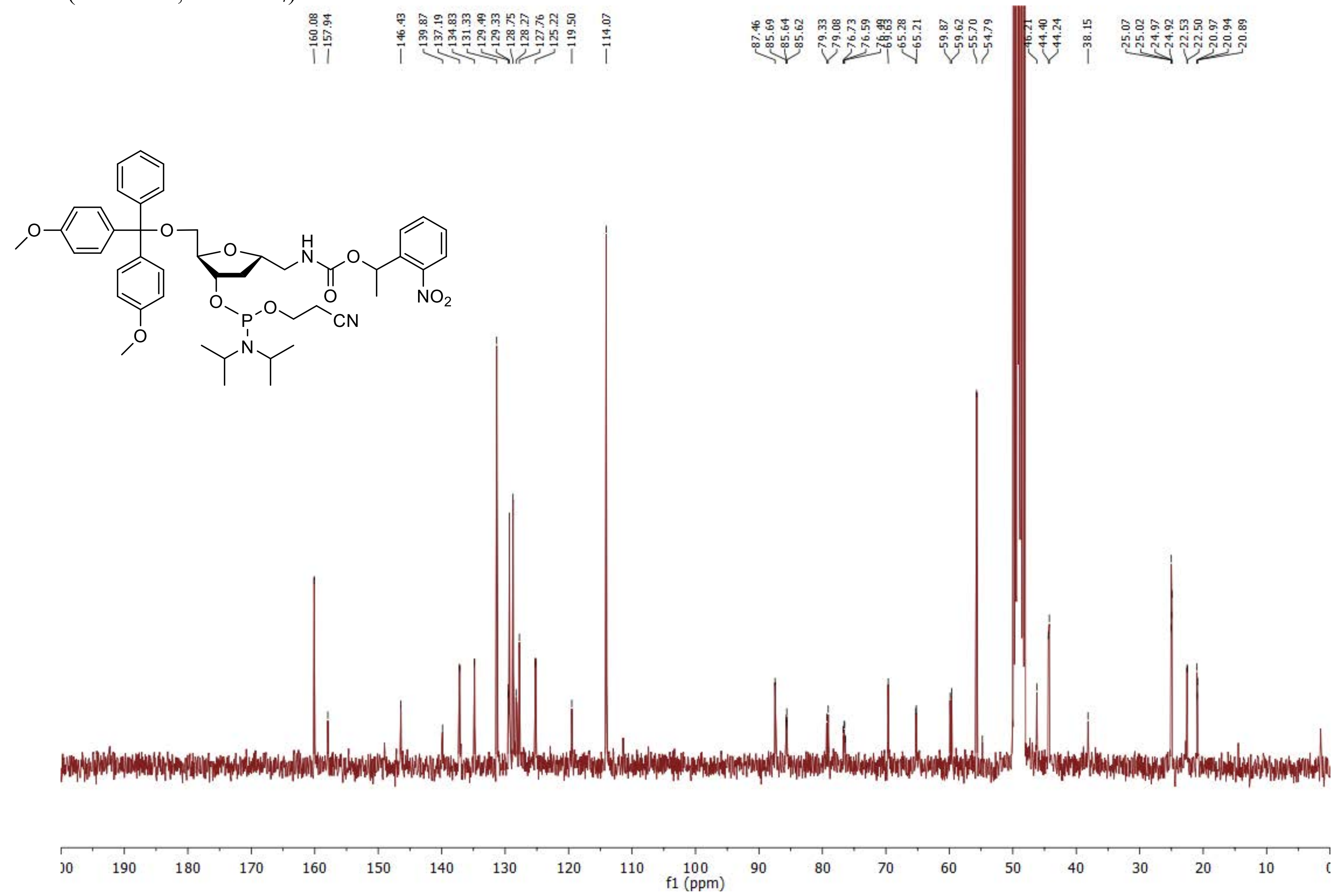

1,2-Dideoxy-5-*O*-(4,4'-dimethoxytrityl)-1α-[(1-(2-nitrophenyl)ethoxy)carbonylamino-methyl]-D-*erythro*-pentofuranosyl-3-*O*-(2-cyanoethyl-*N*,*N*-diisopropyl)phosphoramidite (8α-B)

DEPT 135 NMR (75.5 MHz, MeOH-d_4)

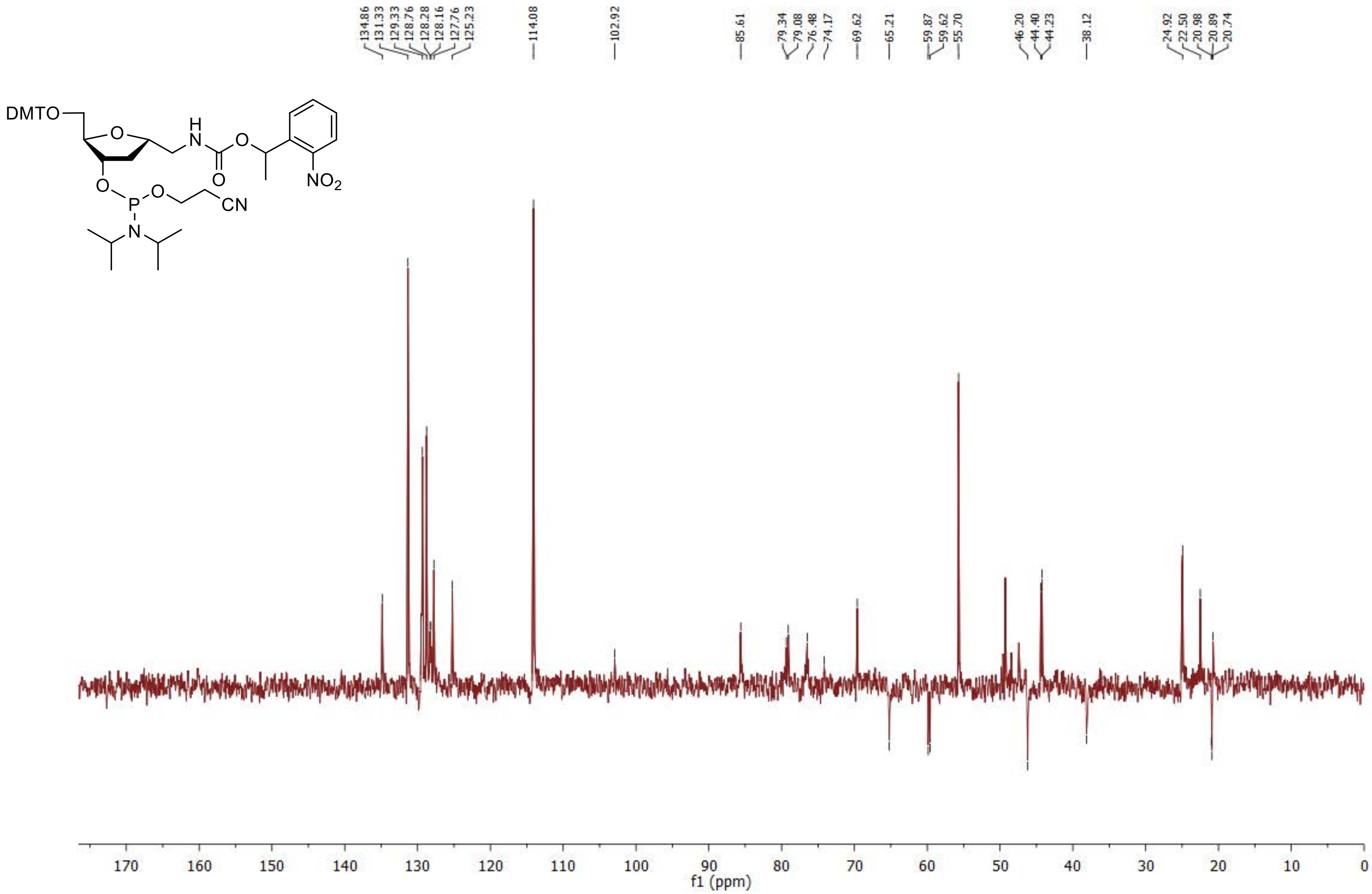

1,2-Dideoxy-5-*O*-(4,4'-dimethoxytrityl)-1α-[(1-(2-nitrophenyl)ethoxy)carbonylamino-methyl]-D-*erythro*-pentofuranosyl-3-*O*-(2-cyanoethyl-*N*,*N*-diisopropyl)phosphoramidite (8α-B)

COSY NMR (MeOH-d_4)

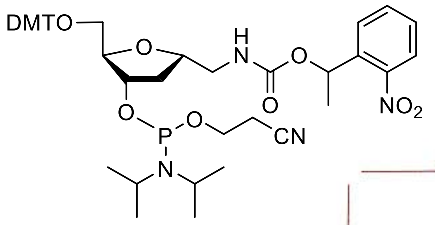

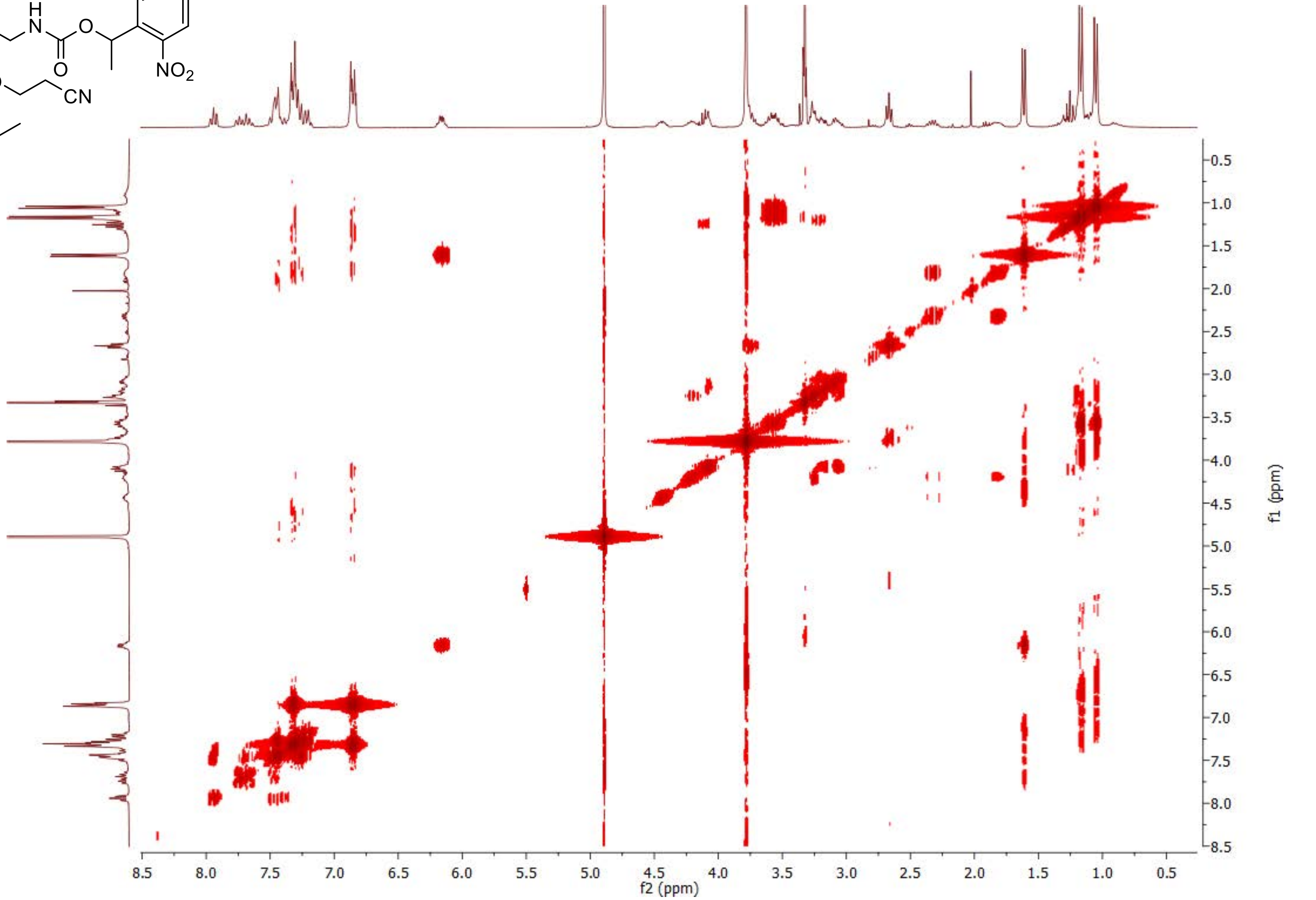

1,2-Dideoxy-5-*O*-(4,4'-dimethoxytrityl)-1α-[(1-(2-nitrophenyl)ethoxy)carbonylamino-methyl]-D-*erythro*-pentofuranosyl-3-*O*-(2-cyanoethyl-*N*,*N*-diisopropyl)phosphoramidite (8α-B)

HSQC NMR (MeOH-d_4)

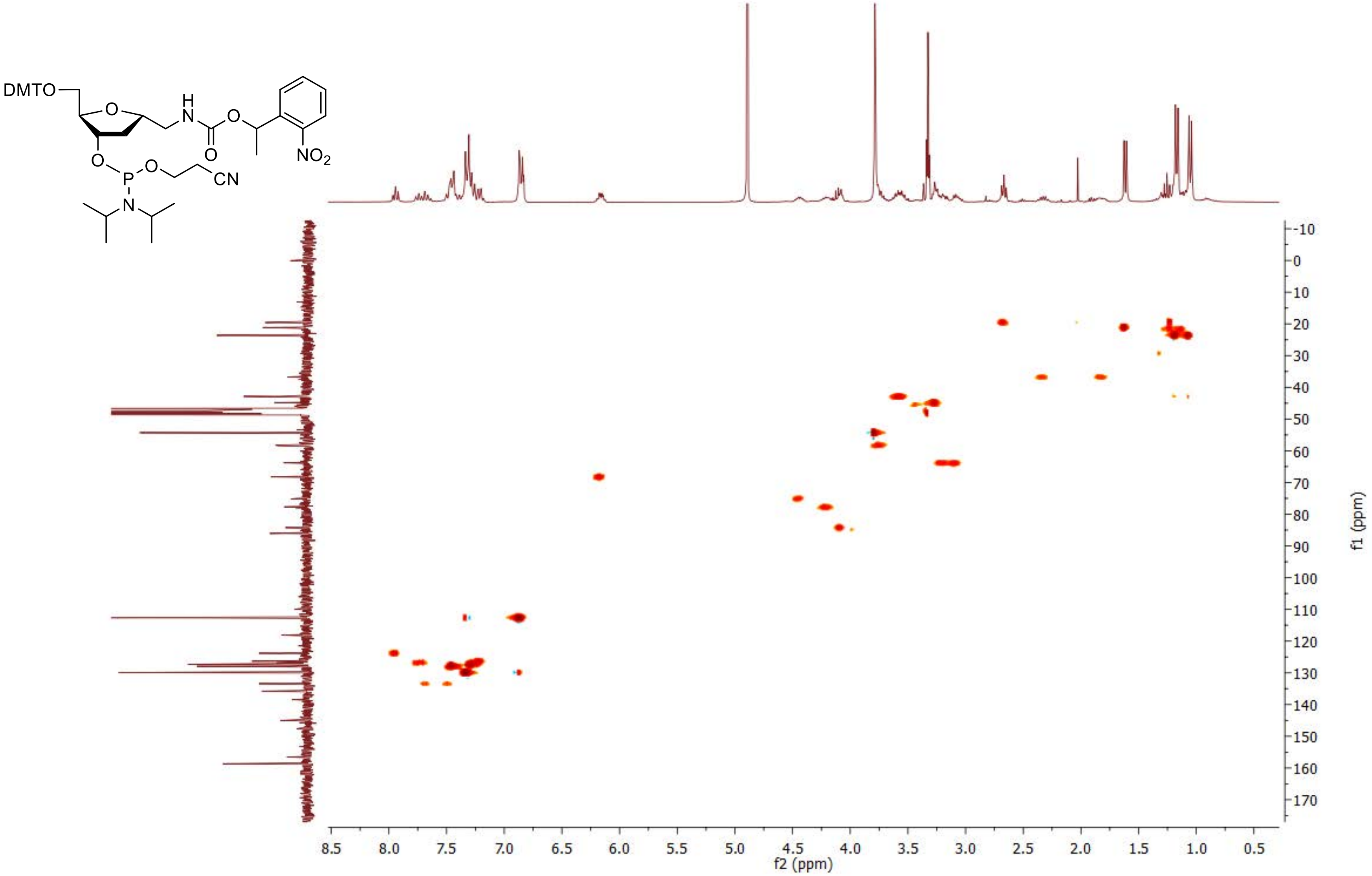

1,2-Dideoxy-5-*O*-(4,4'-dimethoxytrityl)-1α-[(1-(2-nitrophenyl)ethoxy)carbonylamino-methyl]-D-*erythro*-pentofuranosyl-3-*O*-(2-cyanoethyl-*N*,*N*-diisopropyl)phosphoramidite (8α-B)

^{31}P NMR (121.5 MHz, MeOH-d_4)

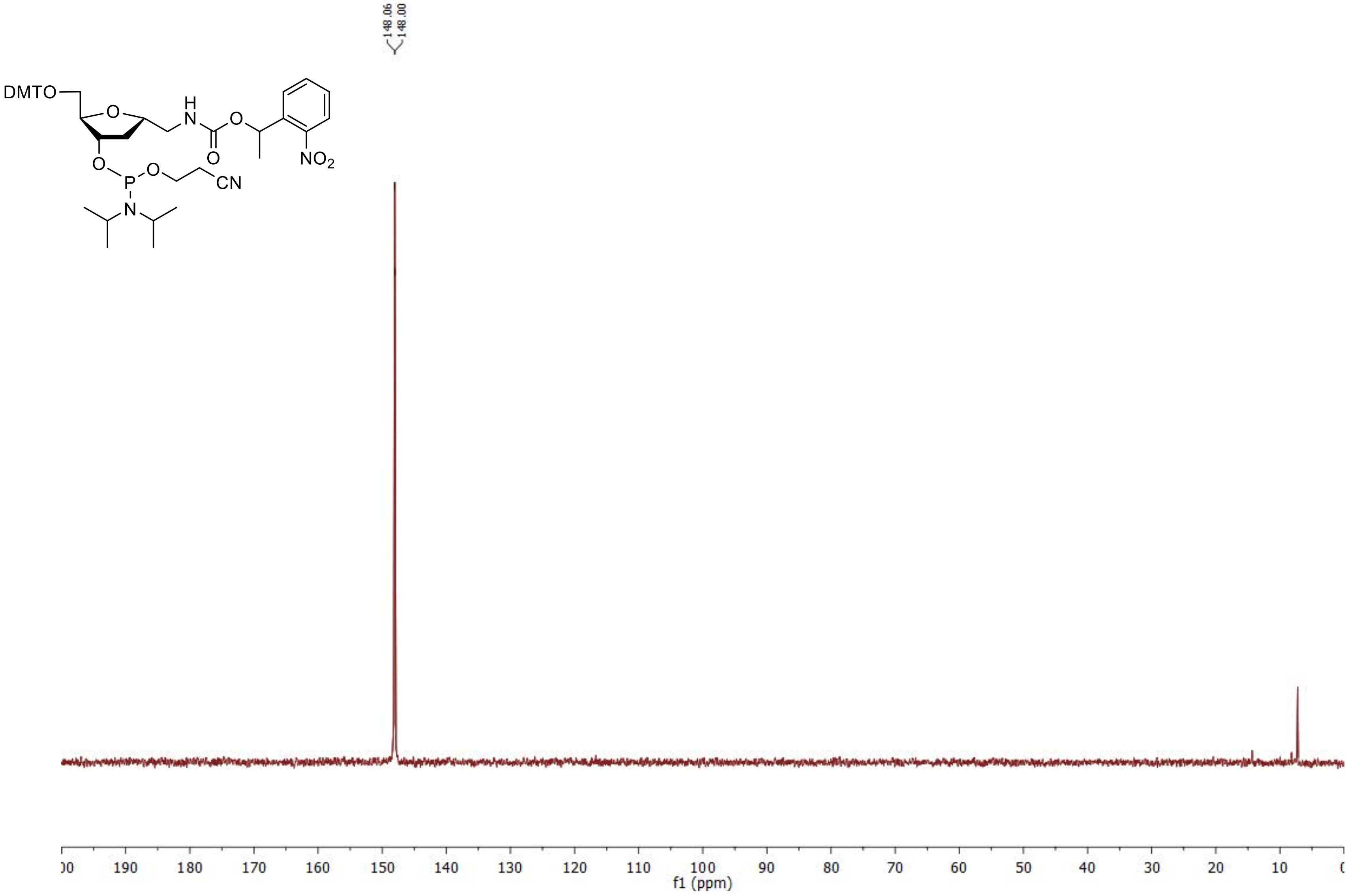

1,2-Dideoxy-5-*O*-(4,4'-dimethoxytrityl)-1β-[(1-(2-nitrophenyl)ethoxy)carbonylamino-methyl]-ᴅ-*erythro*-pentofuranosyl-3-*O*-(2-cyanoethyl-*N*,*N*-diisopropyl)phosphoramidite (8β-A)

^{1}H NMR (300.13 MHz, MeOH-d_4)

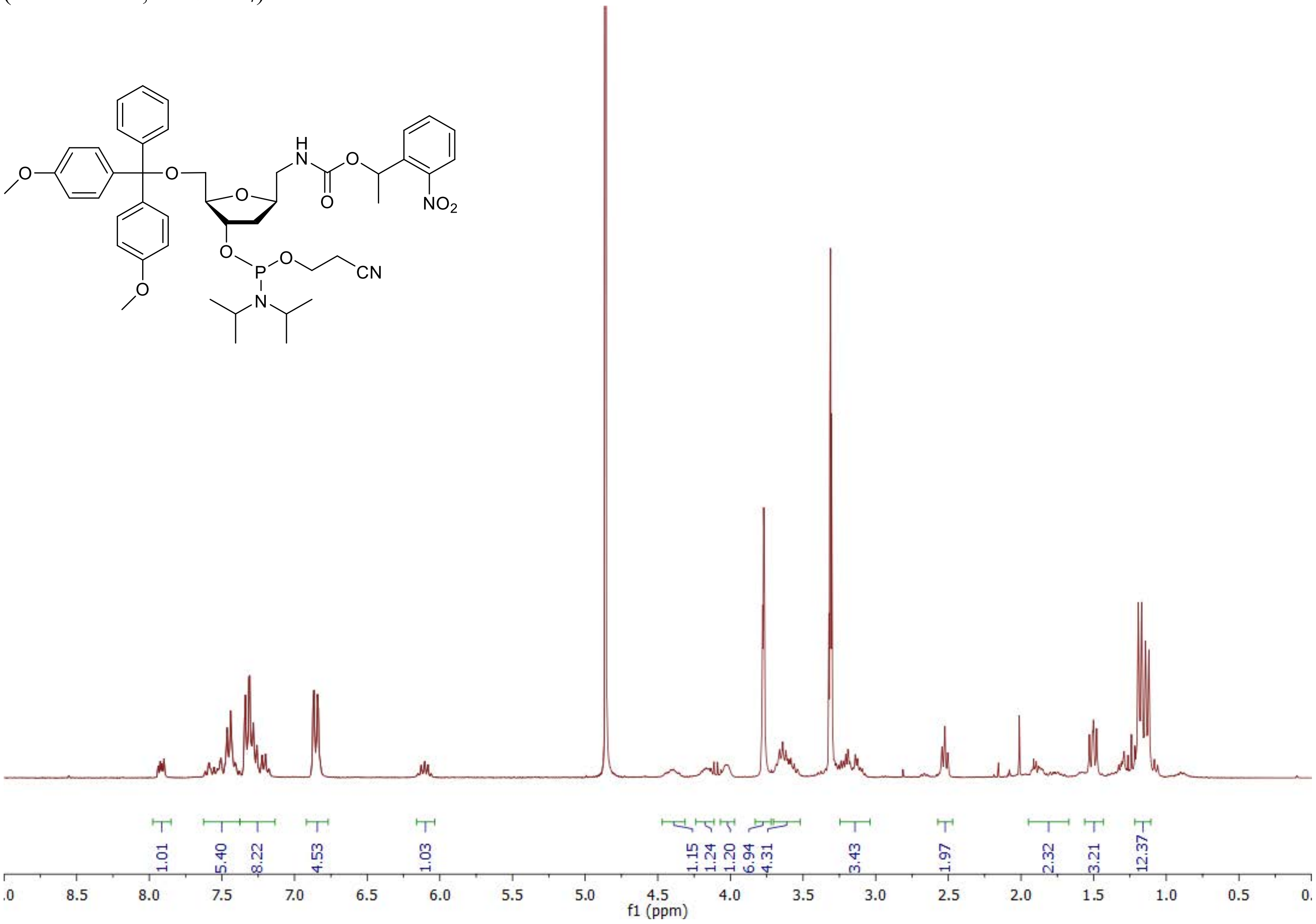

1,2-Dideoxy-5-*O*-(4,4’-dimethoxytrityl)-1β-[(1-(2-nitrophenyl)ethoxy)carbonylamino-methyl]-D-*erythro*-pentofuranosyl-3-*O*-(2-cyanoethyl-*N*,*N*-diisopropyl)phosphoramidite (8β-A)

^{13}C NMR (75.5 MHz, MeOH-d_4)

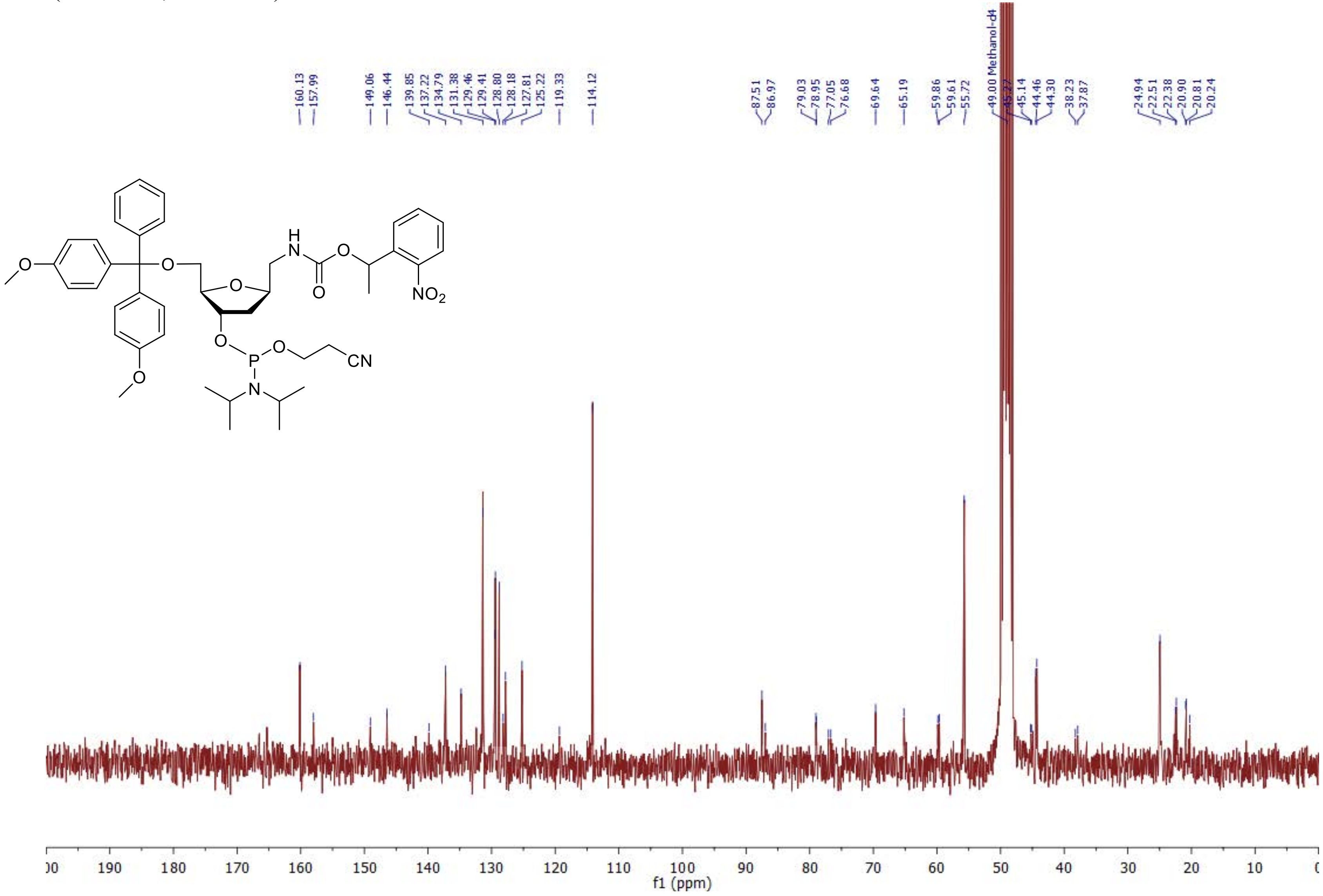

1,2-Dideoxy-5-*O*-(4,4'-dimethoxytrityl)-1β-[(1-(2-nitrophenyl)ethoxy)carbonylamino-methyl]-ᴅ-*erythro*-pentofuranosyl-3-*O*-(2-cyanoethyl-*N*,*N*-diisopropyl)phosphoramidite (8β-A)

DEPT135 NMR (75.5 MHz, MeOH-d_4)

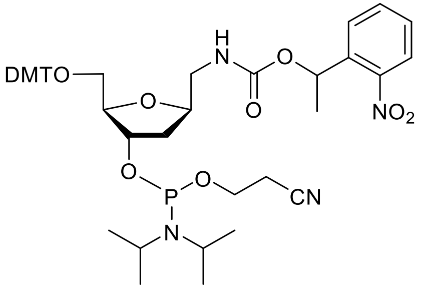

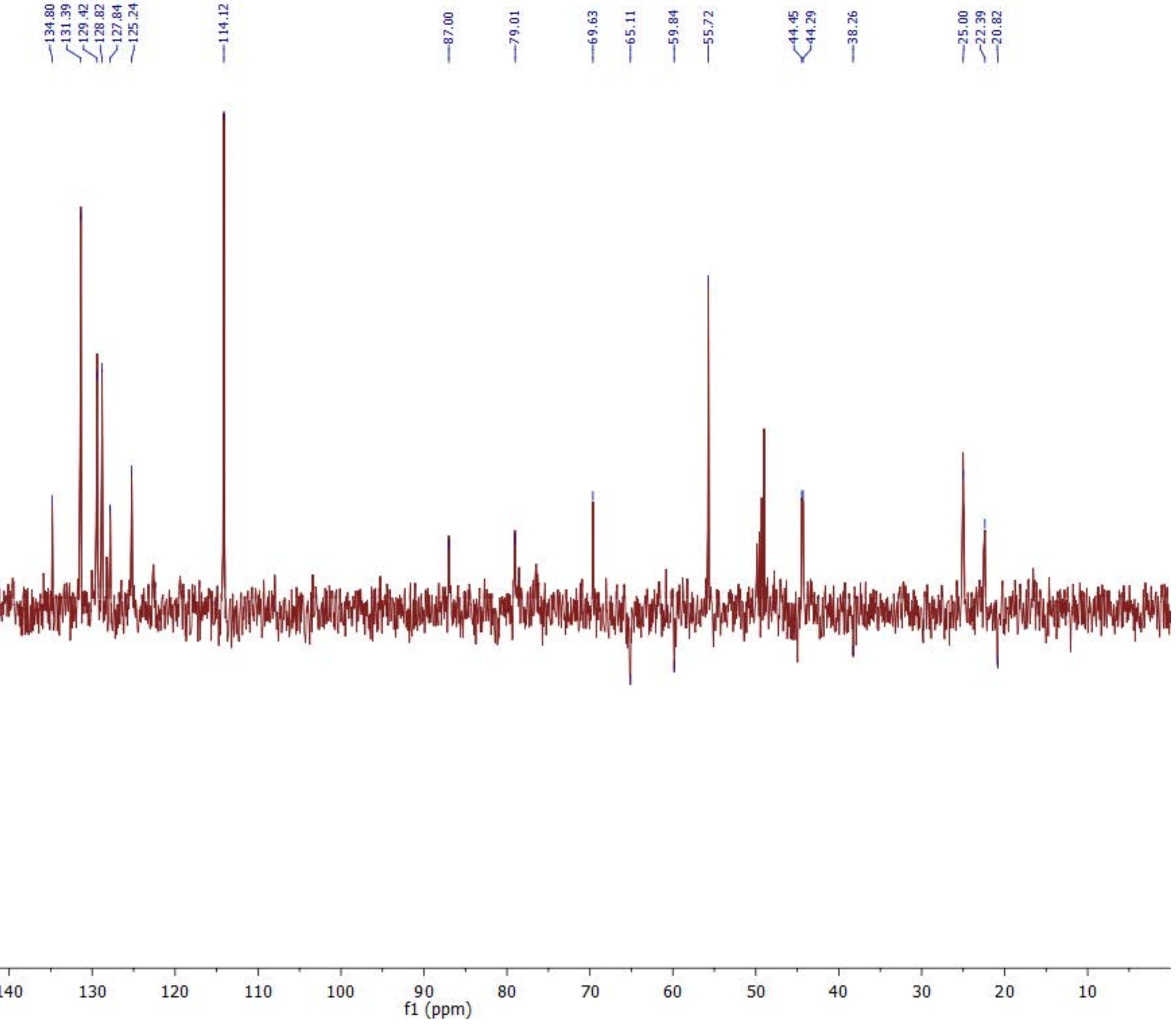

1,2-Dideoxy-5-*O*-(4,4'-dimethoxytrityl)-1β-[(1-(2-nitrophenyl)ethoxy)carbonylamino-methyl]-D-*erythro*-pentofuranosyl-3-*O*-(2-cyanoethyl-*N*,*N*-diisopropyl)phosphoramidite (8β-A)

COSY NMR (MeOH-d_4)

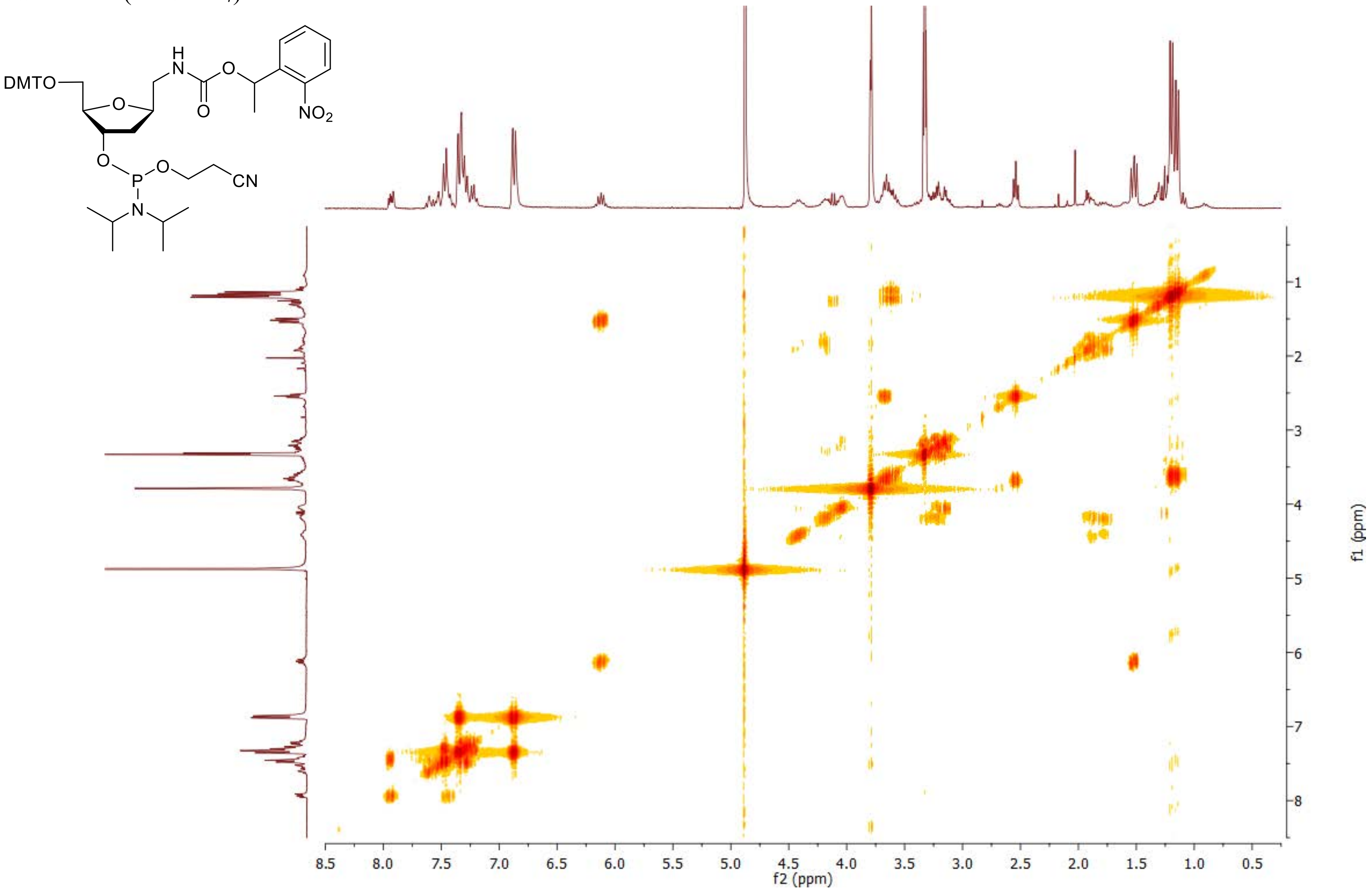

1,2-Dideoxy-5-*O*-(4,4'-dimethoxytrityl)-1β-[(1-(2-nitrophenyl)ethoxy)carbonylamino-methyl]-D-*erythro*-pentofuranosyl-3-*O*-(2-cyanoethyl-*N*,*N*-diisopropyl)phosphoramidite (8β-A)

HSQC NMR (MeOH-d_4)

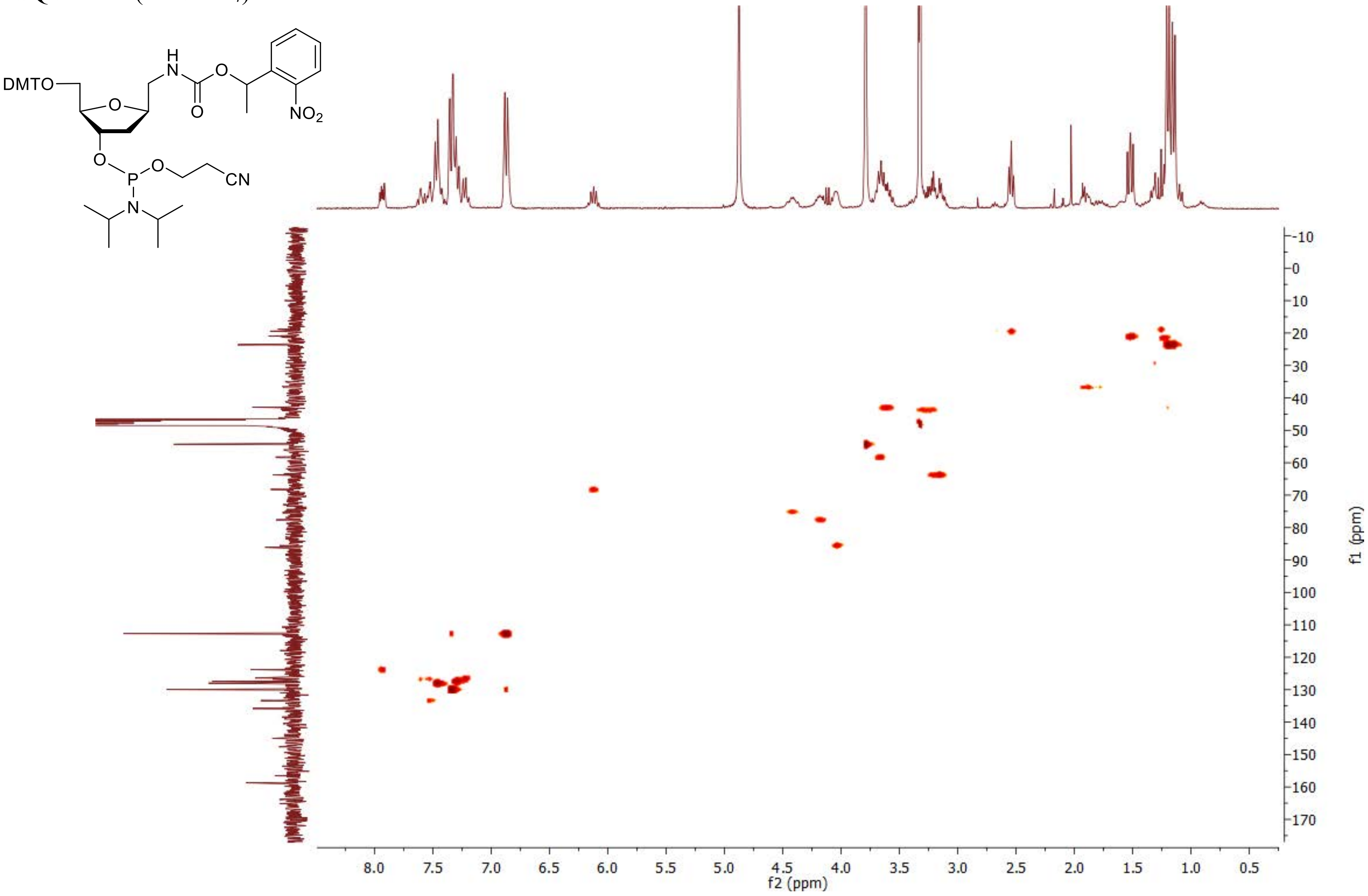

1,2-Dideoxy-5-*O*-(4,4’-dimethoxytrityl)-1β-[(1-(2-nitrophenyl)ethoxy)carbonylamino-methyl]-D-*erythro*-pentofuranosyl-3-*O*-(2-cyanoethyl-*N*,*N*-diisopropyl)phosphoramidite (8β-A)

^{31}P NMR (121.5 MHz, MeOH-d_4)

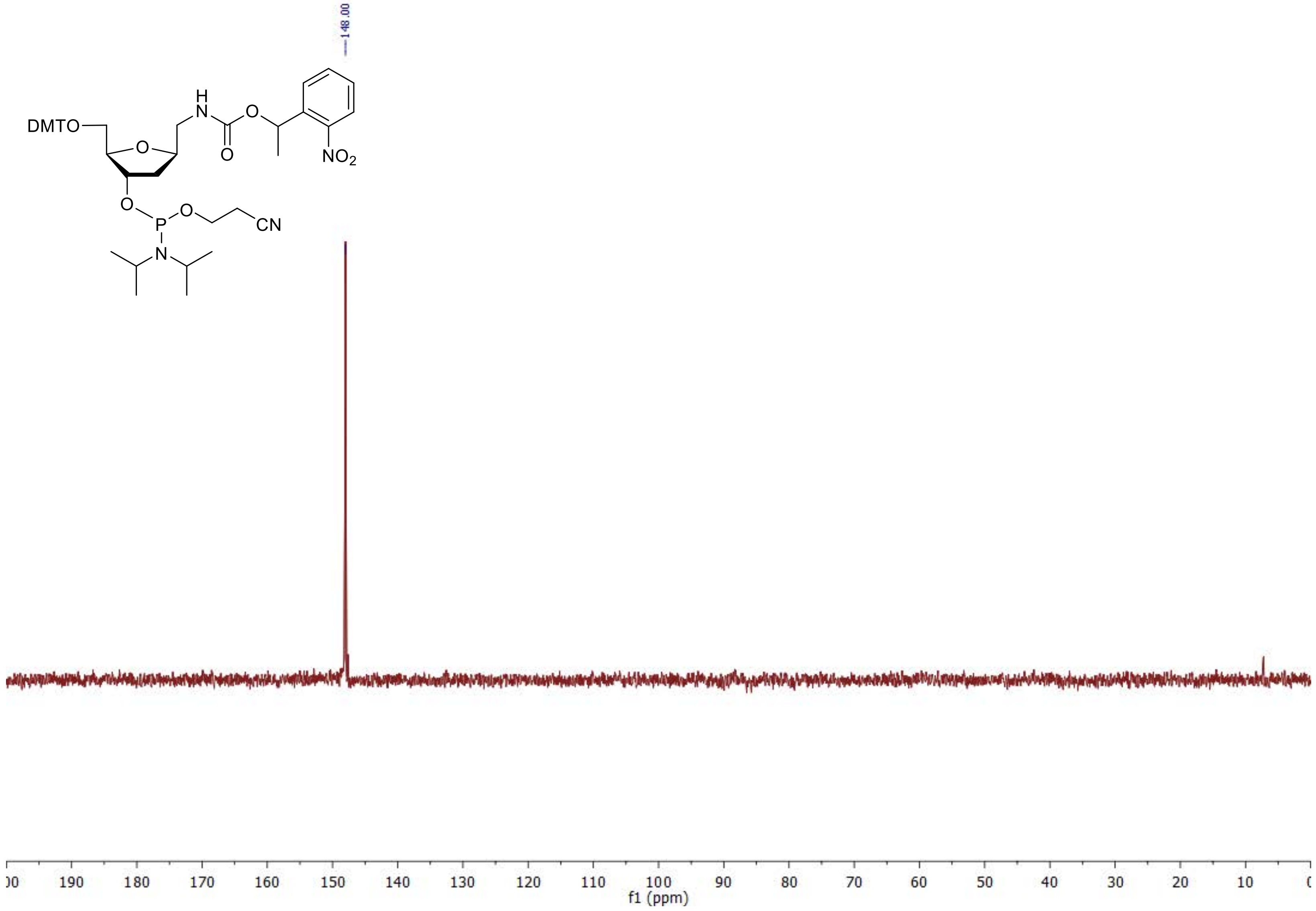

1,2-Dideoxy-5-*O*-(4,4'-dimethoxytrityl)-1β-[(1-(2-nitrophenyl)ethoxy)carbonylamino-methyl]-ᴅ-*erythro*-pentofuranosyl-3-*O*-(2-cyanoethyl-*N*,*N*-diisopropyl)phosphoramidite (8β-B)

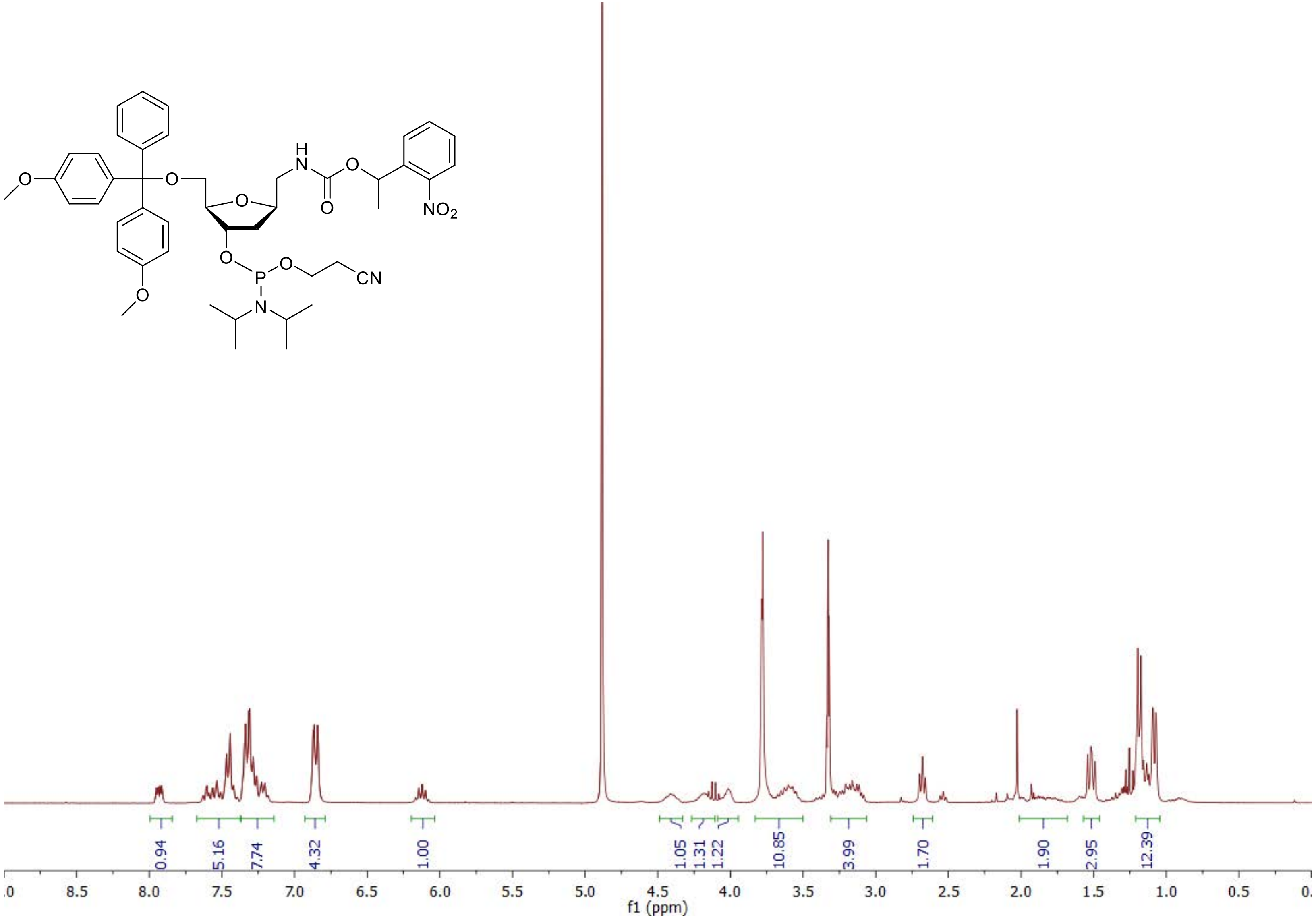

1,2-Dideoxy-5-*O*-(4,4'-dimethoxytrityl)-1β-[(1-(2-nitrophenyl)ethoxy)carbonylamino-methyl]-ᴅ-*erythro*-pentofuranosyl-3-*O*-(2-cyanoethyl-*N*,*N*-diisopropyl)phosphoramidite (8β-B)

^{13}C NMR (75.5 MHz, MeOH-d_4)

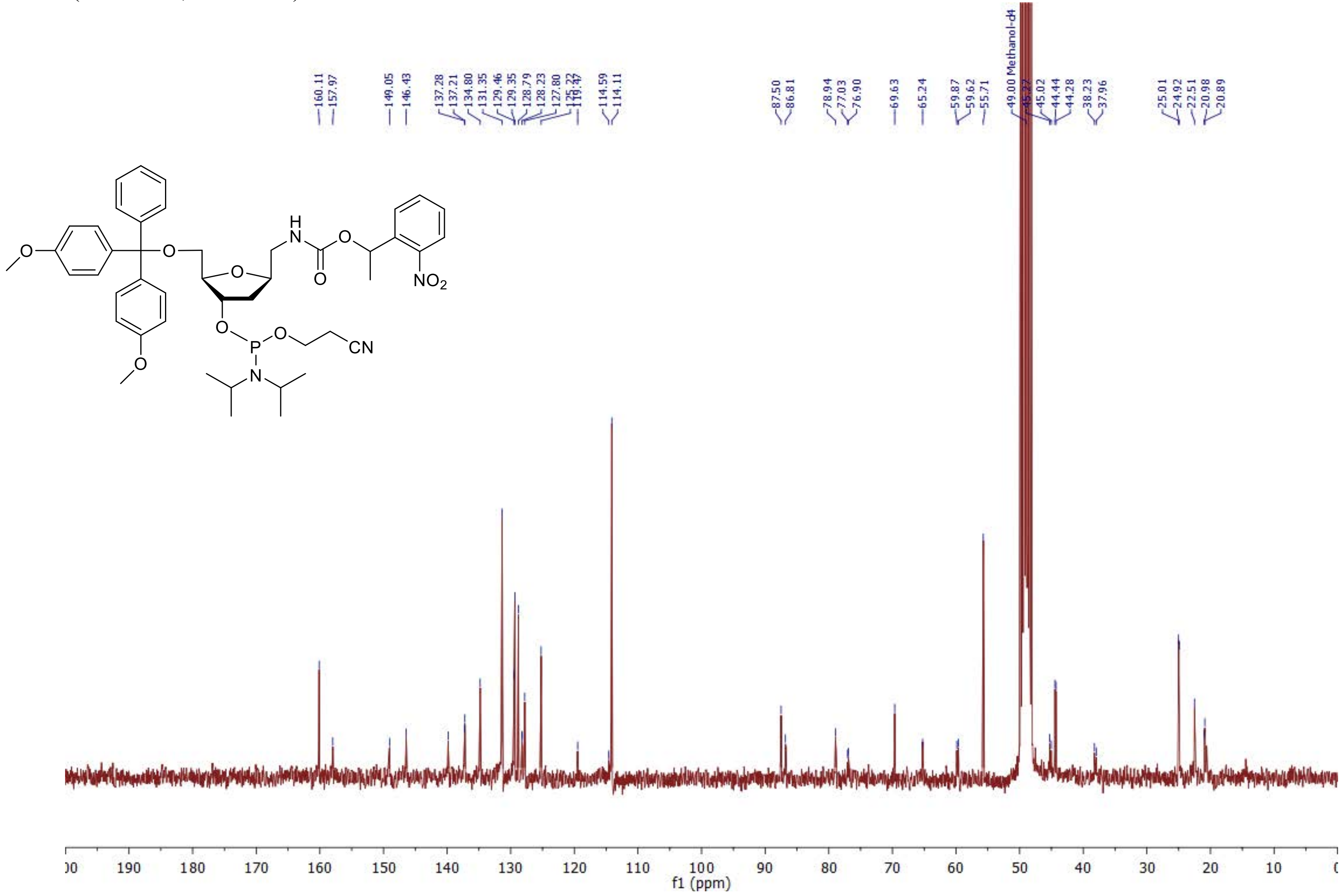

1,2-Dideoxy-5-*O*-(4,4'-dimethoxytrityl)-1β-[(1-(2-nitrophenyl)ethoxy)carbonylamino-methyl]-ᴅ-*erythro*-pentofuranosyl-3-*O*-(2-cyanoethyl-*N*,*N*-diisopropyl)phosphoramidite (8β-B)

DEPT135 NMR (75.5 MHz, MeOH-d_4)

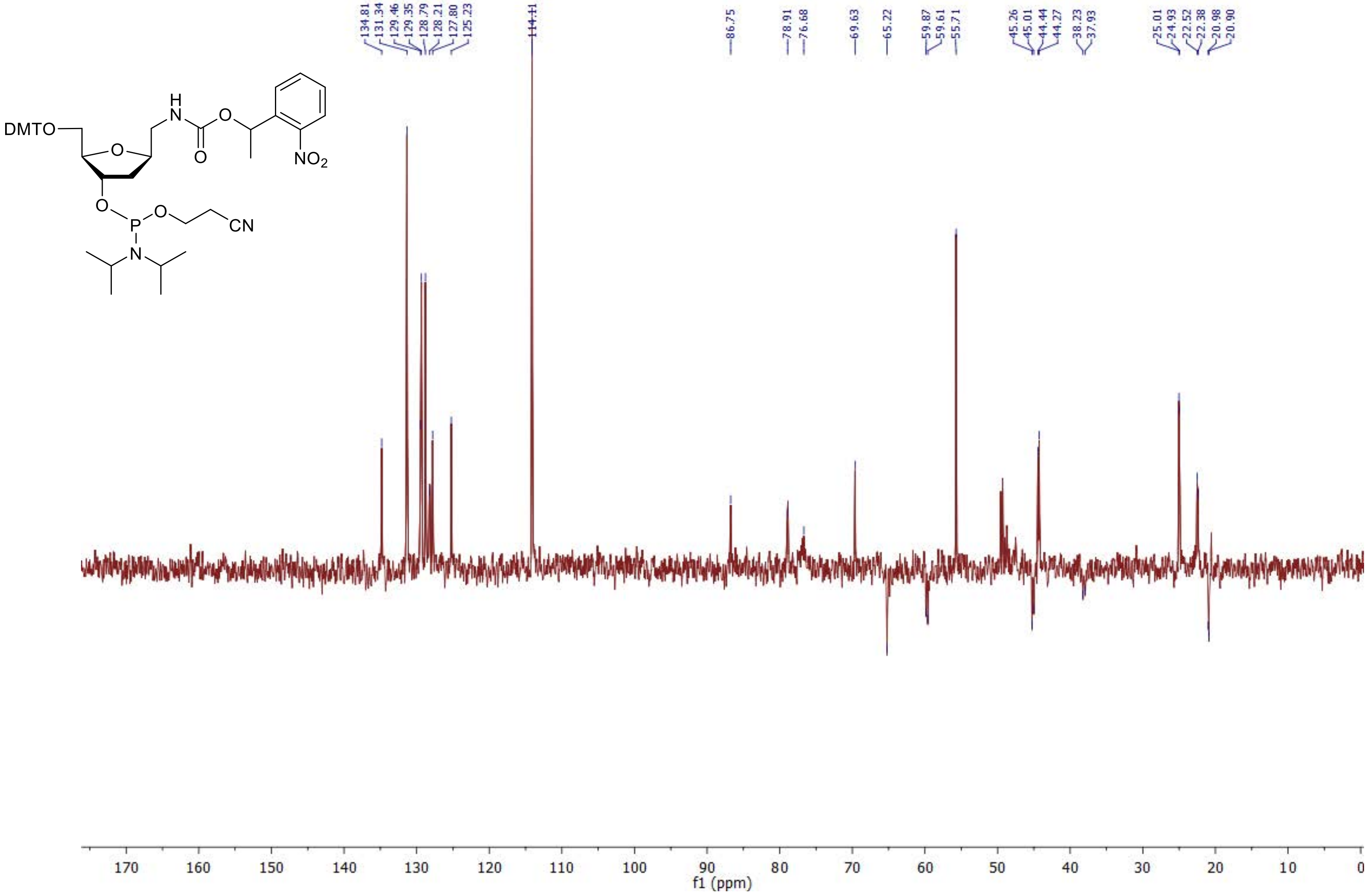

1,2-Dideoxy-5-*O*-(4,4'-dimethoxytrityl)-1β-[(1-(2-nitrophenyl)ethoxy)carbonylamino-methyl]-ᴅ-*erythro*-pentofuranosyl-3-*O*-(2-cyanoethyl-*N*,*N*-diisopropyl)phosphoramidite (8β-B)

COSY NMR (MeOH-d_4)

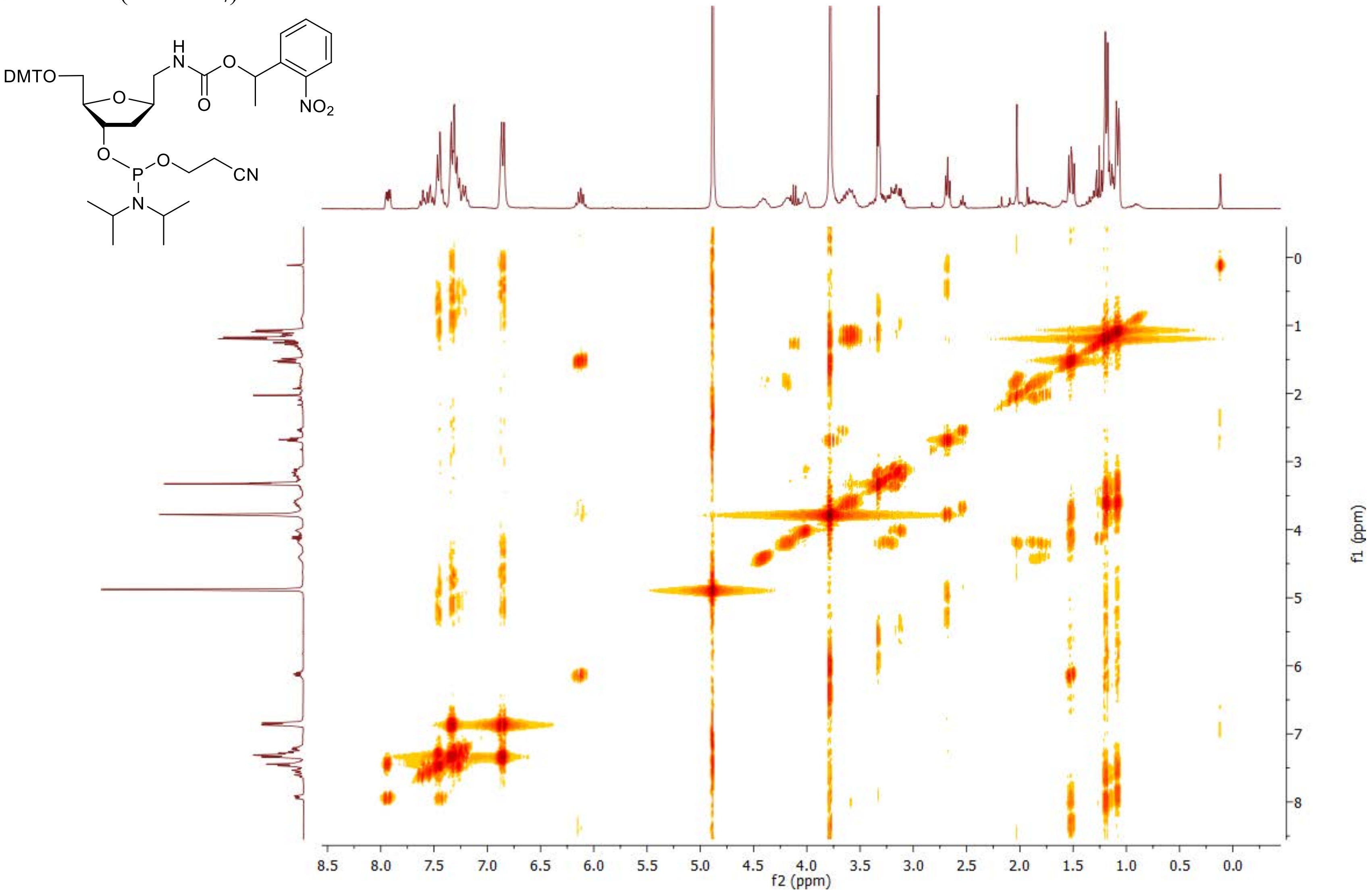

1,2-Dideoxy-5-*O*-(4,4'-dimethoxytrityl)-1β-[(1-(2-nitrophenyl)ethoxy)carbonylamino-methyl]-D-*erythro*-pentofuranosyl-3-*O*-(2-cyanoethyl-*N*,*N*-diisopropyl)phosphoramidite (8β-B)

HSQC NMR (MeOH-d_4)

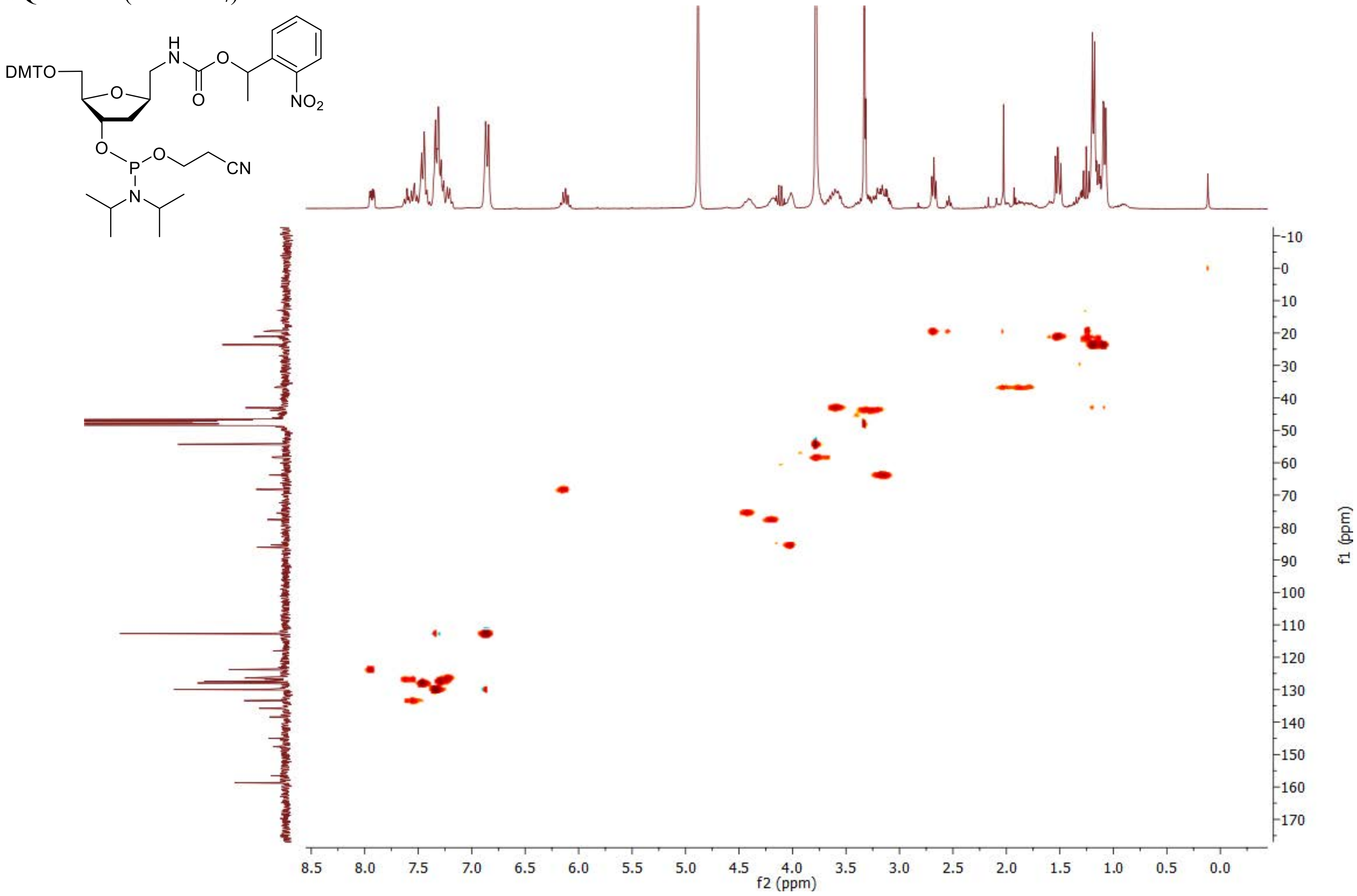

1,2-Dideoxy-5-*O*-(4,4'-dimethoxytrityl)-1β-[(1-(2-nitrophenyl)ethoxy)carbonylamino-methyl]-D-*erythro*-pentofuranosyl-3-*O*-(2-cyanoethyl-*N*,*N*-diisopropyl)phosphoramidite (8β-B)

^{31}P NMR (121.5 MHz, MeOH-d_4)

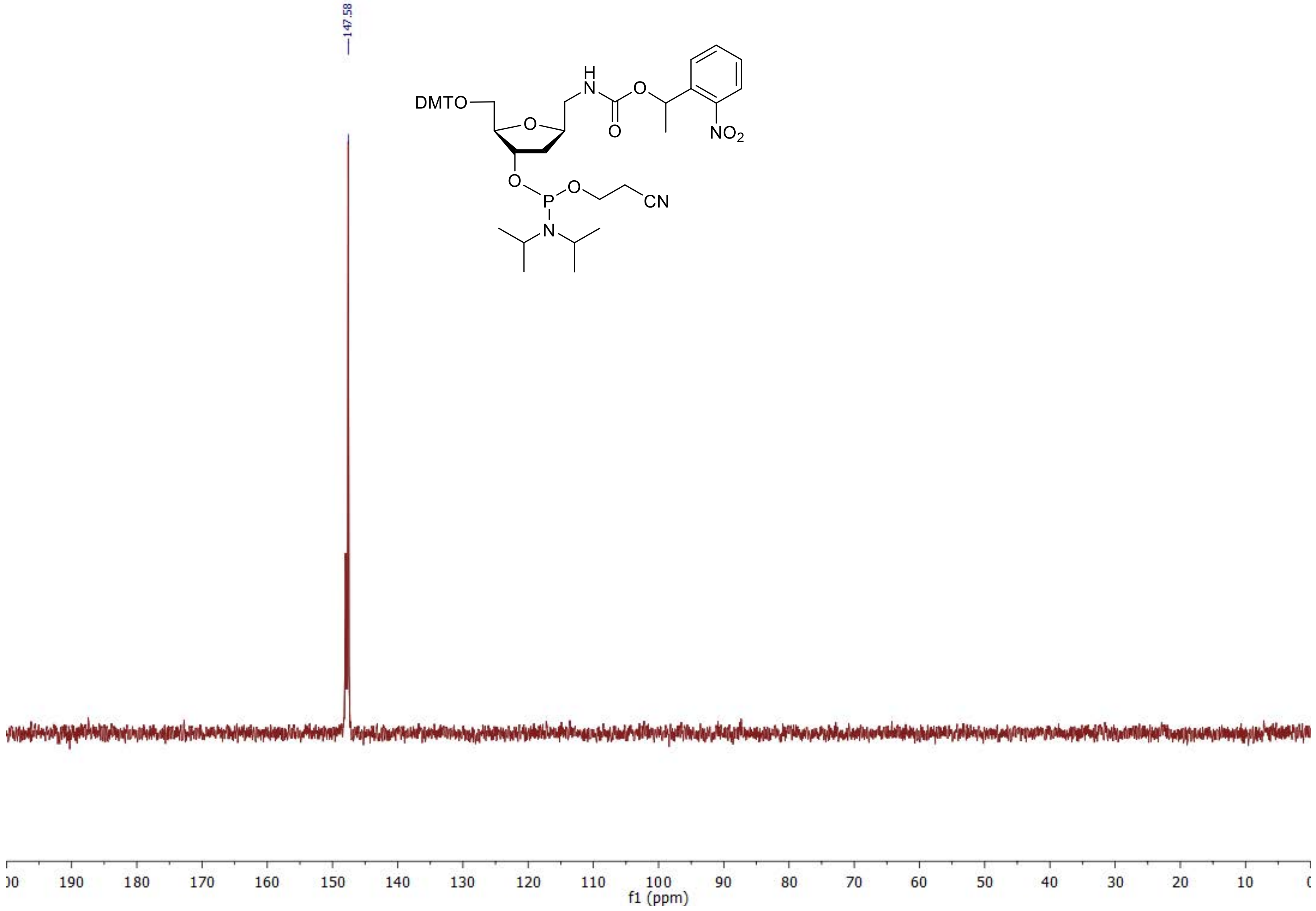

1,2-Dideoxy-1β-(methoxycarbonyl)-D-*erythro*-pentofuranose (9β)

^{1}H NMR (300.3 MHz, MeOH-d_4)

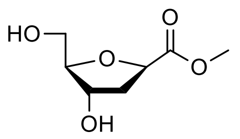

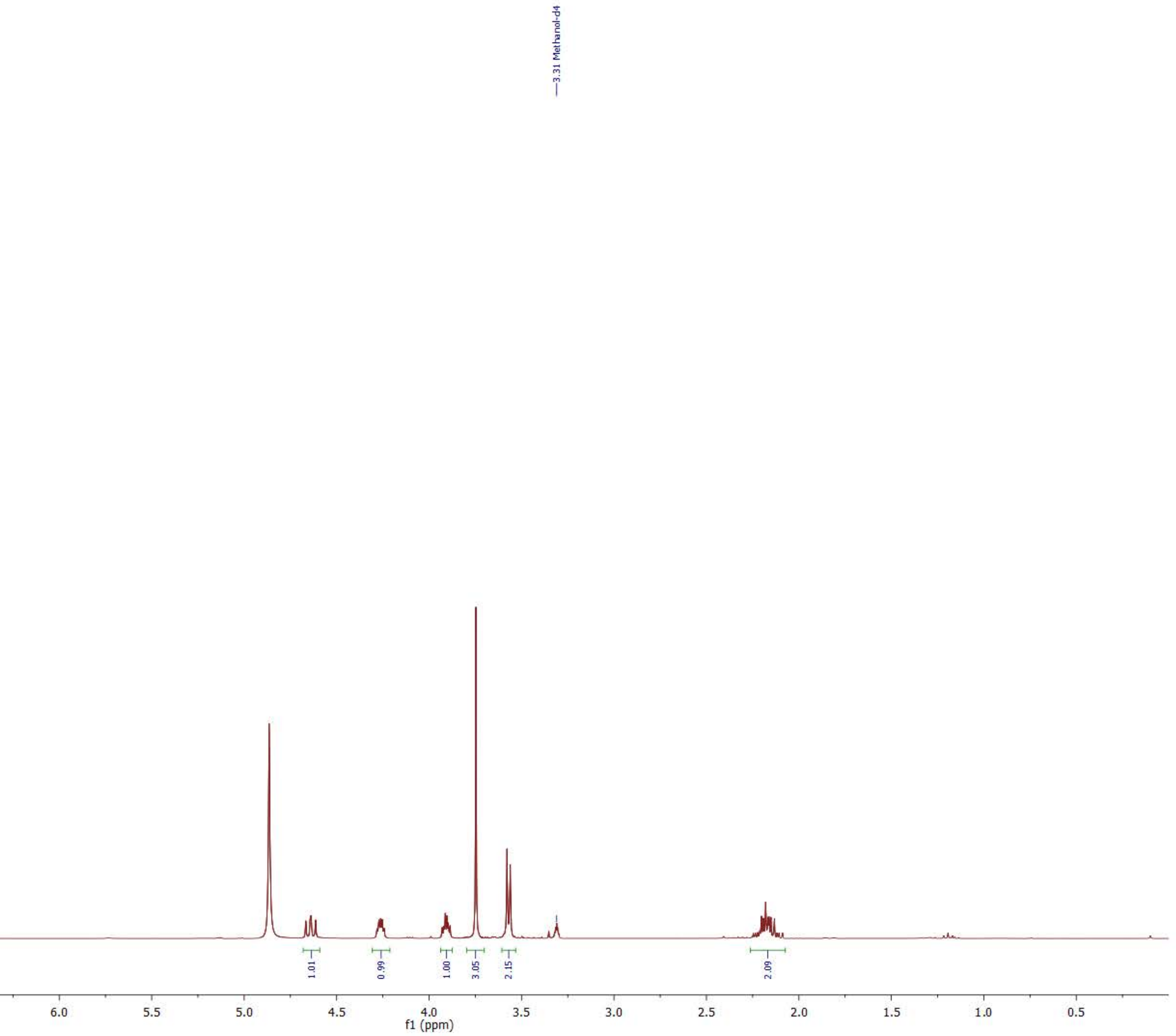

1,2-Dideoxy-1β-(methoxycarbonyl)-D-*erythro*-pentofuranose (9β)

^{13}C NMR (75.5 MHz, MeOH-d_4)

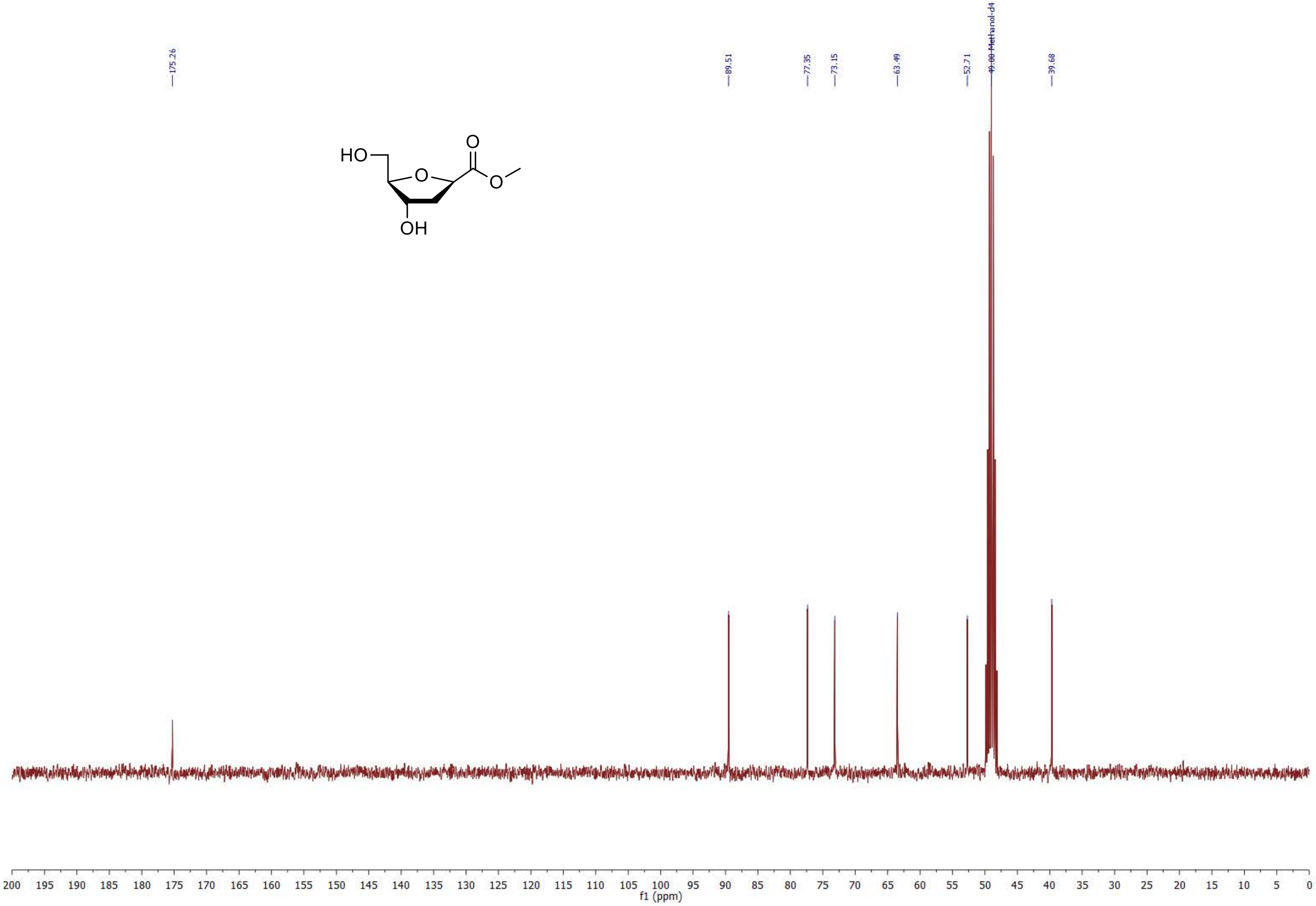

1,2-Dideoxy-1β-(methoxycarbonyl)-D-*erythro*-pentofuranose (9β)

DEPT 135 NMR (75.5 MHz, MeOH-d_4)

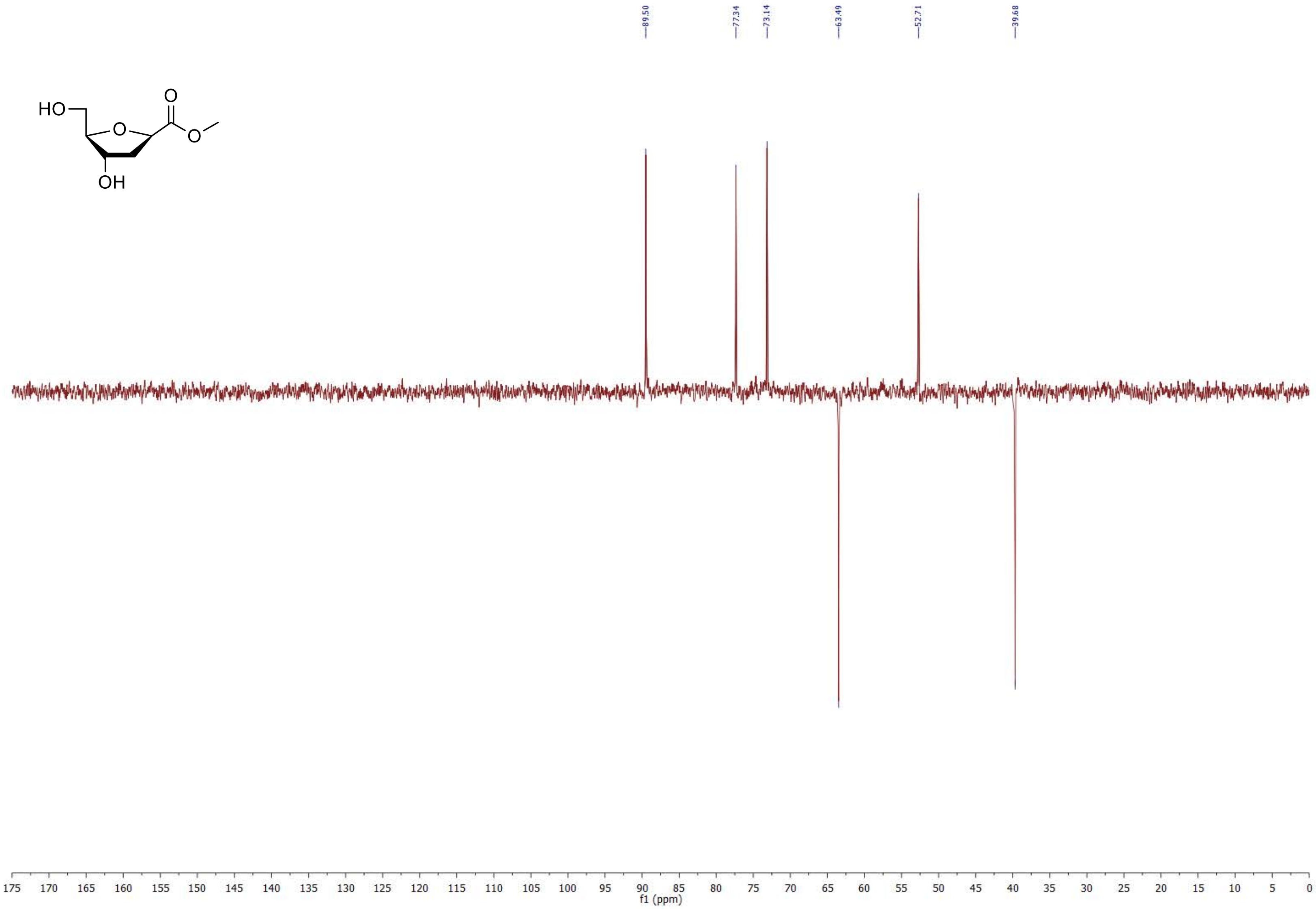

1,2-Dideoxy-1β-(methoxycarbonyl)-D-*erythro*-pentofuranose (9β)

COSY NMR (MeOH-d_4)

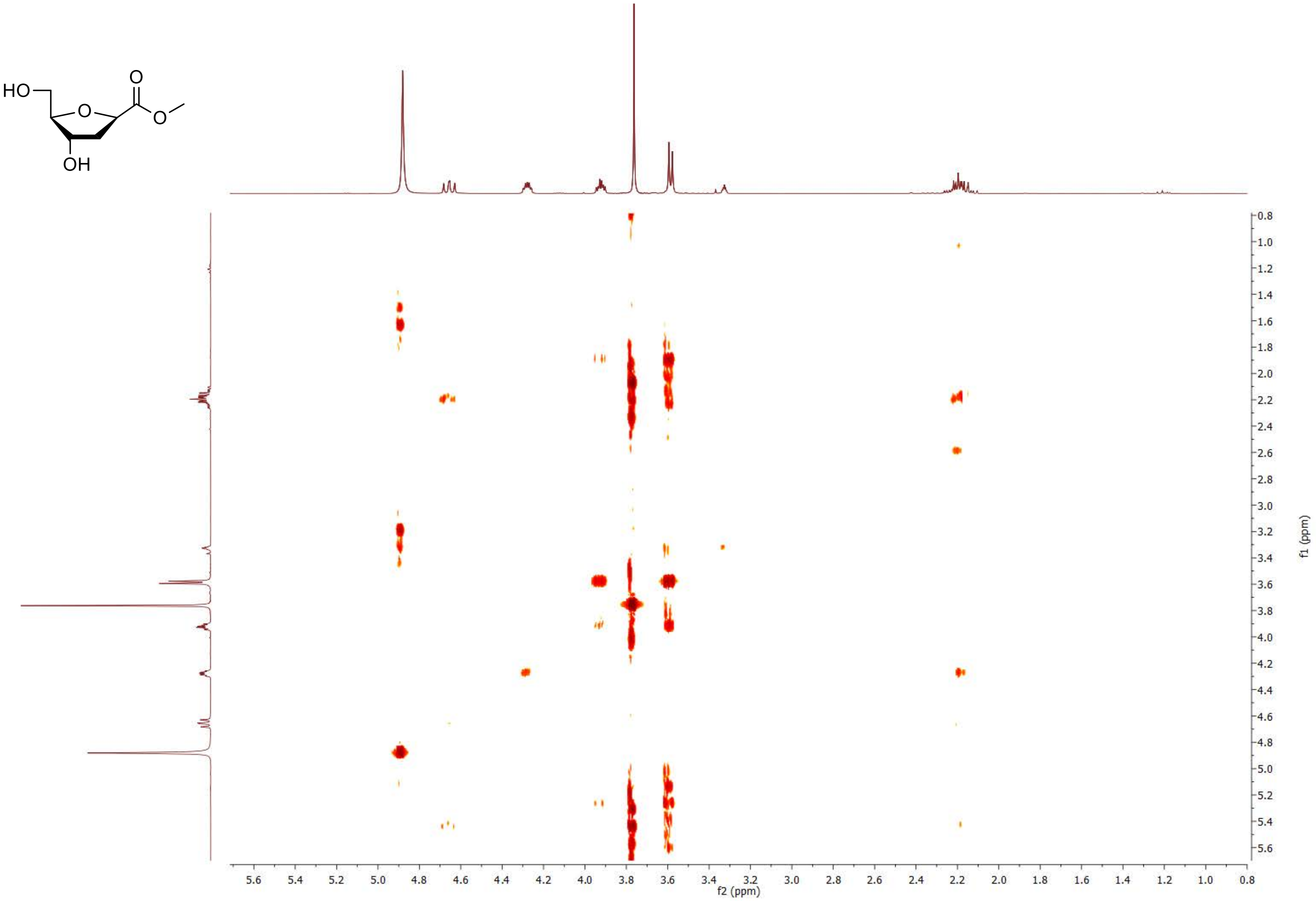

1,2-Dideoxy-1β-(methoxycarbonyl)-D-*erythro*-pentofuranose (9β)

HSQC NMR (MeOH-d_4)

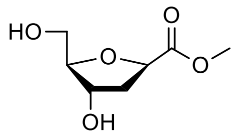

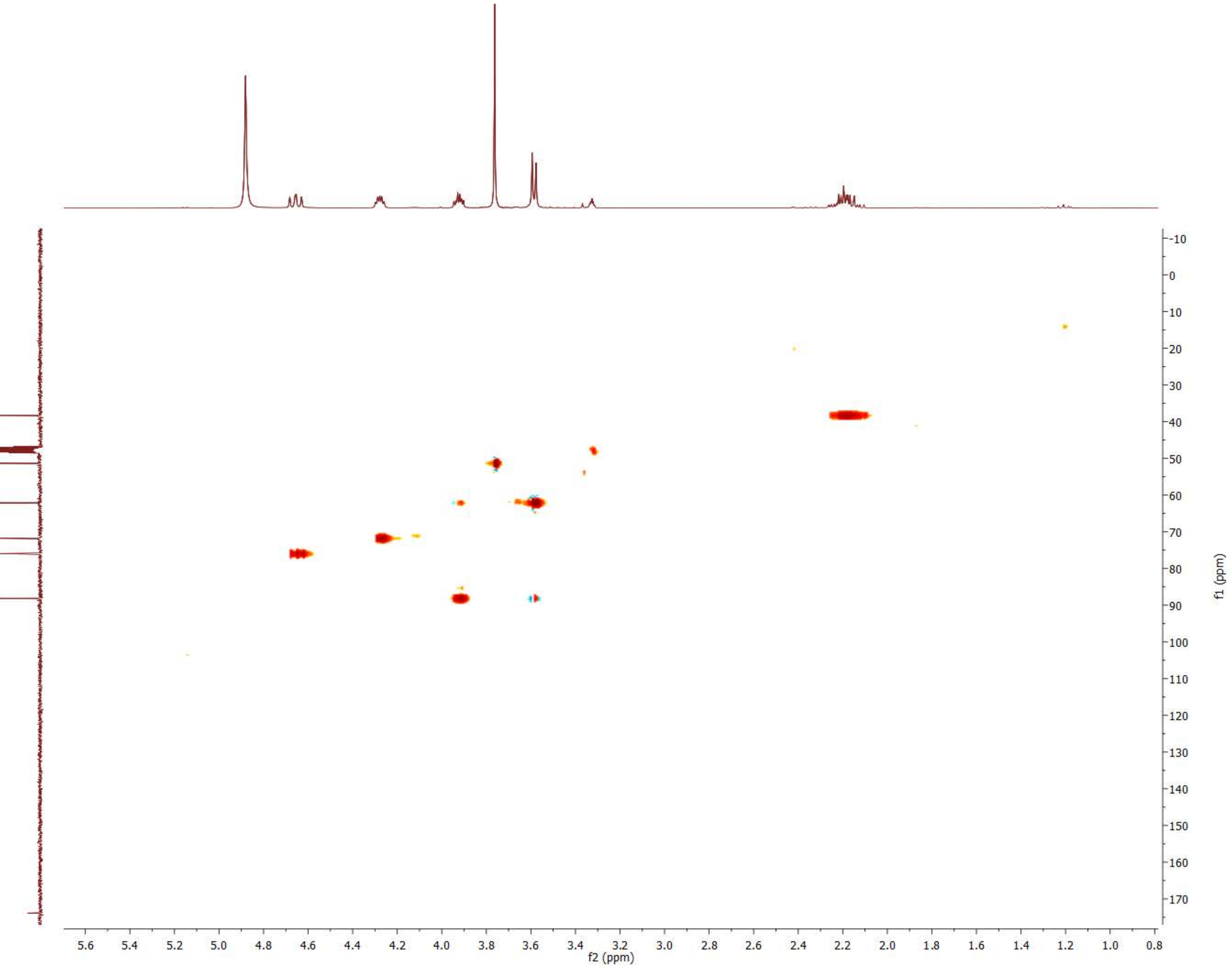

3,5-bis-*O*-(*tert*-Butyldimethylsilyl)-1,2-dideoxy-1β-(methoxycarbonyl)-D-*erythro*-pentofuranose (10β)

^{1}H NMR (300.3 MHz, MeOH-d_4)

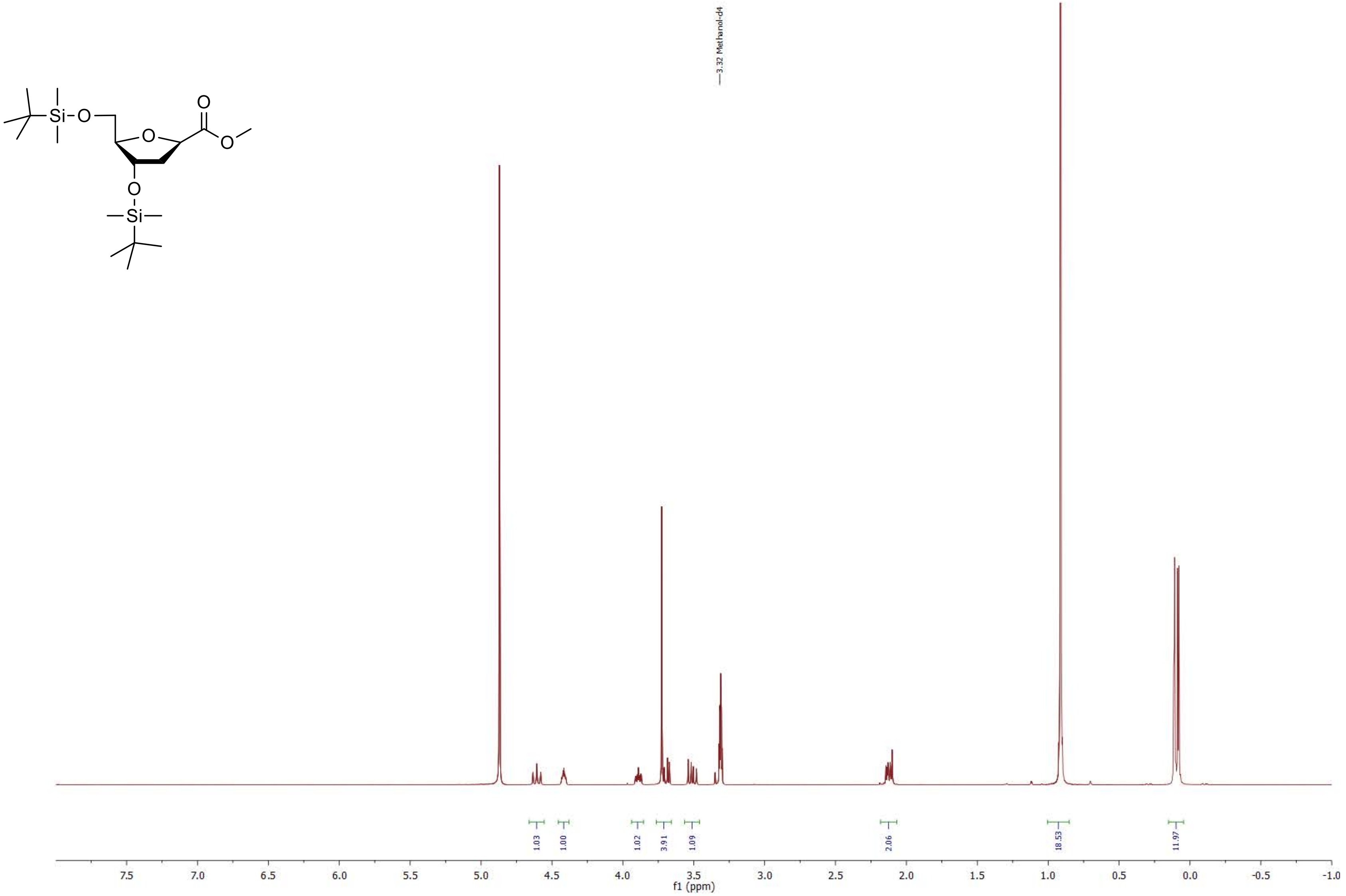

3,5-bis-*O*-(*tert*-Butyldimethylsilyl)-1,2-dideoxy-1β-(methoxycarbonyl)-D-*erythro*-pentofuranose (10β)

^{13}C NMR (75.5 MHz, MeOH-d_4)

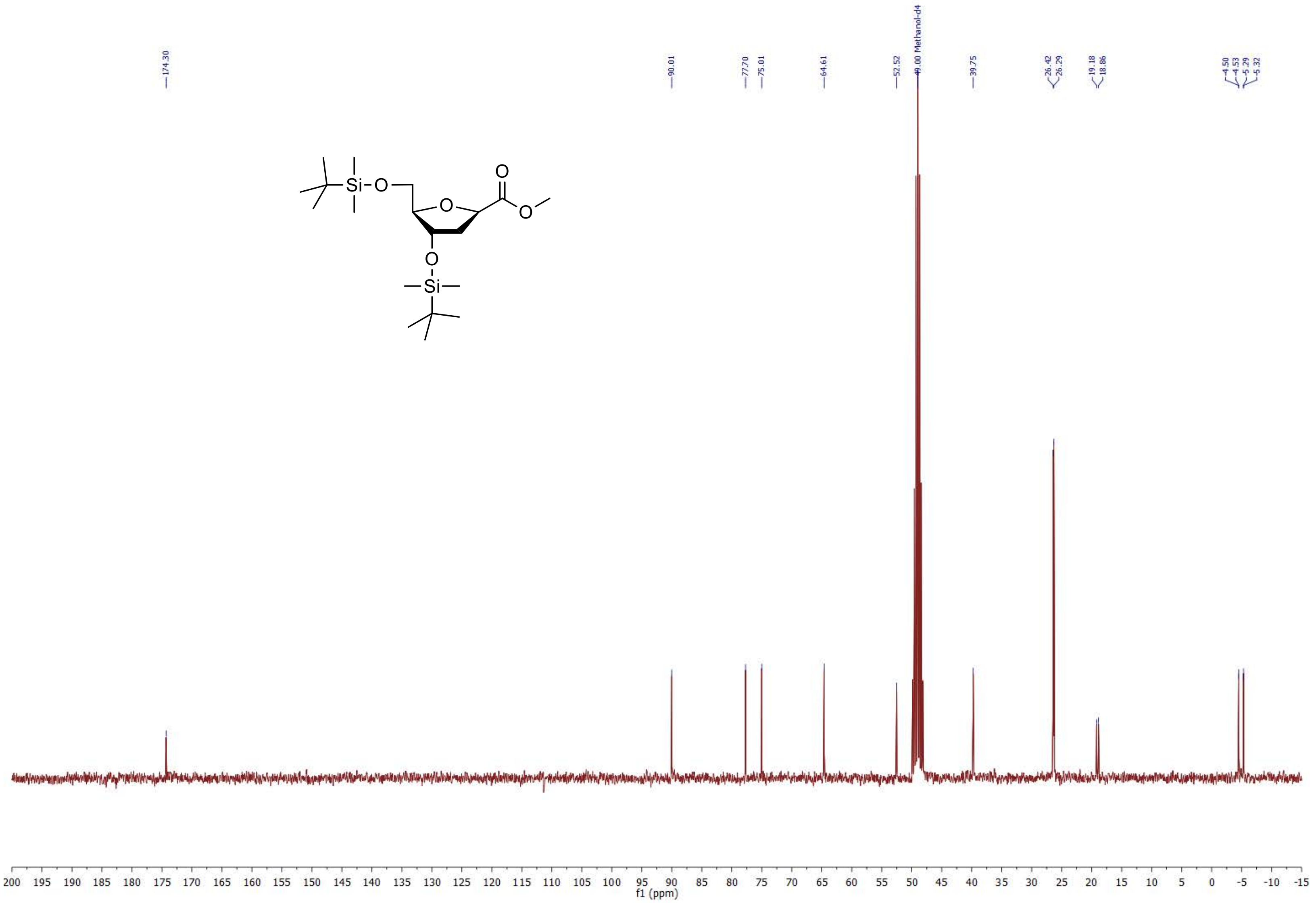

3,5-bis-*O*-(*tert*-Butyldimethylsilyl)-1,2-dideoxy-1β-(methoxycarbonyl)-D-*erythro*-pentofuranose (10β)

DEPT 135 NMR (75.5 MHz, MeOH-d_4)

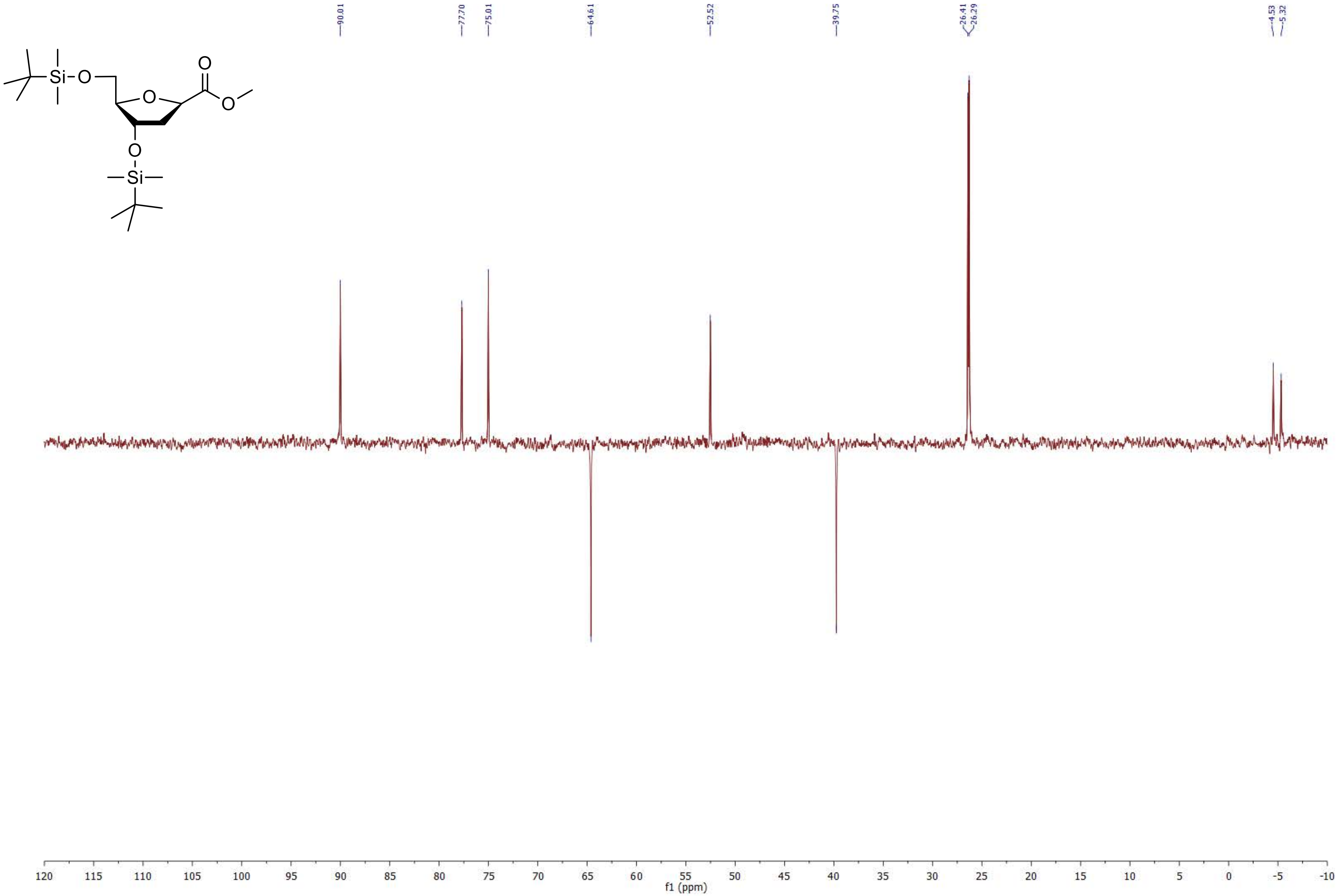

3,5-bis-*O*-(*tert*-Butyldimethylsilyl)-1,2-dideoxy-1β-(methoxycarbonyl)-ᴅ-*erythro*-pentofuranose (10β)

COSY NMR (MeOH-d_4)

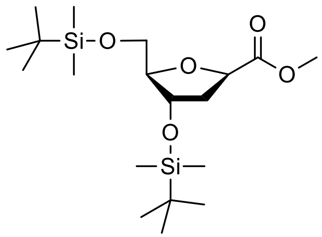

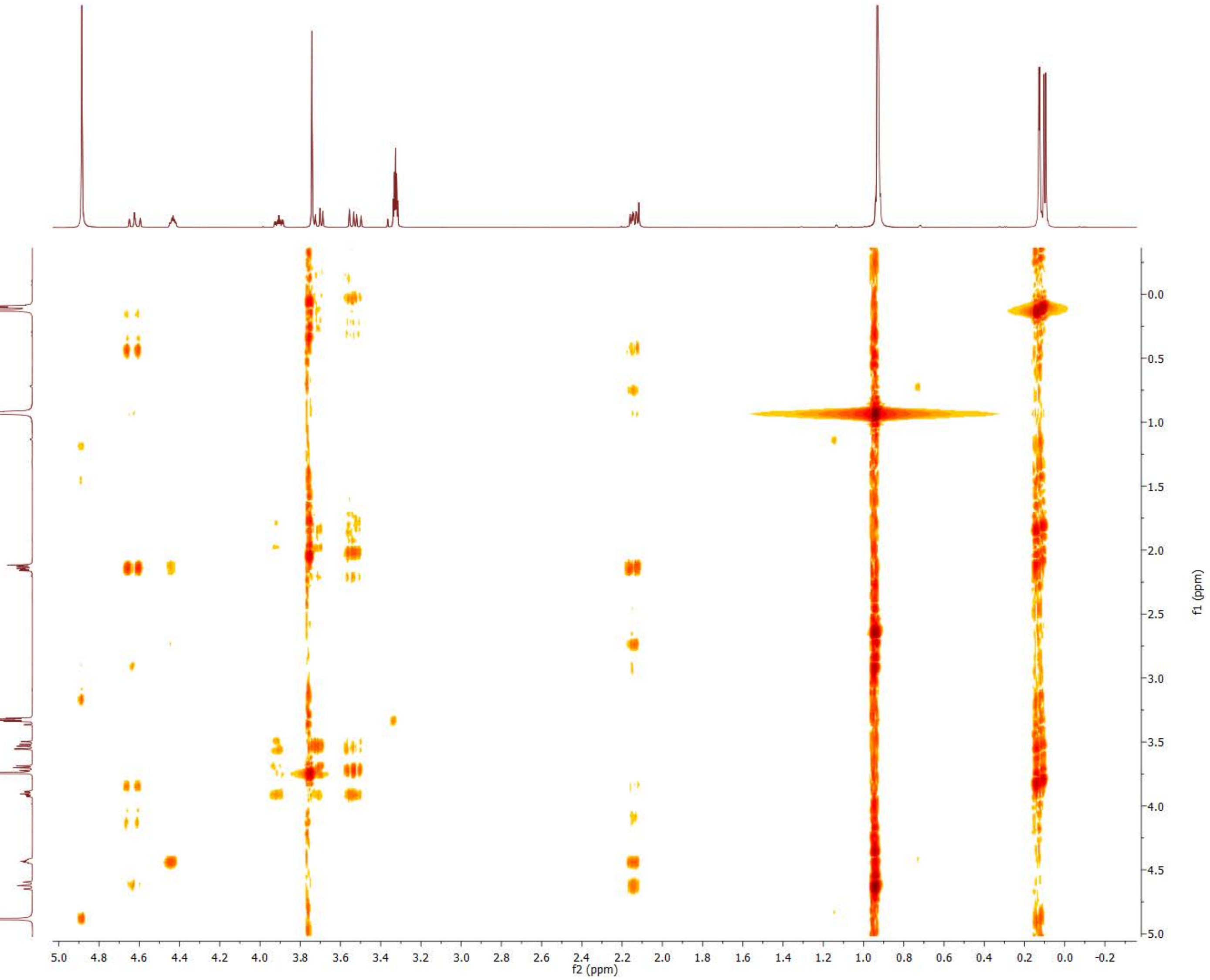

3,5-bis-*O*-(*tert*-Butyldimethylsilyl)-1,2-dideoxy-1β-(methoxycarbonyl)-ᴅ-*erythro*-pentofuranose (10β)

HSQC NMR (MeOH-d_4)

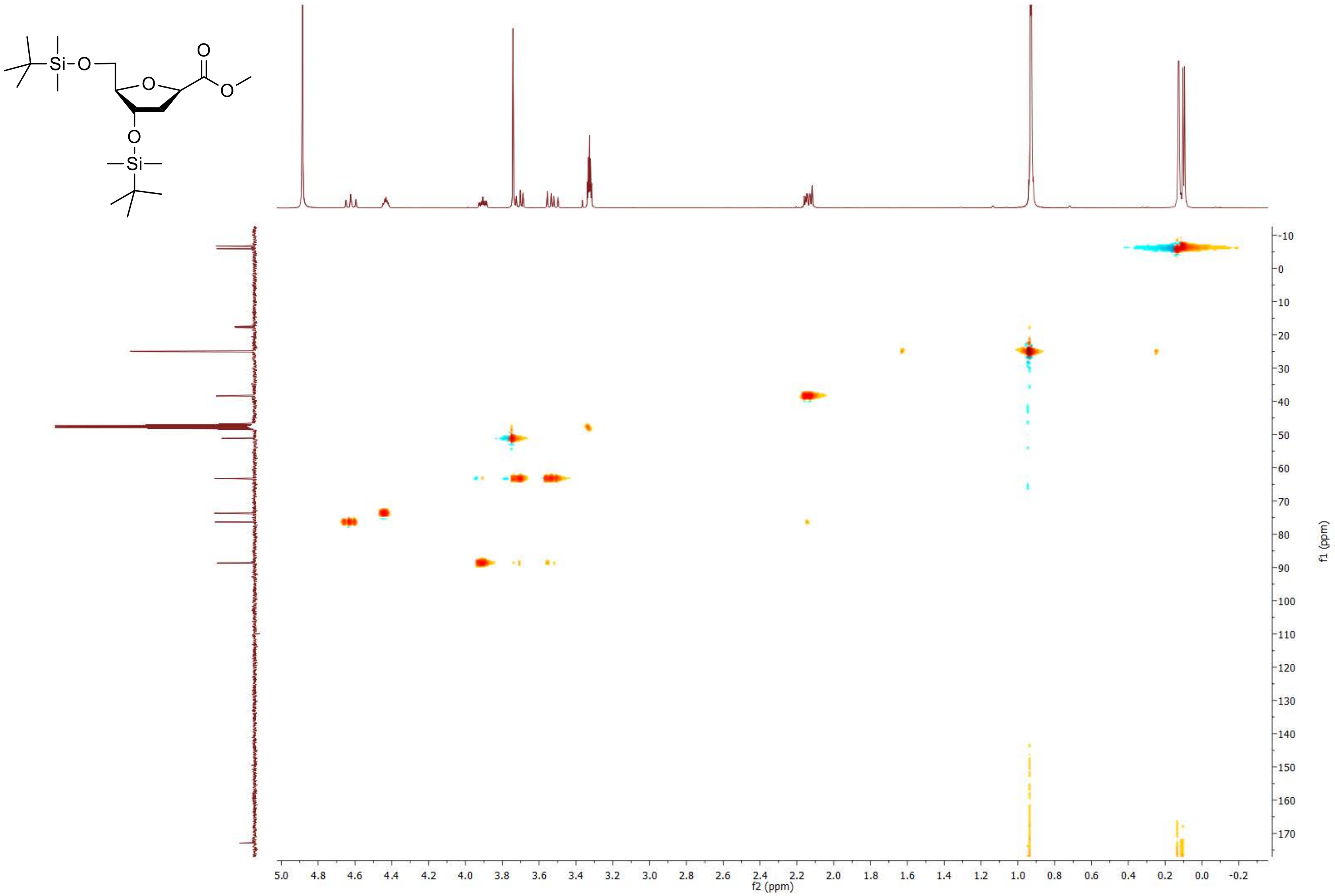

3,5-bis-*O*-(*tert*-Butyldimethylsilyl)-1,2-dideoxy-1β-(hydroxymethyl)-D-*erythro*-pentofuranose (11β)

^{1}H NMR (300.13 MHz, MeOH-d_4)

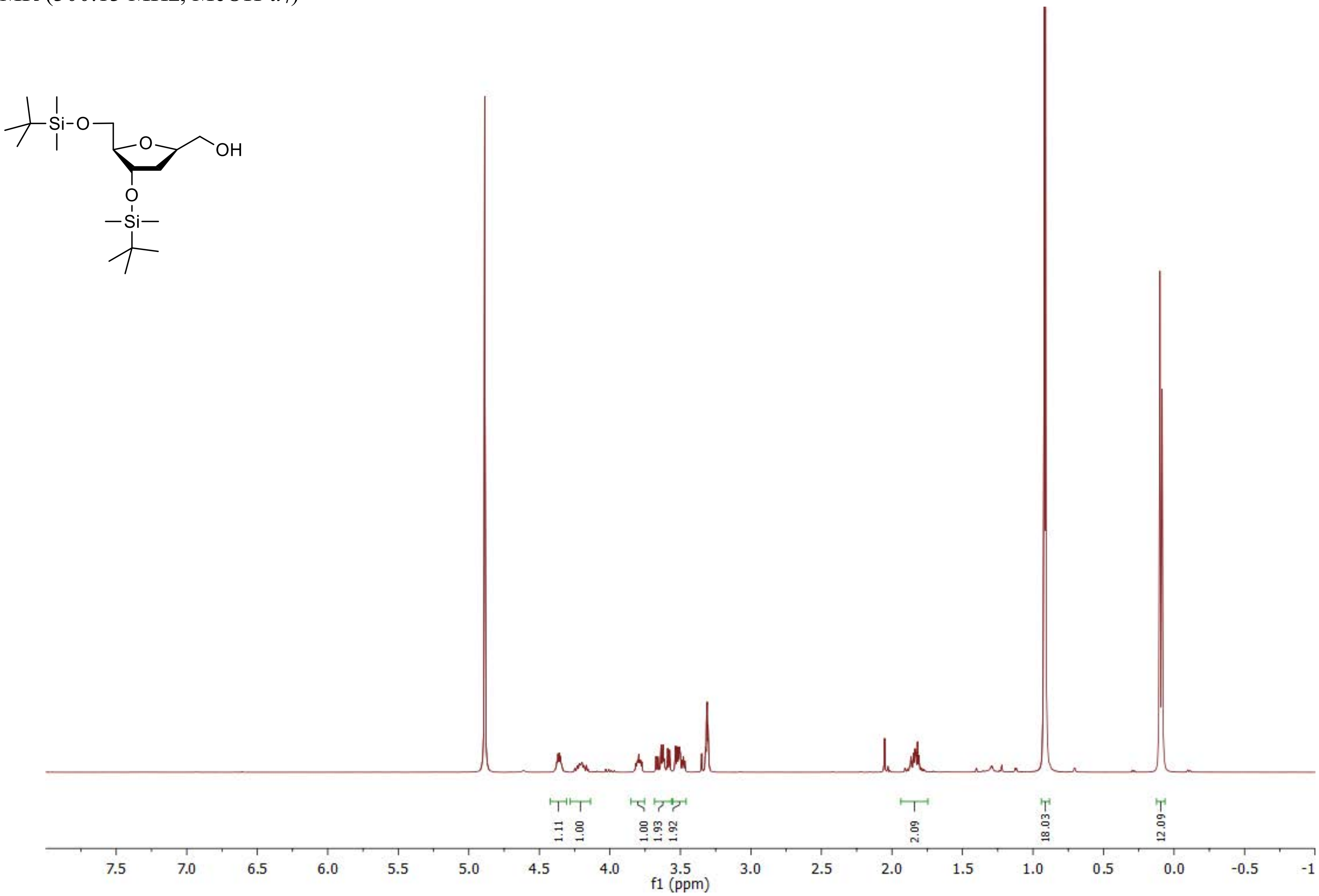

3,5-bis-*O*-(*tert*-Butyldimethylsilyl)-1,2-dideoxy-1β-(hydroxymethyl)-D-*erythro*-pentofuranose (11β)

^{13}C NMR (75.5 MHz, MeOH-d_4)

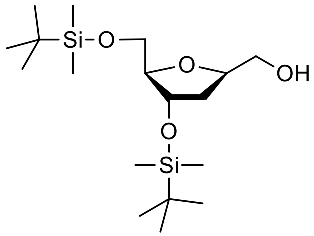

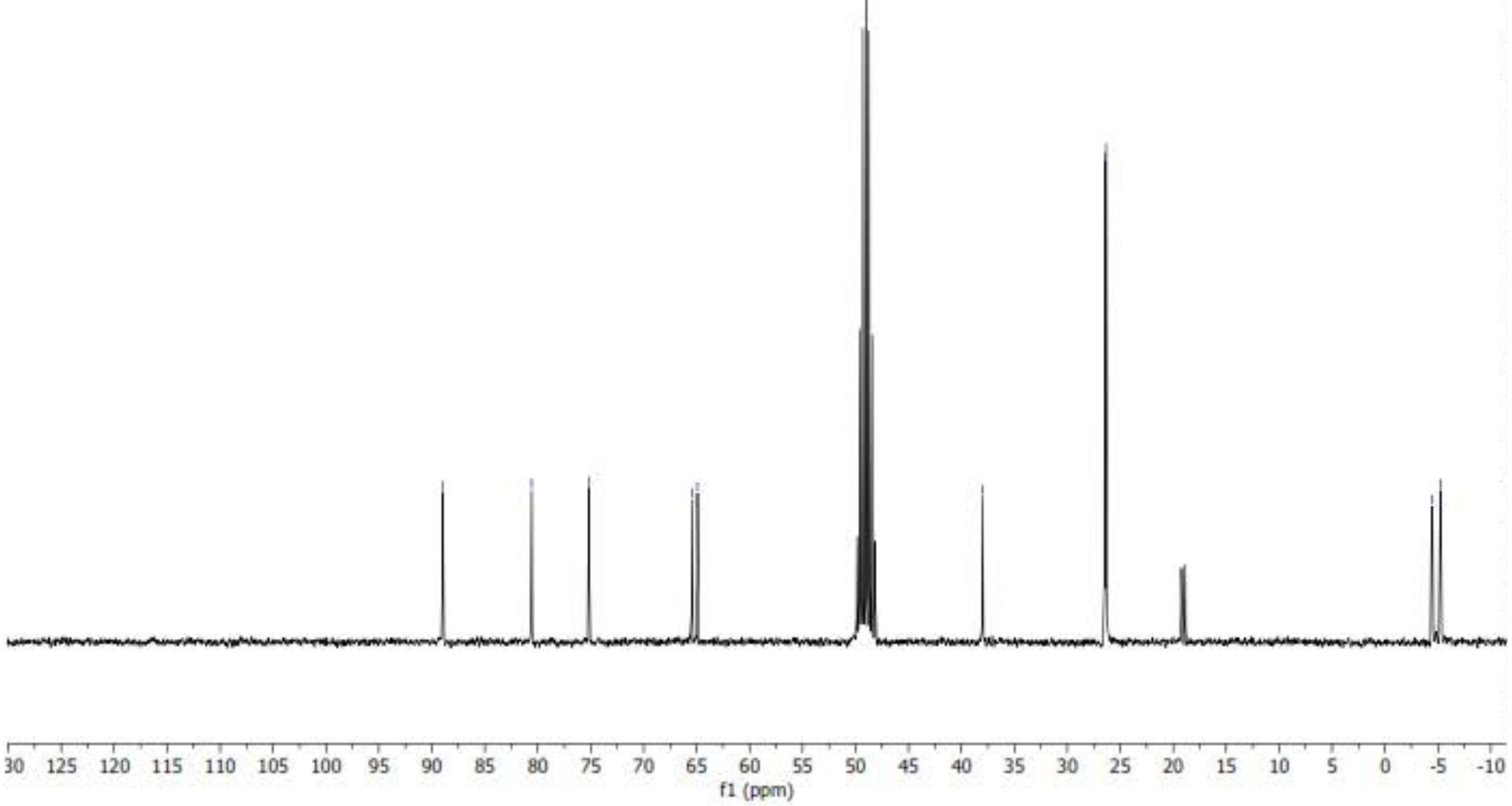

3,5-bis-*O*-(*tert*-Butyldimethylsilyl)-1,2-dideoxy-1β-(tosyloxy)methyl-ᴅ-*erythro*-pentofuranose (12β)

^{1}H NMR (300.13 MHz, MeOH-d_4)

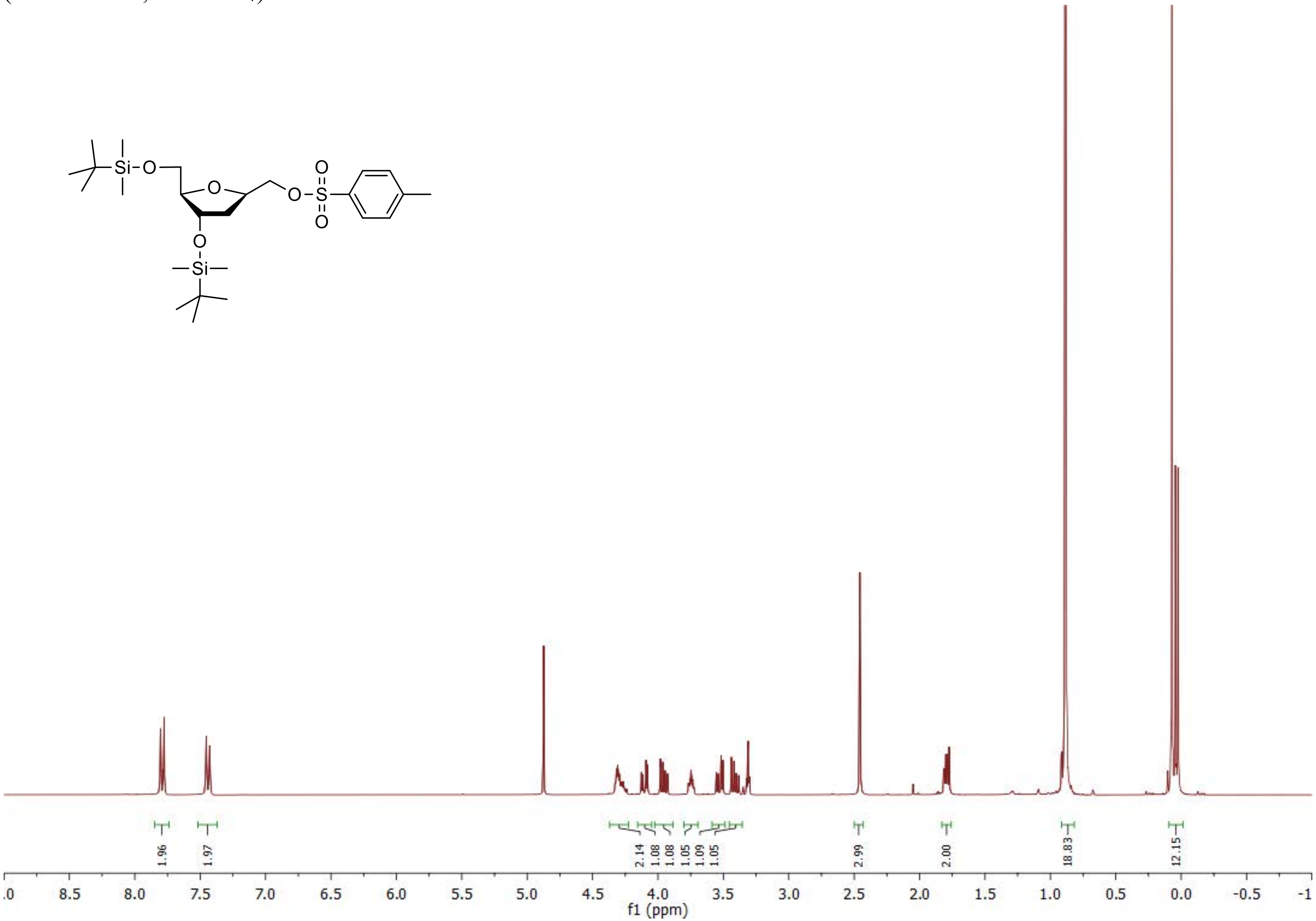

3,5-bis-*O*-(*tert*-Butyldimethylsilyl)-1,2-dideoxy-1β-(tosyloxy)methyl-D-*erythro*-pentofuranose (12β)

^{13}C NMR (75.5 MHz, MeOH-d_4)

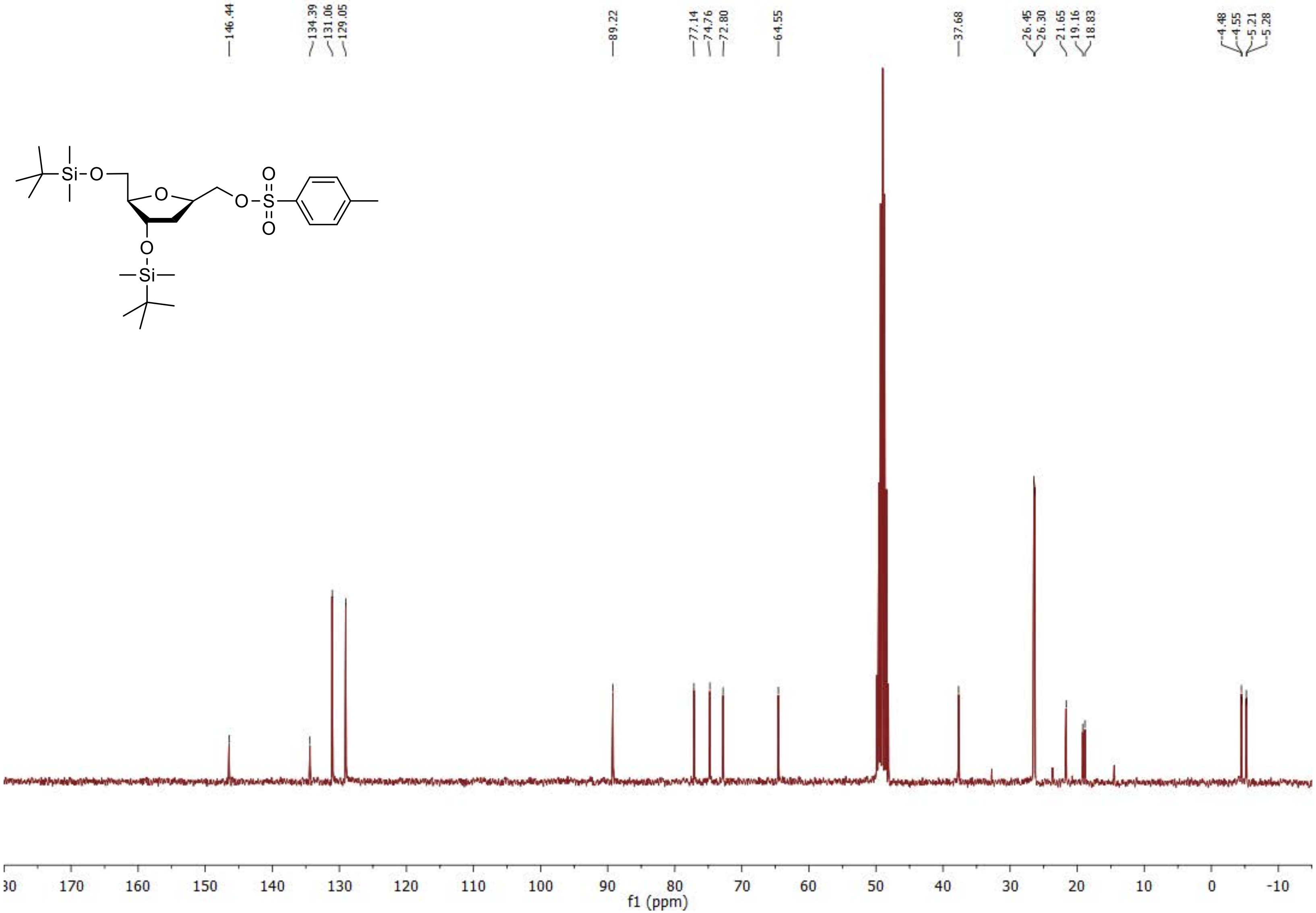

1α-(Acetylmercaptomethyl)-3,5-bis-*O*-(*tert*-butyldimethylsilyl)-1,2-dideoxy-D-*erythro*-pentofuranose (13α)

^{1}H NMR (300.13 MHz, $CDCl_3$)

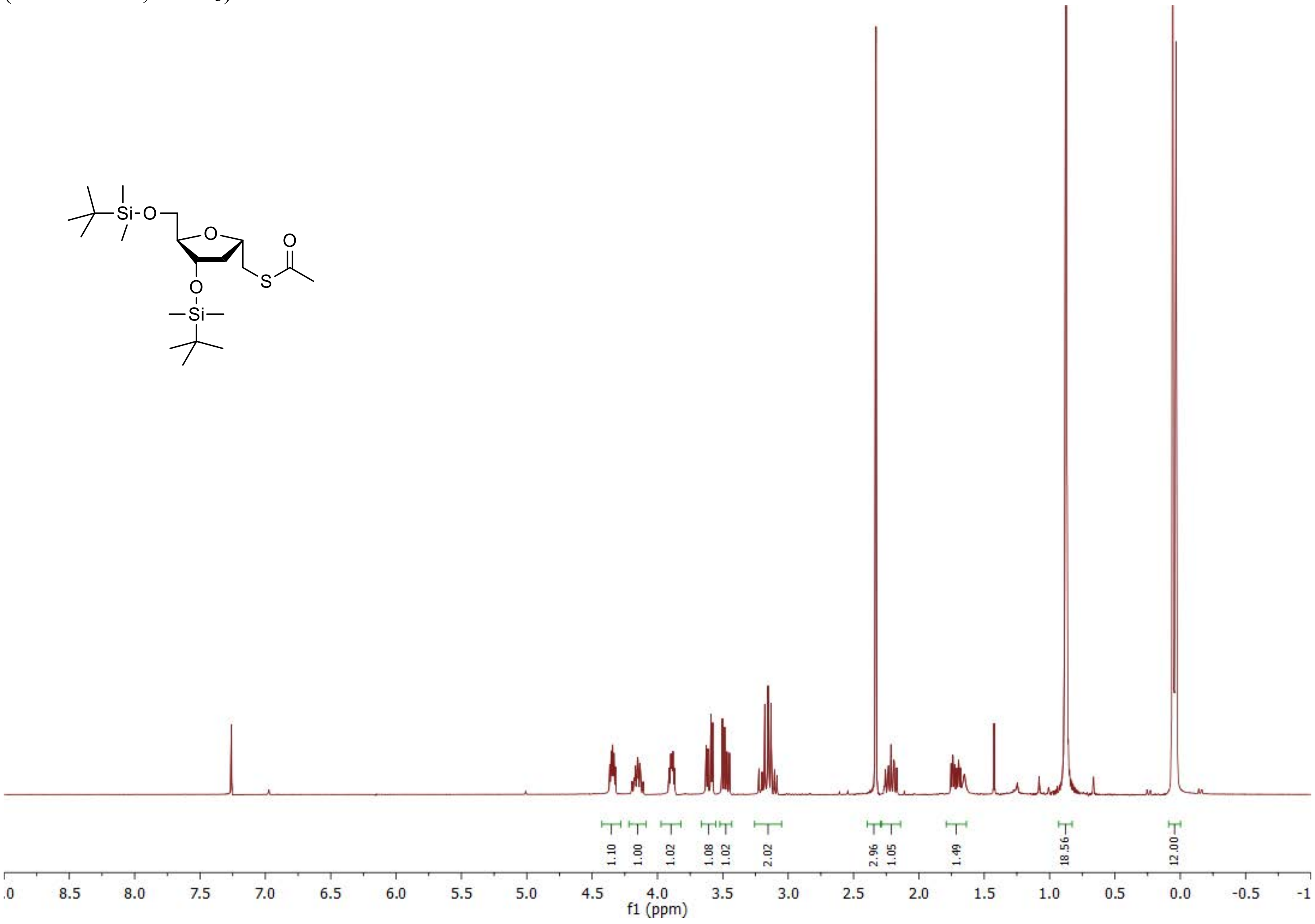

1α-(Acetylmercaptomethyl)-3,5-bis-*O*-(*tert*-butyldimethylsilyl)-1,2-dideoxy-D-*erythro*-pentofuranose (13α)

^{13}C NMR (75.5 MHz, $CDCl_3$)

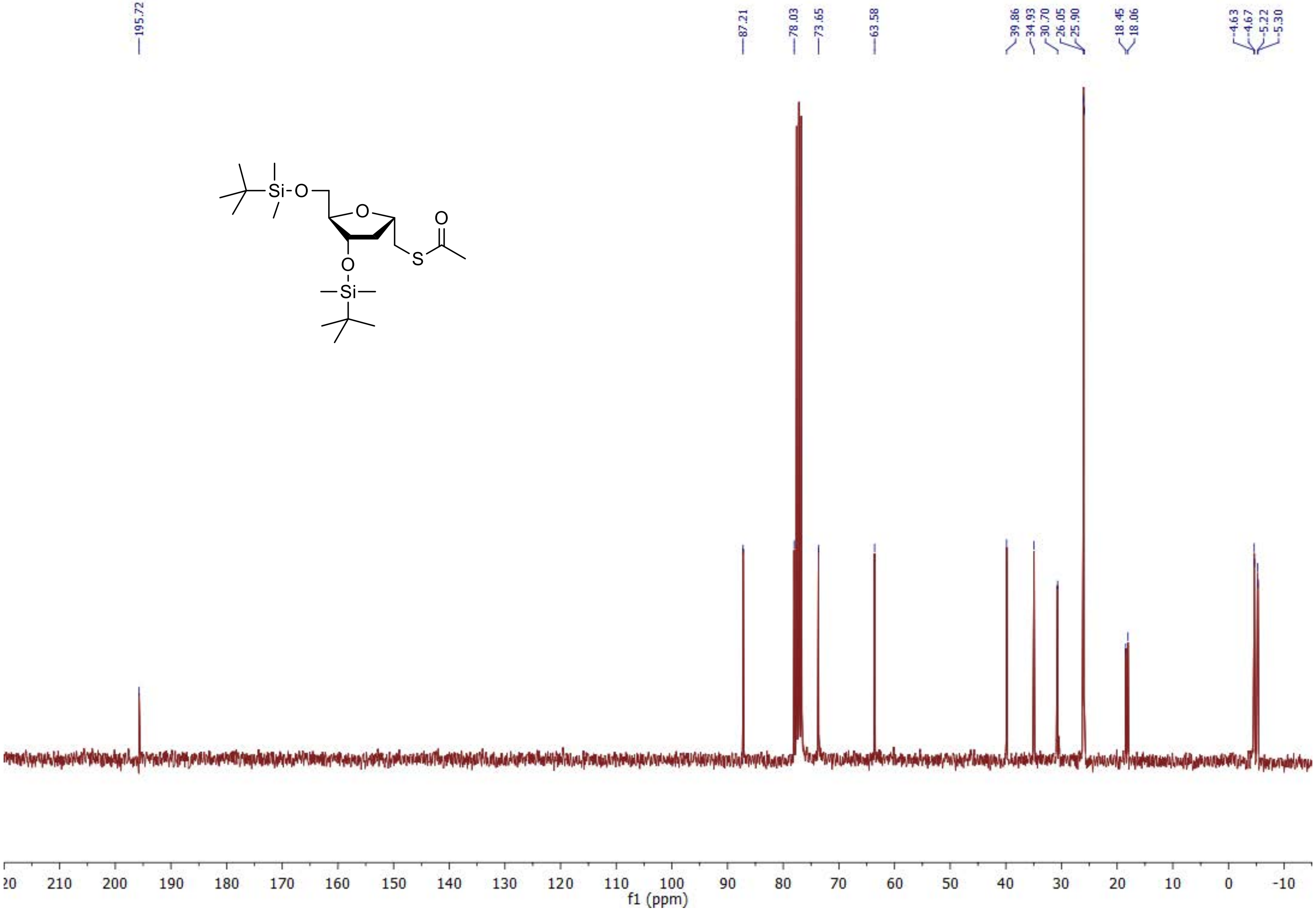

1α-(Acetylmercaptomethyl)-3,5-bis-*O*-(*tert*-butyldimethylsilyl)-1,2-dideoxy-D-*erythro*-pentofuranose (13α)

DEPT 135 NMR (75.5 MHz, $CDCl_3$)

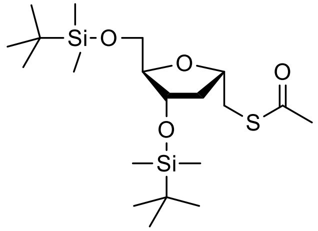

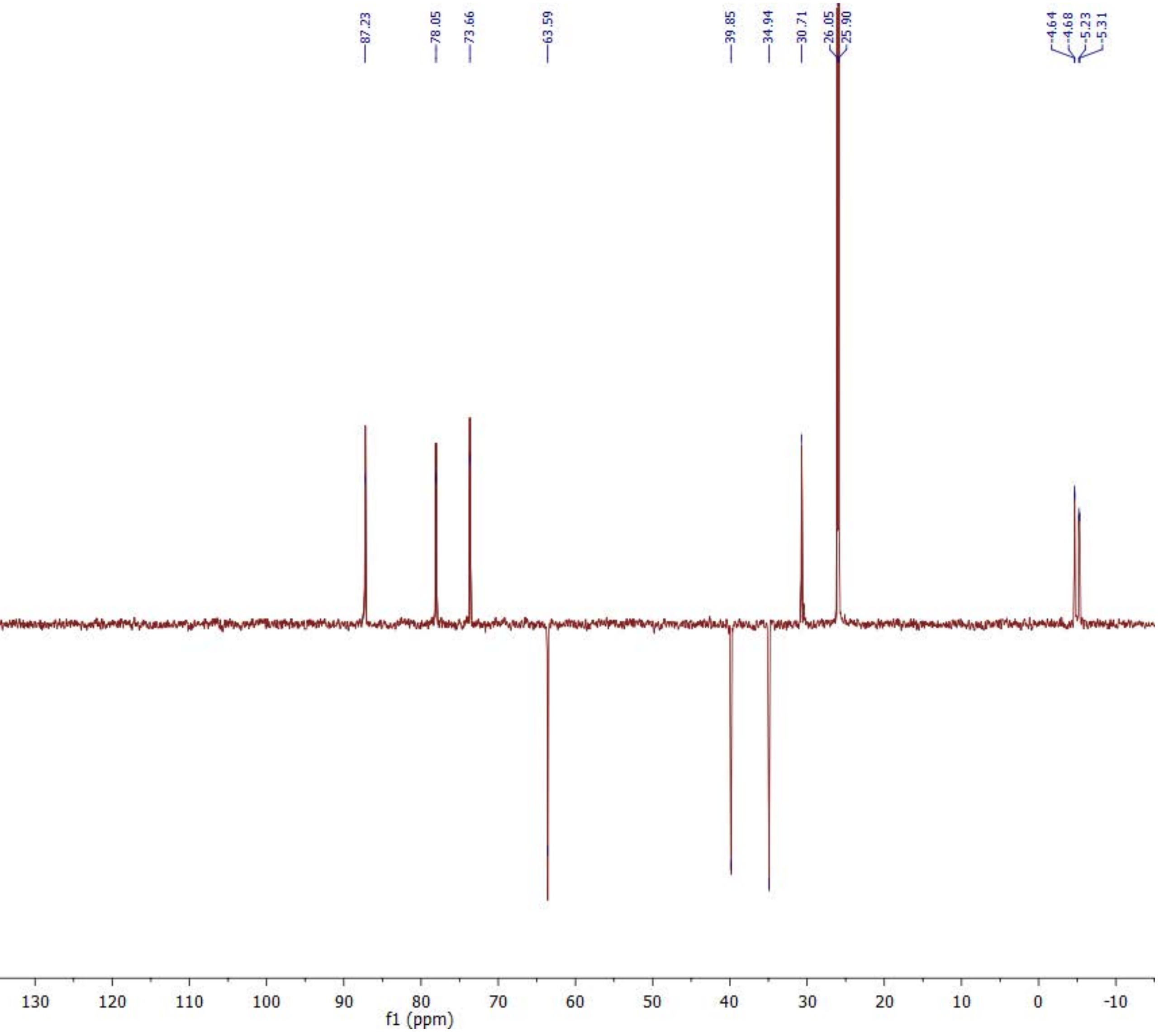

1α-(Acetylmercaptomethyl)-3,5-bis-*O*-(*tert*-butyldimethylsilyl)-1,2-dideoxy-D-*erythro*-pentofuranose (13α)

COSY NMR ($CDCl_3$)

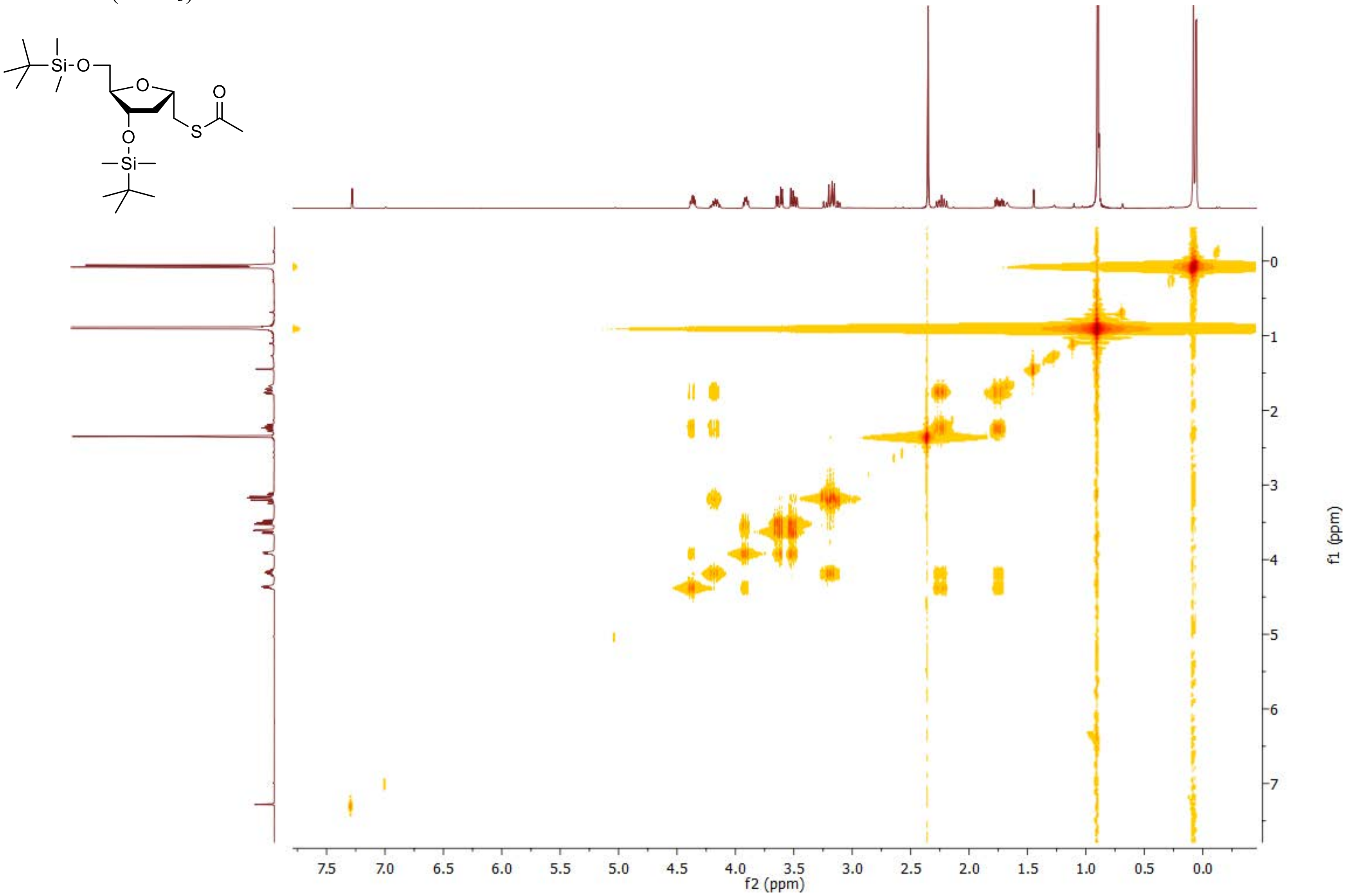

1α-(Acetylmercaptomethyl)-3,5-bis-*O*-(*tert*-butyldimethylsilyl)-1,2-dideoxy-D-*erythro*-pentofuranose (13α)

HSQC NMR ($CDCl_3$)

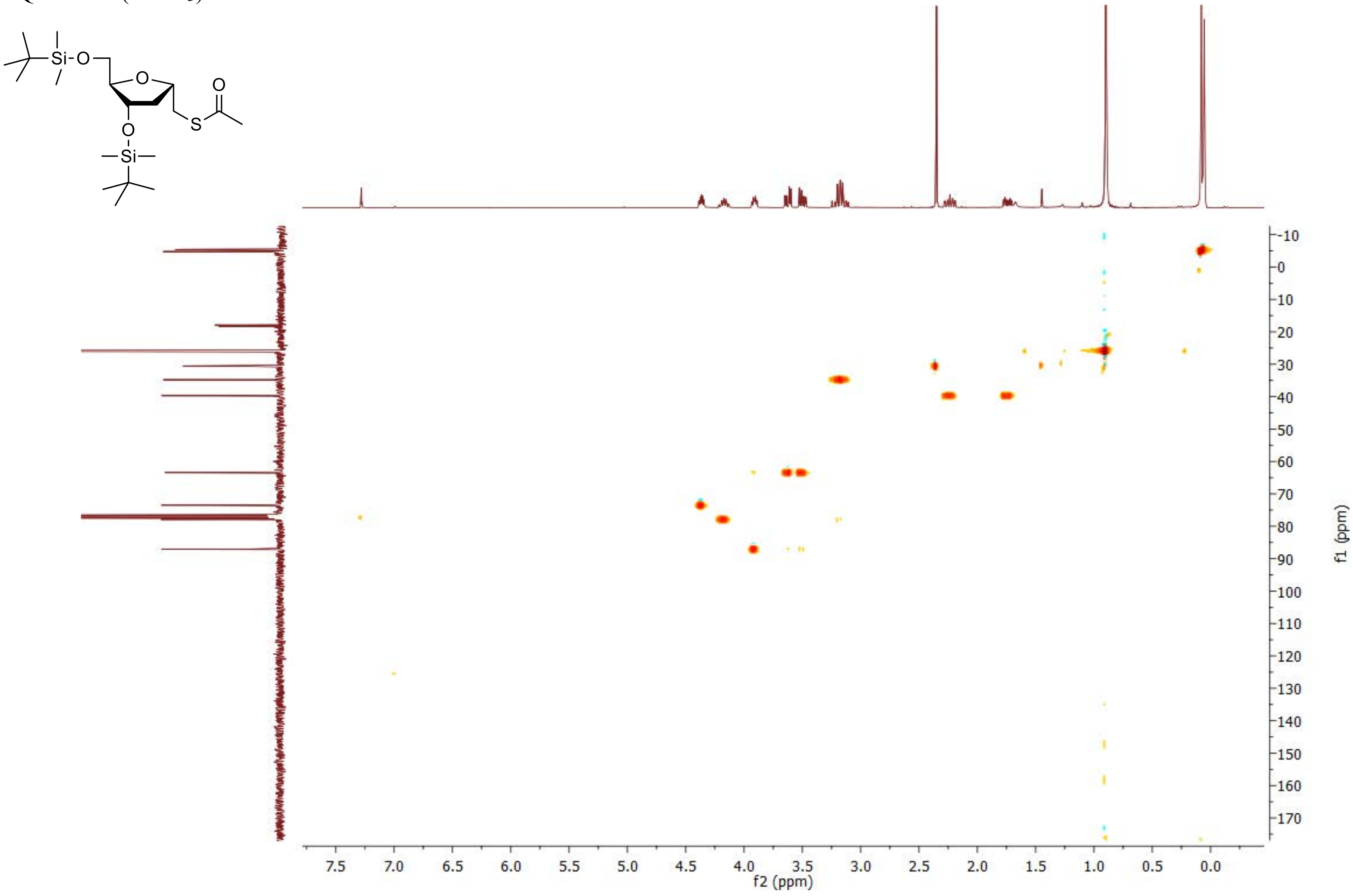

1α-(Acetylmercaptomethyl)-3,5-bis-*O*-(*tert*-butyldimethylsilyl)-1,2-dideoxy-D-*erythro*-pentofuranose (13α)

HMBC NMR ($CDCl_3$)

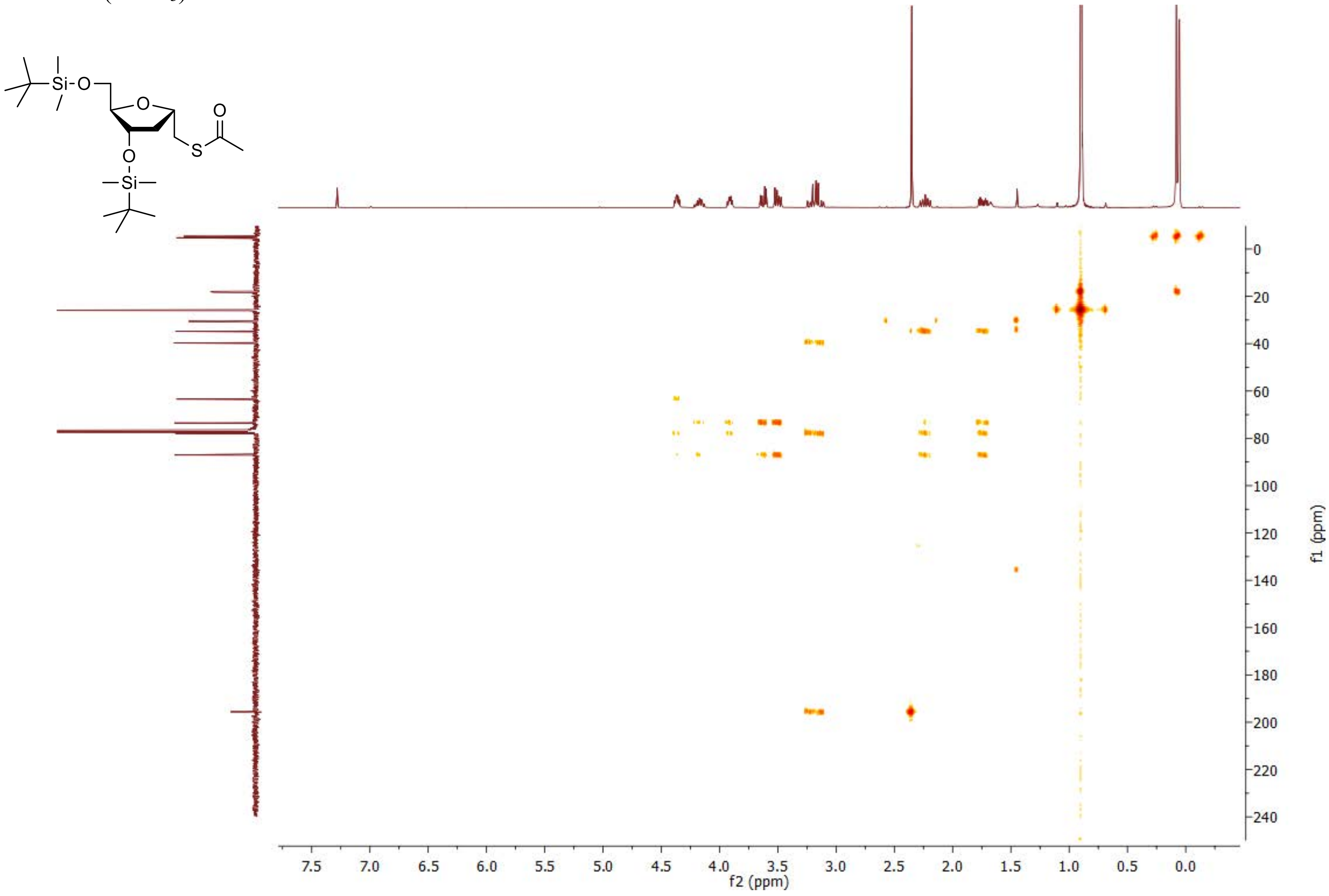

1β-(Acetylmercaptomethyl)-3,5-bis-*O*-(*tert*-butyldimethylsilyl)-1,2-dideoxy-D-*erythro*-pentofuranose (13β)

^{1}H NMR (300.13 MHz, $CDCl_3$)

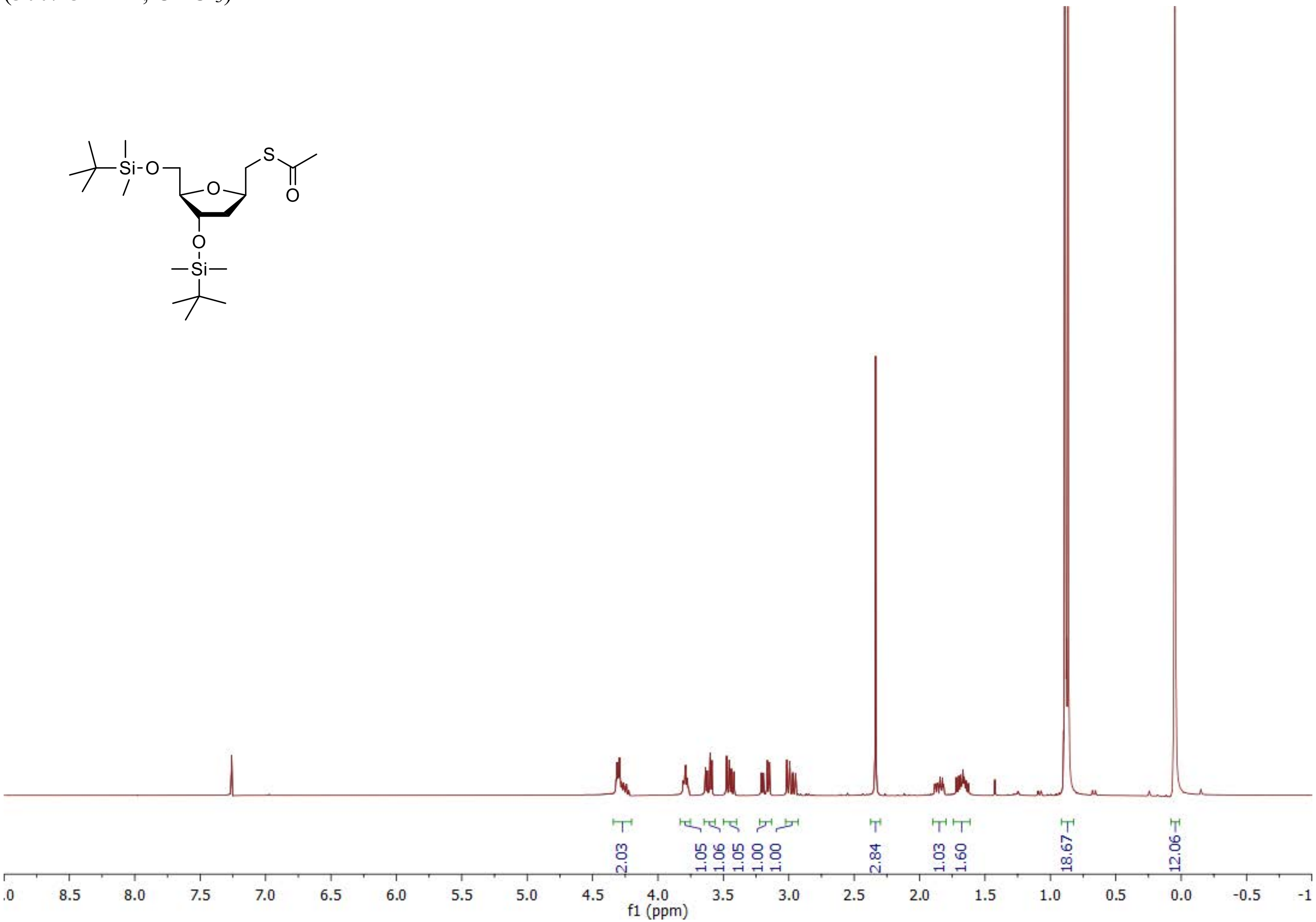

1β-(Acetylmercaptomethyl)-3,5-bis-*O*-(*tert*-butyldimethylsilyl)-1,2-dideoxy-D-*erythro*-pentofuranose (13β)

^{13}C NMR (75.5 MHz, $CDCl_3$)

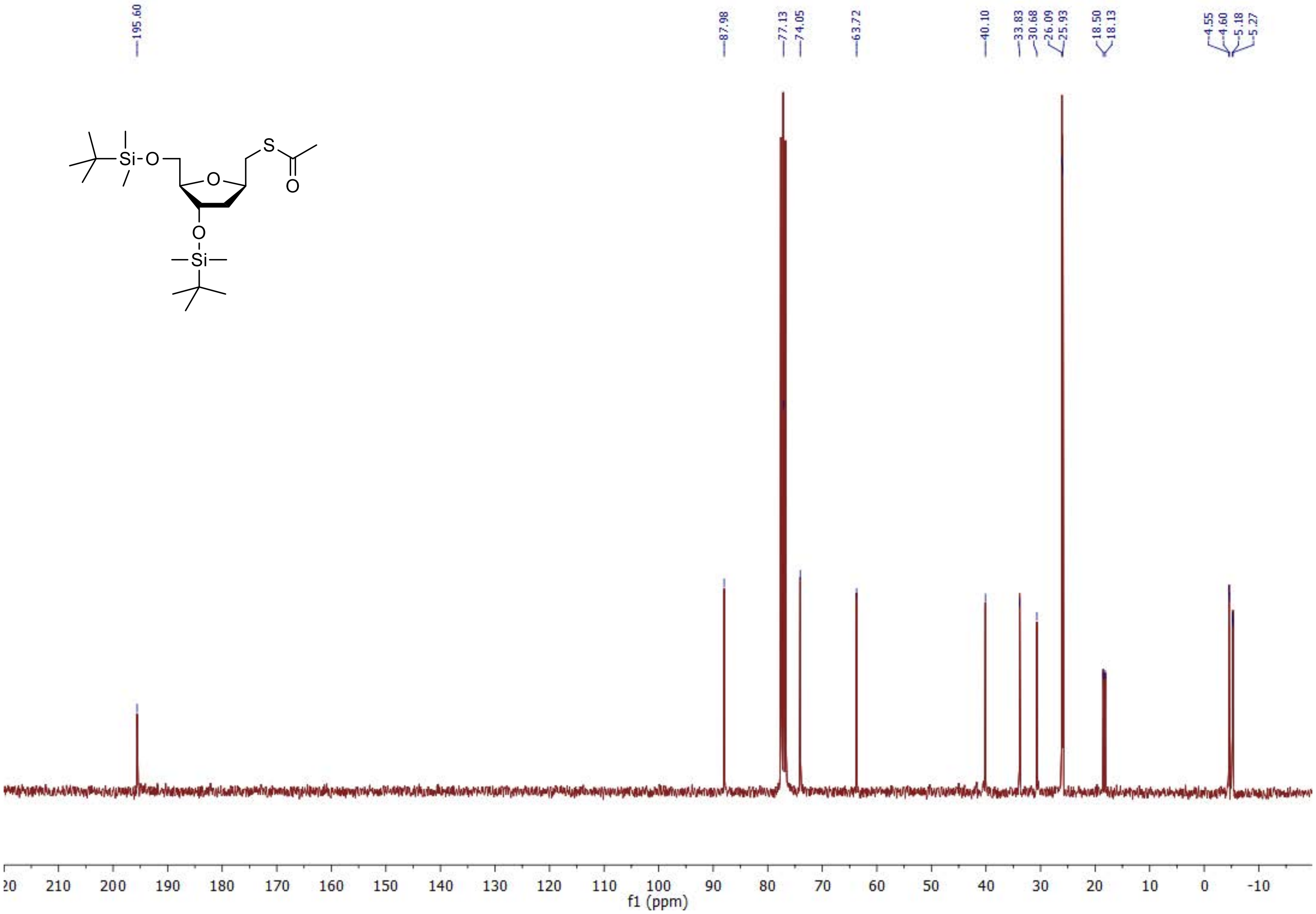

1β-(Acetylmercaptomethyl)-3,5-bis-*O*-(*tert*-butyldimethylsilyl)-1,2-dideoxy-D-*erythro*-pentofuranose (13β)

DEPT 135 NMR (75.5 MHz, $CDCl_3$)

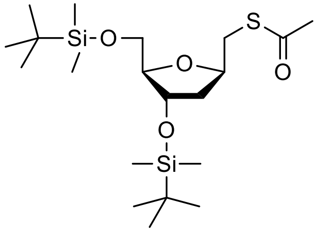

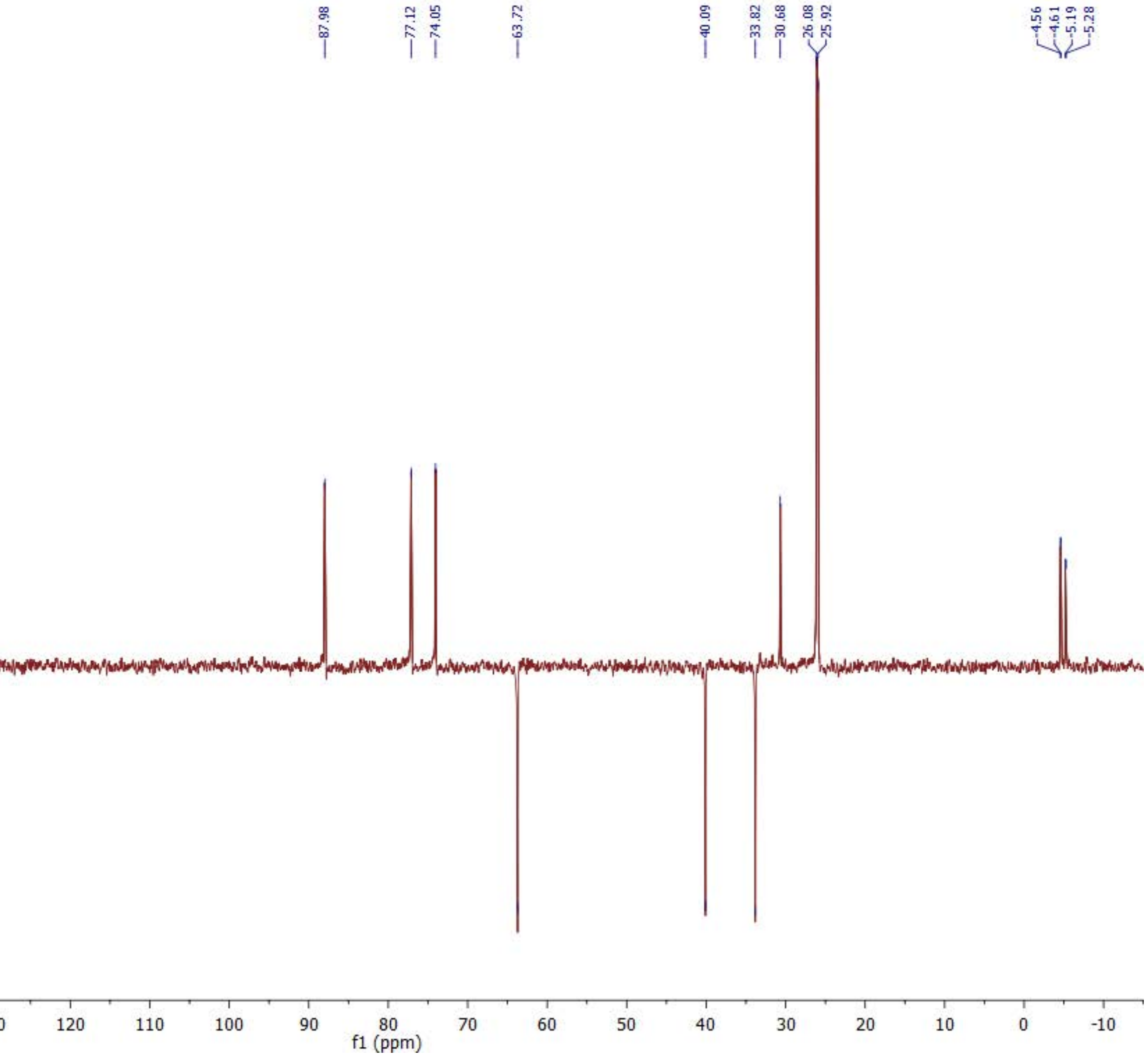

1β-(Acetylmercaptomethyl)-3,5-bis-*O*-(*tert*-butyldimethylsilyl)-1,2-dideoxy-D-*erythro*-pentofuranose (13β)

COSY NMR ($CDCl_3$)

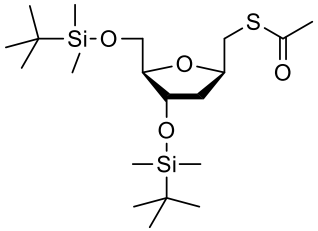

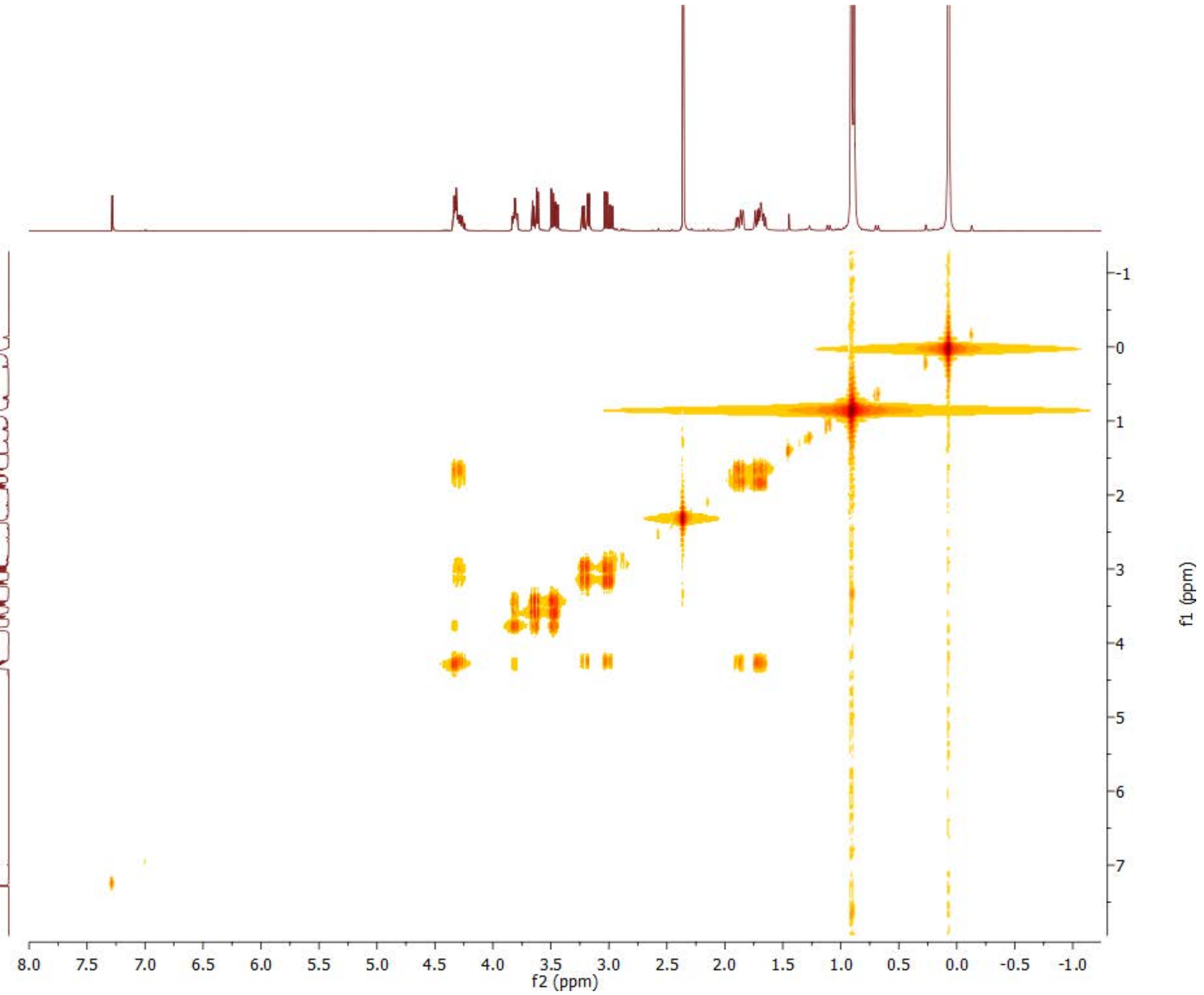

1β-(Acetylmercaptomethyl)-3,5-bis-*O*-(*tert*-butyldimethylsilyl)-1,2-dideoxy-ᴅ-*erythro*-pentofuranose (13β)

HSQC NMR ($CDCl_3$)

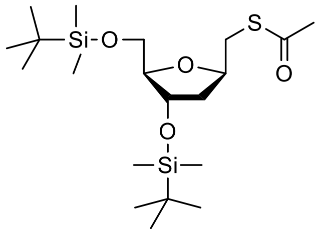

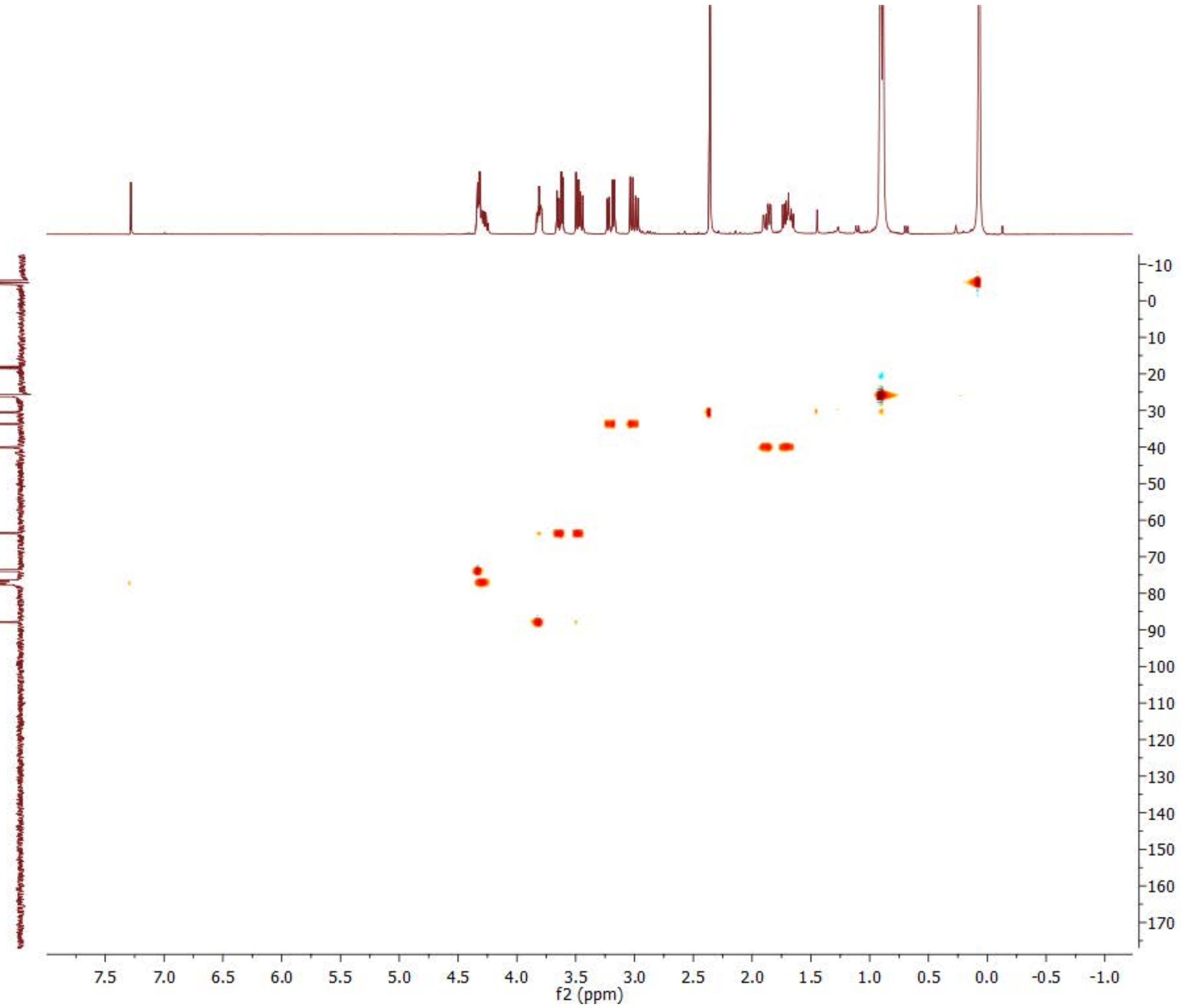

1β-(Acetylmercaptomethyl)-3,5-bis-*O*-(*tert*-butyldimethylsilyl)-1,2-dideoxy-D-*erythro*-pentofuranose (13β)

HMBC NMR ($CDCl_3$)

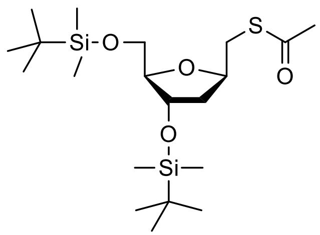

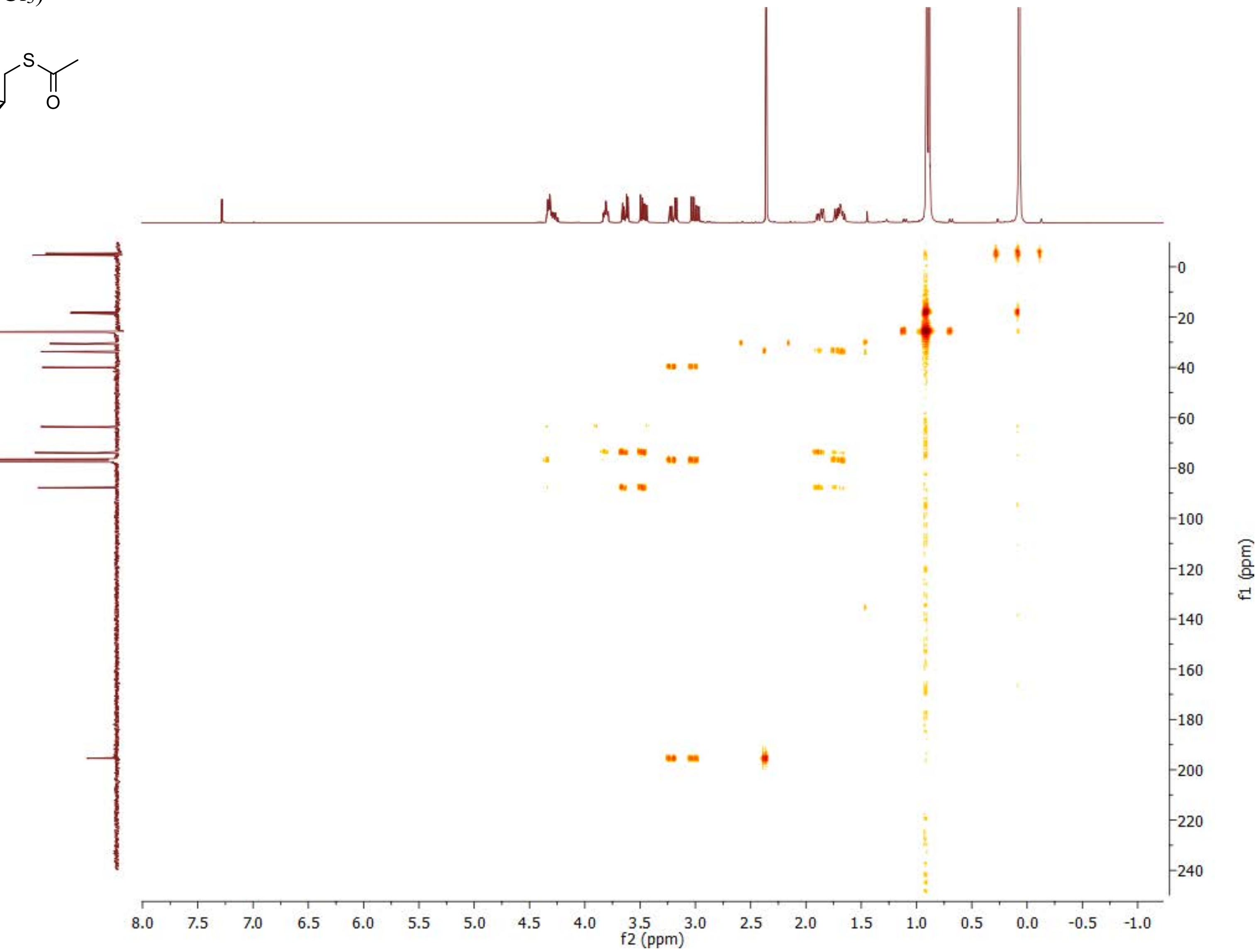

1α-(Acetylmercaptomethyl)-1,2-dideoxy-D-*erythro*-pentofuranose (14α)

^{1}H NMR (300.13 MHz, MeOH-d_4)

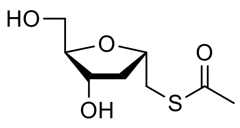

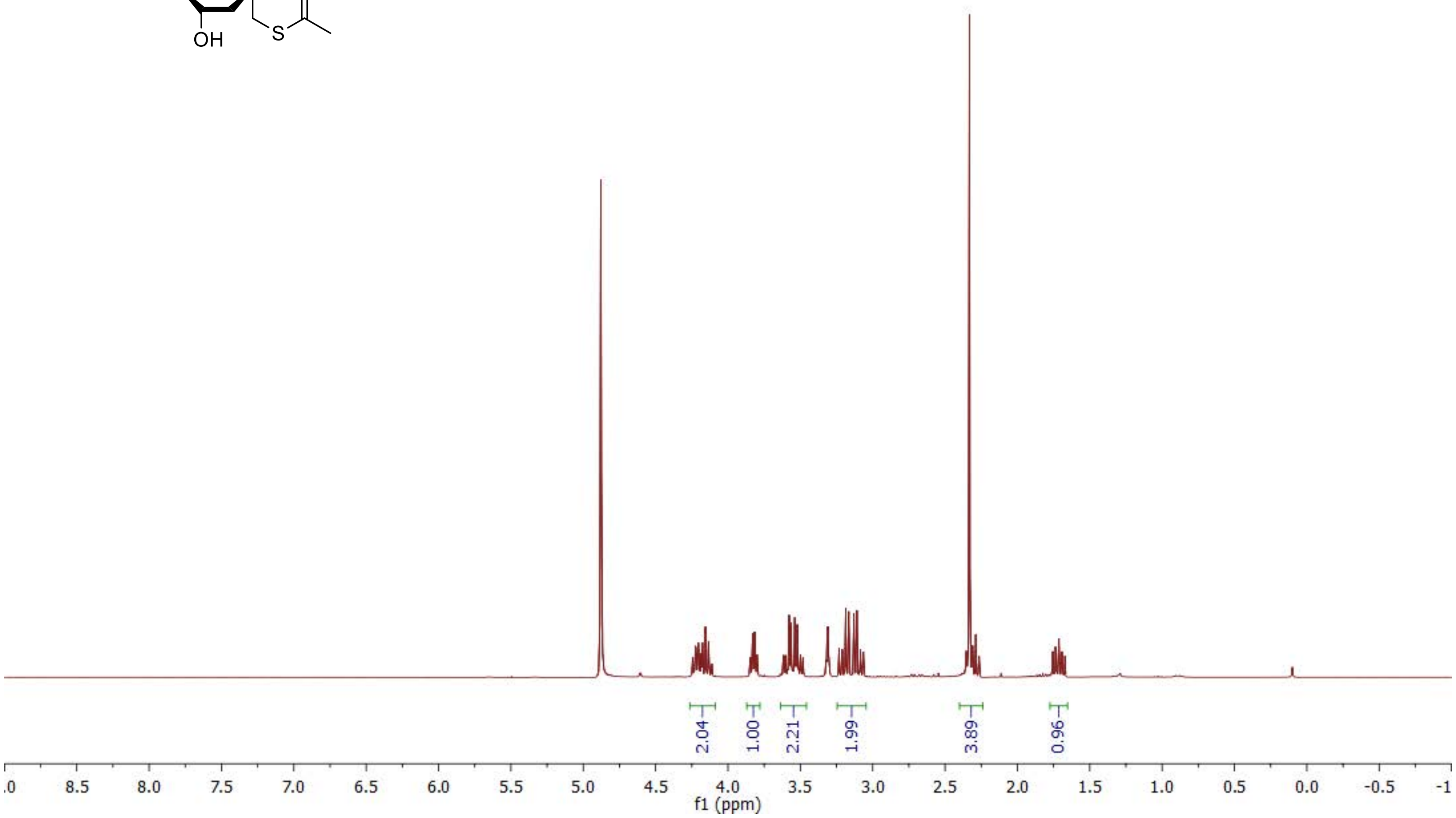

1α-(Acetylmercaptomethyl)-1,2-dideoxy-D-*erythro*-pentofuranose (14α)

^{13}C NMR (75.5 MHz, MeOH-d_4)

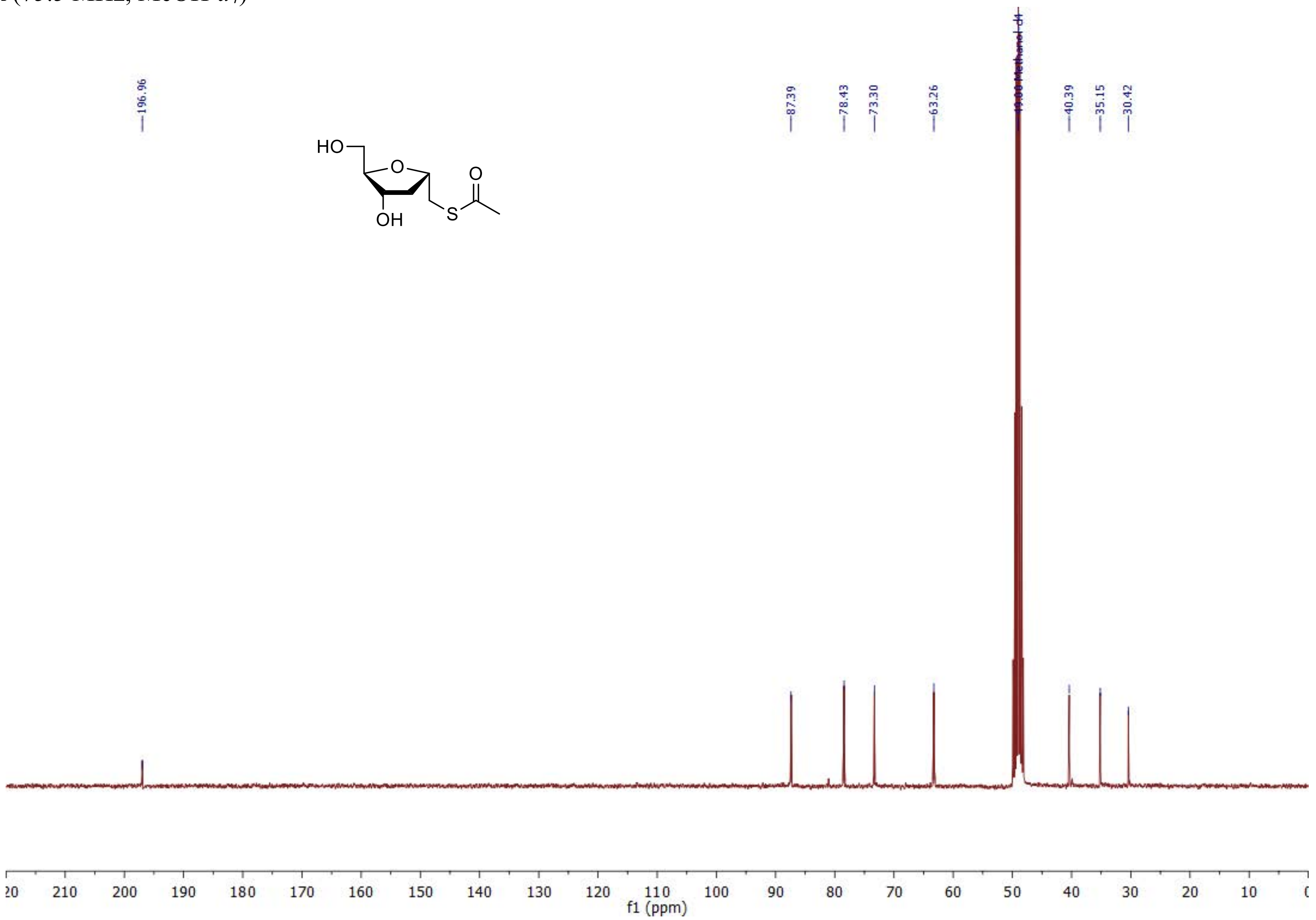

1α-(Acetylmercaptomethyl)-1,2-dideoxy-D-*erythro*-pentofuranose (14α)

DEPT 135 NMR (75.5 MHz, MeOH-d_4)

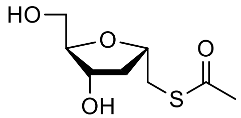

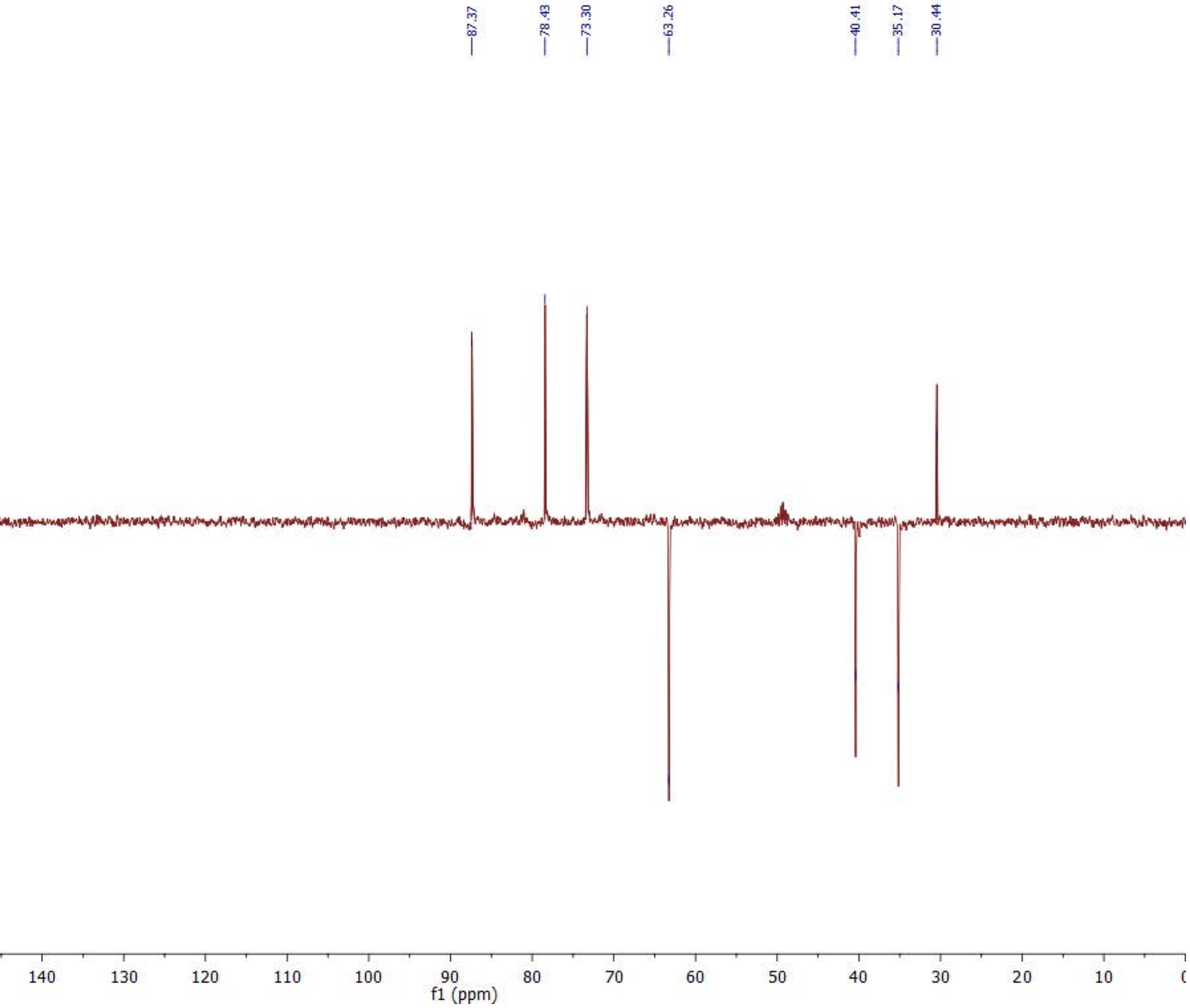

1α-(Acetylmercaptomethyl)-1,2-dideoxy-D-*erythro*-pentofuranose (14α)

COSY NMR (MeOH-d_4)

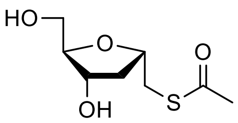

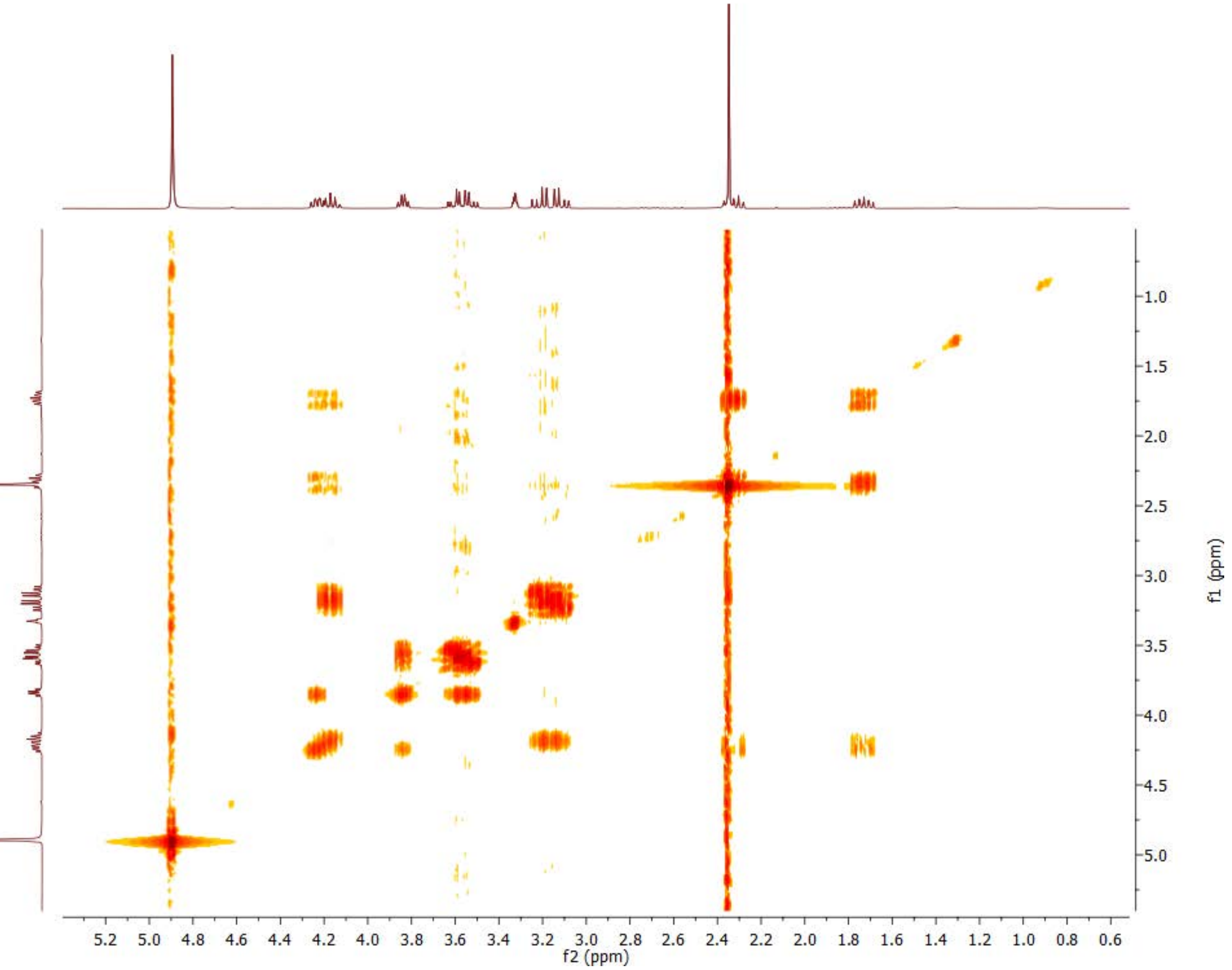

HSQC NMR (MeOH-d_4)

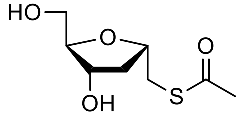

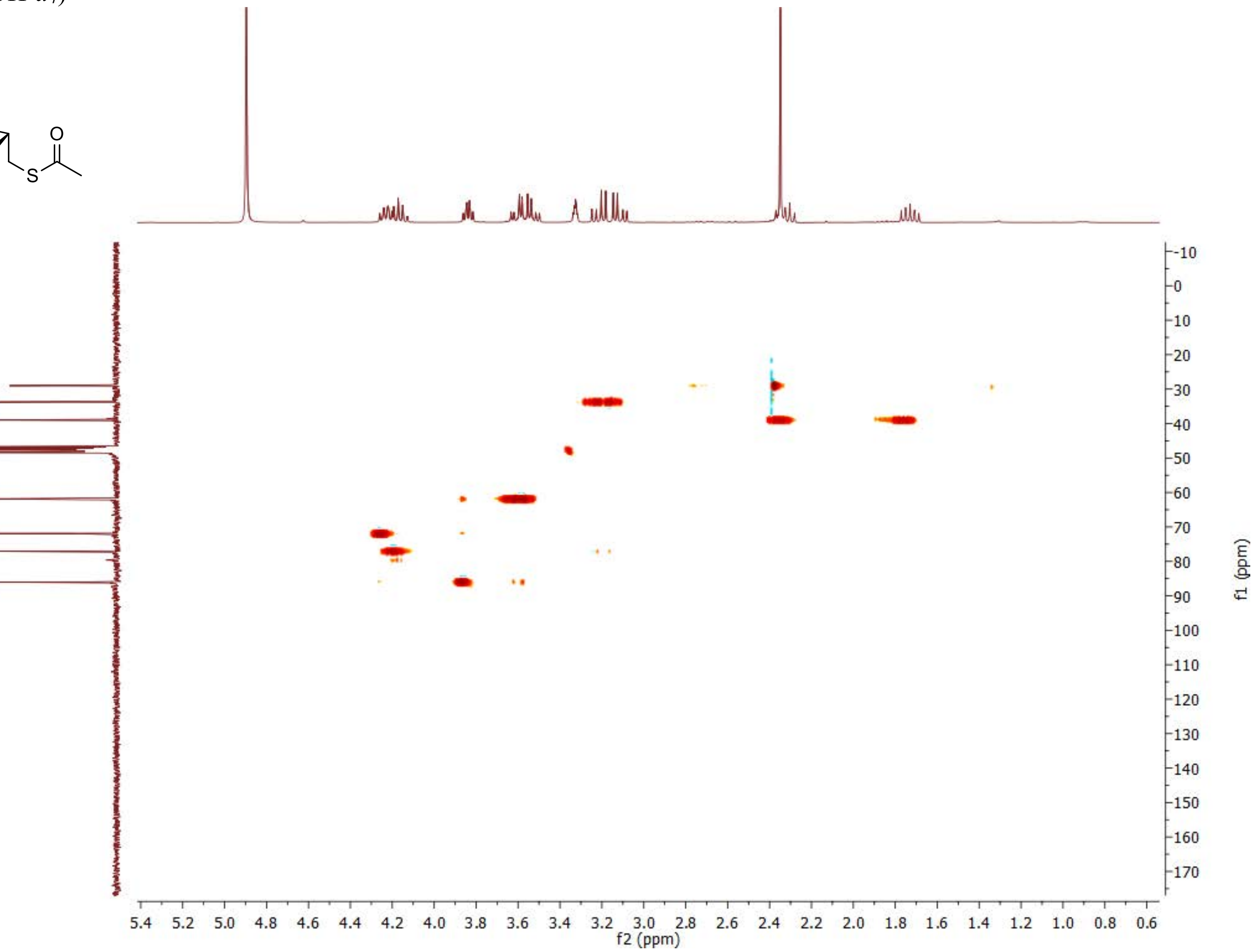

1α-(Acetylmercaptomethyl)-1,2-dideoxy-D-*erythro*-pentofuranose (14α)

HMBC NMR (MeOH-d_4)

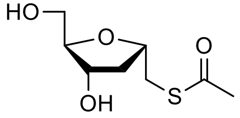

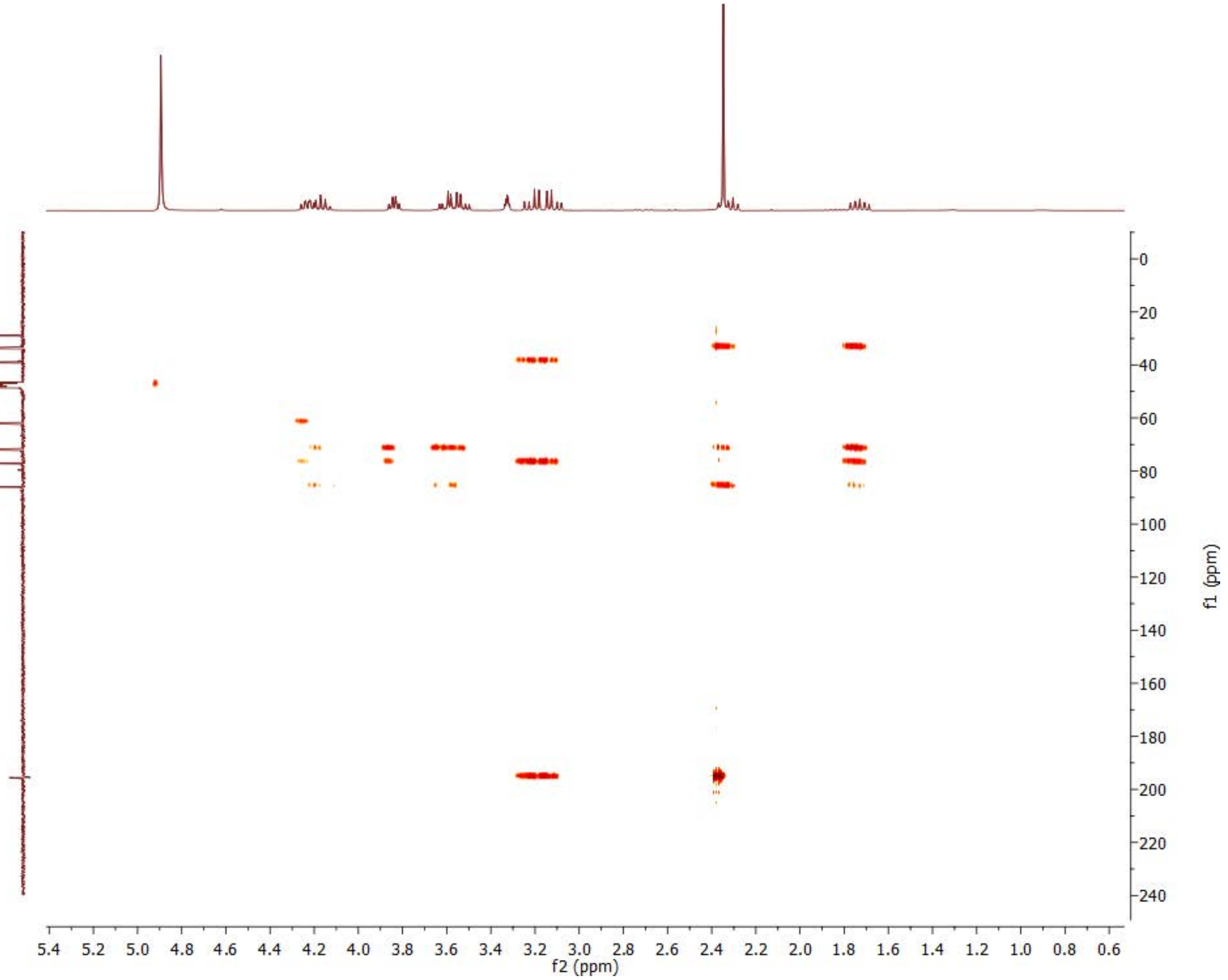

1β-(Acetylmercaptomethyl)-1,2-dideoxy-D-*erythro*-pentofuranose (14β)

^{1}H NMR (300.13 MHz, MeOH-d_4)

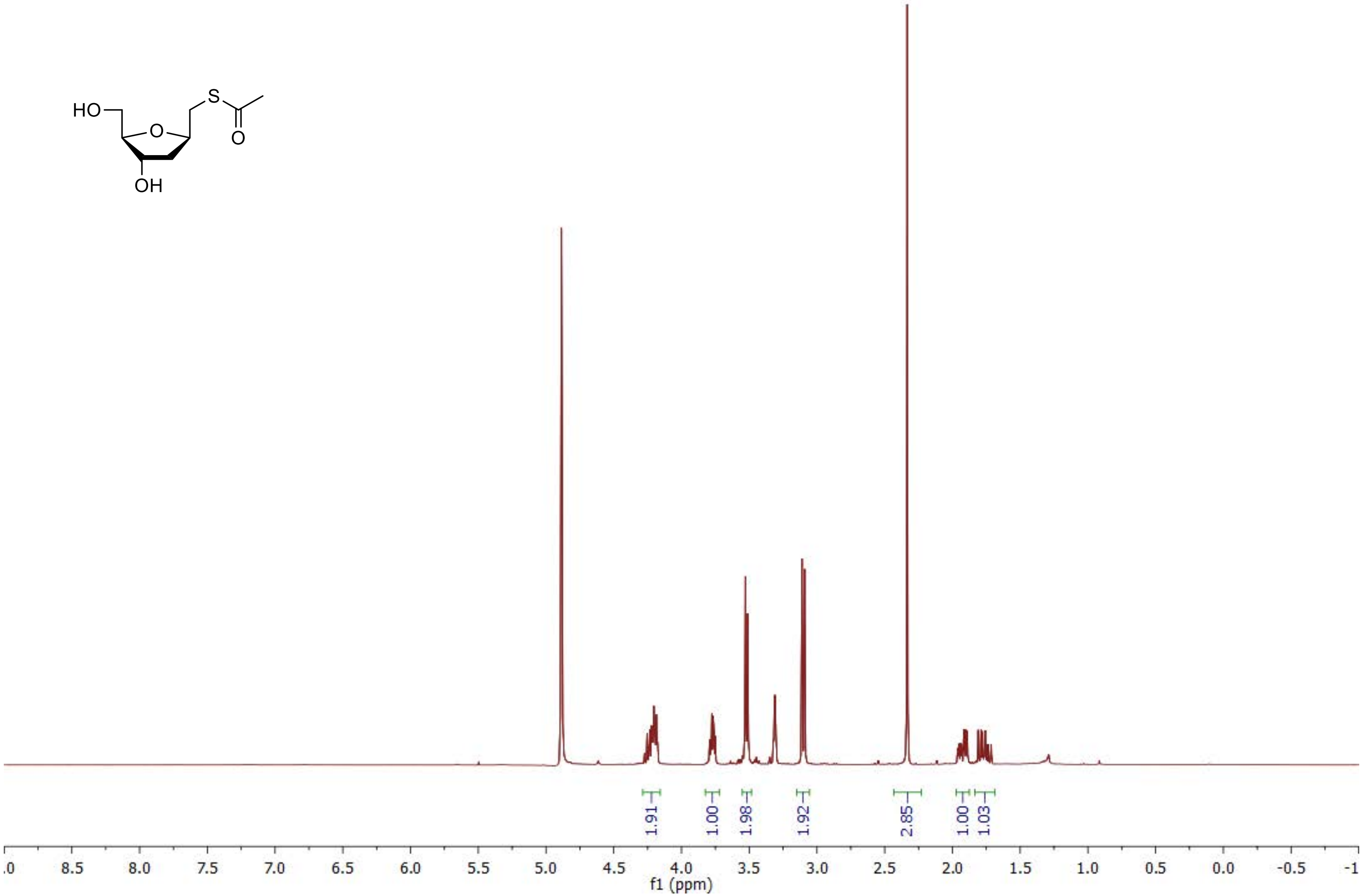

1β-(Acetylmercaptomethyl)-1,2-dideoxy-D-*erythro*-pentofuranose (14β)

^{13}C NMR (75.5 MHz, MeOH-d_4)

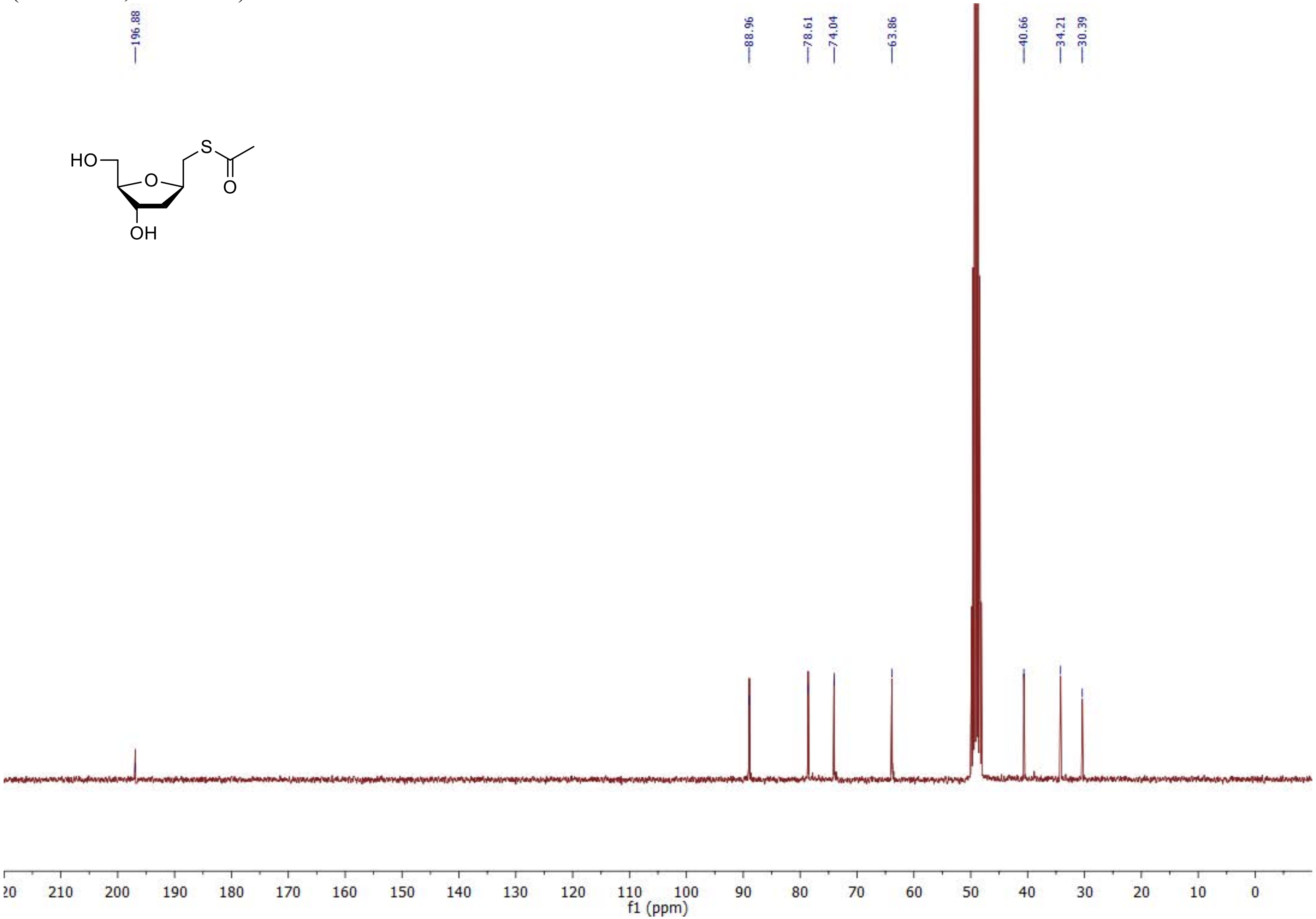

1β-(Acetylmercaptomethyl)-1,2-dideoxy-D-*erythro*-pentofuranose (14β)

DEPT 135 NMR (75.5 MHz, MeOH-d_4)

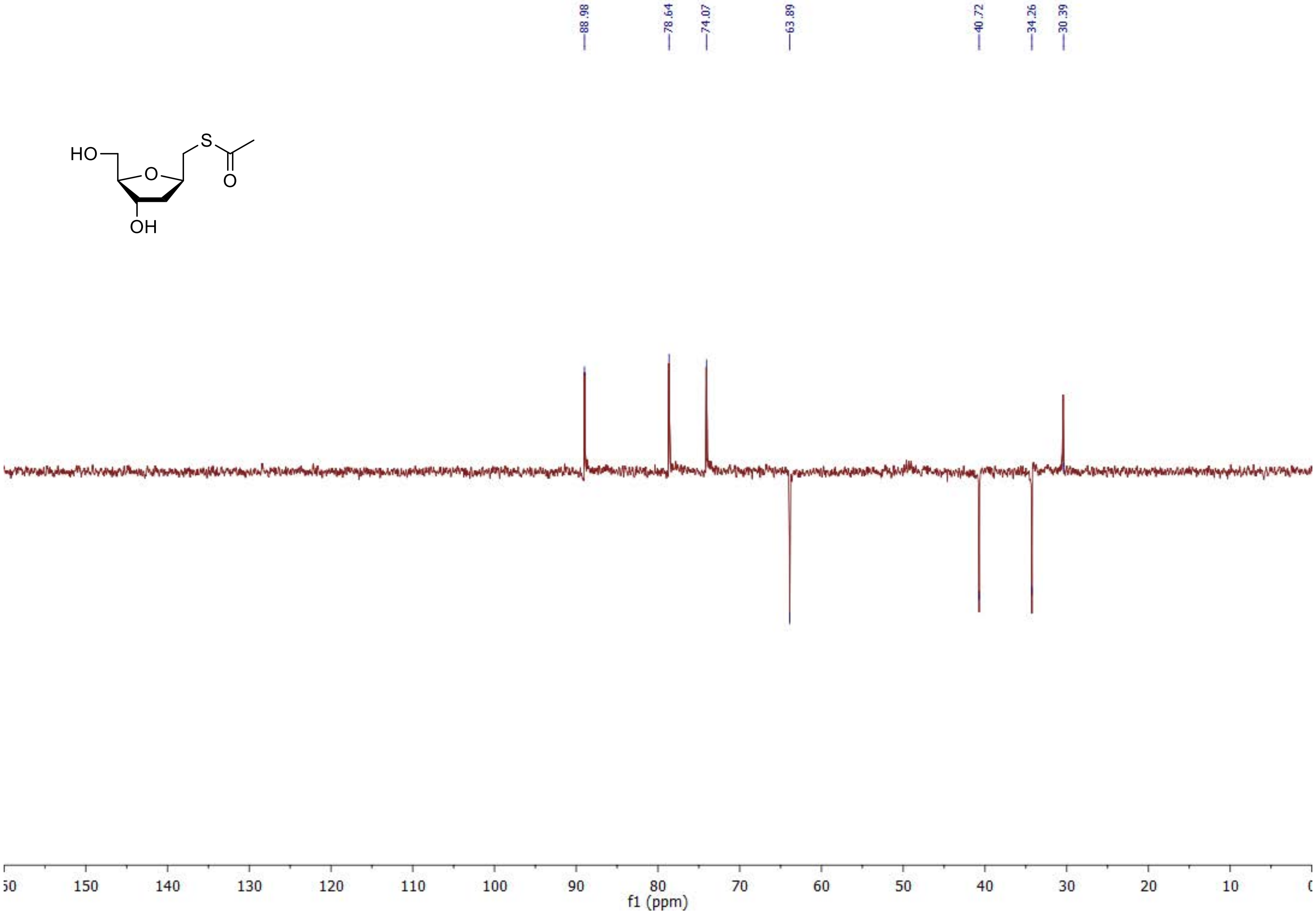

1β-(Acetylmercaptomethyl)-1,2-dideoxy-D-*erythro*-pentofuranose (14β)

COSY NMR (MeOH-d_4)

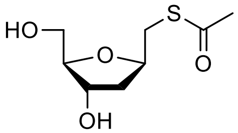

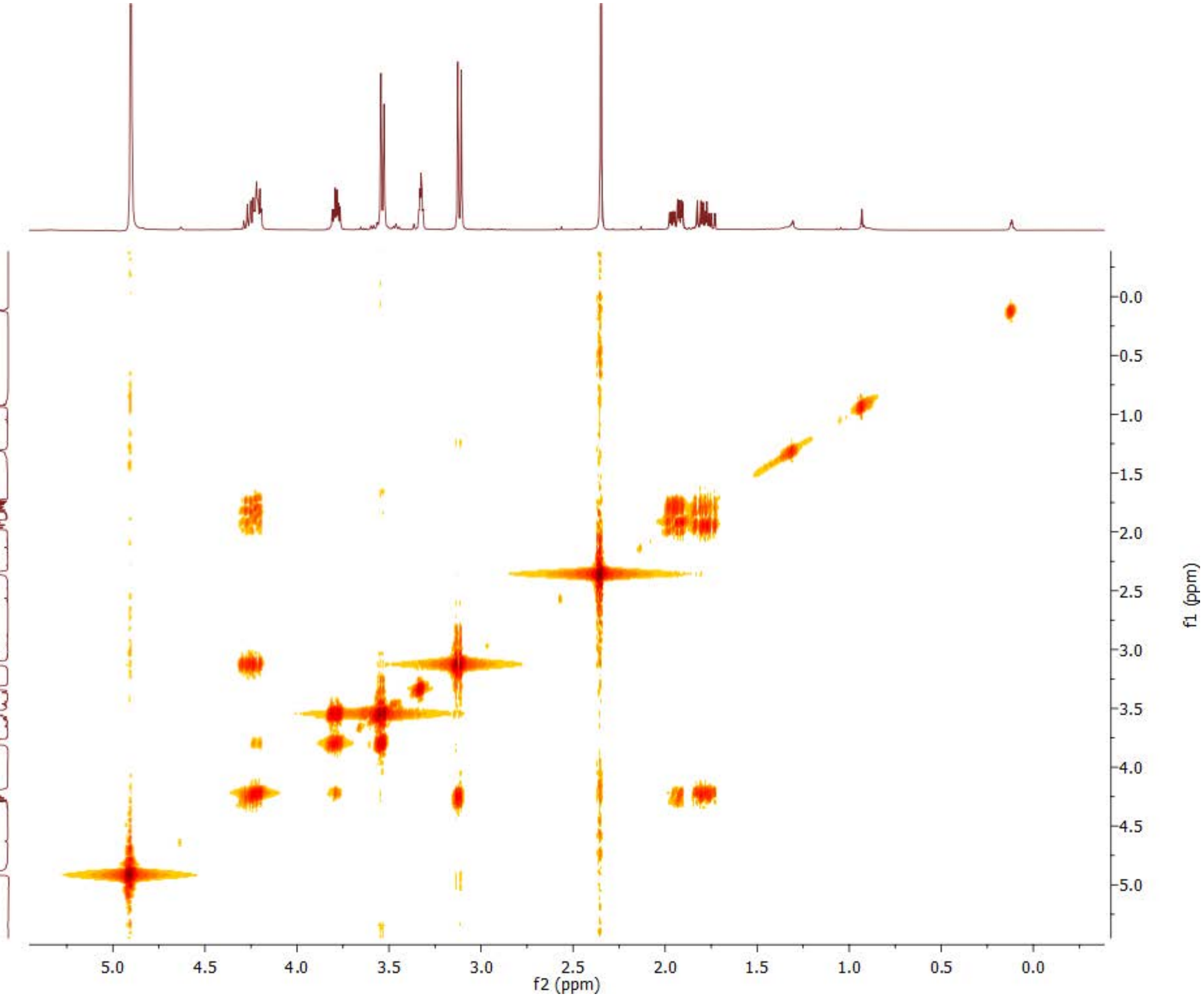

1β-(Acetylmercaptomethyl)-1,2-dideoxy-D-*erythro*-pentofuranose (14β)

HSQC NMR (MeOH-d_4)

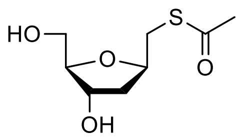

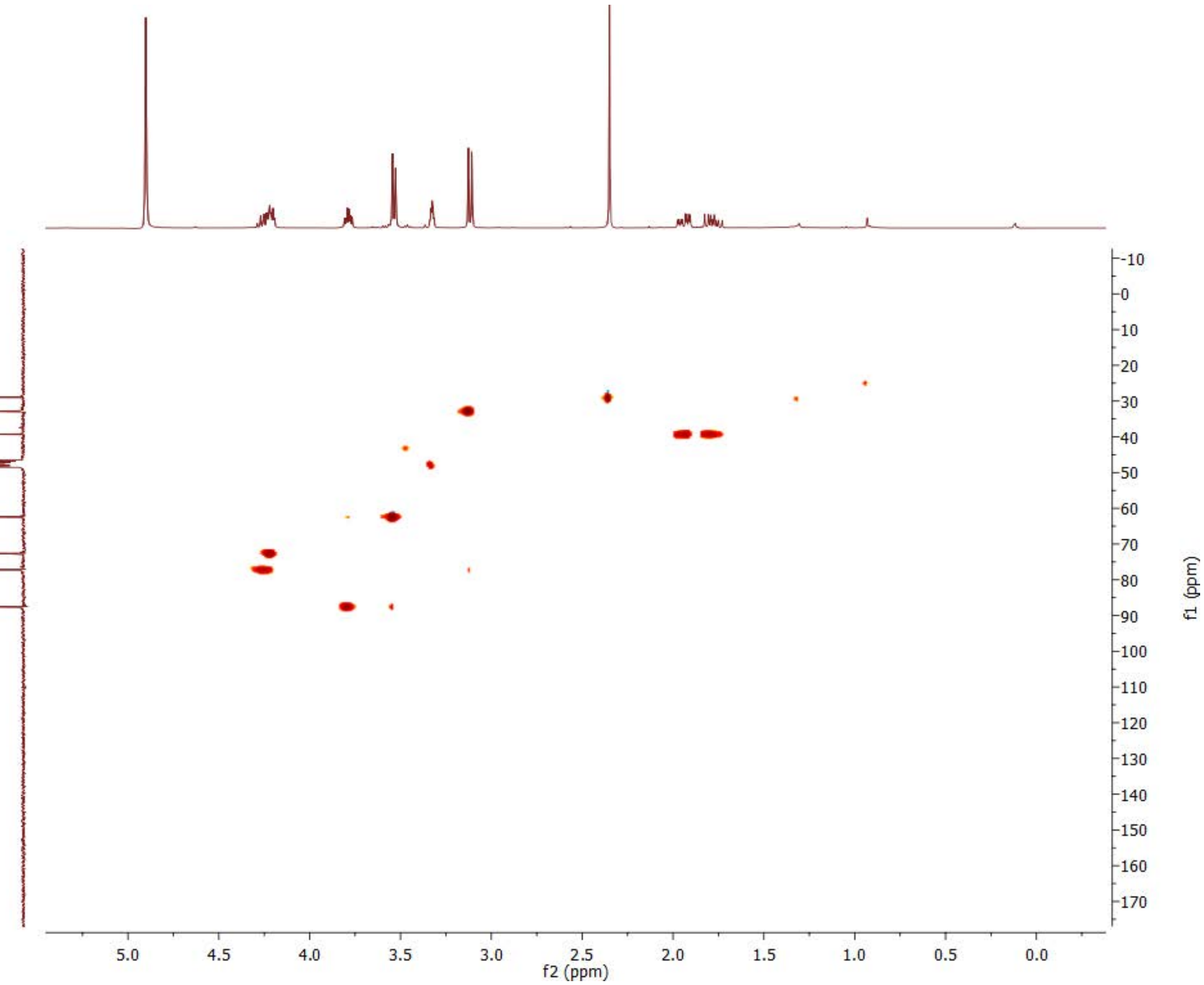

1β-(Acetylmercaptomethyl)-1,2-dideoxy-D-*erythro*-pentofuranose (14β)

HMBC NMR (MeOH-d_4)

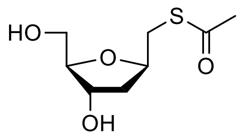

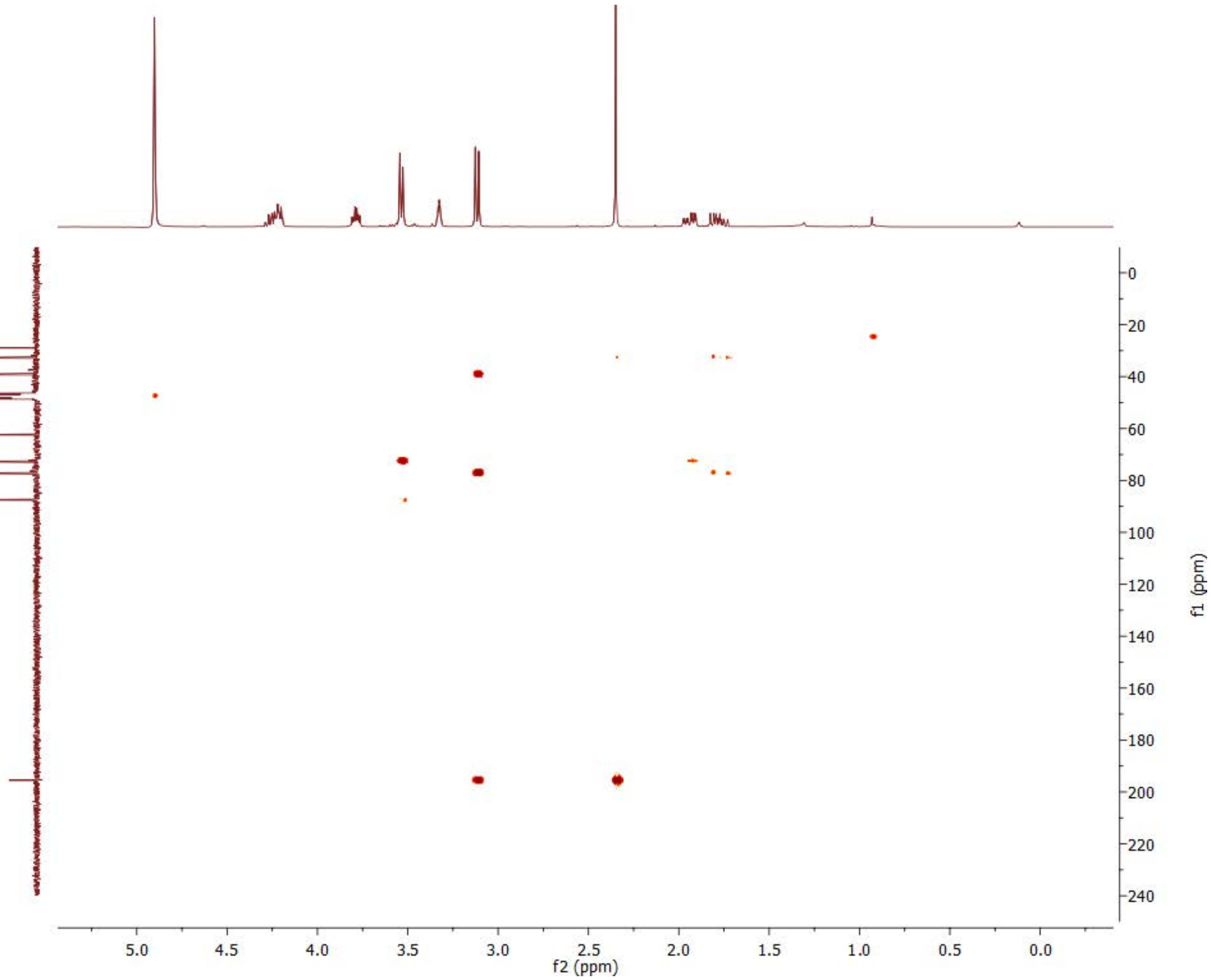

1α-(Acetylmercaptomethyl)-1,2-dideoxy-5-*O*-(4,4'-dimethoxytrityl)-D-*erythro*-pentofuranose (15α)

^{1}H NMR (300.3 MHz, MeOH-d_4)

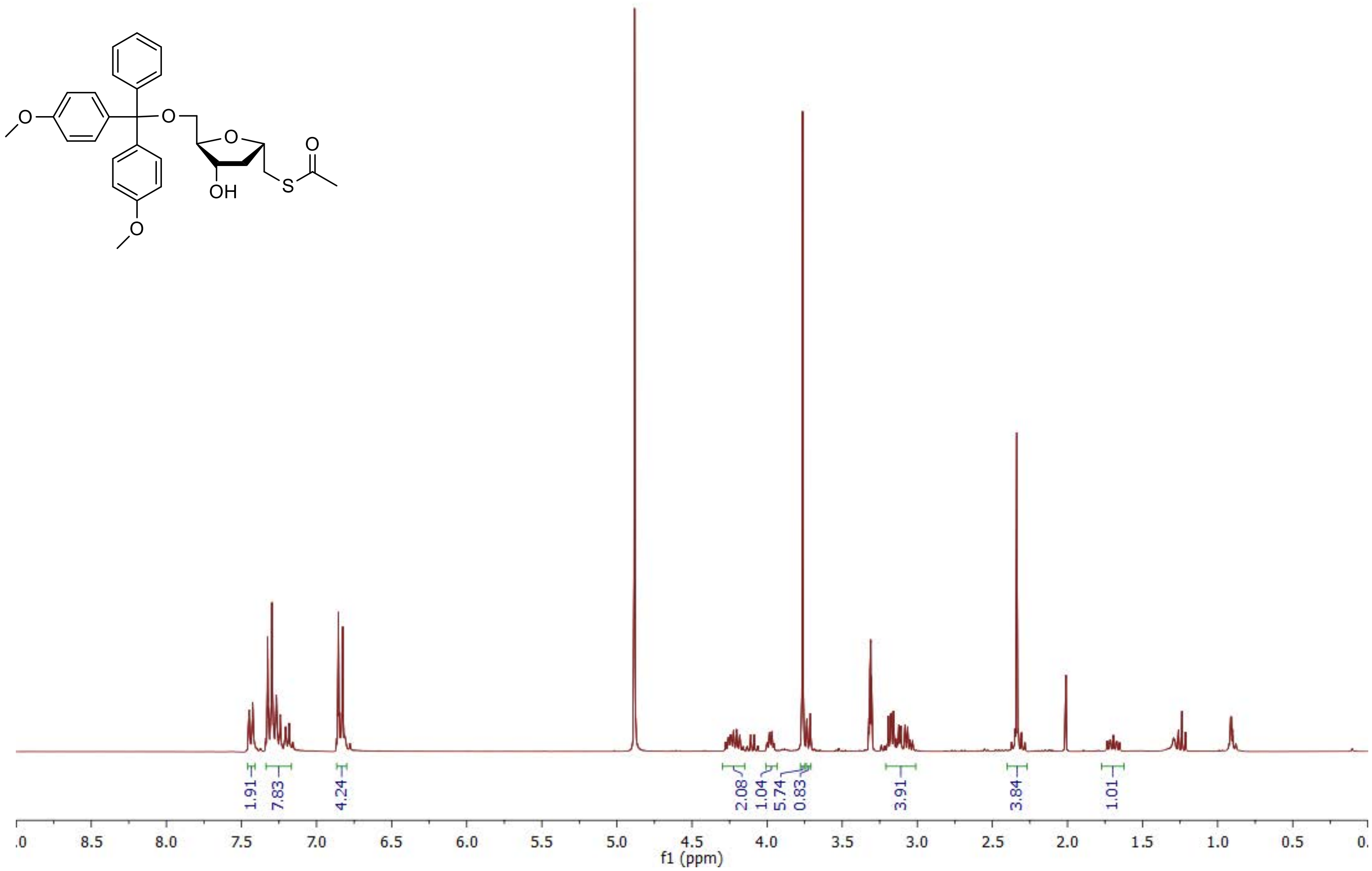

1α-(Acetylmercaptomethyl)-1,2-dideoxy-5-*O*-(4,4'-dimethoxytrityl)-D-*erythro*-pentofuranose (15α)

^{13}C NMR (75.5 MHz, MeOH-d_4)

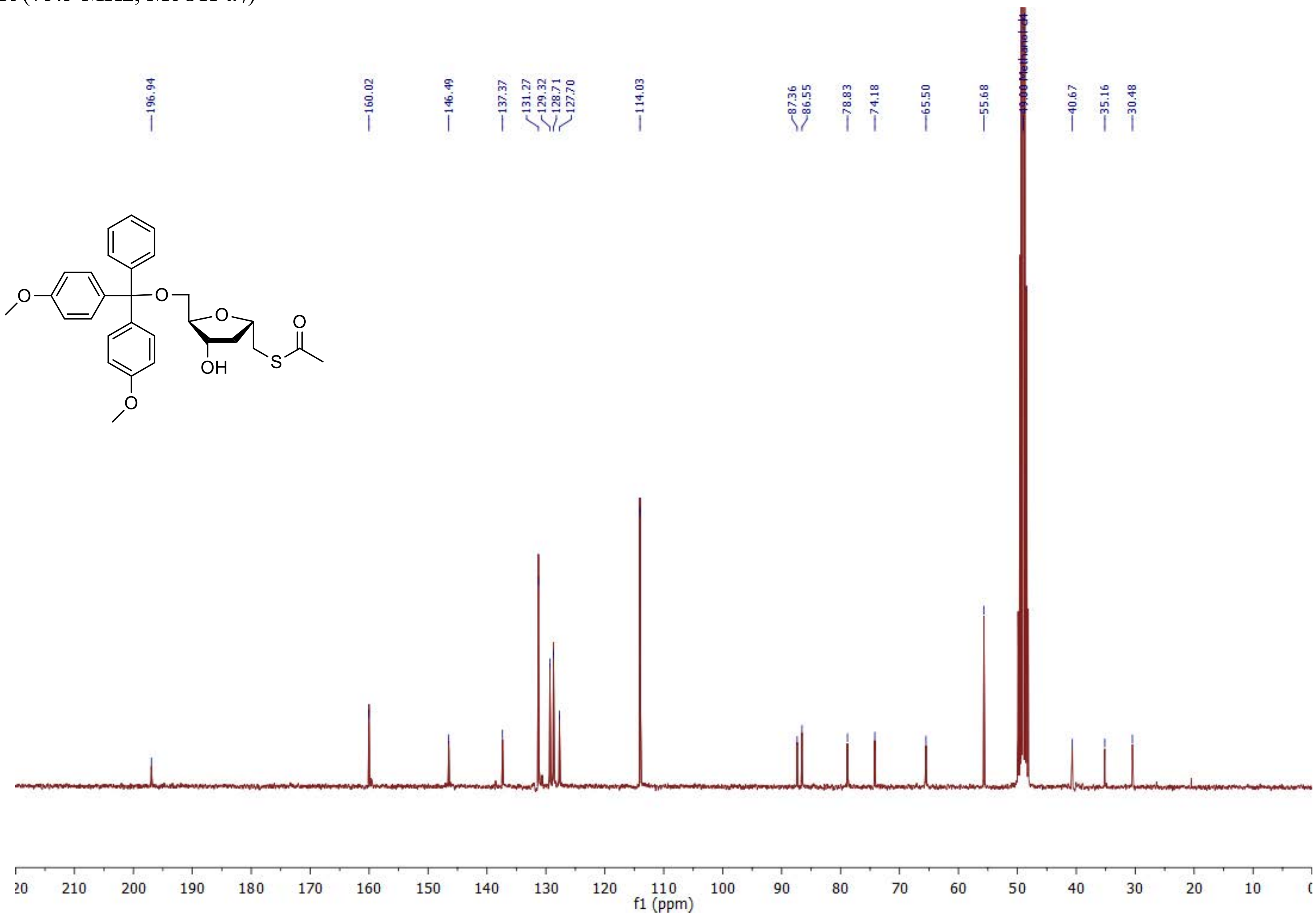

1α-(Acetylmercaptomethyl)-1,2-dideoxy-5-*O*-(4,4'-dimethoxytrityl)-D-*erythro*-pentofuranose (15α)

DEPT 135 NMR (75.5 MHz, MeOH-d_4)

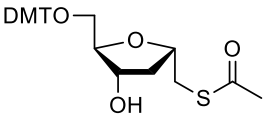

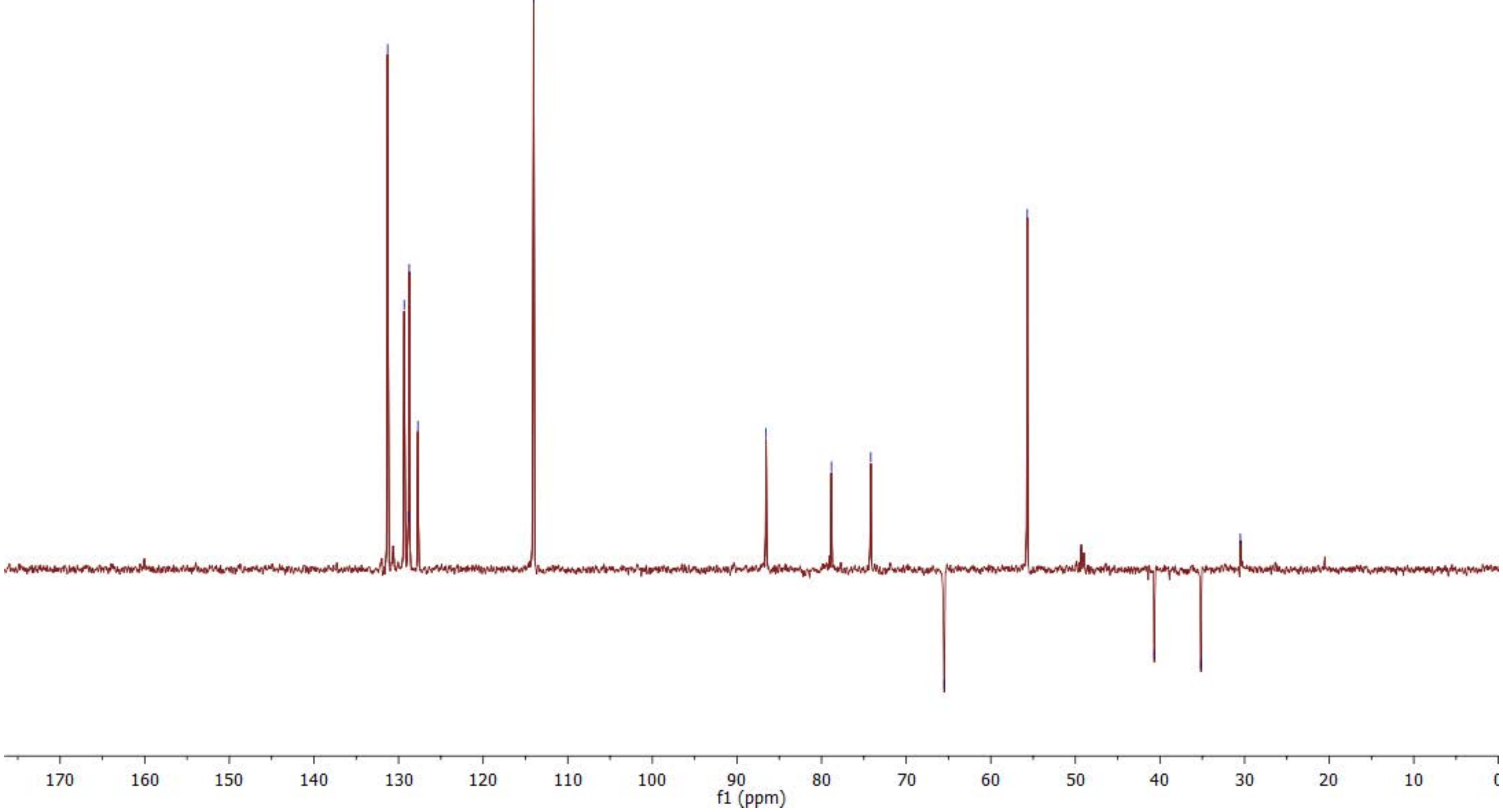

1α-(Acetylmercaptomethyl)-1,2-dideoxy-5-*O*-(4,4'-dimethoxytrityl)-D-*erythro*-pentofuranose (15α)

COSY NMR (MeOH-d_4)

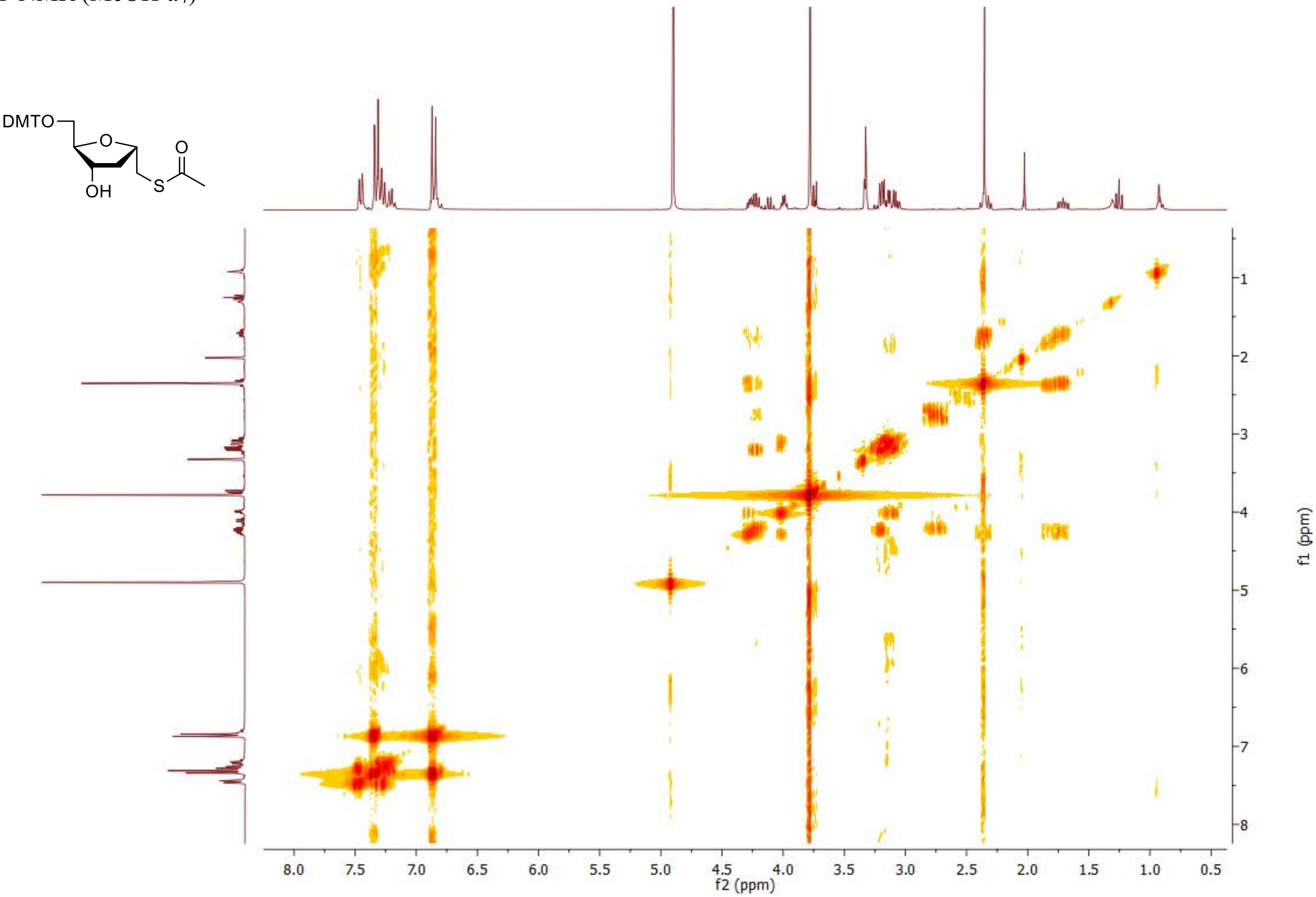

1α-(Acetylmercaptomethyl)-1,2-dideoxy-5-*O*-(4,4'-dimethoxytrityl)-D-*erythro*-pentofuranose (15α)

HSQC NMR (MeOH-d_4)

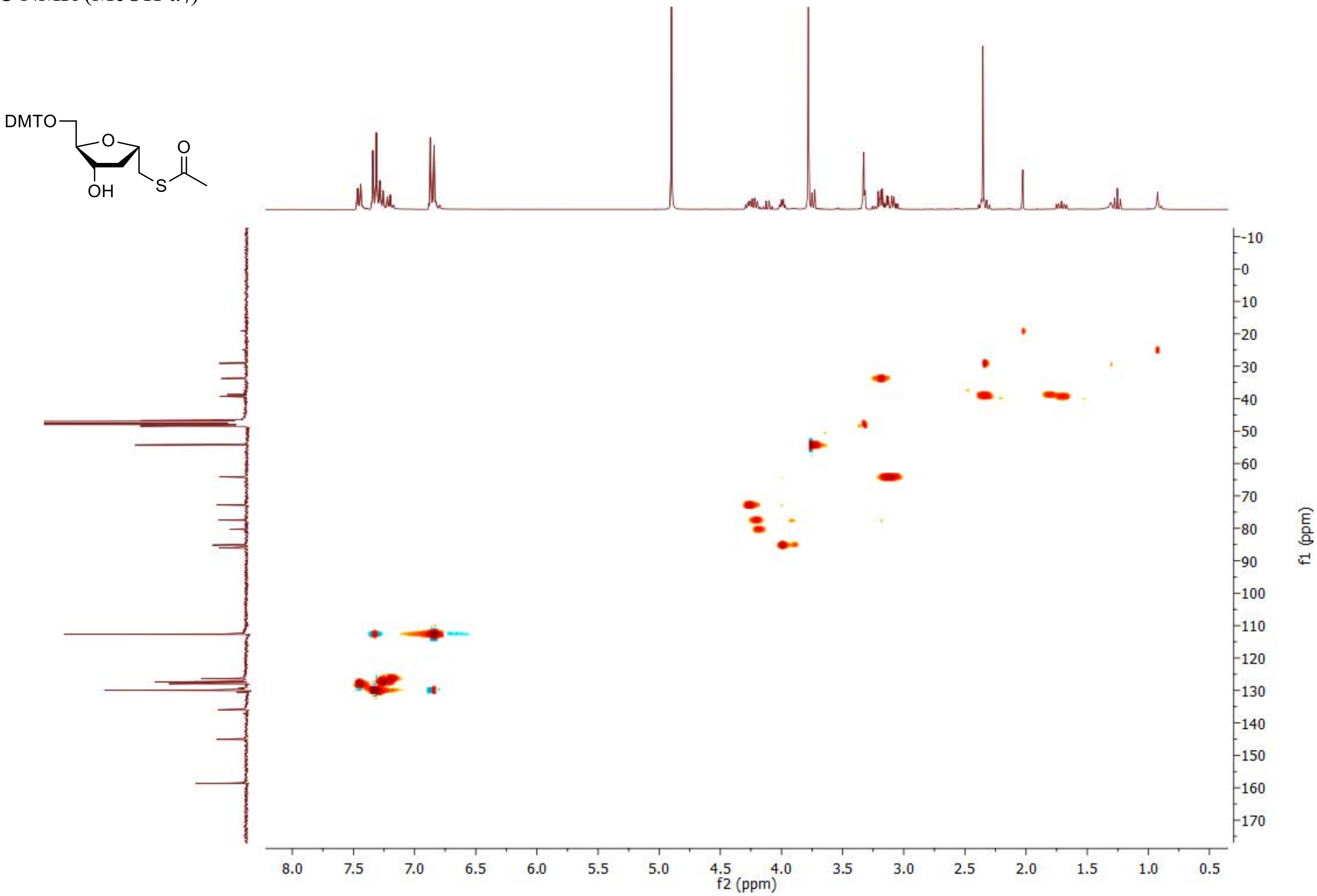

1α-(Acetylmercaptomethyl)-1,2-dideoxy-5-*O*-(4,4'-dimethoxytrityl)-D-*erythro*-pentofuranose (15α)

HMBC NMR (MeOH-d_4)

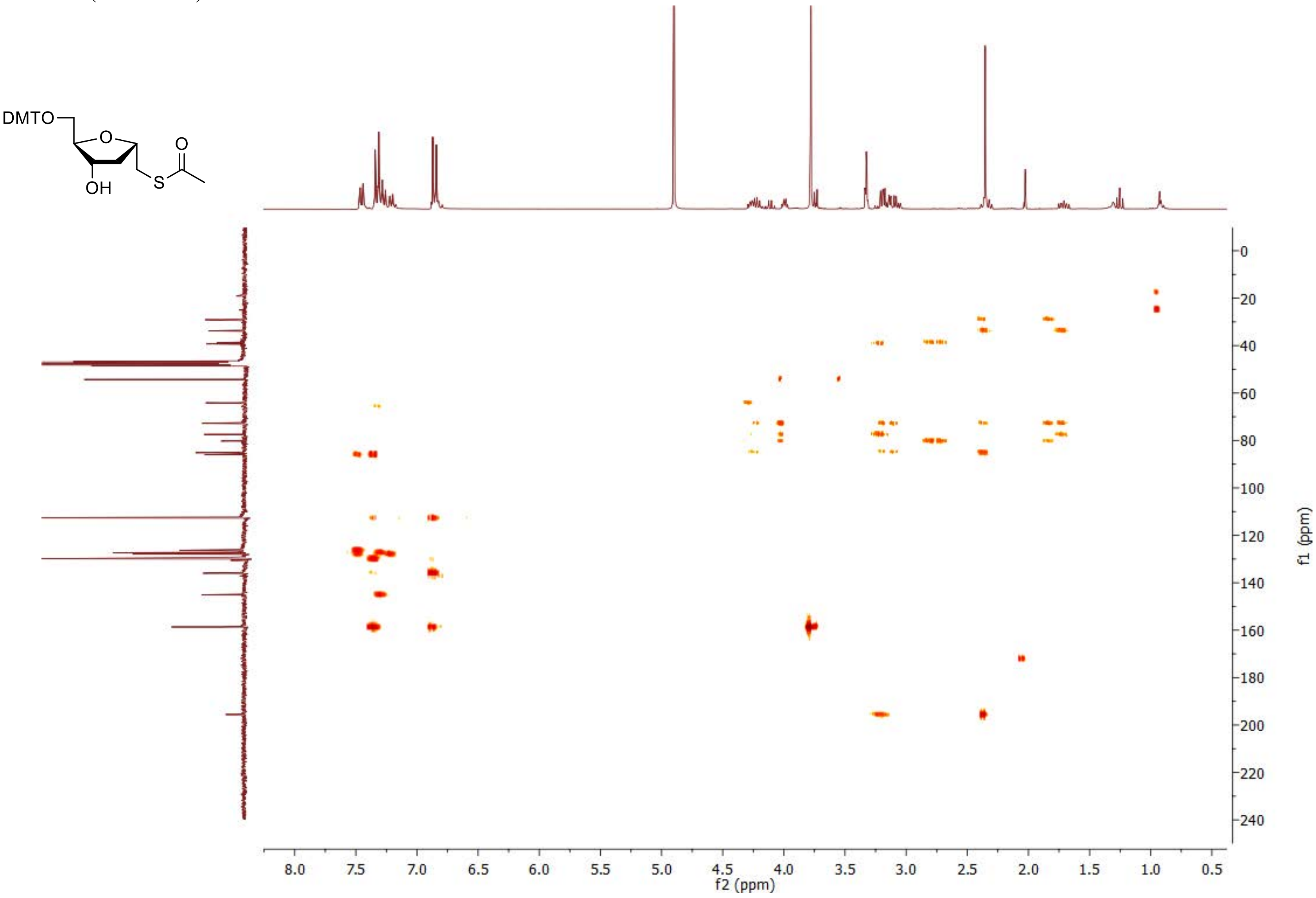

1β-(Acetylmercaptomethyl)-1,2-dideoxy-5-*O*-(4,4’-dimethoxytrityl)-D-*erythro*-pentofuranose (15β)

^{1}H NMR (300.13 MHz, MeOH-d_4)

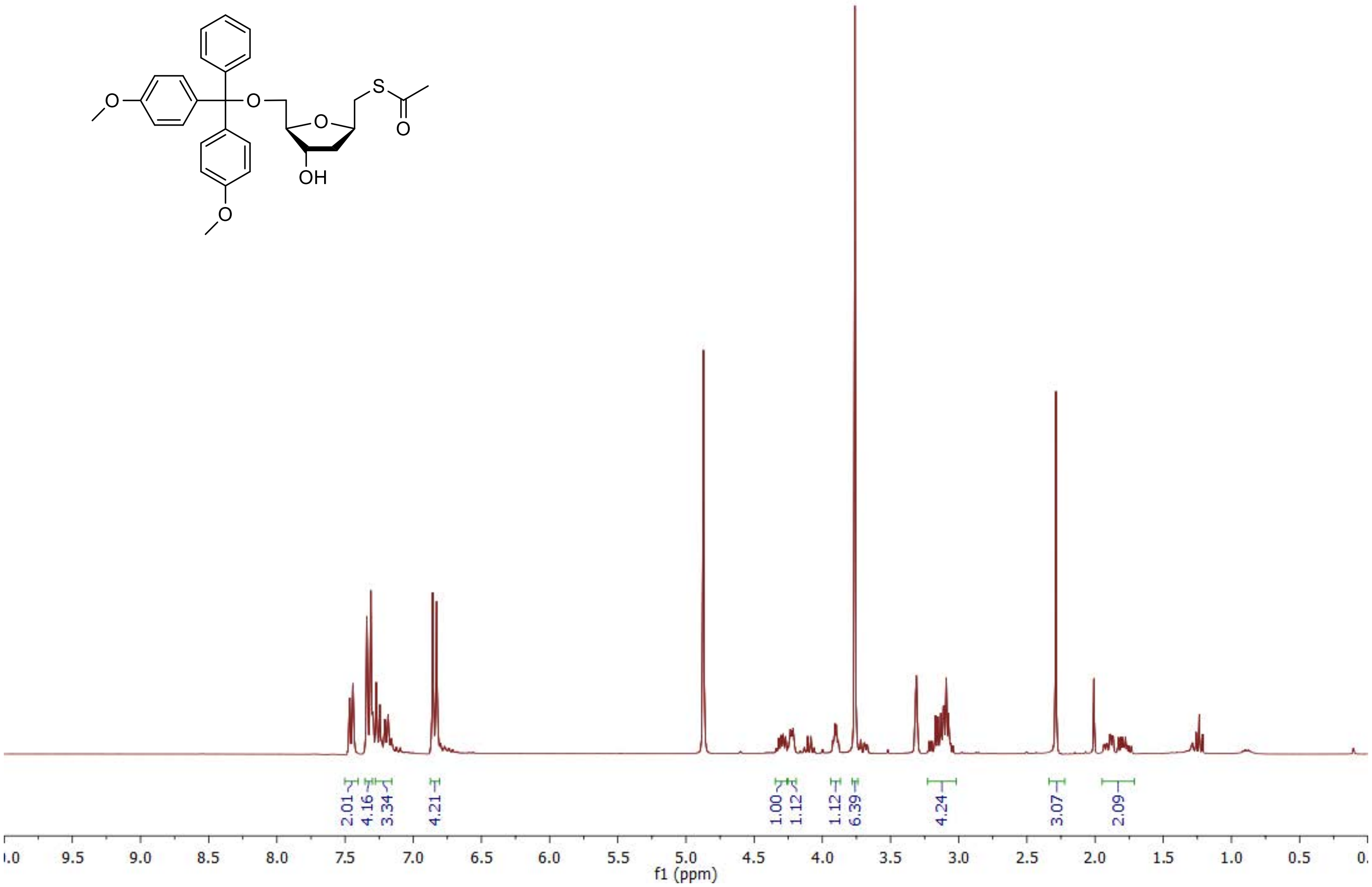

1β-(Acetylmercaptomethyl)-1,2-dideoxy-5-*O*-(4,4'-dimethoxytrityl)-ᴅ-*erythro*-pentofuranose (15β)

^{13}C NMR (75.5 MHz, MeOH-d_4)

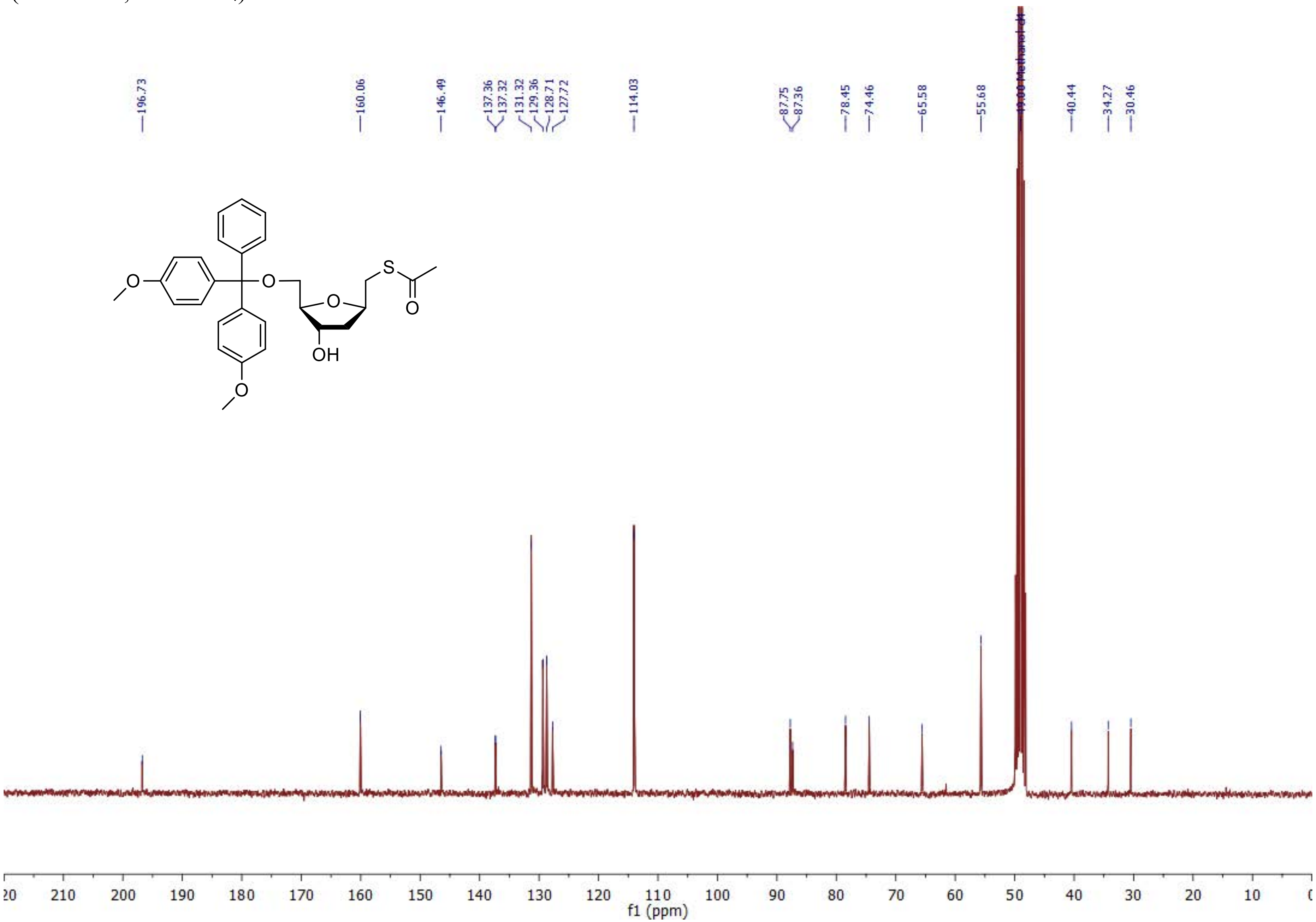

1β-(Acetylmercaptomethyl)-1,2-dideoxy-5-*O*-(4,4'-dimethoxytrityl)-D-*erythro*-pentofuranose (15β)

DEPT 135 NMR (75.5 MHz, MeOH-d_4)

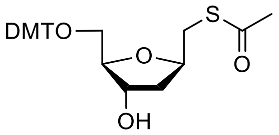

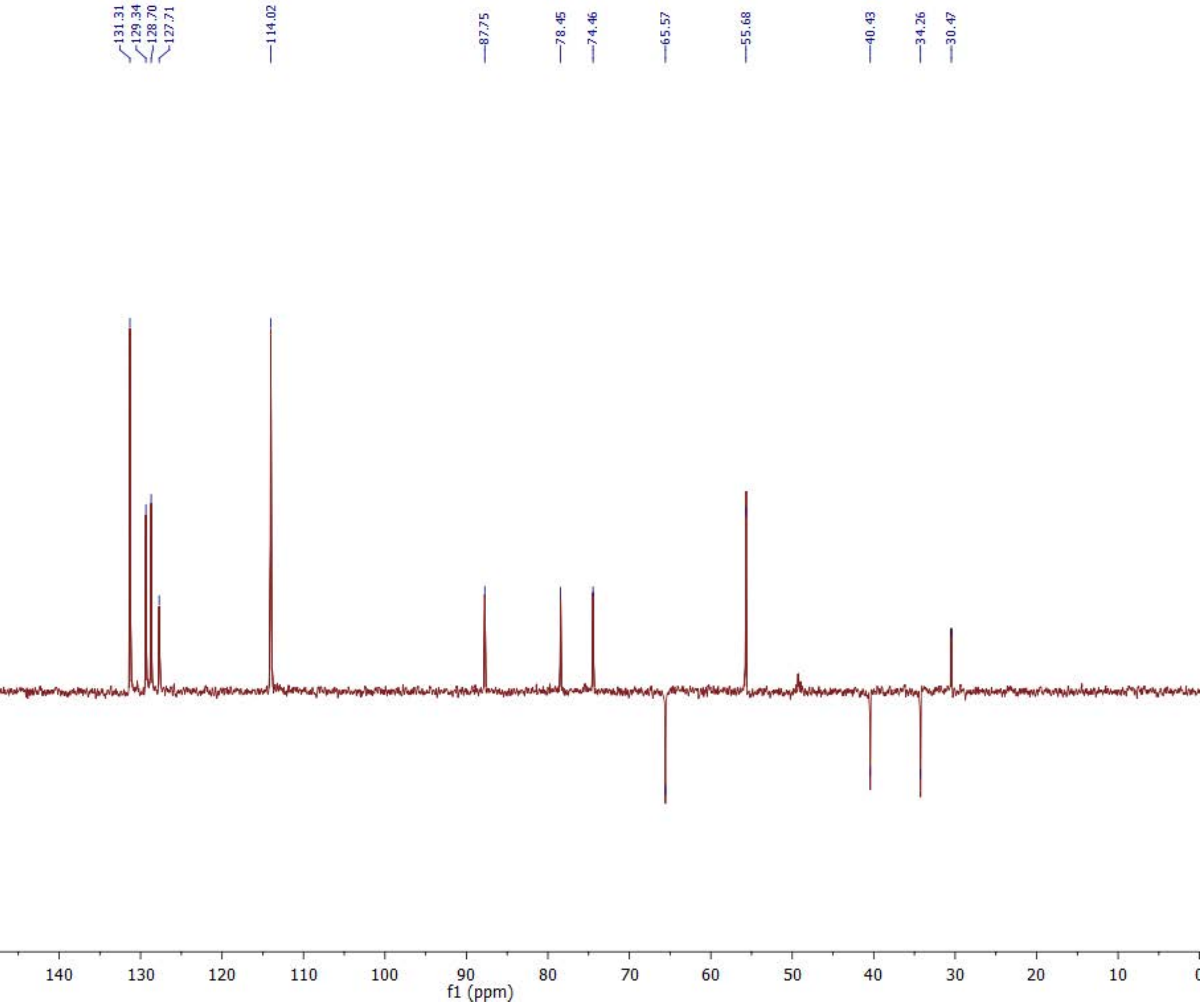

1β-(Acetylmercaptomethyl)-1,2-dideoxy-5-*O*-(4,4’-dimethoxytrityl)-ᴅ-*erythro*-pentofuranose (15β)

COSY NMR (MeOH-d_4)

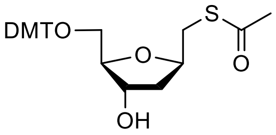

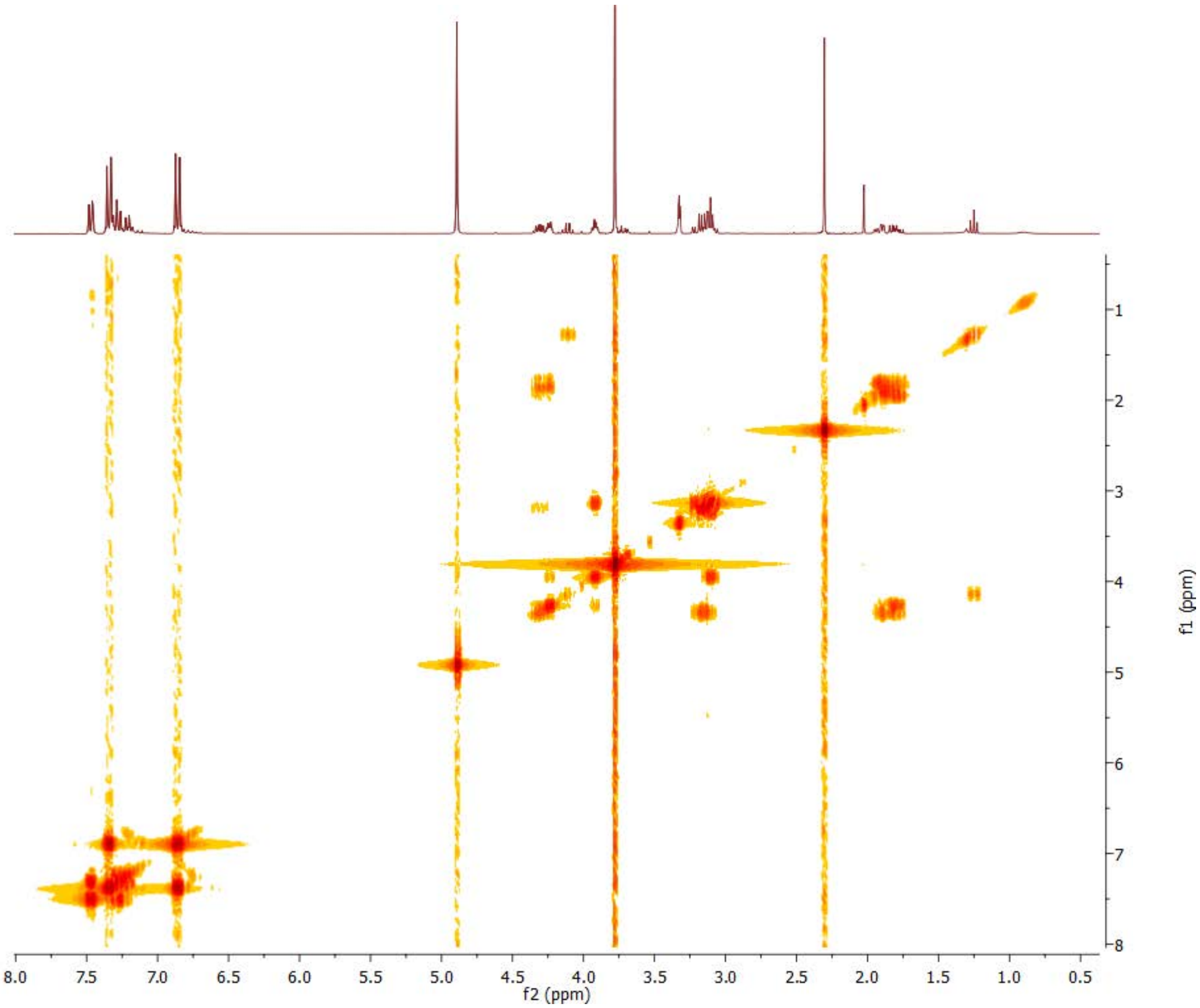

1β-(Acetylmercaptomethyl)-1,2-dideoxy-5-*O*-(4,4’-dimethoxytrityl)-D-*erythro*-pentofuranose (15β)

HSQC NMR (MeOH-d_4)

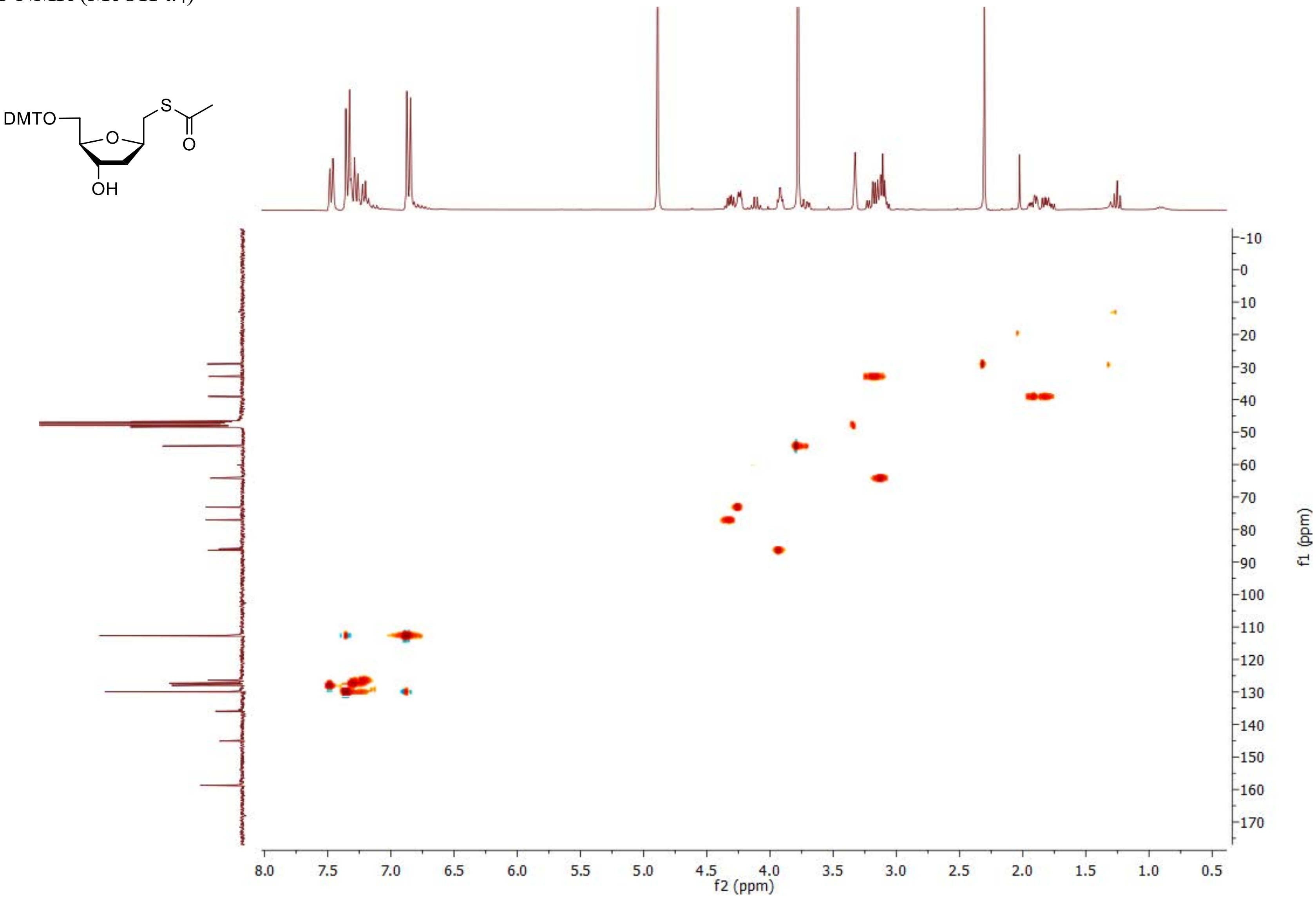

1β-(Acetylmercaptomethyl)-1,2-dideoxy-5-*O*-(4,4'-dimethoxytrityl)-D-*erythro*-pentofuranose (15β)

HMBC NMR (MeOH-d_4)

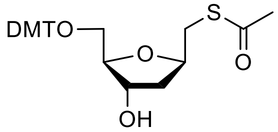

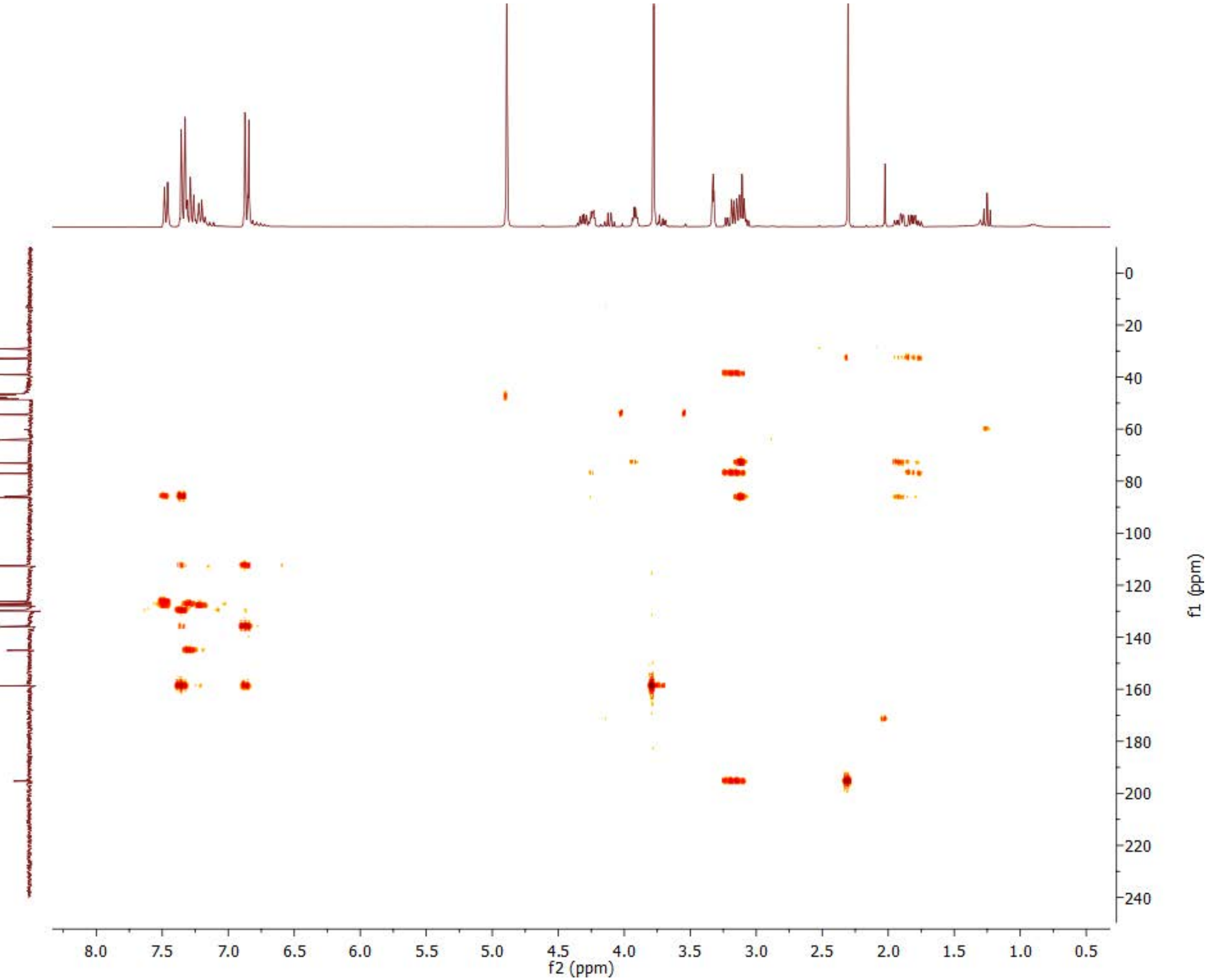

1α-(Acetylmercaptomethyl)-1,2-dideoxy-ᴅ-*erythro*-pentofuranosyl-3-*O*-(2-cyanoethyl-*N,N*-diisopropyl)phosphoramidite (16α-A)

^{1}H NMR (300.3 MHz, MeOH-d_4)

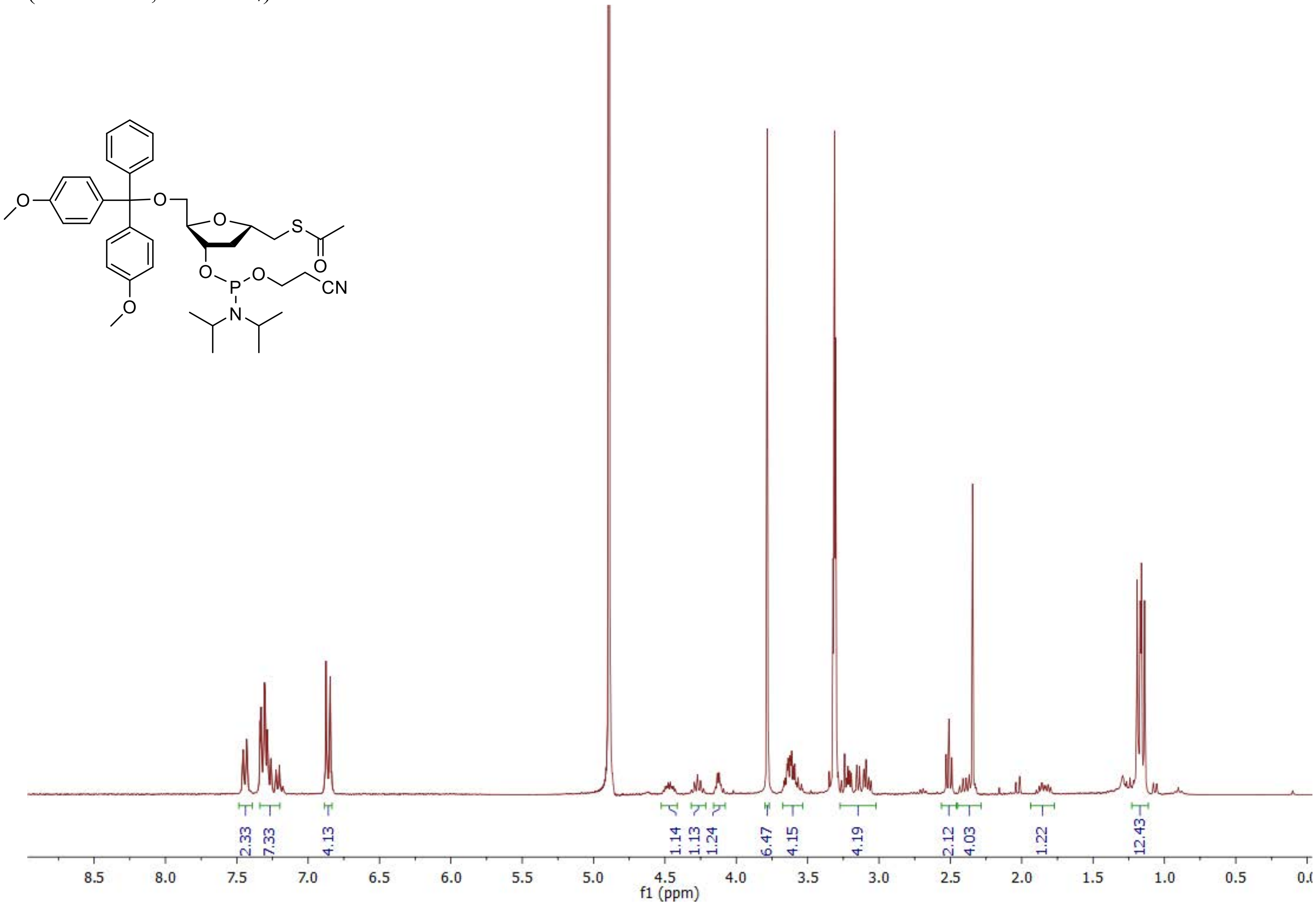

1α-(Acetylmercaptomethyl)-1,2-dideoxy-D-*erythro*-pentofuranosyl-3-*O*-(2-cyanoethyl-*N*,*N*-diisopropyl)phosphoramidite (16α-A)

^{31}P NMR (121.5 MHz, MeOH-d_4)

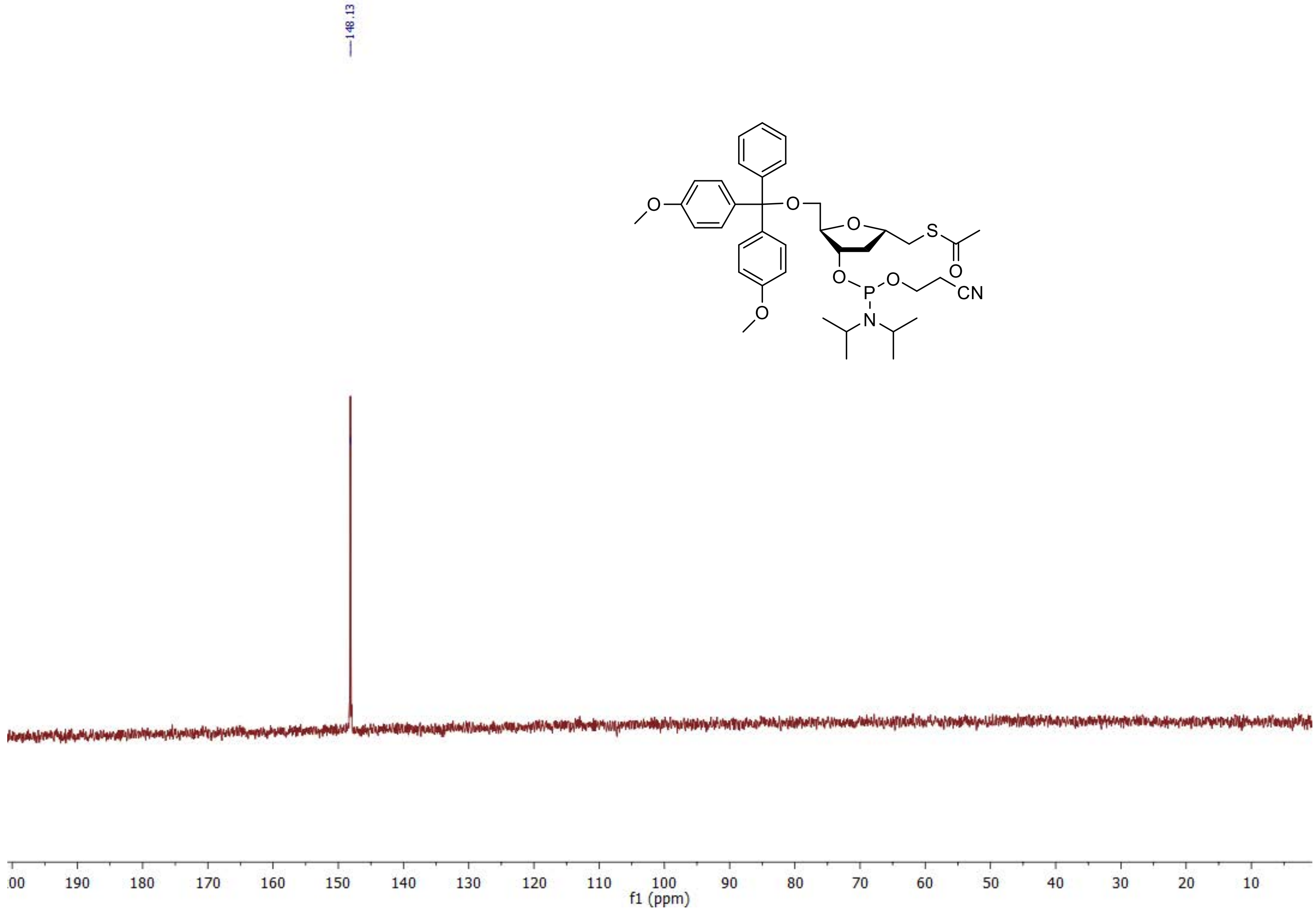

1α-(Acetylmercaptomethyl)-1,2-dideoxy-D-*erythro*-pentofuranosyl-3-*O*-(2-cyanoethyl-*N*,*N*-diisopropyl)phosphoramidite (16α-A+B)

^{1}H NMR (300.3 MHz, MeOH-d_4)

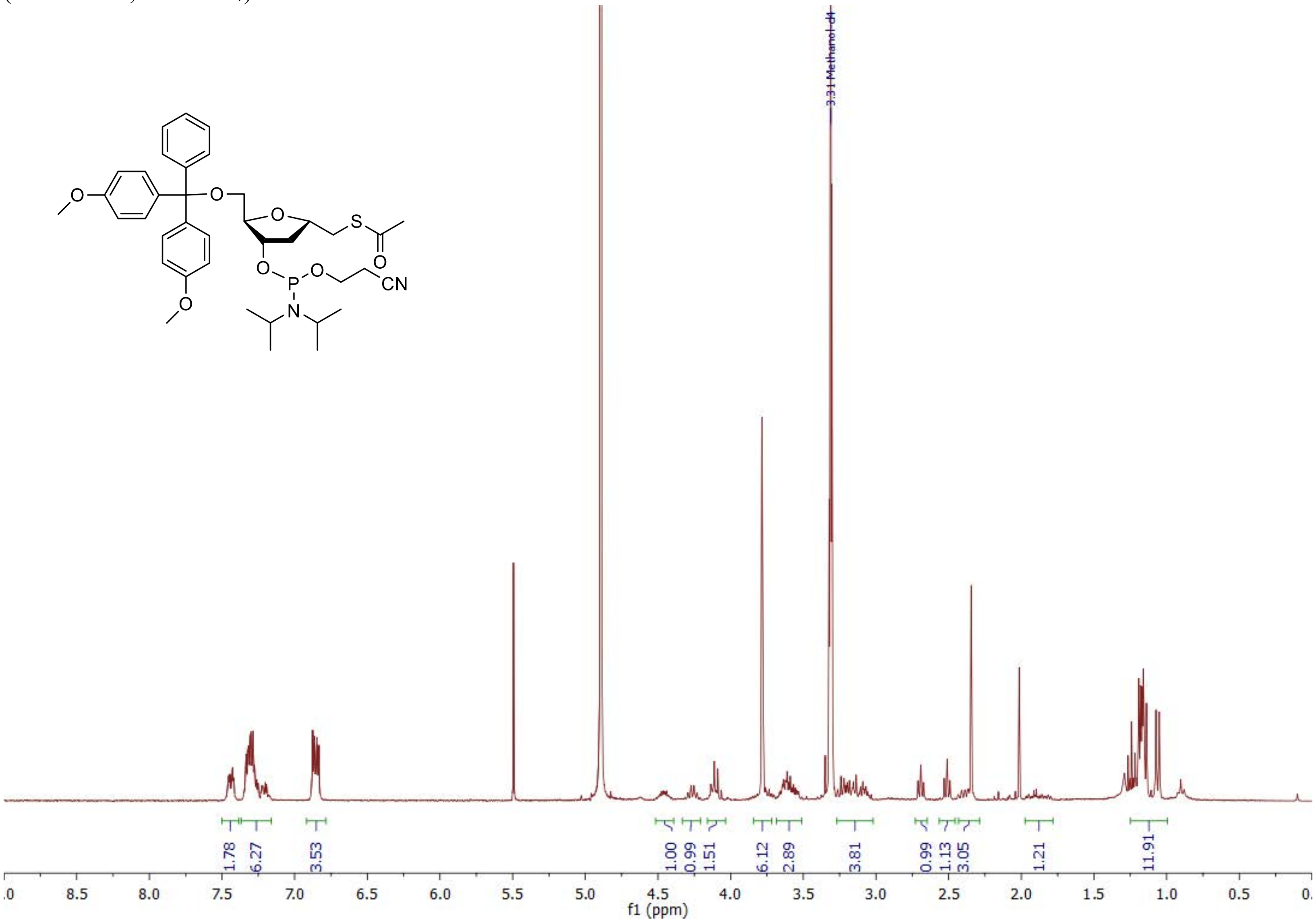

1α-(Acetylmercaptomethyl)-1,2-dideoxy-D-*erythro*-pentofuranosyl-3-*O*-(2-cyanoethyl-*N*,*N*-diisopropyl)phosphoramidite (16α-A+B)

^{31}P NMR (121.5 MHz, MeOH-d_4)

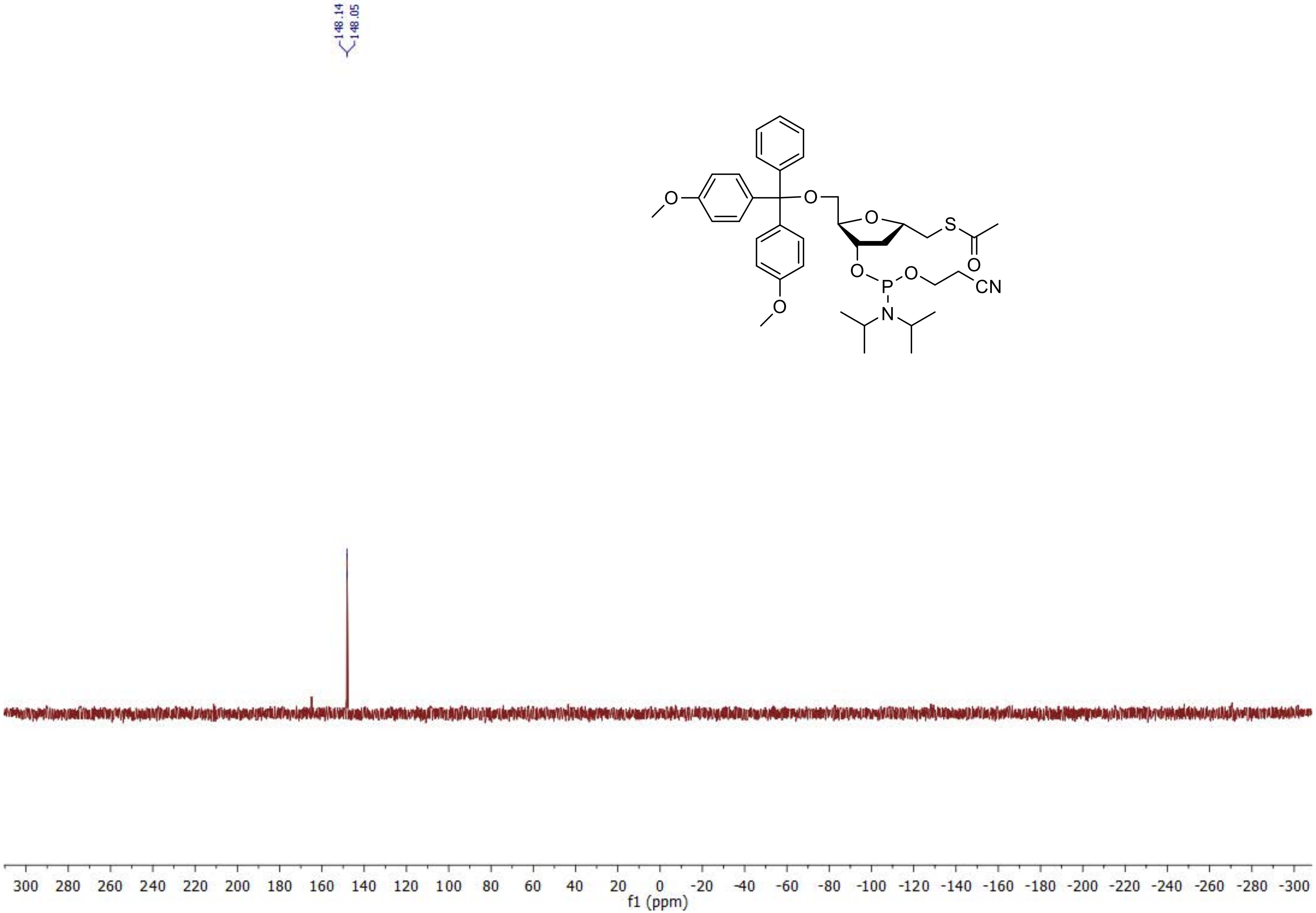

1β-(Acetylmercaptomethyl)-1,2-dideoxy-ᴅ-*erythro*-pentofuranosyl-3-*O*-(2-cyanoethyl-*N,N*-diisopropyl)phosphoramidite (16β-A)

^{1}H NMR (300.13 MHz, MeOH-d_4)

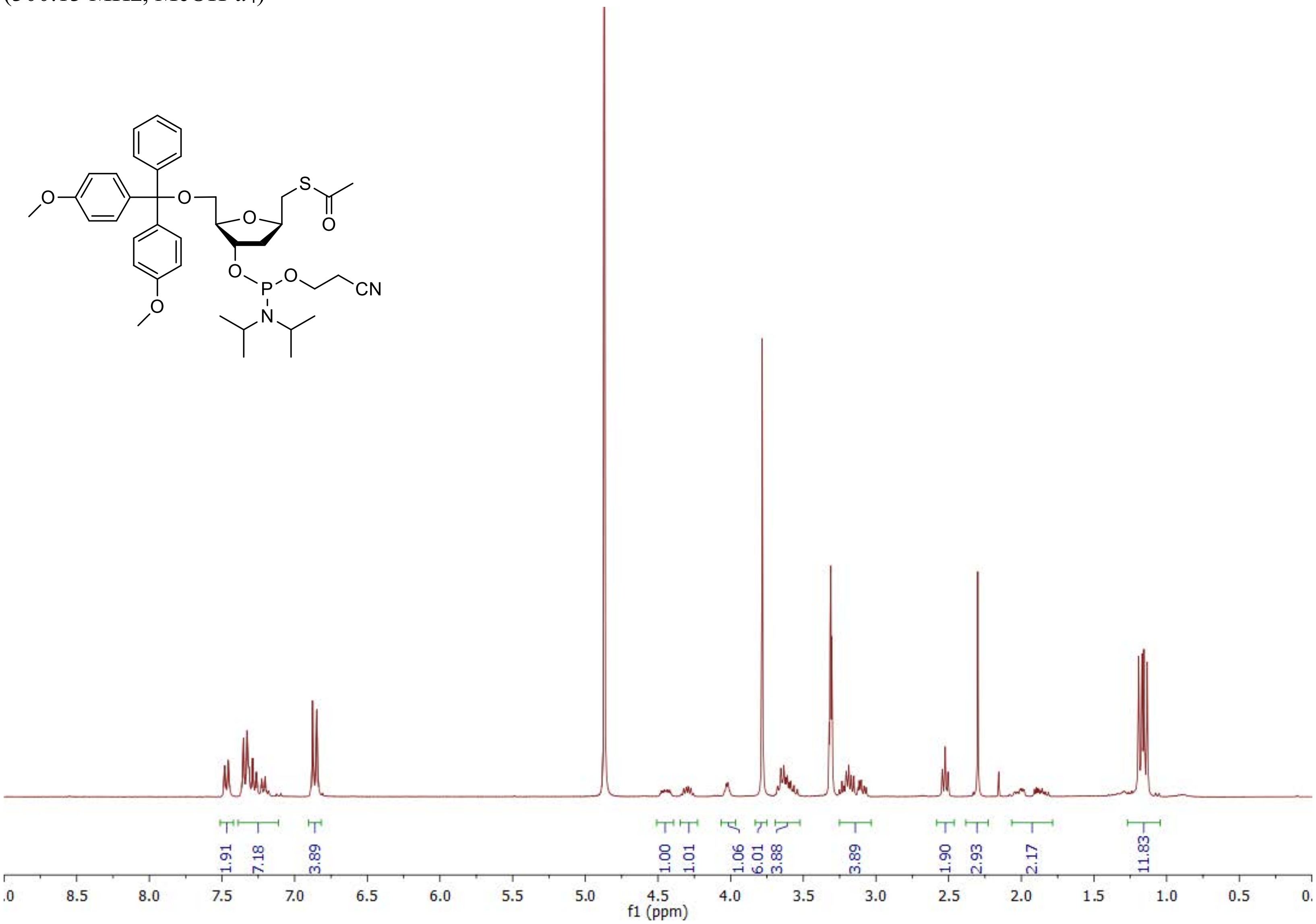

1β-(Acetylmercaptomethyl)-1,2-dideoxy-D-*erythro*-pentofuranosyl-3-*O*-(2-cyanoethyl-*N,N*-diisopropyl)phosphoramidite (16β-A)

^{13}C NMR (75.5 MHz, MeOH-d_4)

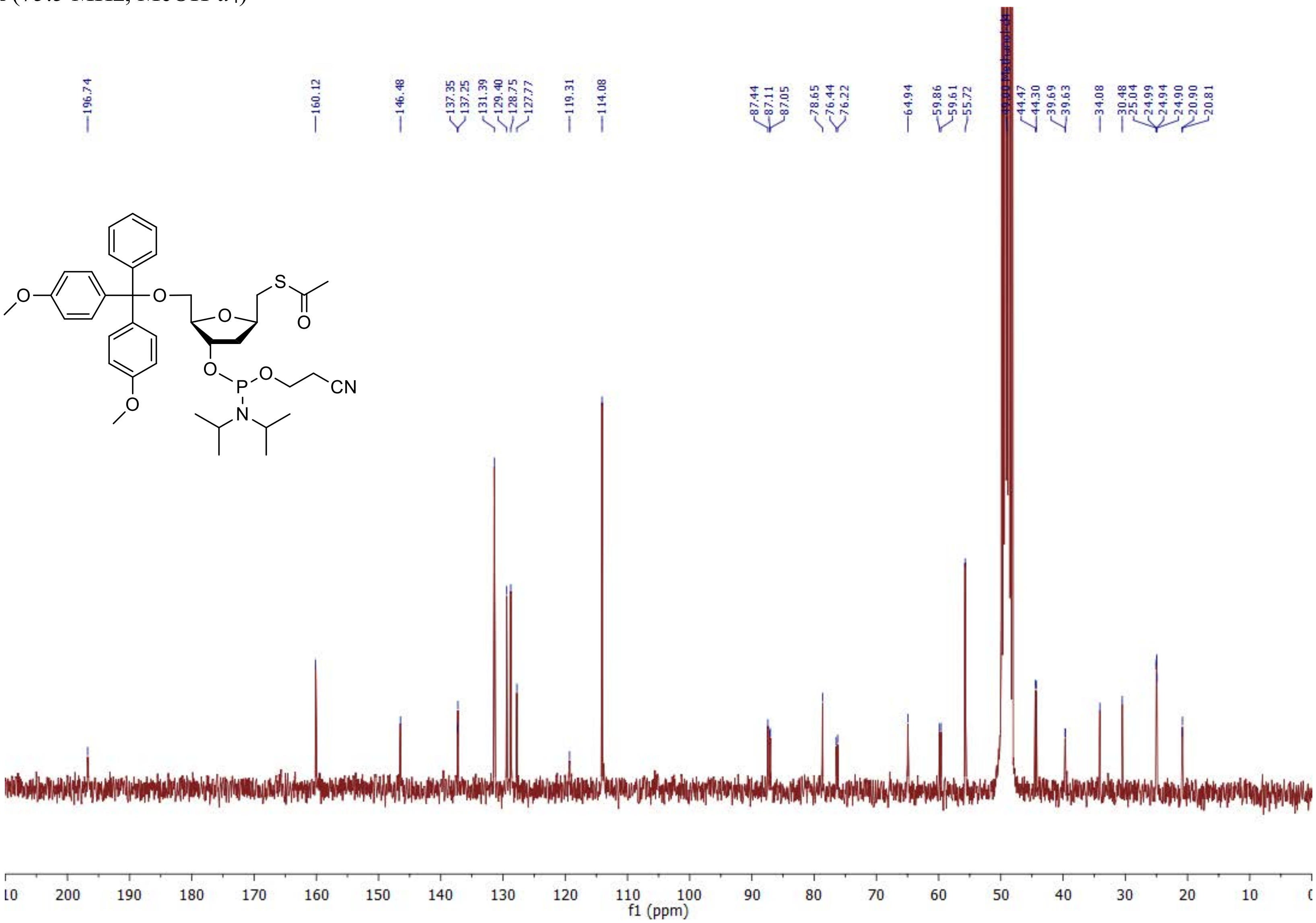

1β-(Acetylmercaptomethyl)-1,2-dideoxy-D-*erythro*-pentofuranosyl-3-*O*-(2-cyanoethyl-*N,N*-diisopropyl)phosphoramidite (16β-A)

DEPT 135 NMR (75.5 MHz, MeOH-d_4)

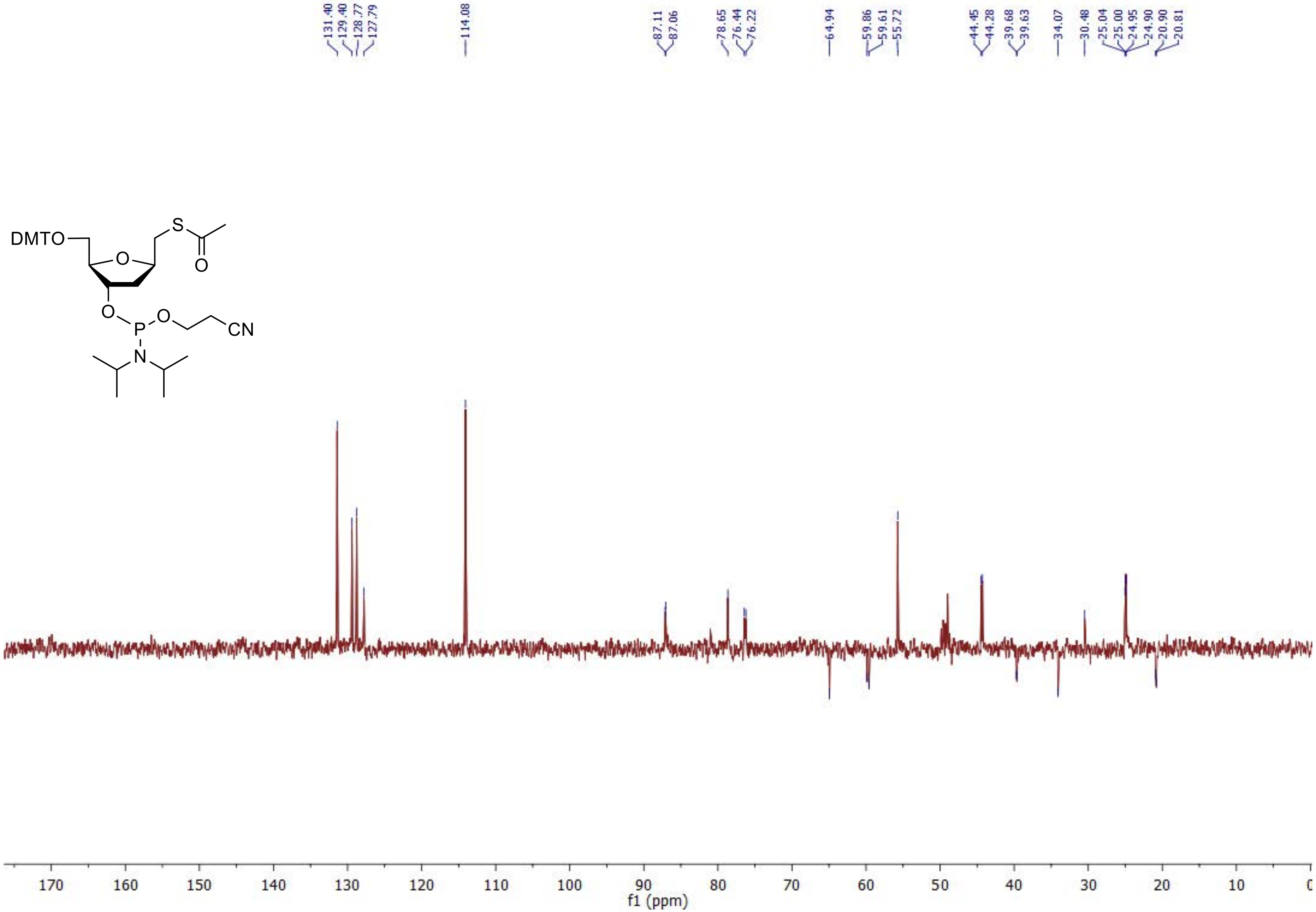

1β-(Acetylmercaptomethyl)-1,2-dideoxy-D-*erythro*-pentofuranosyl-3-*O*-(2-cyanoethyl-*N,N*-diisopropyl)phosphoramidite (16β-A)

COSY NMR (MeOH-d_4)

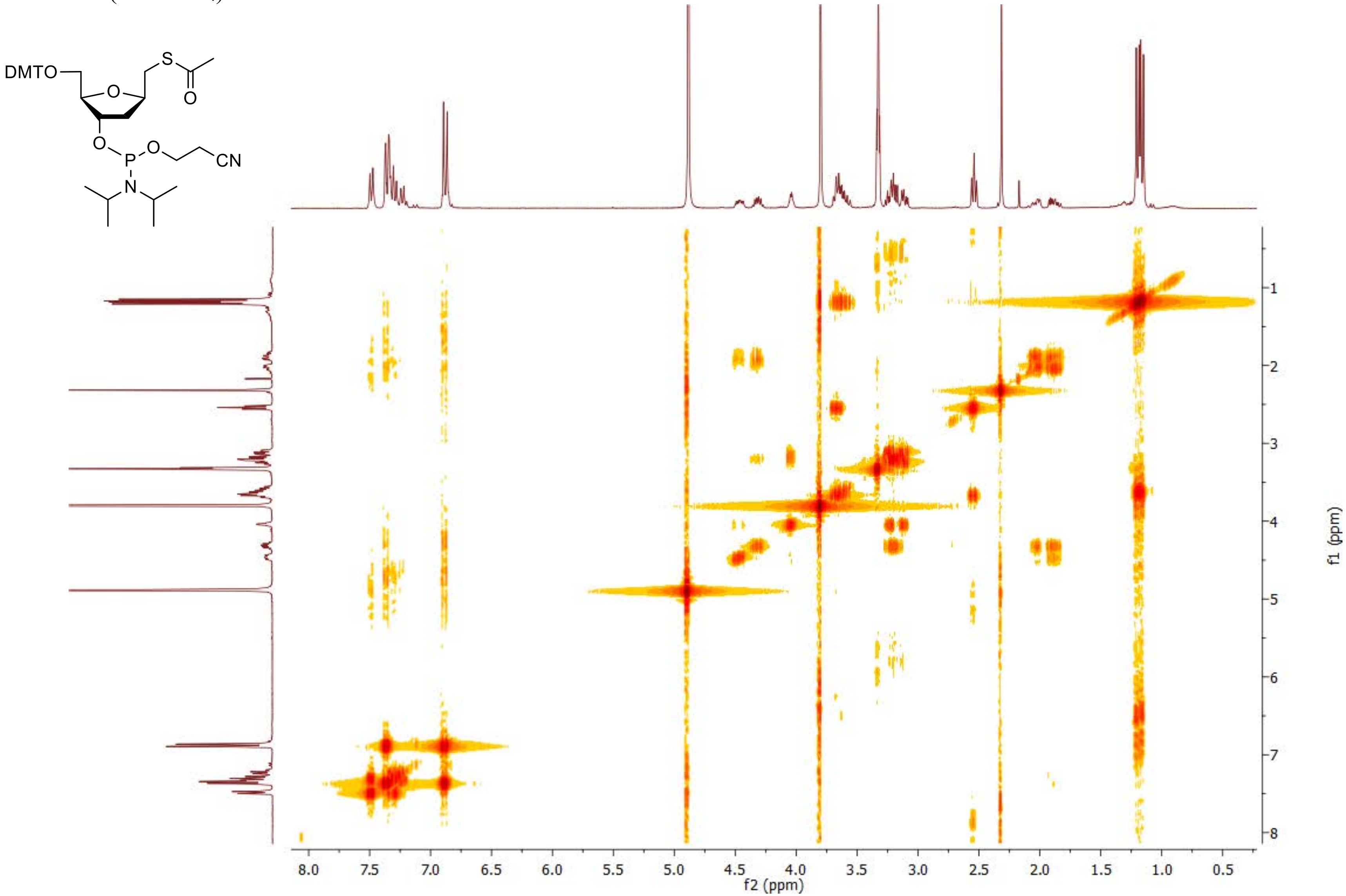

1β-(Acetylmercaptomethyl)-1,2-dideoxy-D-*erythro*-pentofuranosyl-3-*O*-(2-cyanoethyl-*N,N*-diisopropyl)phosphoramidite (16β-A)

HSQC NMR (MeOH-d_4)

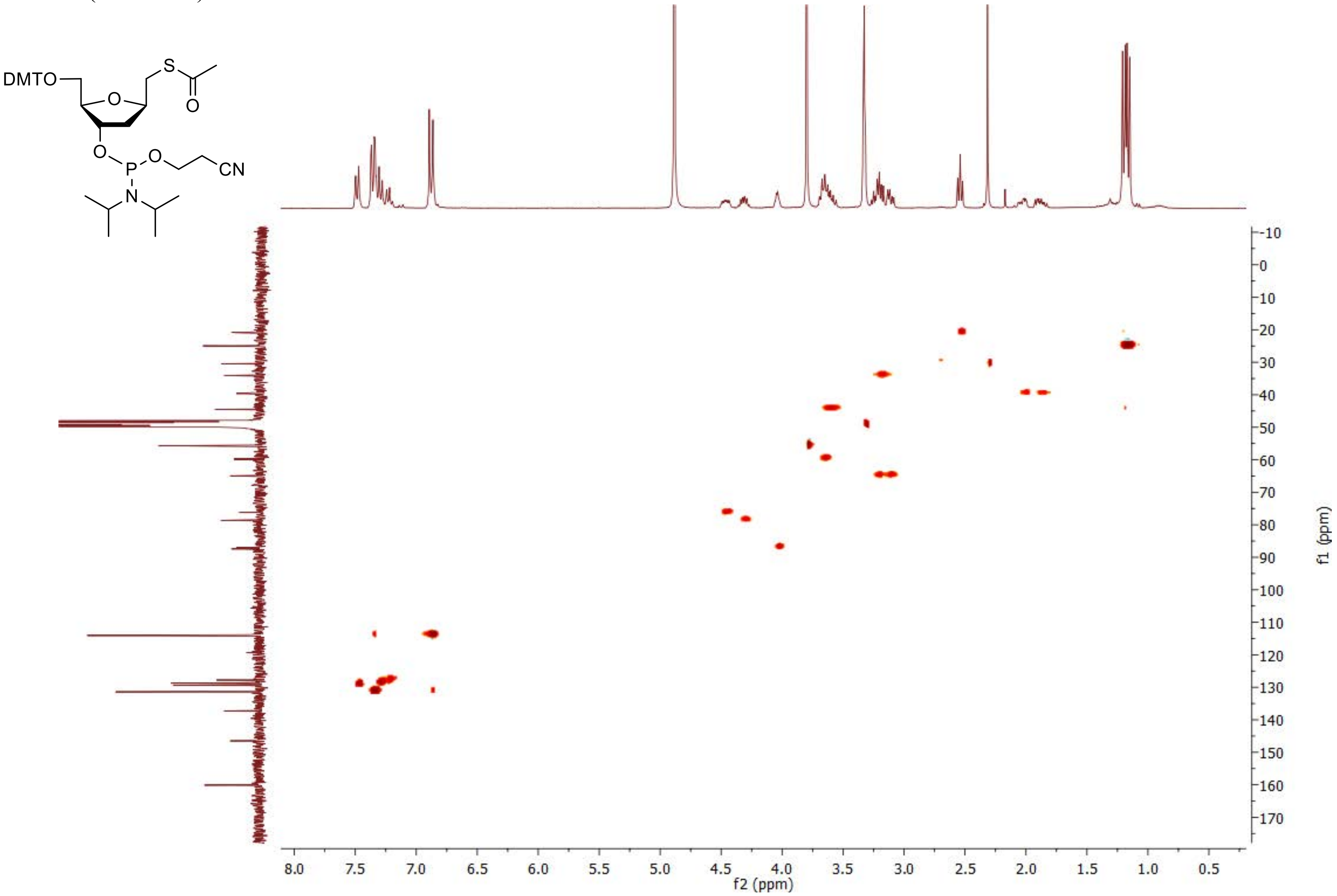

1β-(Acetylmercaptomethyl)-1,2-dideoxy-ᴅ-*erythro*-pentofuranosyl-3-*O*-(2-cyanoethyl-*N,N*-diisopropyl)phosphoramidite (16β-A)

HMBC NMR (MeOH-d_4)

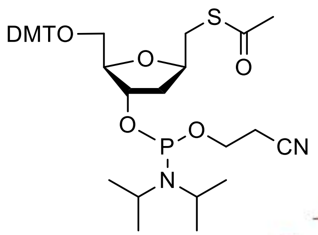

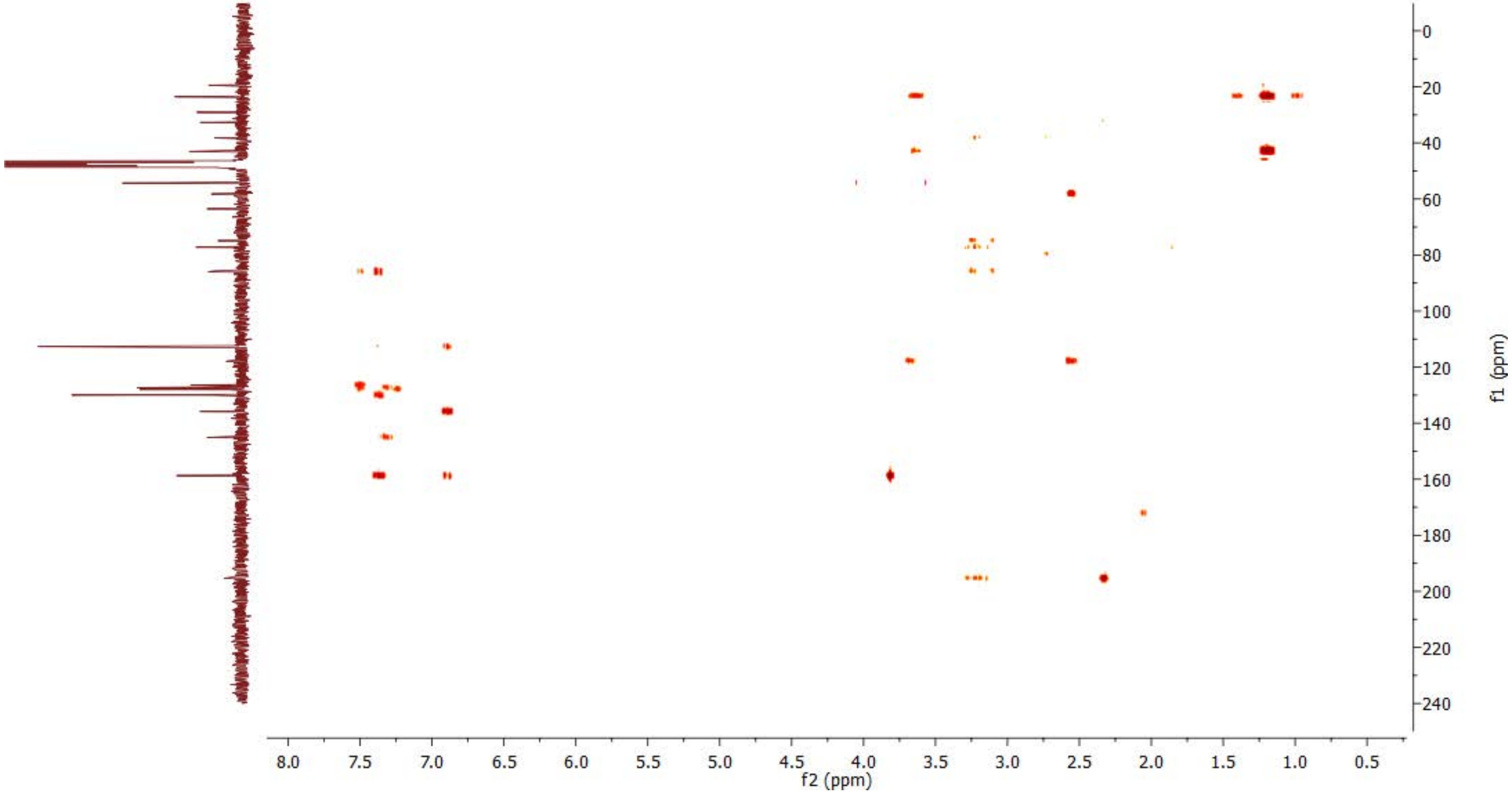

1β-(Acetylmercaptomethyl)-1,2-dideoxy-D-*erythro*-pentofuranosyl-3-*O*-(2-cyanoethyl-*N,N*-diisopropyl)phosphoramidite (16β-A)

^{31}P NMR (121.5 MHz, MeOH-d_4)

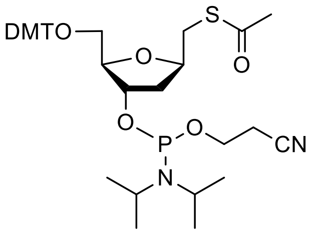

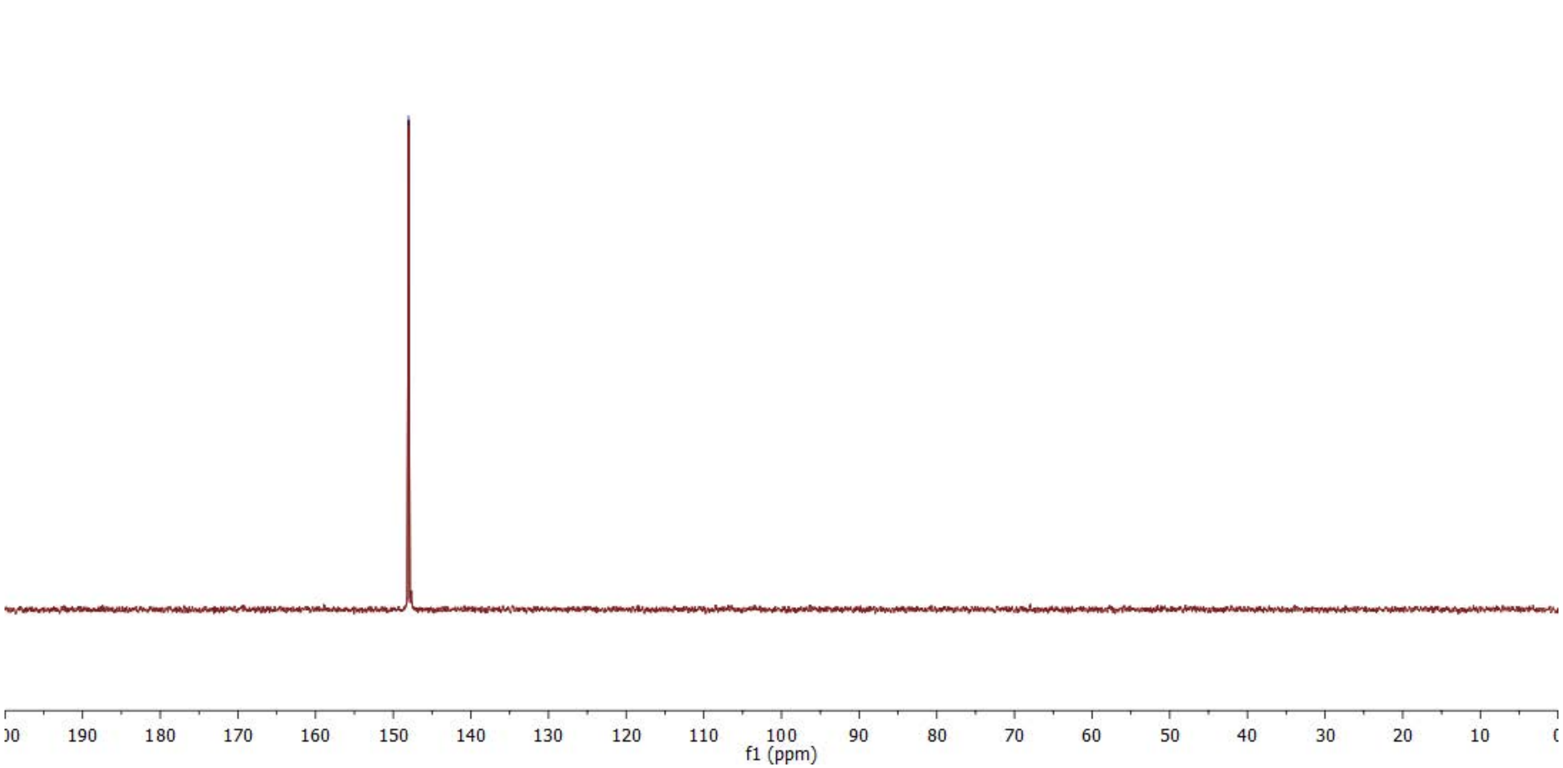

1β-(Acetylmercaptomethyl)-1,2-dideoxy-D-*erythro*-pentofuranosyl-3-*O*-(2-cyanoethyl-*N,N*-diisopropyl)phosphoramidite (16β-B)

^{1}H NMR (300.13 MHz, MeOH-d_4)

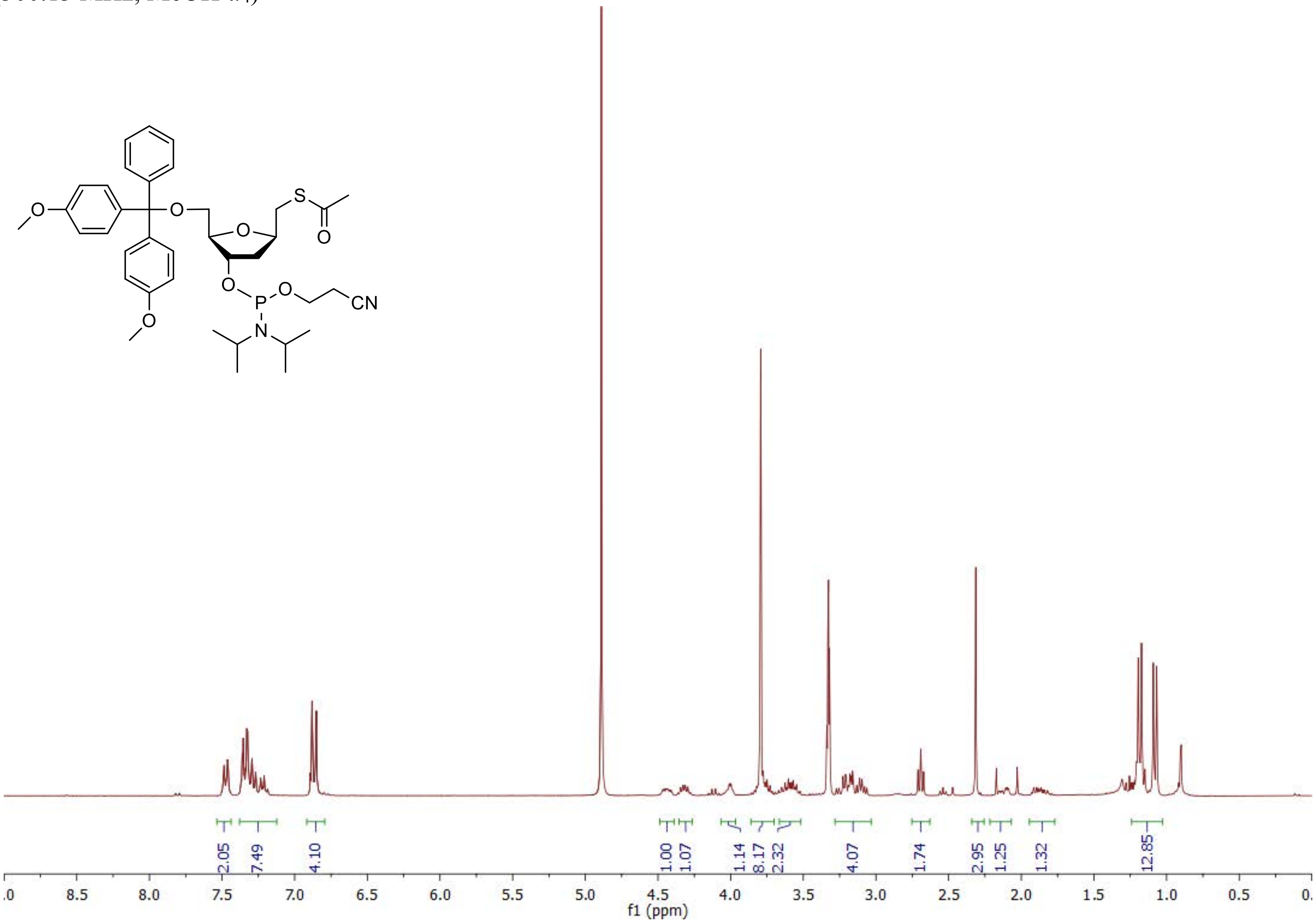

1β-(Acetylmercaptomethyl)-1,2-dideoxy-D-*erythro*-pentofuranosyl-3-*O*-(2-cyanoethyl-*N,N*-diisopropyl)phosphoramidite (16β-B)

^{13}C NMR (75.5 MHz, MeOH-d_4)

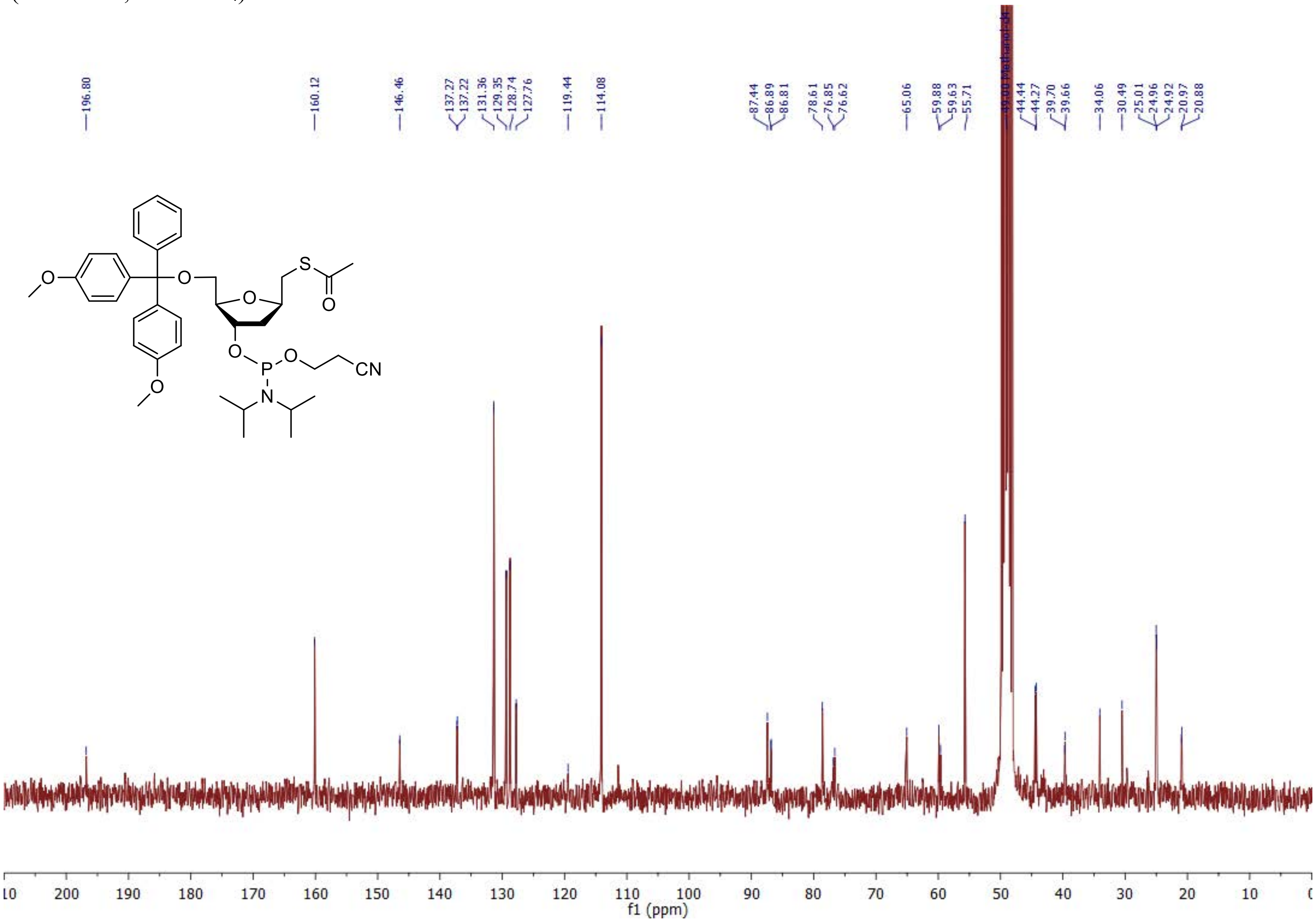

1β-(Acetylmercaptomethyl)-1,2-dideoxy-D-*erythro*-pentofuranosyl-3-*O*-(2-cyanoethyl-*N,N*-diisopropyl)phosphoramidite (16β-B)

COSY NMR (MeOH-d_4)

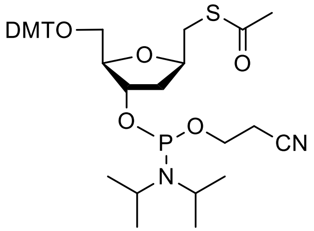

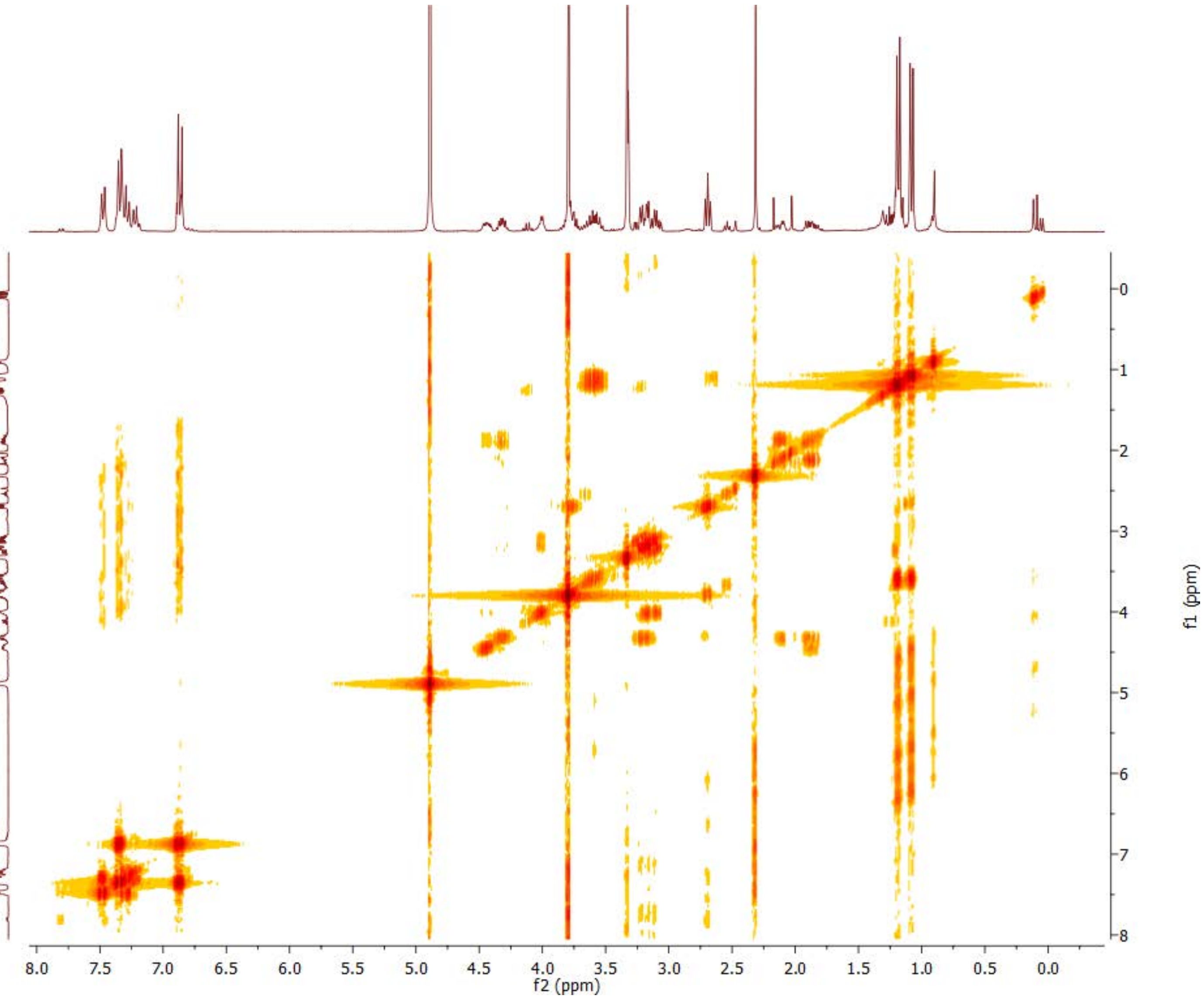

1β-(Acetylmercaptomethyl)-1,2-dideoxy-ᴅ-*erythro*-pentofuranosyl-3-*O*-(2-cyanoethyl-*N,N*-diisopropyl)phosphoramidite (16β-B)

HSQC NMR (MeOH-d_4)

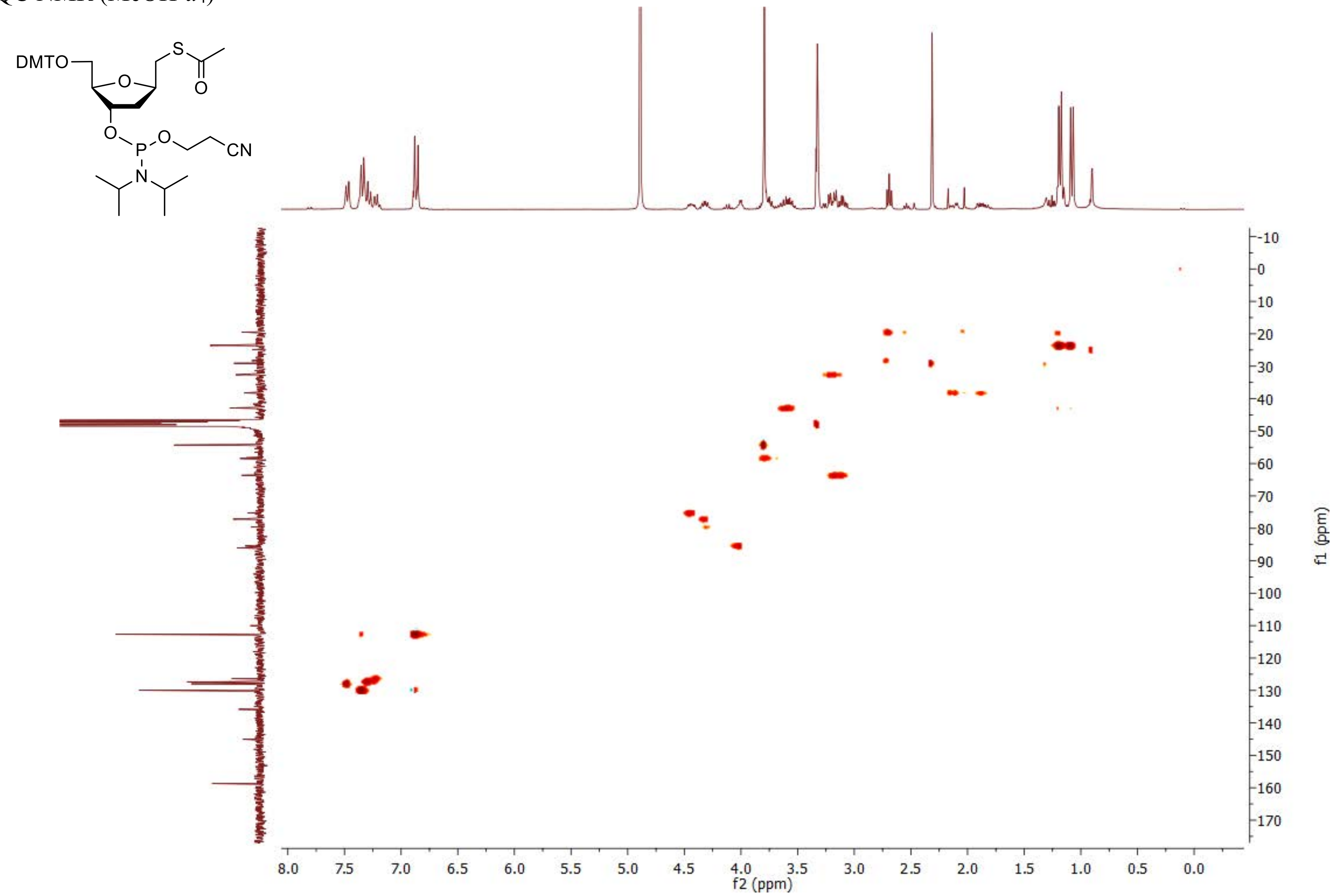

1β-(Acetylmercaptomethyl)-1,2-dideoxy-D-*erythro*-pentofuranosyl-3-*O*-(2-cyanoethyl-*N,N*-diisopropyl)phosphoramidite (16β-B)

HMBC NMR (MeOH-d_4)

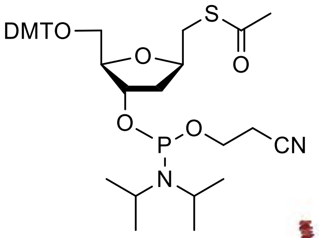

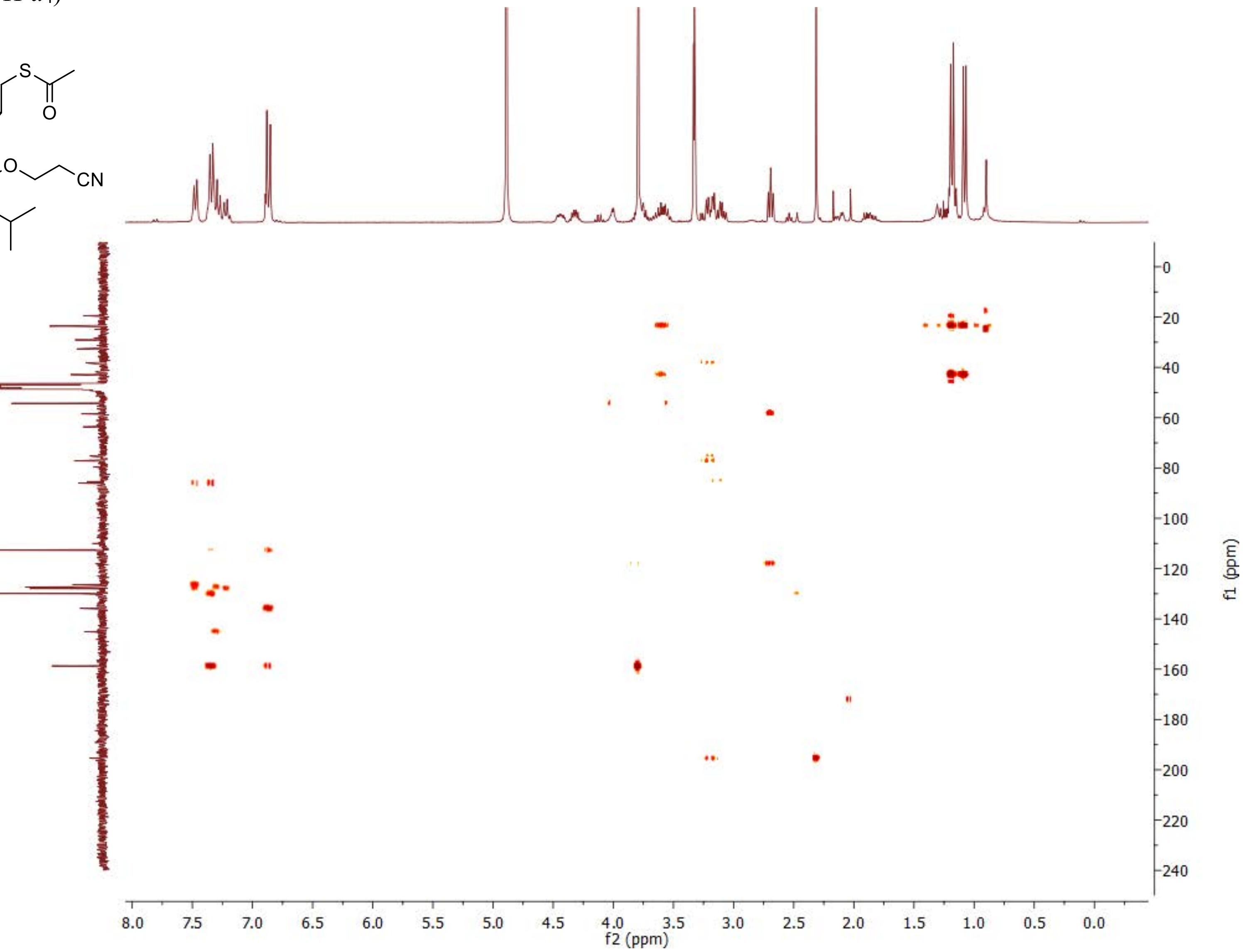

1β-(Acetylmercaptomethyl)-1,2-dideoxy-ᴅ-*erythro*-pentofuranosyl-3-*O*-(2-cyanoethyl-*N,N*-diisopropyl)phosphoramidite (16β-B)

^{31}P NMR (121.5 MHz, MeOH-d_4)

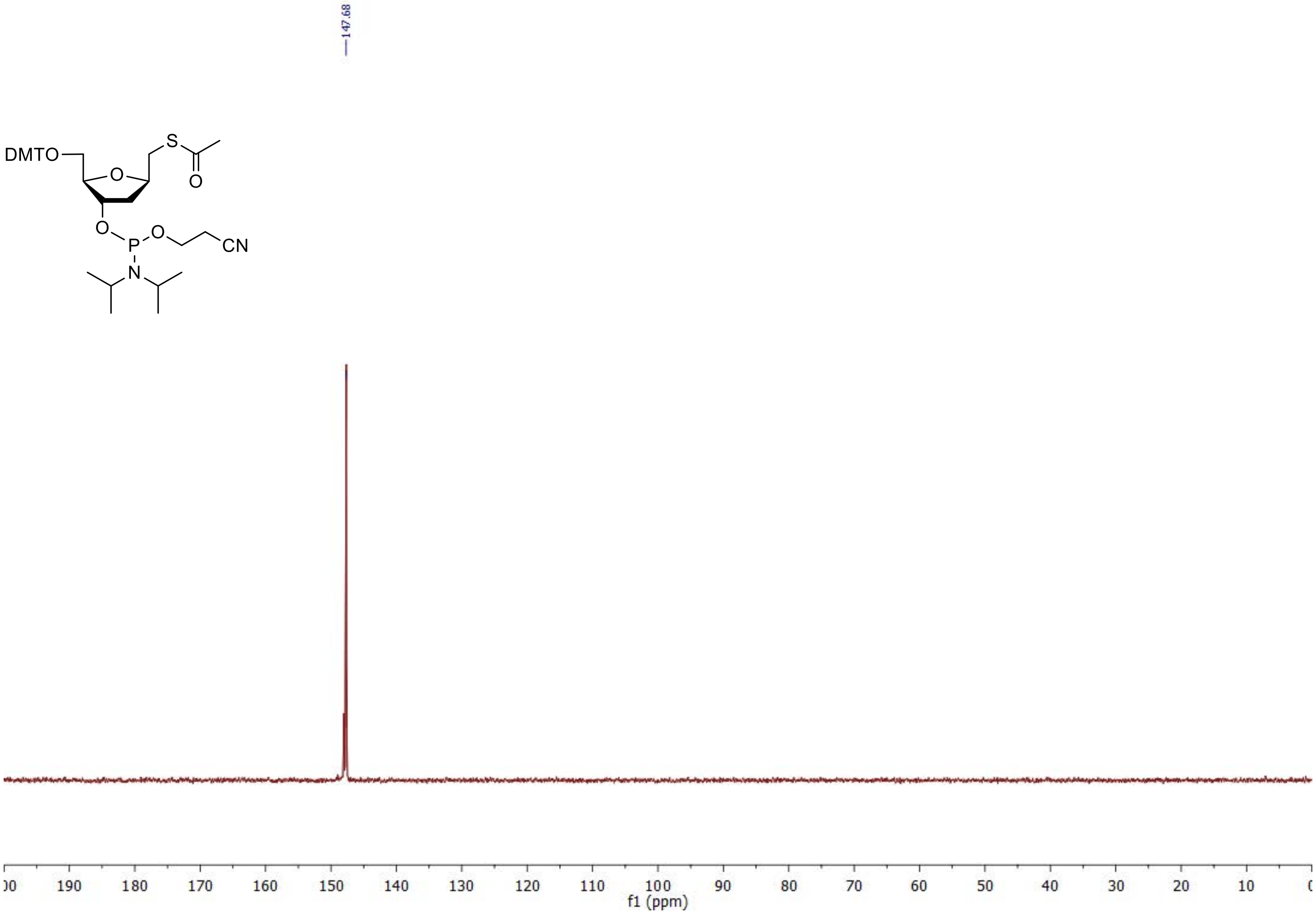

Supplement: Supplementary file 1 — bc0c00717_si_001.pdf [file bc0c00717_si_001.pdf]
